# Selective Quadruple C(sp^3^)-F Functionalization of Polyfluoroalkyl Ketones

**DOI:** 10.1016/j.isci.2020.101259

**Published:** 2020-06-11

**Authors:** Ting Xie, Guo-Qiang Wang, Ya-Wen Wang, Weidong Rao, Haiyan Xu, Shuhua Li, Zhi-Liang Shen, Xue-Qiang Chu

**Affiliations:** 1 Institute of Advanced Synthesis, School of Chemistry and Molecular Engineering, Nanjing Tech University, Nanjing 211816, China; 2 Key Laboratory of Mesoscopic Chemistry of Ministry of Education, Institute of Theoretical and Computational Chemistry, School of Chemistry and Chemical Engineering, Nanjing University, Nanjing 210023, China; 3 Jiangsu Provincial Key Lab for the Chemistry and Utilization of Agro-Forest Biomass, College of Chemical Engineering, Nanjing Forestry University, Nanjing 210037, China; 4 School of Environmental and Chemical Engineering, Jiangsu University of Science and Technology, Zhenjiang, Jiangsu 212003, China

**Keywords:** Chemistry, Organic Chemistry, Organic Chemistry Methods

## Abstract

The significance of organofluorine compounds has inspired the establishment of numerous methods for the functionalization of rather inert C-F bonds. Despite advances achieved in the manipulation of C(sp^2^)-F bonds by employing transition-metal catalysts, such as Pd, Rh, Cu, Ni, Ru, and Ir, strategies that address the paucity of effective pathways for selective activation of multiple C(sp^3^)-F bonds remained challenging. In this context, we present an unprecedented coupling-aromatization-cyclization reaction of polyfluorinated ketones with diverse *N*- and *S*-nucleophiles that forms regiodefined perfluoroalkylated naphtho[1,2-b]furan/benzofuran derivatives by harnessing Co-promoted distinctive quadruple C(sp^3^)-F bonds cleavage relay. This chemistry involving controlled and successive selective defluorination at heteronuclear centers would greatly contribute to the preparation of drug-like heterocycles as well as the late-stage elaboration of biorelevant compounds. Controlled experiments and DFT theoretical studies revealed that the combination of cheap cobalt salt with Cs_2_CO_3_ enable expeditious C-F functionalization.

## Highlights

• First example of consecutive selective quadruple C(sp^3^)-F functionalization
• Selectively and controllably partial defluorination at heteronuclear sites
• Conversion to value-added chemicals by using polyfluorocarbons and nucleophiles
• DFT calculations were performed to support mechanistic proposal

## Graphical Abstract

**Figure:**
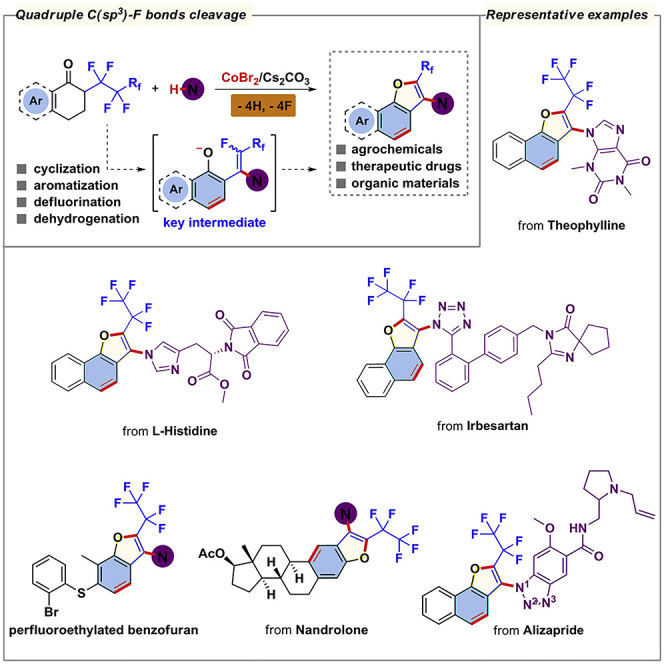

## Introduction

The past decades have witnessed a boom in organofluorine chemistry mainly owing to the unique physical and chemical benefits conferred by the incorporation of fluorine atom or fluorine-containing fragments into organic molecules, which have gained widespread recognition throughout drug discovery, crop protection, polymer chemistry, and materials science (Shao et al., 2015; Feng et al., 2018; Ni and Hu, 2016; Chu and Qing, 2014; Jeschke, 2004; Wang et al., 2014; Cardoso et al., 2018; Ragni et al., 2018). However, it is still challenging and highly desirable to develop reliable tools for performing controlled and selective cleavage of C-F bonds because of the notorious inertness of fluorinated entities arising from their thermodynamic stability and kinetic issues (Wang et al., 2014; Cardoso et al., 2018; Ragni et al., 2018; O'Hagan, 2008). In this context, not only C-F bond construction but also C-F bond activation and functionalization have become attractive subjects for realizing efficient preparation of oligofluorinated compounds, especially starting from readily available polyfluorinated bulk chemicals (Eisenstein et al., 2017; Ahrens et al., 2015; Amii and Uneyama, 2009).

**Figure 1:**
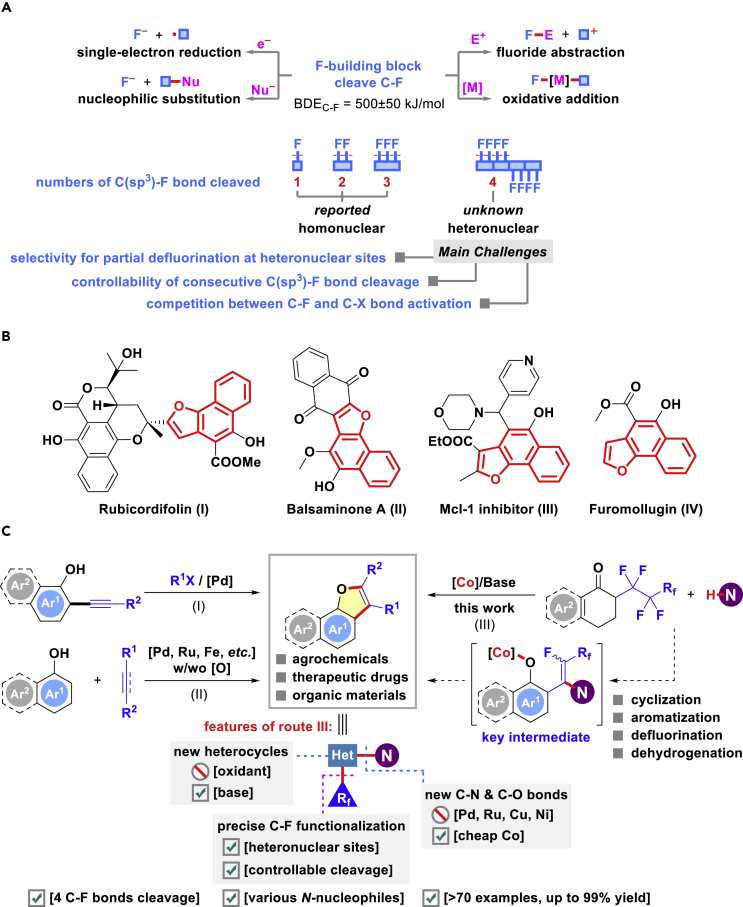
C(sp^3^)-F Bonds Cleavage Strategy (A) Existing approaches through fluoride abstraction, oxidative addition, single-electron reduction, and nucleophilic substitution, all of which are not suitable for the multiple C-F bonds cleavage. (B) Naphtho[1,2-*b*]furan skeleton in naturally occurring and pharmaceutically relevant compounds. (C) Our strategy toward the synthesis of naphtho[1,2-*b*]furan or benzofuran derivatives via highly selective quadruple C(sp^3^)-F functionalization.

Substantial progress has been made in the manipulation of alkenyl (Fujita et al., 2019; Hu et al., 2017, 2018; Lu et al., 2017; Thornbury and Toste, 2016; Tian et al., 2015) and aryl C(sp^2^)-F bonds (Shao et al., 2017; Honeycutt and Hoov, 2018; Priya and Weaver, 2018; Tian et al., 2018) by means of transition-metal catalysis, photocatalysis, and electrochemical techniques (through oxidative addition, single-electron reduction, fluoride abstraction, elimination, nucleophilic substitution, etc.). Although there are many reactions involving aliphatic C-F bond activation by electrophilic compounds (Si-, B-, and P-based cations), Lewis acids, and transition metal species (Jaroschik, 2018; Shen et al., 2015; Stahl et al., 2013), selective transformations of multiple unactivated aliphatic C(sp^3^)-F bonds attached remote to π-system (such as benzylic and allylic moieties) is still scarce (Wang et al., 2018a, 2018b; Kojima et al., 2018; Huang and Hayashi, 2016; Choi et al., 2011; Wang and Jui, 2018; Chen et al., 2017; Romanov-Michailidis et al., 2018; Xu et al., 2015; Wang et al., 2019; Giffin et al., 2013; Pigeon et al., 2010; Gu et al., 2009; Blessley et al., 2012; Hazari et al., 2009; Xue et al., 2015) (Figure 1A). A remarkable particularity of multifluorocarbons is that their reactivity decreases along with an increase in the number of geminal fluorine atoms; this situation increases their difficulties for partial or complete defluorination through discriminating even slightly different reactivity among several C-F bonds (Luo, 2007). On the other hand, although palladium- (Thornbury and Toste, 2016; Xu et al., 2015; Pigeon et al., 2010; Blessley et al., 2012; Hazari et al., 2009), ruthenium- (Tian et al., 2015, 2018; Wang et al., 2018a, 2018b; Huang and Hayashi, 2016), copper- (Hu et al., 2017, 2018; Wang et al., 2018a, 2018b; Kojima et al., 2018), nickel- (Lu et al., 2017; Honeycutt and Hoov, 2018; Tian et al., 2018; Giffin et al., 2013), and iridium-catalyzed (Priya and Weaver, 2018; Choi et al., 2011; Chen et al., 2017; Romanov-Michailidis et al., 2018) C-F functionalization have recently stimulated intense research efforts, the use of earth-abundant and cheap cobalt salts as feasible promoters for such transformations remained less explored (Kuehnel et al., 2013; Ehm et al., 2016; Andrella et al., 2019; Jaeger et al., 2018a, 2018b; Krüger et al., 2016; Dugan et al., 2011, 2012; Li et al., 2013). Moreover, multiple defluorination of polyfluorinated molecules will also be elusive presumably due to the problems posed by the poor controllability and chemical selectivity and high bond dissociation energy. Despite the degradation of perfluoroalkyl substance (Renner, 2001), successive C(sp^3^)-F bond cleavage at heteronuclear sites thus far has not been successfully realized in organic synthesis (Guan et al., 1997) (Figure 1A). As a result, the exploration and discovery of multiple defluorinative reaction mode at different centers may open a new door to allow for the facile synthesis of fine chemicals with a site-selective fluoride-pattern retention and simultaneously avoid the interference from applying exogenously sensitive fluorinating sources.

Herein, we report a novel and efficient strategy involving the successive dehydrogenation and defluorination of α-polyfluoroalkyl ketones with various *N*-nucleophiles and *S*-nucleophiles for accessing modular fluoroalkylated furan derivative, which is a structural unit ubiquitously present in naturally occurring products and biologically active molecules (Kwiecien et al., 2012; Prchalova et al., 2014) (Figure 1B). Compared with typical methods relying on annulation with pre-synthesized heterocycle precursors (Figures 1C-I and 1C-II), such as naphthols and phenols (Heravi and Zadsirjan, 2015), this newly developed procedure features the following salient aspects (Figure 1C-III): (1) realizes a rationally designed aromatization-cyclization cascade along with three C(sp^3^)-H and four C(sp^3^)-F bonds cleavage under simple reaction conditions; (2) cleaves similar C(sp^3^)-F bonds at two different positions for the first time for heterocycle synthesis; (3) succeeds in the assembly of valuable nitrogen-containing pharmacophores with regiodefined fluoroalkyl retention (Lishchynskyi and Grushin, 2013; Xie et al., 2018; Huang et al., 2019); (4) distinguishes itself by efficient C-N or C-S/C-O couplings and concomitant formation of two five-/six-membered (hetero)aromatic rings in a one-pot operation without employing stoichiometric oxidants; (5) enables expeditious late-stage modification of biologically relevant compounds with structural complexity (Cernak et al., 2016).

## Results and Discussion

### Optimization of Reaction Conditions

Initially, we commenced our investigation by using 2-(perfluorobutyl)-3,4-dihydronaphthalen-1(2*H*)-one (**1a**) as a substrate, along with 2-methyl-1*H*-benzo[*d*]imidazole (**2a**) as a nitrogen nucleophile, in the presence of 10 mol% of CoBr_2_, 1.0 equiv of ^*n*^Bu_4_NBr (TBAB), and 2.5 equiv of Cs_2_CO_3_ in DMSO at 70°C under N_2_ for 10 h (Table 1; also see Tables S1–S5 in Supplemental Information for details). To our delight, the proposed tandem strategy could be successfully realized to afford C–N bond-forming (Bariwal and Van der Eycken, 2013) product **3a** in 74% NMR yield (70% isolated yield; Table 1, entry 1). Notably, the excellent performance of the reaction required the simultaneous use of cobalt salt, additive TBAB, and base (Mao et al., 2019; Xue et al., 2015) (Table 1, entries 2–4), as the reaction proceeded with reduced efficiency in the absence of any of them. It is noteworthy that decreasing reaction temperature even to room temperature still gave the same good yield of the product **3a** (Table 1, entry 5). In addition, in a striking comparison with other solvents, including MeCN and DMF (Table 1, entries 6–7), DMSO was found to be the best reaction medium for the transformation (Table 1, entry 5). Furthermore, careful screening of other bases and cobalt sources indicated that Cs_2_CO_3_ and CoBr_2_ were still the base and catalyst of choices in the present reaction (Table 1, entries 8–12).

**Table 1:** Four C(sp^3^)-F Bonds Functionalization: Optimization of Reaction Conditions


Entry	Catalyst	Additive	Base	Solvent	Yield (%)^a^^,^^b^
1	CoBr_2_	TBAB	Cs_2_CO_3_	DMSO	74 (70)^c^
2	–	TBAB	Cs_2_CO_3_	DMSO	39
3	CoBr_2_	–	Cs_2_CO_3_	DMSO	58
4	CoBr_2_	TBAB	–	DMSO	trace
5	CoBr_2_	TBAB	Cs_2_CO_3_	DMSO	74 (71)^c^^,^^d^
6	CoBr_2_	TBAB	Cs_2_CO_3_	MeCN	55^d^
7	CoBr_2_	TBAB	Cs_2_CO_3_	DMF	61^d^
8	CoBr_2_	TBAB	K_2_CO_3_	DMSO	67^d^
9	CoBr_2_	TBAB	Li_2_CO_3_	DMSO	0^d^
10	CoBr_2_	TBAB	DABCO	DMSO	<10^d^
11	Co(OAc)_2_	TBAB	Cs_2_CO_3_	DMSO	49^d^
12	Co(C_2_O_4_)_2_⋅2H_2_O	TBAB	Cs_2_CO_3_	DMSO	0^d^

a Reaction conditions: 2-(perfluorobutyl)-3,4-dihydronaphthalen-1(2*H*)-one (**1a**, 0.30 mmol), 2-methyl-1*H*-benzo[*d*]imidazole (**2a**, 0.60 mmol), catalyst (0.03 mmol), additive (0.3 mmol), and base (0.75 mmol) in solvent (2.0 mL) at 70°C for 10 h under N_2_; TBAB = tetrabutylammonium bromide.

b Yields were determined by NMR analysis with 1,4-dimethoxybenzene as an internal standard.

c Isolated yield.

d At room temperature.

### Substrate Scope Study

**Scheme 1:**
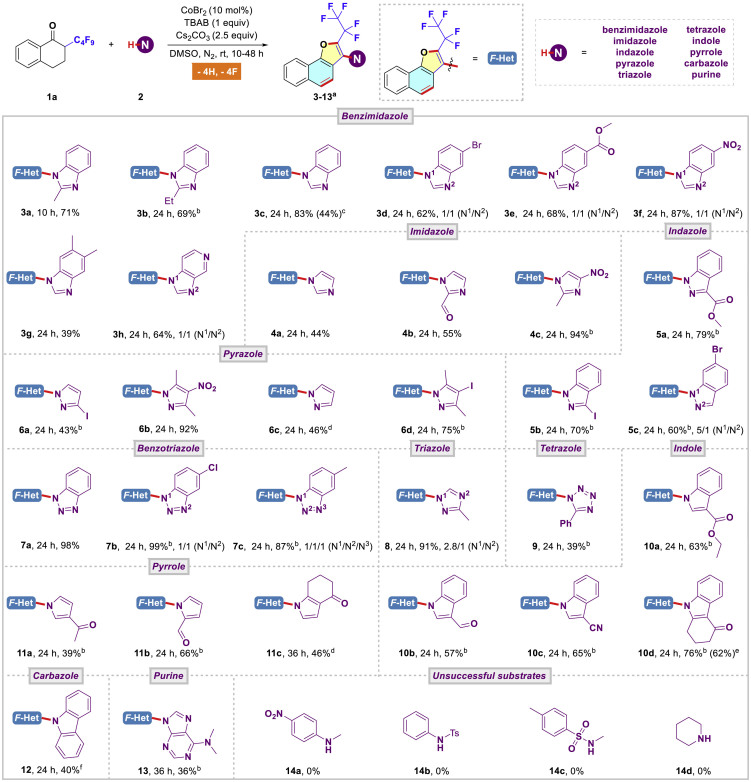
Four C(sp^3^)-F Bonds Functionalization: Substrate Scope of Various Nitrogen Nucleophiles ^a^Standard reaction conditions (0.3 mmol scale); isolated yields. ^b^At 70°C. ^c^10 h. ^d^At 100°C. ^e^3 mmol scale reaction for 48 h. ^f^At 120°C.

**Figure 2:**
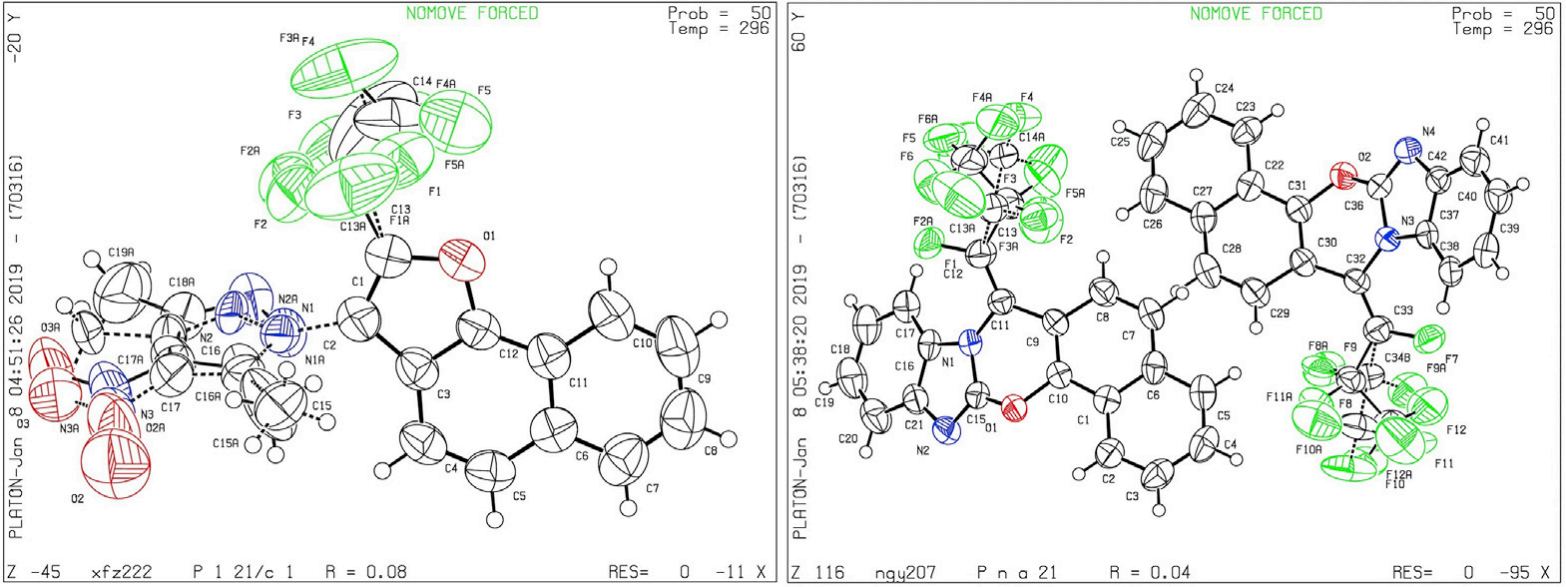
The X-ray Crystal Structures of Products 6b (CCDC, 1881997, left) and 19 (CCDC, 1881996, right)

With the optimized reaction conditions in hand, the general applicability of this predictable and mild cascade reaction was tested with a wide range of electronically disparate nitrogen nucleophiles. As shown in Scheme 1, almost every kind of privileged *N*-heterocycle components in medicinal chemistry (Taylor et al., 2014), including, but not limited to, benzimidazoles (**3a**-**3h**, 39%–87%), imidazoles (**4a**-**4c**, 44%–94%), indazoles (**5a**-**5c**, 60%–79%), pyrazoles (**6a**-**6d**, 43%–92%), triazoles (**7**-**8**, 87%–99%), tetrazole (**9**, 39%), indoles (**10a**-**10d**, 57%–76%), pyrroles (**11a**-**11c**, 39%–66%), carbazole (**12**, 40%), and purine (**13**, 36%), could directly couple with substrate **1a** to produce pentafluoroethylated naphtho[1,2-*b*]furan in moderate to good yields. Particularly, besides the ready introduction of polyfluoroalkyl group and *N*-heterocycle in naphtho[1,2-*b*]furan skeleton, the present protocol also serves as an efficient method for the direct construction of naphtho[1,2-*b*]furan scaffold via defluorination and cyclization cascade, which also have its merit when compared with previous methods where preprepared or commercial naphtho[1,2-*b*]furan was directly employed for further functionalization (Heravi and Zadsirjan, 2015). Importantly, some synthetically valuable functional groups, such as halogen (Cl, Br, I), ester, nitro, formyl, keto, as well as cyano group, were amenable to the present catalytic system, which offered the synthetic potential for further elaboration. Notably, as for nucleophiles such as indazole, triazole, indole, pyrrole, and carbazole, the isolated yields of the corresponding products could be remarkably improved by means of increasing reaction temperature and prolonging reaction time. In addition, considering the procedural simplicity and synthetically easy accessibility, a concise procedure was successfully achieved for the large-scale construction of product **10d** under slightly modified reaction conditions (62%, 0.88 g). These results clearly demonstrated the high efficiency and unique advantages of this amination protocol. However, our attempts to expand the substrate scope to anilines (**14a**-**14b**), sulfonamide (**14c**), and cyclic amine (**14d**) have been proven fruitless. Moreover, the structure of product **6b** was unambiguously confirmed by single crystal X-ray diffraction analysis (CCDC 1881997; Figure 2; also see Supplemental Information for details).

**Scheme 2:**
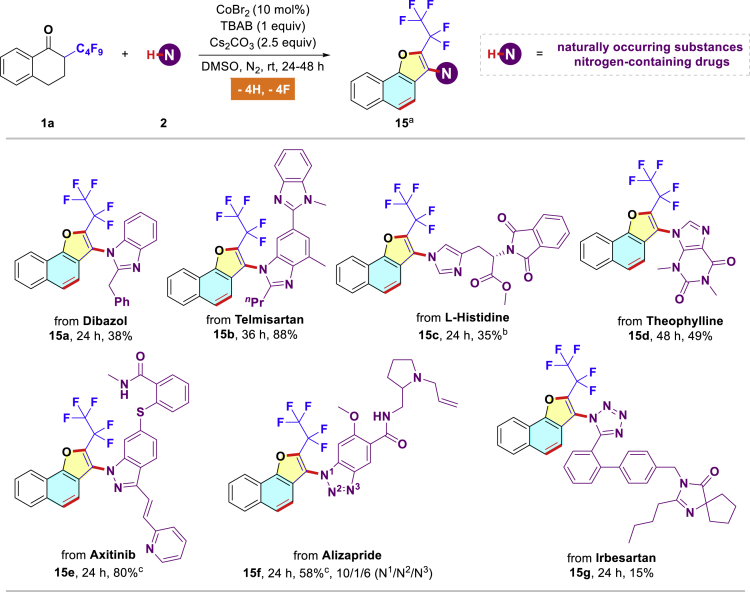
Four C(sp^3^)-F Bonds Functionalization: Application in the Synthesis of Complex Molecules to Access Druglike Scaffolds ^a^Standard reaction conditions (0.3 mmol scale); isolated yields. ^b^3 equiv of **2** was used. ^c^0.1 mmol scale.

In view of the importance of late-stage modification in drug discovery, this practical approach has been specifically evaluated with respect to representative nitrogen-containing complex molecules (Scheme 2). For example, the hypertension therapeutic dibazol could be smoothly incorporated into the naphtho[1,2-*b*]furan derivative **15a** in 38% yield. A pharmaceutical unit of telmisartan readily underwent this dehydrogenative and defluorinative reactions, leading to the desirable heterocycle **15b** in a good yield (88%). Naturally occurring substances such as L-histidine and theophylline also reacted chemoselectively with α-perfluoroalkyl ketone **1a** to furnish the corresponding products **15c** and **15d** in 35% and 49% yields, respectively. Furthermore, by using three known nitrogen-containing drugs (axitinib, alizapride, irbesartan) as viable coupling partners in the present protocol, we were also able to achieve the functionalization of these pharmaceuticals (**15e**-**15g**). Interestingly, the problems arising from the competitive couplings with nucleophilic amide moieties were well avoided in these cases (**15e**-**15f**).

**Scheme 3:**
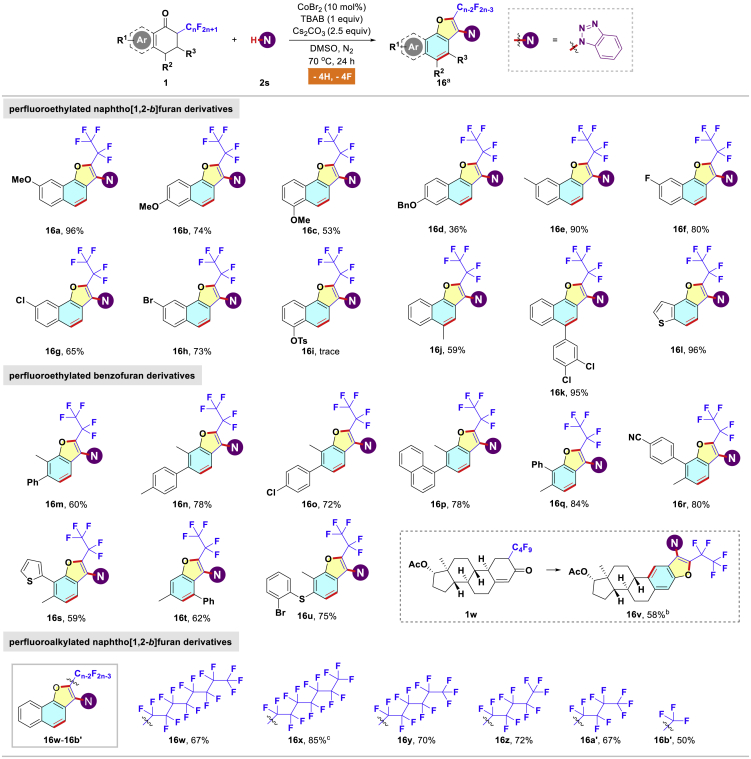
Four C(sp^3^)-F Bonds Functionalization: Substrate Scope of Various Perfluoroalkyl Ketones ^a^Standard reaction conditions (0.2 mmol scale); isolated yields. ^b^0.05 mmol scale. ^c^0.3 mmol scale.

Driven by the success of above reactions, subsequently we attempted to expand the substrate scope of four C(sp^3^)-F bonds cleavage to encompass various α-polyfluoroalkyl ketones as starting materials (Scheme 3). A variety of ketones bearing either electron-donating groups (including MeO, OBn, Me) or electron-withdrawing groups (such as F, Cl, Br) on the phenyl rings were tolerated, affording the polycyclic products with acceptable yields (**16a**-**16h**, 36%–96% yields). However, ketone **1j** was proven to be an inappropriate candidate for the present reaction (**16i**), and it remained intact in the reaction. In addition, substrate **1** possessing an alkyl or aryl substituent at the C4-position underwent the aromatization-cyclization cascade with high efficiency (**16j**-**16k**, 59%–95% yields). In a similar manner, the reaction worked equally well with heteroaryl ketone to provide the anticipated heterocyclic variant **16l** in 96% yield. Apart from perfluorobutyl 3,4-dihydronaphthalen-1(2*H*)-one (**1a**), we were pleased to observe that the generality of this transformation could be further broadened by employing diverse perfluorobutyl cyclohex-2-en-1-one derivatives, which produced perfluoroethyl benzofuran derivatives **16m**-**16v** in 58%–84% yields. The substrate **1w** derived from Nandrolone could also participate into the coupling with **2s** to produce the product **16v** in 58% yield. Also, our method was able to address the paucity of process for site-selective fluoroalkylation. Interestingly, the perfluoroalkyl chain length ranging from 10 to 3 carbons only has a slight impact on the reaction outcomes (**16w**-**16b′**, 50%–85% yields). Furthermore, our protocol allowed convenient access to trifluoromethyl-substituted drug analogue **16b'** (Taylor et al., 2014). In view that π-conjugated benzofuran derivatives are pivotal structural constituents of optoelectronic materials and pharmaceutical molecules, the present distinctive methodology will provide chemists an attractive alternative for manufacturing these fluorinated polyfused skeletons (Tsuji and Nakamura, 2017).

**Scheme 4:**
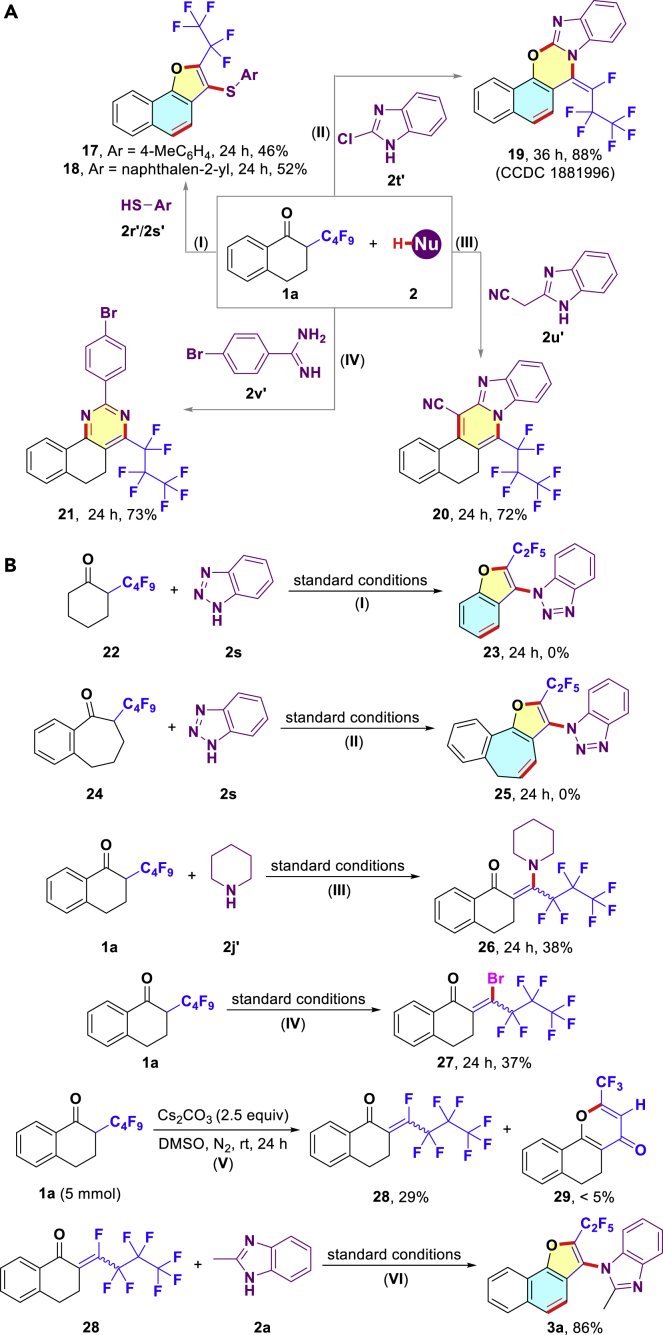
Four C(sp^3^)-F Bonds Functionalization with Other Mono- or Dinucleophiles and Control Experiments (A) Four C(sp^3^)-F bonds cleavage with aryl mercaptan or 1,3-dinucleophiles. (B) Some control experiments performed for gaining more mechanistic insight.

The development of original and novel scaffolds is a persistent quest in medicinal chemistry. Finally, the reactions employing aryl mercaptan (**2r′-2s′**) forged the desired sulfoethers **17** and **18** containing heterocycles (Scheme 4A–I). Moreover, the use of benzoimidazole **2t′**, which possessed a halogen at the C2 position, produced an unexpected pentacyclic fused compound **19** through the removal of three fluorides [Scheme 4A-II; also see the X-ray crystal structure of compound **19** (CCDC 1881996; Figure 2) in Supplemental Information for details]. However, 1,3-bis-nucleophiles possessing a carbon atom as the tether could not participate in the designed aromatization-annulation and predominantly afforded condensed dihydrobenzoquinazoline **20** and dihydrobenzoquinoline **21** in 72% and 73% yields, respectively (Schemes 4A-III and 1A-IV). These results would significantly contribute to the mechanistic understanding of the reaction pathway.

### Mechanism Study

Further insights were obtained for elementary information of the reaction mechanism via the control experiments outlined in Scheme 4B. First, no anticipated product **23** was isolated when 2-(perfluorobutyl)cyclohexan-1-one (**22**) was employed as a prefluorinated building block, revealing that the phenyl group or unsaturated C=C moiety in the α-polyfluoroalkyl ketone was essential for the established cascade defluorination (Scheme 4B-I). Next, the experiment employing seven-membered ring **24** under the standard conditions has been proven futile, indicating that the autoaromatization of the dihydronaphthalenone might become an important driving force for the successive C(sp^3^)-F bonds cleavage (Scheme 4B-II). Then, the significance of the nucleophiles was demonstrated through the fact that piperidine exclusively coupled with **1a**, leading to β-aminated ketone **26** in 38% yield (Scheme 4B-III; also see Scheme 1, **14d**). Moreover, it was found that α-defluorination occurred to form intermediate **27** (with TBAB, via 1,4-conjugate addition of Br anion) and **28** (without TBAB) in the absence of an *N*-nucleophile (Schemes 4B-IV and 4B-V). We believed that TBAB additive might accelerate the sequential events of C-N/C-O couplings, aromatization, and defluorination. On the other hand, the poor solubility of Cs_2_CO_3_ might lead to inconsistencies in mixing the reagents and thus poor reproducibility, and this issue could be alleviated by the addition of TBAB (Sasson and Neumann, 1997). Simultaneously, less than 5% yield of byproduct **29** was obtained under basic conditions (Scheme 4B-V). As expected, key intermediate **28** could be converted to the corresponding product **3a** in 86% yield under the standard conditions (Scheme 4B-VI).

### Density Functional Theory Calculations

**Figure 3:**
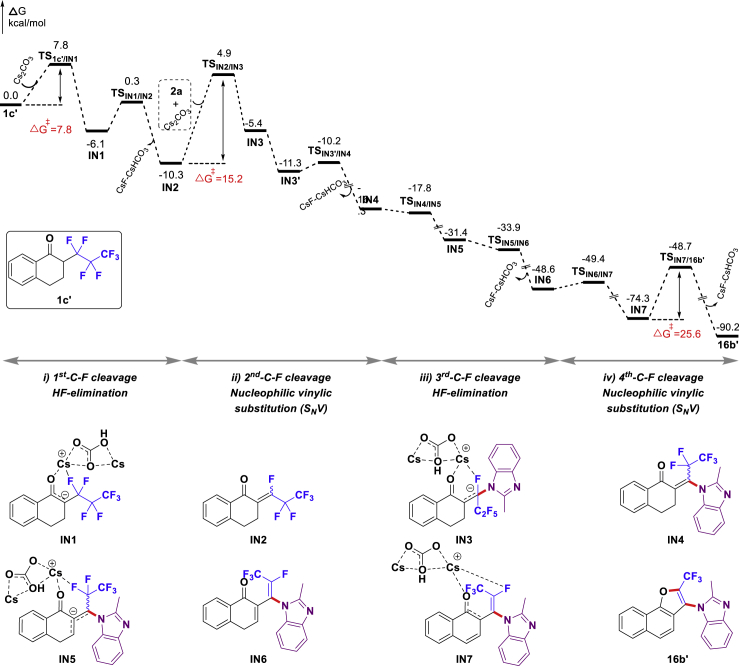
Free Energy Profile of the Cs_2_CO_3_-Mediated Four C(sp^3^)-F Bonds Cleavage and C-N/O Coupling Cascade Reaction (in kcal/mol)

Density functional theory (DFT) calculations with the B3LYP functional (Becke, 1993; 1988; Lee et al., 1988), including Grimme's D3 dispersion correction (DFT-D3) (Grimme et al., 2010; Goerigk and Grimme, 2011), were carried out to explore the role of Cs_2_CO_3_ and CoBr_2_ on this quadruple C(sp^3^)-F bond functionalization (Data S1) [all calculations were performed with the Gaussian 09 package (Frisch et al., 2013), and optimized structures were visualized using CYLview (Legault, 2009); see Supplemental Informationfor computational details]. It should be noted that the desired product **3a** could be obtained in 39% yield in the absence of CoBr_2_ (Table 1, entry 2). Therefore, we first investigated the possible pathway of the Cs_2_CO_3_–mediated defluorinative C-N/C-O coupling reaction by using 2-(perfluoropropyl)-3,4-dihydronaphthalen-1(2*H*)-one (**1c′**) and 2-methyl-1*H*-benzo[*d*]imidazole (**2a**) as model substrates. As indicated earlier, the perfluoroalkyl chain length only has a slight impact on the reaction outcomes. Therefore, we chose simpler **1c′** rather than **1a** as a model substrate to simplify the theoretical calculation. The calculated free energy profile and optimized transition state structures are listed in Figure 3.

**Figure 4:**
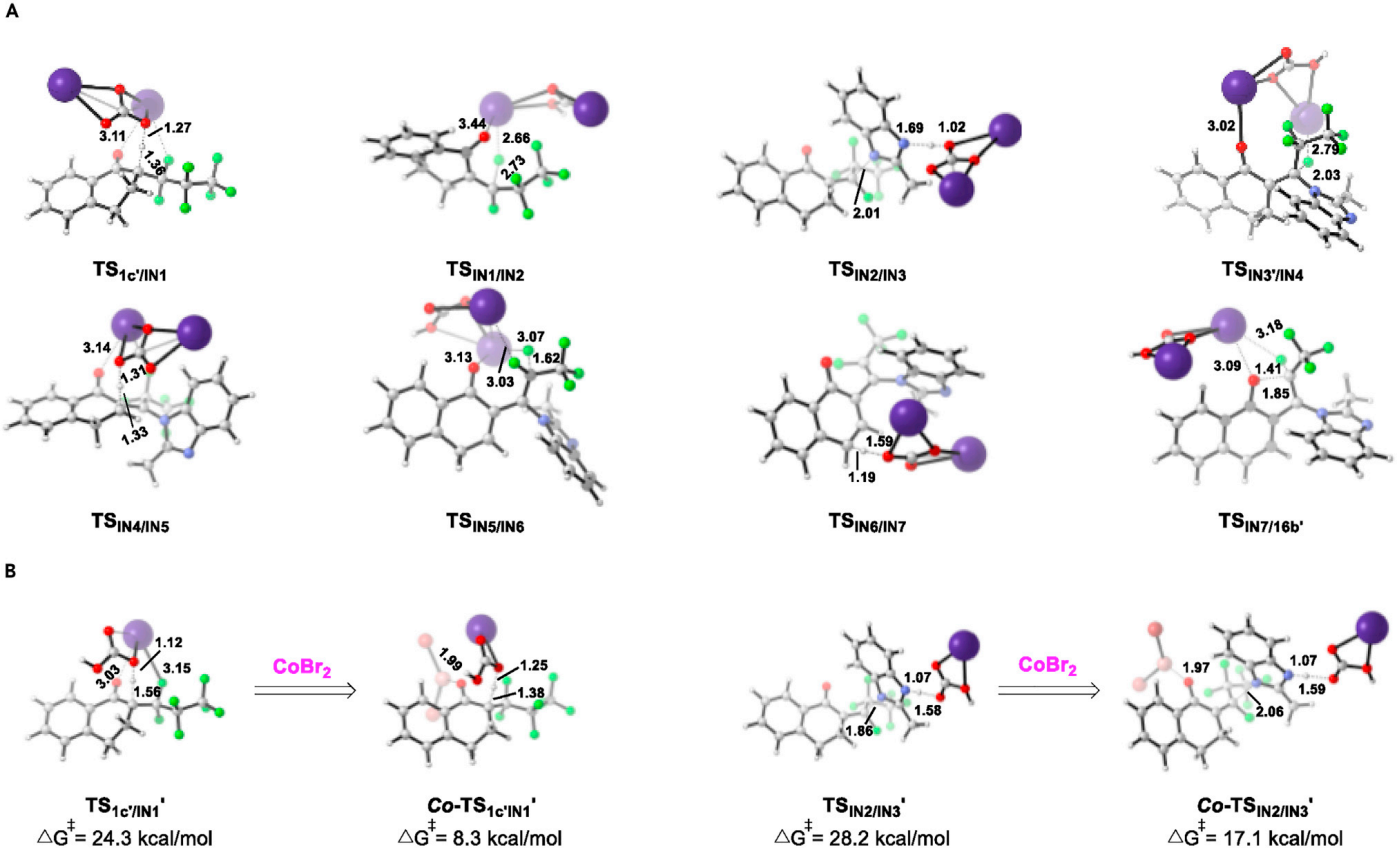
Optimized Transition State Structures (A) Optimized transition state structures (distances are in Å). (B) Transition state structures of CsHCO_3_-mediated deprotonation of **1c′** and *N*-nucleophilic addition step of **IN2** without or with CoBr_2_.

As shown in Figures 3 and 4A, the entire pathway consists of the following steps: (1) a Cs_2_CO_3_-assisted elimination of the first HF, proceeding through the deprotonation of the α-hydrogen of carbonyl compound **1c'** (via **TS**_**1c'/IN1**_) and the subsequent elimination of fluorine anion (via **TS**_**IN1/IN2**_) in an **E2** elimination manner, generates an α,β-unsaturated intermediate **IN2**. The corresponding barrier of these two transition states are only 7.8 and 6.4 kcal/mol, respectively. (2) The nucleophilic vinylic substitution (**S**_**N**_**V**) of **IN2**, in which the fluorine atom is replaced by a nitrogen nucleophile (**2a**), produces a C-N coupling intermediate **IN4**. The rate-determining step of this process is the π-perpendicular attack of **2a** toward **IN2** (**TS**_**IN2/IN3**_, with a barrier of 15.2 kcal/mol). Abstraction of F^−^ by the Cs_2_HCO_3_ cation from the tetrahedral intermediate **IN3** forms the enamine intermediate **IN4**, which is exergonic by 16.3 kcal/mol. (3) Further elimination of the third HF proceeding through a similar deprotonation (via **TS**_**IN4/IN5**_) and fluorine anion elimination sequence (via **TS**_**IN5/IN6**_) affords a delocalized naphthalen-1(4*H*)-one intermediate **IN6**. Owing to the π-conjugated effect as well as the existence of Cs^+^ … O and Cs^+^ … F interaction, the third C-F bond cleavage step is ready to occur (highly exergonic by about 48.6 kcal/mol) (Li et al., 2013). (4) Rearomatization of **IN6** via the deprotonation of naphthalen-1(4*H*)-one (**TS**_**IN6/IN7**_) furnishes a zwitterionic complex **IN7**, which could further undergo an intramolecular **S**_**N**_**V**-type cyclization (via **TS**_**IN7/16b'**_) to give the desired product **16b'**. The whole defluorination coupling reaction is totally exergonic by 90.2 kcal/mol (relative to the separated reactants). The intramolecular cyclization reaction of **IN7**, involving a single-step *O*-nucleophilic σ-attack (**TS**_**IN7/16b'**_), is the rate-limiting step of the reaction pathway with a barrier of 25.6 kcal/mol. It is noted that Cs_2_CO_3_ plays a crucial role for facilitating both the HF elimination and the nucleophilic vinylic substitution. These computational results are consistent with experimental observations.

Next, we also performed DFT calculations to reveal the influence of CoBr_2_ additive on this defluorination reactions. Some key steps with relatively high activation barriers discussed earlier (**TS**_**IN2/IN3**_ and **TS**_**IN7/16b'**_) were calculated in the presence of CoBr_2_ (see Figures S235 and S236 in Supplemental Information for details). Our calculations show that CoBr_2_ could lower the activation barrier of *N*-nucleophilic vinylic substitution step from 15.2 kcal/mol to 5.9 kcal/mol (***Co*-TS**_**IN2/IN3**_), which makes the intermolecular C-N bond formation more readily. However, it does not have significant influence on the rate-limiting step (26.7 kcal/mol for ***Co*-TS**_**IN7/16b'**_, and 25.6 kcal/mol for **TS**_**IN7/16b'**_). We hypothesized that the basicity of conjugated acid CsHCO_3_, which is formed with the consumption of strong base Cs_2_CO_3_ during the reaction process, is not sufficient to deprotonate the related substrates or intermediates in the absence of Co(II) salt. Both the barriers of the deprotonation of **1c'** (via **TS**_**1c'/IN1**_**'**) and the nucleophilic addition of nucleophile **2a** toward **IN2** (via **TS**_**IN2/IN3**_**'**) activated by CsHCO_3_ are higher than those activated by Cs_2_CO_3_ (24.3 kcal/mol versus 7.8 kcal/mol and 28.2 kcal/mol versus 15.2 kcal/mol) (Figure 4B). Interestingly, energy barriers of transition states with CoBr_2_, ***Co***-**TS**_**1c'/IN1**_**'** and ***Co*-TS**_**IN2/IN3**_**'**, are calculated to be lower in energies than those with only CsHCO_3_ (8.3 kcal/mol versus 24.3 kcal/mol and 17.1 kcal/mol versus 28.2 kcal/mol). These computational results suggest that CoBr_2_ might act as a Lewis acid (Stahl et al., 2013) to facilitate the base-mediated defluorinative cascade. It was found that other Lewis acid alternatives could also promote the defluorinative cascade under the optimal reaction conditions (see Table S6 in Supplemental Information for details), which is consistent with this computational result.

**Scheme 5:**
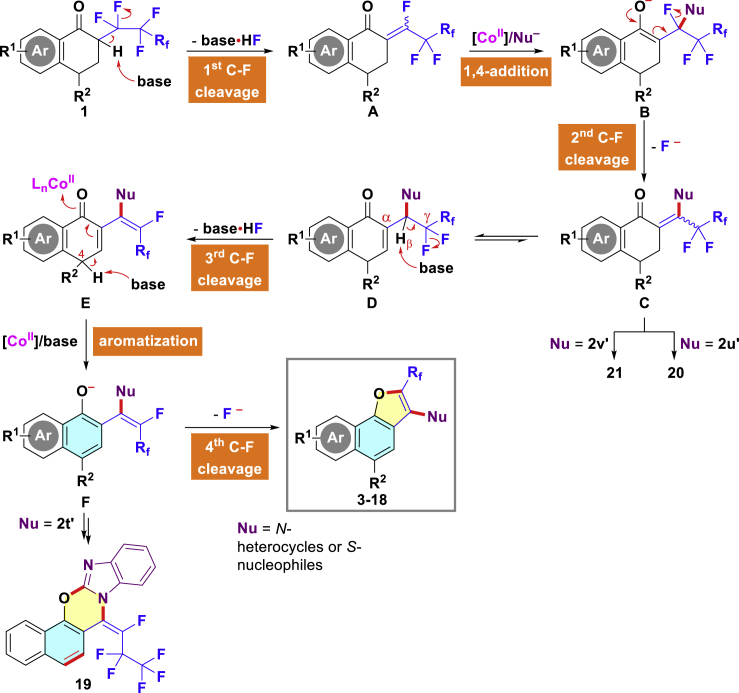
Proposed Mechanism

On the basis of the abovementioned control experiments, DFT calculations, and literature survey (Jaroschik, 2018; Shen et al., 2015; Stahl et al., 2013), a possible mechanism of the Co(II)-assisted/base-promoted defluorination for the formation of the observed products is described in Scheme 5. Initially, a rapid nucleophilic 1,4-addition/fluoride elimination event of the *N-* or *S*-nucleophile with α,β-unsaturated carbonyl compound A, which is *in situ* generated from substrate **1** by spontaneously removing a molecule of HF with the assistance of Cs_2_CO_3_, occurs to give the β-coupled species **C**. Subsequently, tautomerization of nascent **C** affords a more stable endocyclic naphthalen-1(4*H*)-one **D**. Next, β,γ-desaturation readily proceeds in the presence of Cs_2_CO_3_ by extrusion of the third fluoride ion to produce the transient intermediate **E**, mainly owing to the π-conjugated effect. The subsequent formation of a naphthol/phenol anion **F** may be explained by a base-assisted elimination of a proton at C4-position of **E**, and autoaromatization greatly contributes to the driving force of this step (Pigeon et al., 2010). Finally, occurrence of an intramolecular *O*-nucleophilic vinylic substitution (Li et al., 2013) (S_N_V; v*ia* intermediate **F**) delivers the ring-closure products **3**-**18** via readily cleaving the fourth C-F bond. On the other hand, the possibility that the reaction proceeds through 5-*endo*-*trig* cyclization could not be ruled out (Ichikawa et al., 2002). Alternatively, external nucleophilic attack by 2-(1*H*-benzo[*d*]imidazol-2-yl)acetonitrile (**2u′**) or 4-bromobenzimidamide (**2v′**), which bears two reactive sites (Schemes 4-III and 4-IV), would preferentially condense to produce conventional heterocycles **20**-**21** rather than undergoing successive defluorination. Notably, intramolecular nucleophilic annulation of intermediate **F** would also furnish the polycyclic fused product **19**, where the new C-O bond was forged with benzoimidazole keeping γ-C-F bond intact. This result indirectly reflects the formation of intermediate **F** in the reaction. It should be mentioned that the reactions are highly regioselective because a wide variety of perfluoroalkylated naphtho[1,2-*b*]furan derivatives could be exclusively accessed even if there might potentially exist several competitive side reactions.

### Conclusions

In summary, we have developed an appealing cobalt(II)/Cs_2_CO_3_-promoted quadruple defluorinative mode for accessing perfluoroalkylated naphtho[1,2-*b*]furan//benzofuran derivatives by using prefluoroalkylated ketones with various *N*-heterocycles, including benzimidazole, imidazole, indazole, pyrazole, triazole, tetrazole, indole, pyrrole, carbazole, and purine. This method exhibited mild reaction conditions, broad substrate scope, and good functional group compatibility. Extension of the method to other kinds of *S*-nucleophiles also improved the synthetic potentials of the present method in the context of diversity-oriented synthesis. Moreover, the method could also be applied to the late-stage functionalization of some representative nitrogen-containing druglike molecules, which might potentially find applications in medicinal chemistry and pharmaceutical industry. Controlled experiments and DFT theoretical studies revealed that the combination of cheap cobalt salt with Cs_2_CO_3_ enables expeditious C-F cleavage. As such, we anticipate that this strategy will provide a complementary new approach to enable the fluorine-containing modification of complex biological molecules that are not easy to achieve by using current state-of-the-art methods.

### Limitations of the Study

However, the substrate scope of *N*-nucleophile is somewhat limited, as anilines, sulfonamide, and cyclic amine have been proven fruitless in the present reactions.

### Resource Availability

#### Lead Contact

Further information and requests for resources and reagents should be directed to and will be fulfilled by the Lead Contact, Zhi-Liang Shen (ias_zlshen@njtech.edu.cn).

#### Materials Availability

All unique/stable reagents generated in this study are available from the Lead Contact with a completed Materials Transfer Agreement.

#### Data and Code Availability

The crystallography data have been deposited at the Cambridge Crystallographic Data Center (CCDC) under accession number CCDC: 1881996 (**19**) and CCDC: 1881997 (**6b**) and can be obtained free of charge from www.ccdc.cam.ac.uk/getstructures.

Original/source data for Figure 1, Figure 2, Figure 3, Figure 4, Scheme 1, Scheme 2, Scheme 3, Scheme 4, Scheme 5, and Table 1 in the paper are available at https://doi.org/10.1016/j.isci.2020.101259. Cartesian coordinate is provided as a xyz file (Data S1.xyz).

## Methods

All methods can be found in the accompanying Transparent Methods supplemental file.

## Supplemental Information

Document S1. Transparent Methods, Figures S1–S251, and Tables S1–S6

Data S1. Cartesian Coordinates, Related to Figures 3 and 4
