# Supplementary material for: Selective Quadruple C(sp3)-F Functionalization of Polyfluoroalkyl Ketones
Source: iScience. 2020 Jun 11;23(7):101259. doi: 10.1016/j.isci.2020.101259 (PMC7327834; doi:10.1016/j.isci.2020.101259)
Supplement: Document S1. Transparent Methods, Figures S1–S251, and Tables S1–S6 [file mmc1.pdf]

iScience, Volume *23*

Supplemental Information

Selective Quadruple C(sp^3)-F Functionalization of Polyfluoroalkyl Ketones

Ting Xie, Guo-Qiang Wang, Ya-Wen Wang, Weidong Rao, Haiyan Xu, Shuhua Li, Zhi-Liang Shen, and Xue-Qiang Chu

Supplemental Figures for NMR spectrums:

Figure S1. ^{1}H NMR spectrum of **3a**, related to **Scheme 1**.

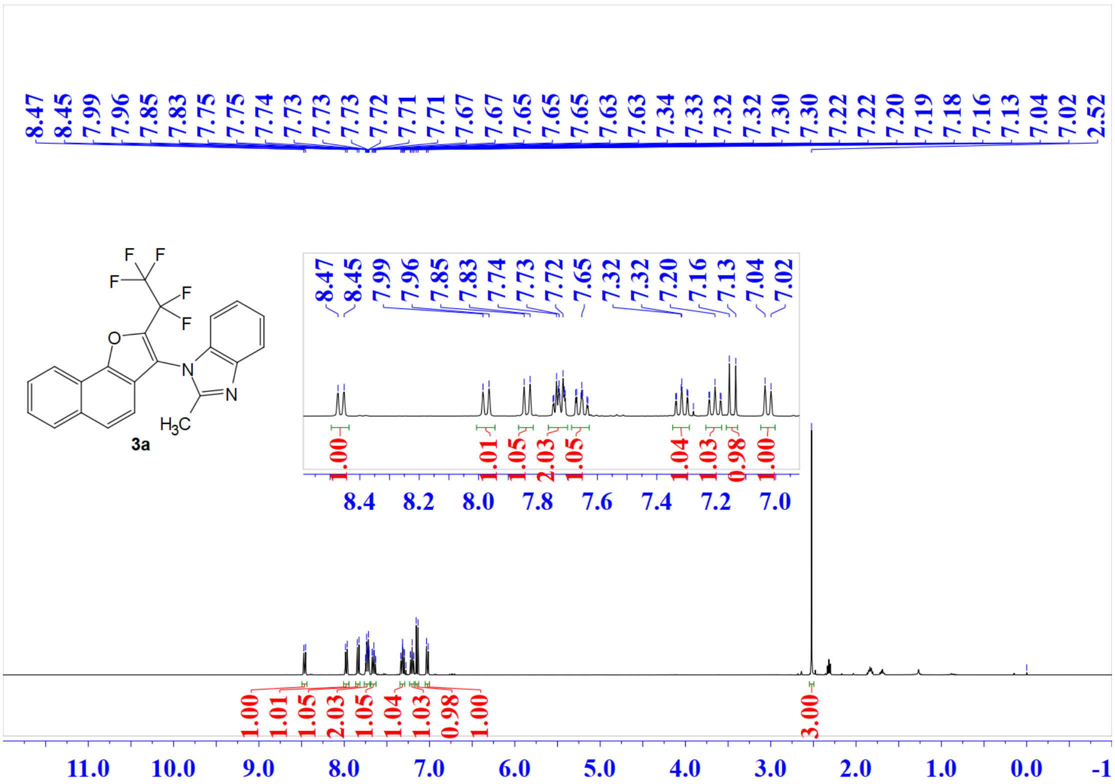

Figure S2. ^{19}F NMR spectrum of **3a**, related to **Scheme 1**.

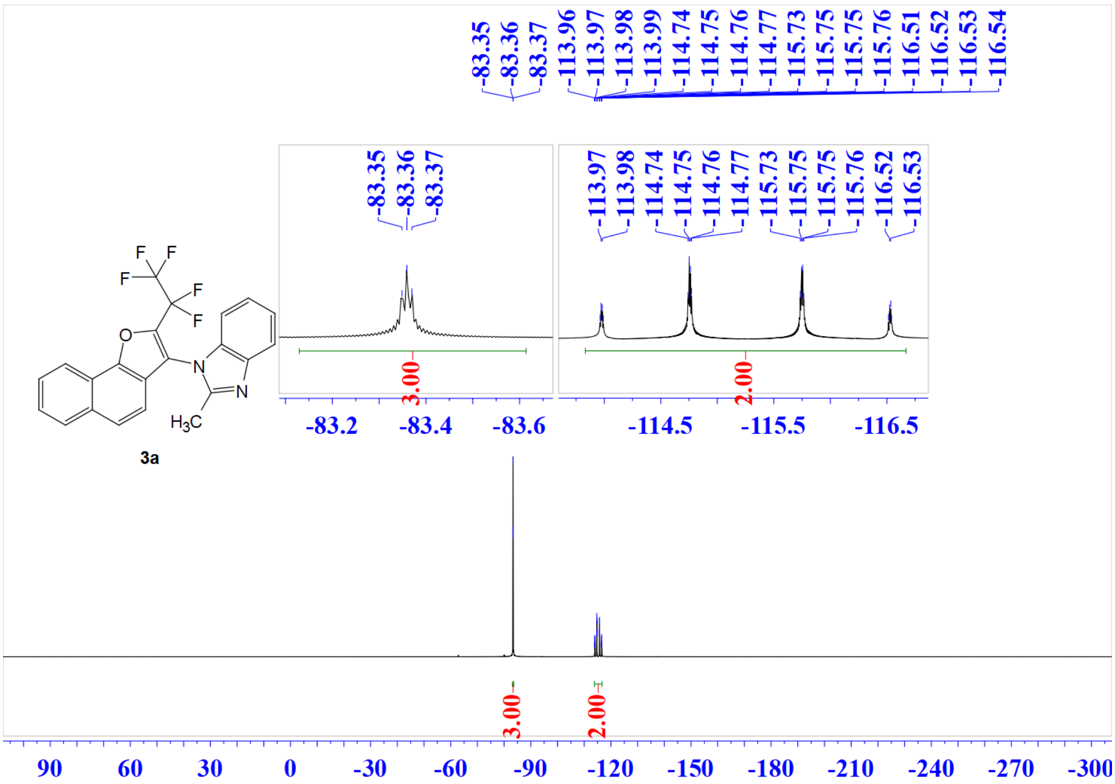

Figure S3. ^{13}C NMR spectrum of **3a**, related to **Scheme 1**.

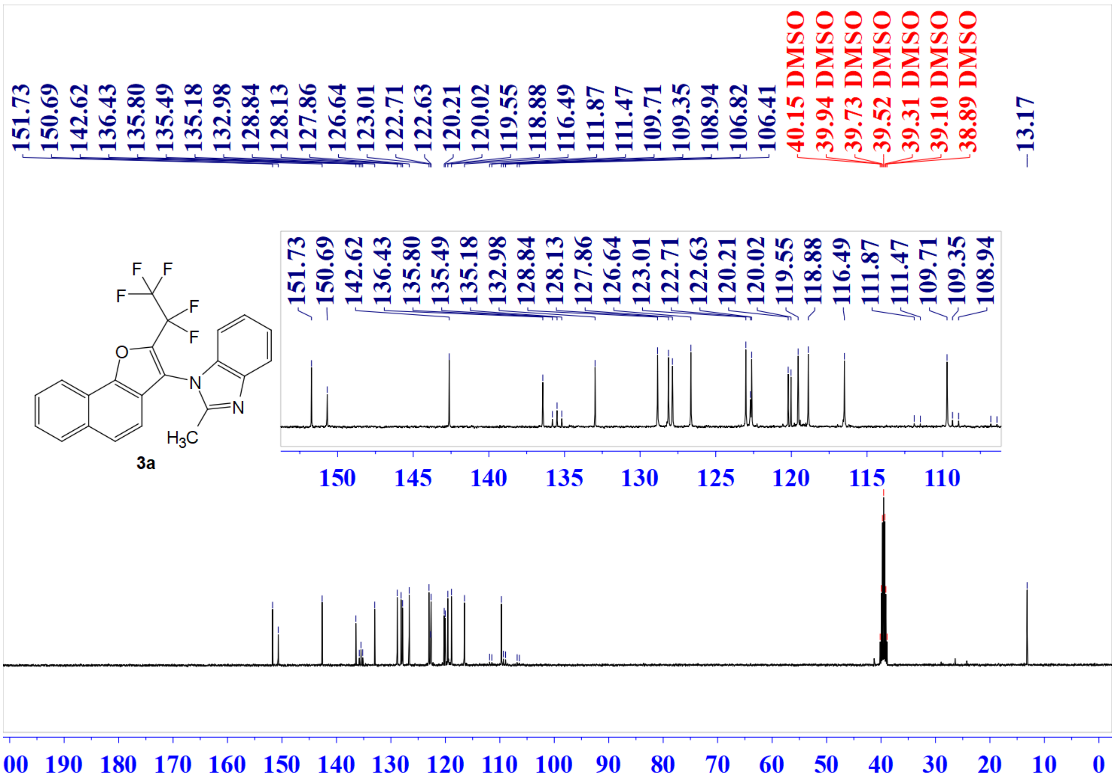

Figure S4. ^{1}H NMR spectrum of **3b**, related to **Scheme 1**.

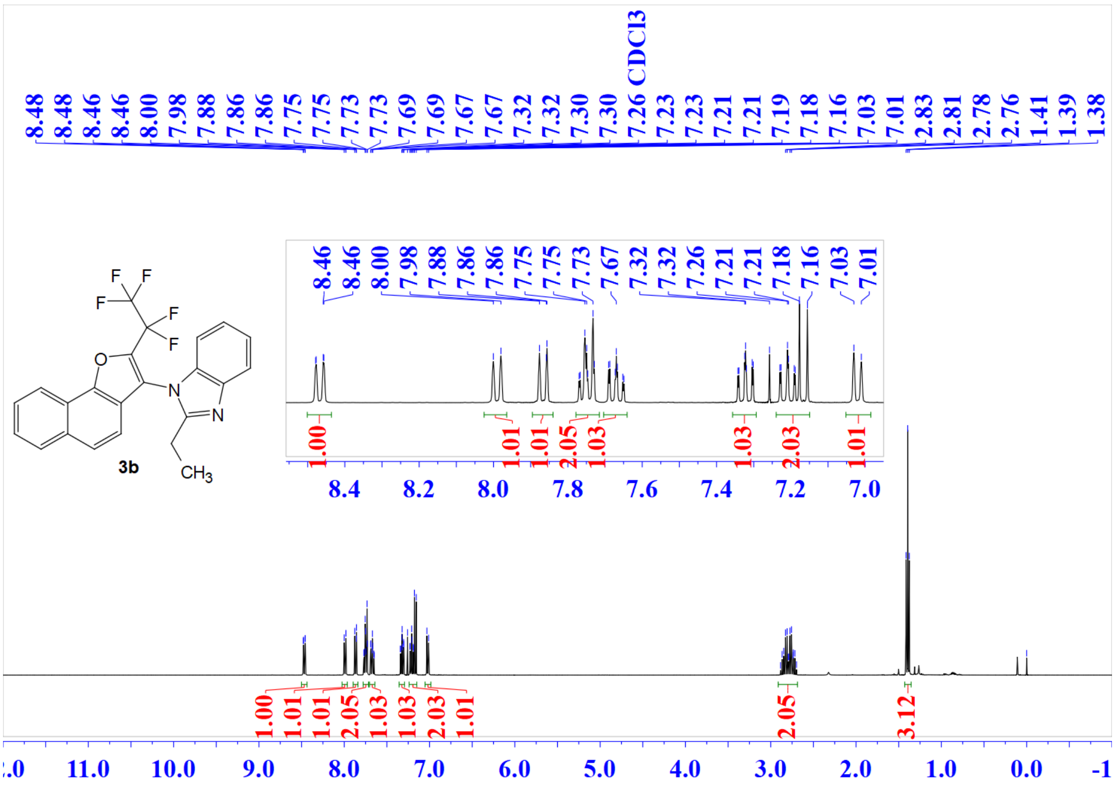

Figure S5. ^{19}F NMR spectrum of **3b**, related to **Scheme 1**.

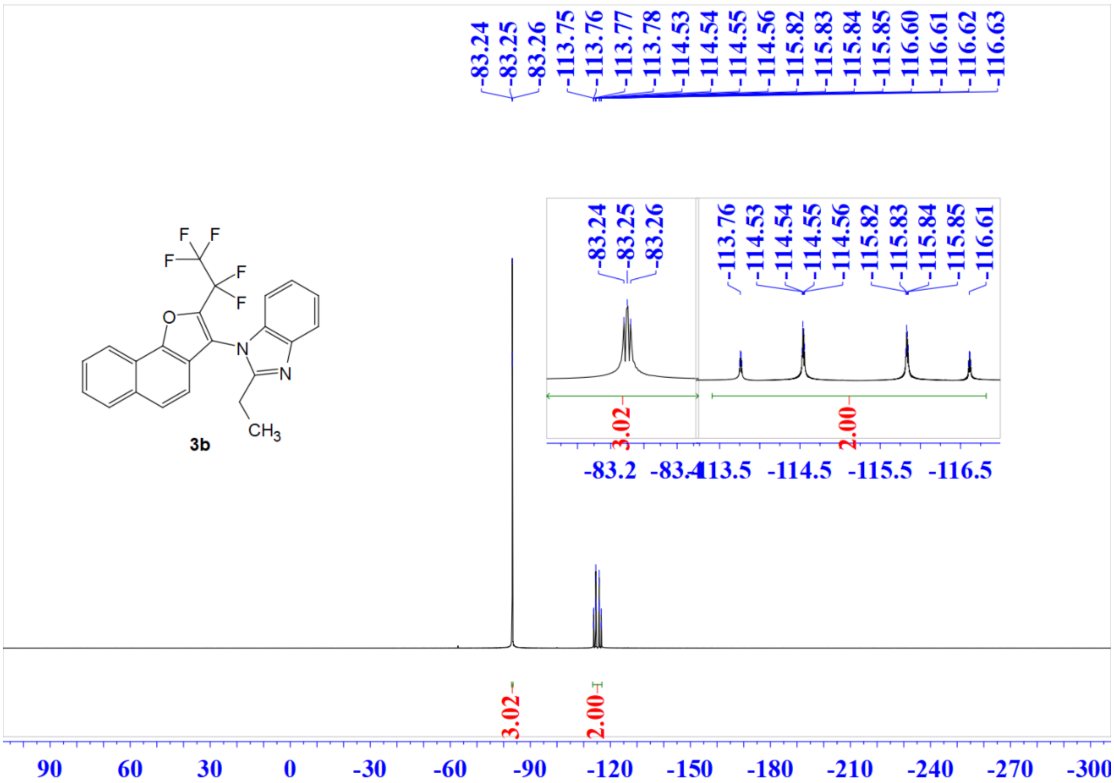

Figure S6. ^{13}C NMR spectrum of **3b**, related to **Scheme 1**.

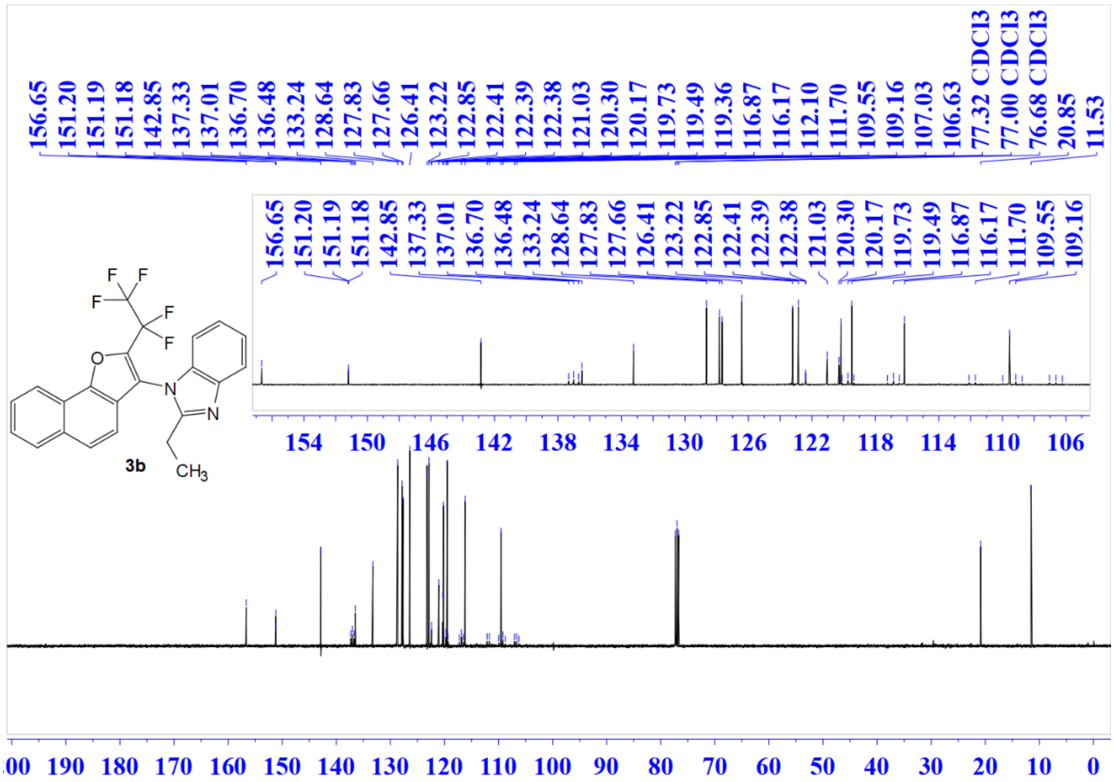

Figure S7. ^{1}H NMR spectrum of **3c**, related to **Scheme 1**.

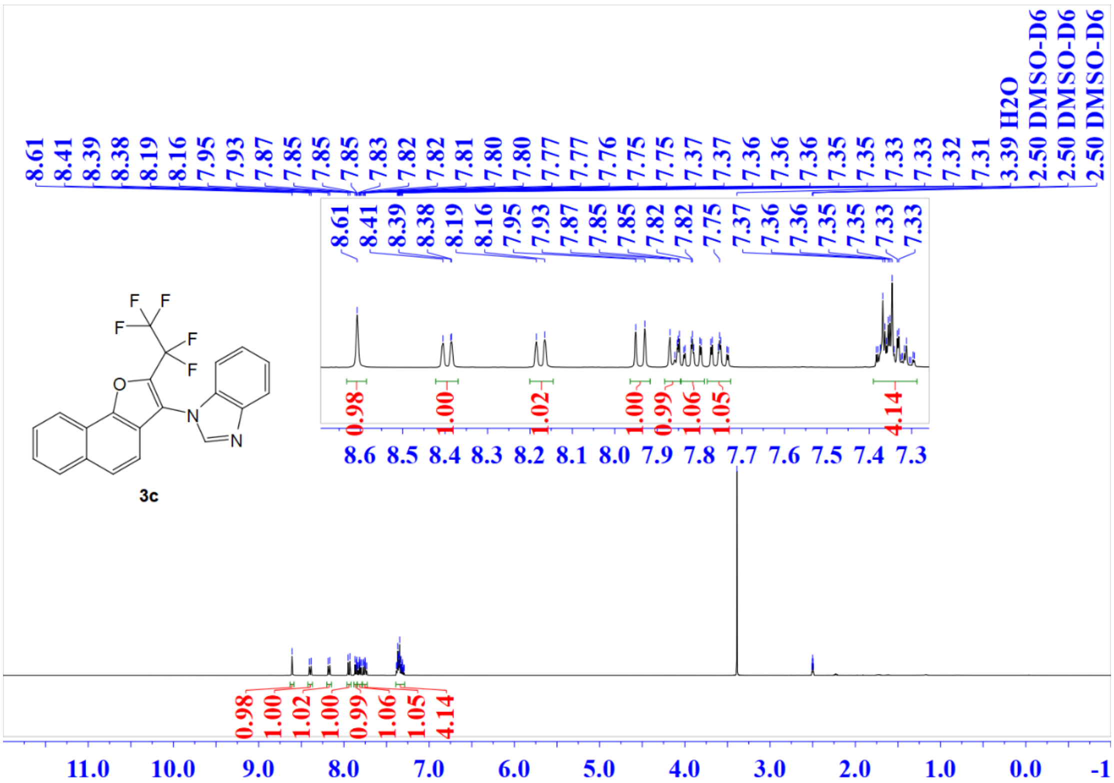

Figure S8. ^{19}F NMR spectrum of **3c**, related to **Scheme 1**.

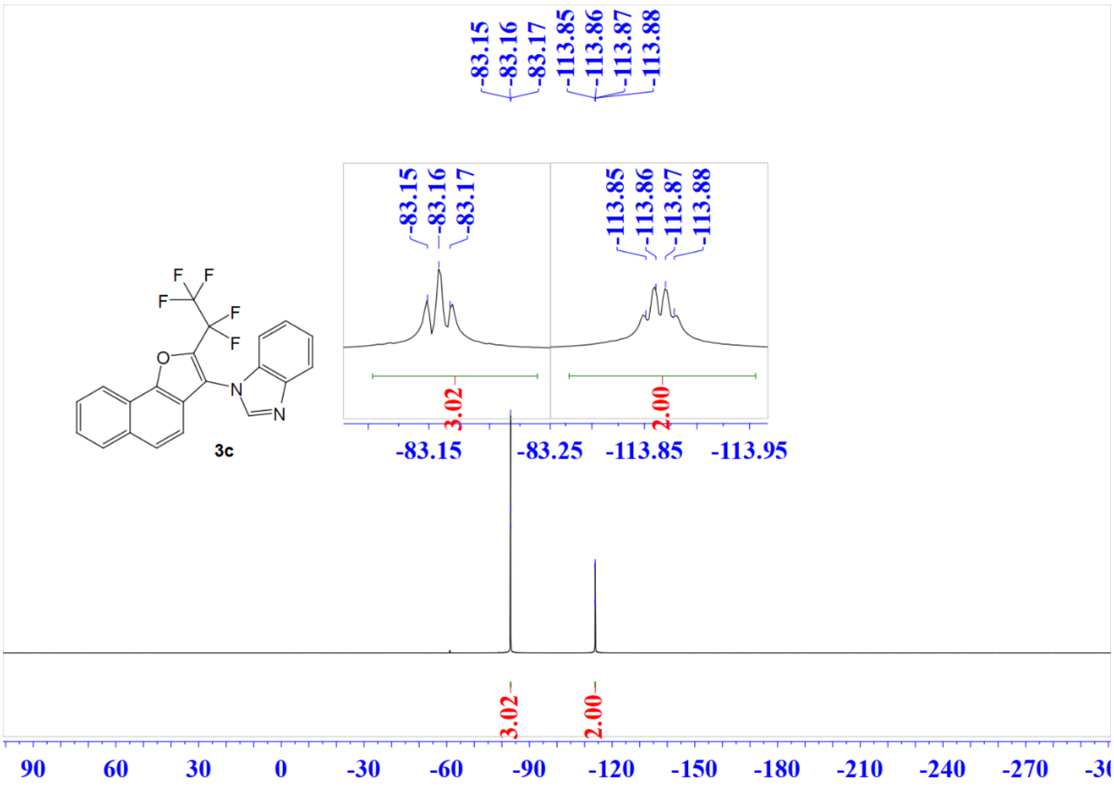

Figure S9. ^{13}C NMR spectrum of **3c**, related to **Scheme 1**.

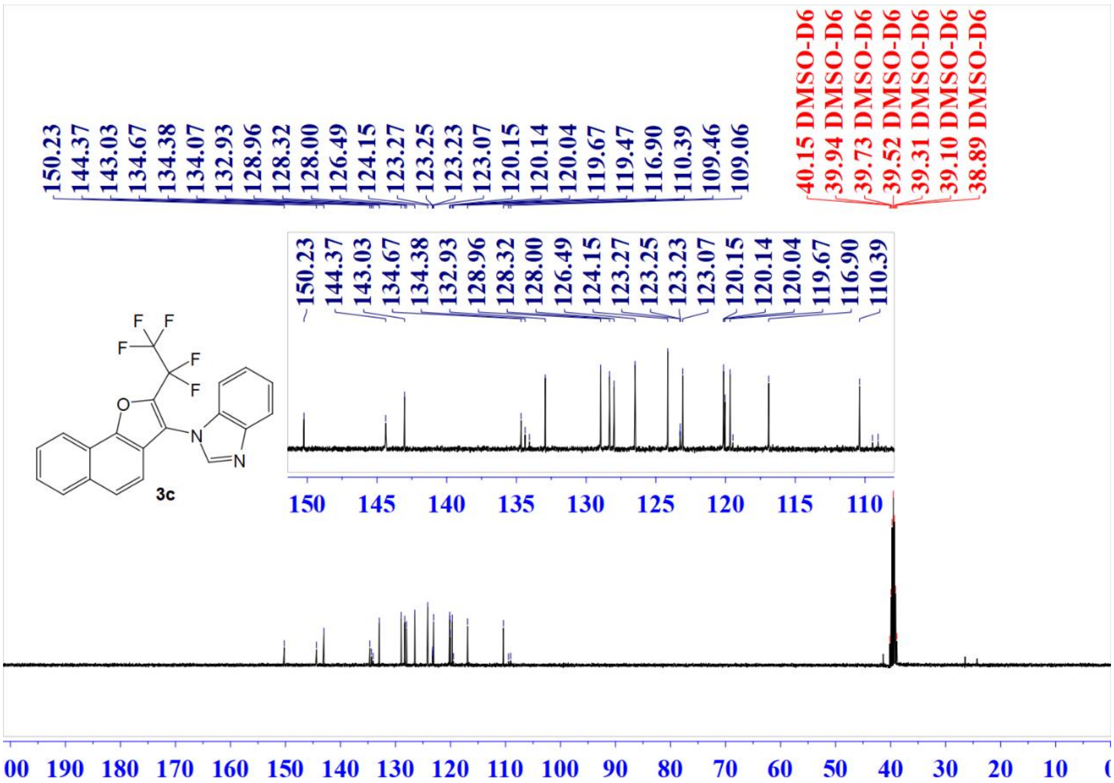

Figure S10. ^{1}H NMR spectrum of **3d-I** or **3d-II**, related to **Scheme 1**.

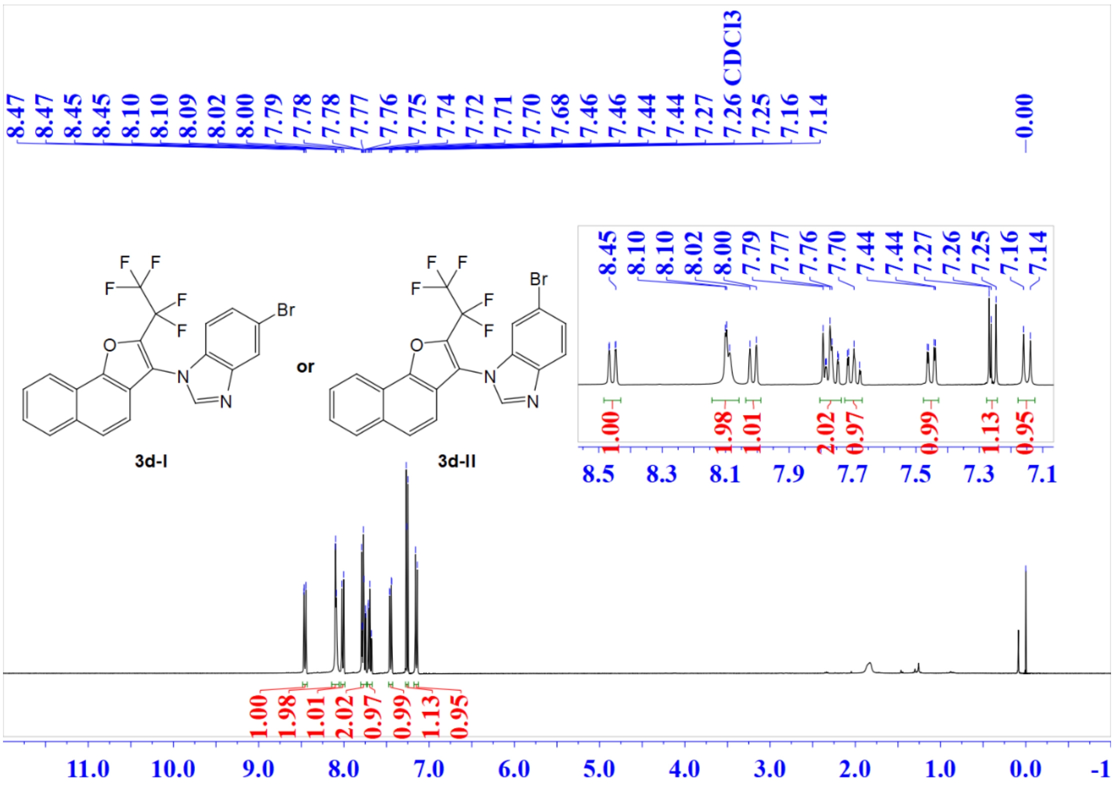

Figure S11. ^{19}F NMR spectrum of **3d-I** or **3d-II**, related to **Scheme 1**.

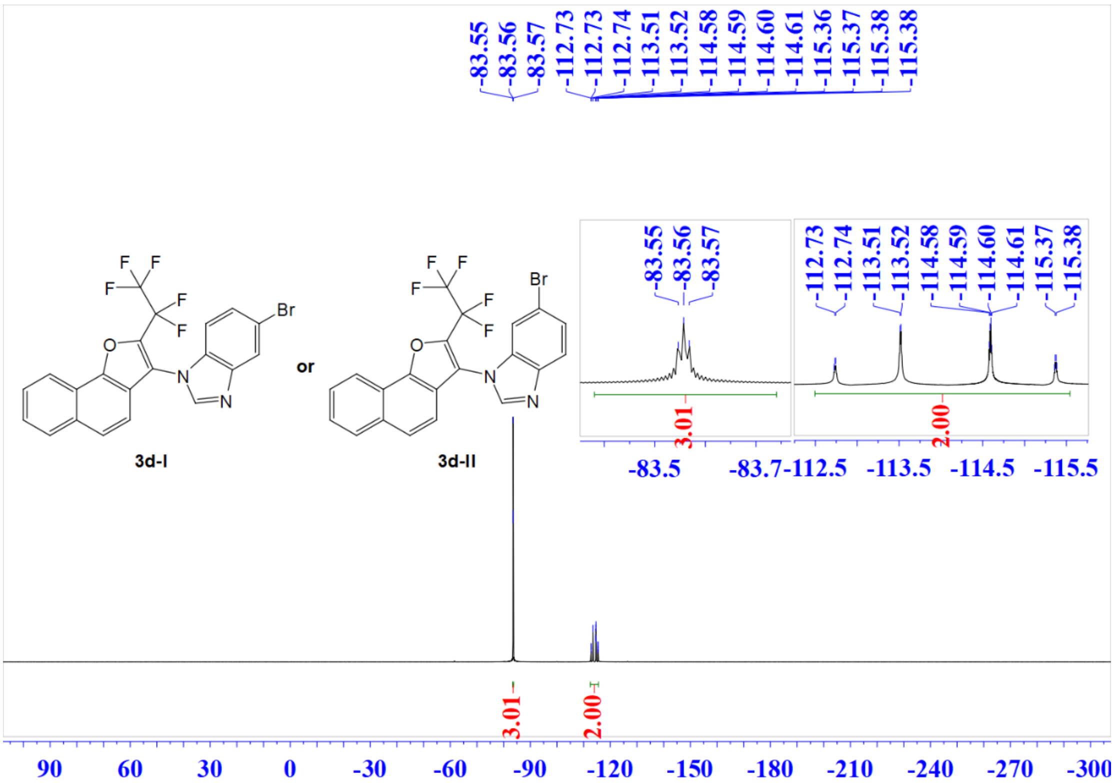

Figure S12. ^{13}C NMR spectrum of **3d-I** or **3d-II**, related to **Scheme 1**.

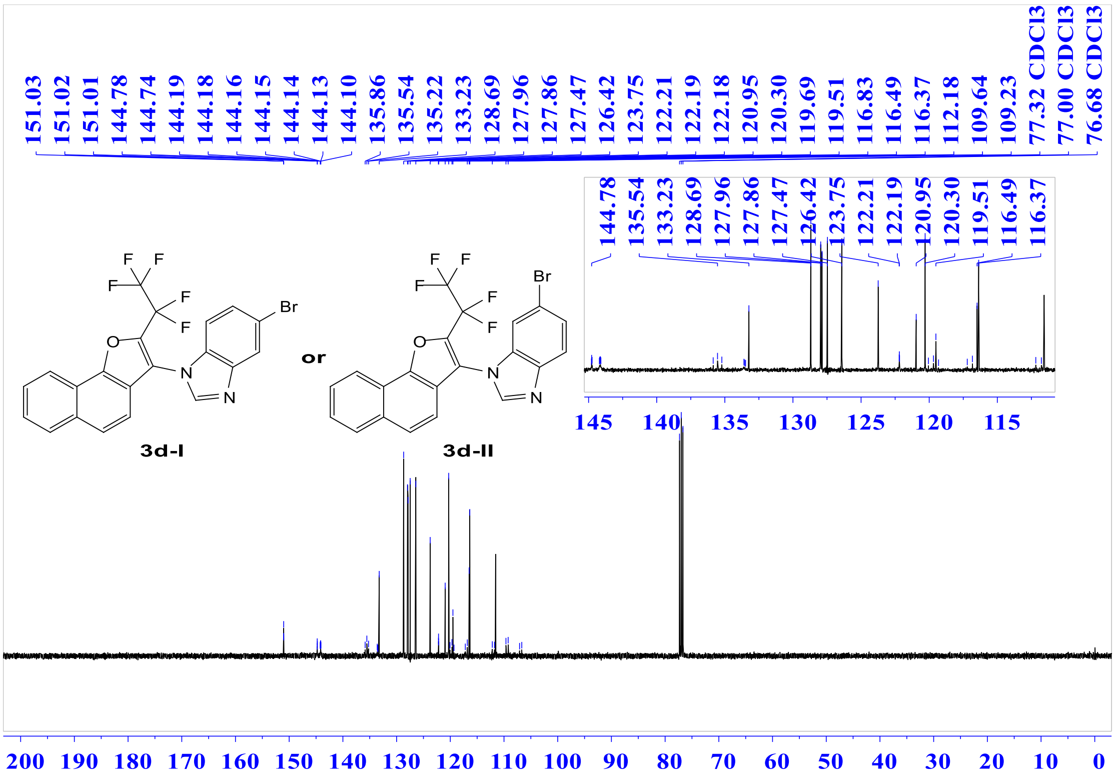

Figure S13. ^{1}H NMR spectrum of **3d-I** or **3d-II**, related to **Scheme 1**.

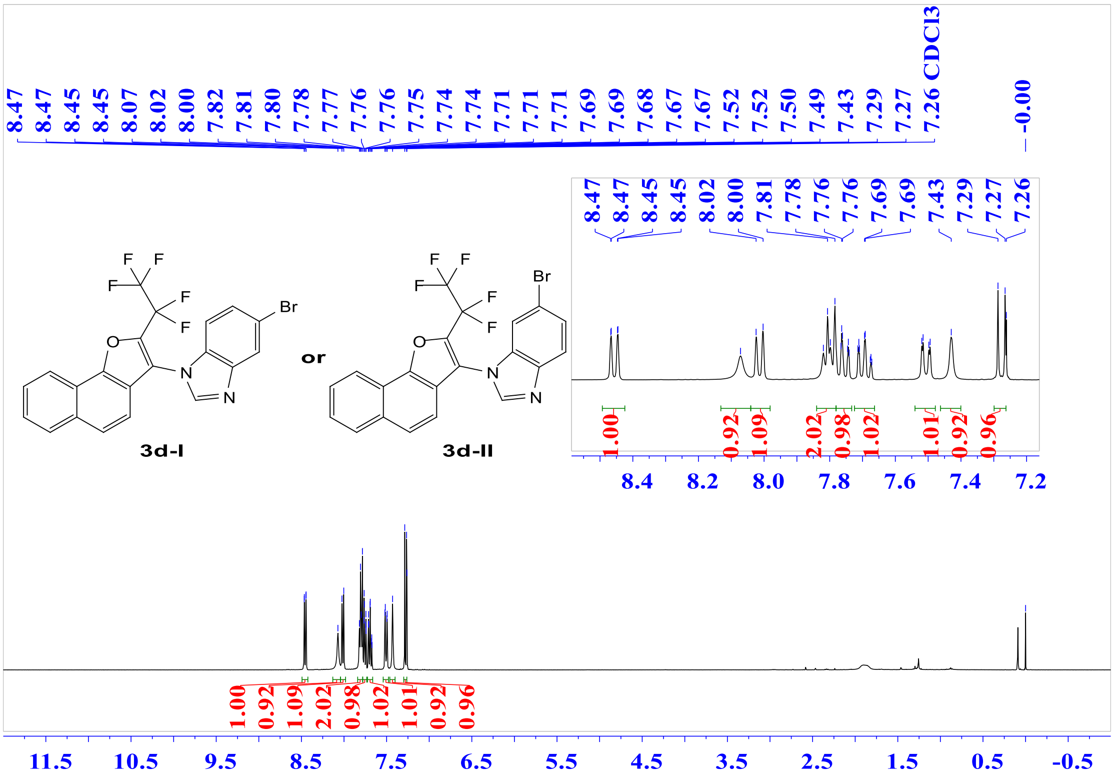

Figure S14. ^{19}F NMR spectrum of **3d-I** or **3d-II**, related to **Scheme 1**.

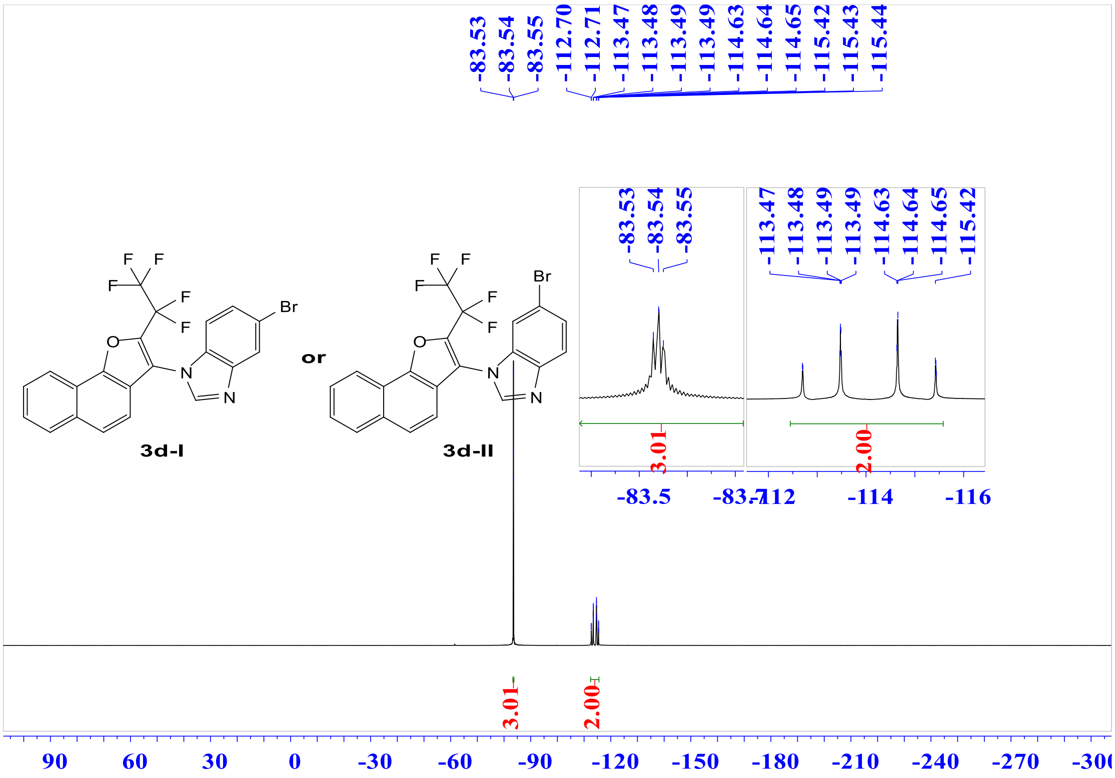

Figure S15. ^{13}C NMR spectrum of **3d-I** or **3d-II**, related to **Scheme 1**.

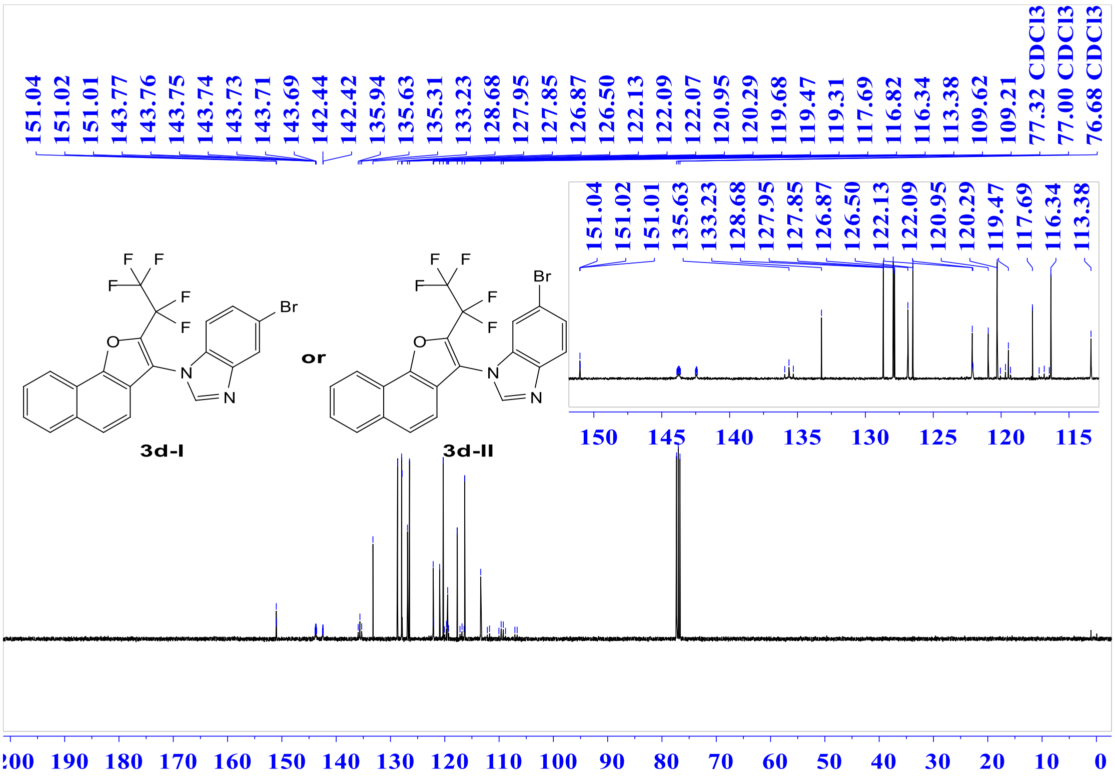

Figure S16. ^{1}H NMR spectrum of **3e-I** and **3e-II**, related to **Scheme 1**.

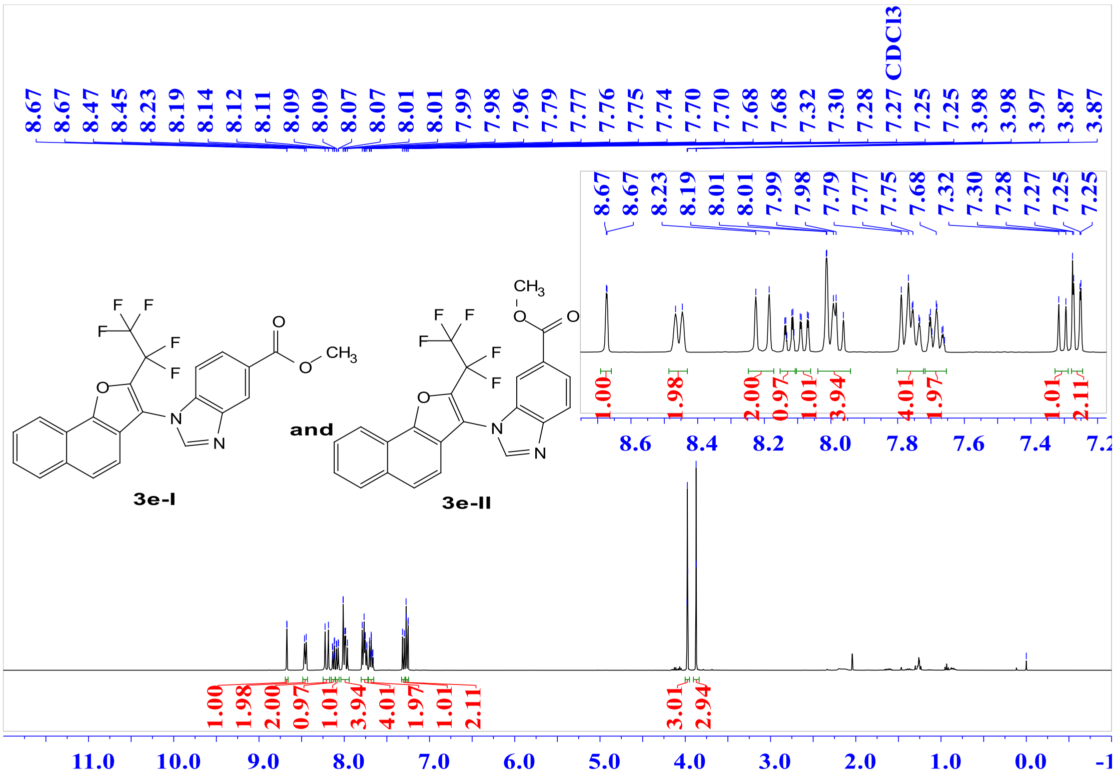

Figure S17. ^{19}F NMR spectrum of **3e-I** and **3e-II**, related to **Scheme 1**.

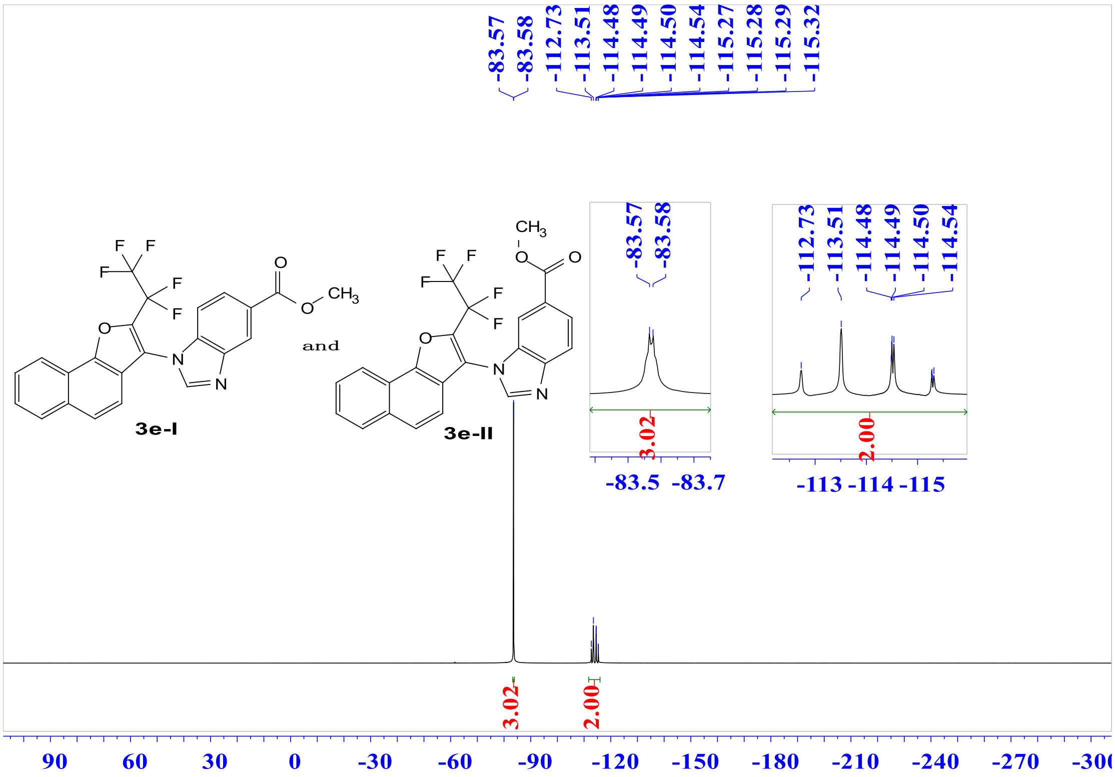

Figure S18. ^{13}C NMR spectrum of **3e-I** and **3e-II**, related to **Scheme 1**.

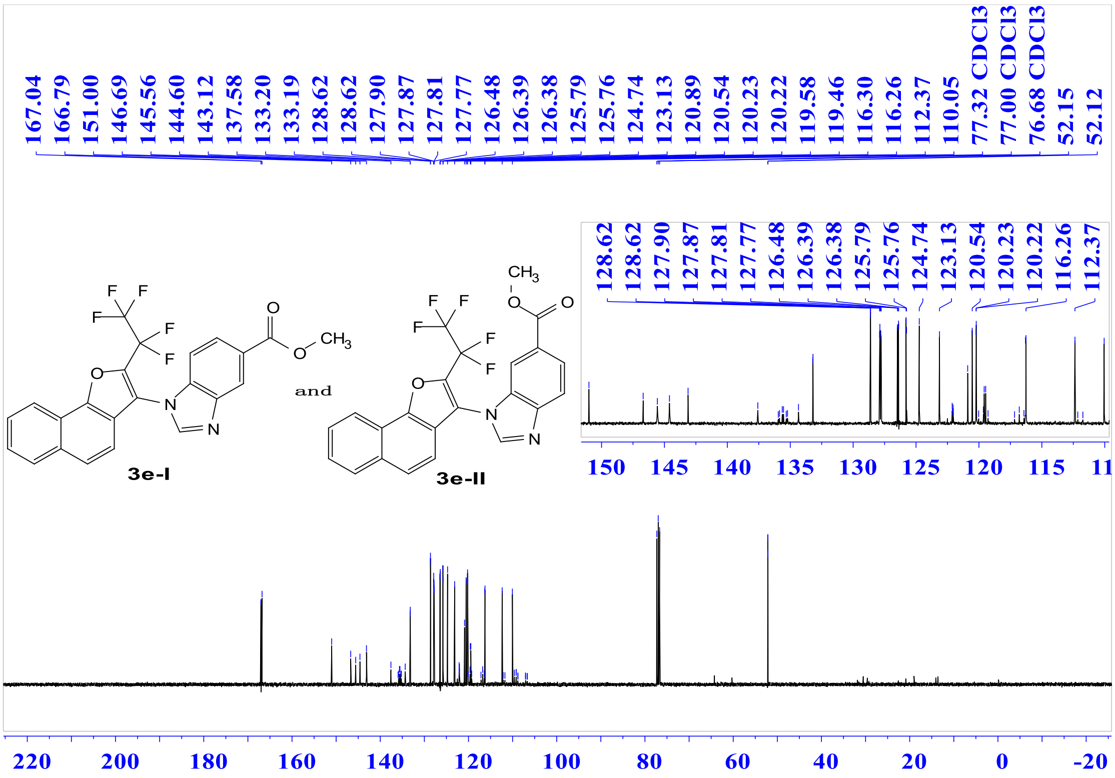

Figure S19. ^{1}H NMR spectrum of **3f-I** or **3f-II**, related to **Scheme 1**.

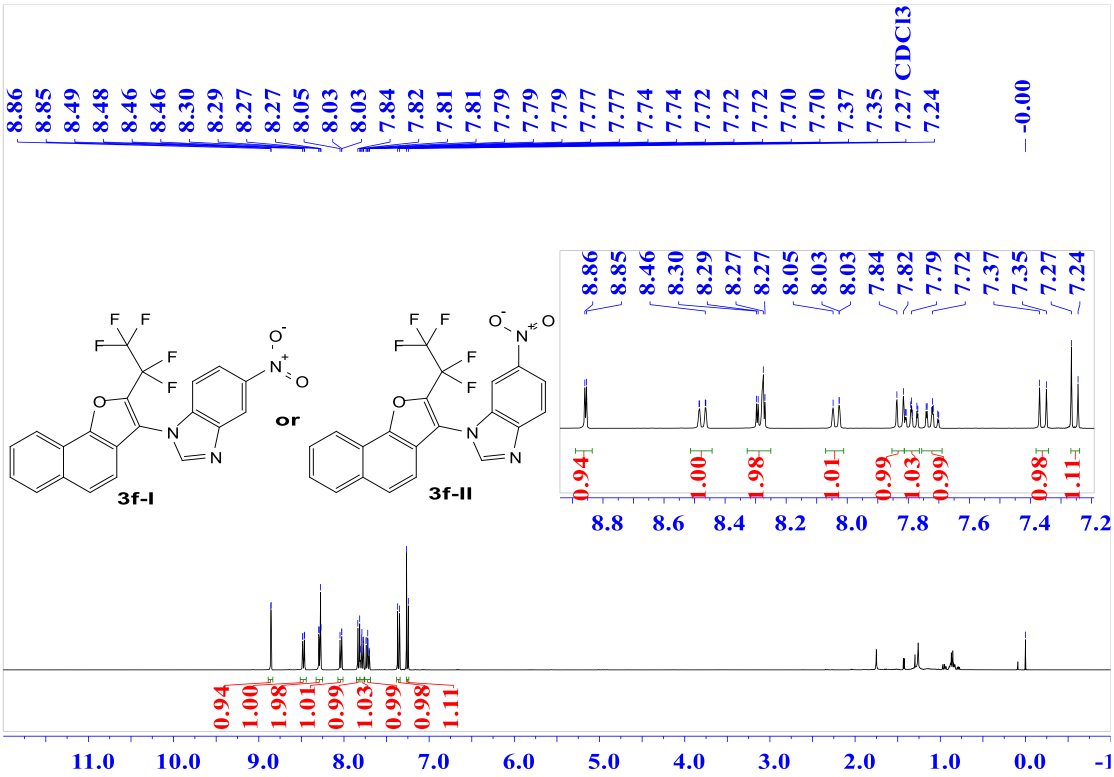

Figure S20. ^{19}F NMR spectrum of **3f-I** or **3f-II**, related to **Scheme 1**.

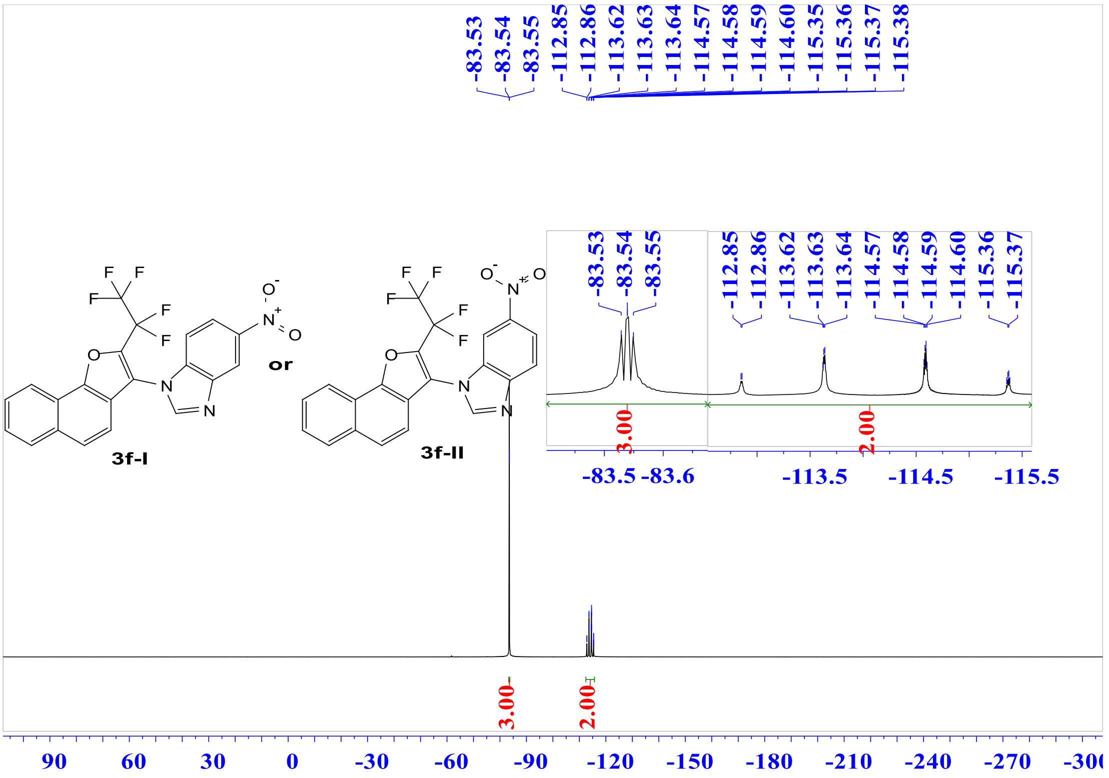

Figure S21. ^{13}C NMR spectrum of **3f-I** or **3f-II**, related to **Scheme 1**.

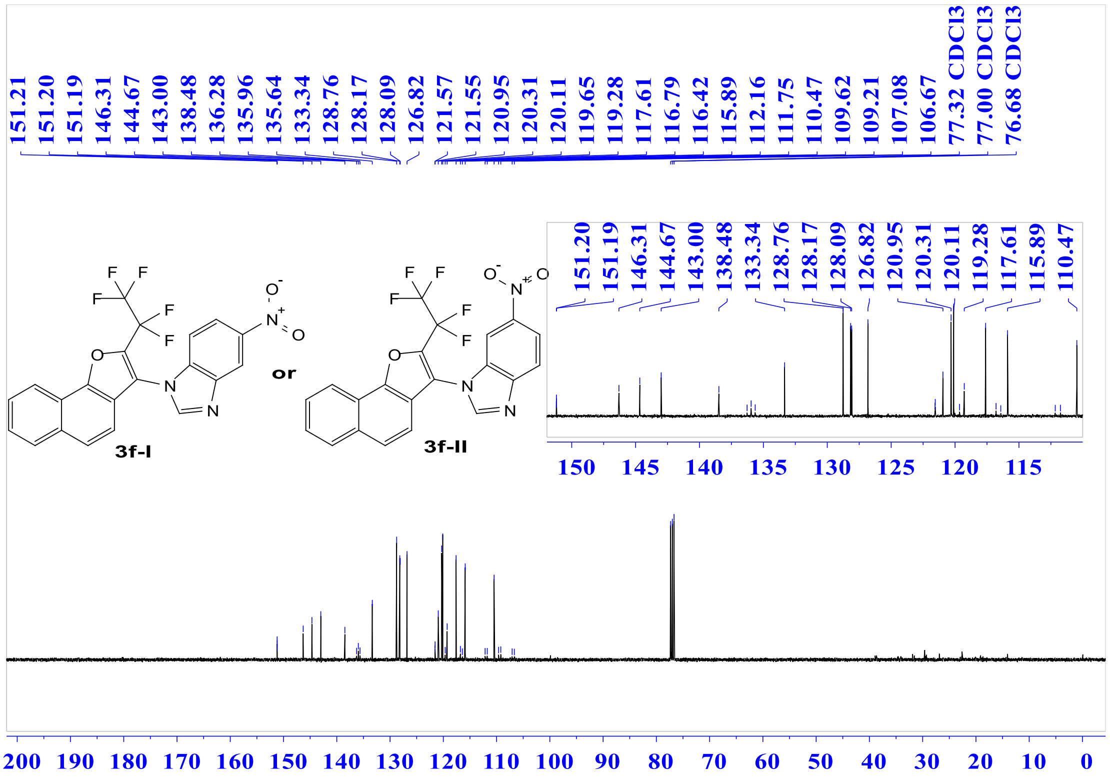

Figure S22. ^{1}H NMR spectrum of **3f-I** or **3f-II**, related to **Scheme 1**.

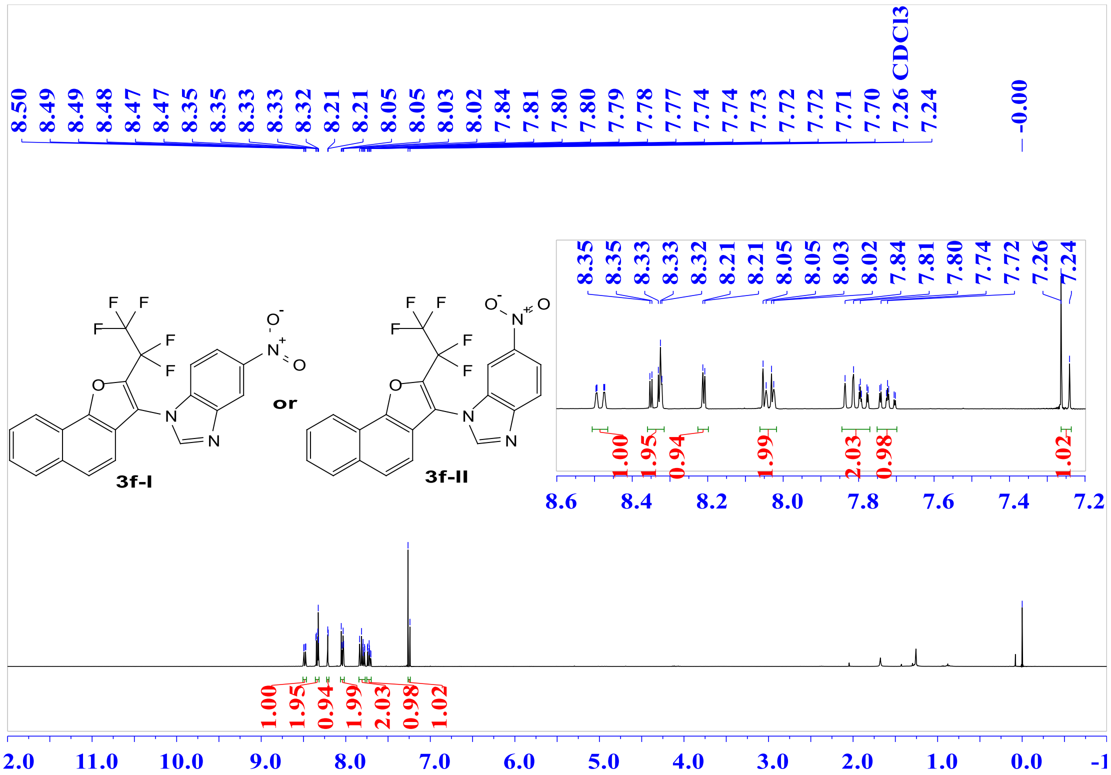

Figure S23. ^{19}F NMR spectrum of **3f-I** or **3f-II**, related to **Scheme 1**.

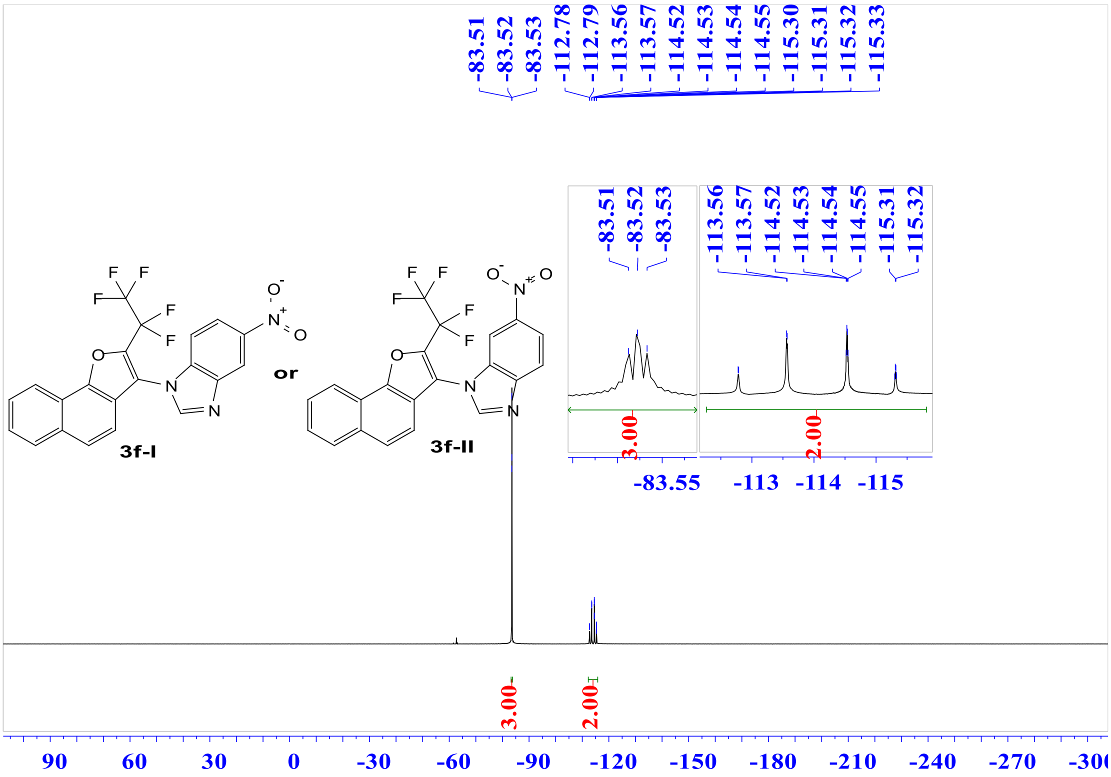

Figure S24. ^{13}C NMR spectrum of **3f-I** or **3f-II**, related to **Scheme 1**.

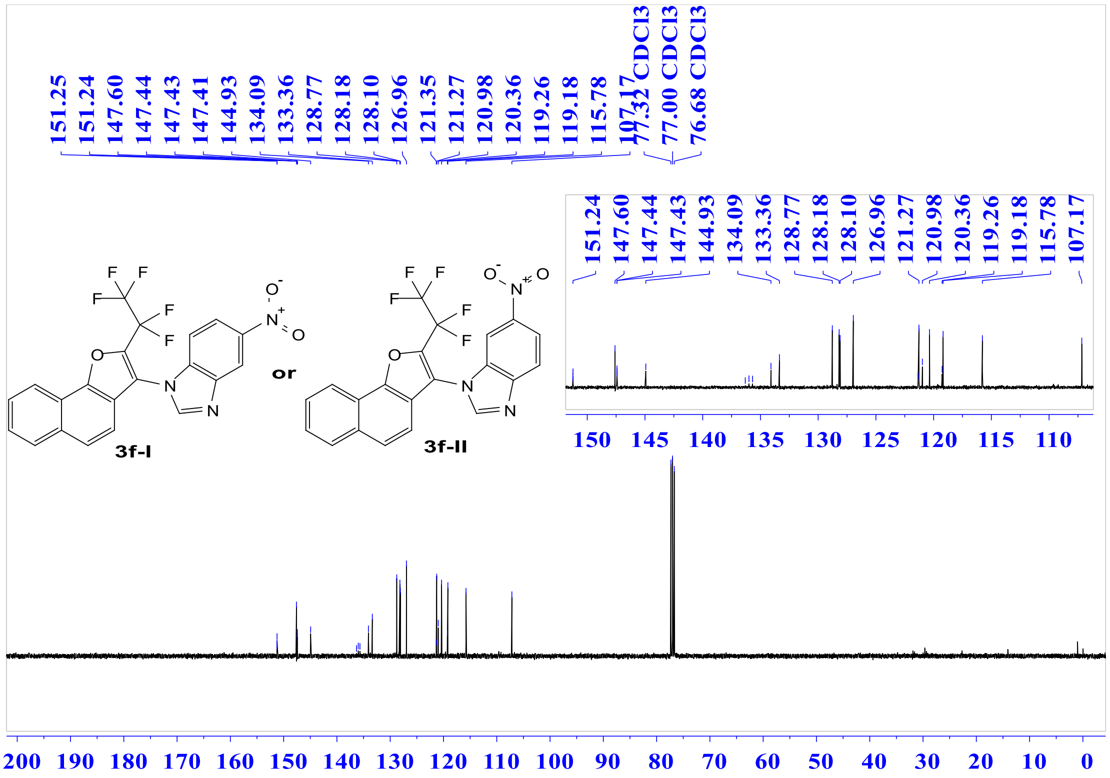

Figure S25. ^{1}H NMR spectrum of **3g**, related to **Scheme 1**.

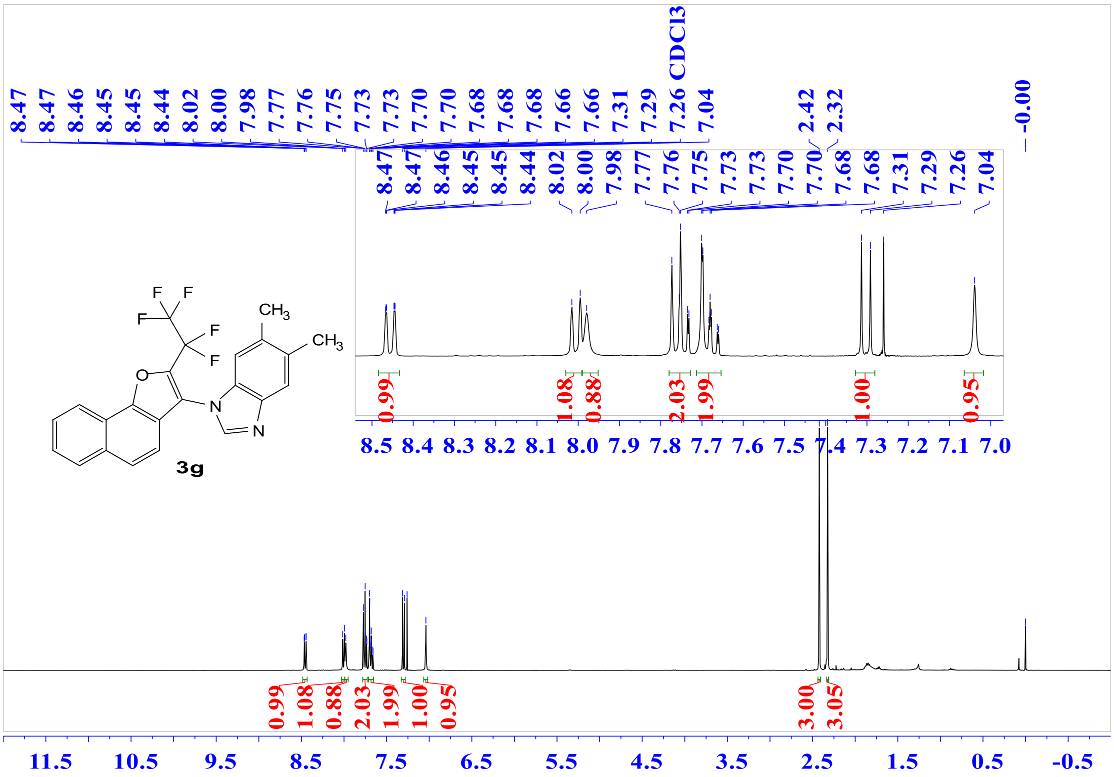

Figure S26. ^{19}F NMR spectrum of **3g**, related to **Scheme 1**.

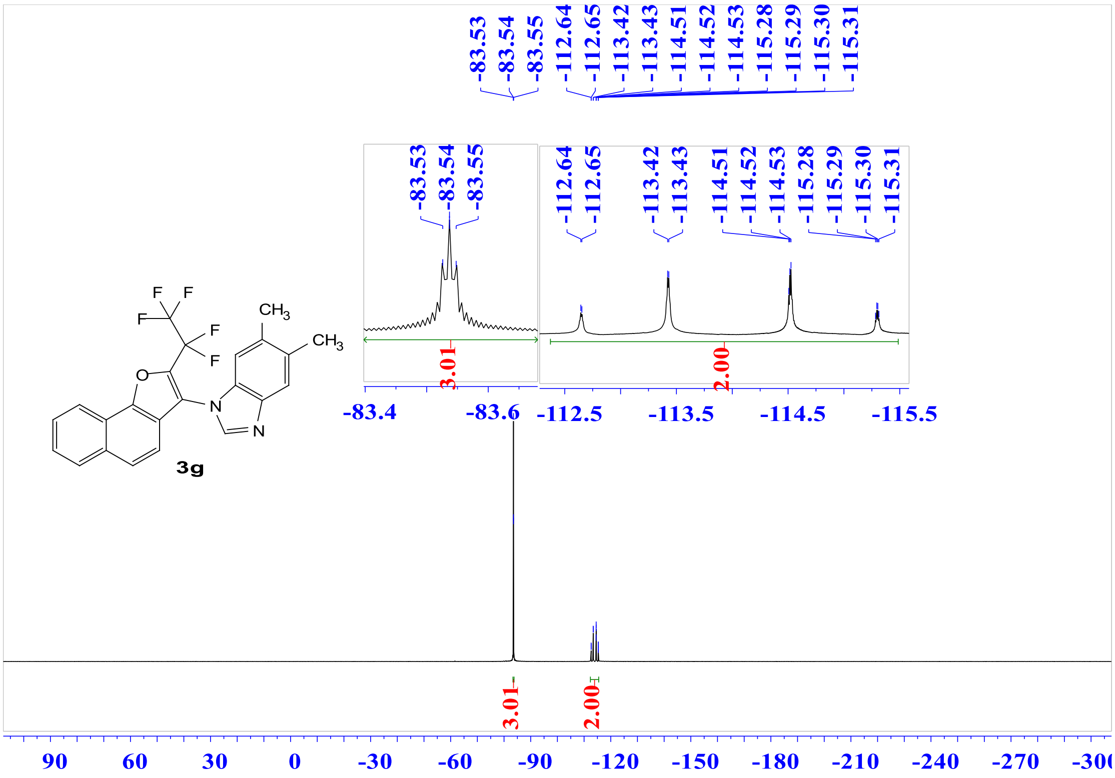

Figure S27. ^{13}C NMR spectrum of **3g**, related to **Scheme 1**.

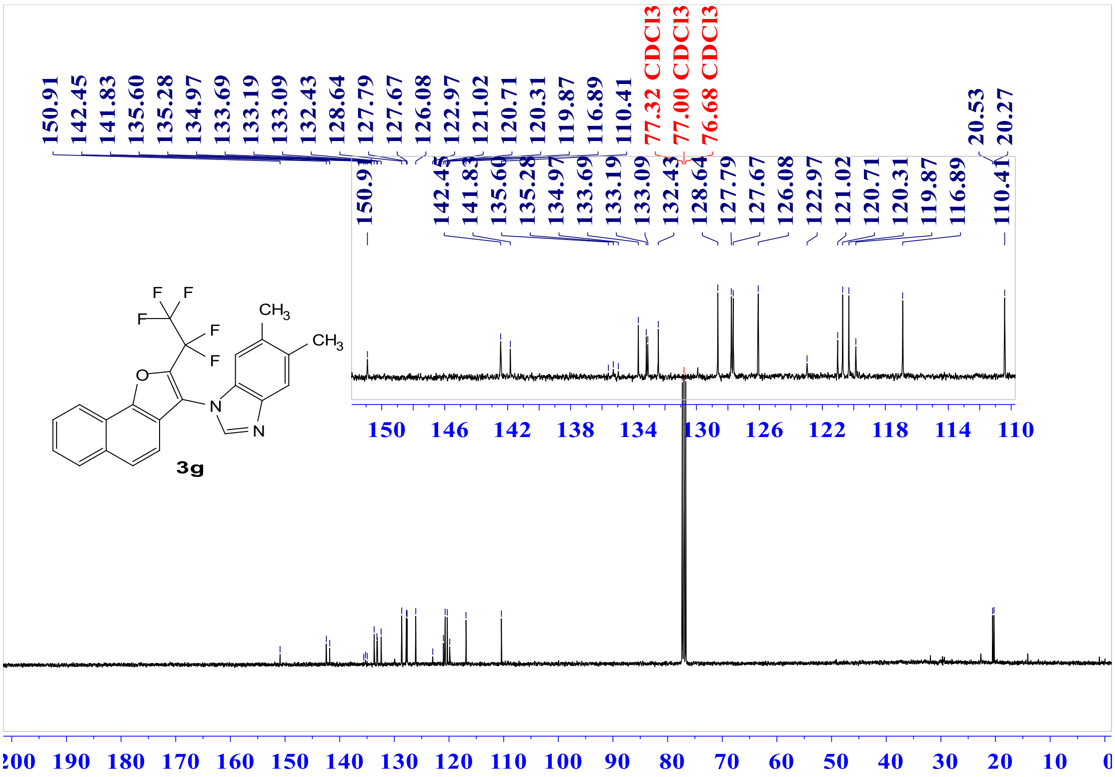

Figure S28. ^{1}H NMR spectrum of **3h-I** or **3h-II**, related to **Scheme 1**.

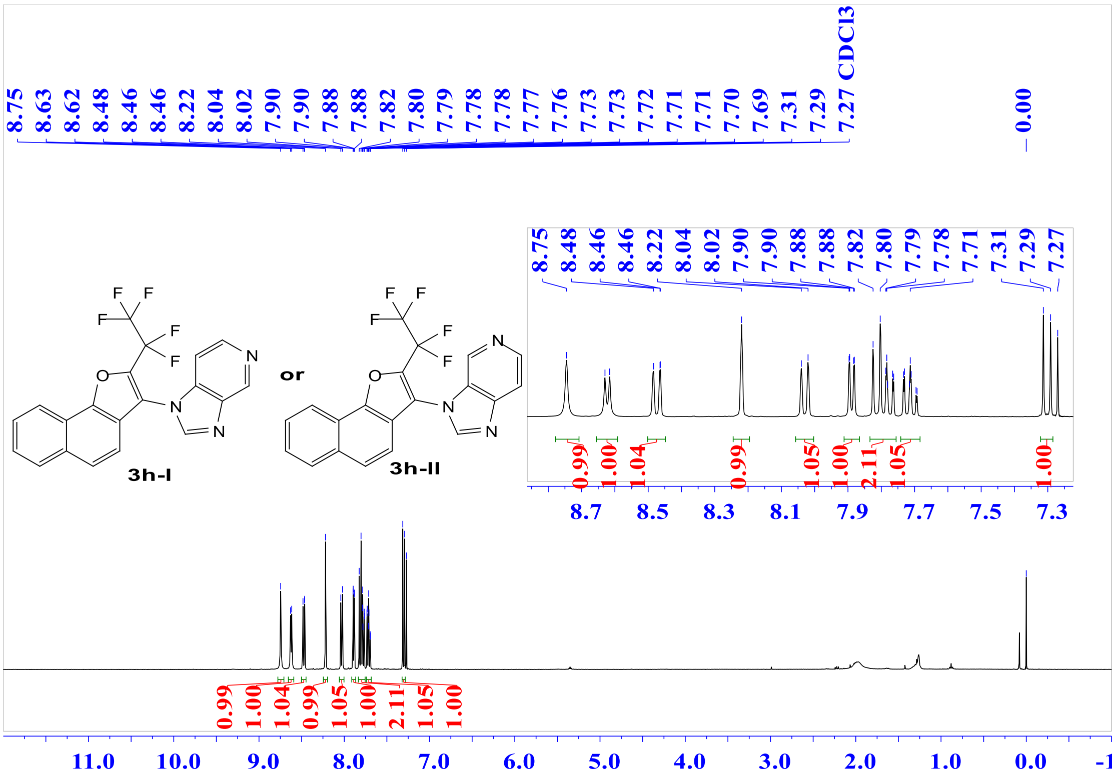

Figure S29. ^{19}F NMR spectrum of **3h-I** or **3h-II**, related to **Scheme 1**.

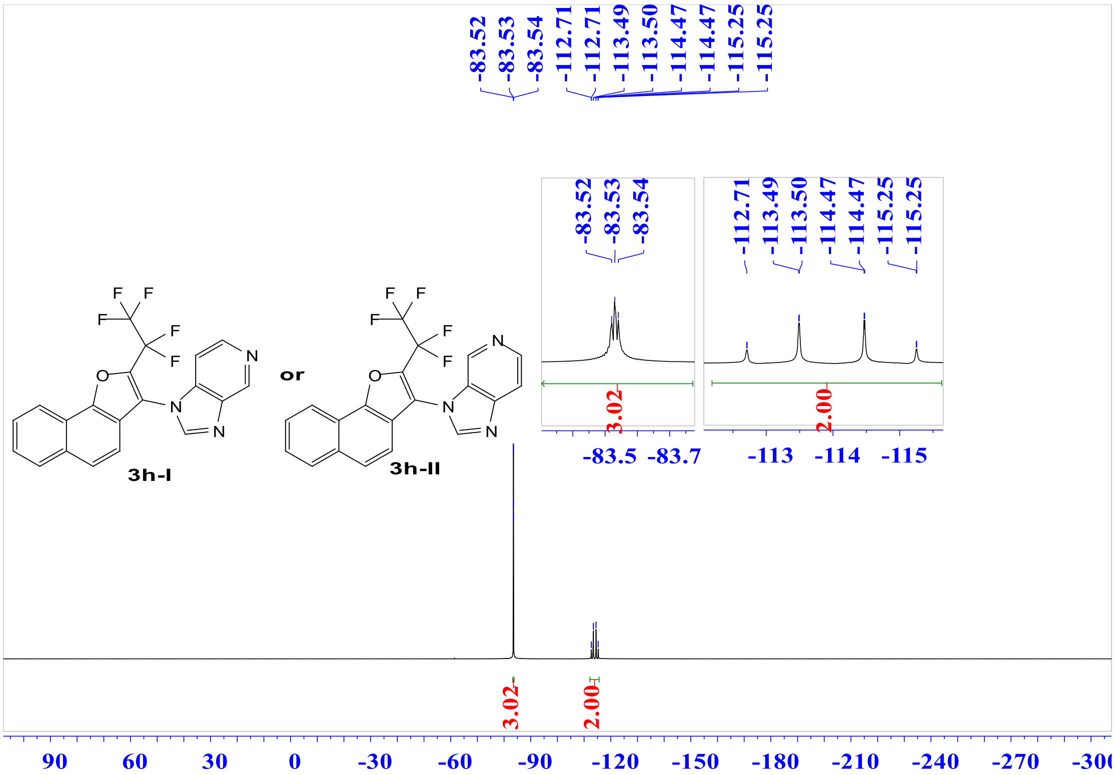

Figure S30. ^{13}C NMR spectrum of **3h-I** or **3h-II**, related to **Scheme 1**.

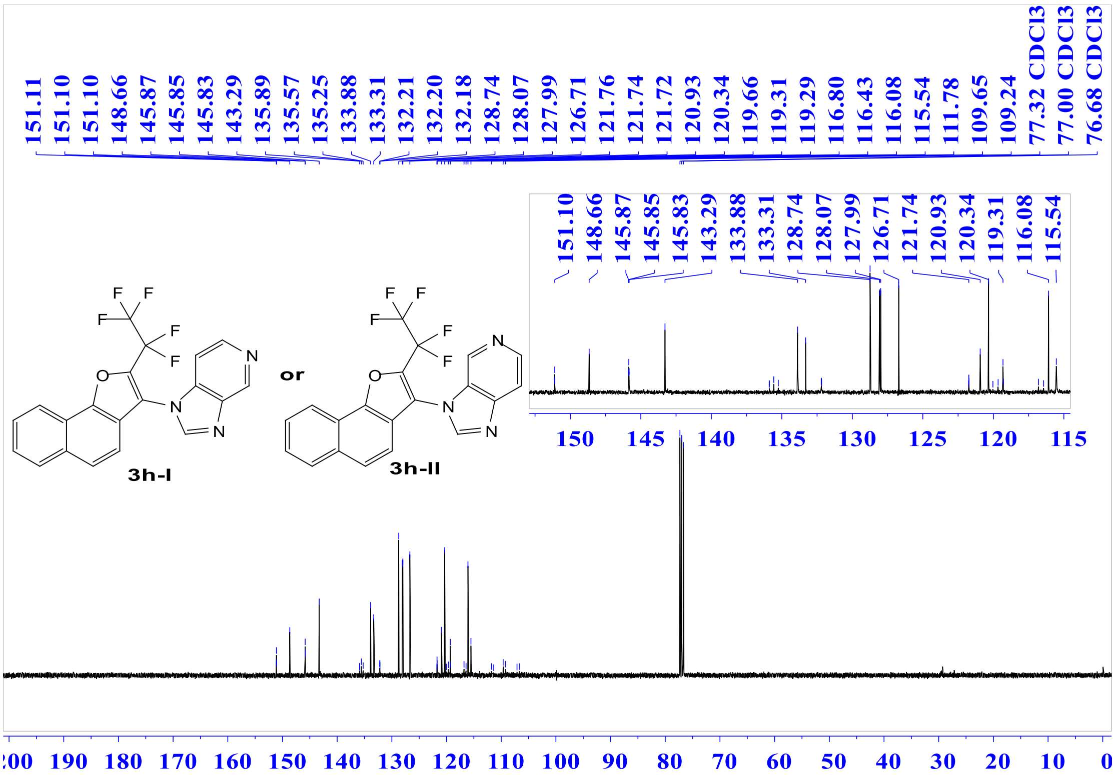

Figure S31. ^{1}H NMR spectrum of **3h-I** or **3h-II**, related to **Scheme 1**.

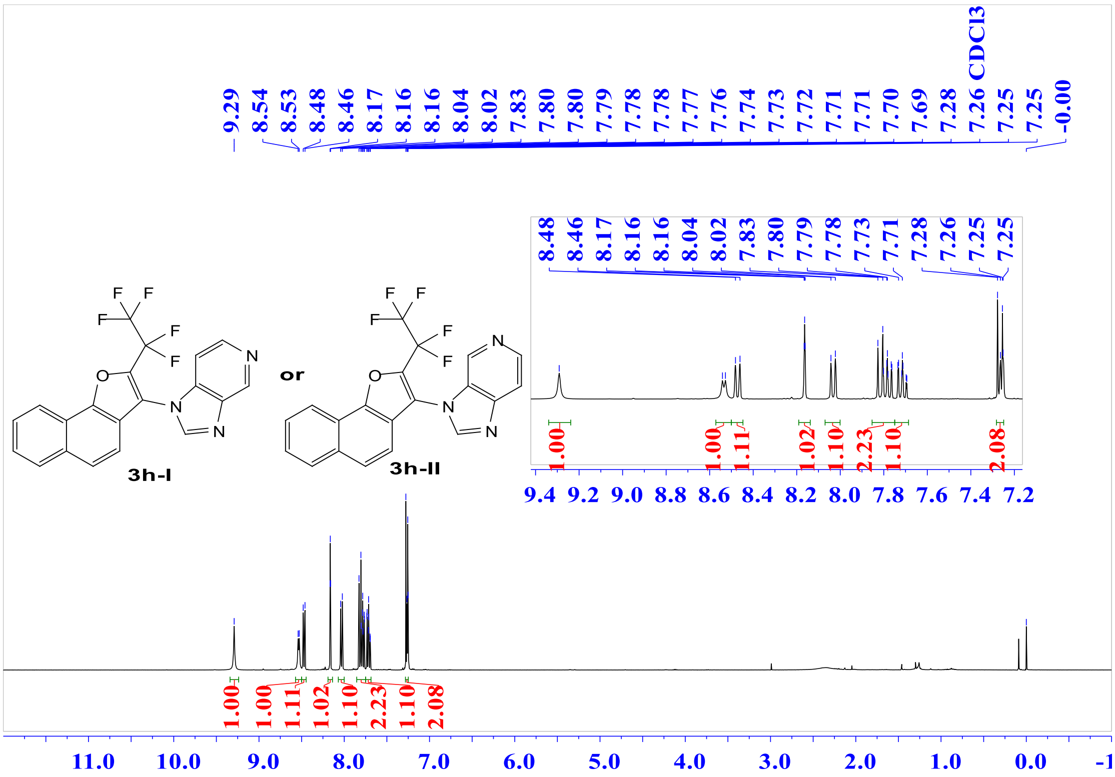

Figure S32. ^{19}F NMR spectrum of **3h-I** or **3h-II**, related to **Scheme 1**.

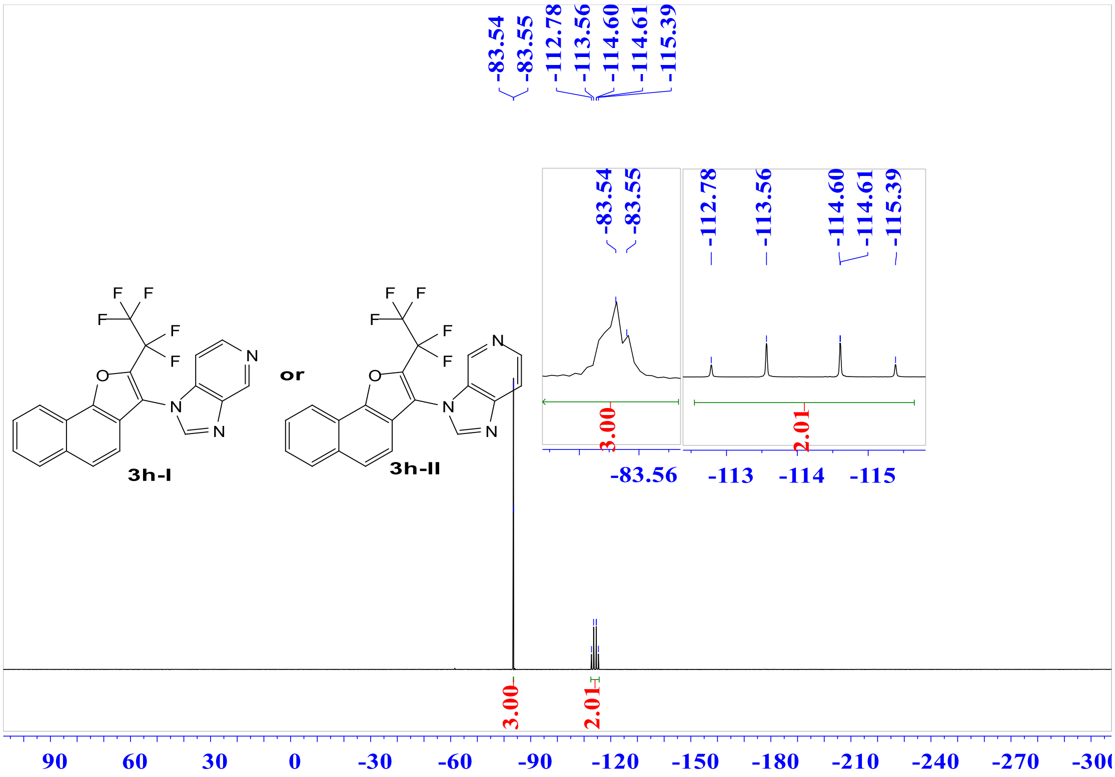

Figure S33. ^{13}C NMR spectrum of **3h-I** or **3h-II**, related to **Scheme 1**.

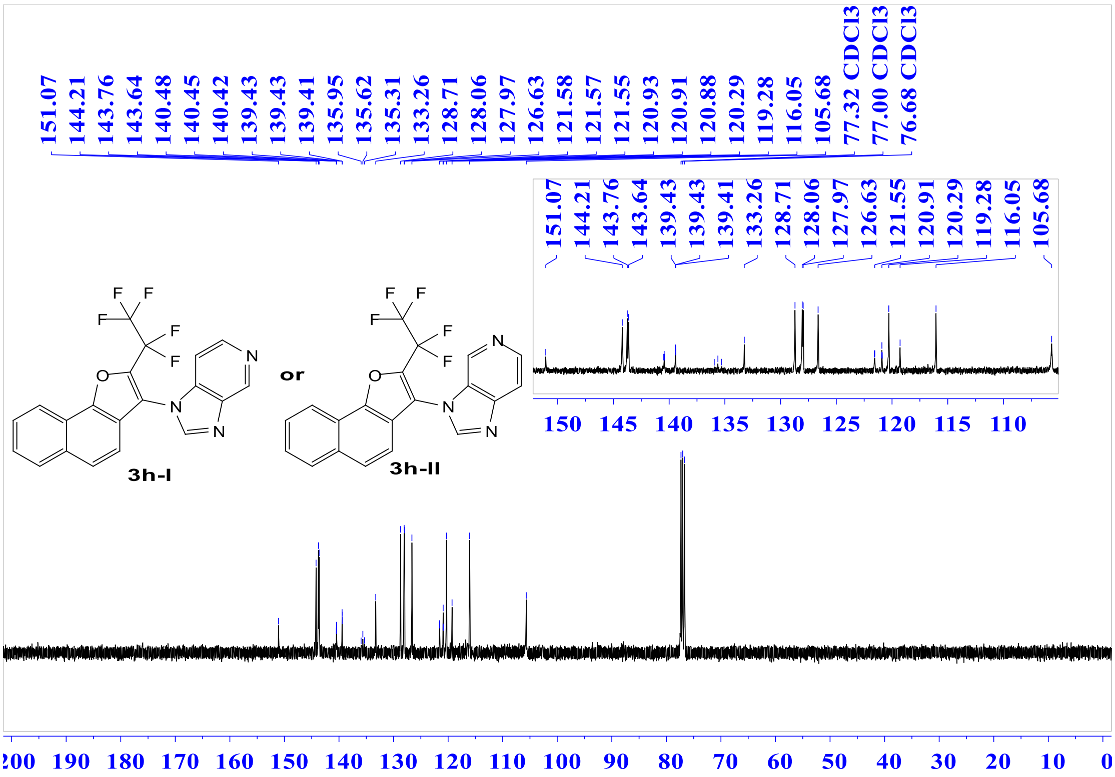

Figure S34. ^{1}H NMR spectrum of **4a**, related to **Scheme 1**.

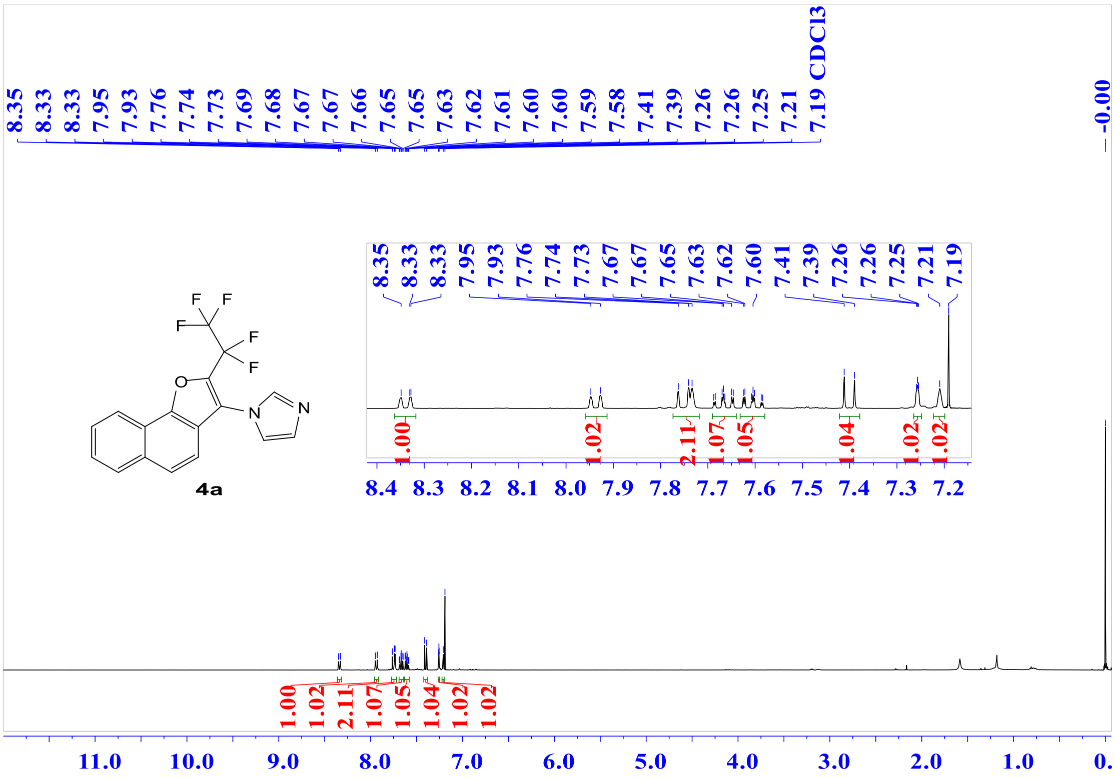

Figure S35. ^{19}F NMR spectrum of **4a**, related to **Scheme 1**.

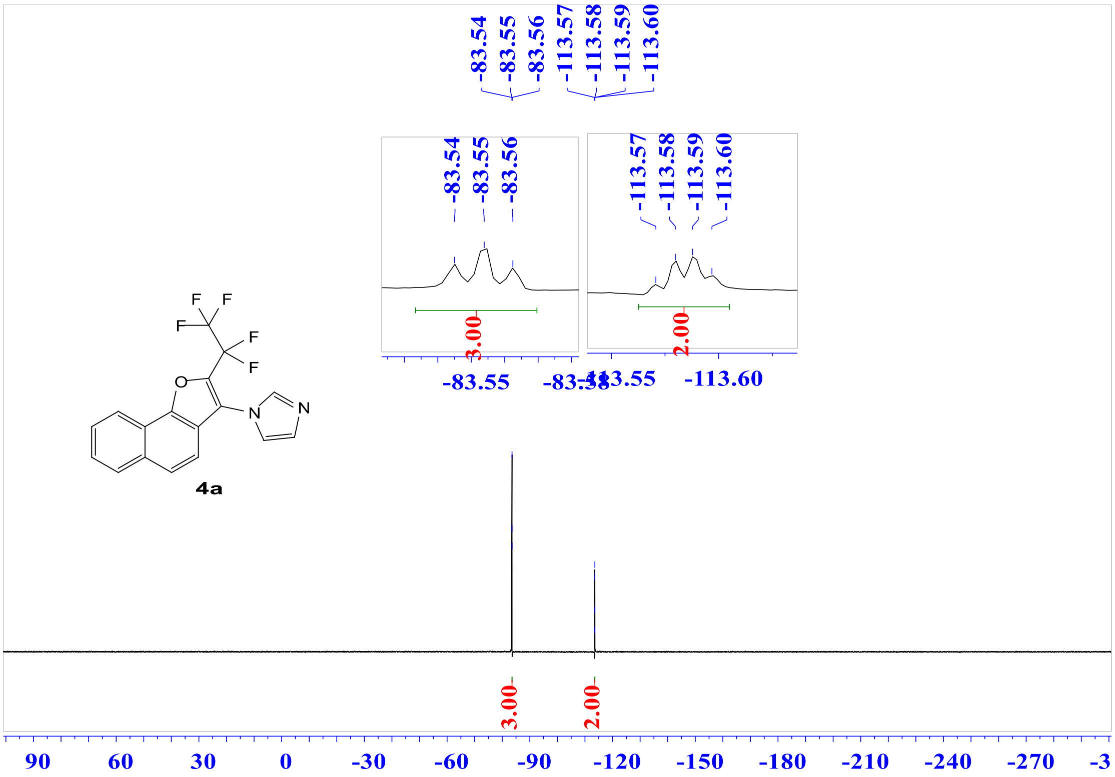

Figure S36. ^{13}C NMR spectrum of **4a**, related to **Scheme 1**.

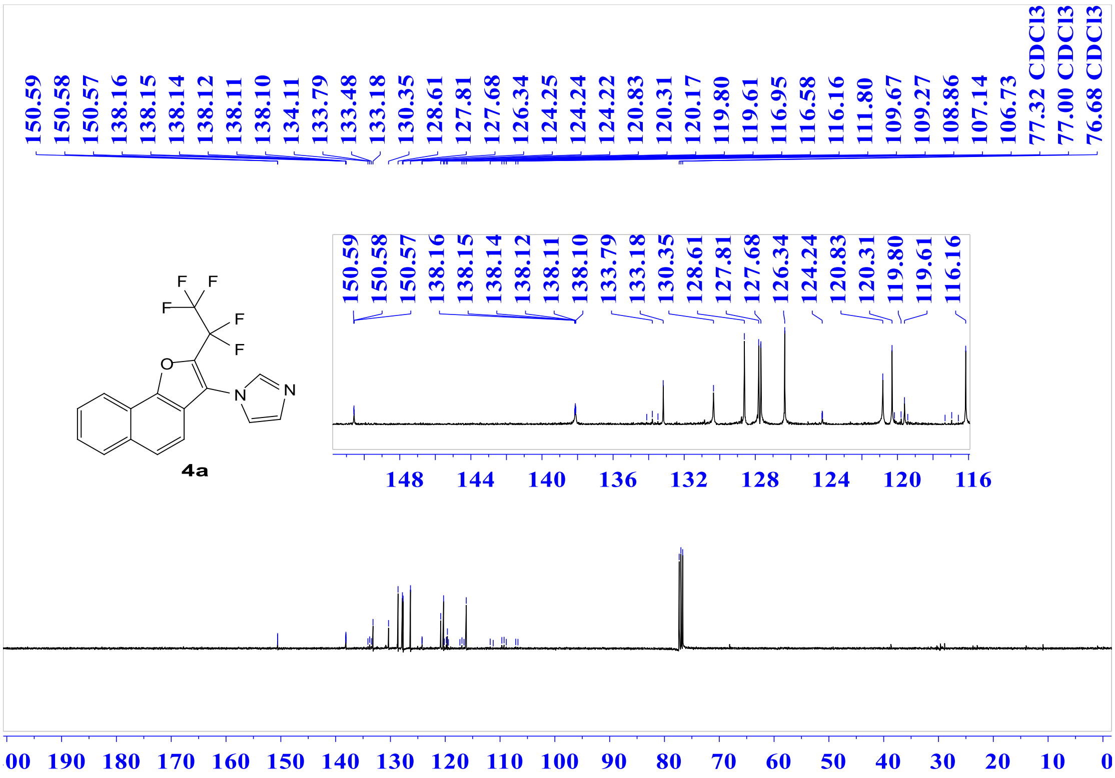

Figure S37. ¹H NMR spectrum of **4b**, related to **Scheme 1**.

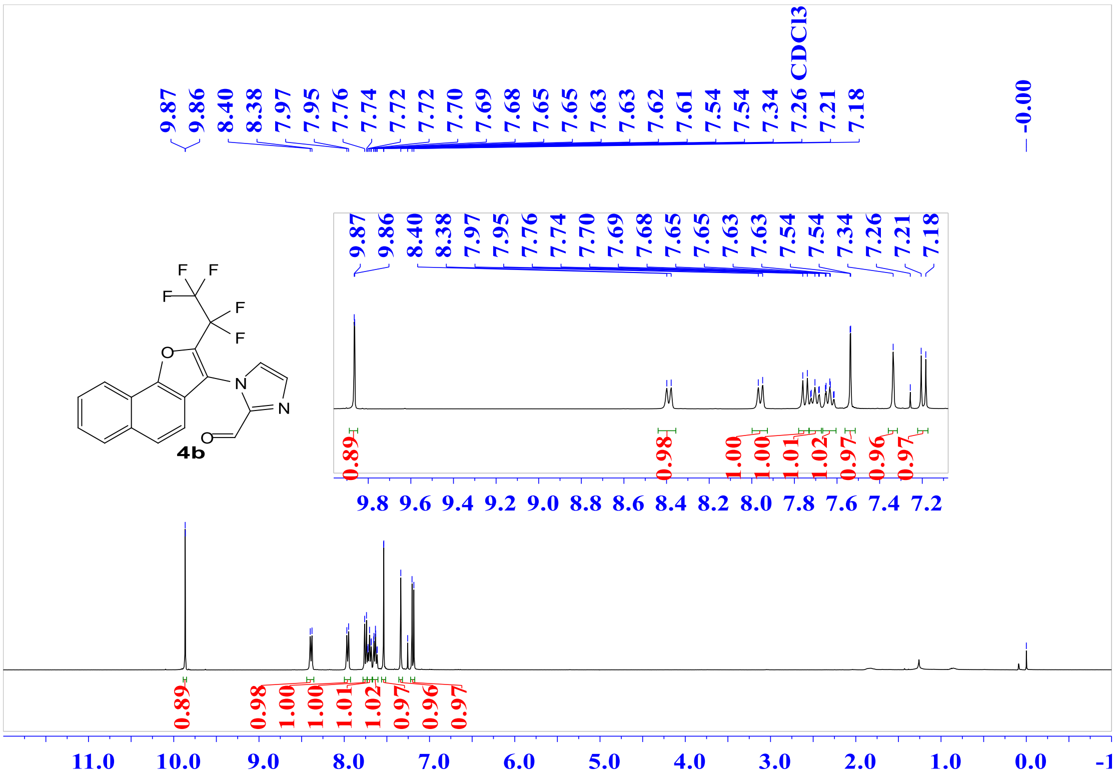

Figure S38. ¹⁹F NMR spectrum of **4b**, related to **Scheme 1**.

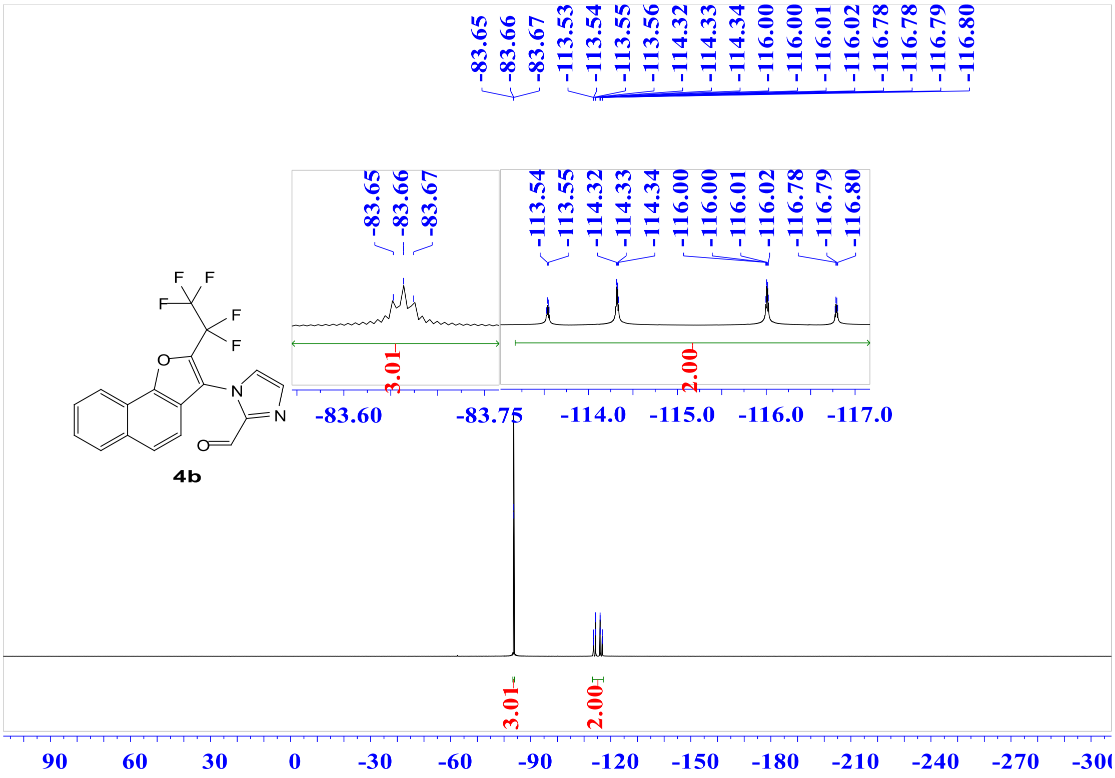

Figure S39. ^{13}C NMR spectrum of **4b**, related to **Scheme 1**.

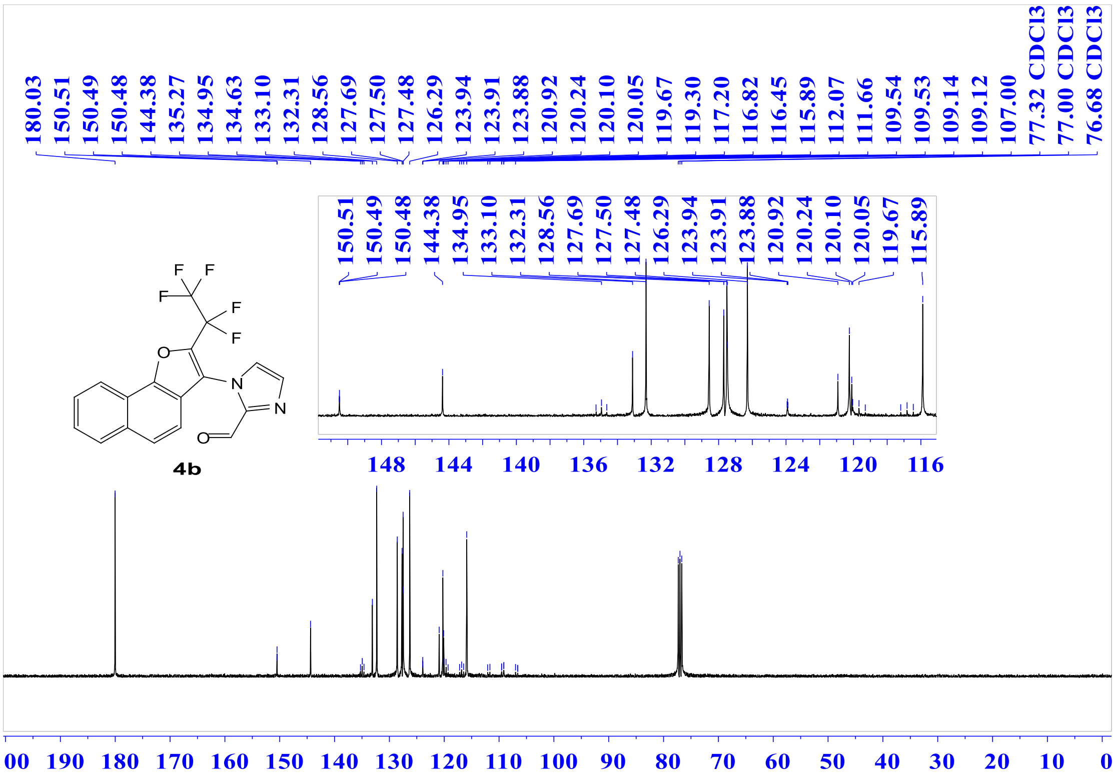

Figure S40. ^{1}H NMR spectrum of **4c**, related to **Scheme 1**.

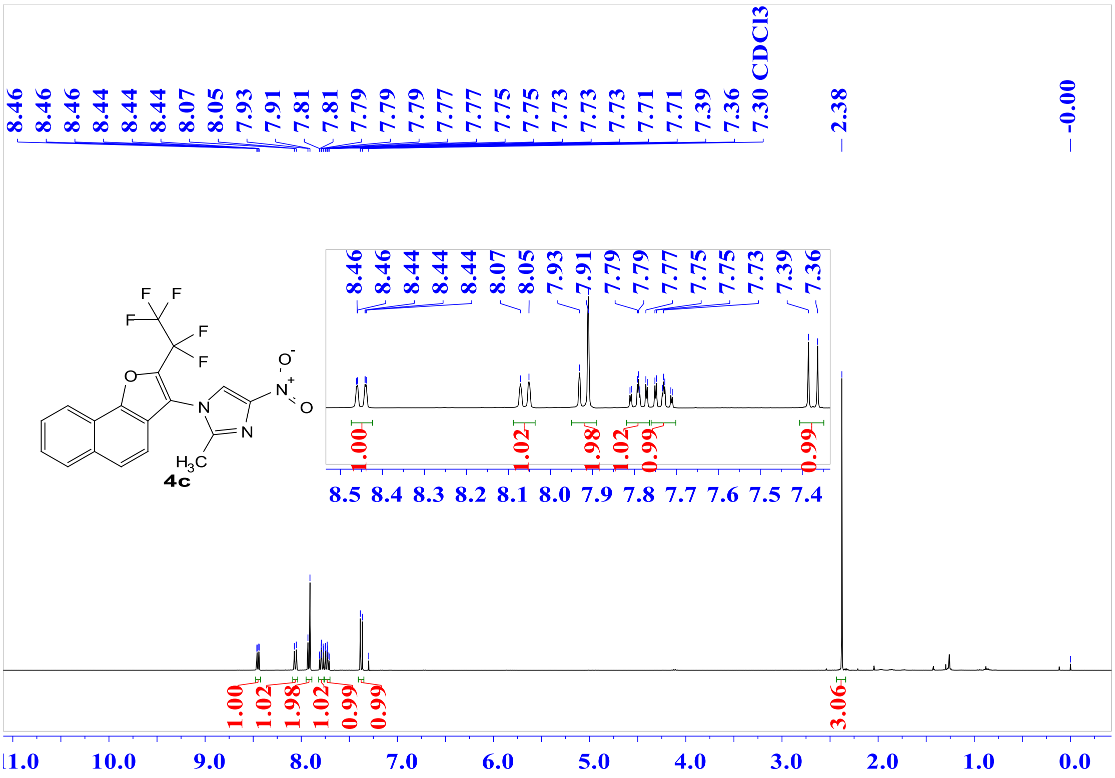

Figure S41. ^{19}F NMR spectrum of **4c**, related to **Scheme 1**.

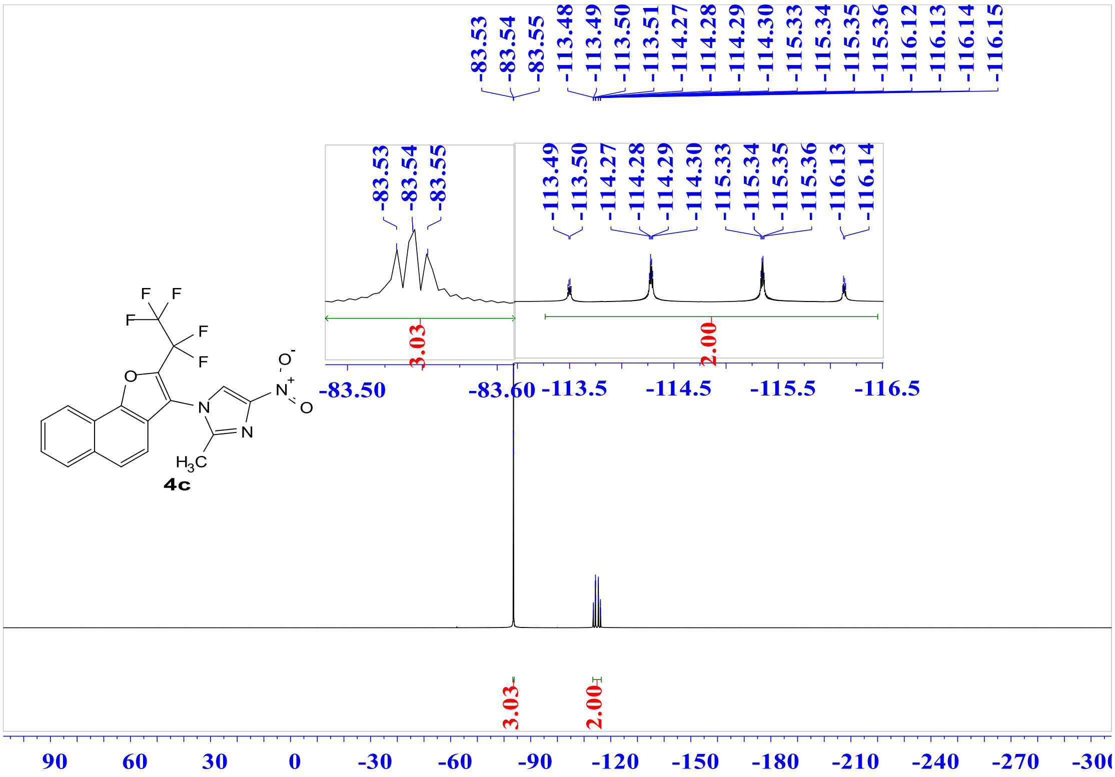

Figure S42. ^{13}C NMR spectrum of **4c**, related to **Scheme 1**.

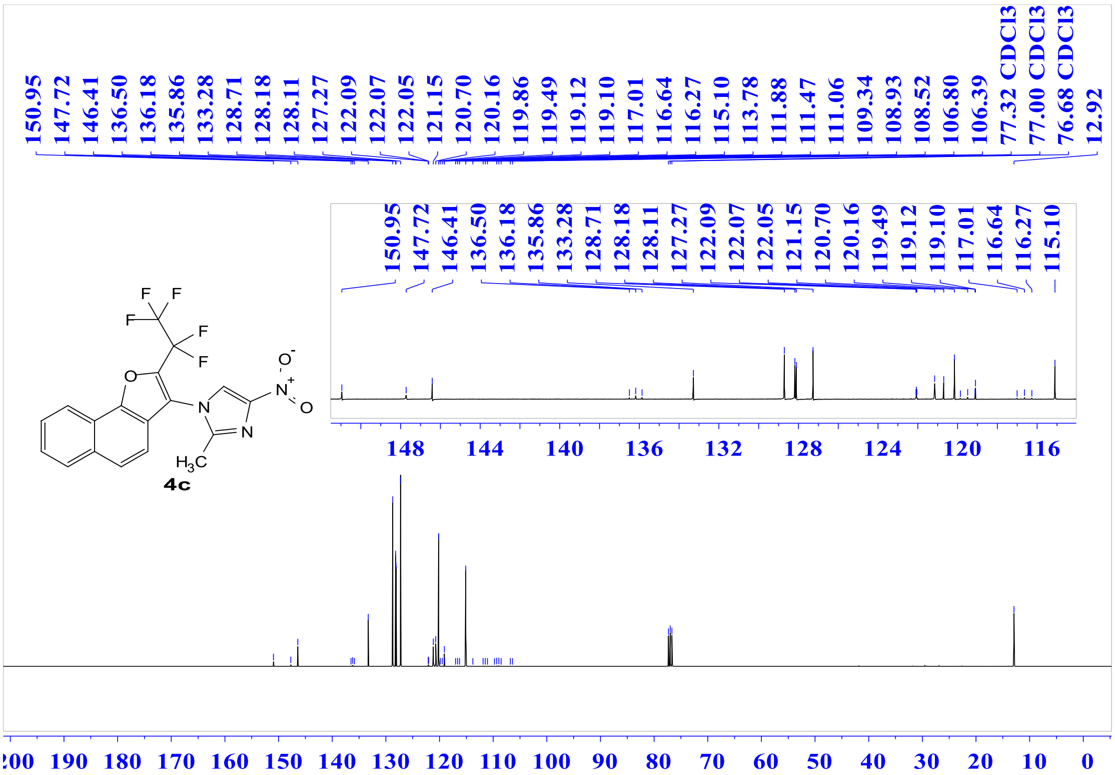

Figure S43. ^{1}H NMR spectrum of **5a**, related to **Scheme 1**.

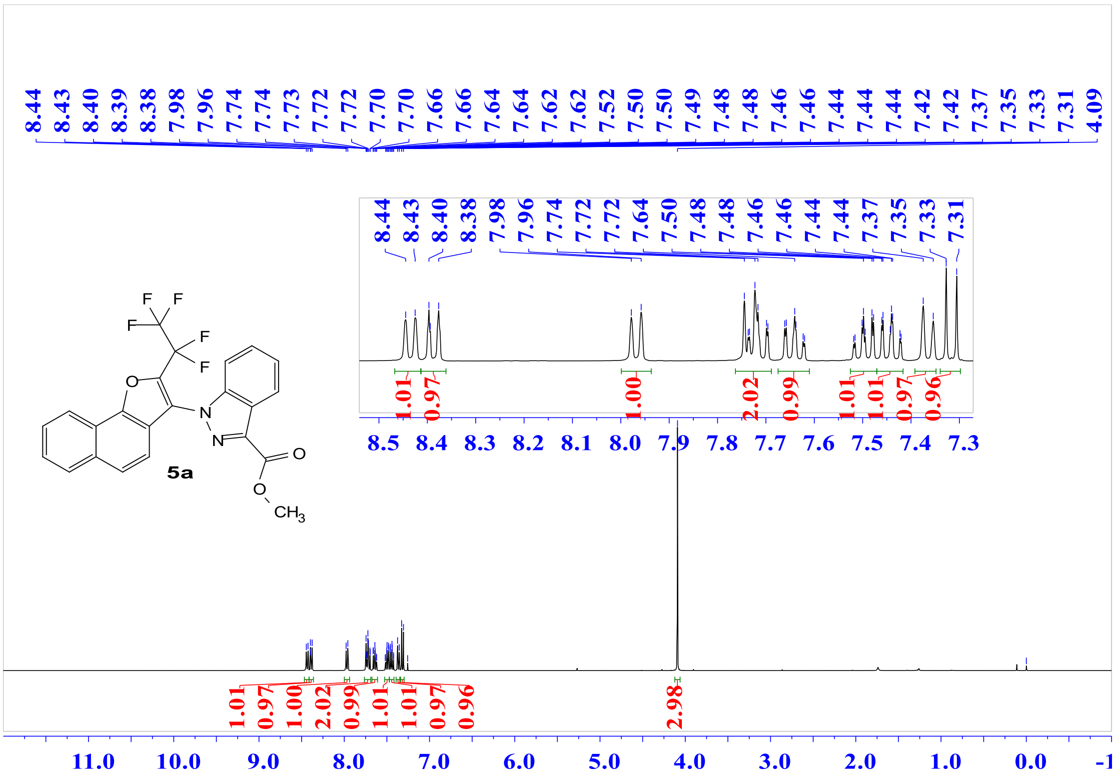

Figure S44. ^{19}F NMR spectrum of **5a**, related to **Scheme 1**.

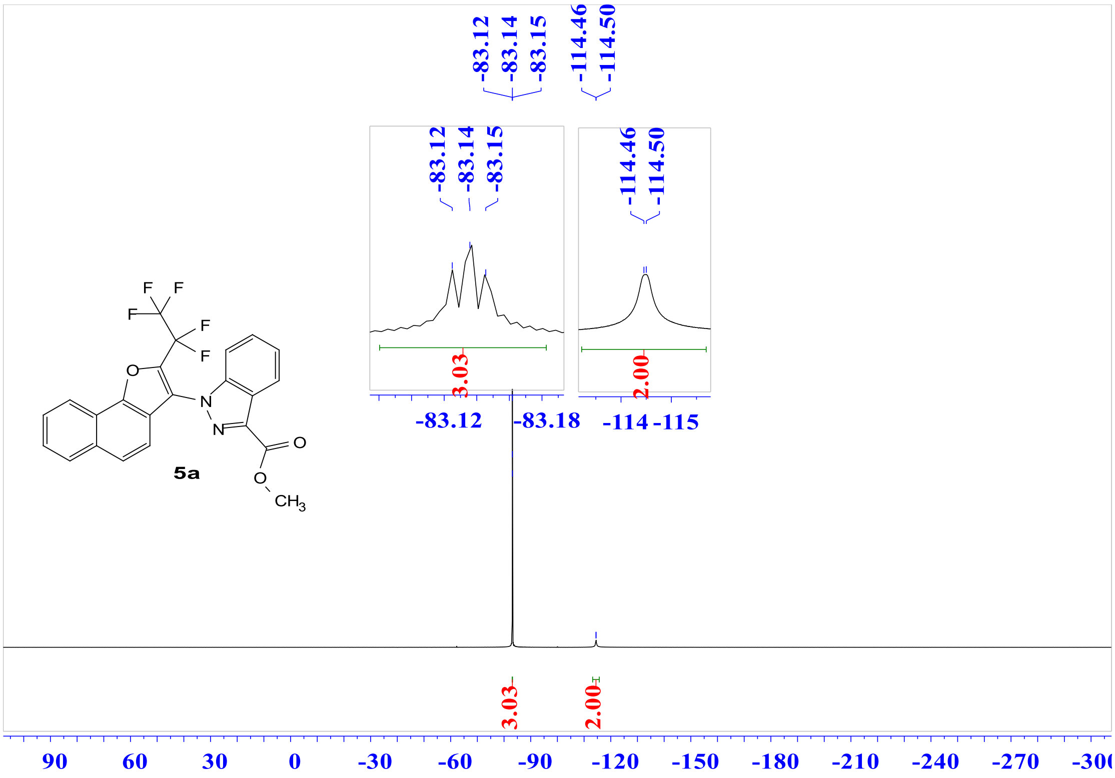

Figure S45. ^{13}C NMR spectrum of **5a**, related to **Scheme 1**.

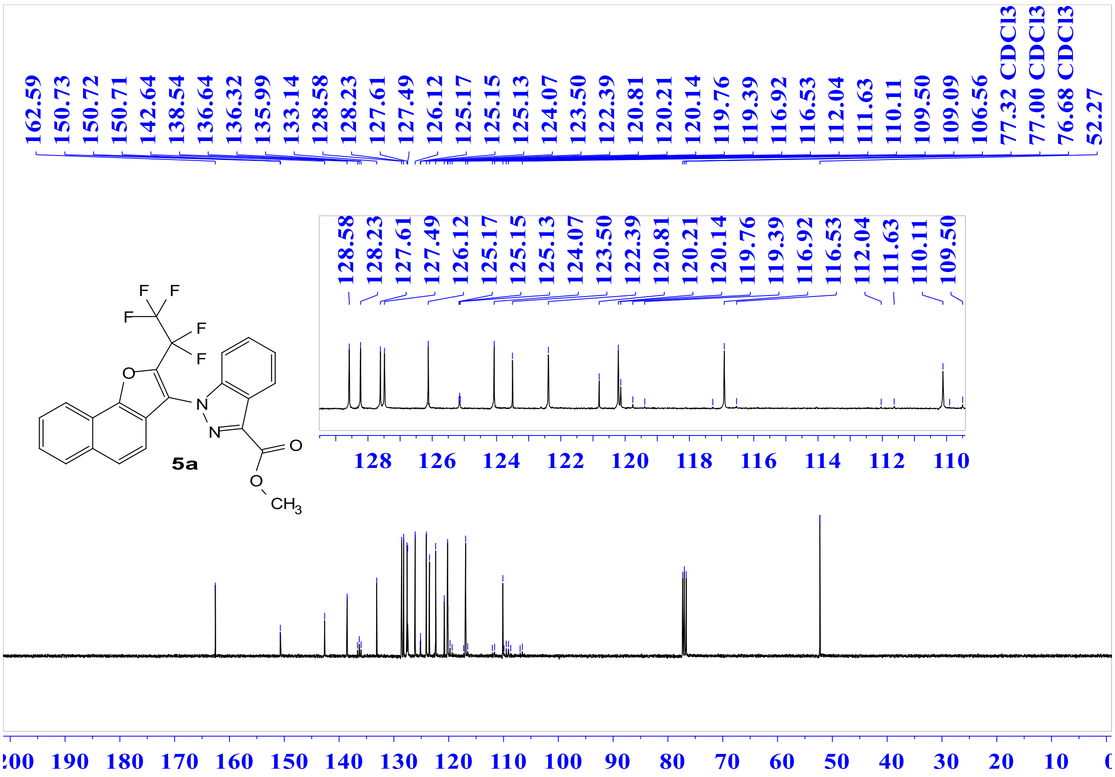

Figure S46. ^{1}H NMR spectrum of **5b**, related to **Scheme 1**.

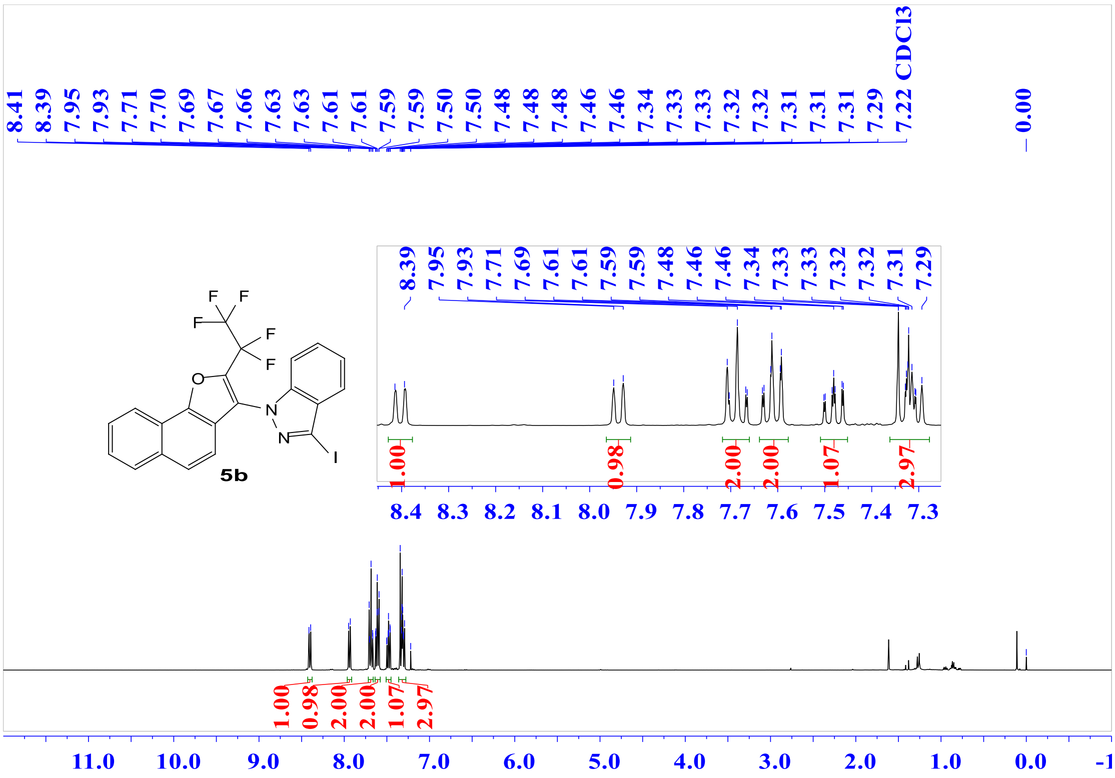

Figure S47. ^{19}F NMR spectrum of **5b**, related to **Scheme 1**.

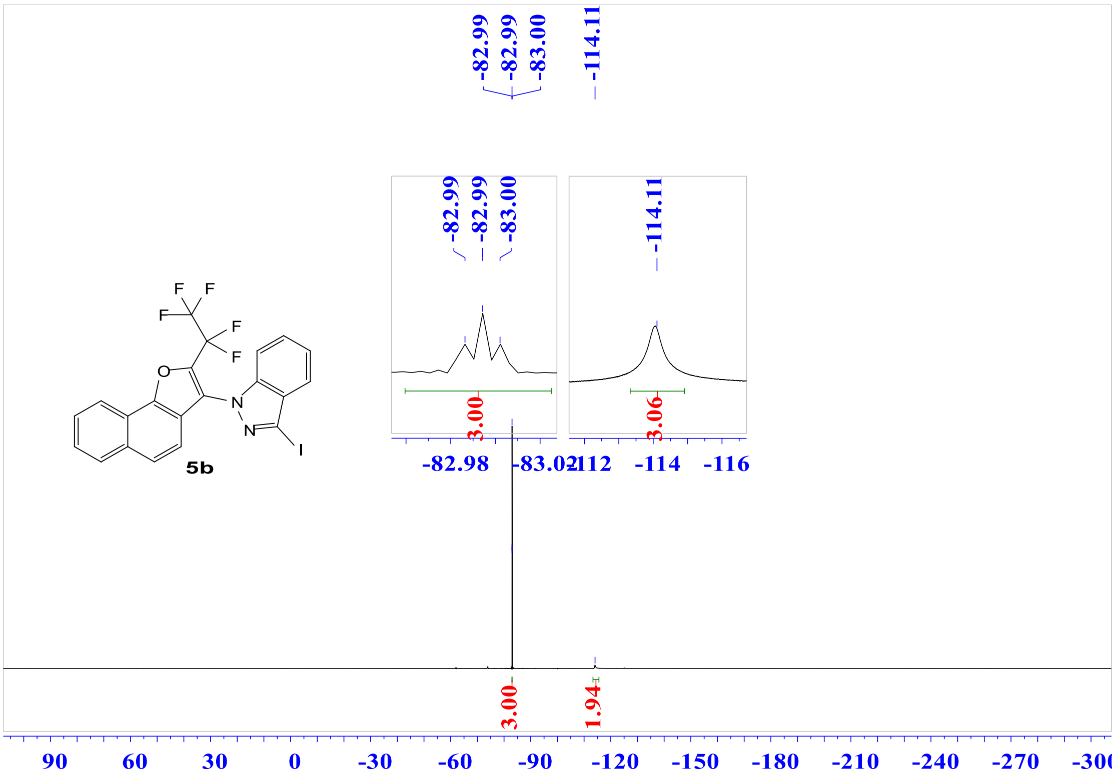

Figure S48. ^{13}C NMR spectrum of **5b**, related to **Scheme 1**.

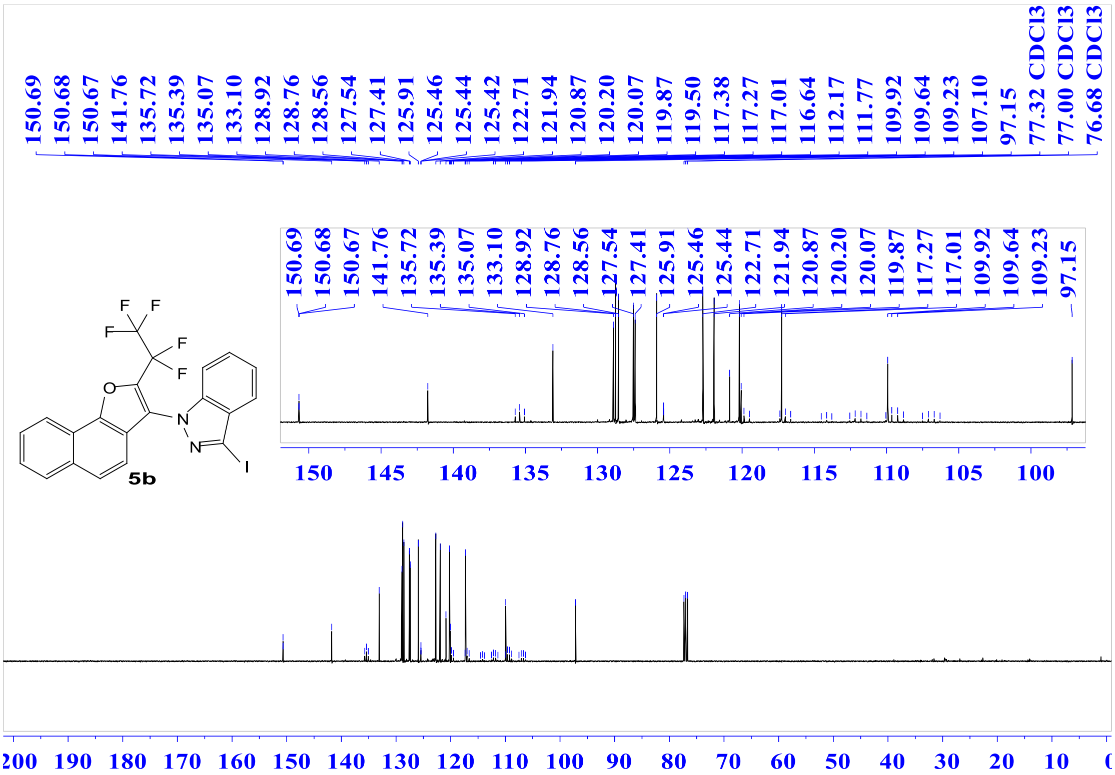

Figure S49. ^{1}H NMR spectrum of **5c-I**, related to **Scheme 1**.

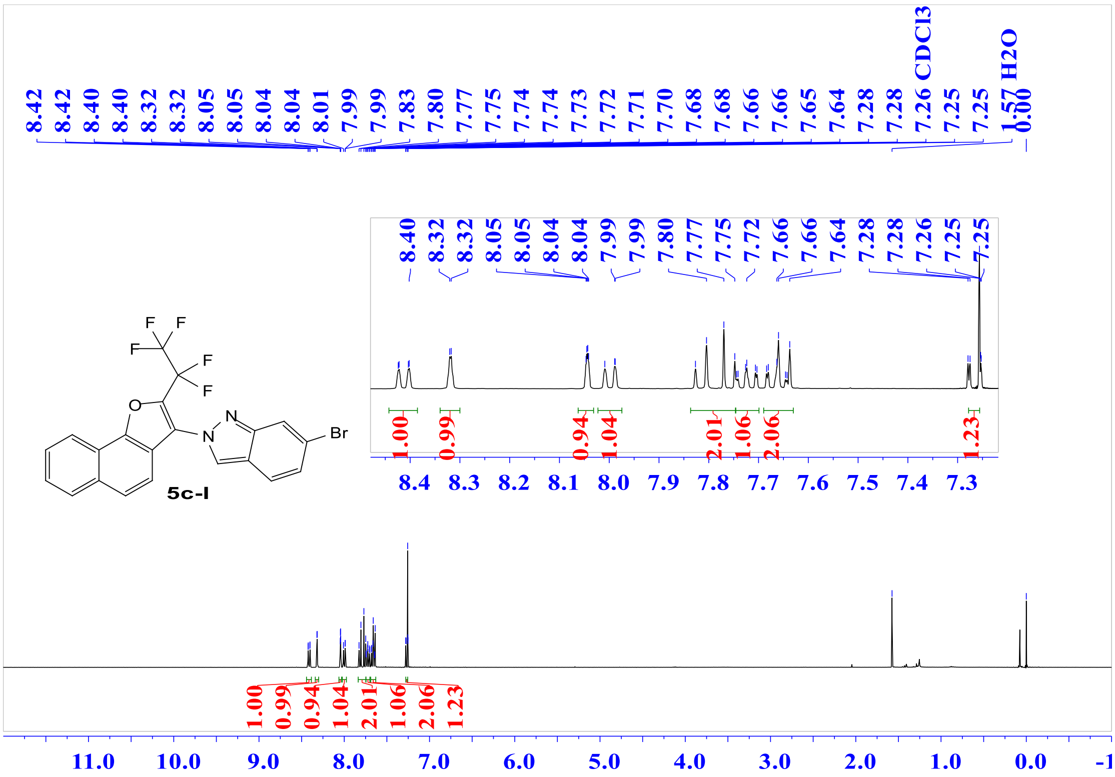

Figure S50. ^{19}F NMR spectrum of **5c-I**, related to **Scheme 1**.

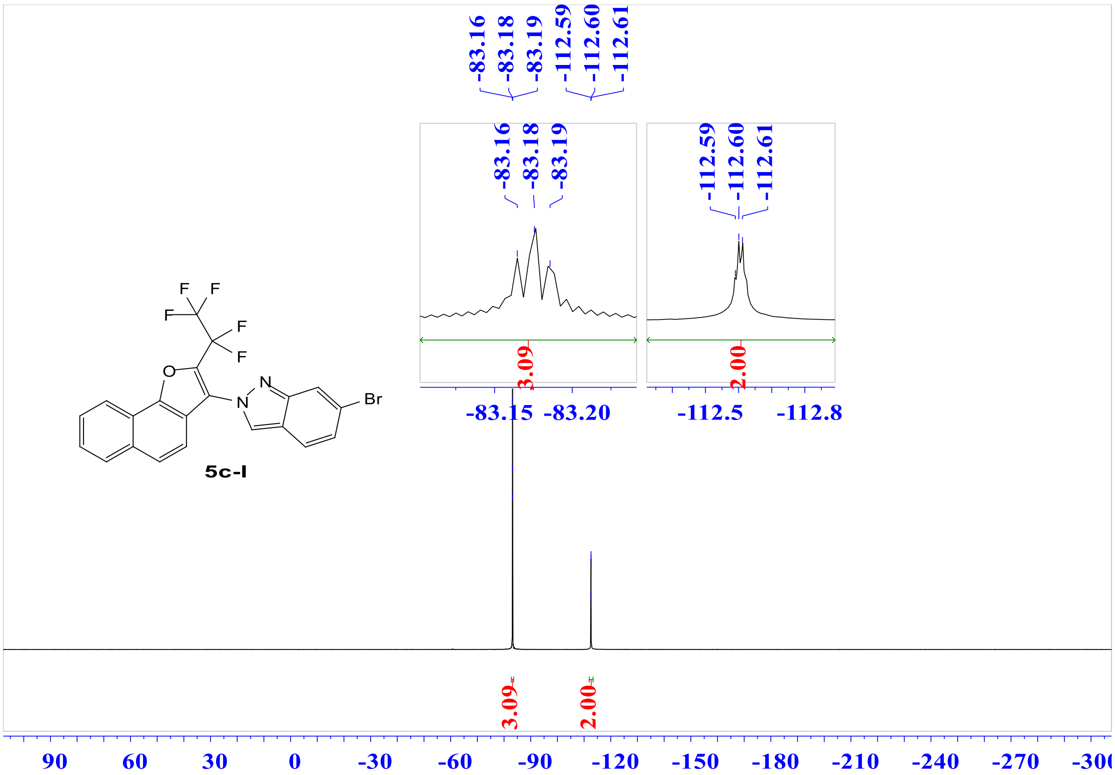

Figure S51. ^{13}C NMR spectrum of **5c-I**, related to **Scheme 1**.

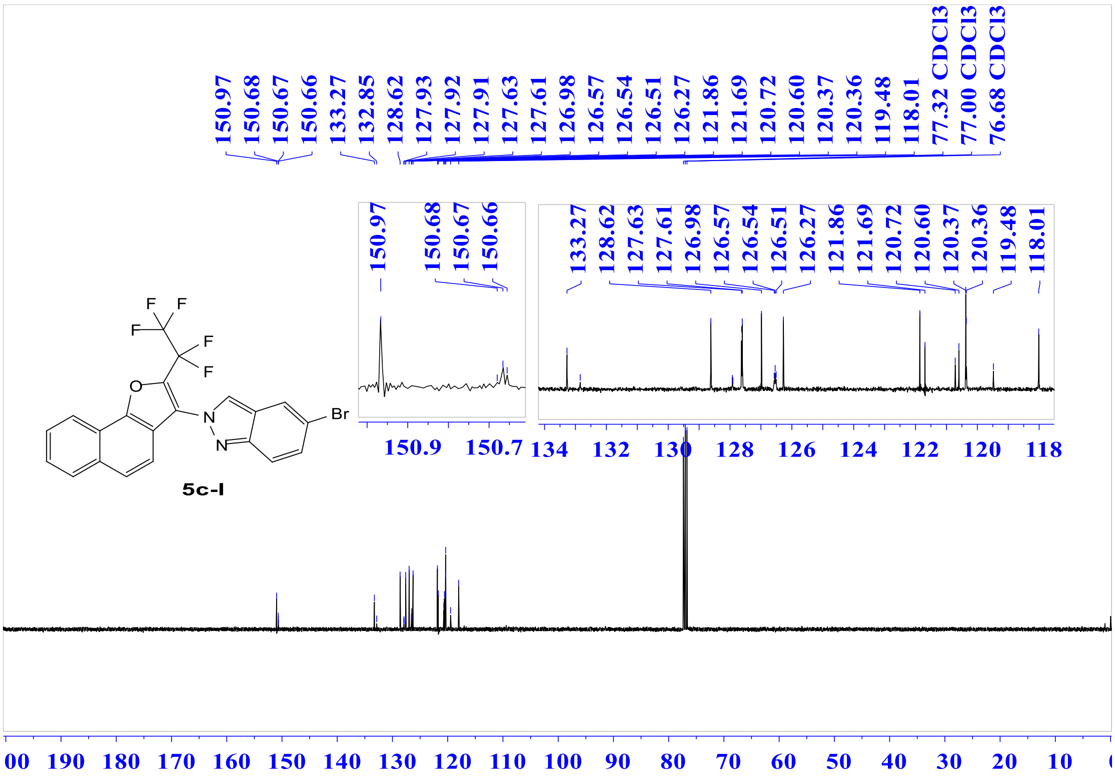

Figure S52. ^{1}H NMR spectrum of **5c-II**, related to **Scheme 1**.

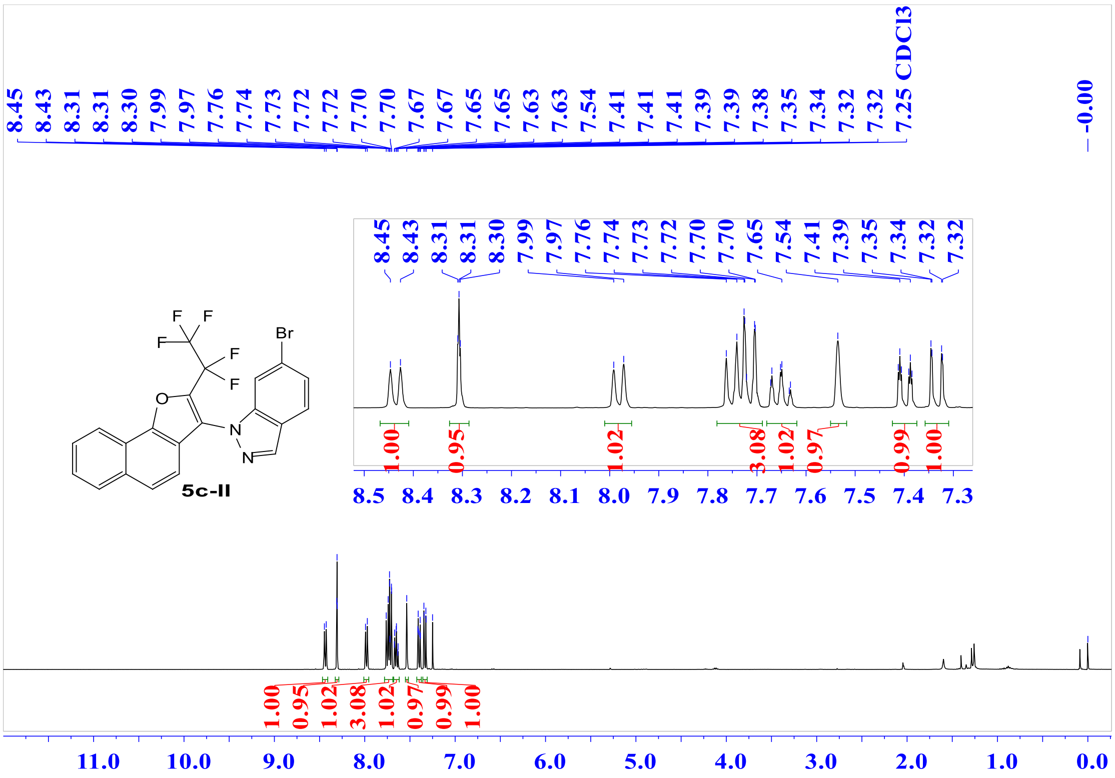

Figure S53. ^{19}F NMR spectrum of **5c-II**, related to **Scheme 1**.

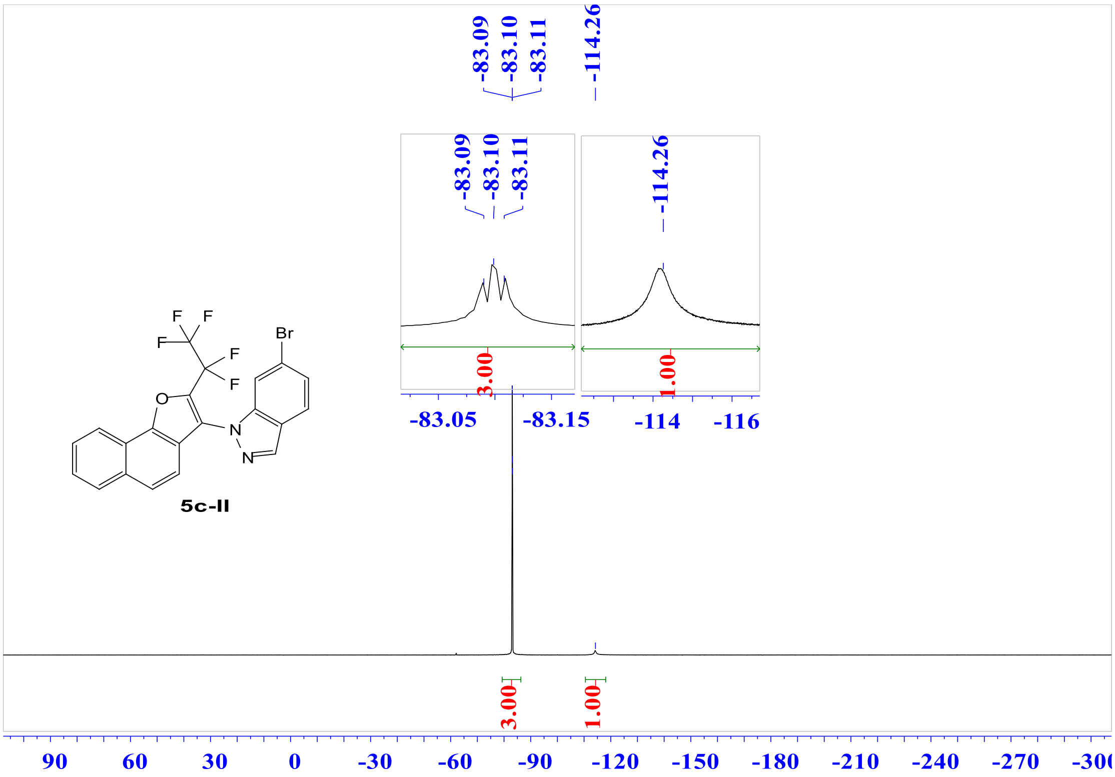

Figure S54. ^{13}C NMR spectrum of **5c-II**, related to **Scheme 1**.

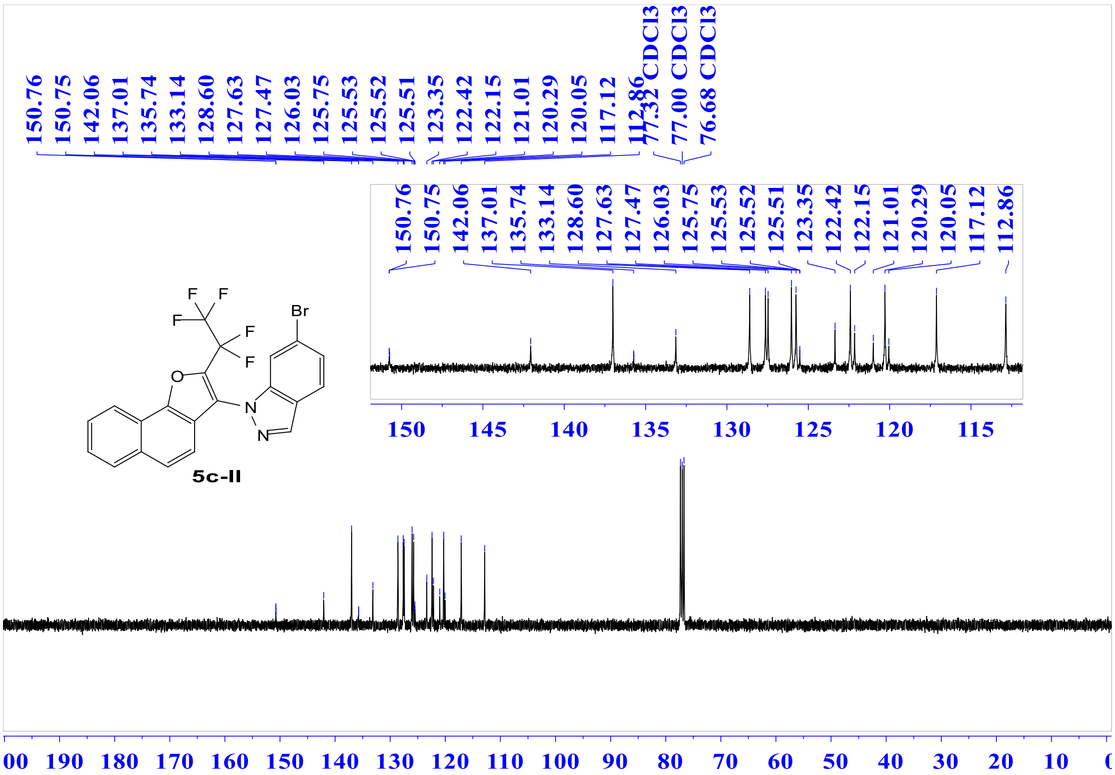

Figure S55. ^{1}H NMR spectrum of **6a**, related to **Scheme 1**.

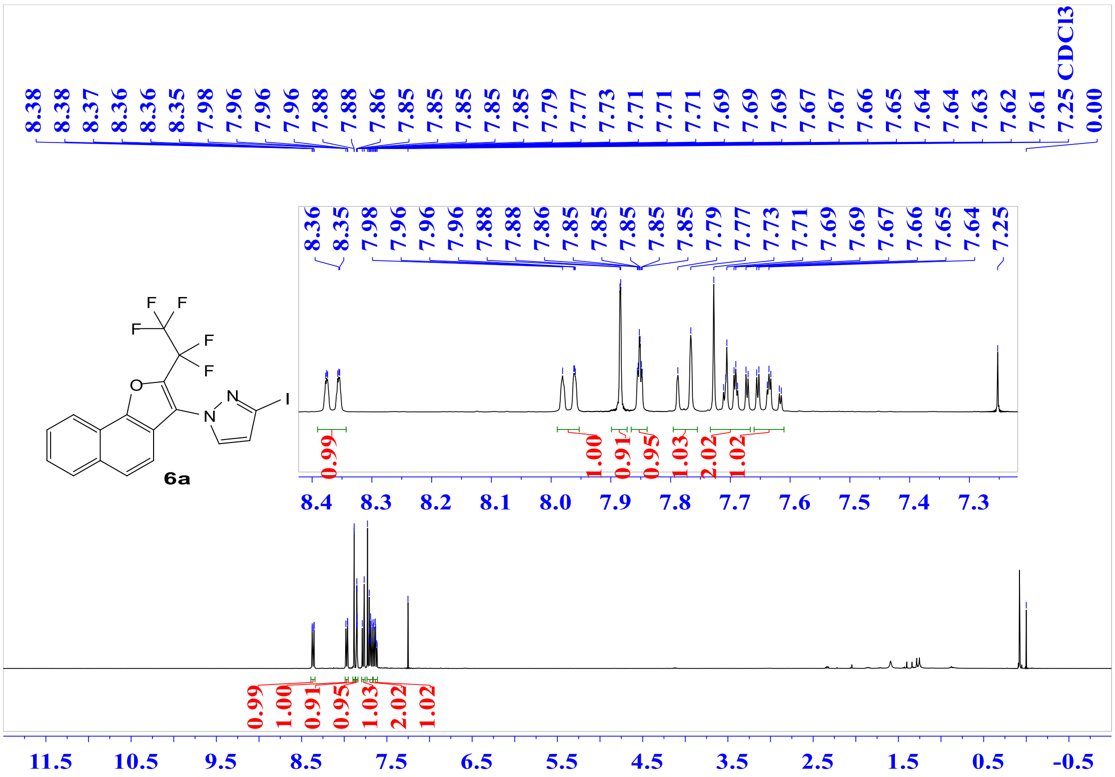

Figure S56. ^{19}F NMR spectrum of **6a**, related to **Scheme 1**.

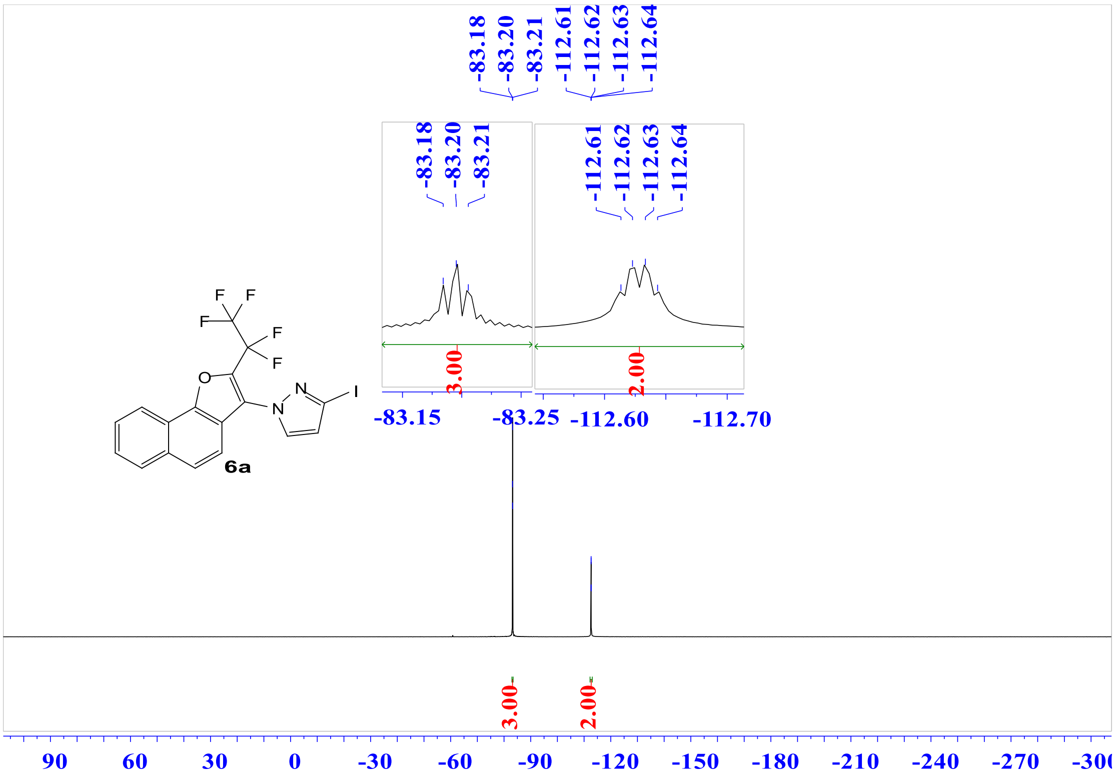

Figure S57. ^{13}C NMR spectrum of **6a**, related to **Scheme 1**.

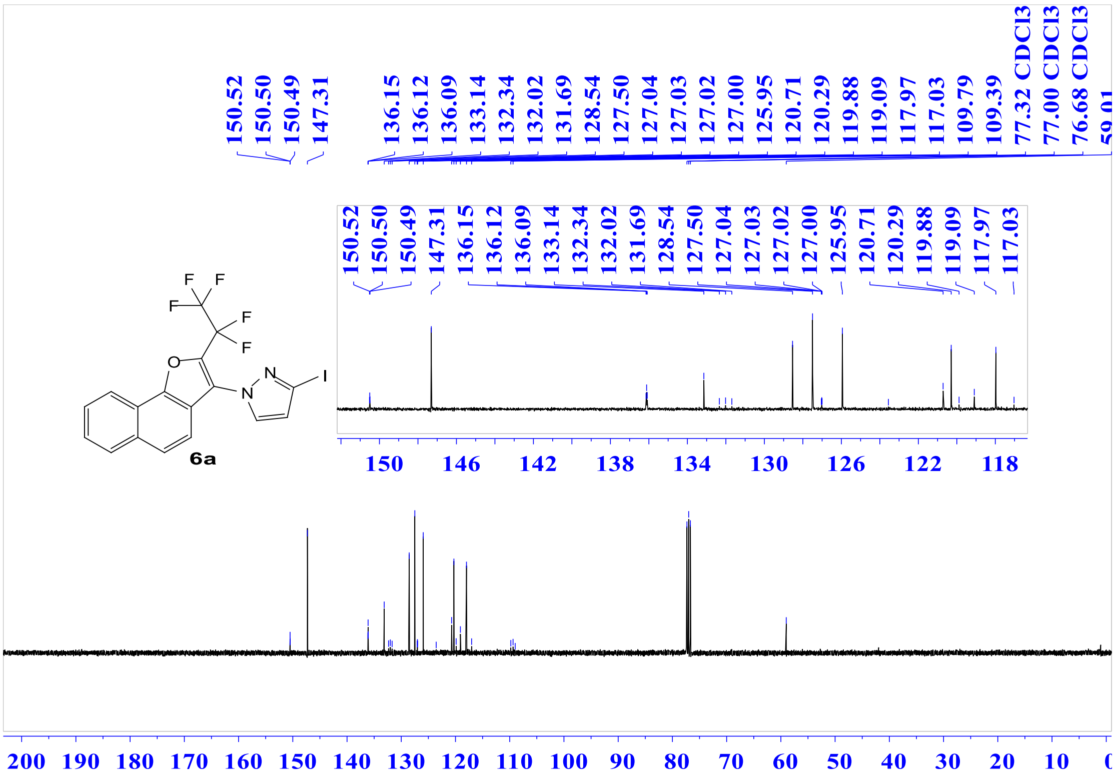

Figure S58. ^{1}H NMR spectrum of **6b**, related to **Scheme 1**.

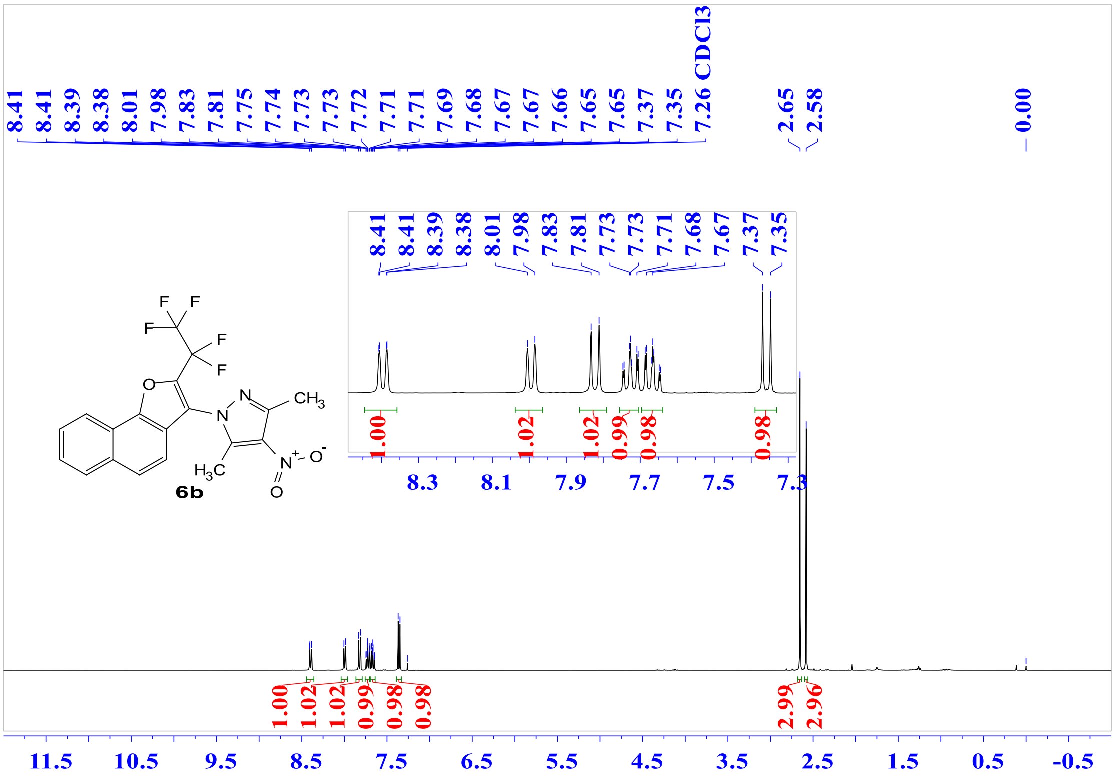

Figure S59. ^{19}F NMR spectrum of **6b**, related to **Scheme 1**.

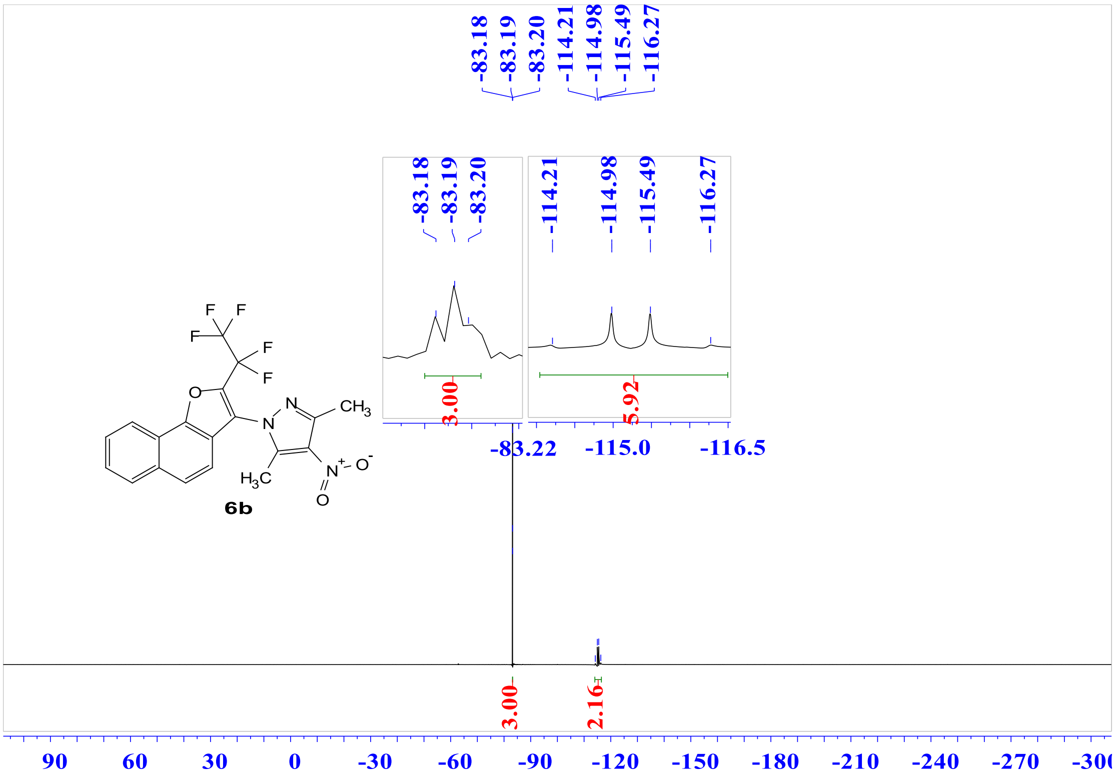

Figure S60. ^{13}C NMR spectrum of **6b**, related to **Scheme 1**.

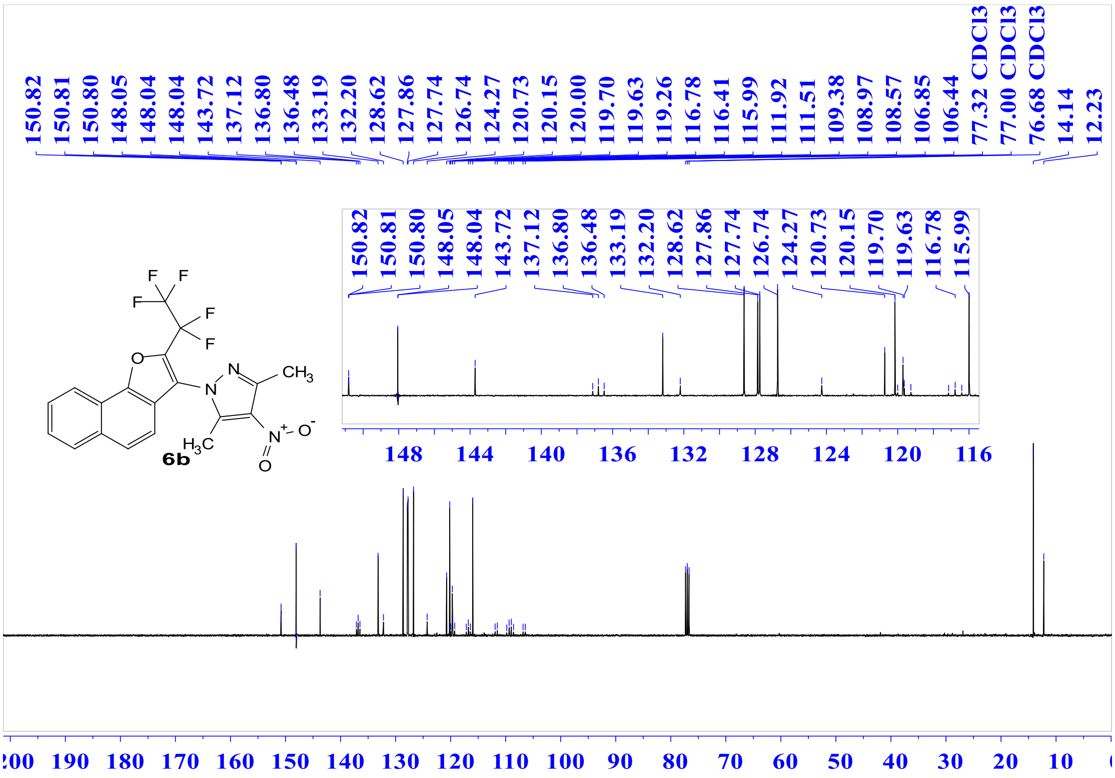

Figure S61. ^{1}H NMR spectrum of **6c**, related to **Scheme 1**.

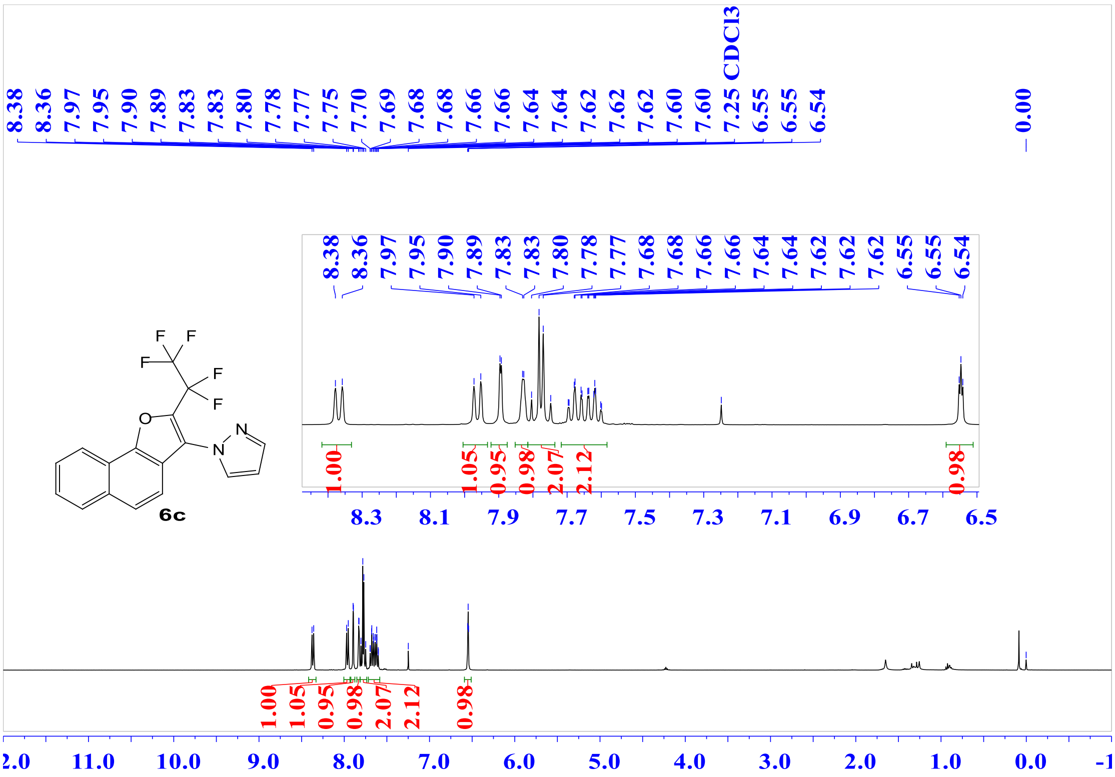

Figure S62. ^{19}F NMR spectrum of **6c**, related to **Scheme 1**.

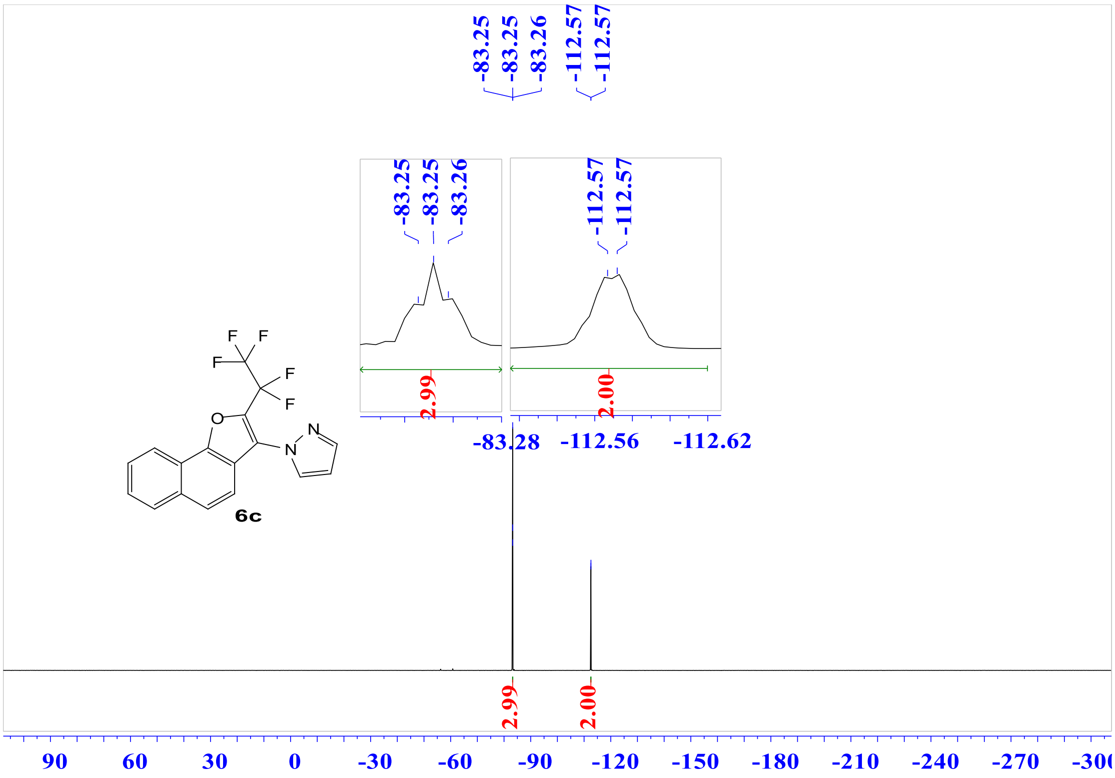

Figure S63. ^{13}C NMR spectrum of **6c**, related to **Scheme 1**.

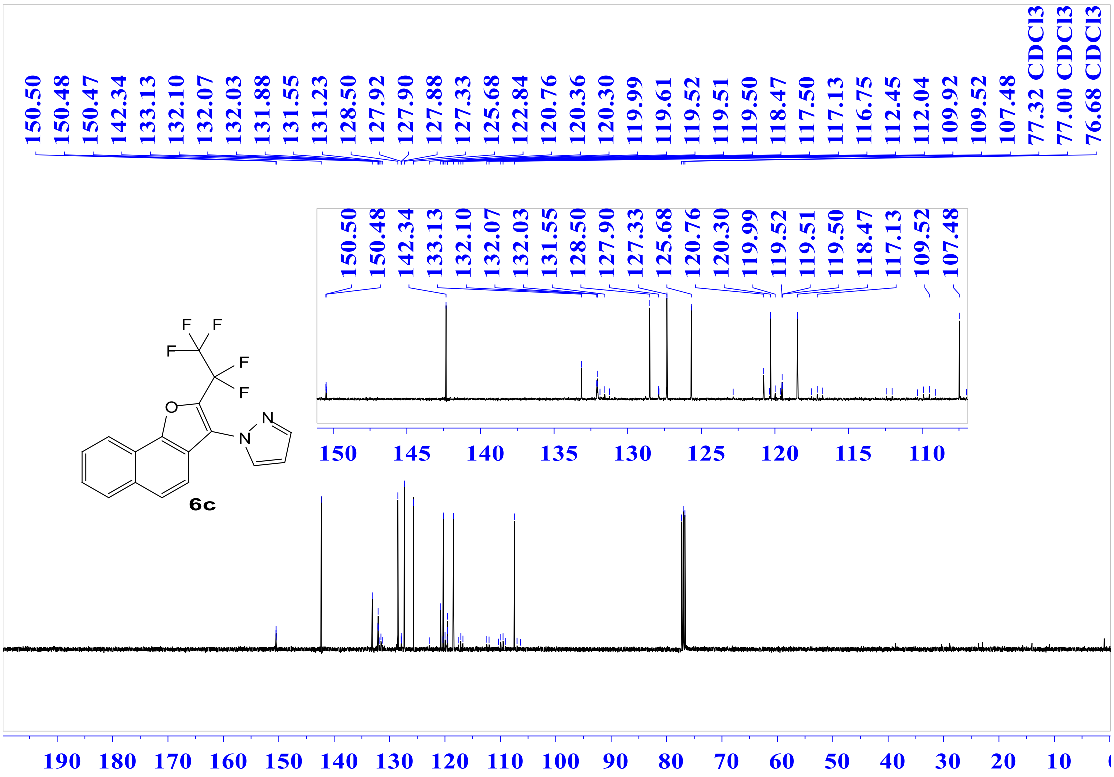

Figure S64. ^{1}H NMR spectrum of **6d**, related to **Scheme 1**.

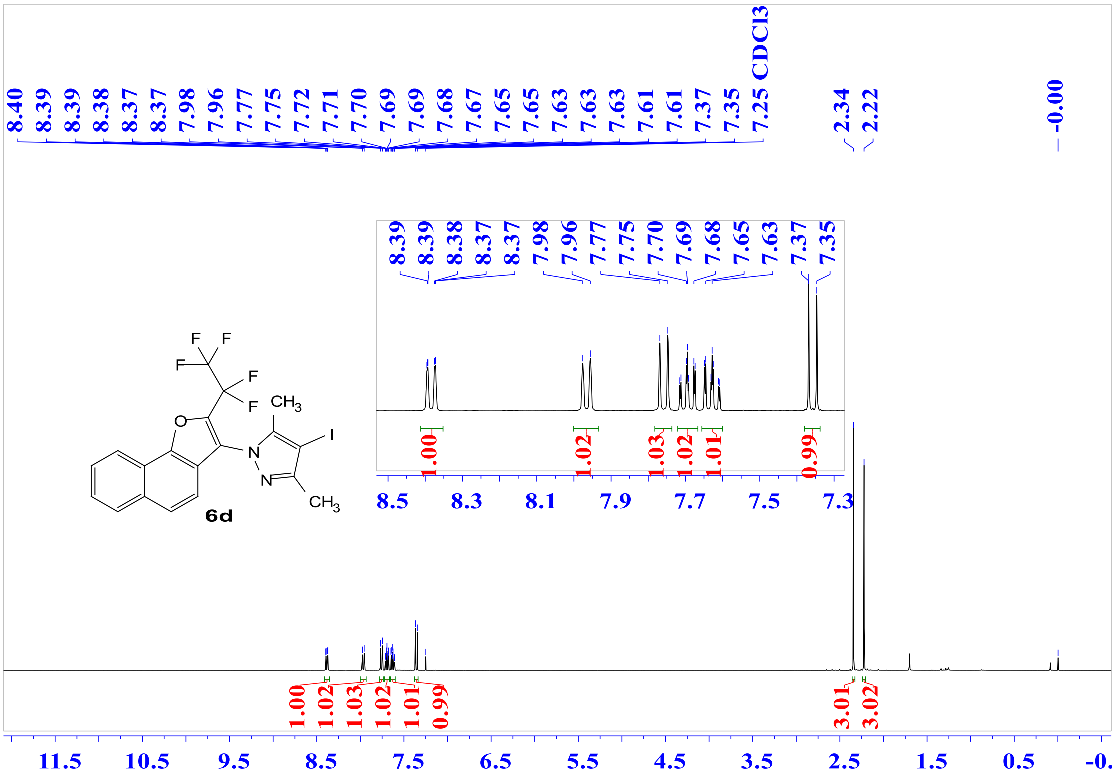

Figure S65. ^{19}F NMR spectrum of **6d**, related to **Scheme 1**.

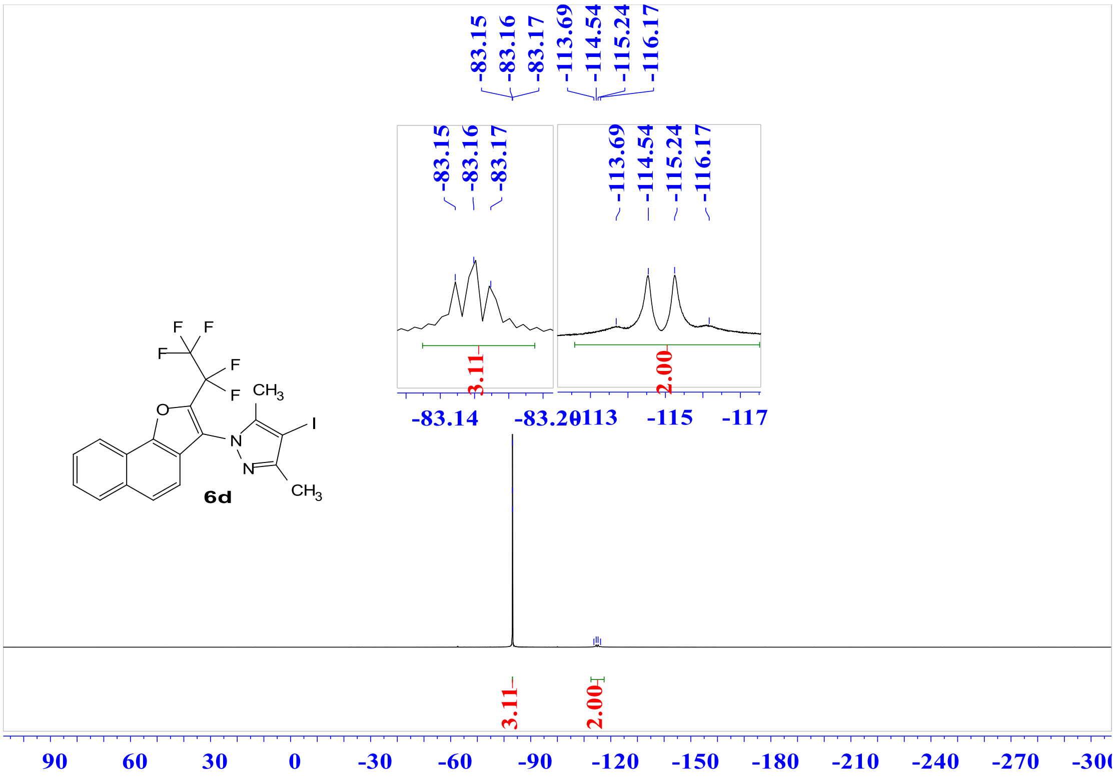

Figure S66. ^{13}C NMR spectrum of **6d**, related to **Scheme 1**.

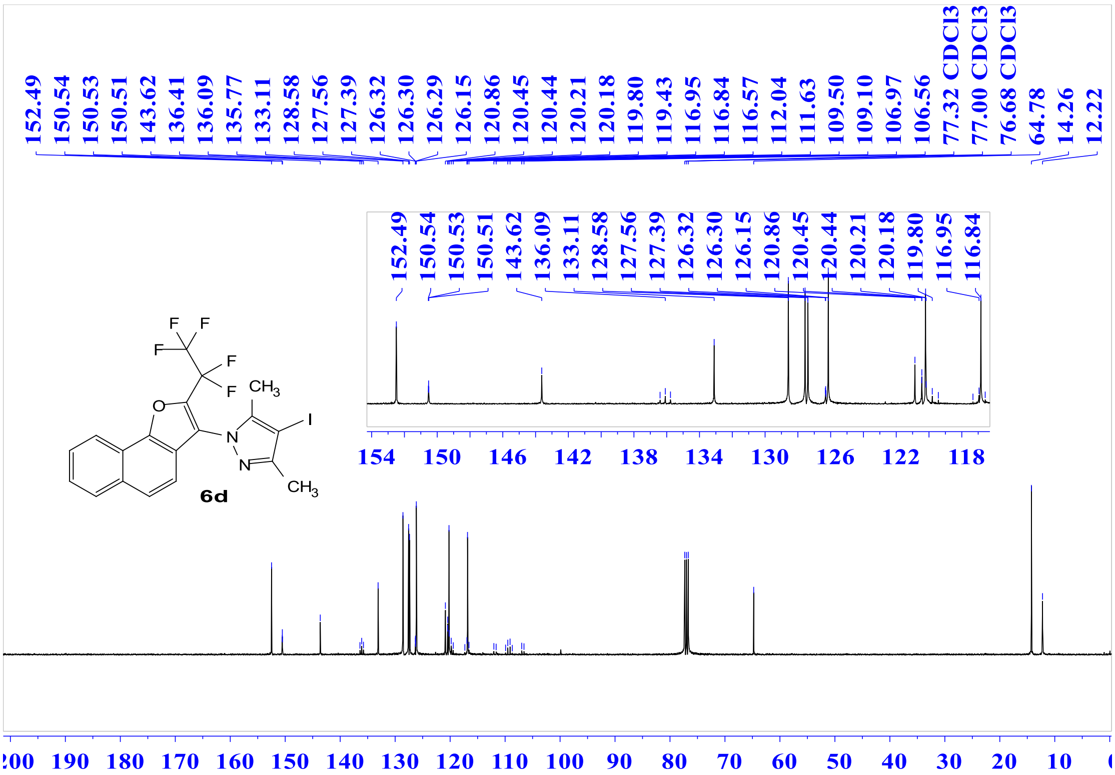

Figure S67. ^{1}H NMR spectrum of **7a**, related to **Scheme 1**.

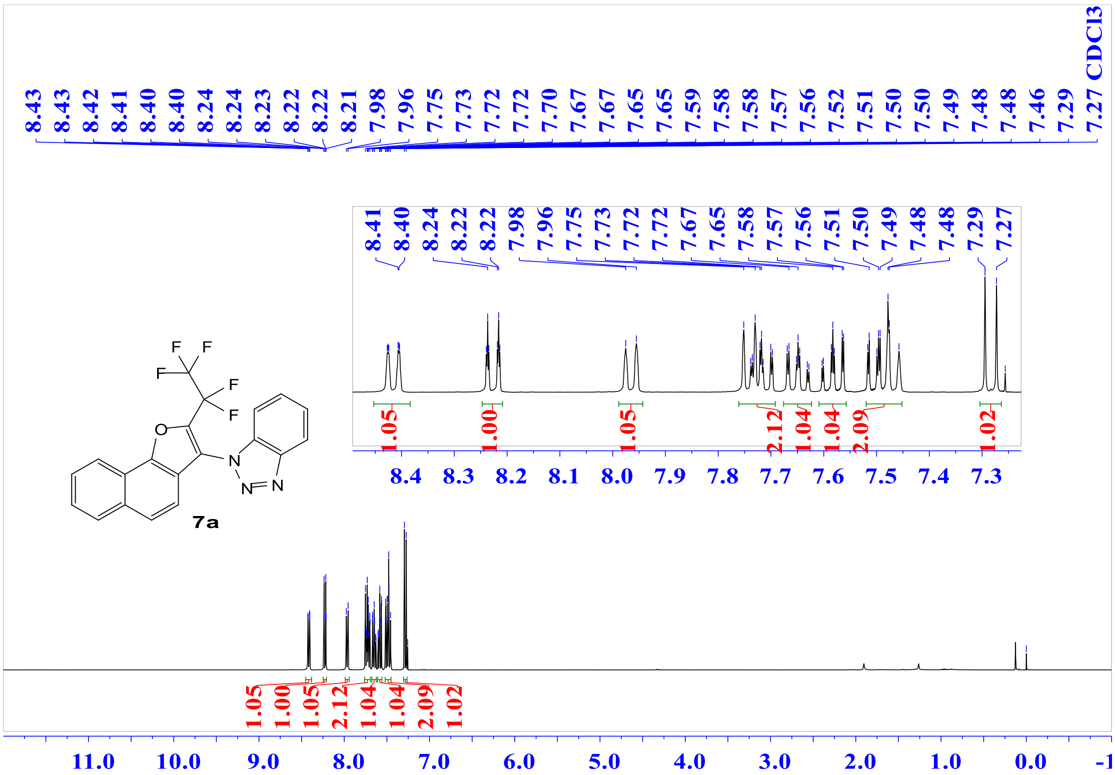

Figure S68. ^{19}F NMR spectrum of **7a**, related to **Scheme 1**.

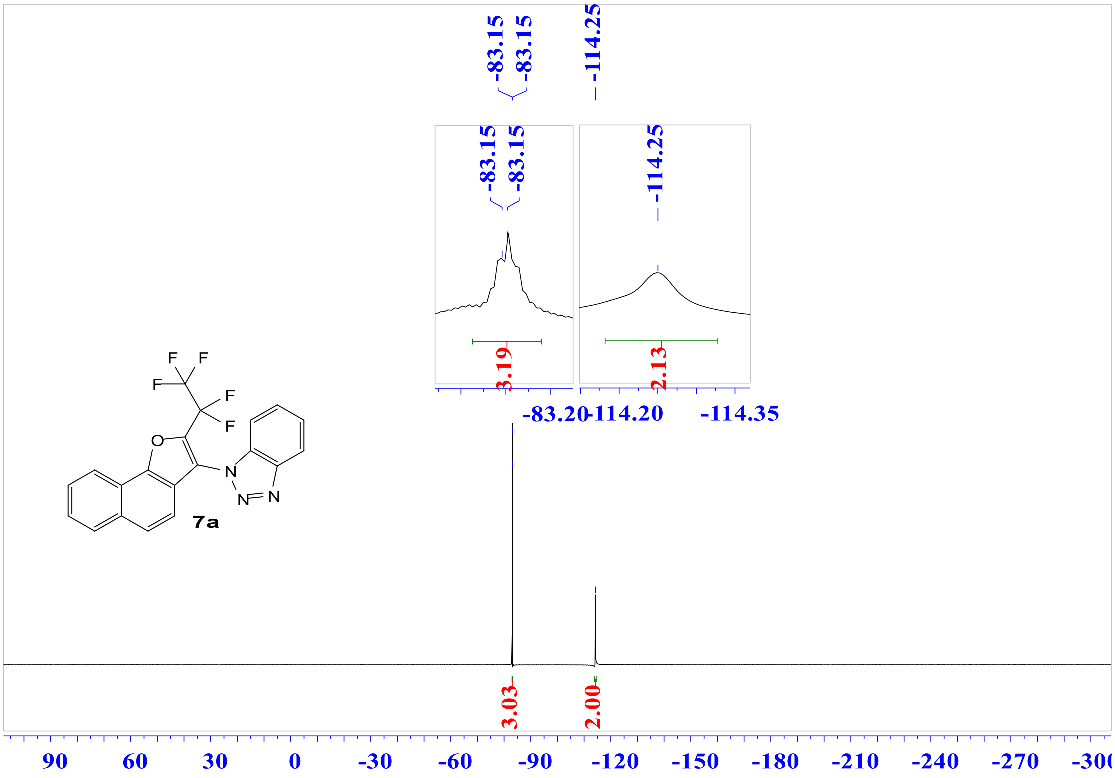

Figure S69. ^{13}C NMR spectrum of **7a**, related to **Scheme 1**.

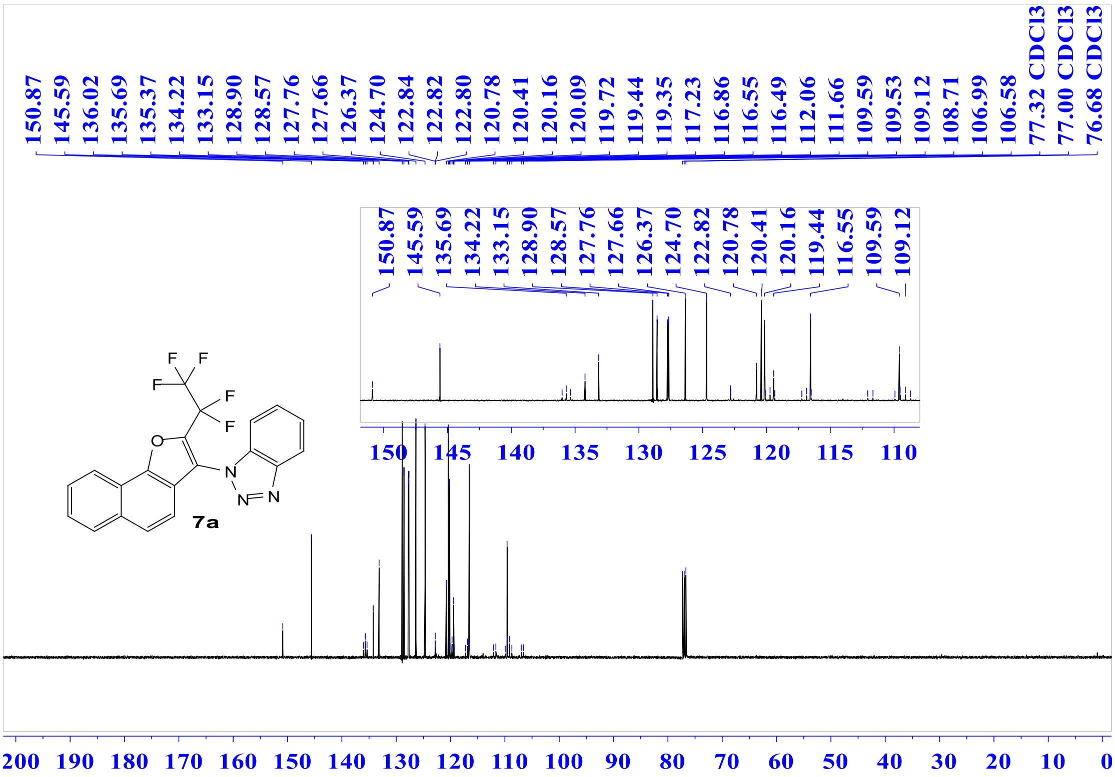

Figure S70. ^{1}H NMR spectrum of **7b-I** or **7b-II**, related to **Scheme 1**.

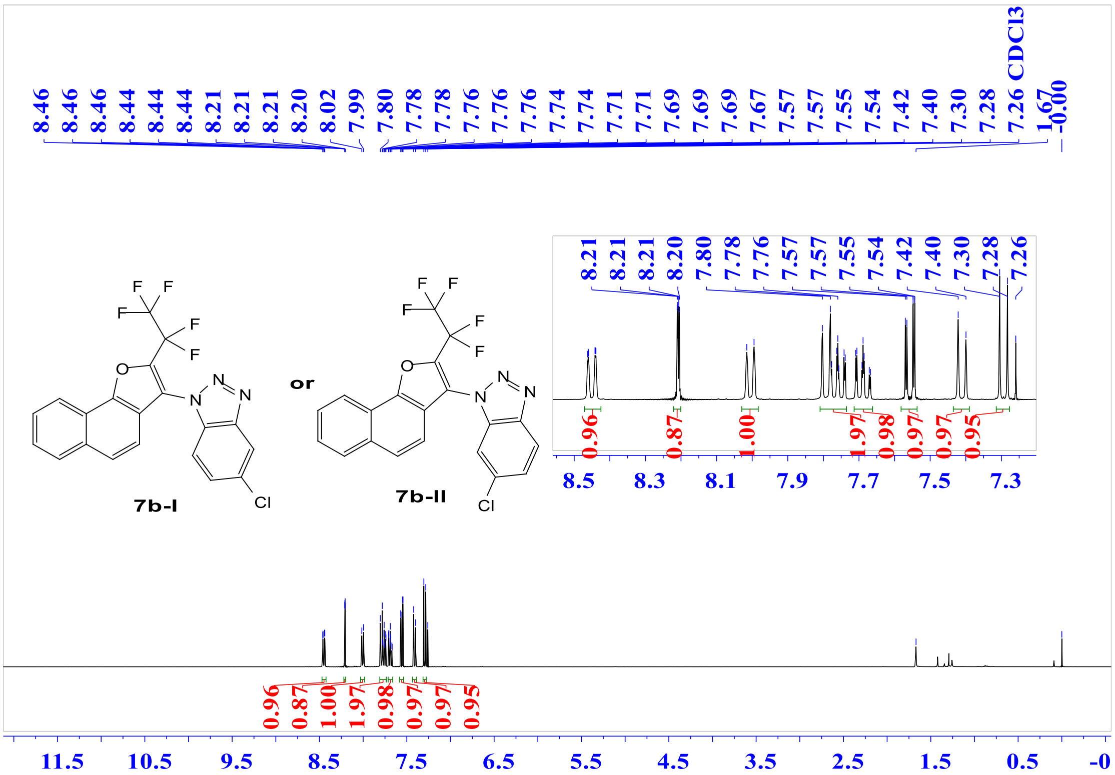

Figure S71. ^{19}F NMR spectrum of **7b-I** or **7b-II**, related to **Scheme 1**.

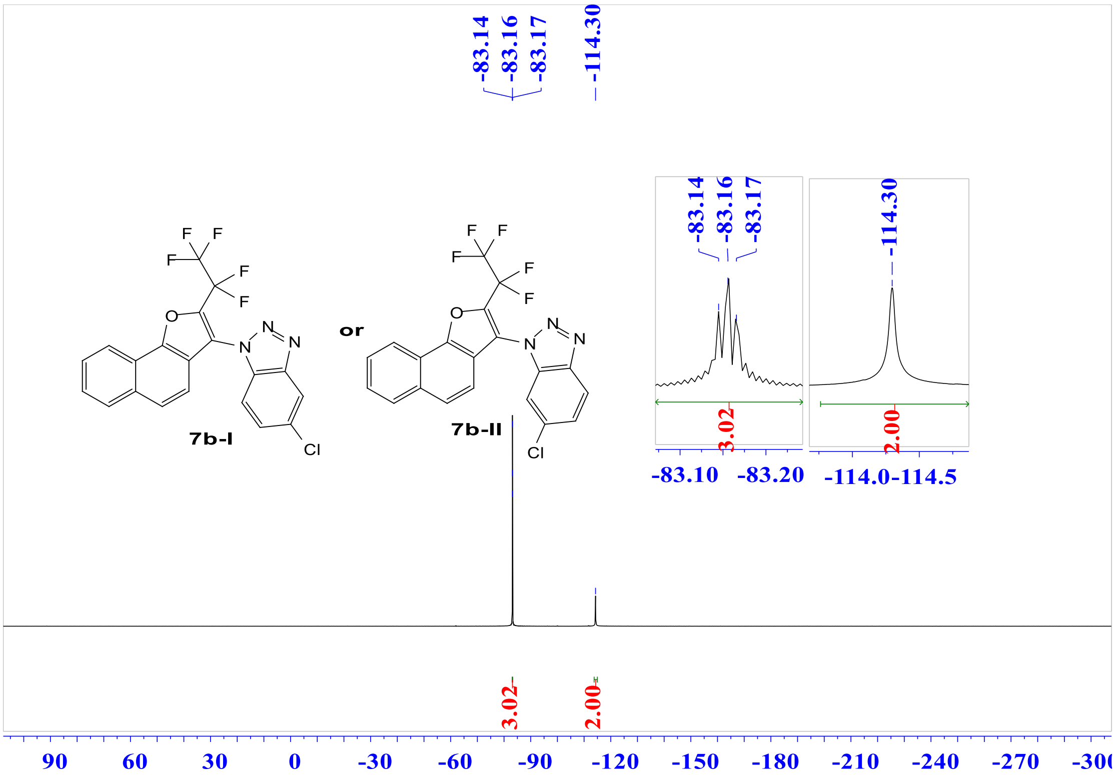

Figure S72. ^{13}C NMR spectrum of **7b-I** or **7b-II**, related to **Scheme 1**.

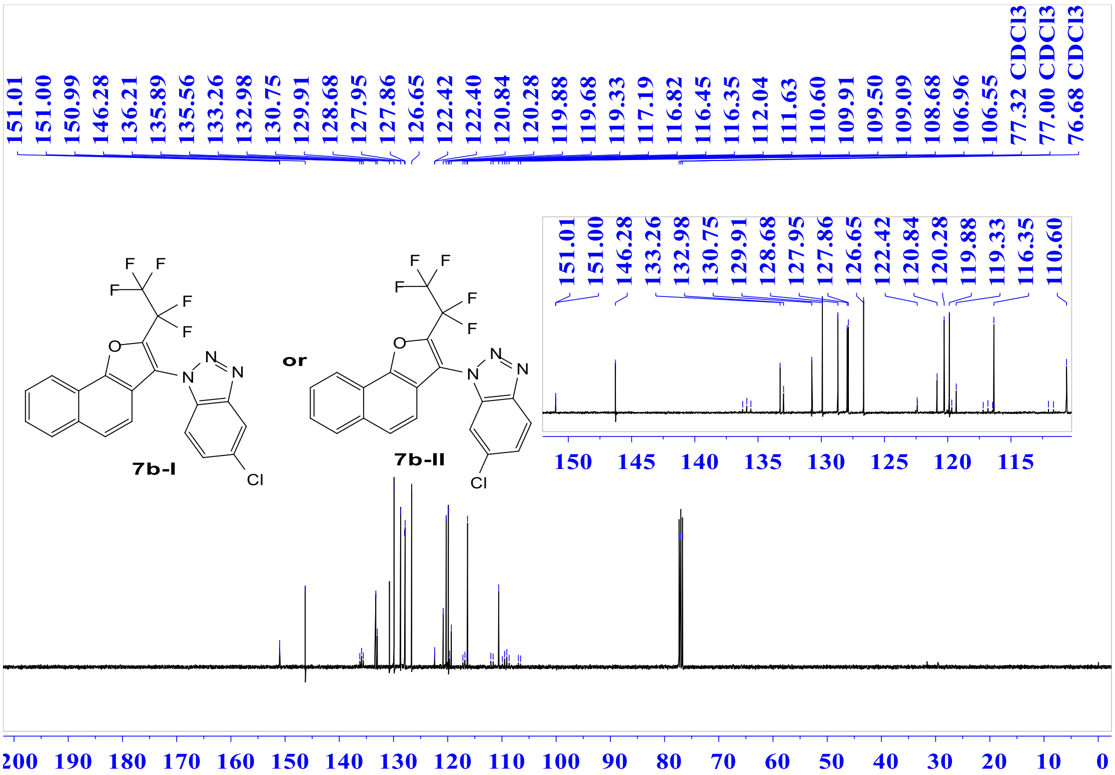

Figure S73. ^{1}H NMR spectrum of **7b-I** or **7b-II**, related to **Scheme 1**.

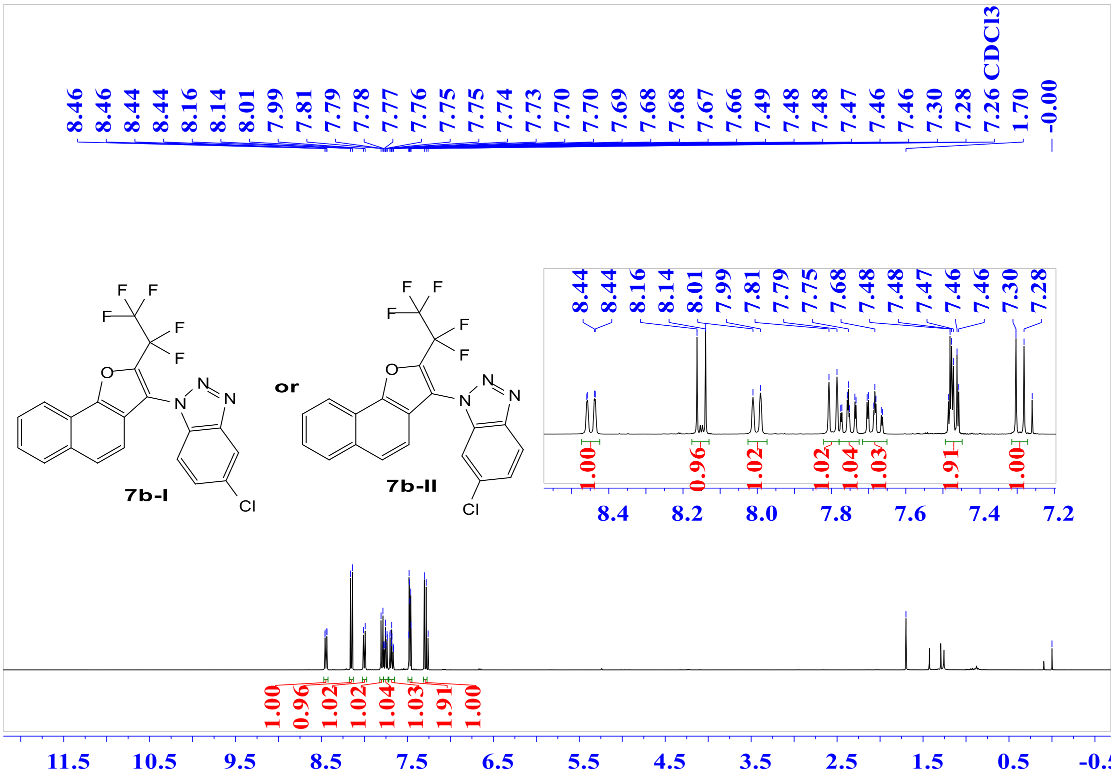

Figure S74. ^{19}F NMR spectrum of **7b-I** or **7b-II**, related to **Scheme 1**.

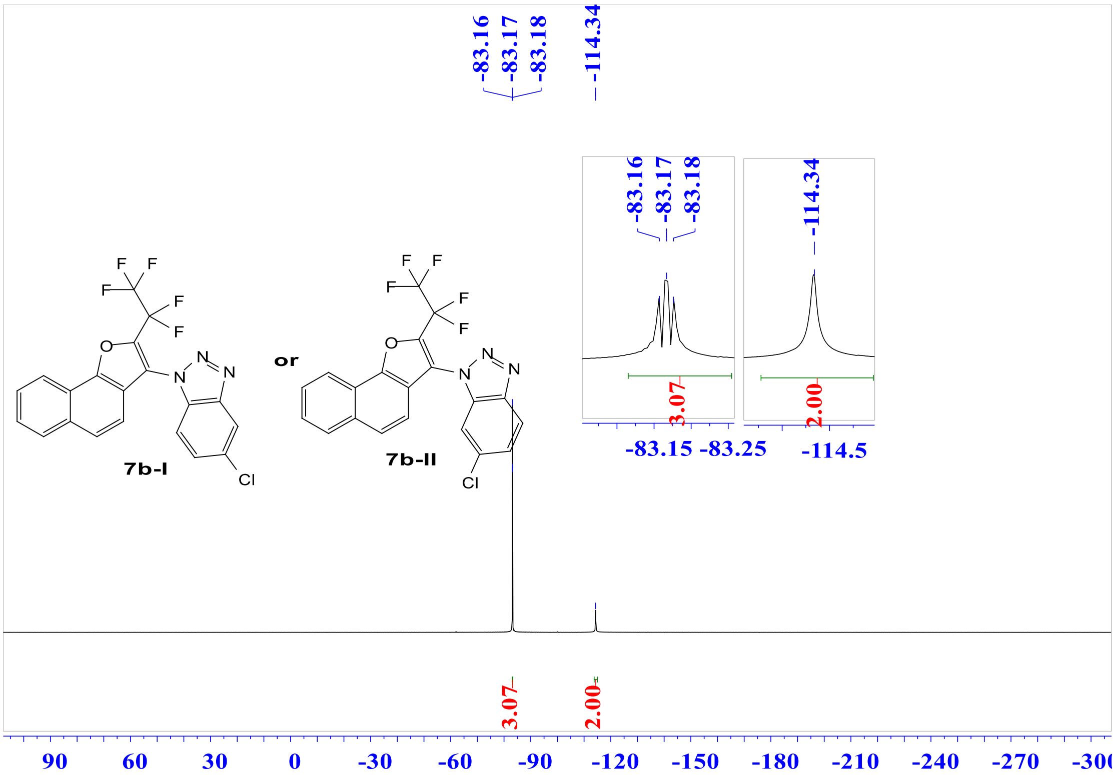

Figure S75. ^{13}C NMR spectrum of **7b-I** or **7b-II**, related to **Scheme 1**.

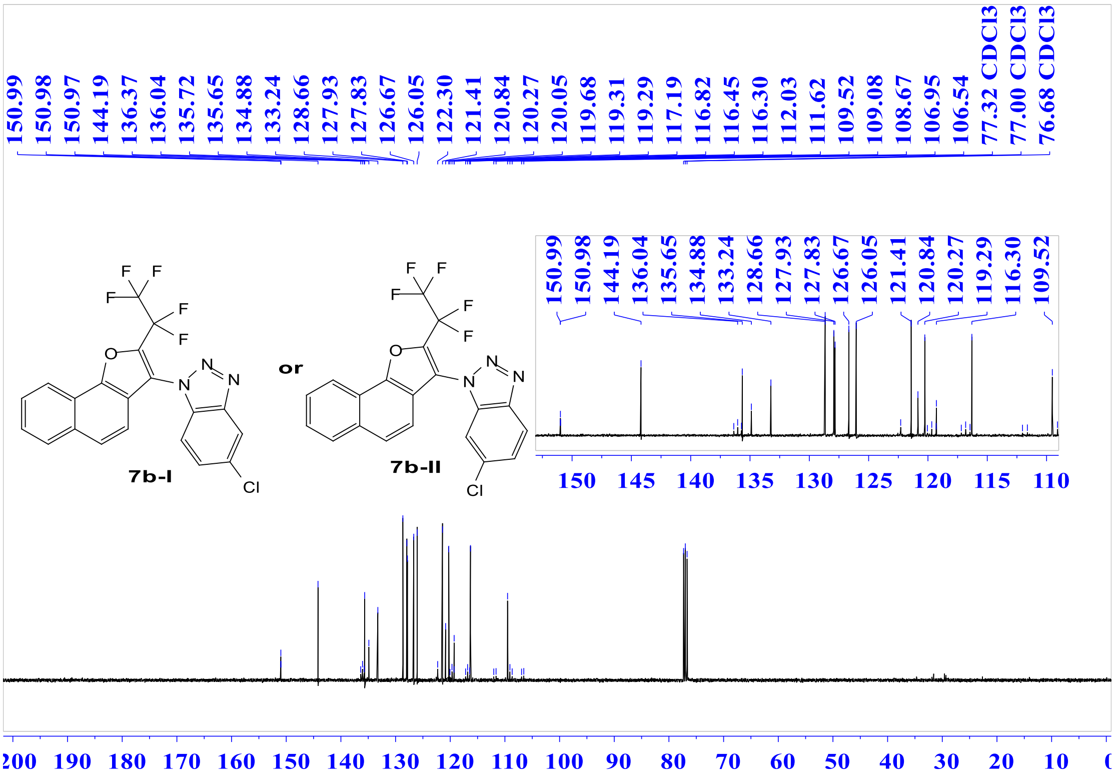

Figure S76. ^{1}H NMR spectrum of **7c-I**, **7c-II**, or **7c-III**, related to **Scheme 1**.

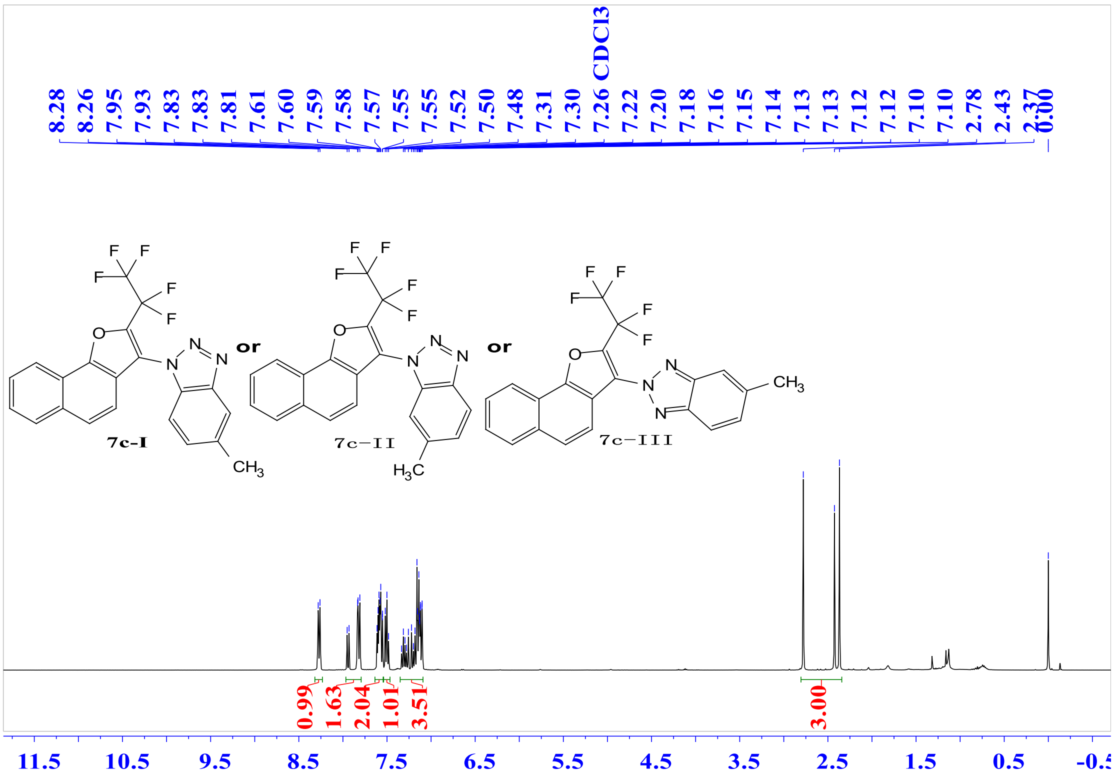

Figure S77. ^{19}F NMR spectrum of **7c-I**, **7c-II**, or **7c-III**, related to **Scheme 1**.

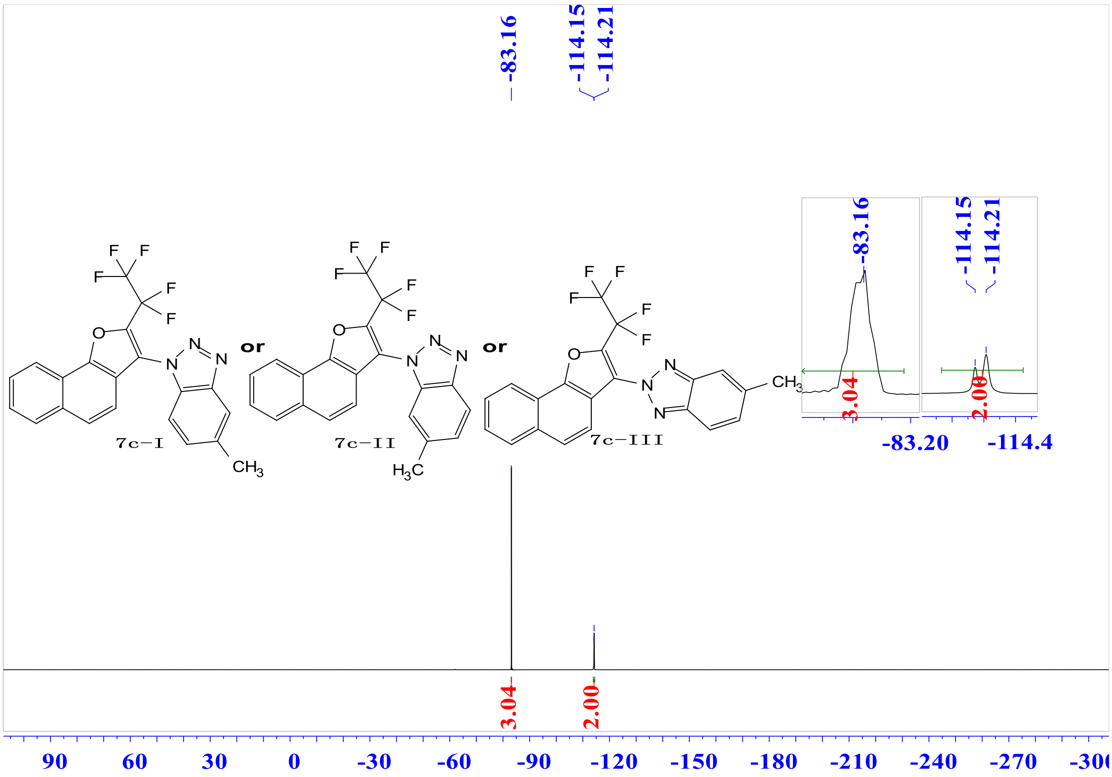

Figure S78. ^{13}C NMR spectrum of **7c-I**, **7c-II**, or **7c-III**, related to **Scheme 1**.

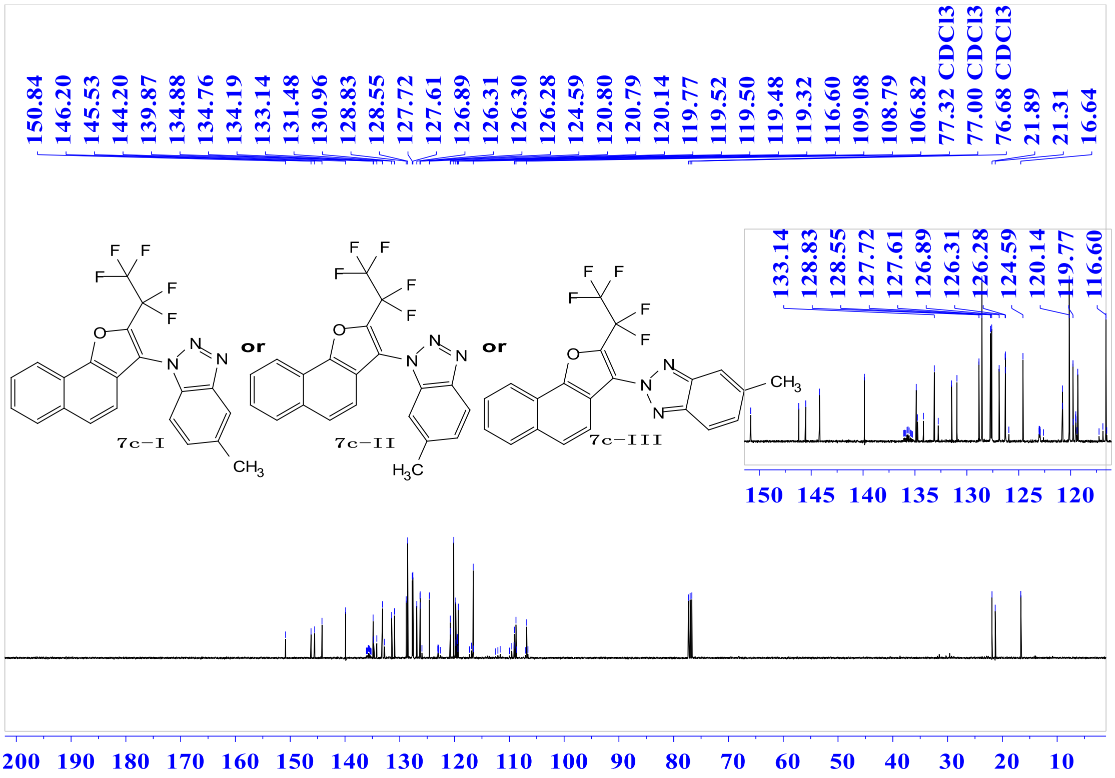

Figure S79. ^{1}H NMR spectrum of **8-l**, related to **Scheme 1**.

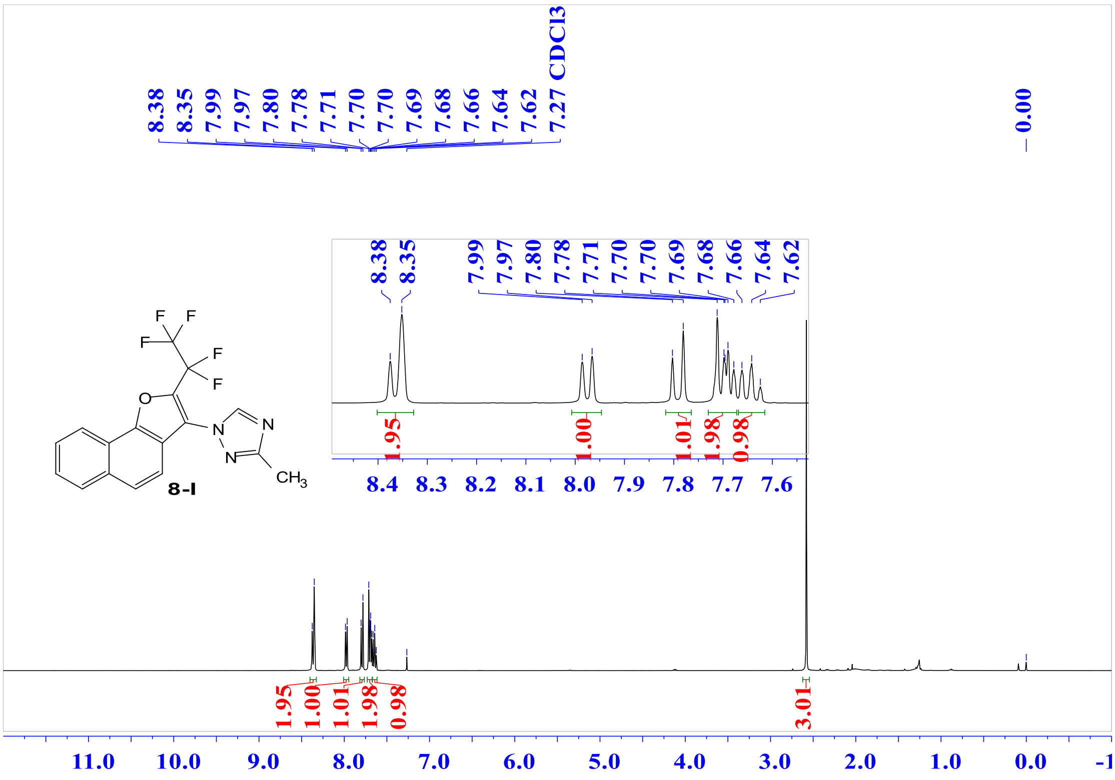

Figure S80. ^{19}F NMR spectrum of **8-l**, related to **Scheme 1**.

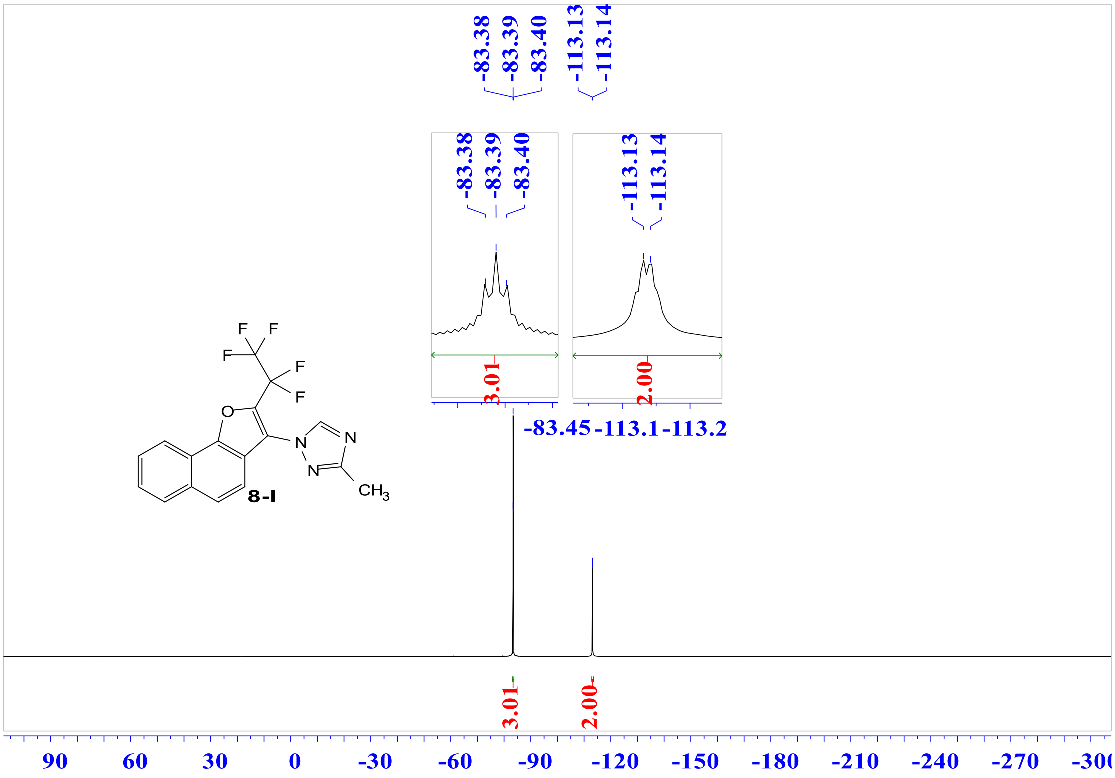

Figure S81. ^{13}C NMR spectrum of **8-I**, related to **Scheme 1**.

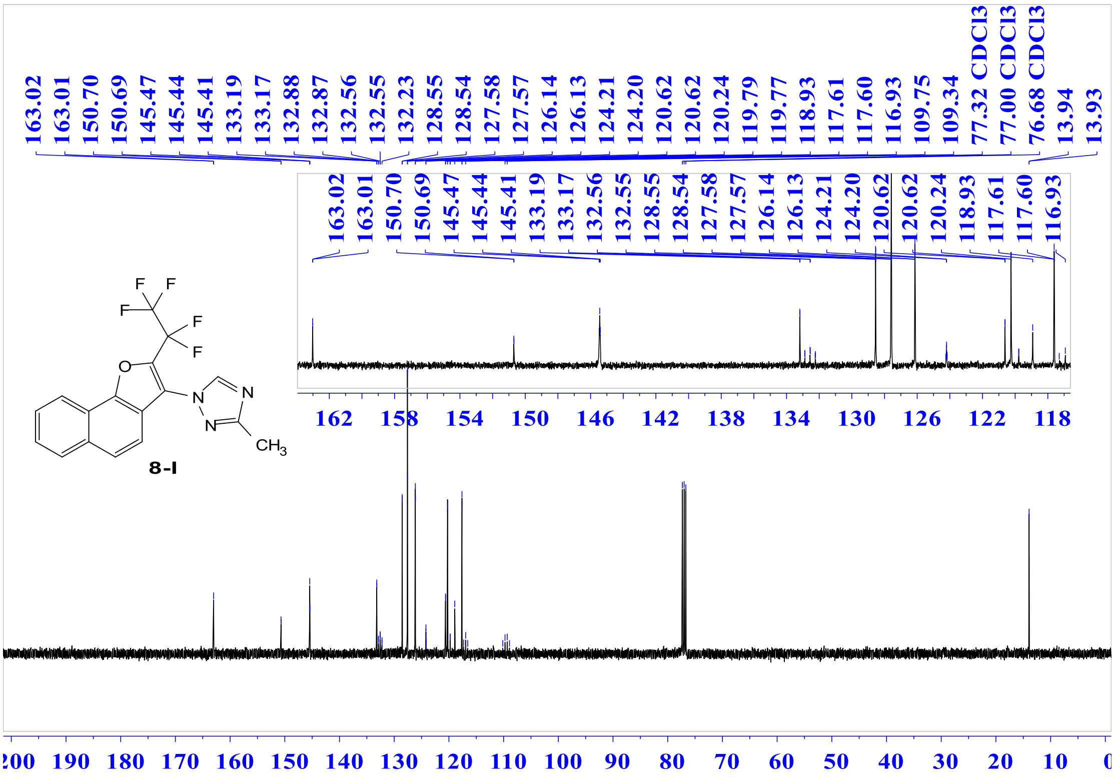

Figure S82. ^{1}H NMR spectrum of **8-II**, related to **Scheme 1**.

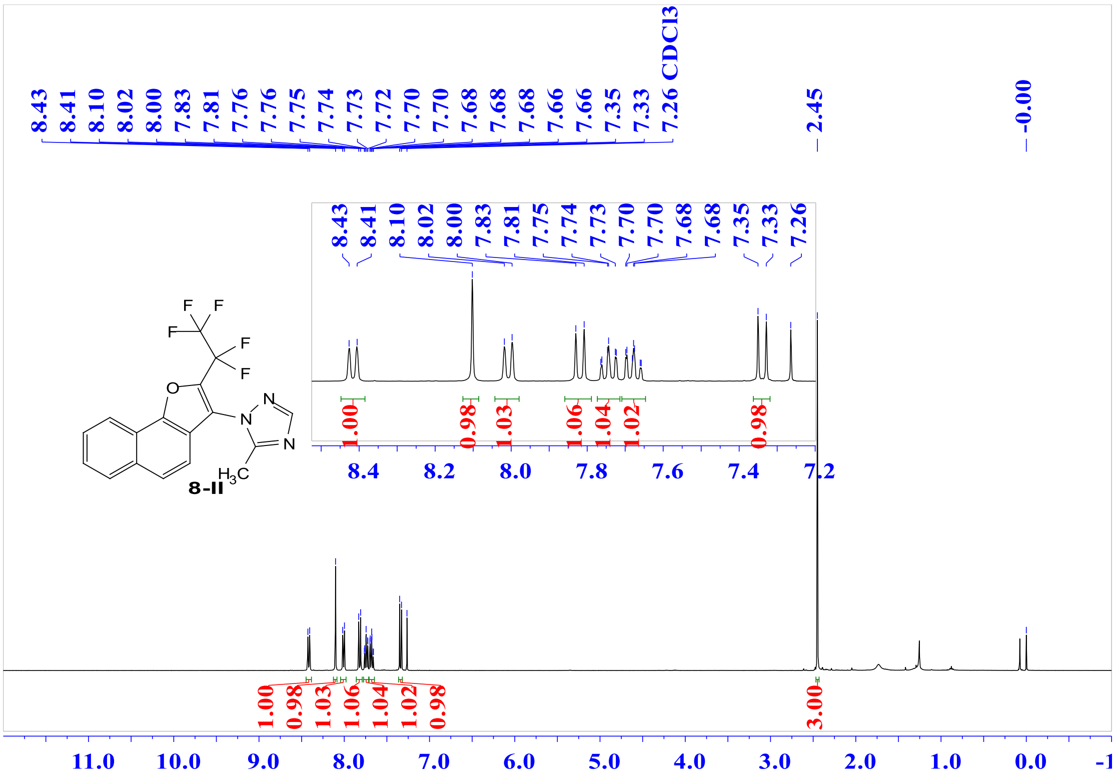

Figure S83. ^{19}F NMR spectrum of **8-II**, related to **Scheme 1**.

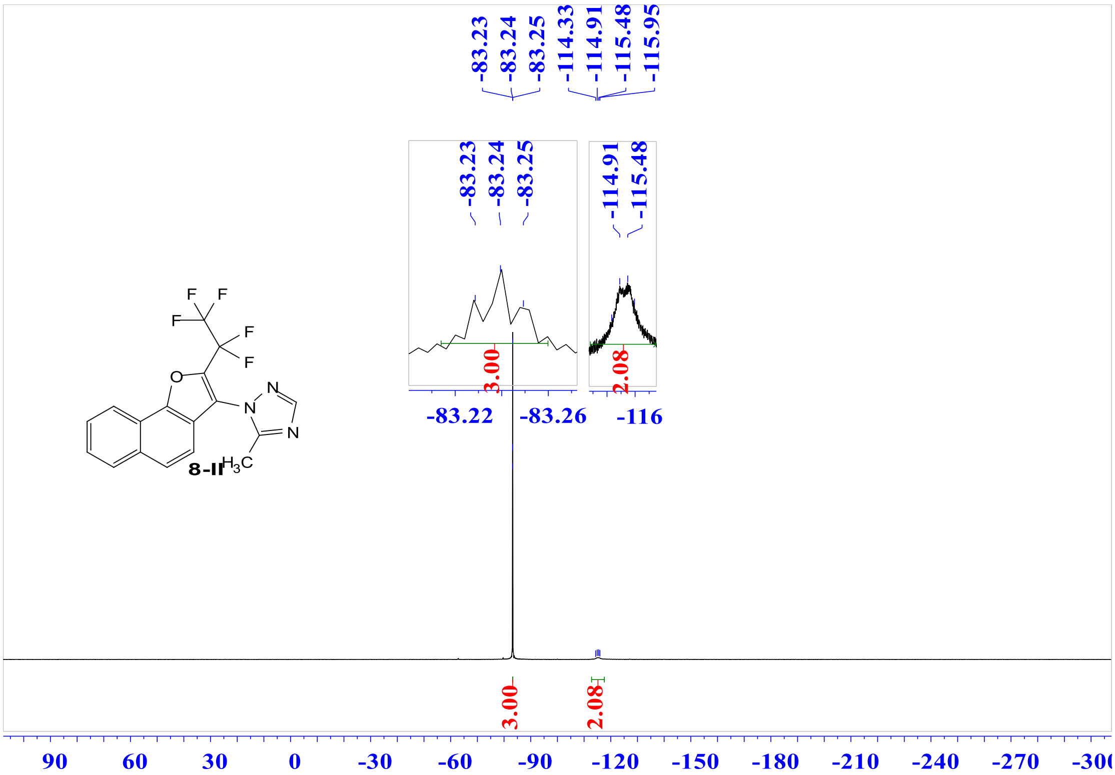

Figure S84. ^{13}C NMR spectrum of **8-II**, related to **Scheme 1**.

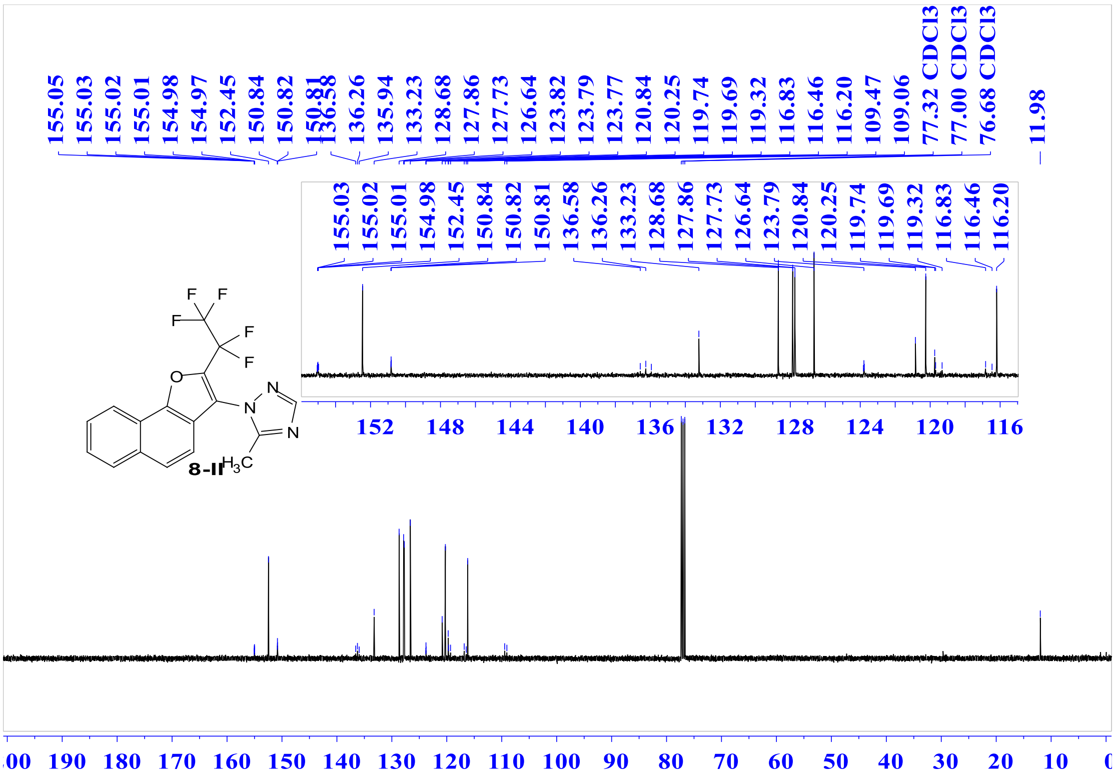

Figure S85. ^{1}H NMR spectrum of **9**, related to **Scheme 1**.

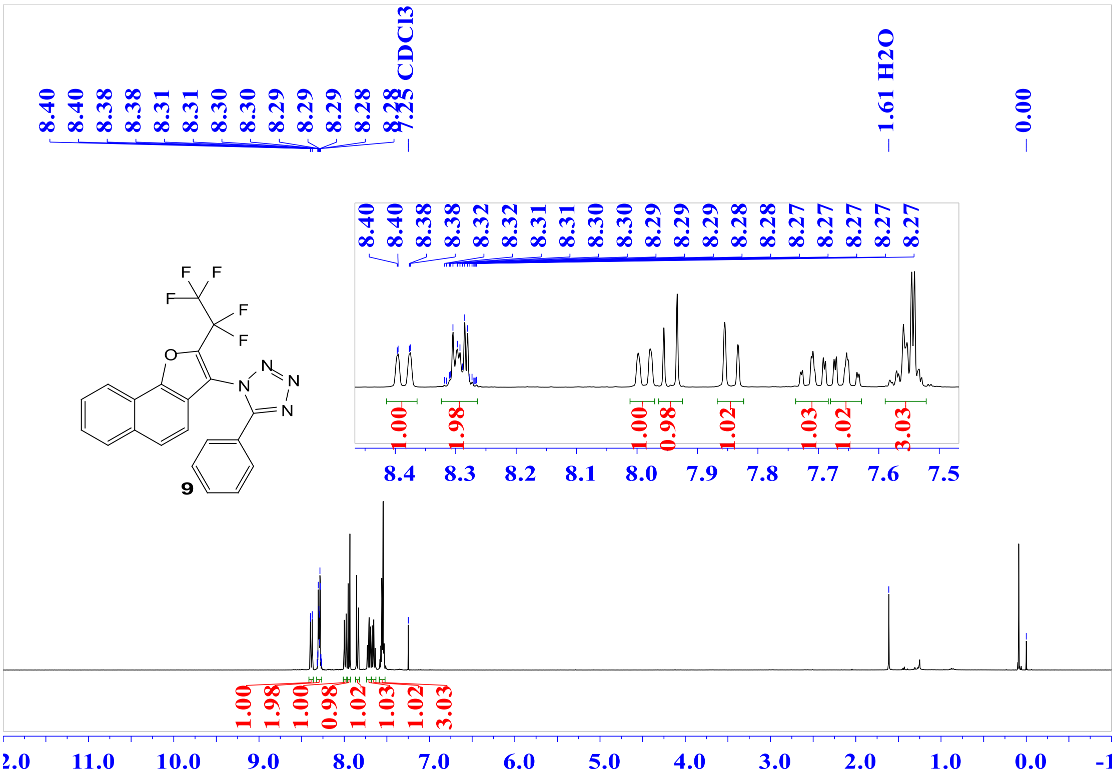

Figure S86. ^{19}F NMR spectrum of **9**, related to **Scheme 1**.

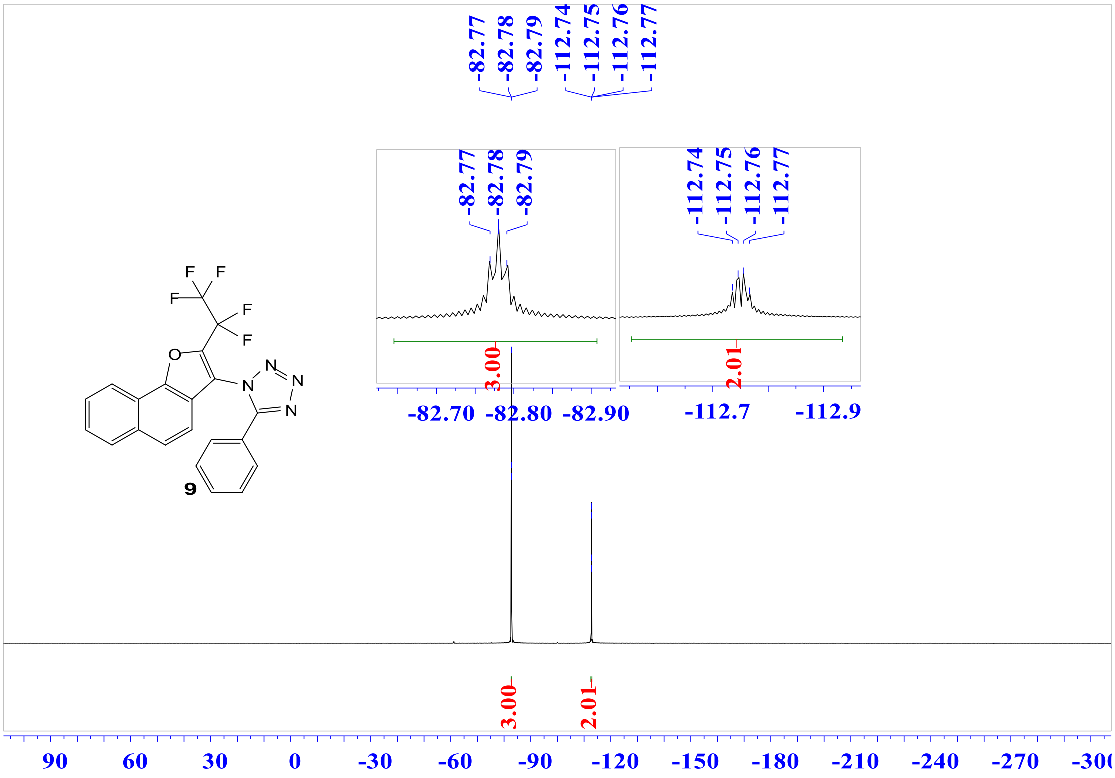

Figure S87. ^{13}C NMR spectrum of **9**, related to **Scheme 1**.

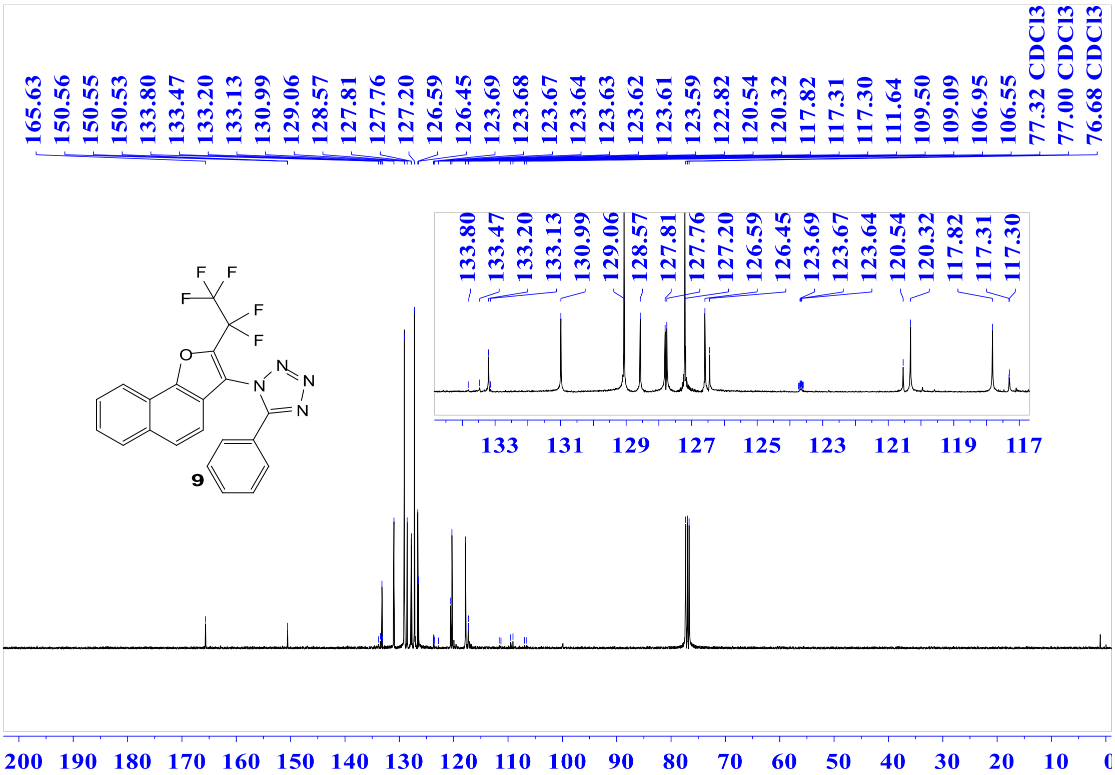

Figure S88. ^{1}H NMR spectrum of **10a**, related to **Scheme 1**.

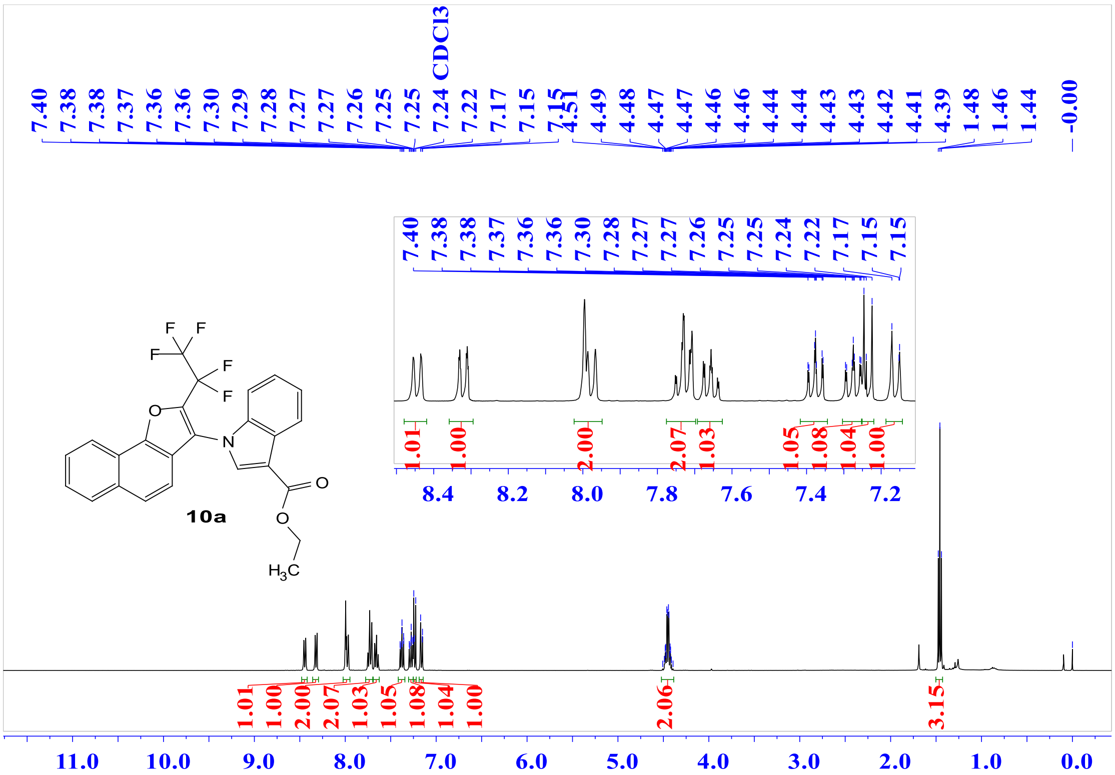

Figure S89. ^{19}F NMR spectrum of **10a**, related to **Scheme 1**.

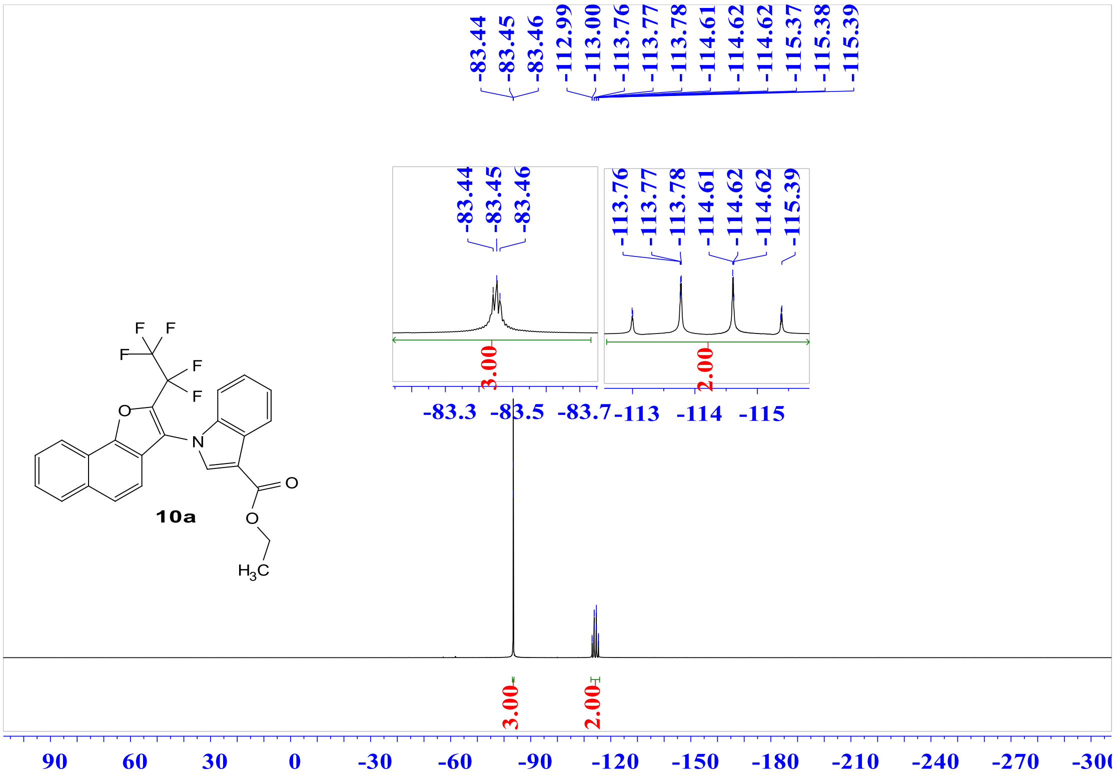

Figure S90. ^{13}C NMR spectrum of **10a**, related to **Scheme 1**.

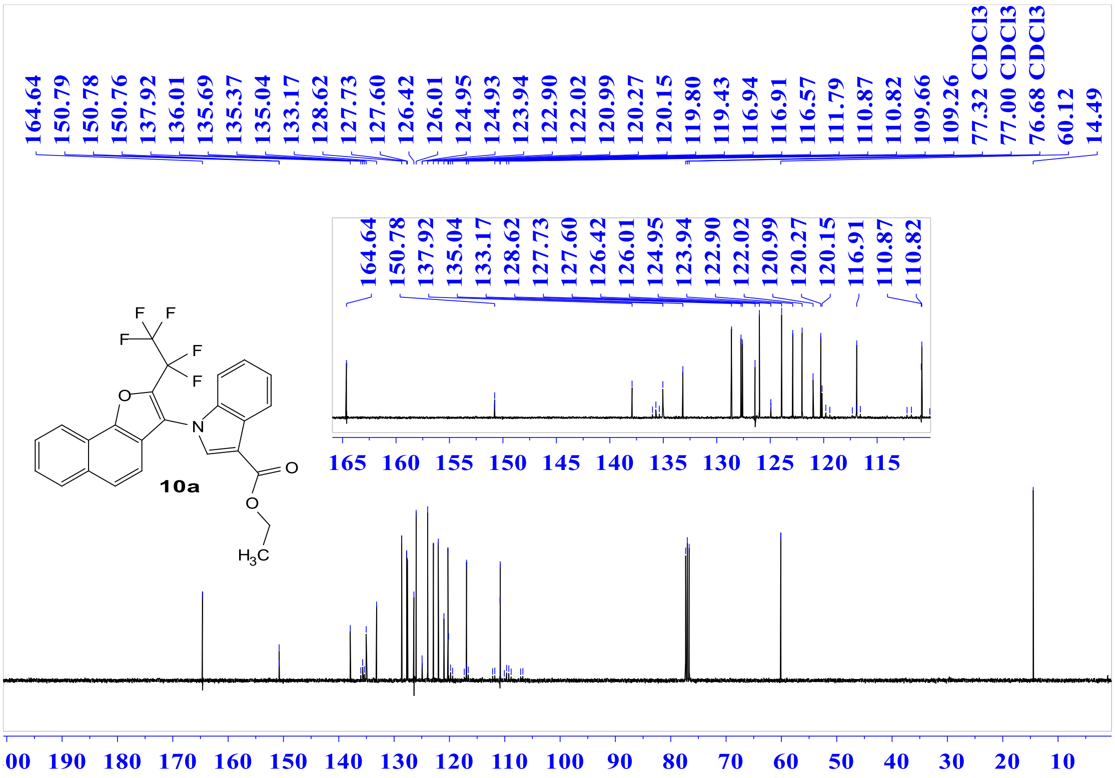

Figure S91. ^{1}H NMR spectrum of **10b**, related to **Scheme 1**.

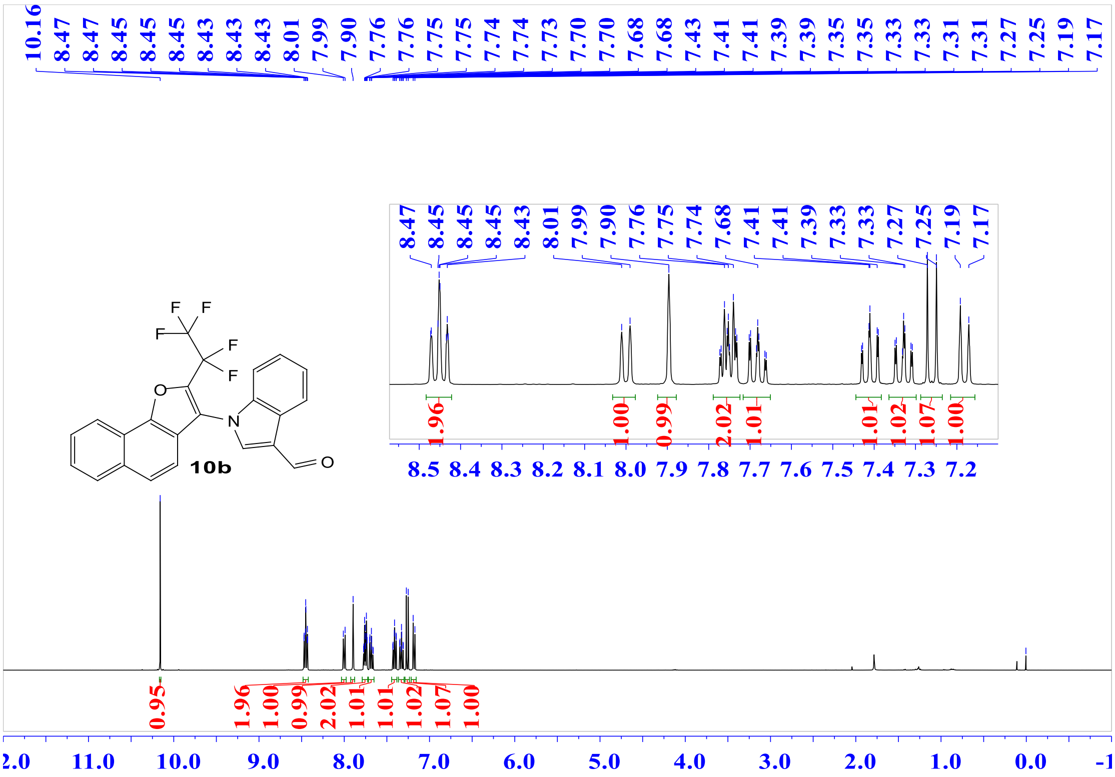

Figure S92. ^{19}F NMR spectrum of **10b**, related to **Scheme 1**.

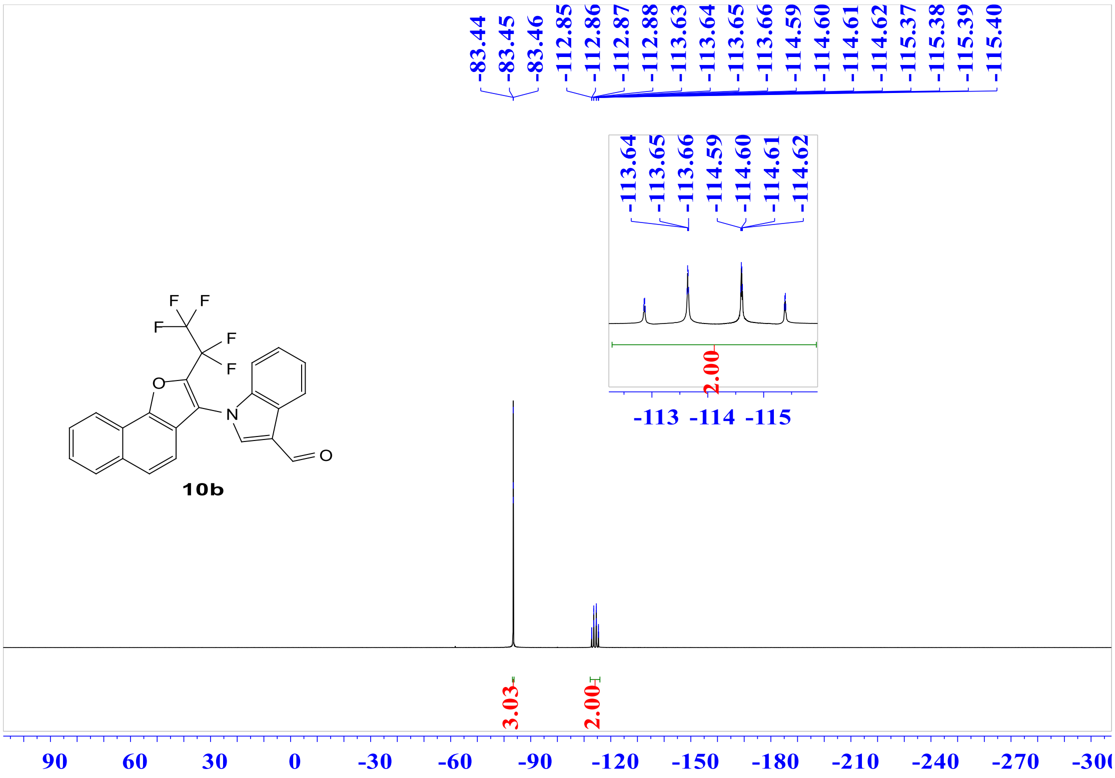

Figure S93. ^{13}C NMR spectrum of **10b**, related to **Scheme 1**.

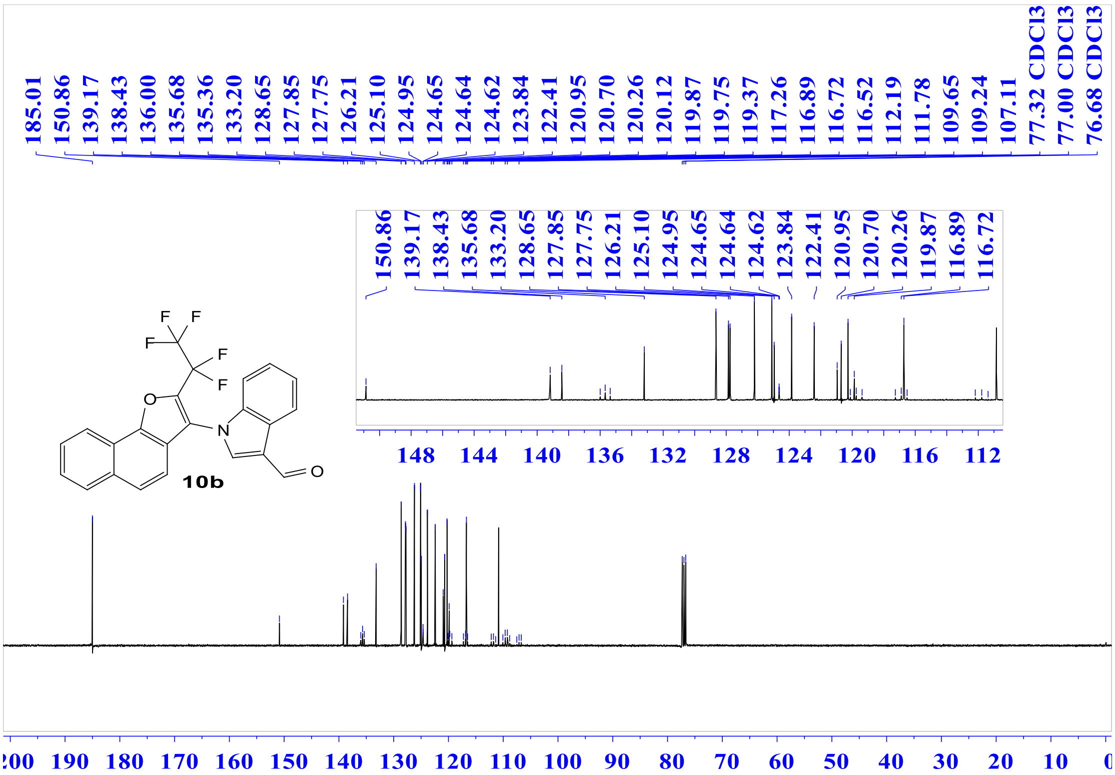

Figure S94. ^{1}H NMR spectrum of **10c**, related to **Scheme 1**.

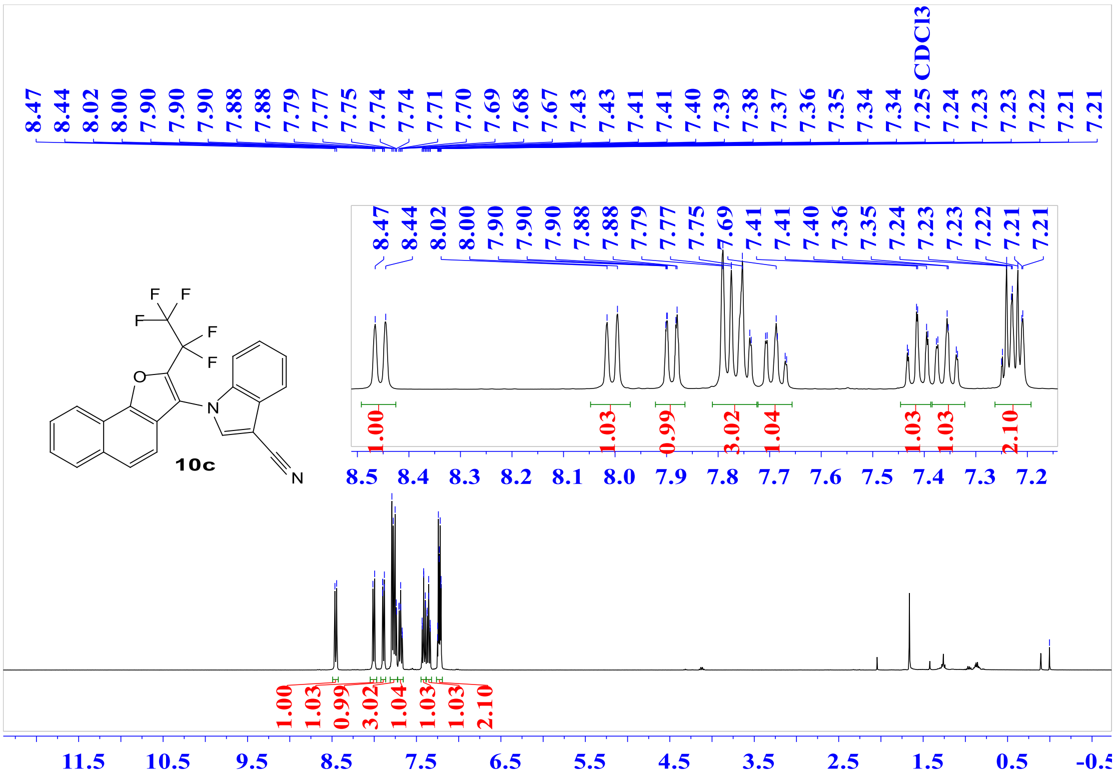

Figure S95. ^{19}F NMR spectrum of **10c**, related to **Scheme 1**.

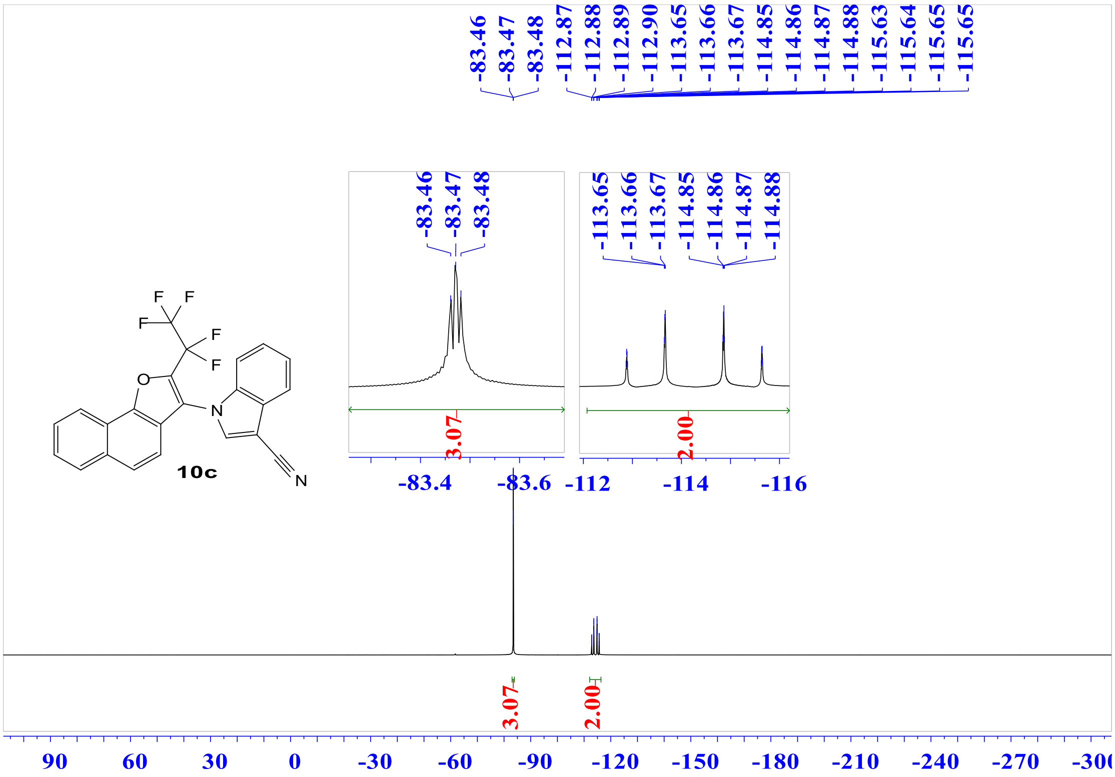

Figure S96. ^{13}C NMR spectrum of **10c**, related to **Scheme 1**.

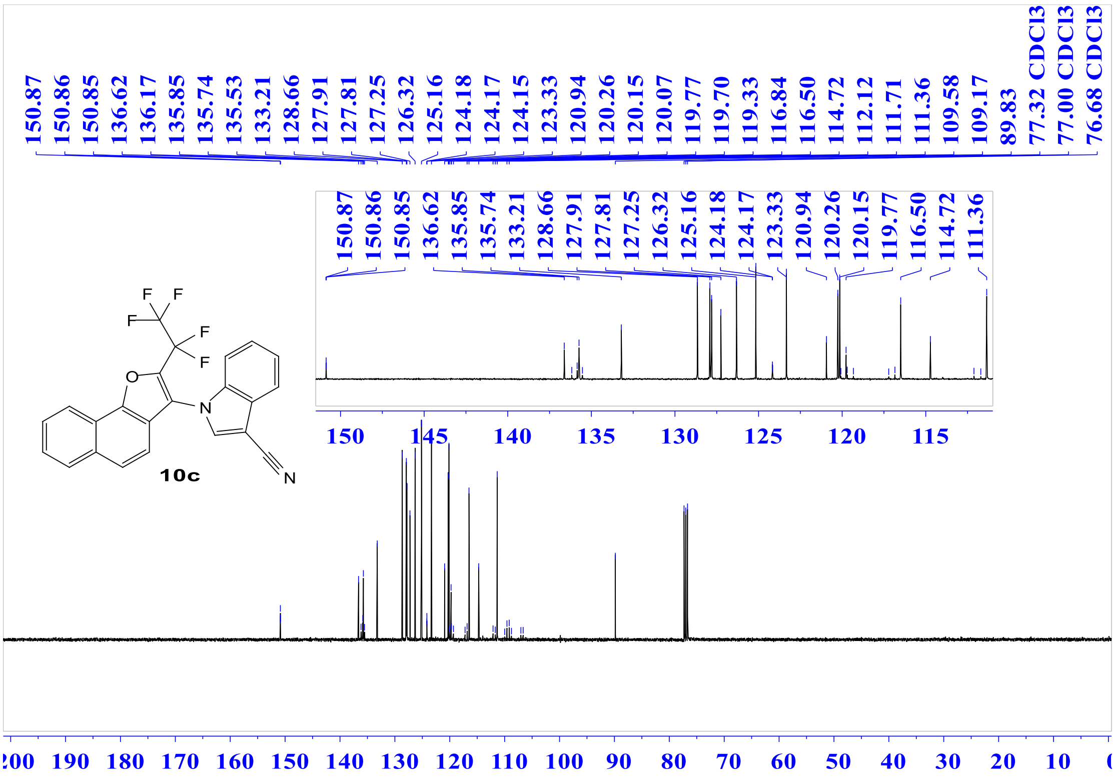

Figure S97. ^{1}H NMR spectrum of **10d**, related to **Scheme 1**.

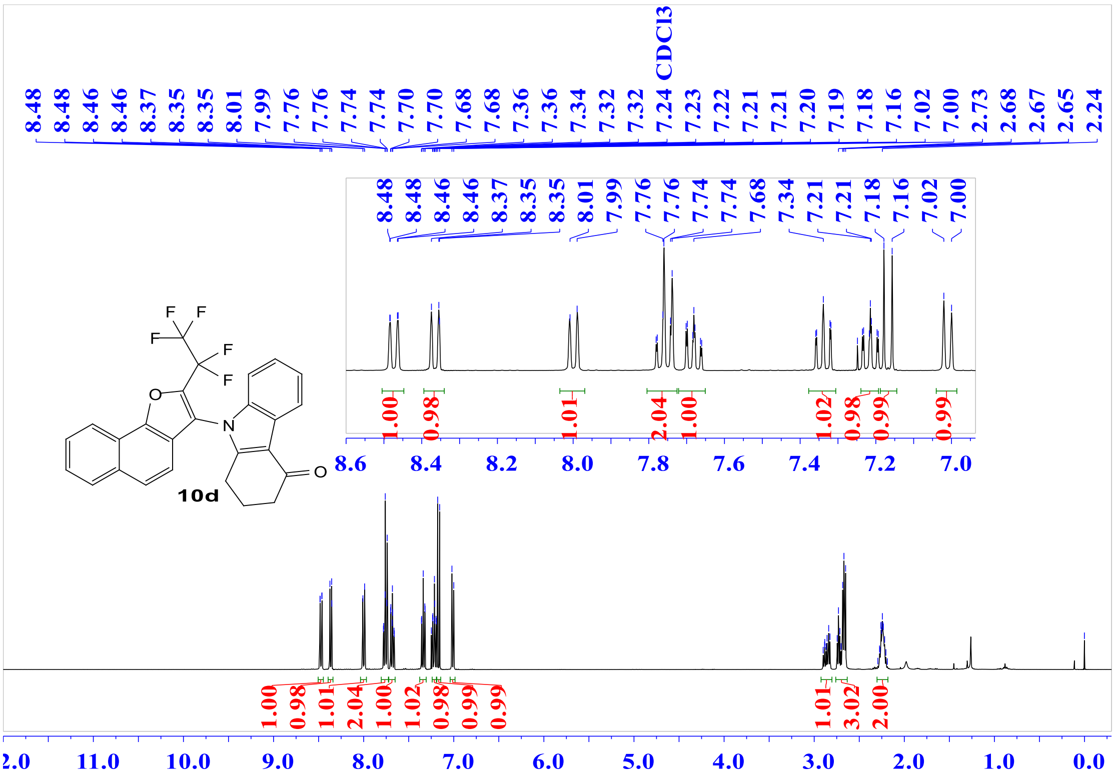

Figure S98. ^{19}F NMR spectrum of **10d**, related to **Scheme 1**.

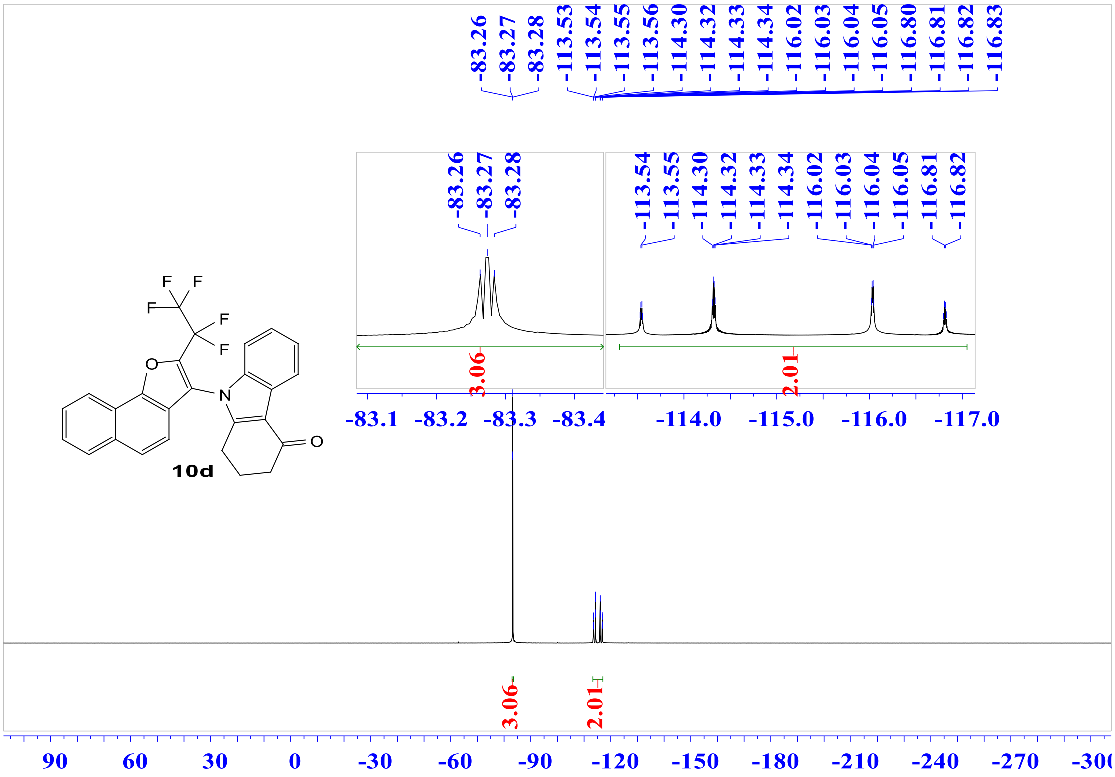

Figure S99. ^{13}C NMR spectrum of **10d**, related to **Scheme 1**.

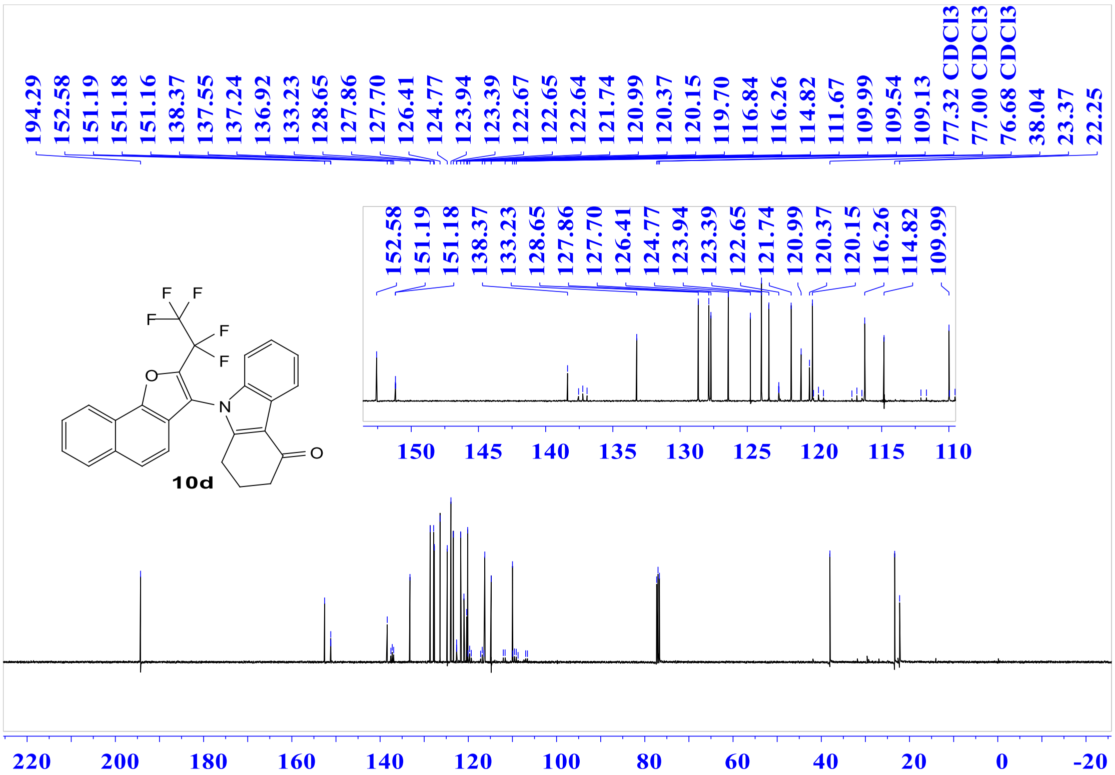

Figure S100. ^{1}H NMR spectrum of **11a**, related to **Scheme 1**.

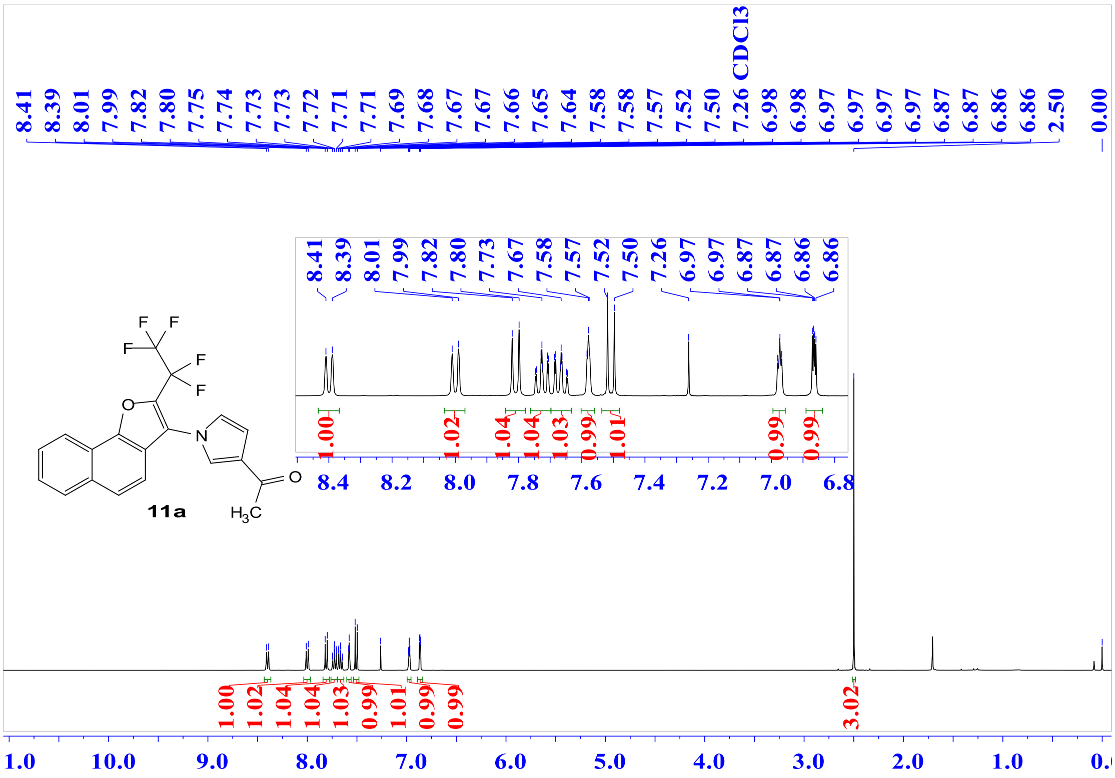

Figure S101. ^{19}F NMR spectrum of **11a**, related to **Scheme 1**.

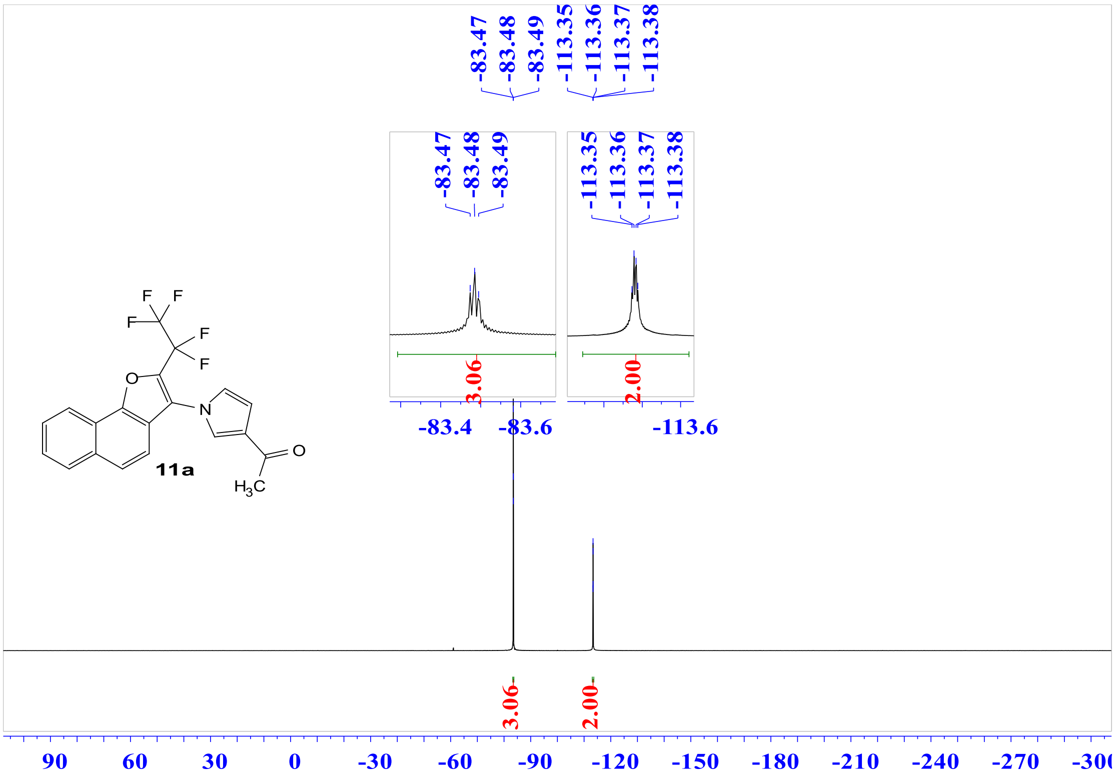

Figure S102. ^{13}C NMR spectrum of **11a**, related to **Scheme 1**.

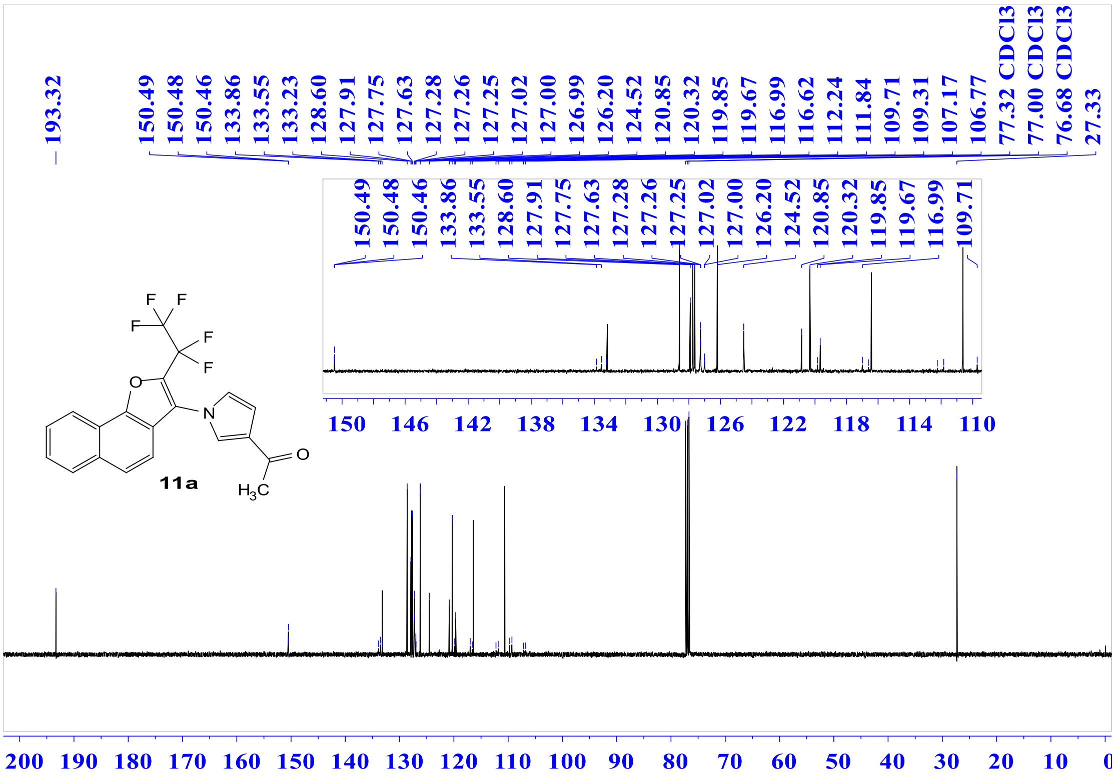

Figure S103. ^{1}H NMR spectrum of **11b**, related to **Scheme 1**.

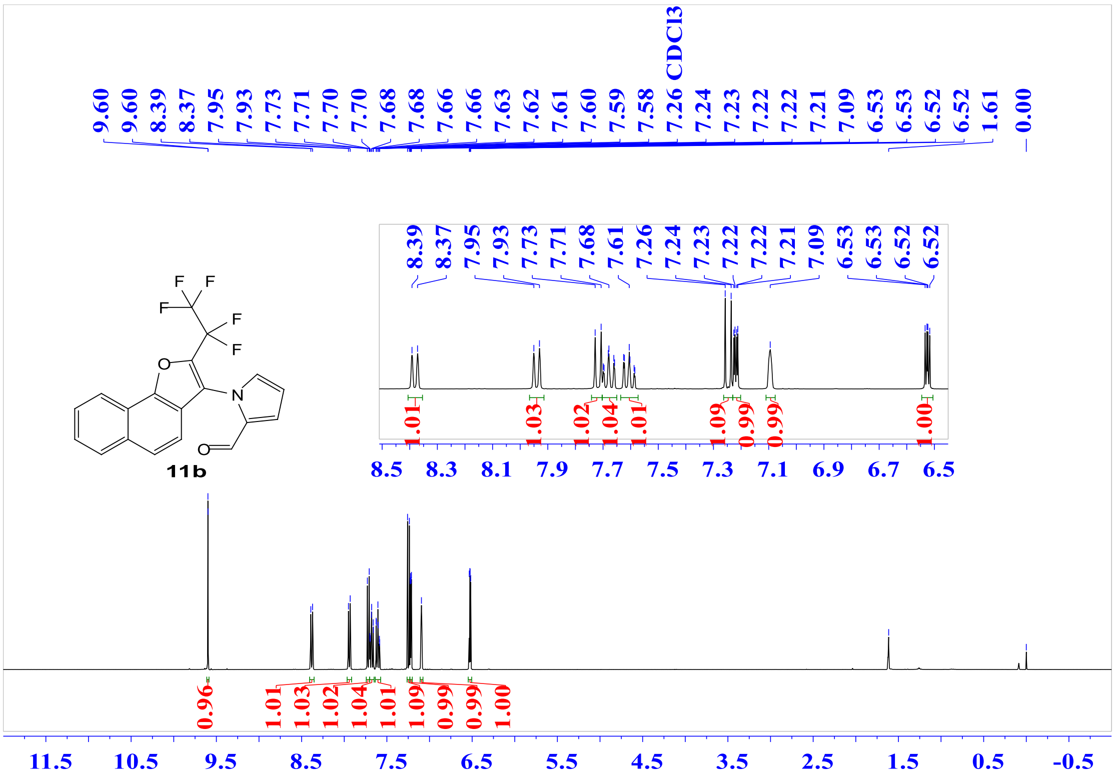

Figure S104. ^{19}F NMR spectrum of **11b**, related to **Scheme 1**.

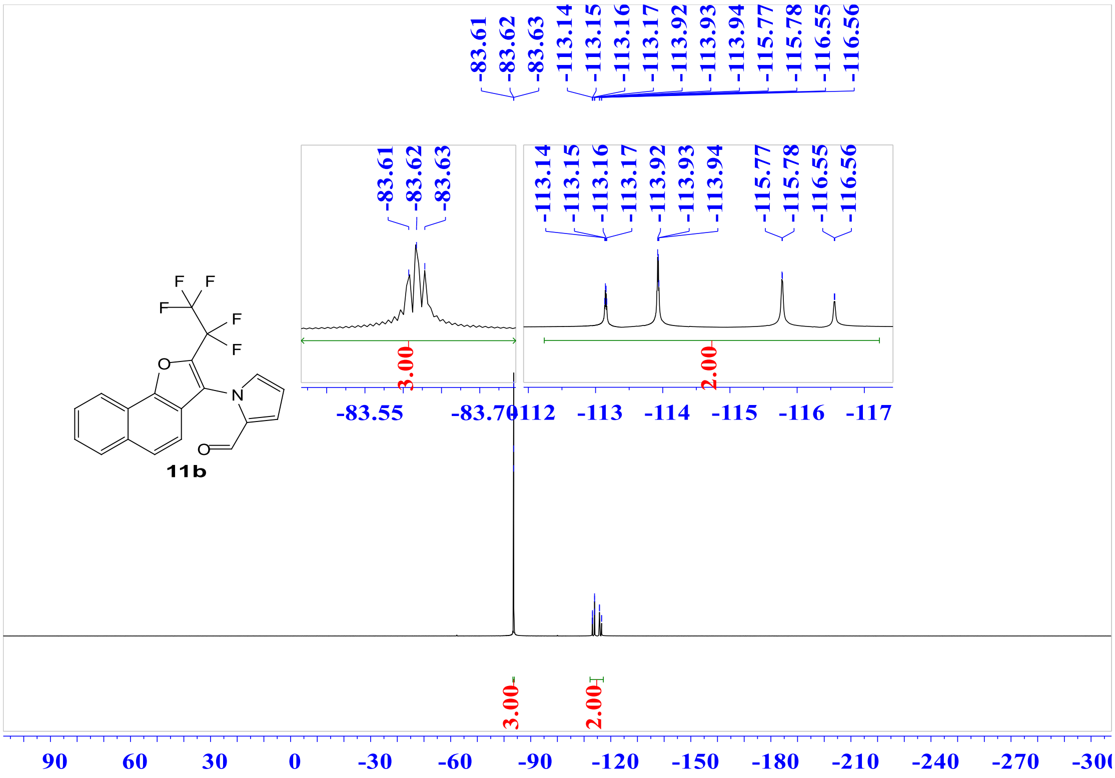

Figure S105. ^{13}C NMR spectrum of **11b**, related to **Scheme 1**.

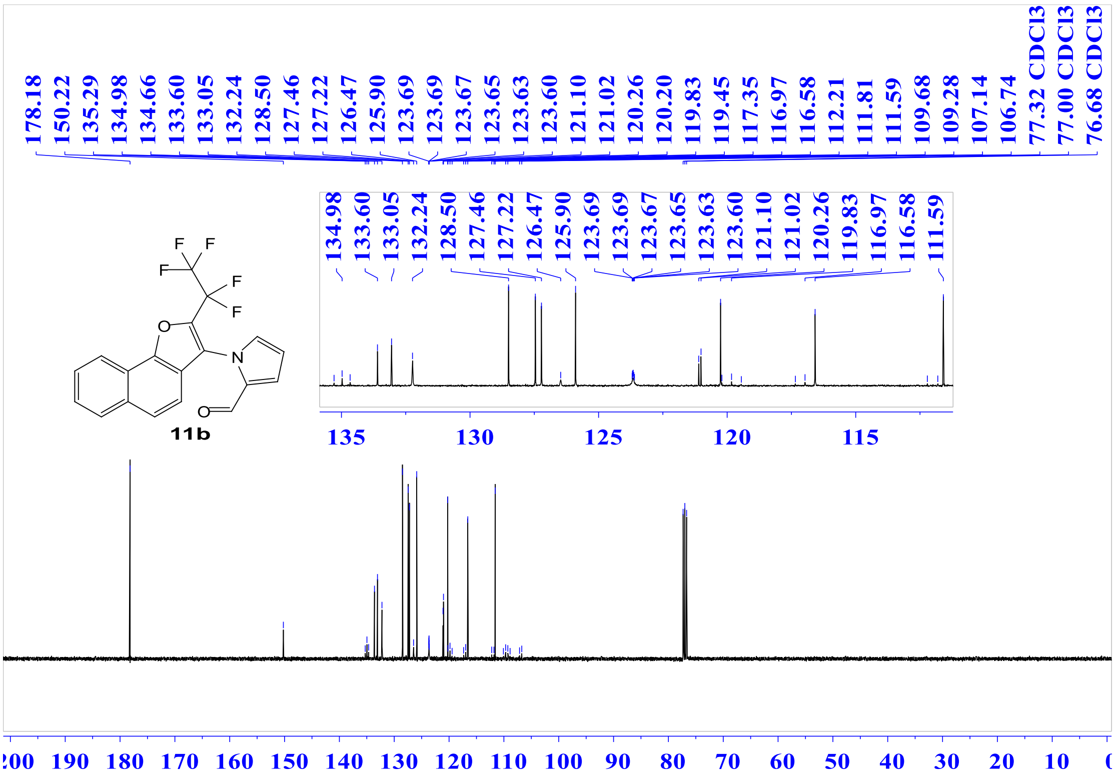

Figure S106. ^{1}H NMR spectrum of **11c**, related to **Scheme 1**.

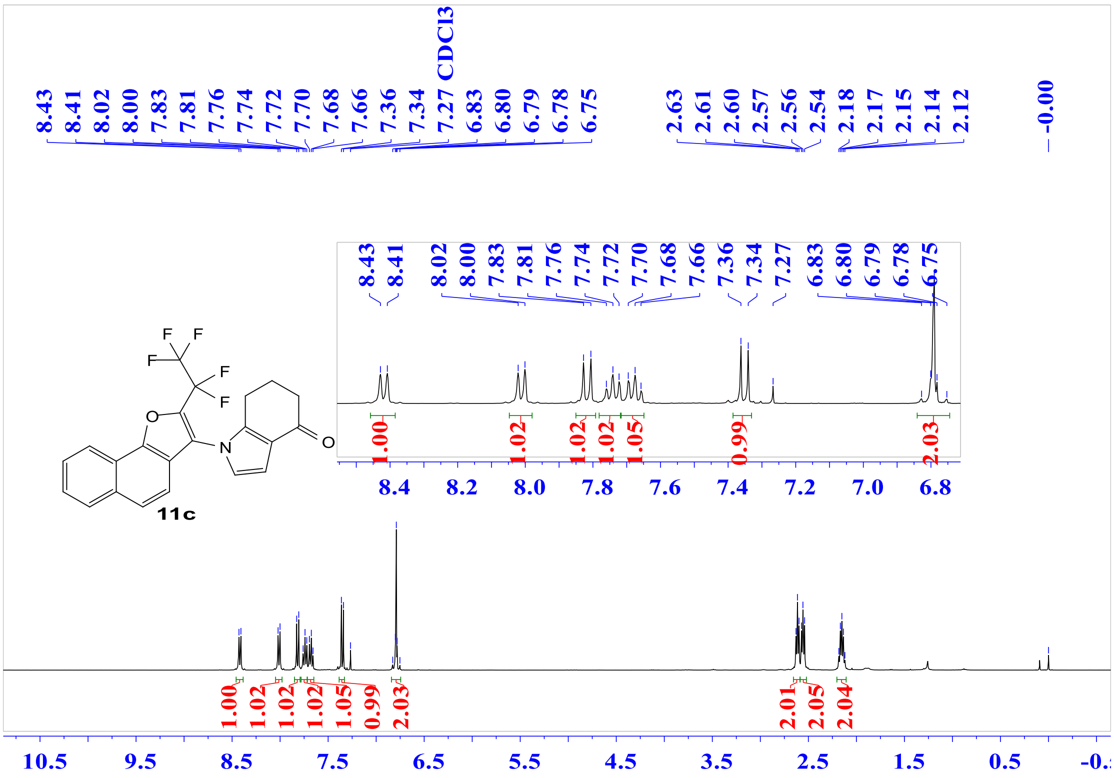

Figure S107. ^{19}F NMR spectrum of **11c**, related to **Scheme 1**.

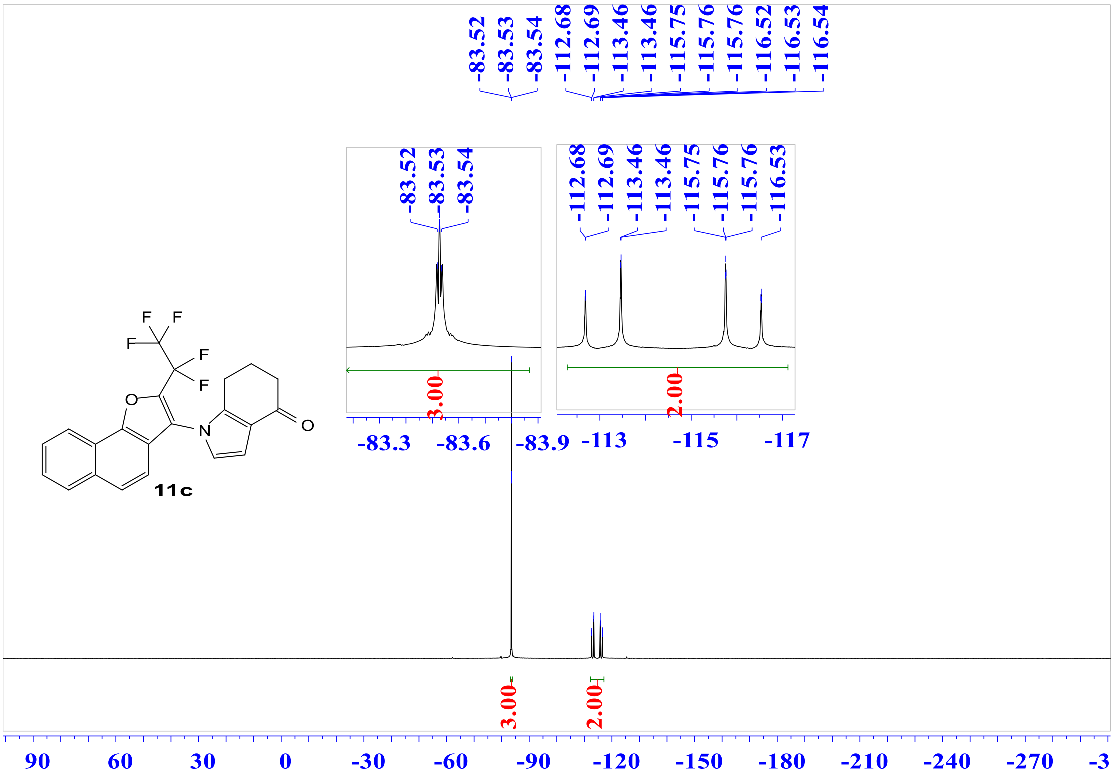

Figure S108. ^{13}C NMR spectrum of **11c**, related to **Scheme 1**.

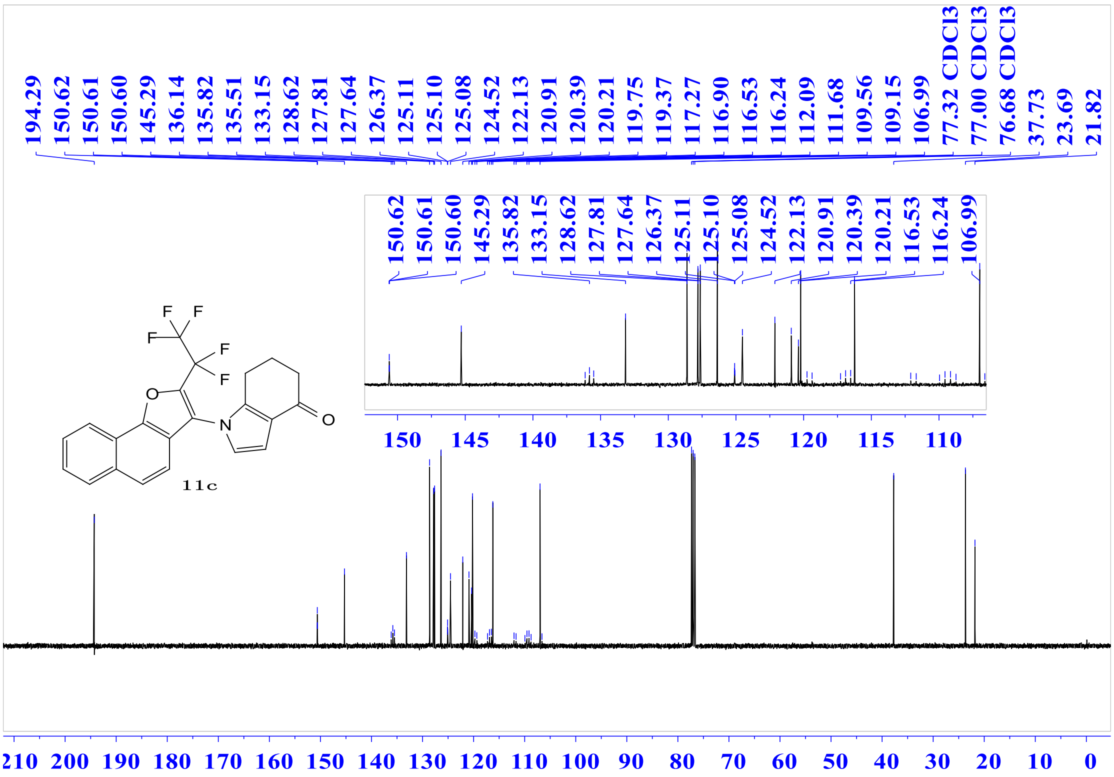

Figure S109. ^{1}H NMR spectrum of **12**, related to **Scheme 1**.

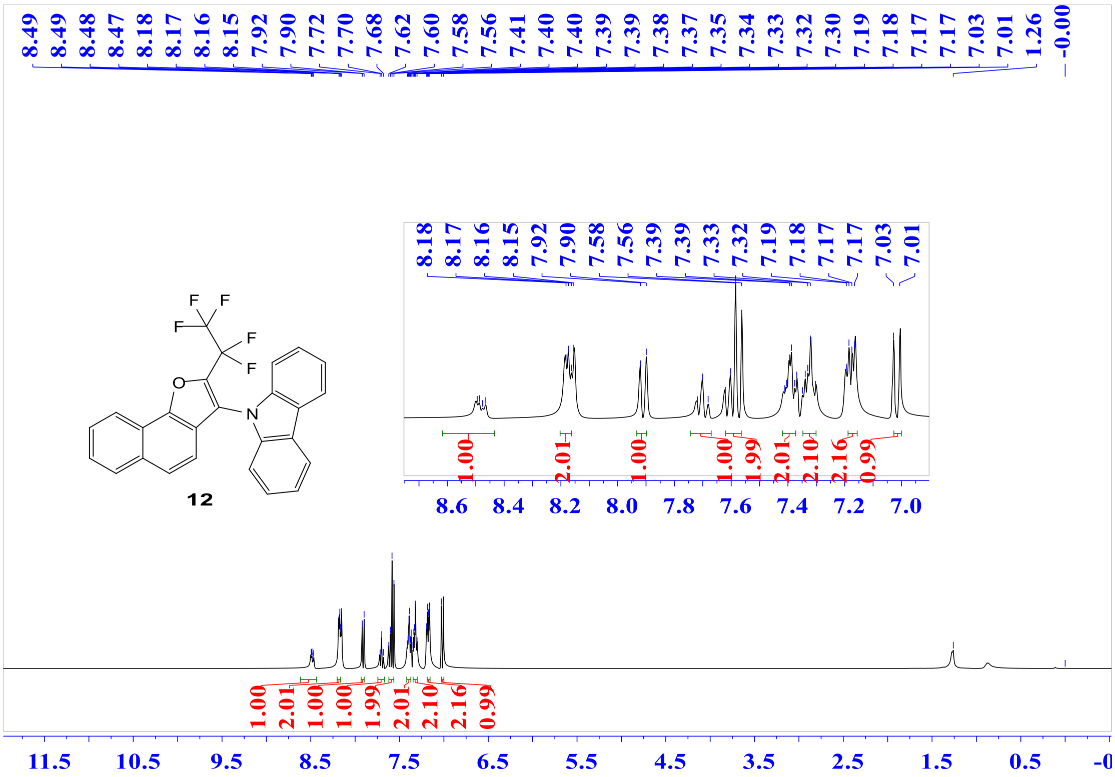

Figure S110. ^{19}F NMR spectrum of **12**, related to **Scheme 1**.

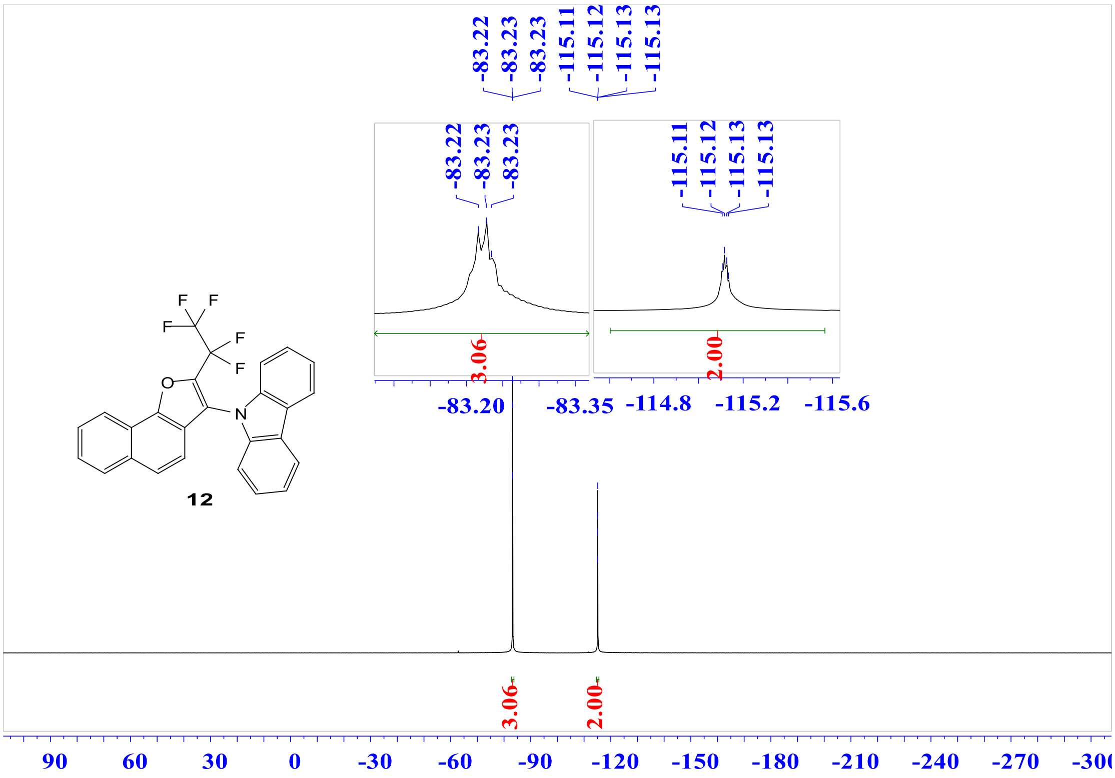

Figure S111. ^{13}C NMR spectrum of **12**, related to **Scheme 1**.

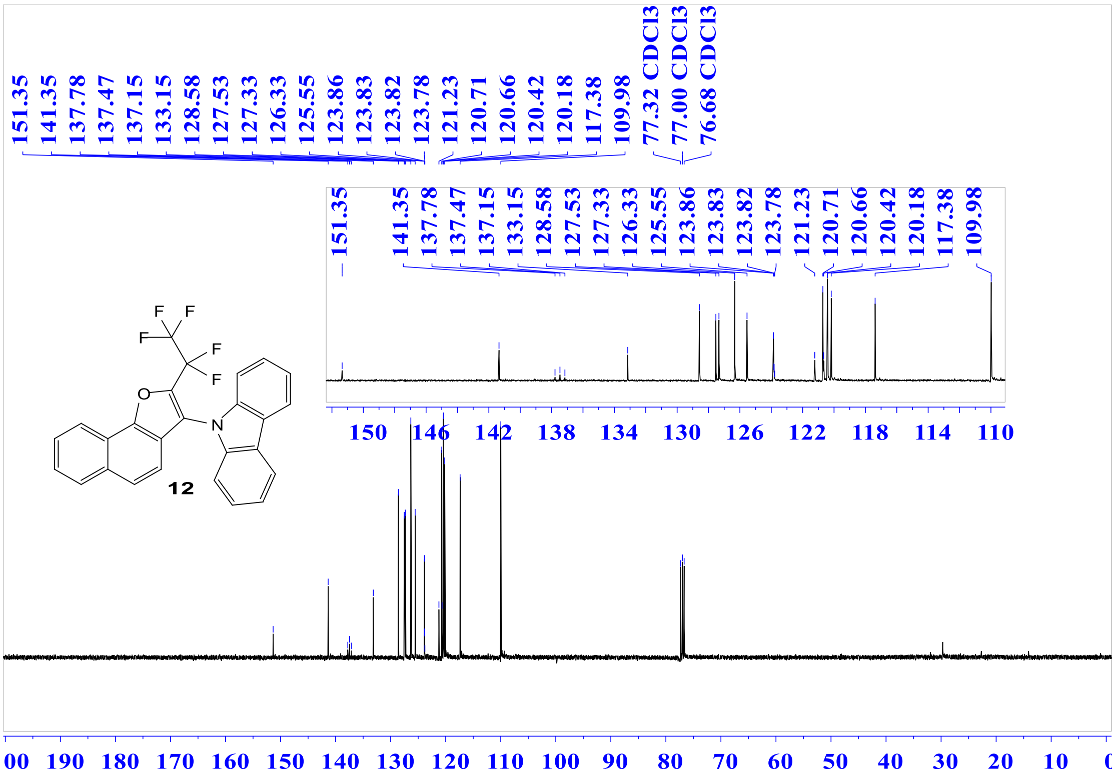

Figure S112. ^{1}H NMR spectrum of **13**, related to **Scheme 1**.

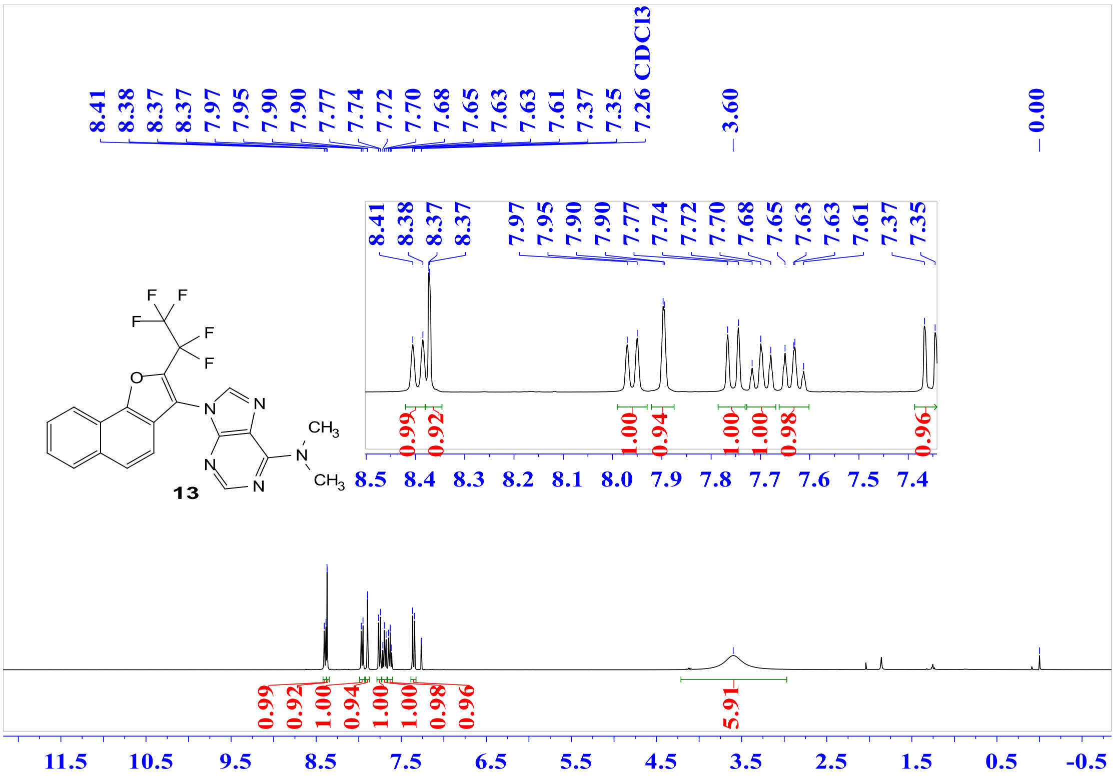

Figure S113. ^{19}F NMR spectrum of **13**, related to **Scheme 1**.

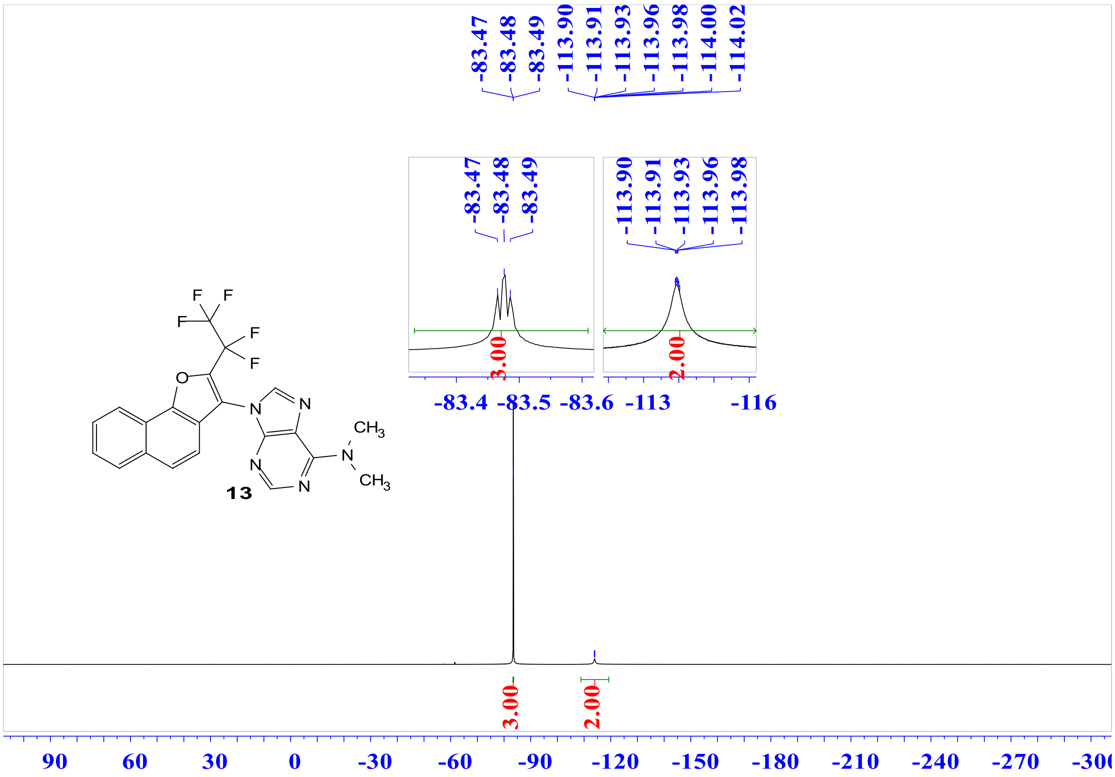

Figure S114. ^{13}C NMR spectrum of **13**, related to **Scheme 1**.

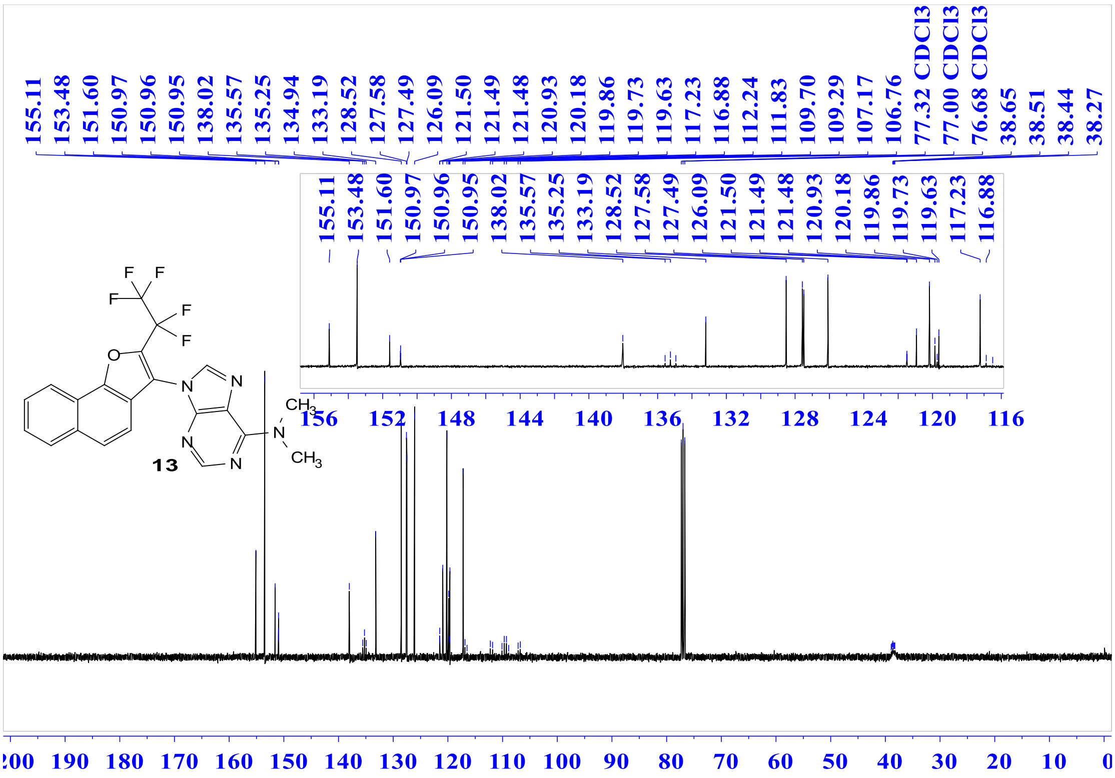

Figure S115. ^{1}H NMR spectrum of **15a**, related to **Scheme 2**.

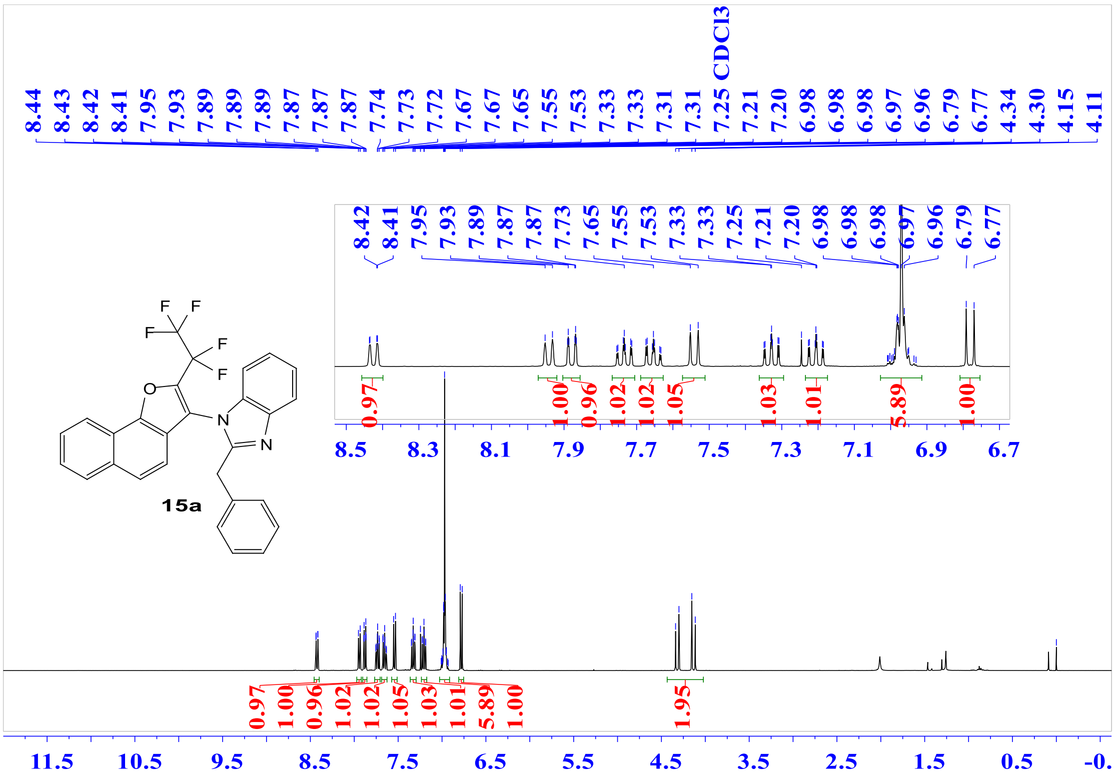

Figure S116. ^{19}F NMR spectrum of **15a**, related to **Scheme 2**.

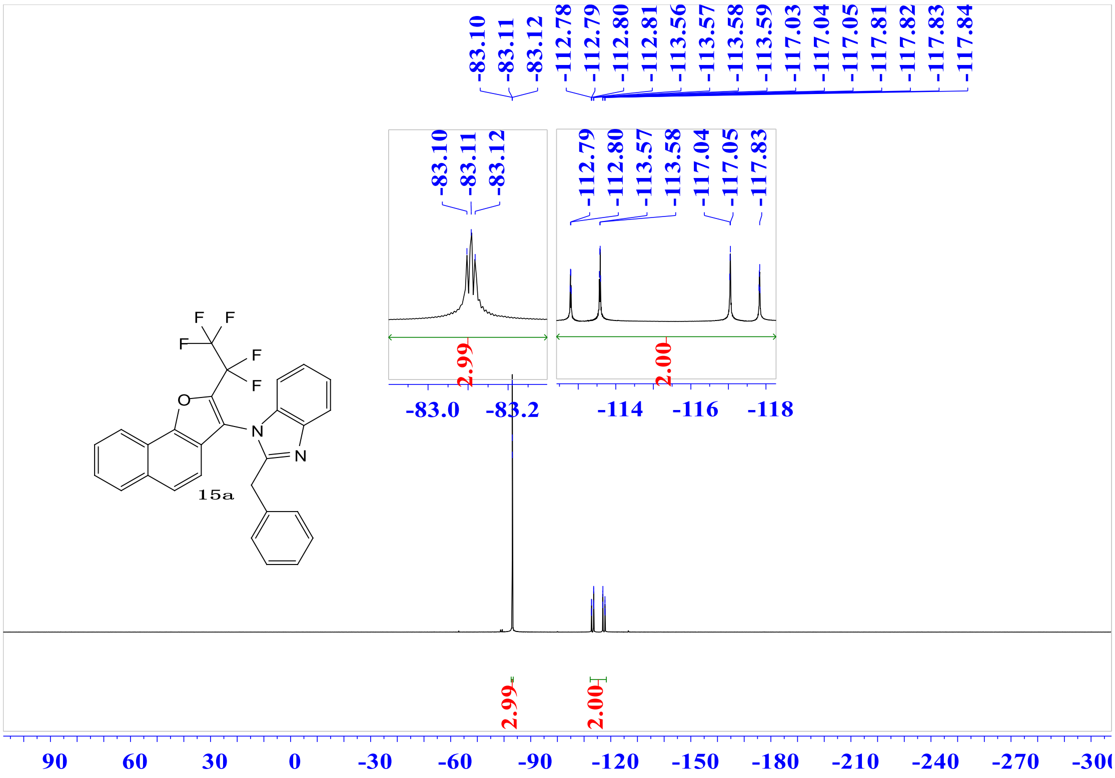

Figure S117. ^{13}C NMR spectrum of **15a**, related to **Scheme 2**.

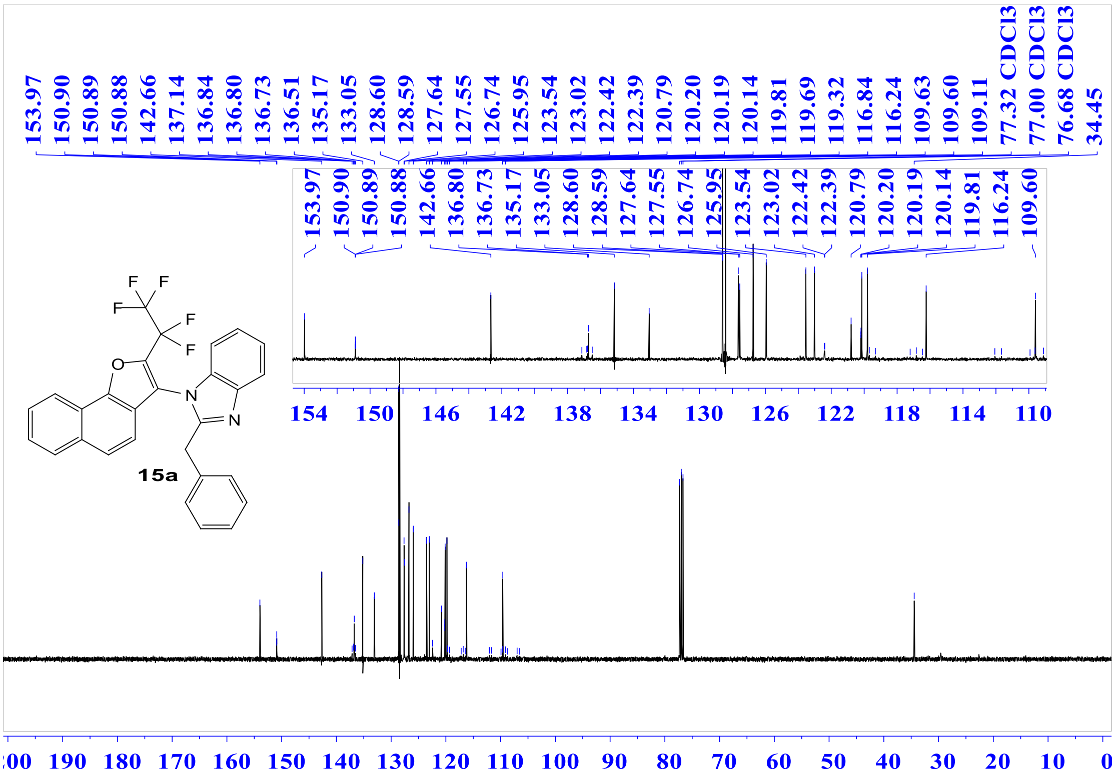

Figure S118. ^{1}H NMR spectrum of **15b**, related to **Scheme 2**.

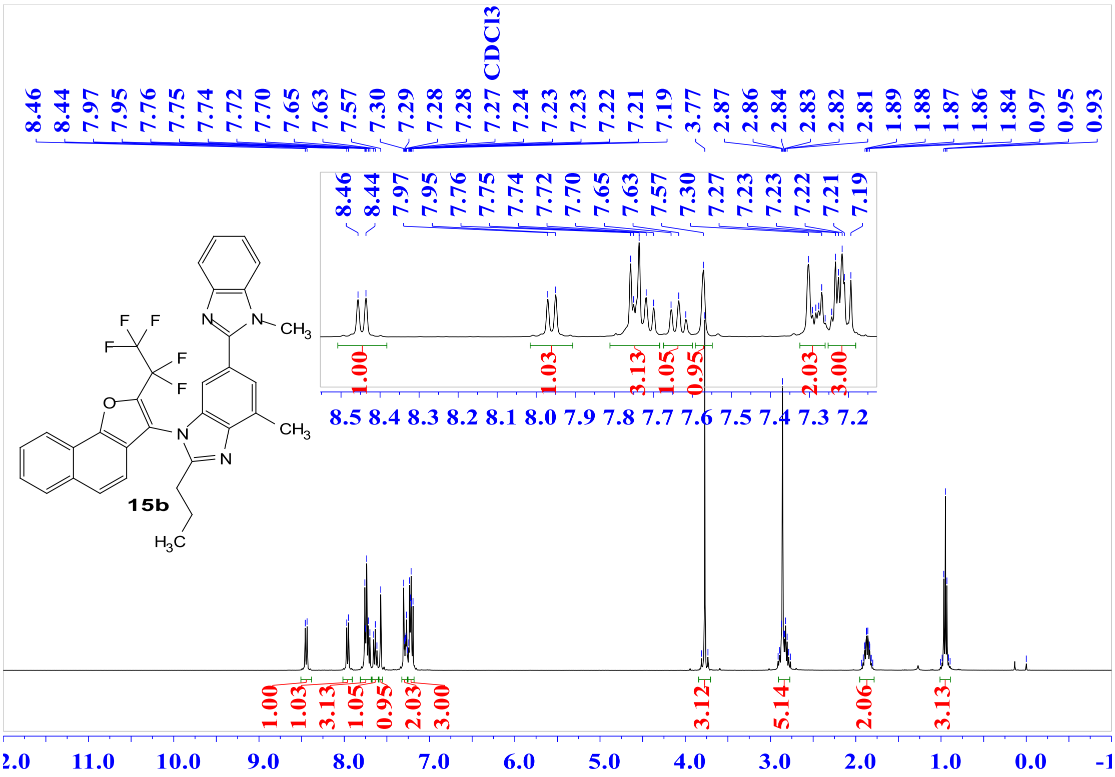

Figure S119. ^{19}F NMR spectrum of **15b**, related to **Scheme 2**.

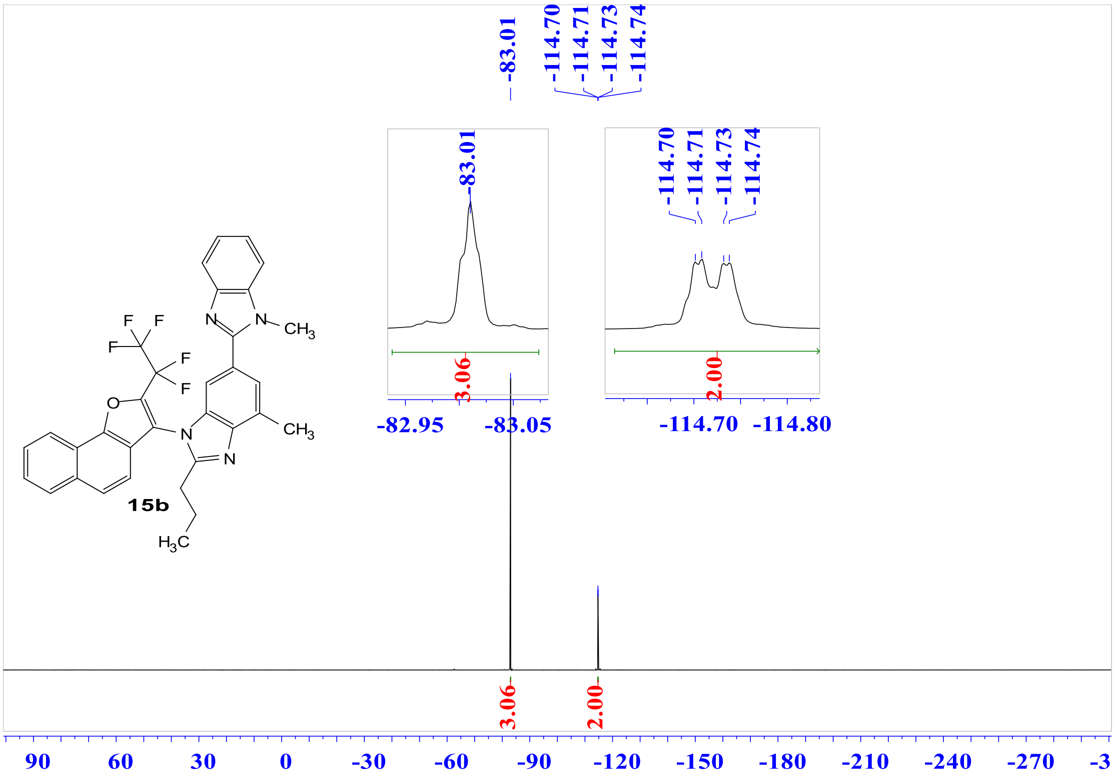

Figure S120. ^{13}C NMR spectrum of **15b**, related to **Scheme 2**.

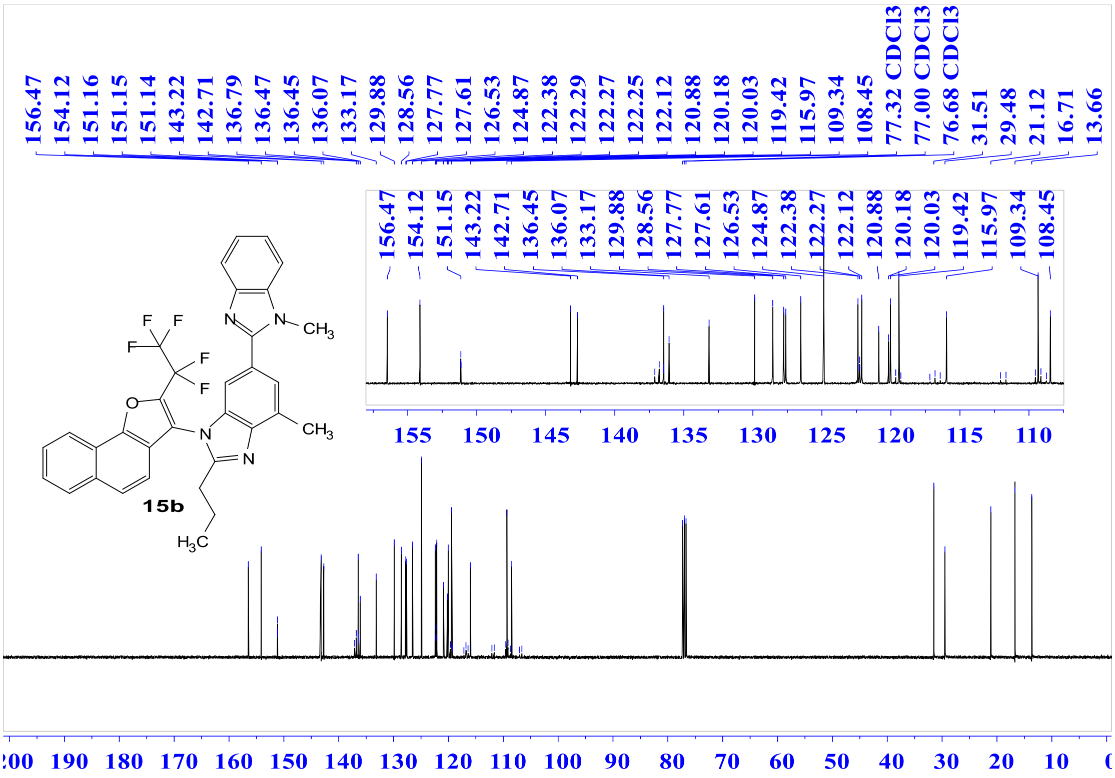

Figure S121. ^{1}H NMR spectrum of **15c**, related to **Scheme 2**.

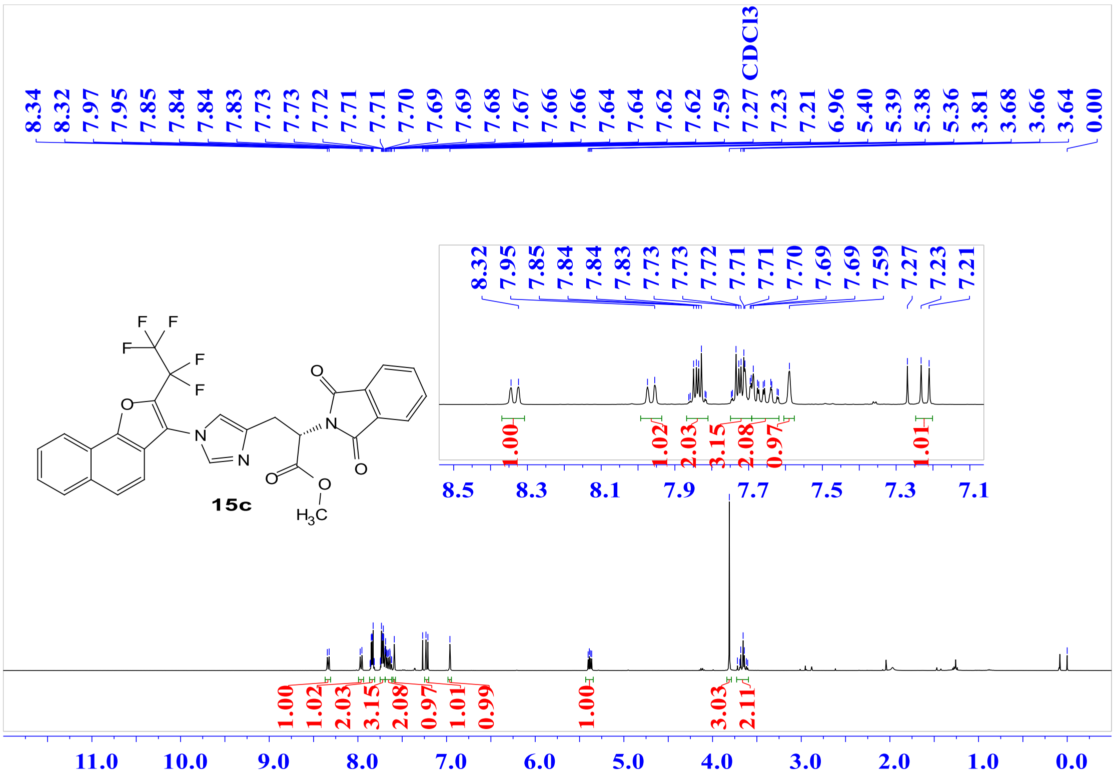

Figure S122. ^{19}F NMR spectrum of **15c**, related to **Scheme 2**.

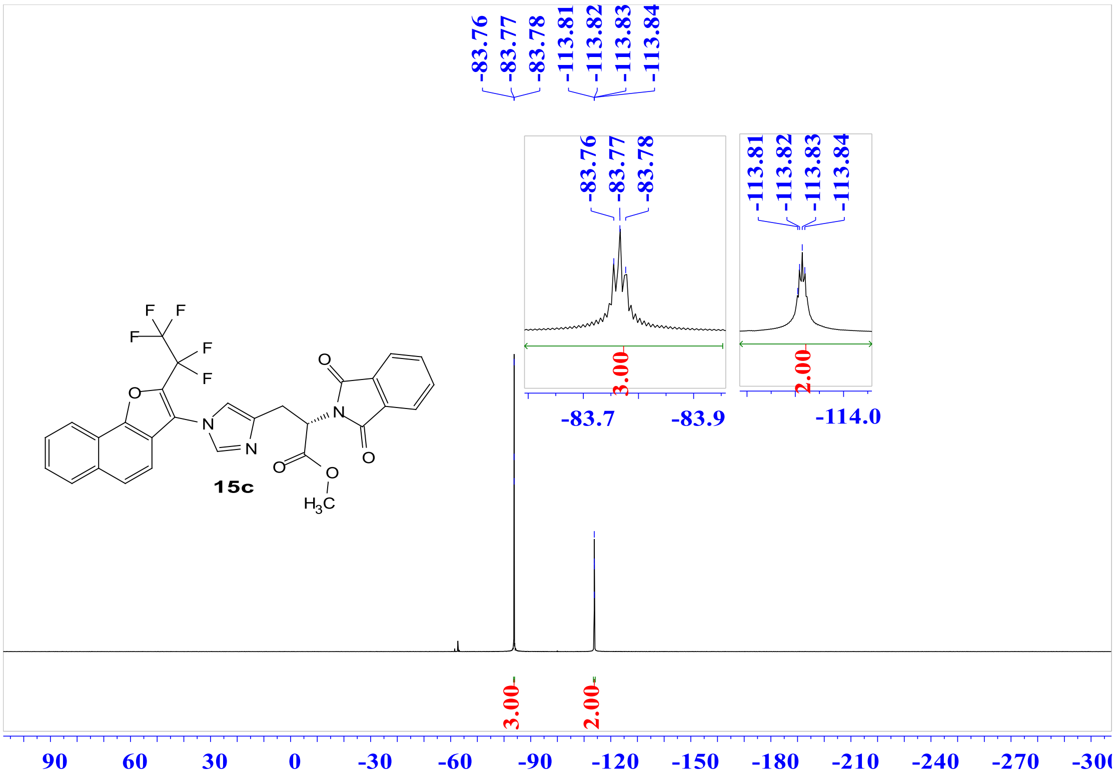

Figure S123. ^{13}C NMR spectrum of **15c**, related to **Scheme 2**.

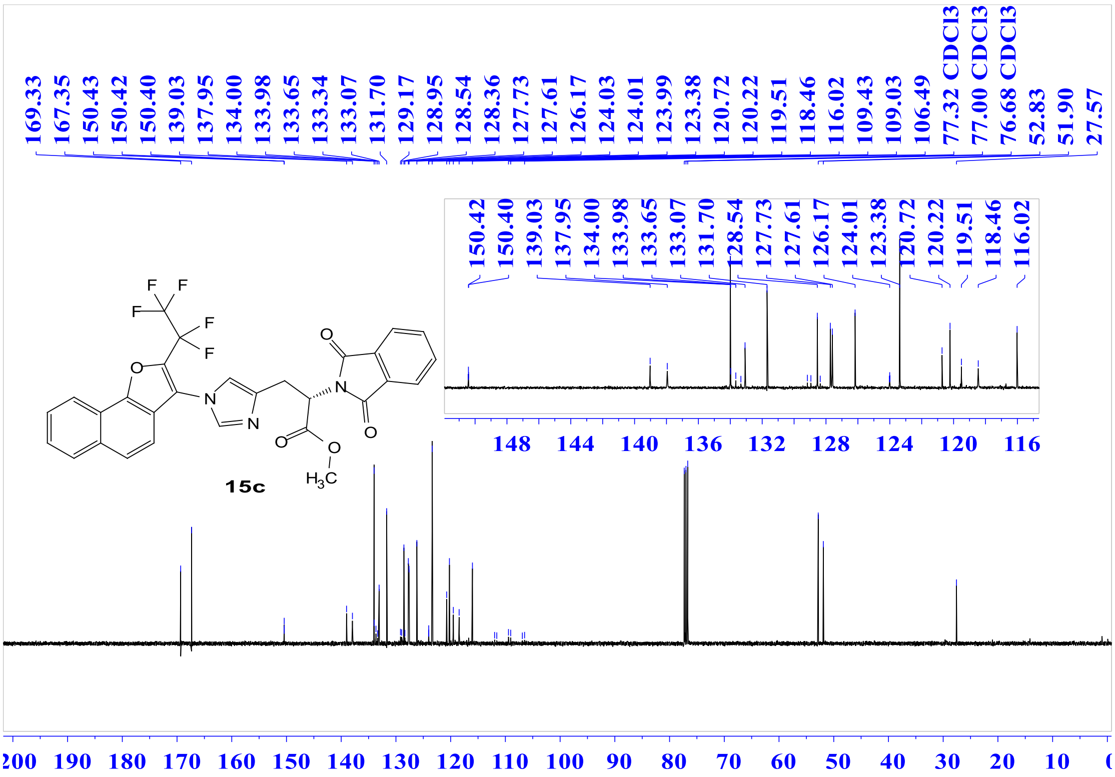

Figure S124. ^{1}H NMR spectrum of **15d**, related to **Scheme 2**.

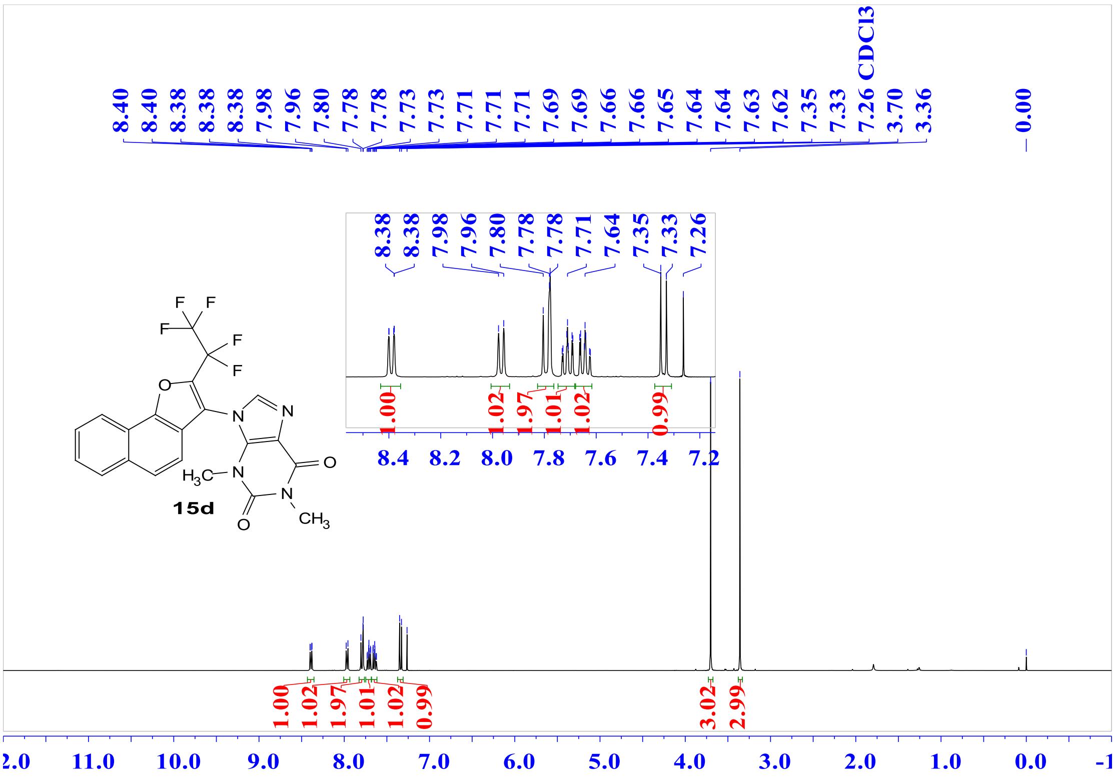

Figure S125. ^{19}F NMR spectrum of **15d**, related to **Scheme 2**.

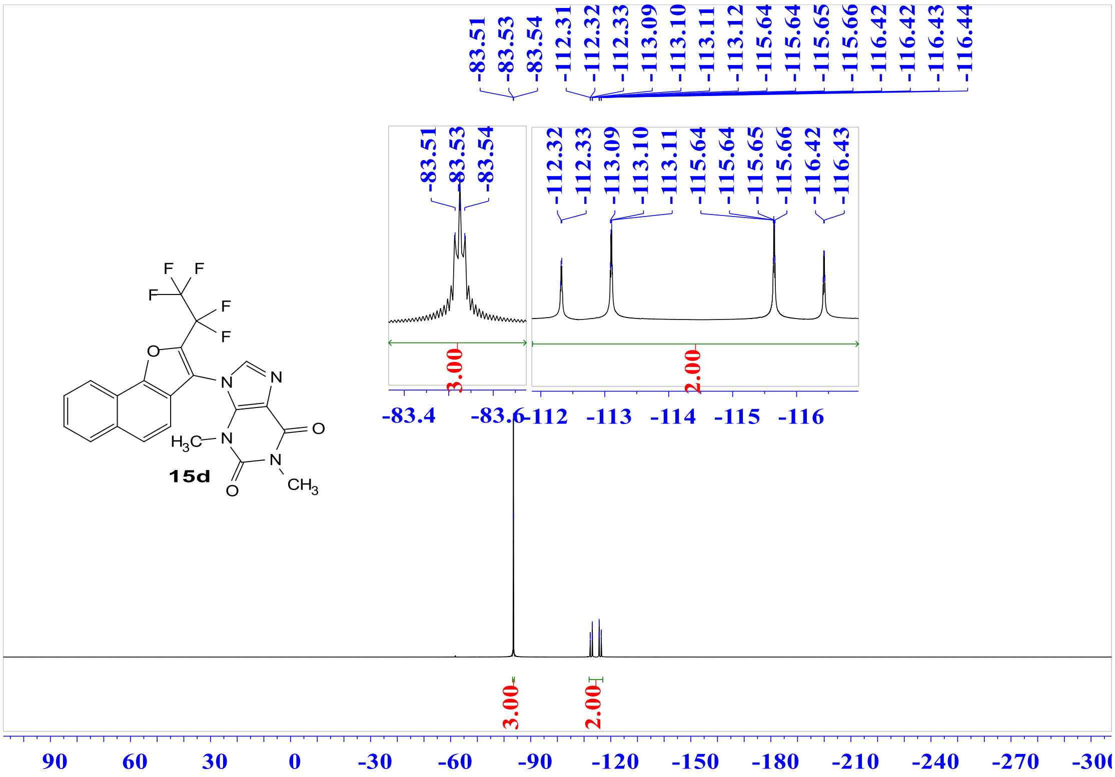

Figure S126. ^{13}C NMR spectrum of **15d**, related to **Scheme 2**.

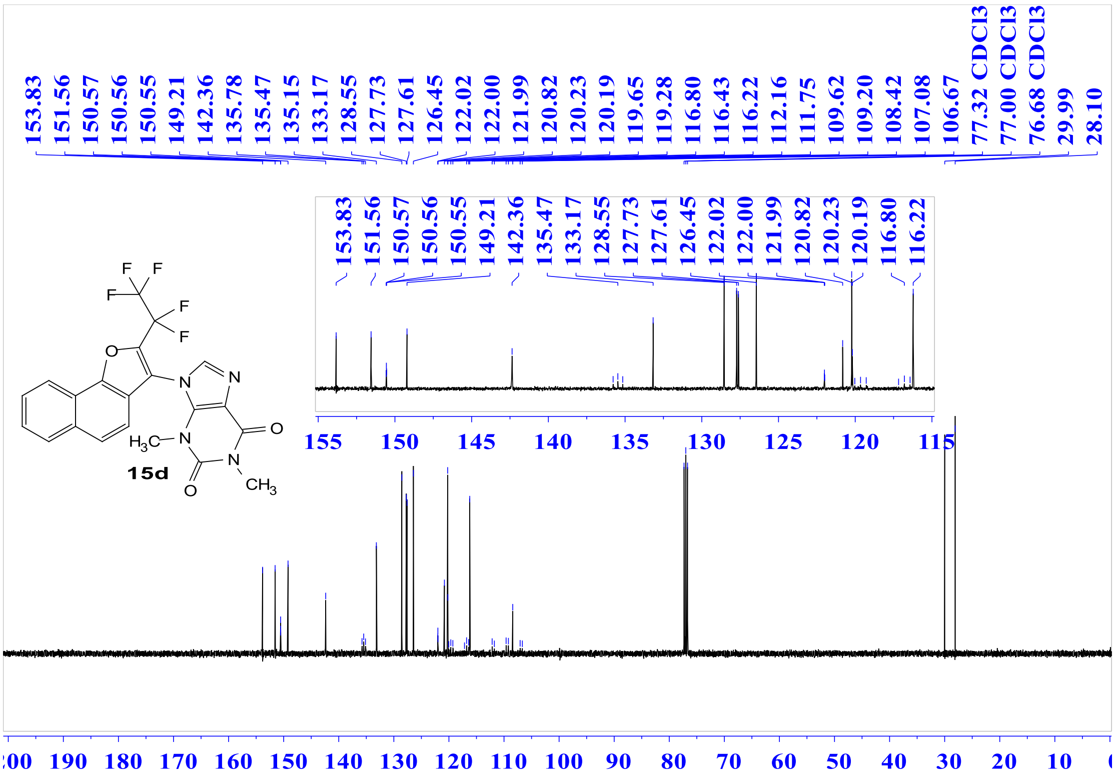

Figure S127. ^{1}H NMR spectrum of **15e**, related to **Scheme 2**.

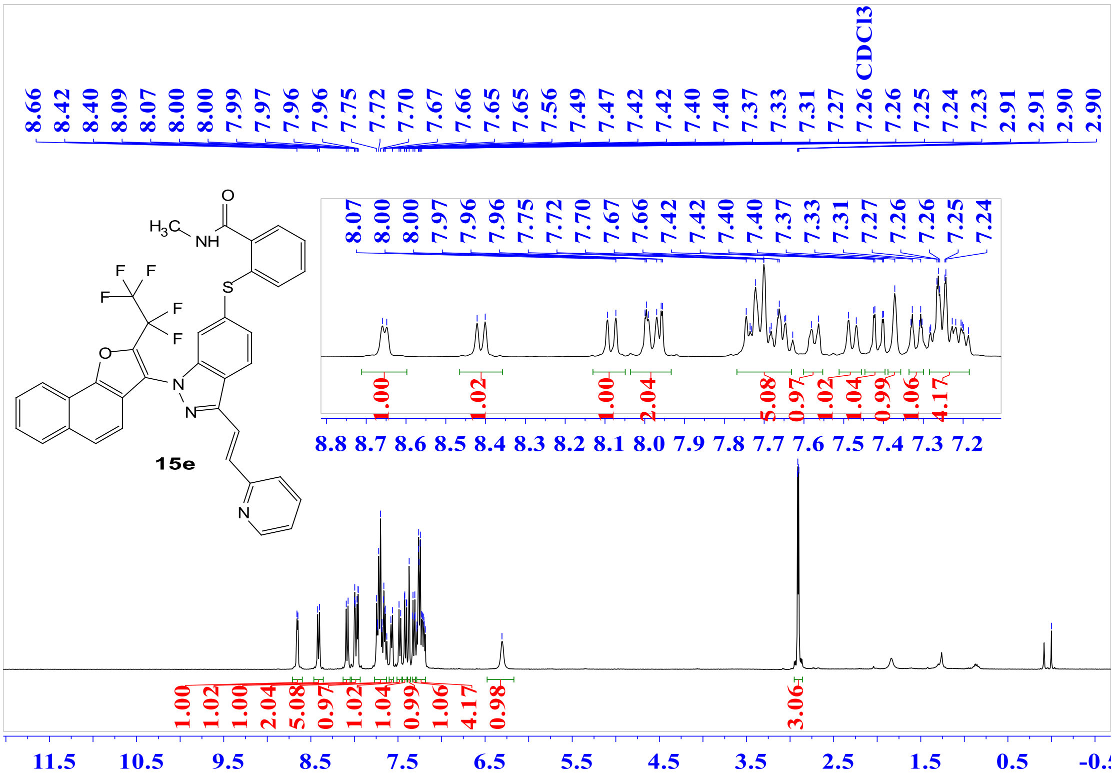

Figure S128. ^{19}F NMR spectrum of **15e**, related to **Scheme 2**.

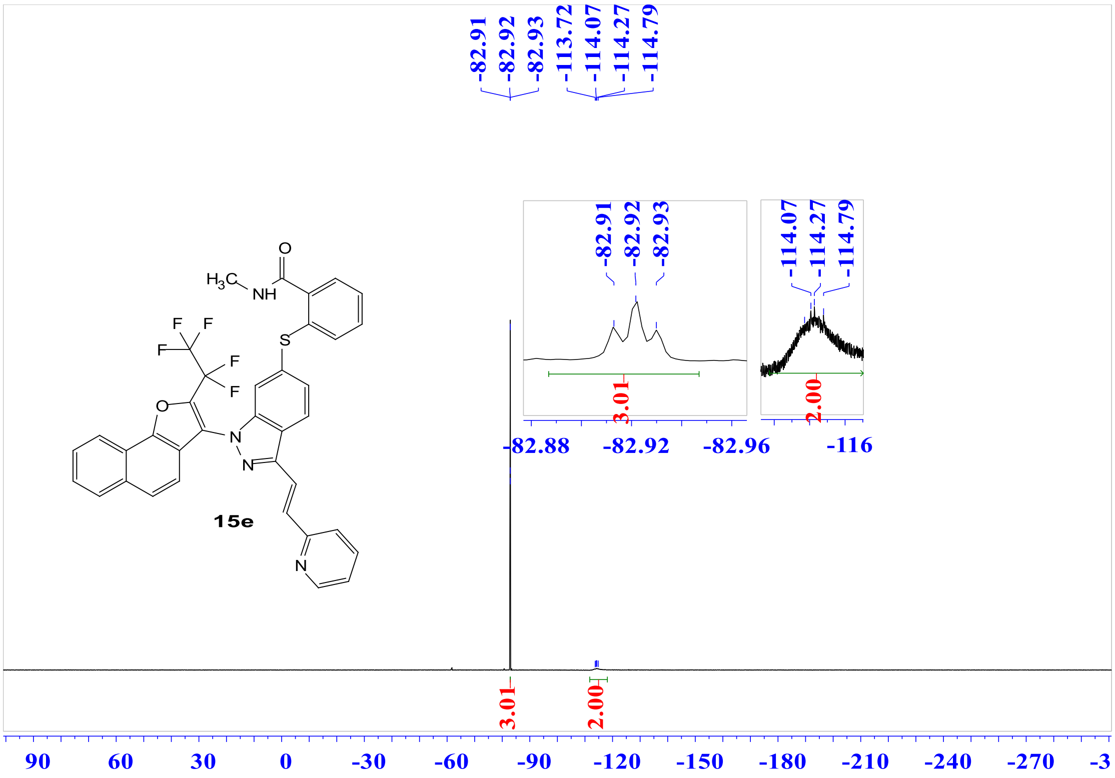

Figure S129. ^{13}C NMR spectrum of **15e**, related to **Scheme 2**.

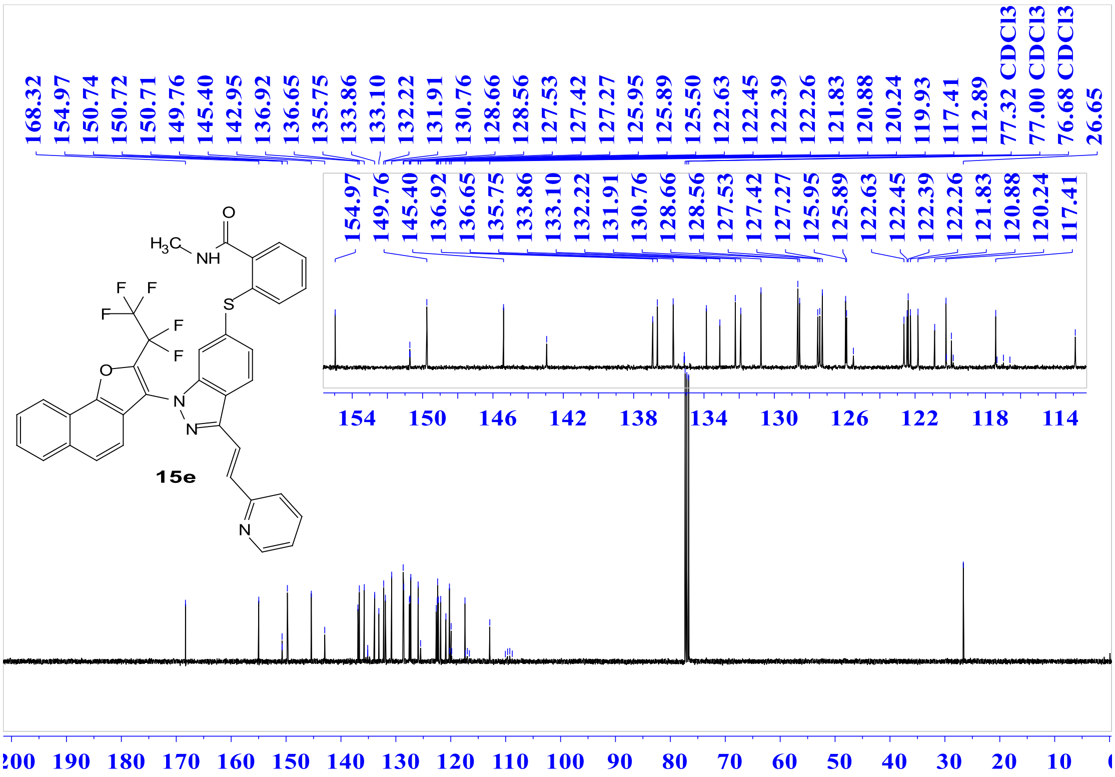

Figure S130. ^{1}H NMR spectrum of **15f-I**, **15f-II**, or **15f-III**, related to **Scheme 2**.

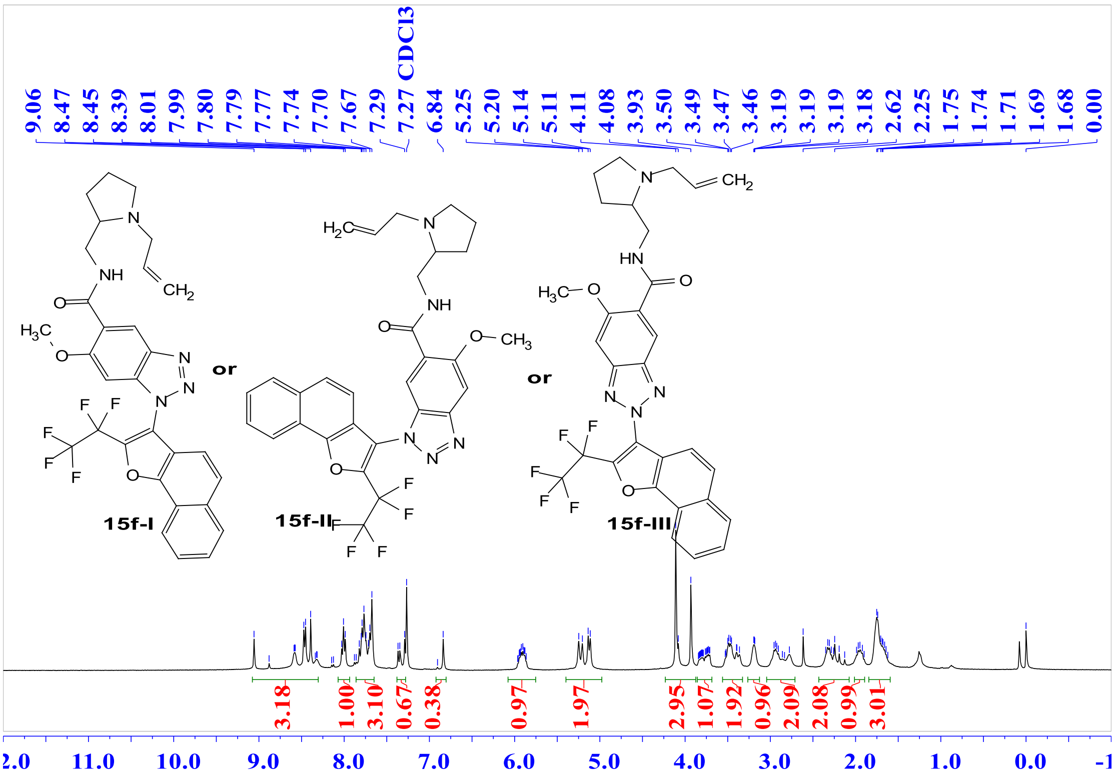

Figure S131. ^{19}F NMR spectrum of **15f-I**, **15f-II**, or **15f-III**, related to **Scheme 2**.

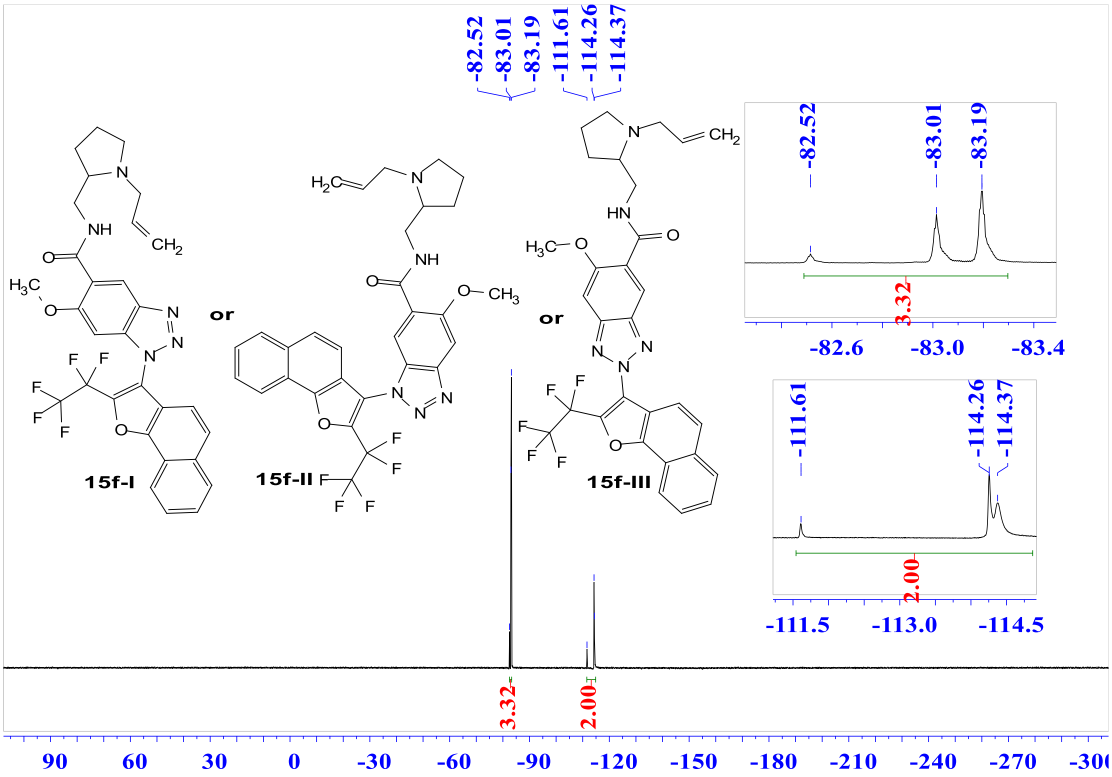

Figure S132. ^{13}C NMR spectrum of **15f-I**, **15f-II**, or **15f-III**, related to **Scheme 2**.

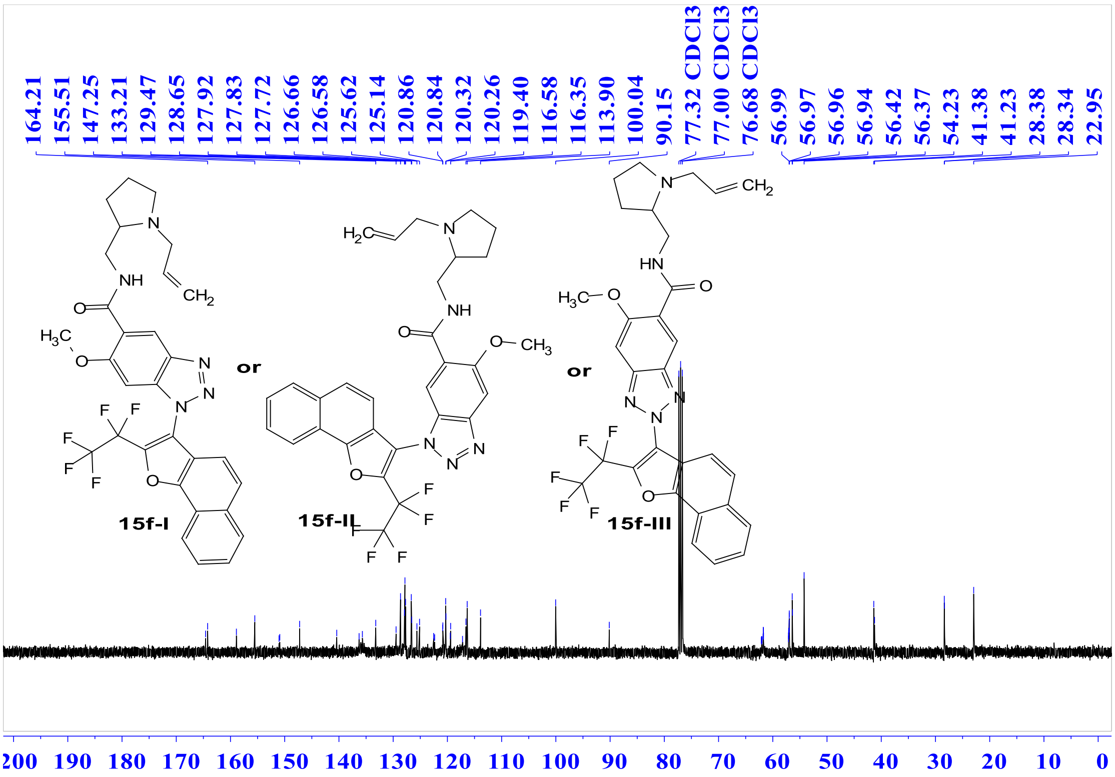

Figure S133. ^{1}H NMR spectrum of **15g**, related to **Scheme 2**.

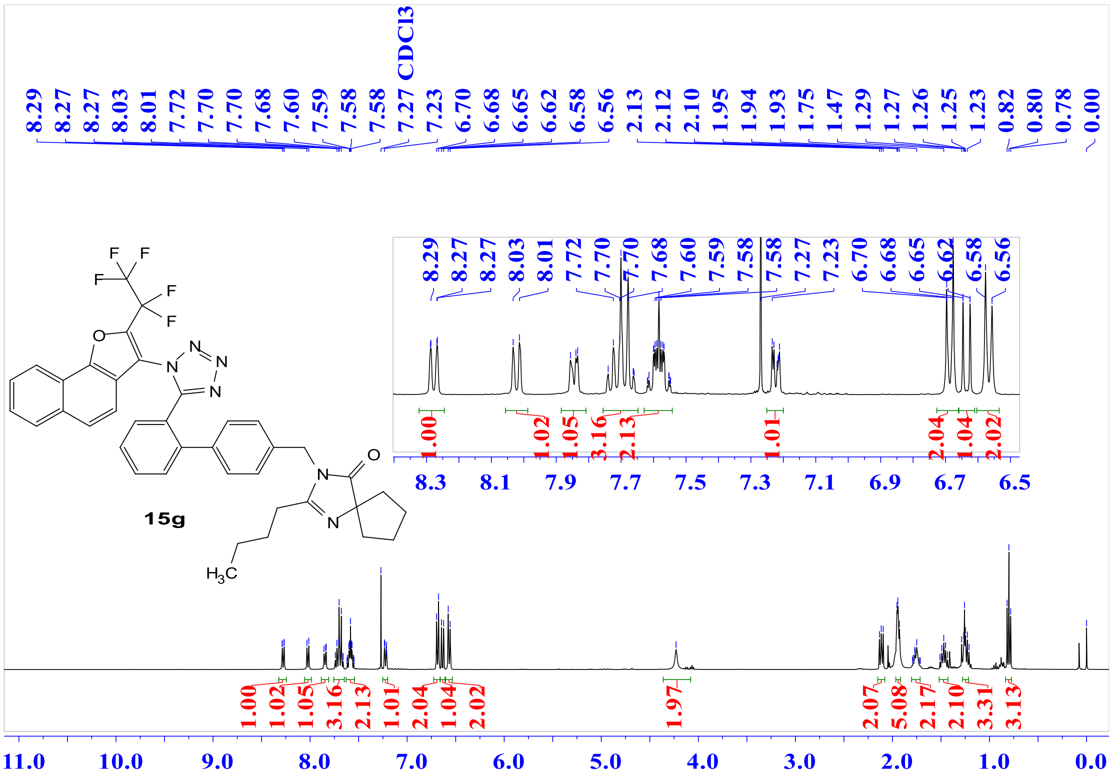

Figure S134. ^{19}F NMR spectrum of **15g**, related to **Scheme 2**.

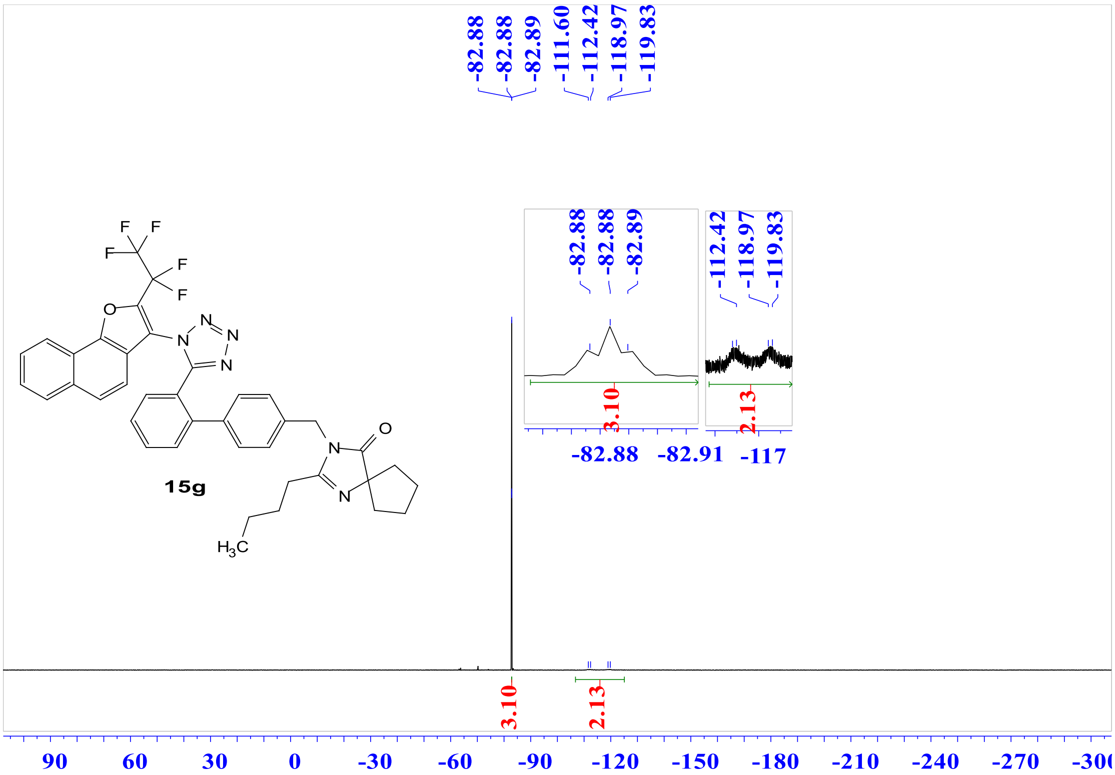

Figure S135. ^{13}C NMR spectrum of **15g**, related to **Scheme 2**.

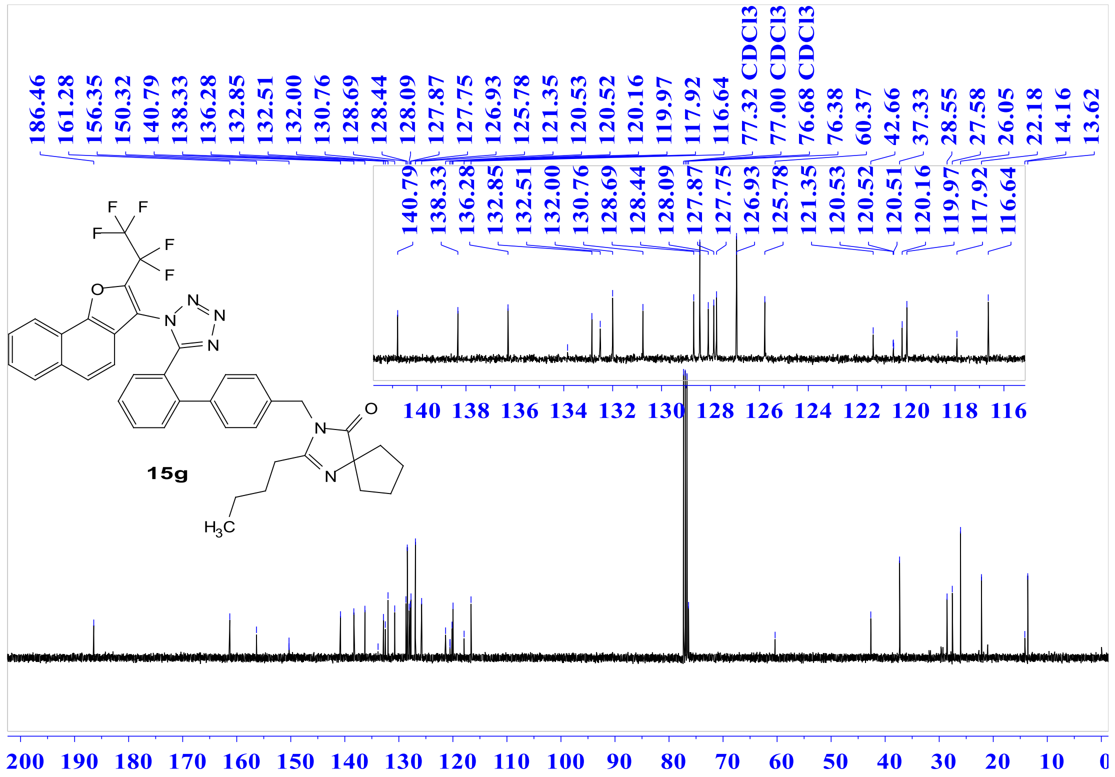

Figure S136. ^{1}H NMR spectrum of **16a**, related to **Scheme 3**.

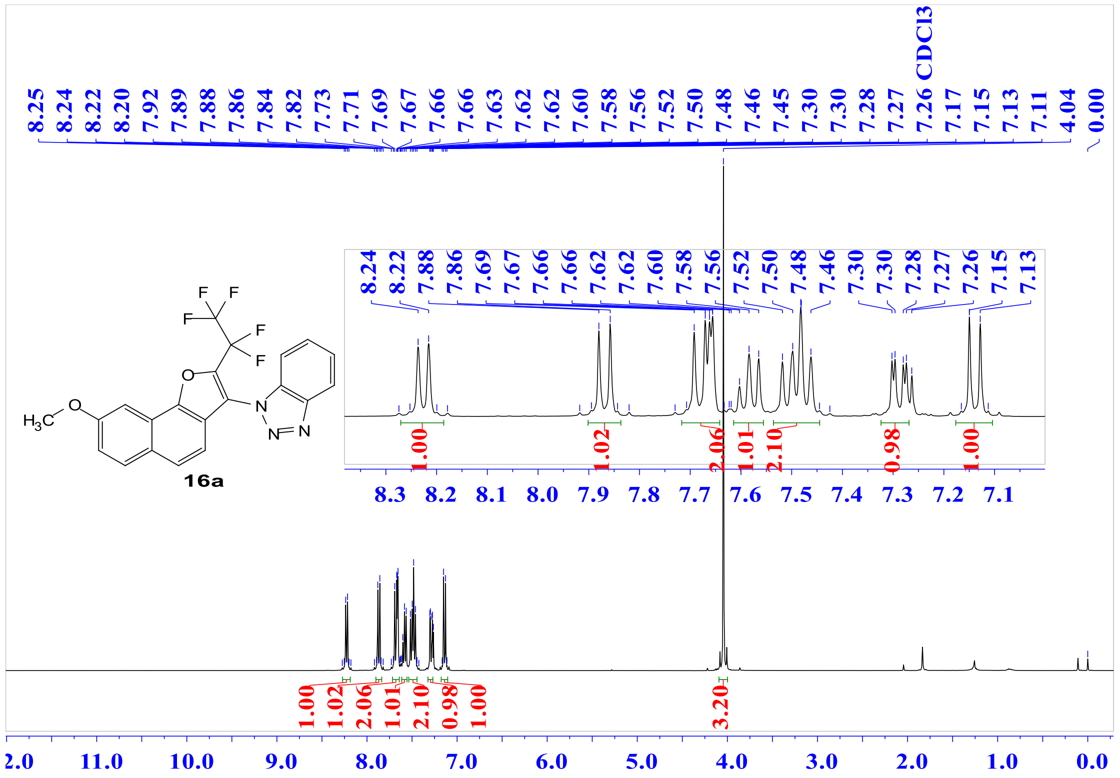

Figure S137. ^{19}F NMR spectrum of **16a**, related to **Scheme 3**.

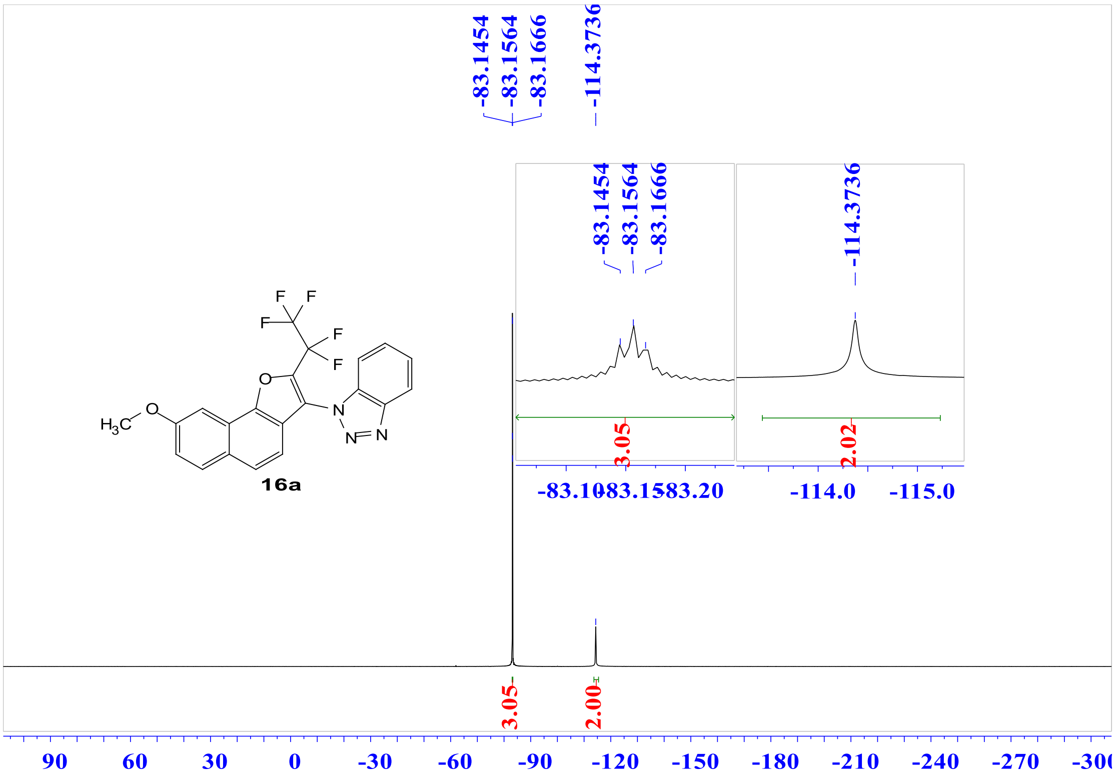

Figure S138. ^{13}C NMR spectrum of **16a**, related to **Scheme 3**.

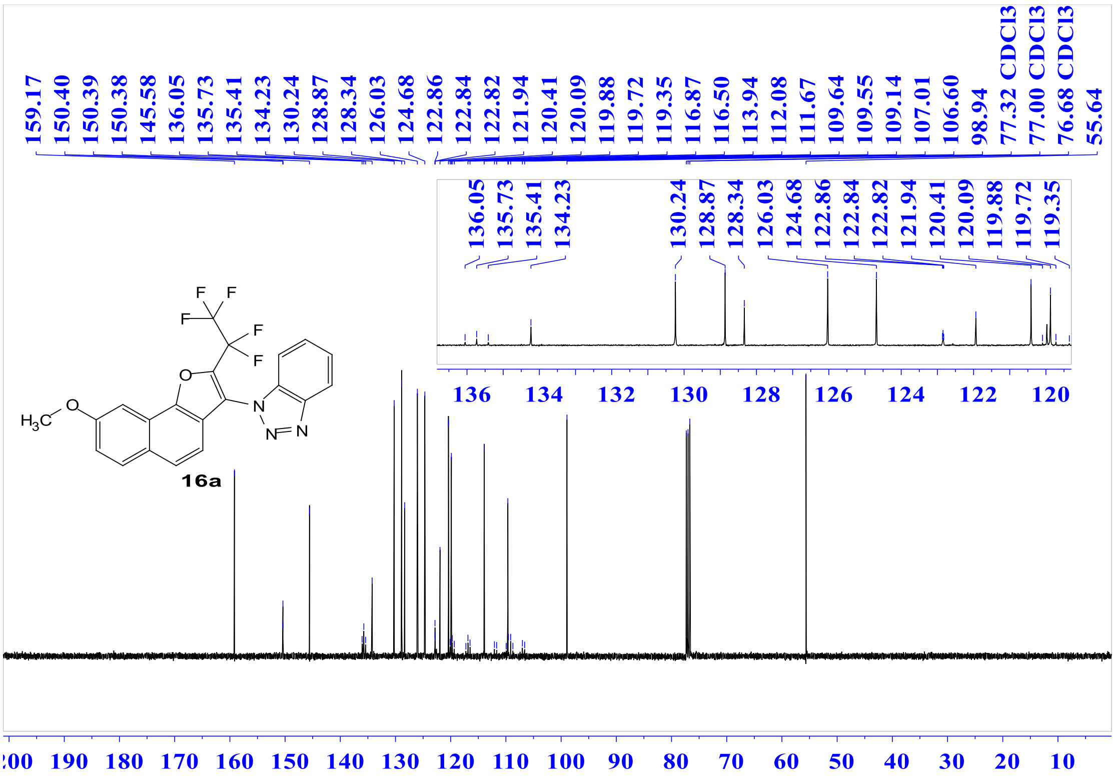

Figure S139. ^{1}H NMR spectrum of **16b**, related to **Scheme 3**.

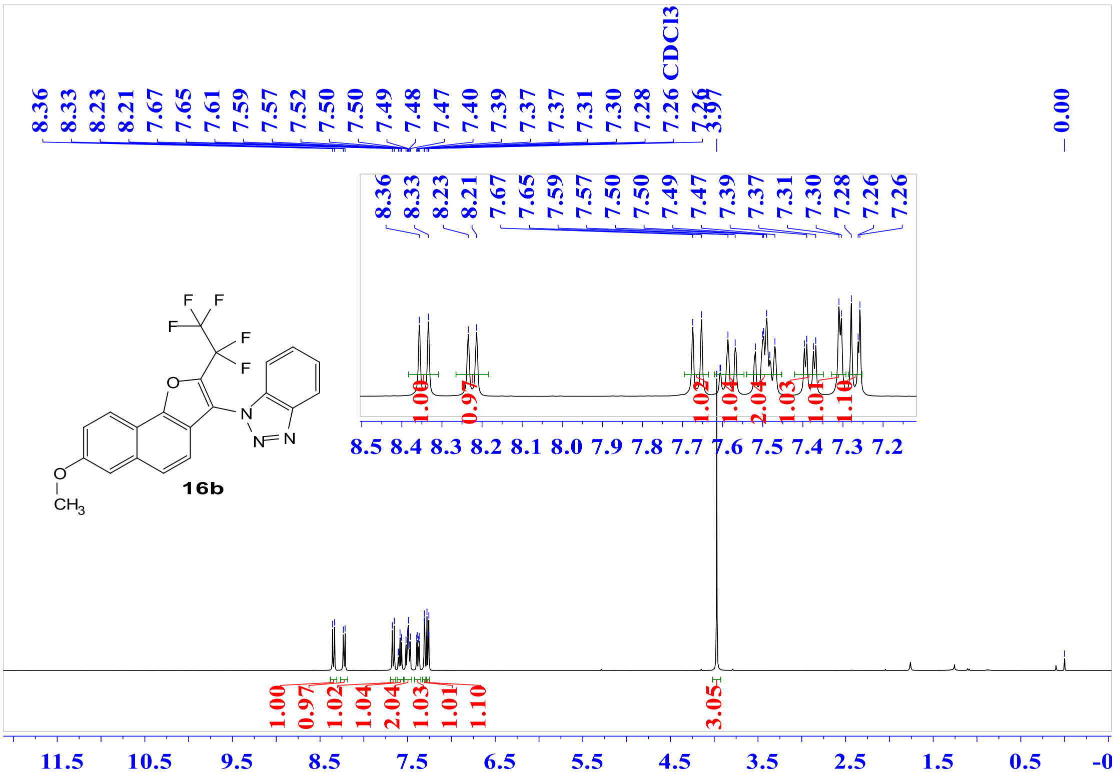

Figure S140. ^{19}F NMR spectrum of **16b**, related to **Scheme 3**.

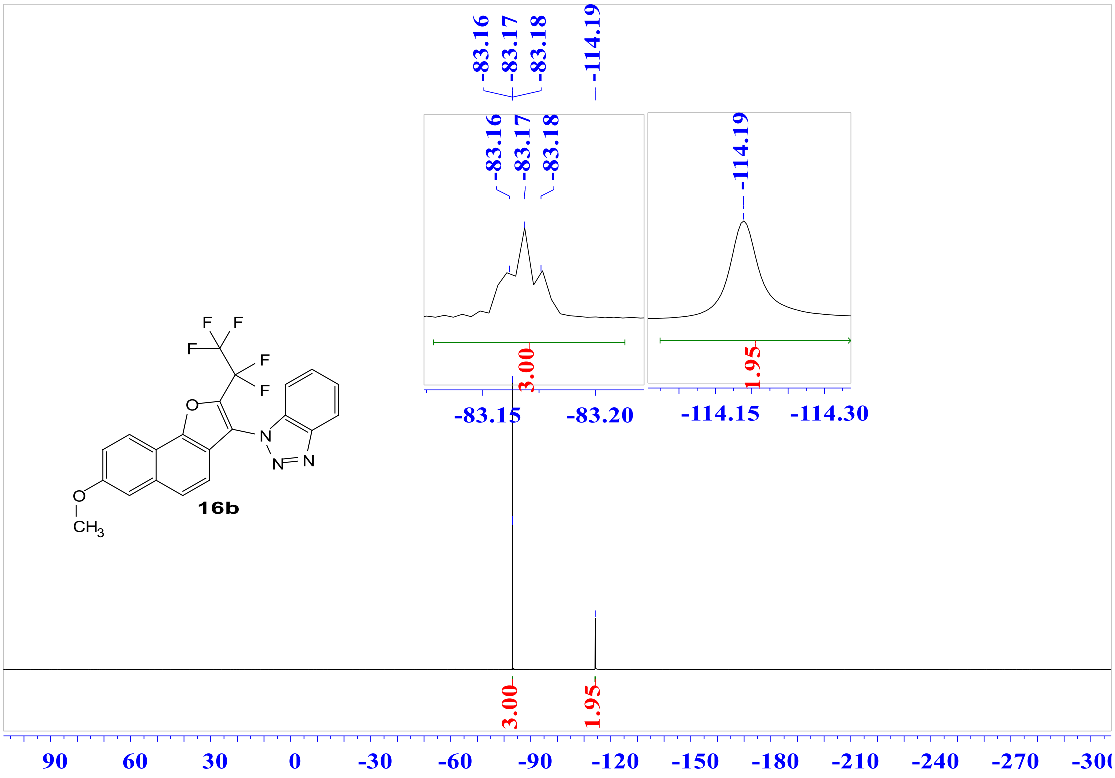

Figure S141. ^{13}C NMR spectrum of **16b**, related to **Scheme 3**.

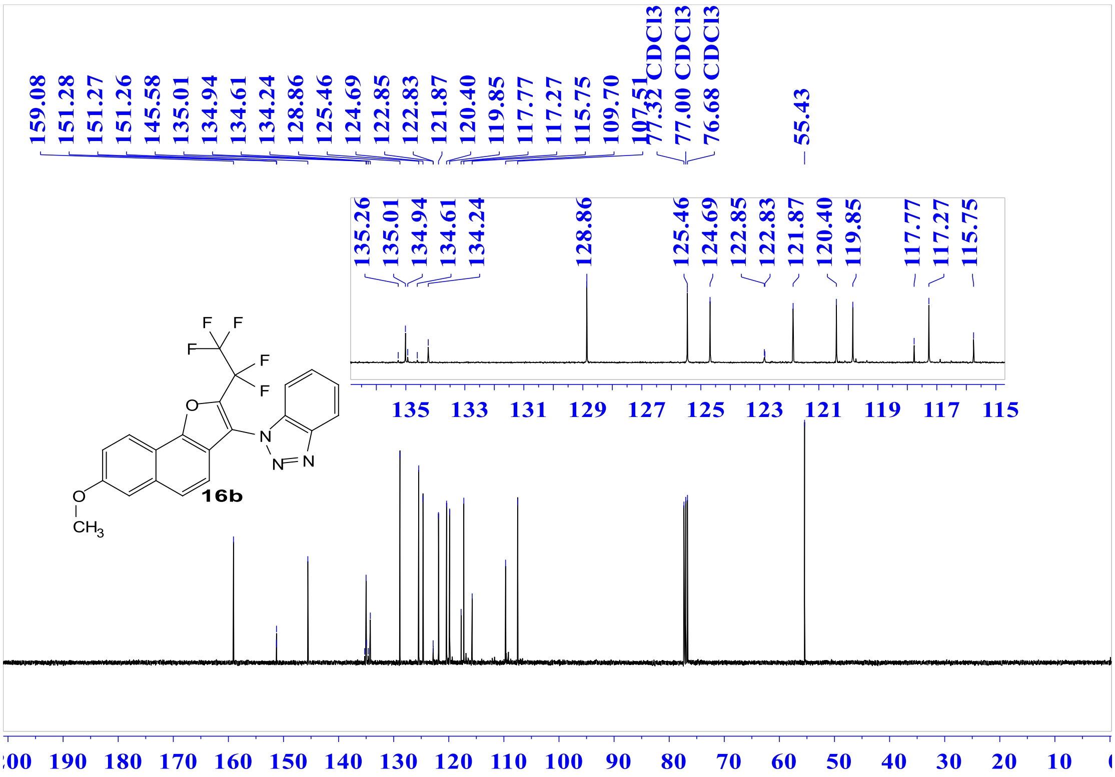

Figure S142. ^{1}H NMR spectrum of **16c**, related to **Scheme 3**.

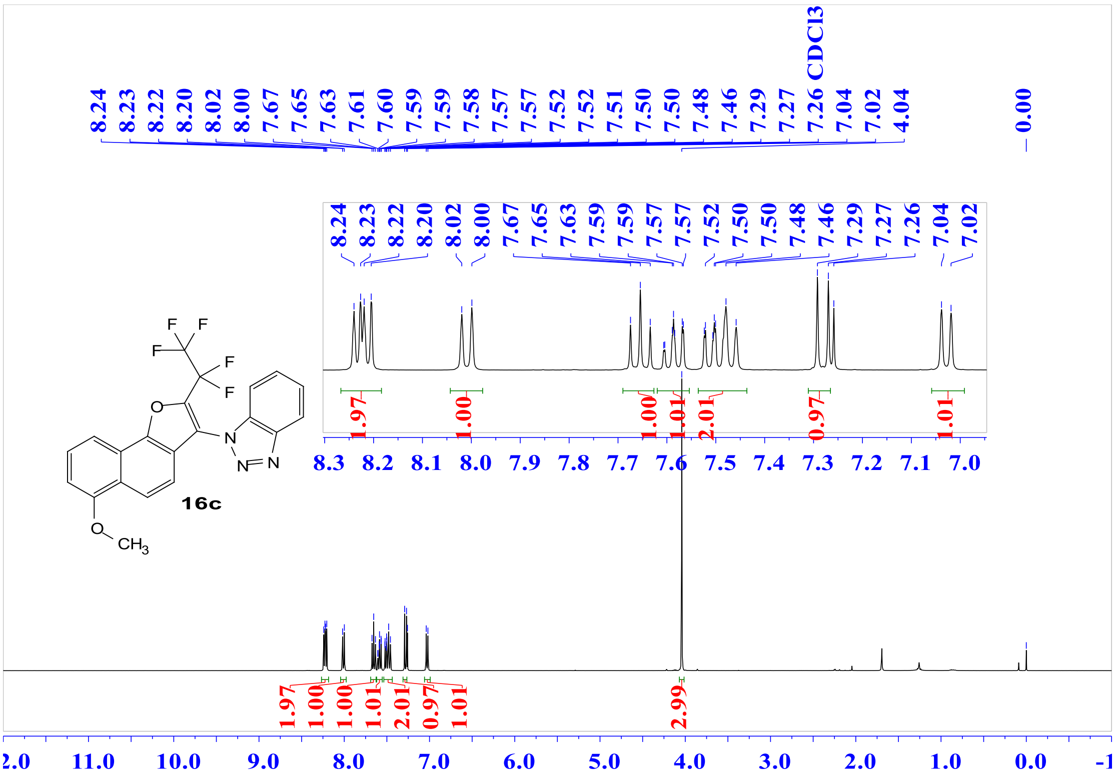

Figure S143. ^{19}F NMR spectrum of **16c**, related to **Scheme 3**.

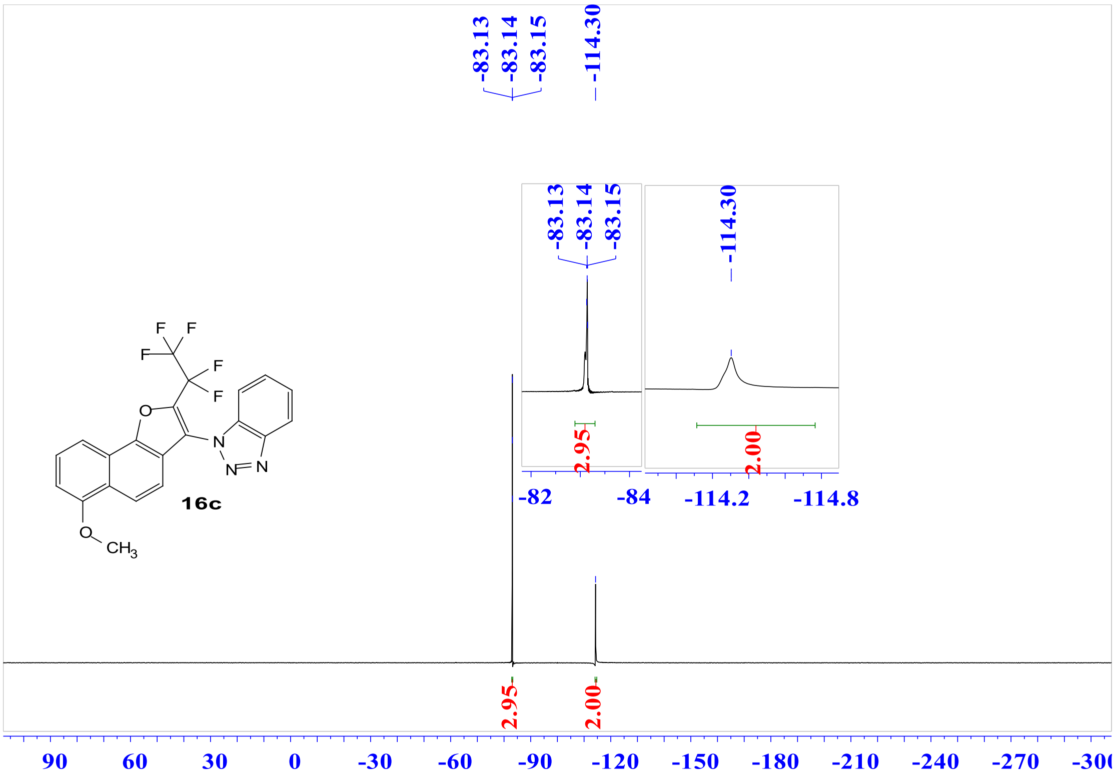

Figure S144. ^{13}C NMR spectrum of **16c**, related to **Scheme 3**.

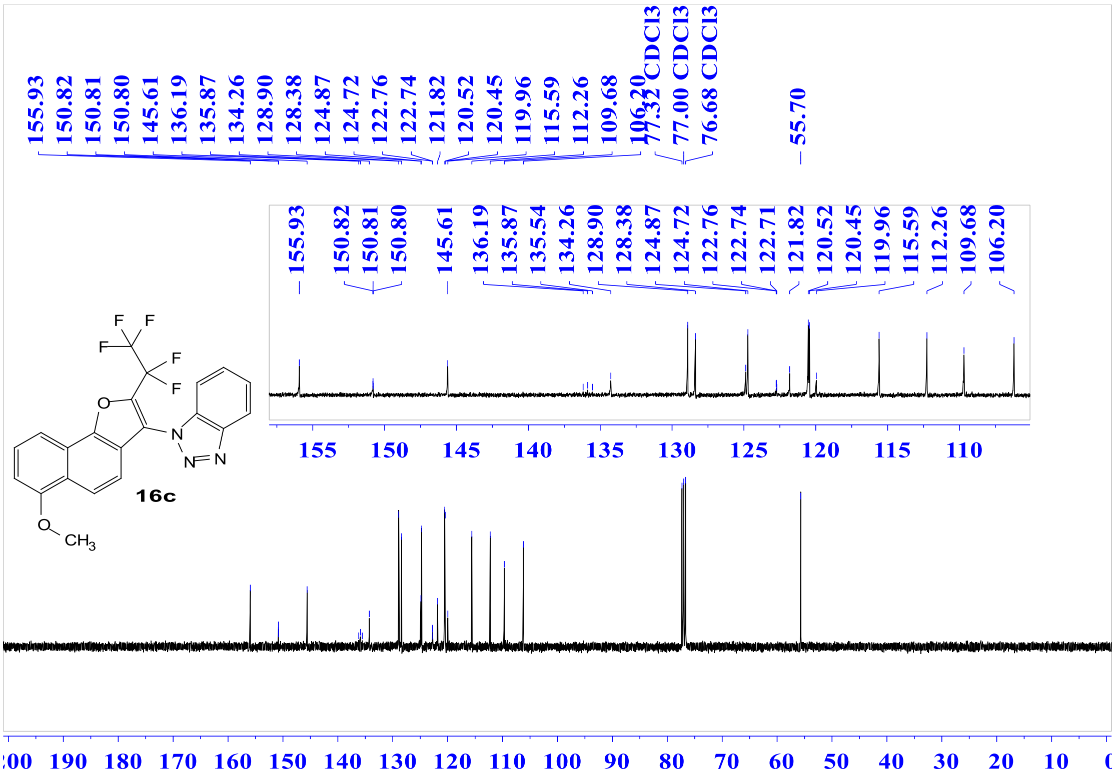

Figure S145. ^{1}H NMR spectrum of **16d**, related to **Scheme 3**.

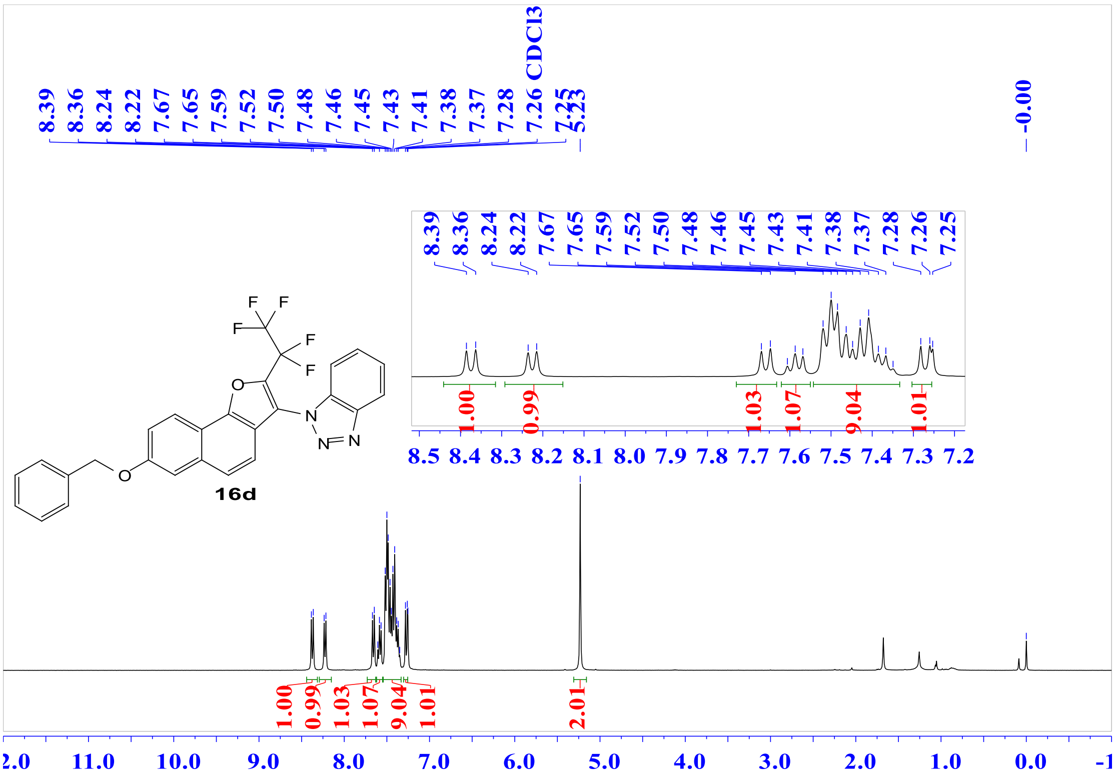

Figure S146. ^{19}F NMR spectrum of **16d**, related to **Scheme 3**.

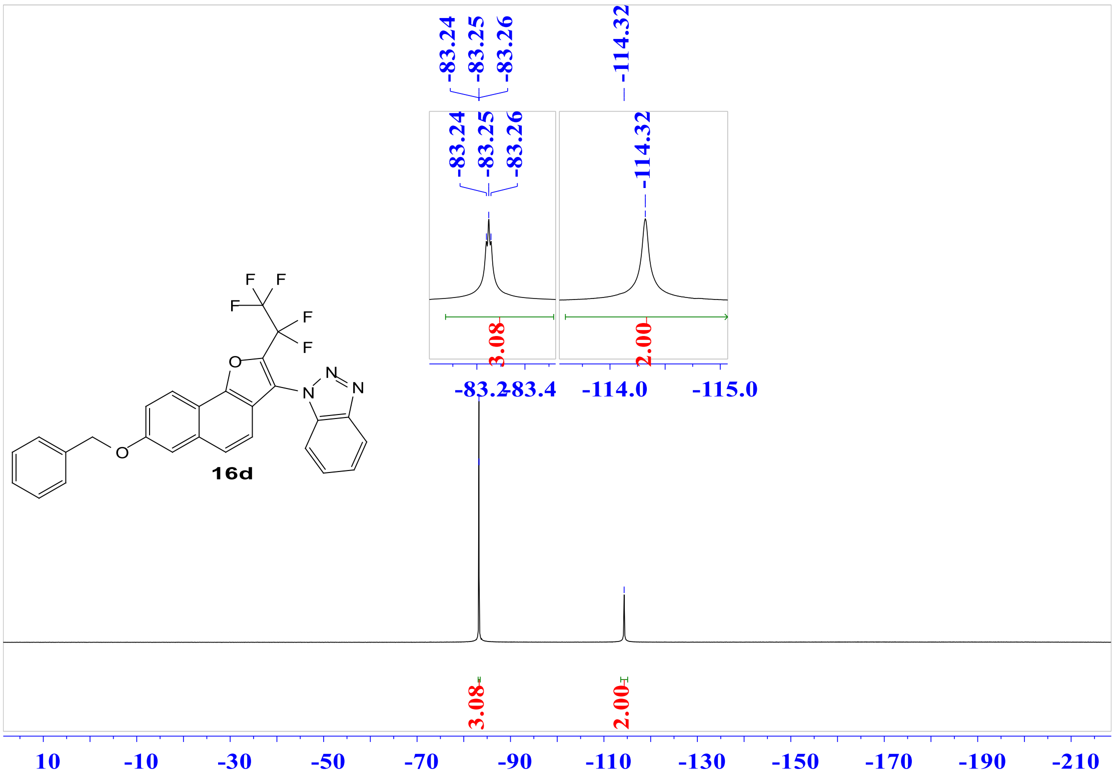

Figure S147. ^{13}C NMR spectrum of **16d**, related to **Scheme 3**.

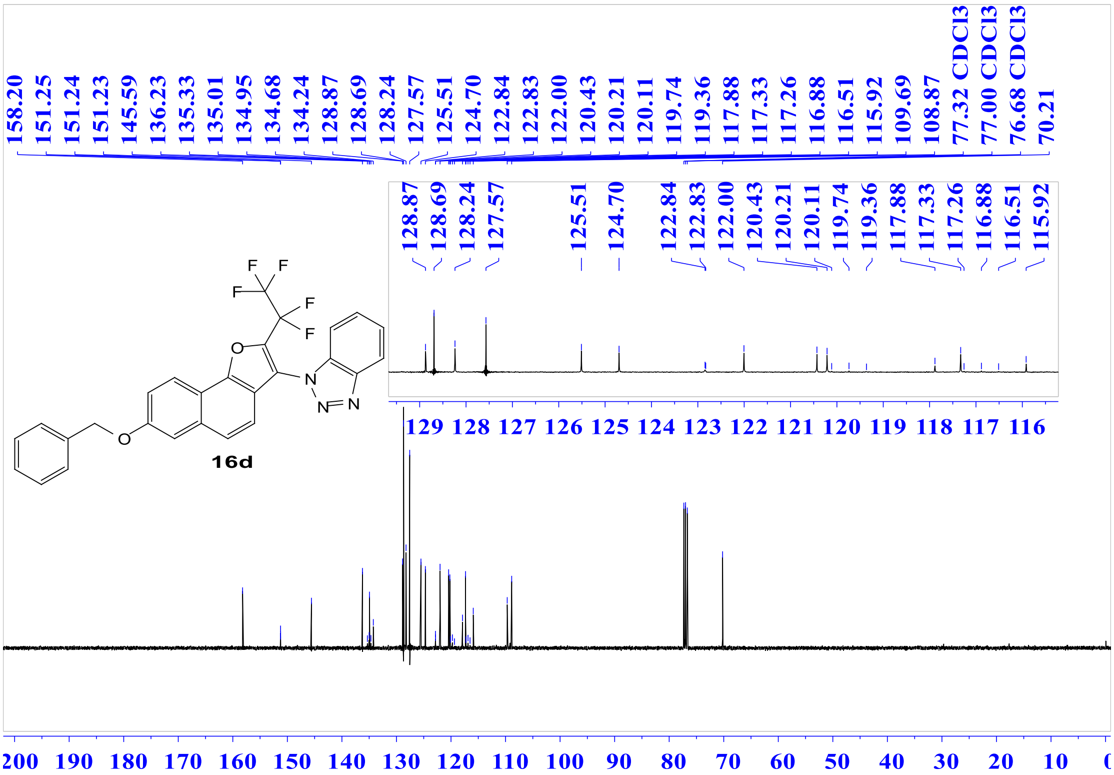

Figure S148. ^{1}H NMR spectrum of **16e**, related to **Scheme 3**.

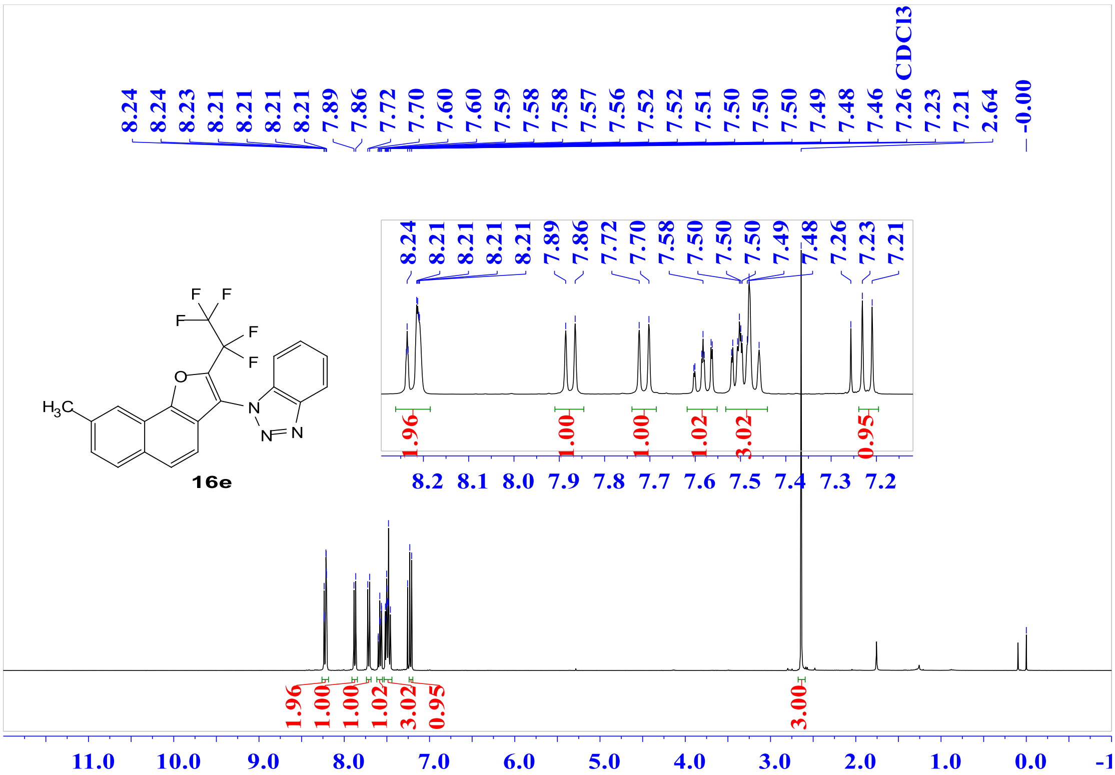

Figure S149. ^{19}F NMR spectrum of **16e**, related to **Scheme 3**.

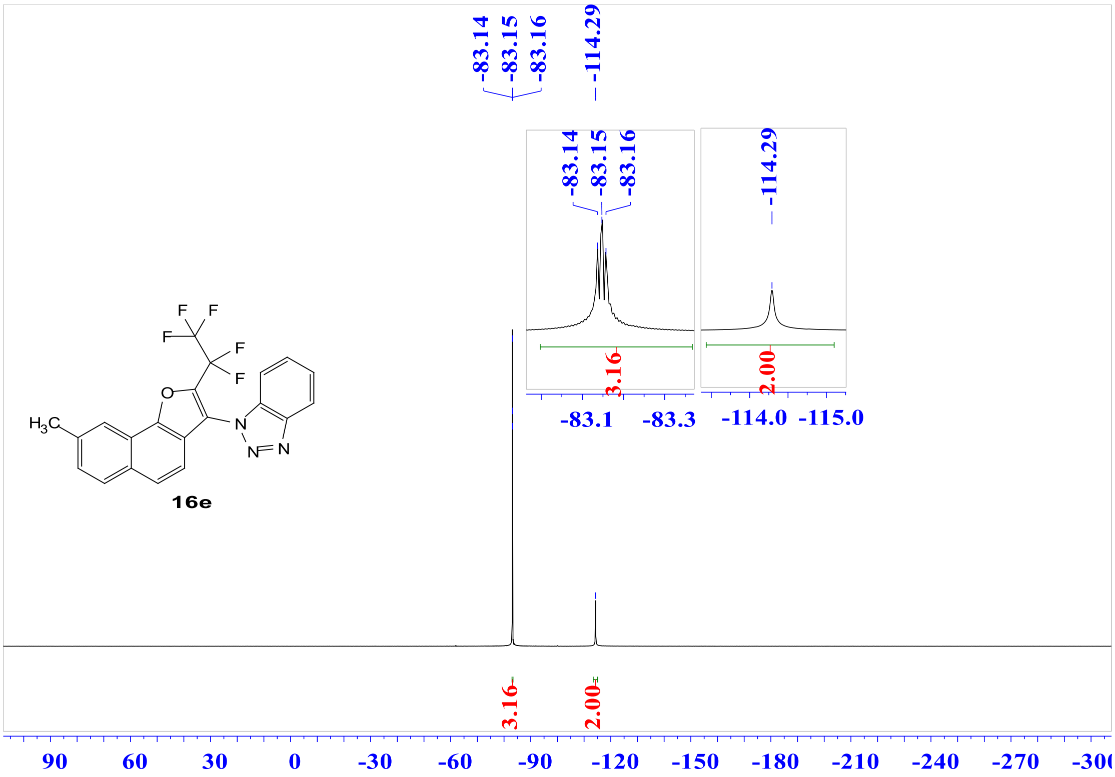

Figure S150. ^{13}C NMR spectrum of **16e**, related to **Scheme 3**.

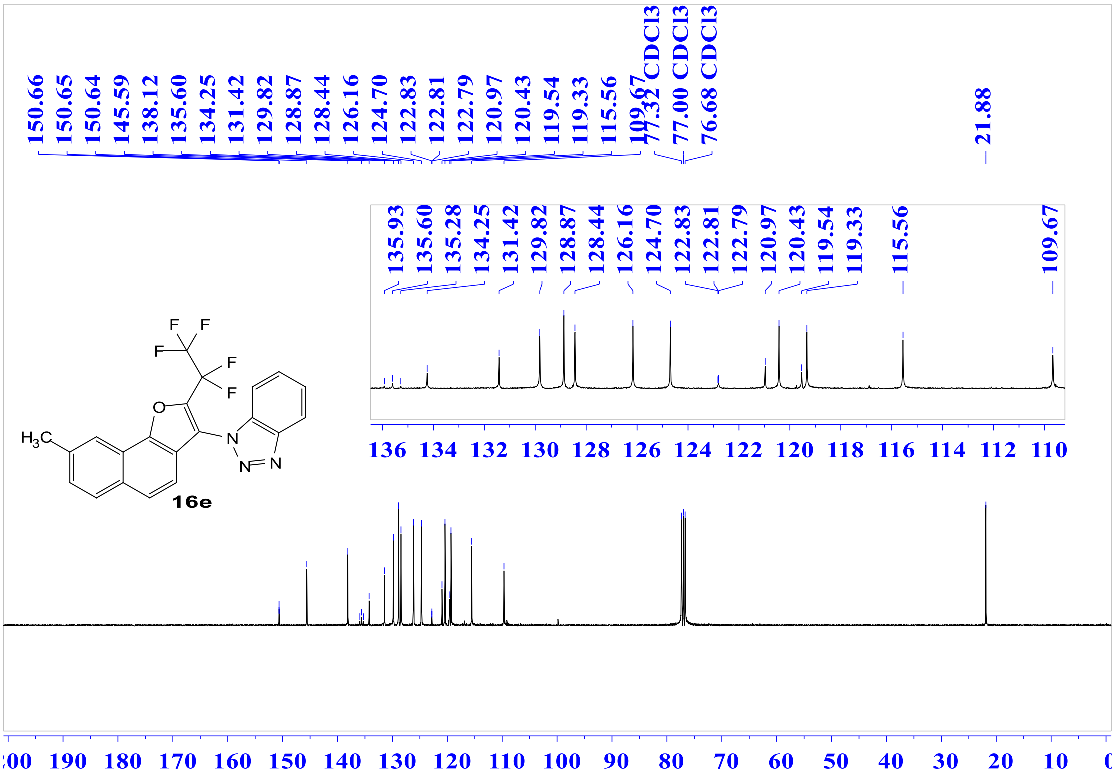

Figure S151. ^{1}H NMR spectrum of **16f**, related to **Scheme 3**.

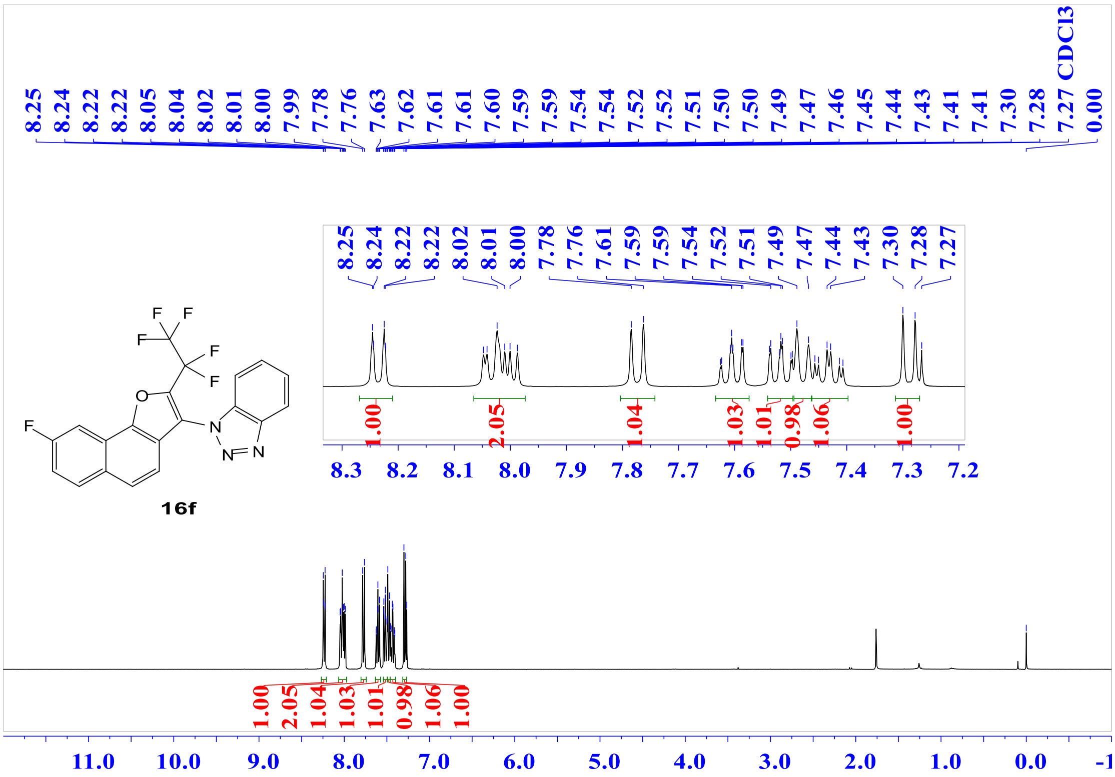

Figure S152. ^{19}F NMR spectrum of **16f**, related to **Scheme 3**.

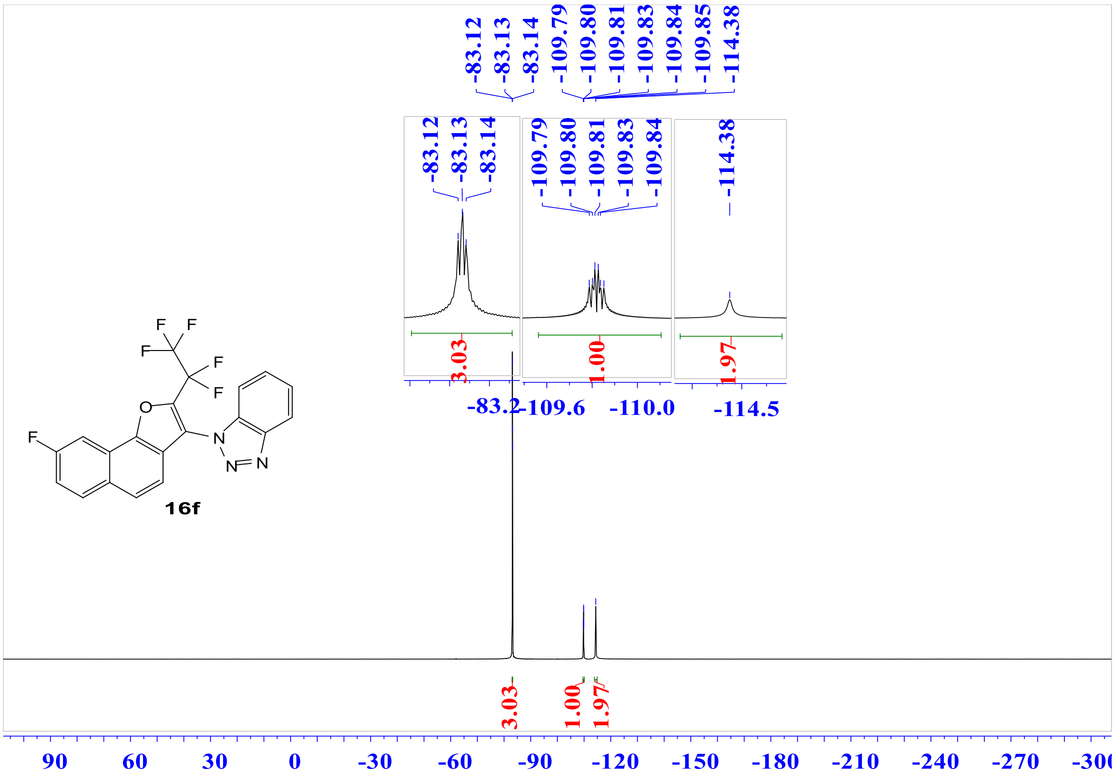

Figure S153. ^{13}C NMR spectrum of **16f**, related to **Scheme 3**.

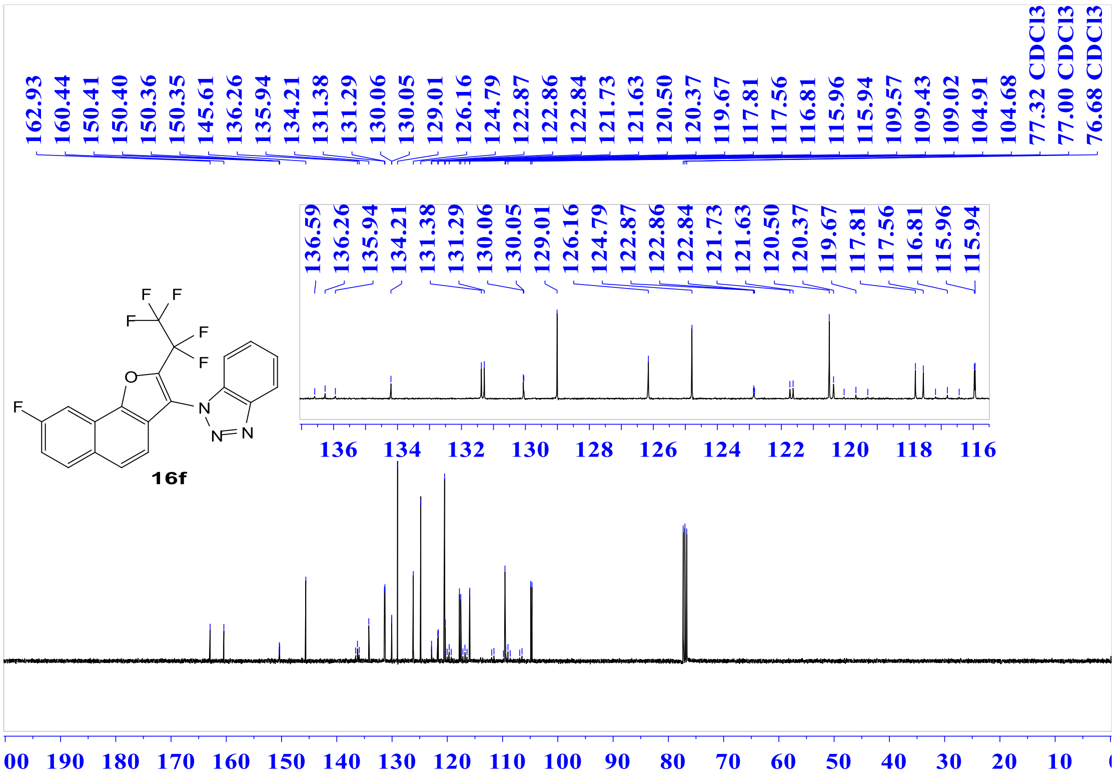

Figure S154. ^{1}H NMR spectrum of **16g**, related to **Scheme 3**.

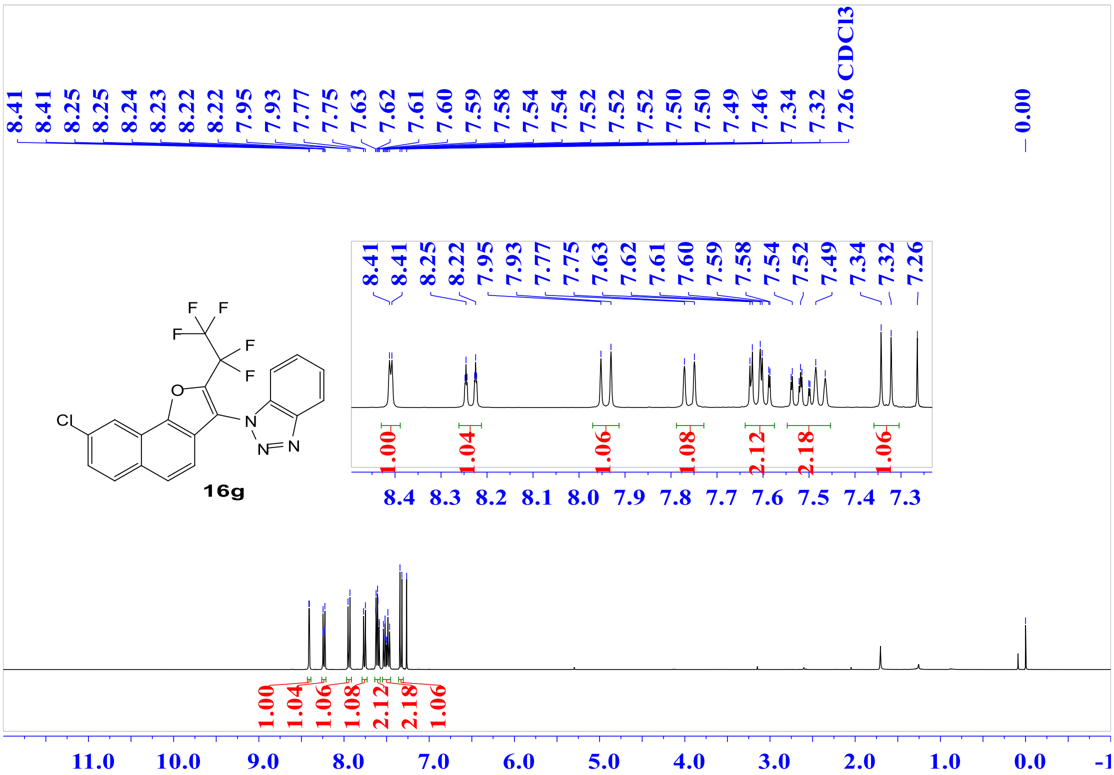

Figure S155. ^{19}F NMR spectrum of **16g**, related to **Scheme 3**.

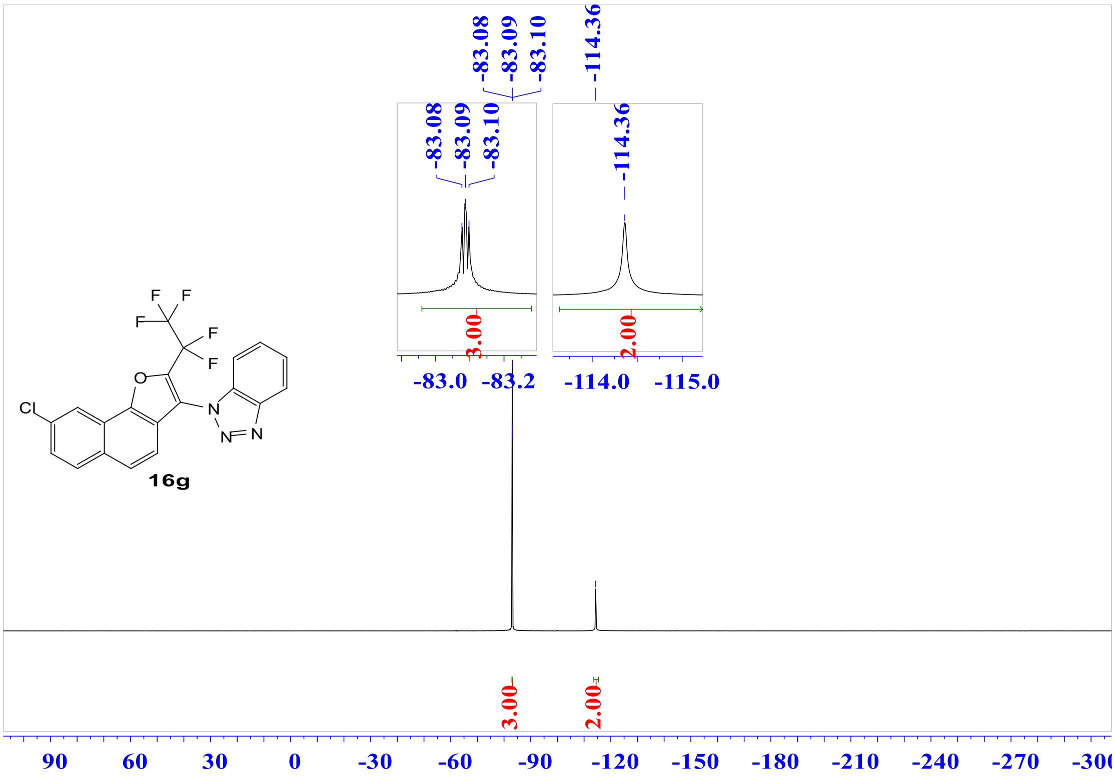

Figure S156. ^{13}C NMR spectrum of **16g**, related to **Scheme 3**.

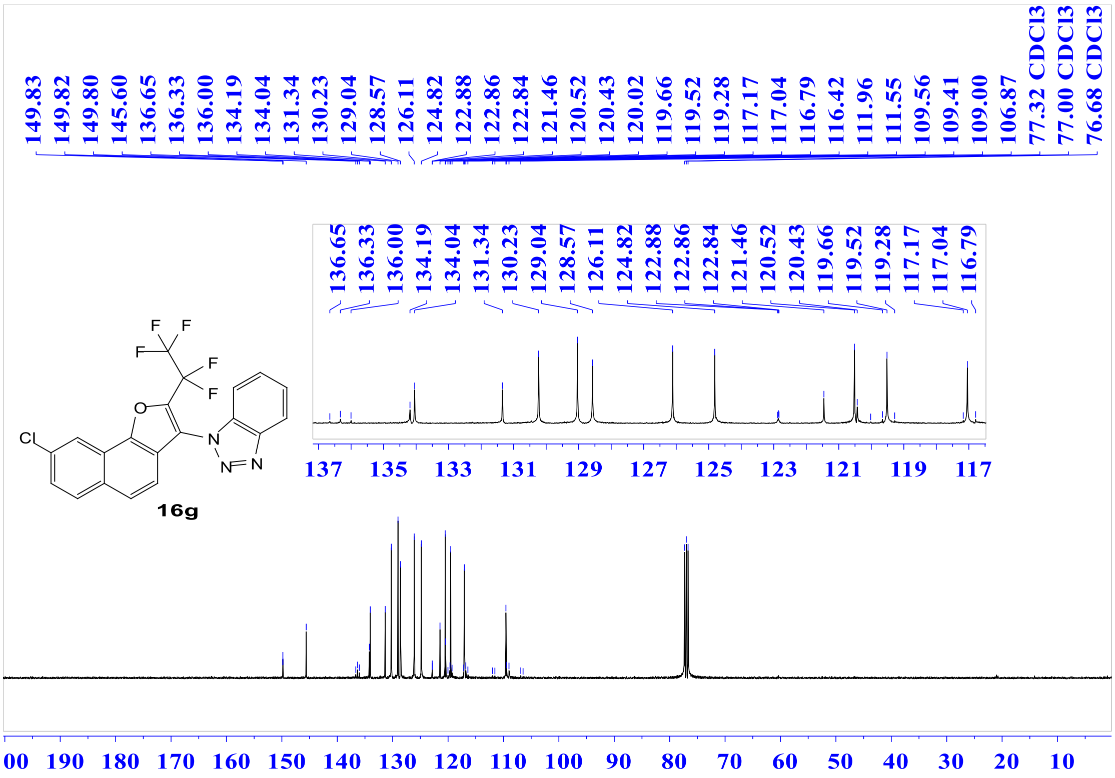

Figure S157. ^{1}H NMR spectrum of **16g**, related to **Scheme 3**.

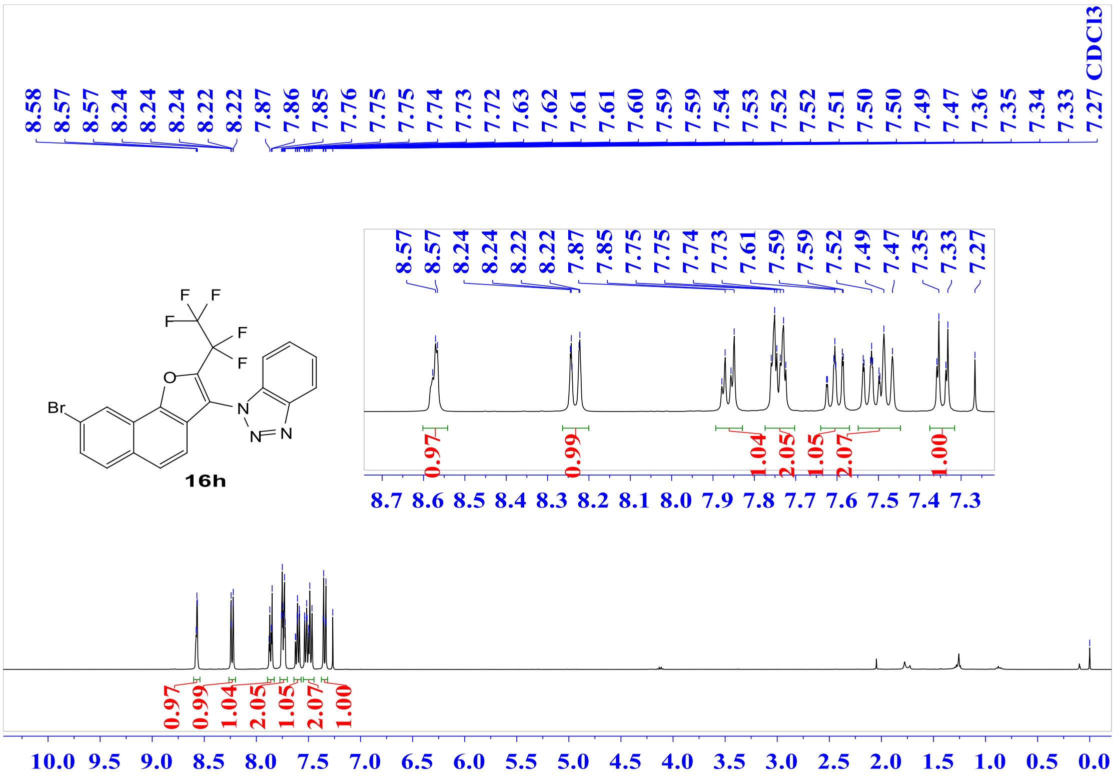

Figure S158. ^{19}F NMR spectrum of **16h**, related to **Scheme 3**.

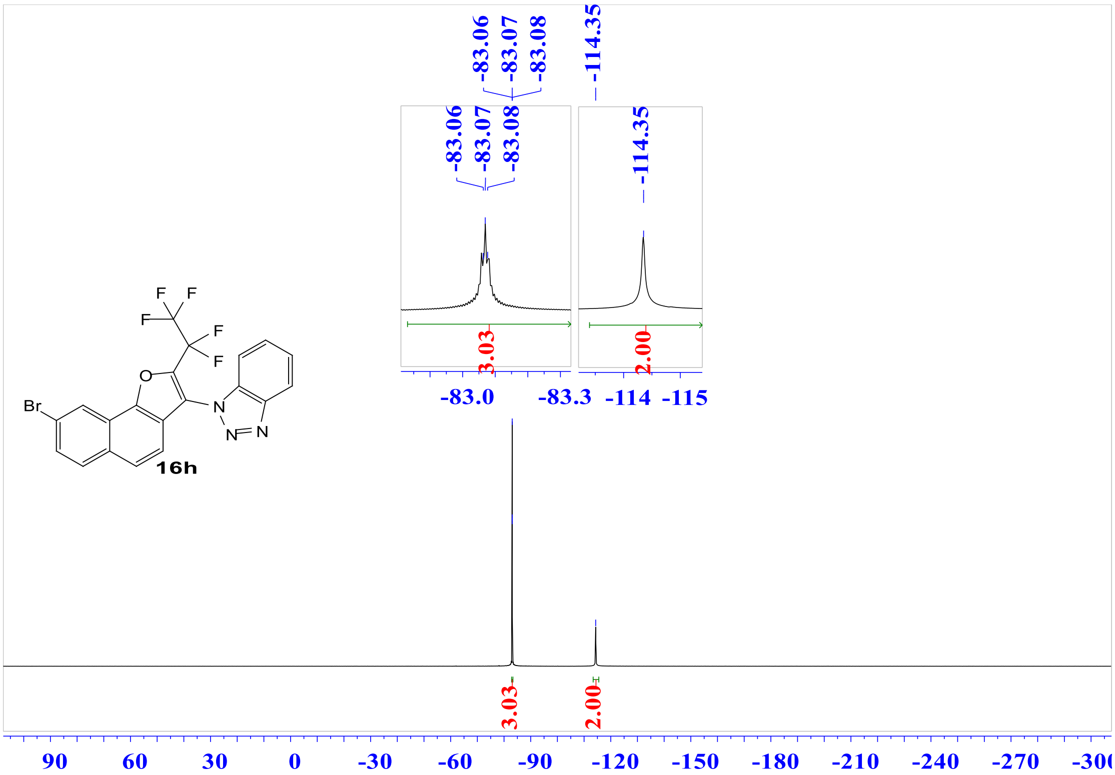

Figure S159. ^{13}C NMR spectrum of **16h**, related to **Scheme 3**.

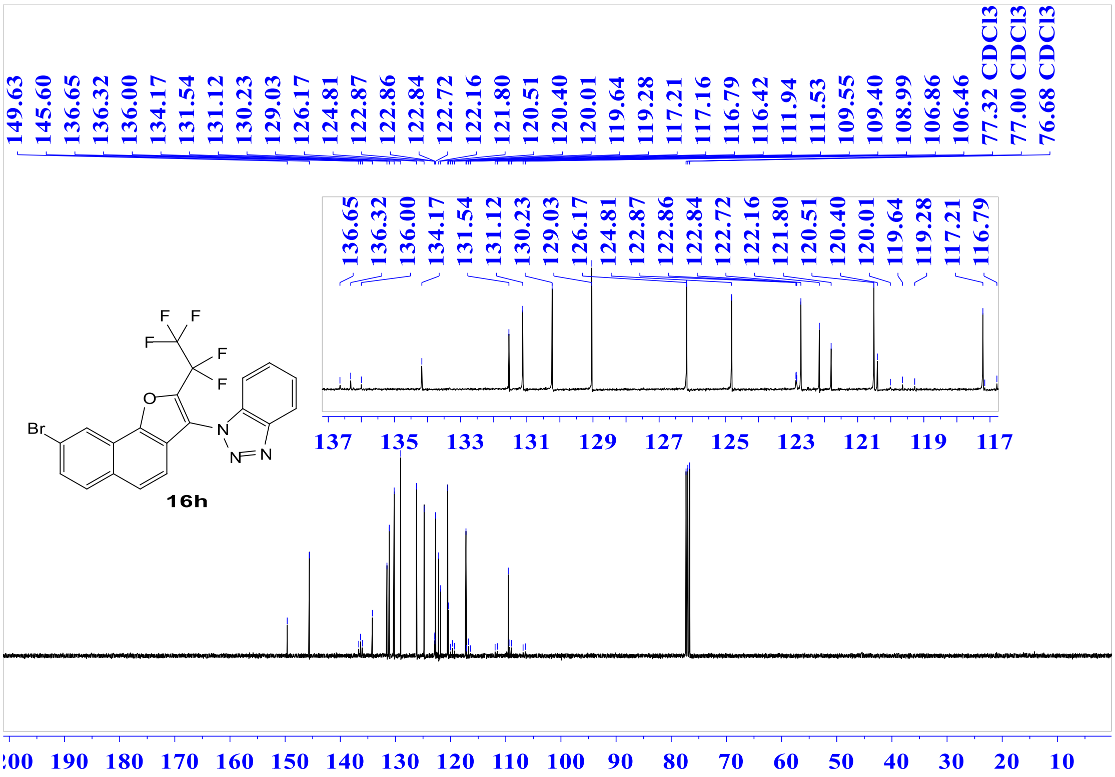

Figure S160. ^{1}H NMR spectrum of **16j**, related to **Scheme 3**.

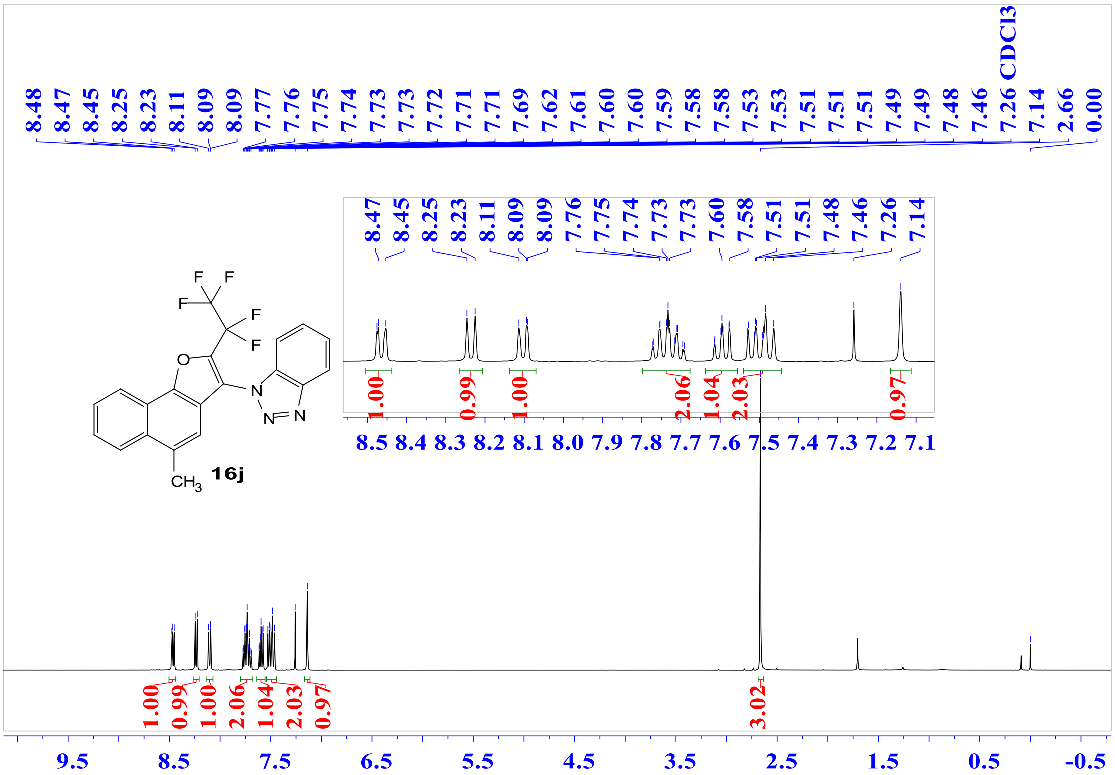

Figure S161. ¹⁹F NMR spectrum of **16j**, related to **Scheme 3**.

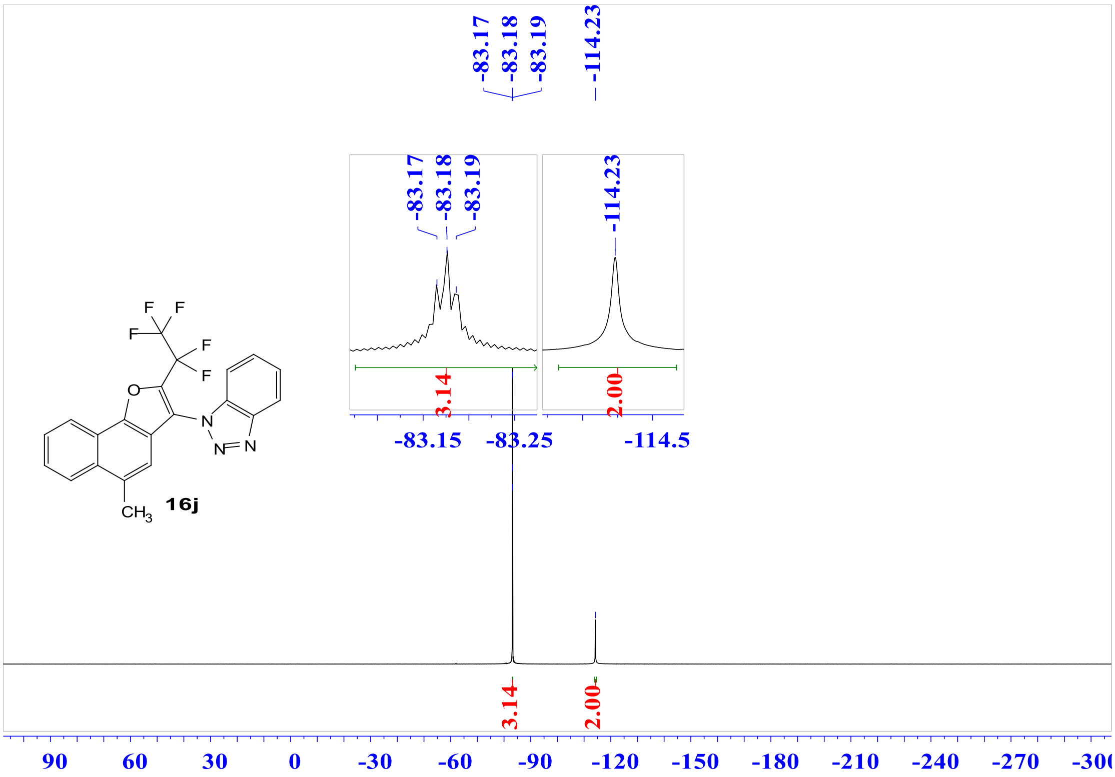

Figure S162. ¹³C NMR spectrum of **16j**, related to **Scheme 3**.

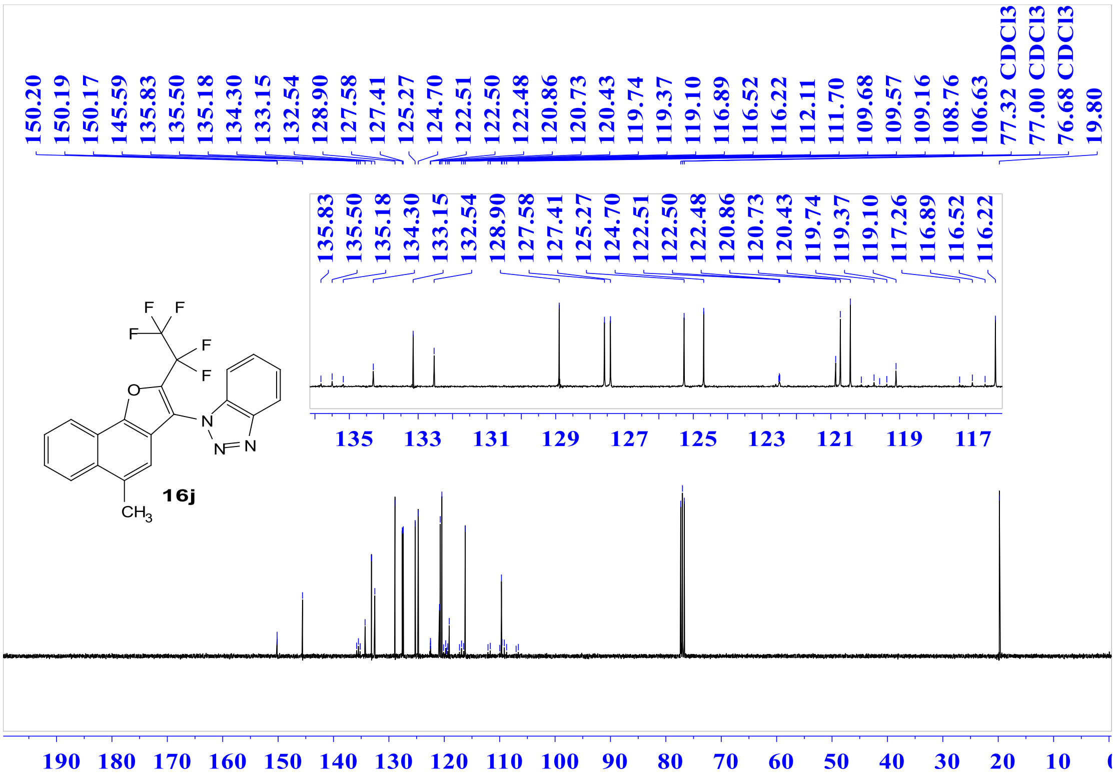

Figure S163. ^{1}H NMR spectrum of **16k**, related to **Scheme 3**.

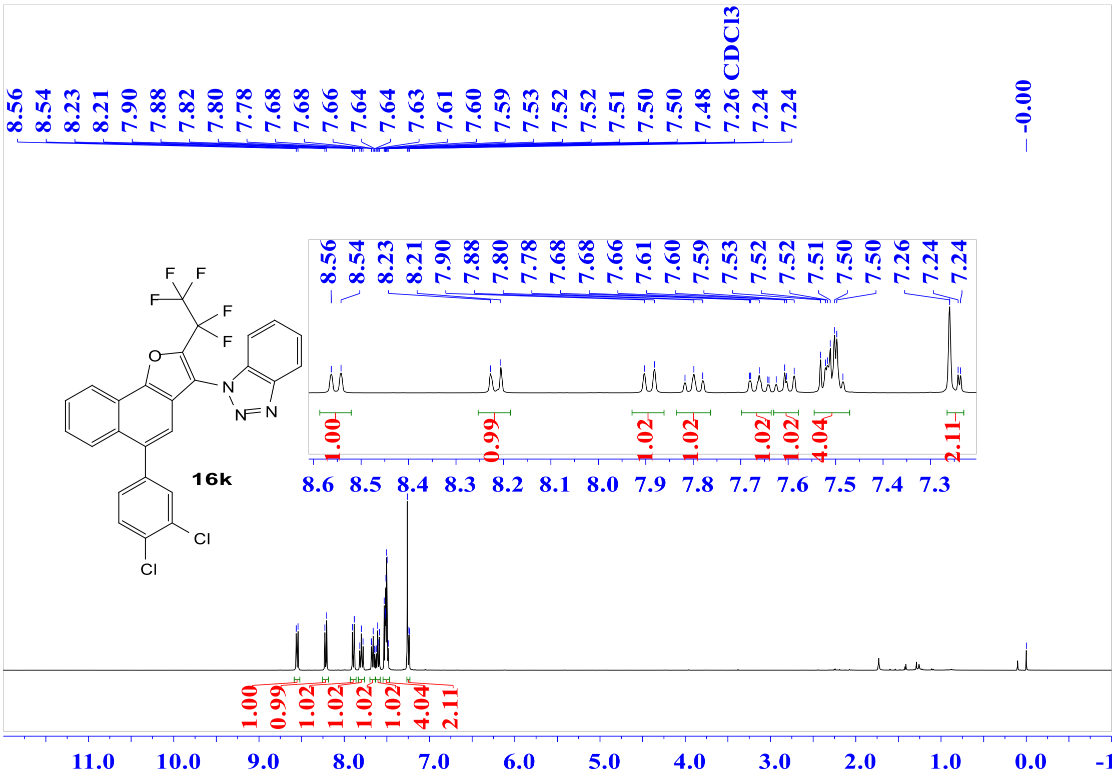

Figure S164. ^{19}F NMR spectrum of **16k**, related to **Scheme 3**.

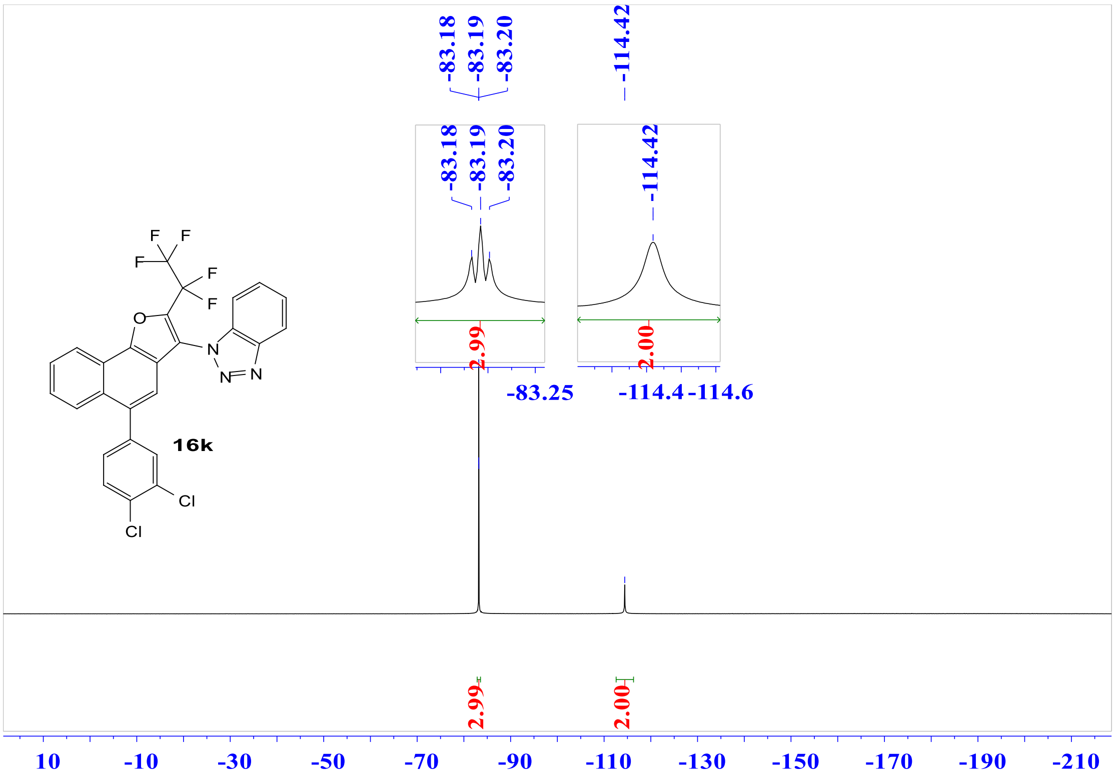

Figure S165. ^{13}C NMR spectrum of **16k**, related to **Scheme 3**.

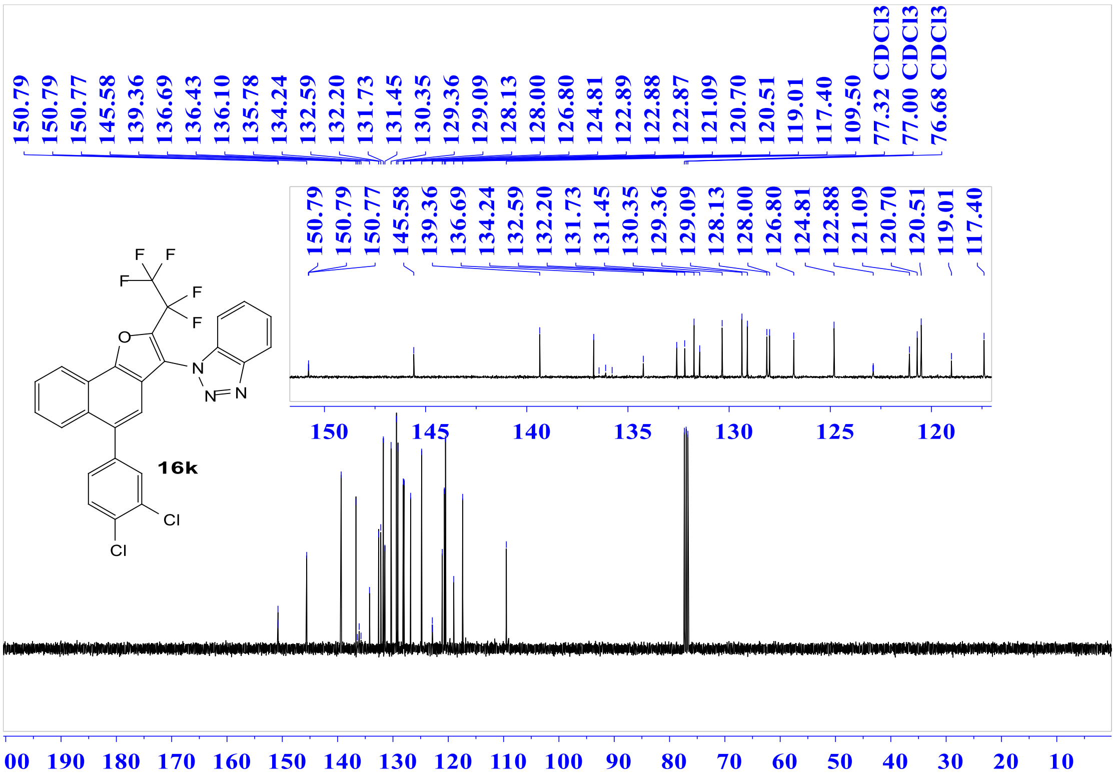

Figure S166. ^{1}H NMR spectrum of **16l**, related to **Scheme 3**.

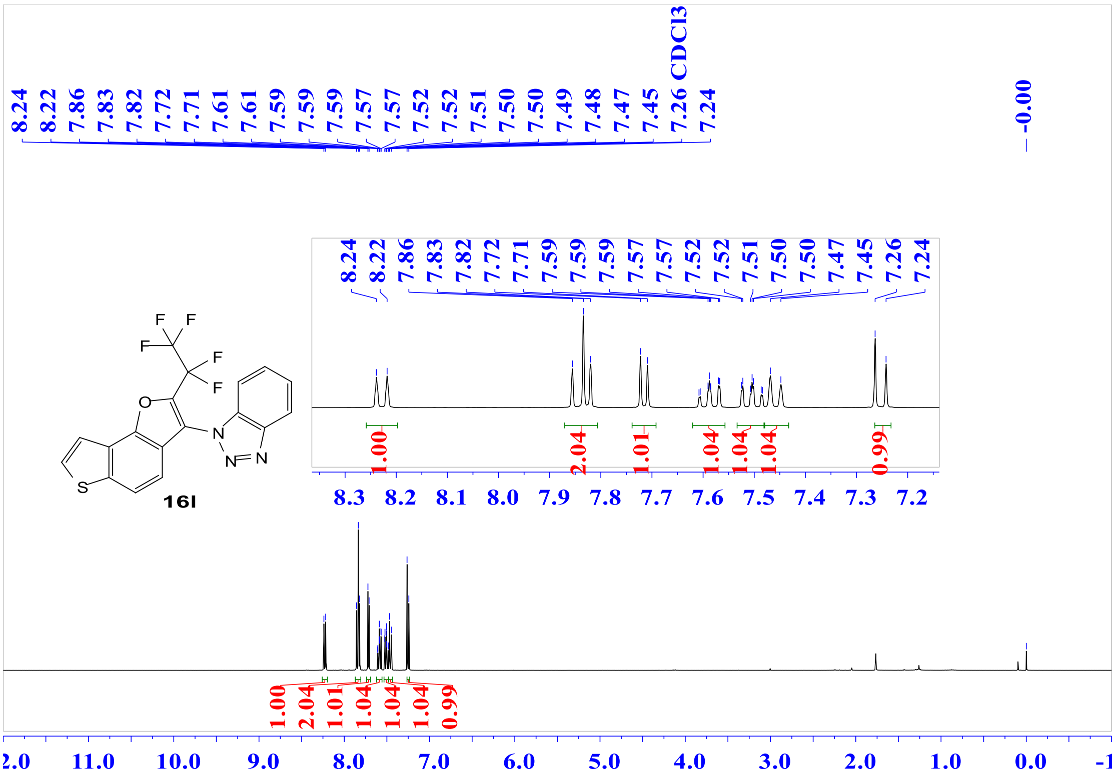

Figure S167. ^{19}F NMR spectrum of **16l**, related to **Scheme 3**.

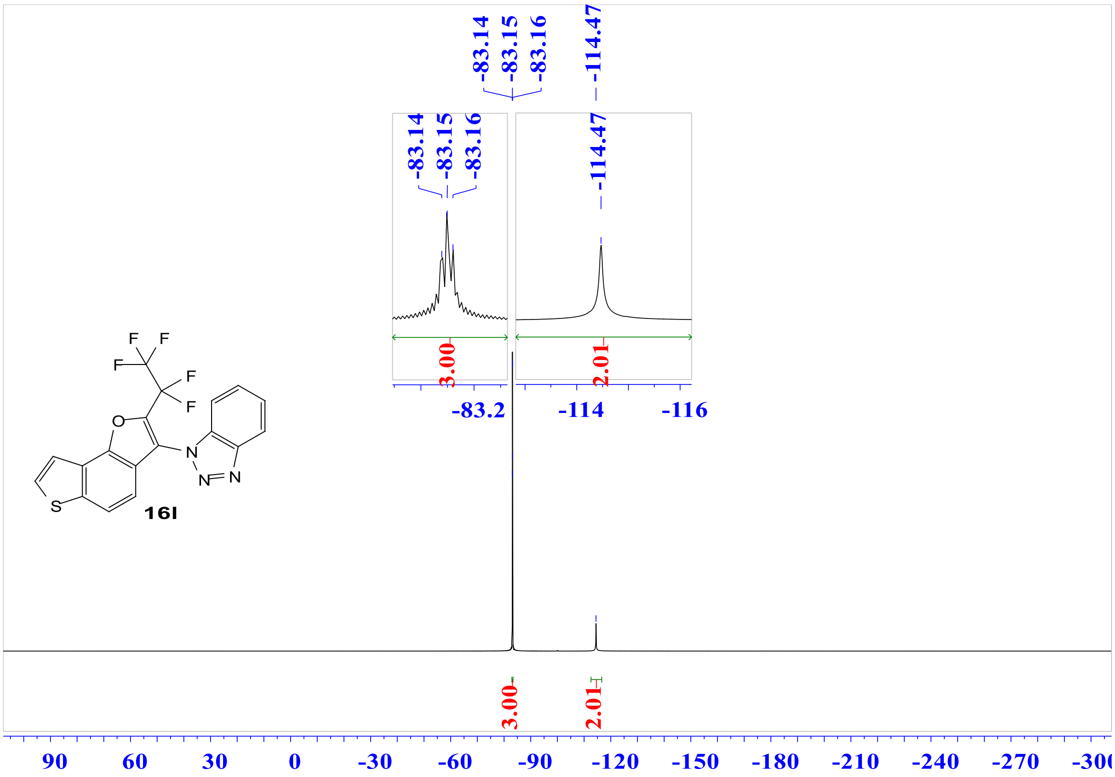

Figure S168. ^{13}C NMR spectrum of **16l**, related to **Scheme 3**.

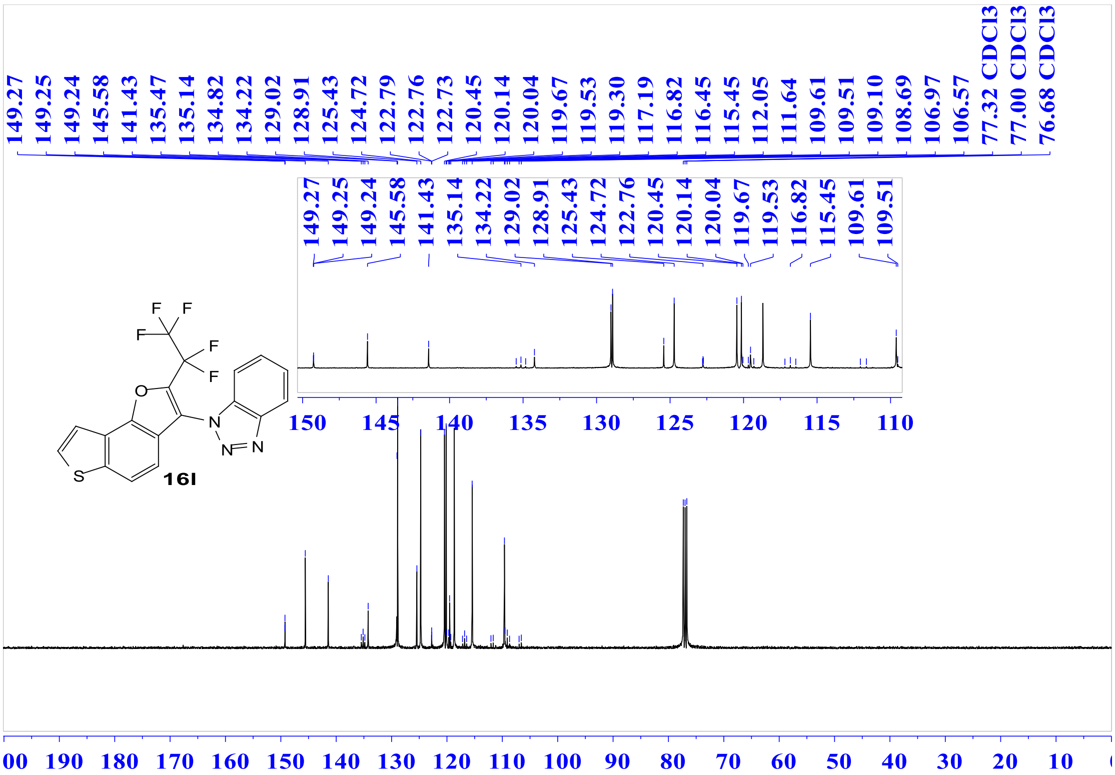

Figure S169. ^{1}H NMR spectrum of **16m**, related to **Scheme 3**.

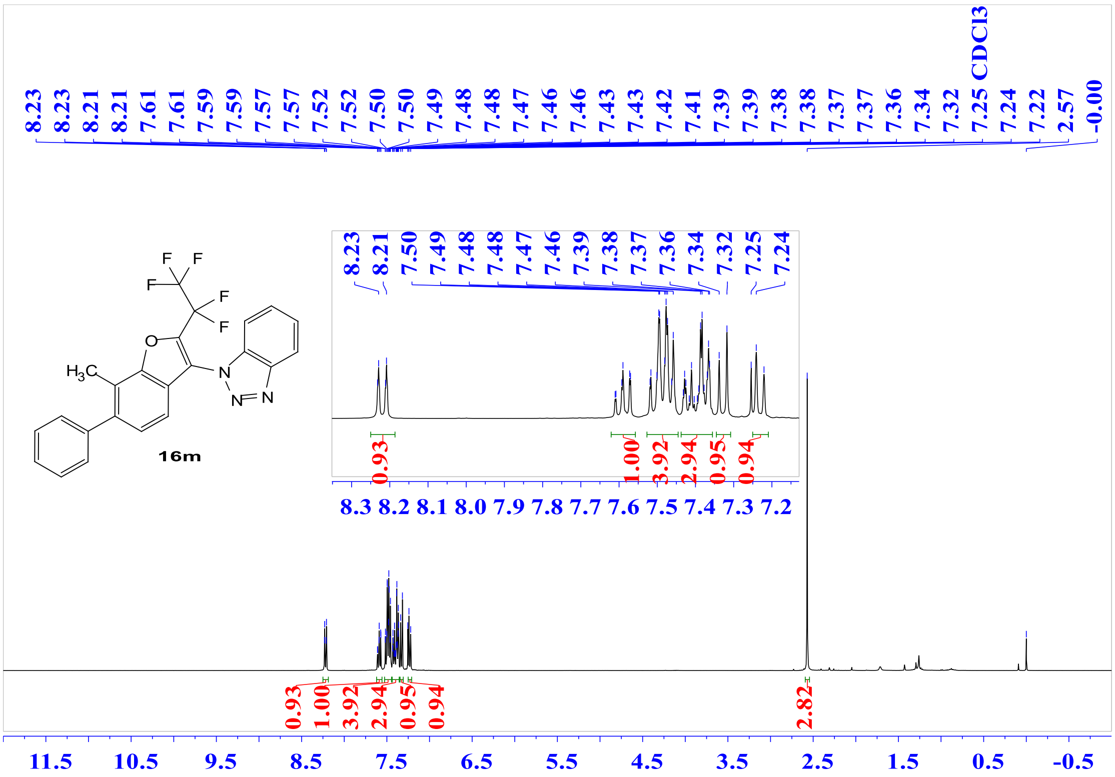

Figure S170. ^{19}F NMR spectrum of **16m**, related to **Scheme 3**.

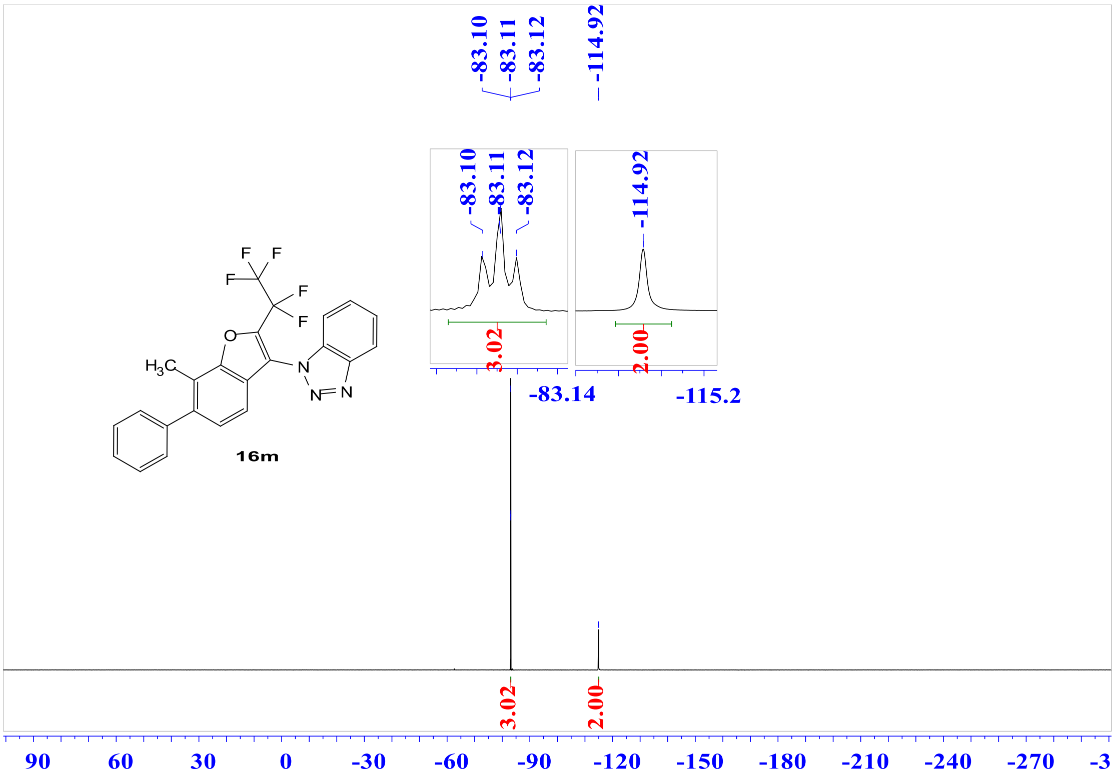

Figure S171. ^{13}C NMR spectrum of **16m**, related to **Scheme 3**.

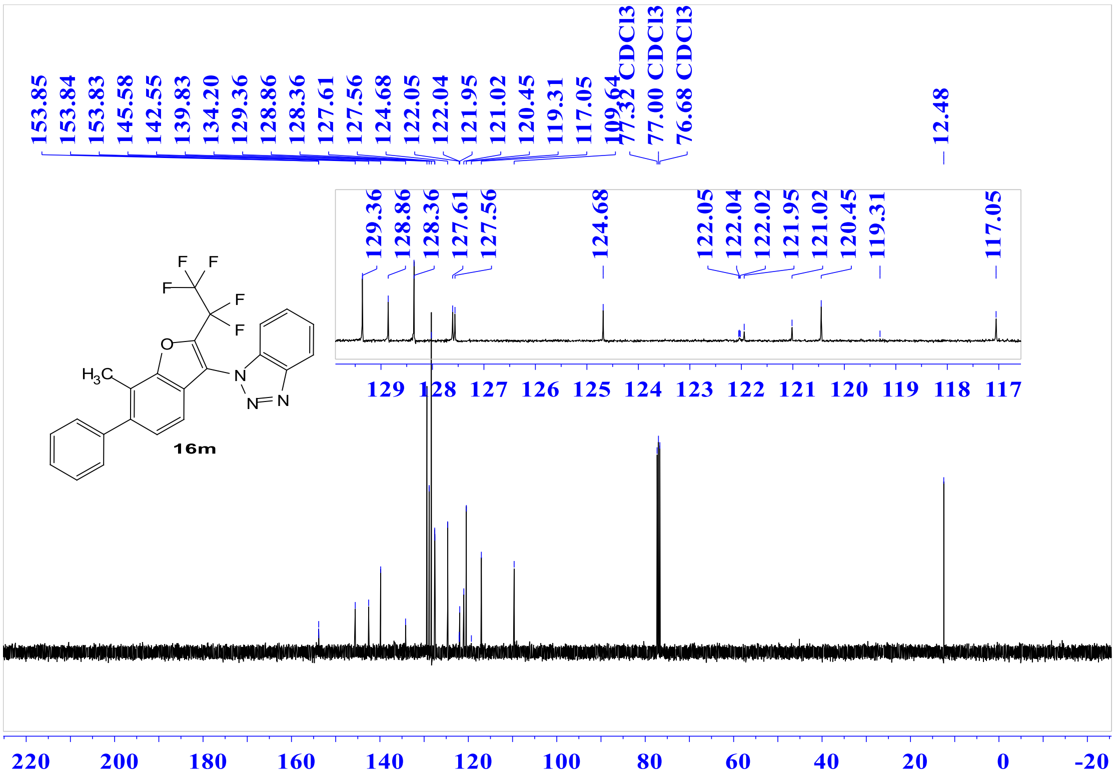

Figure S172. ^{1}H NMR spectrum of **16n**, related to **Scheme 3**.

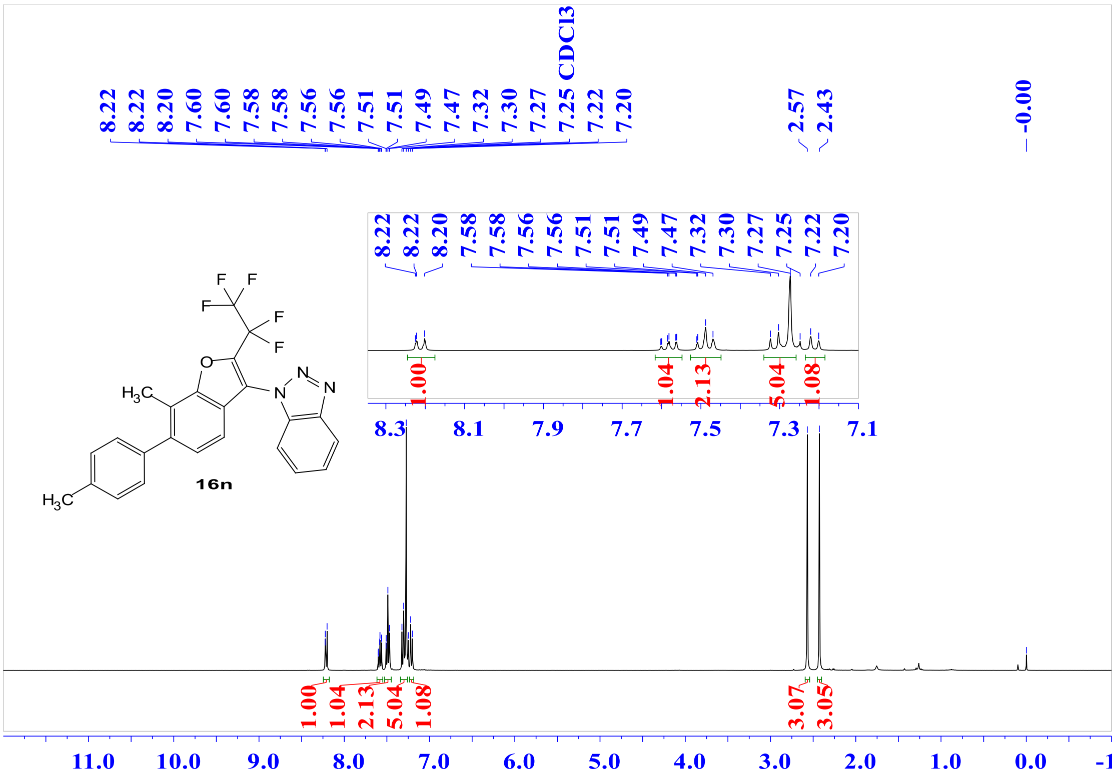

Figure S173. ^{19}F NMR spectrum of **16n**, related to **Scheme 3**.

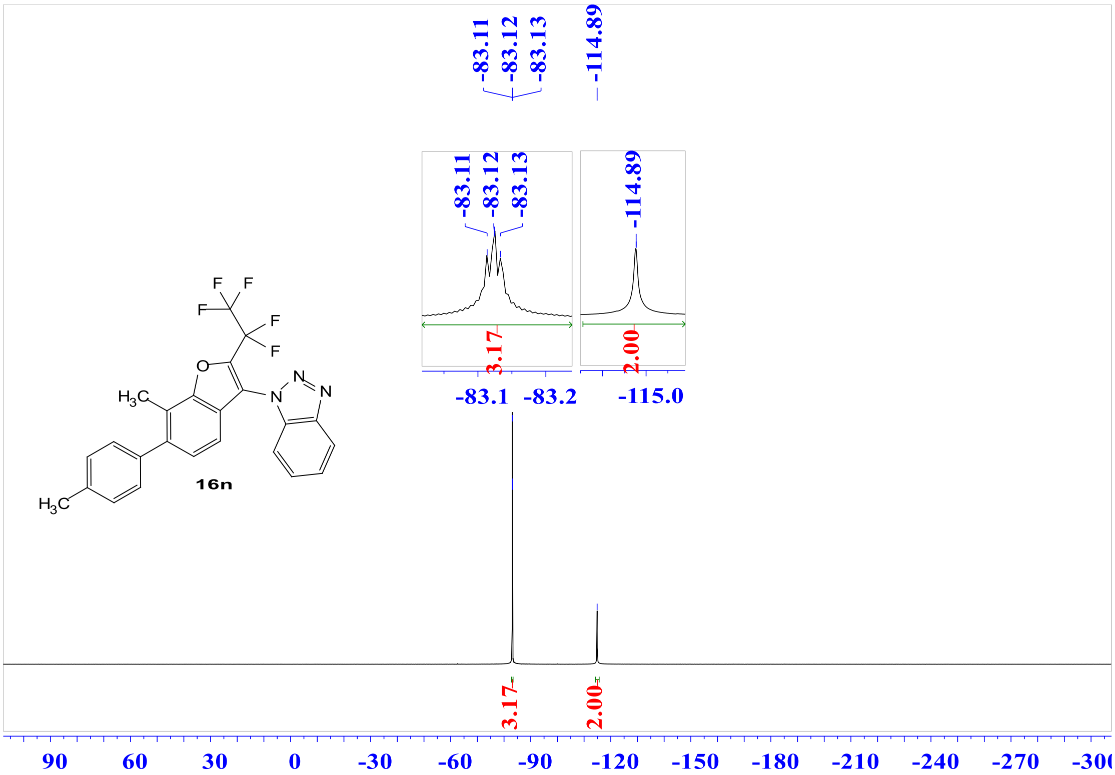

Figure S174. ^{13}C NMR spectrum of **16n**, related to **Scheme 3**.

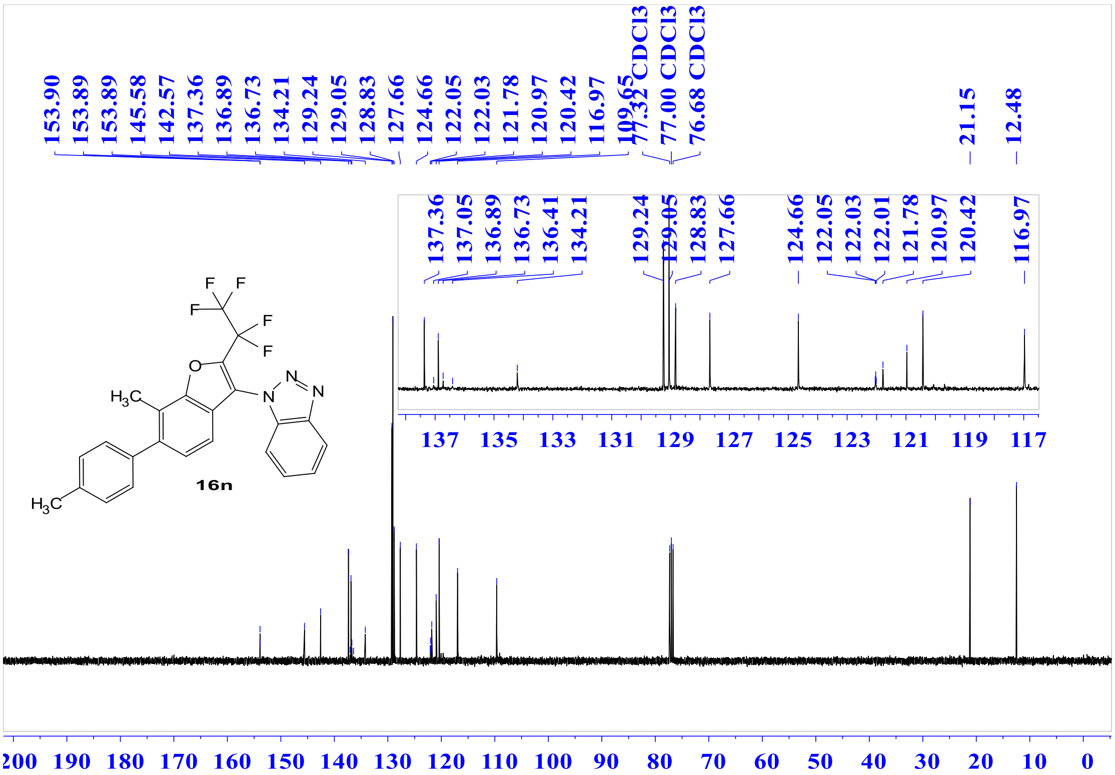

Figure S175. ^{1}H NMR spectrum of **16o**, related to **Scheme 3**.

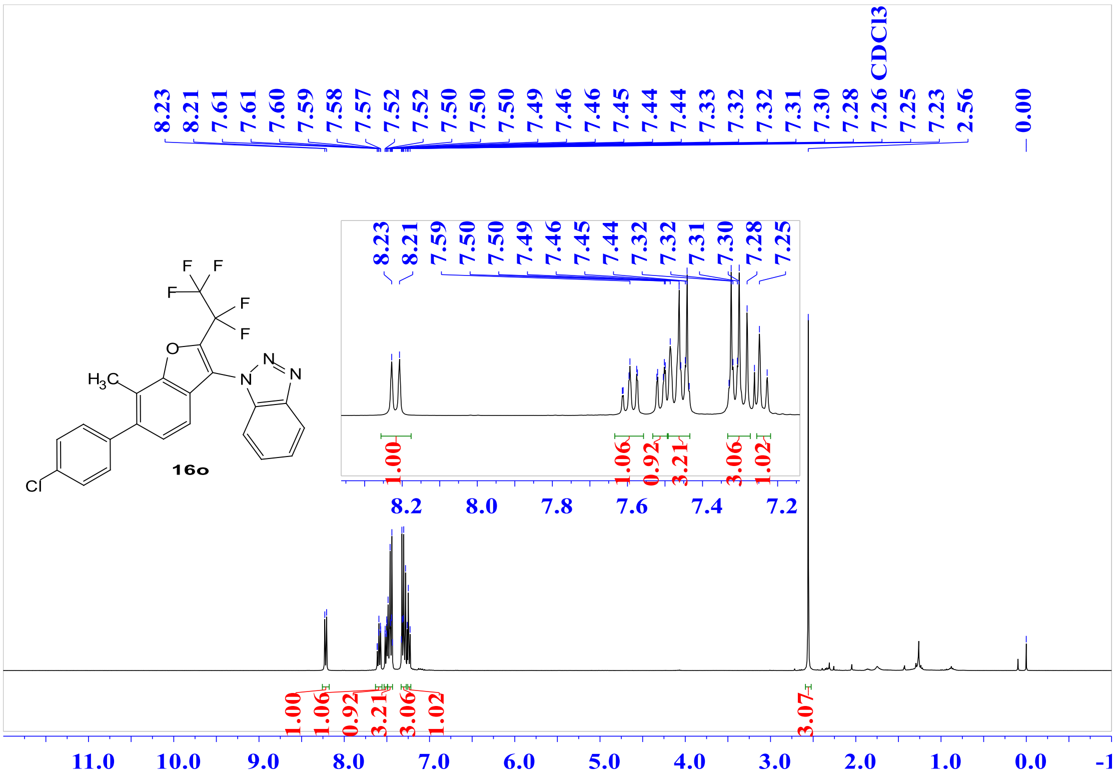

Figure S176. ^{19}F NMR spectrum of **16o**, related to **Scheme 3**.

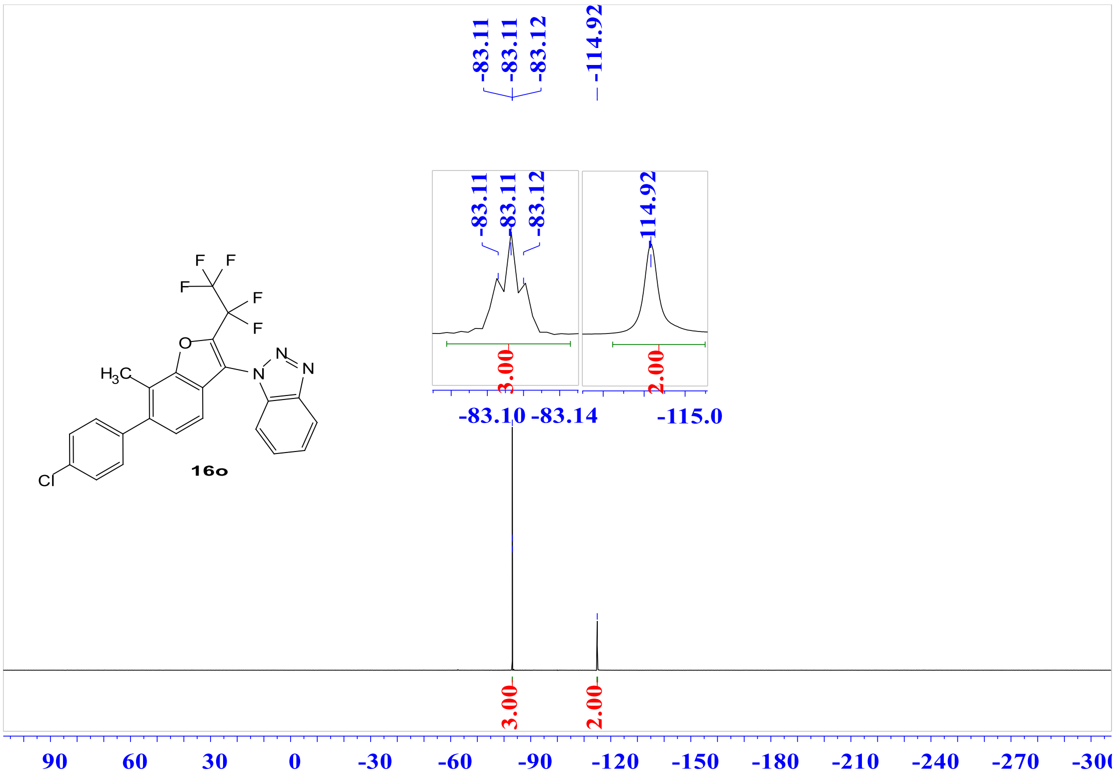

Figure S177. ^{13}C NMR spectrum of **16o**, related to **Scheme 3**.

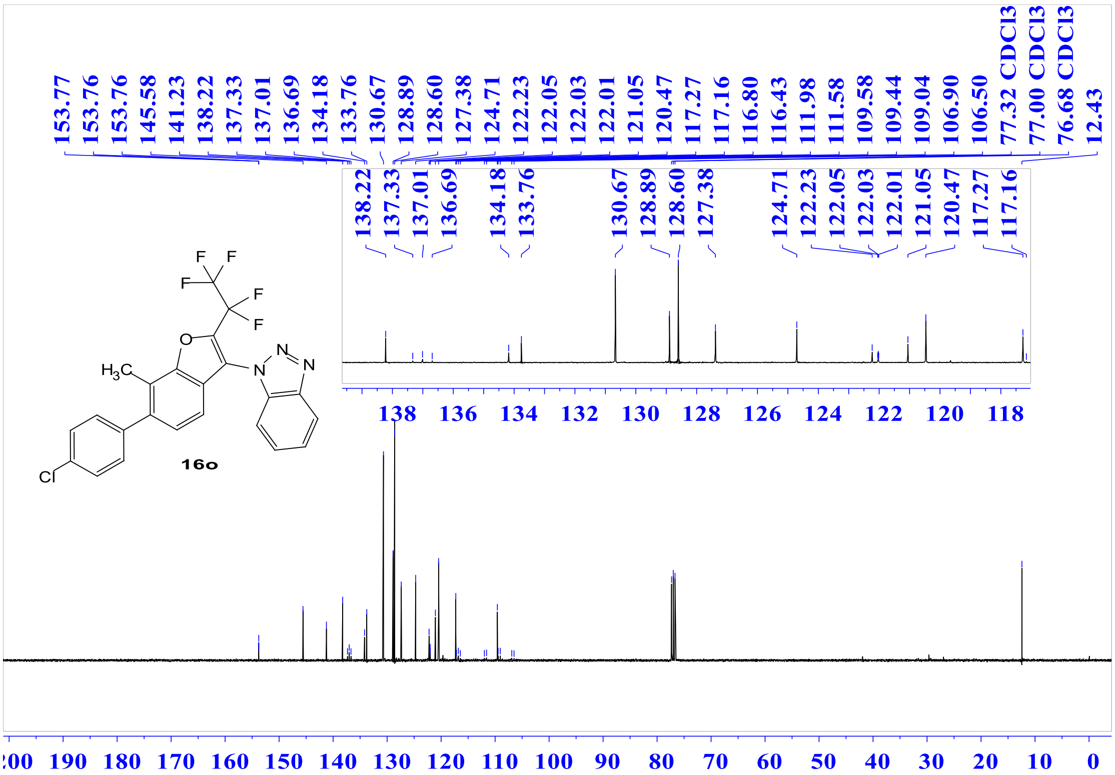

Figure S178. ^{1}H NMR spectrum of **16p**, related to **Scheme 3**.

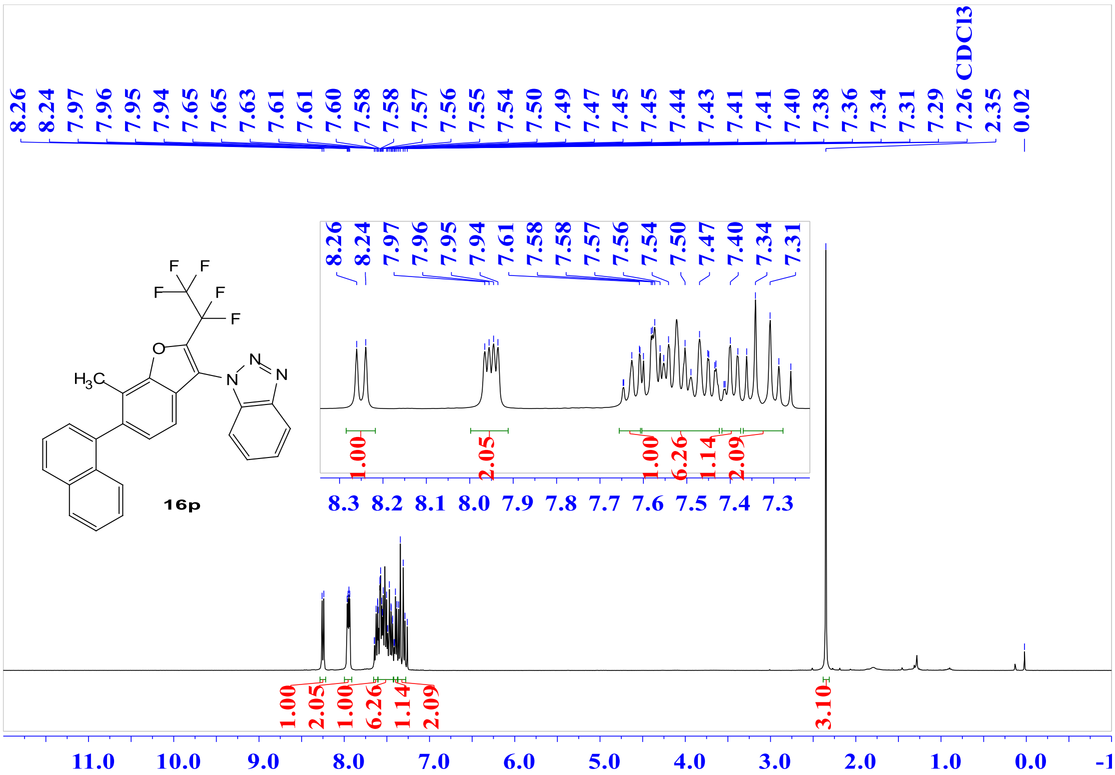

Figure S179. ^{19}F NMR spectrum of **16p**, related to **Scheme 3**.

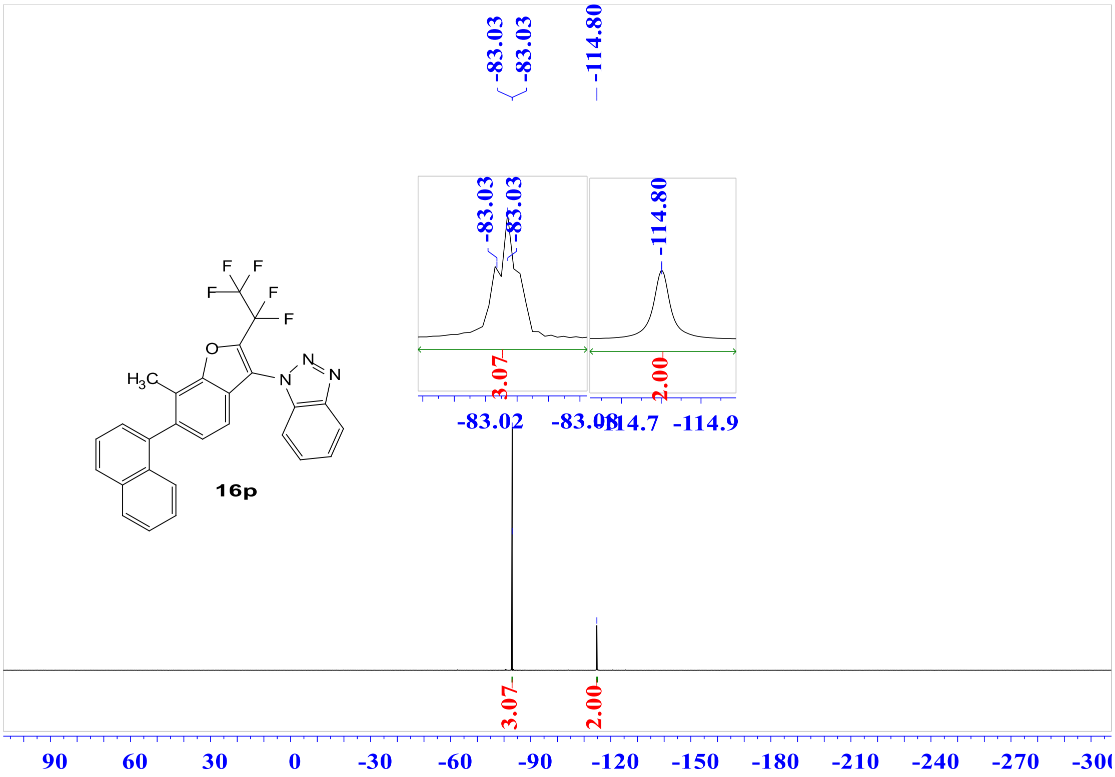

Figure S180. ^{13}C NMR spectrum of **16p**, related to **Scheme 3**.

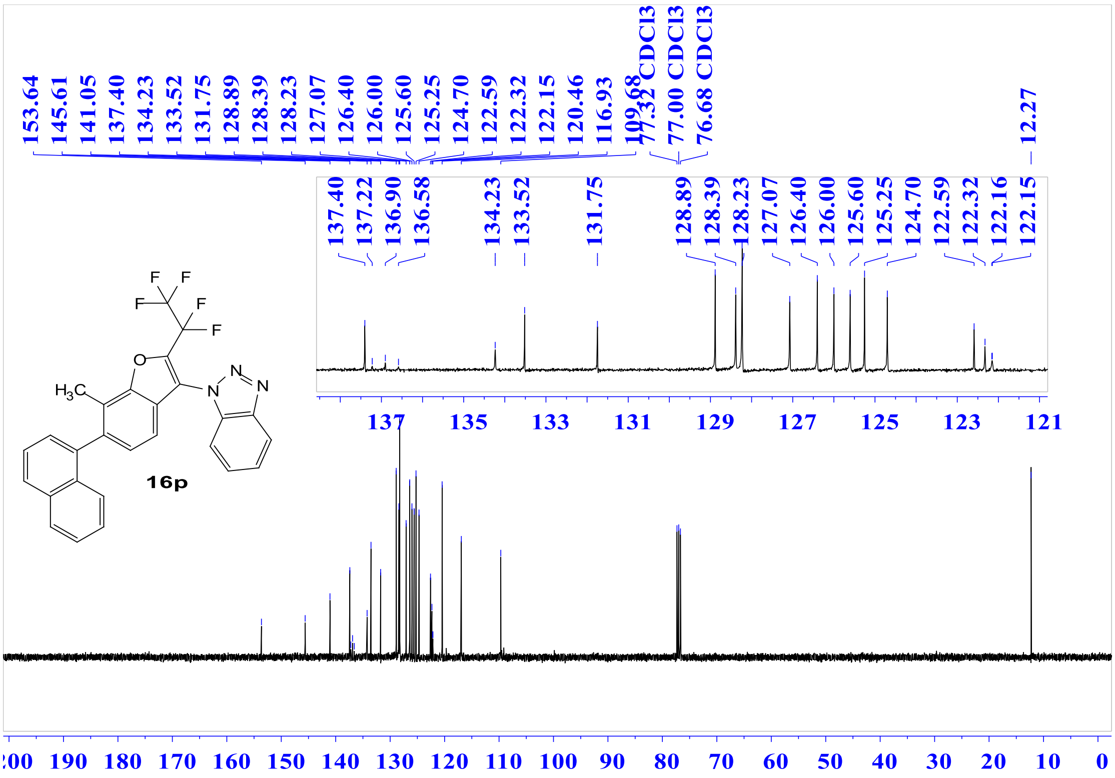

Figure S181. ^{1}H NMR spectrum of **16q**, related to **Scheme 3**.

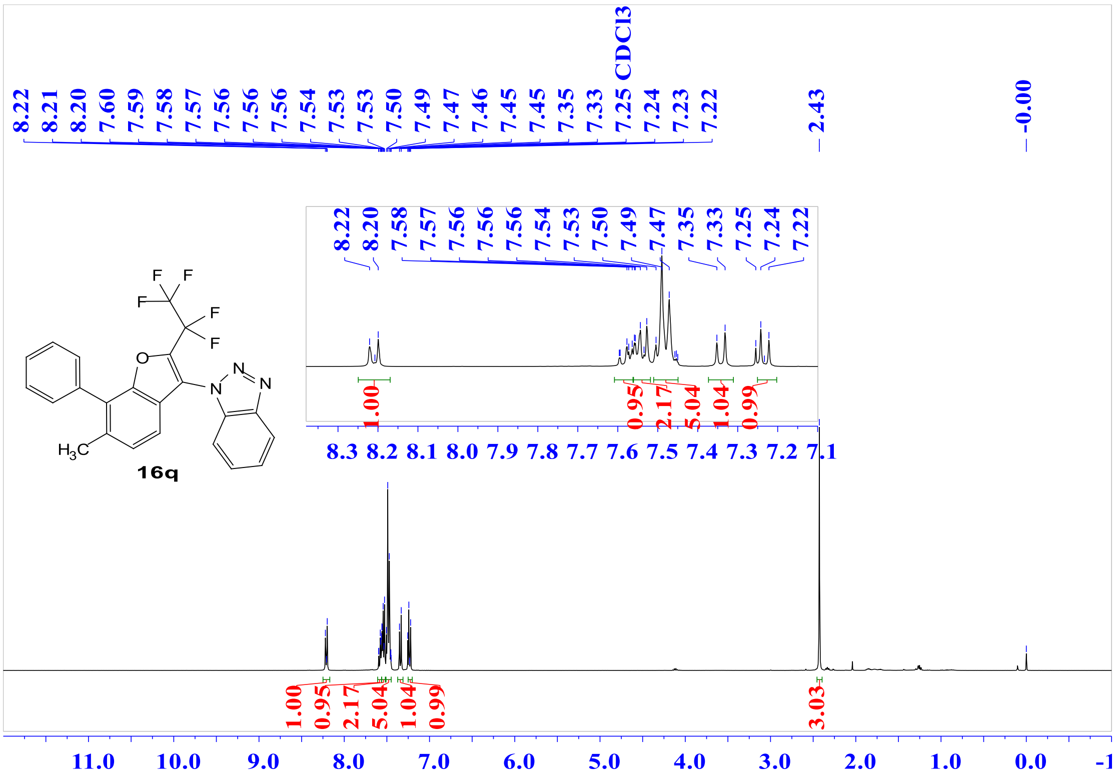

Figure S182. ^{19}F NMR spectrum of **16q**, related to **Scheme 3**.

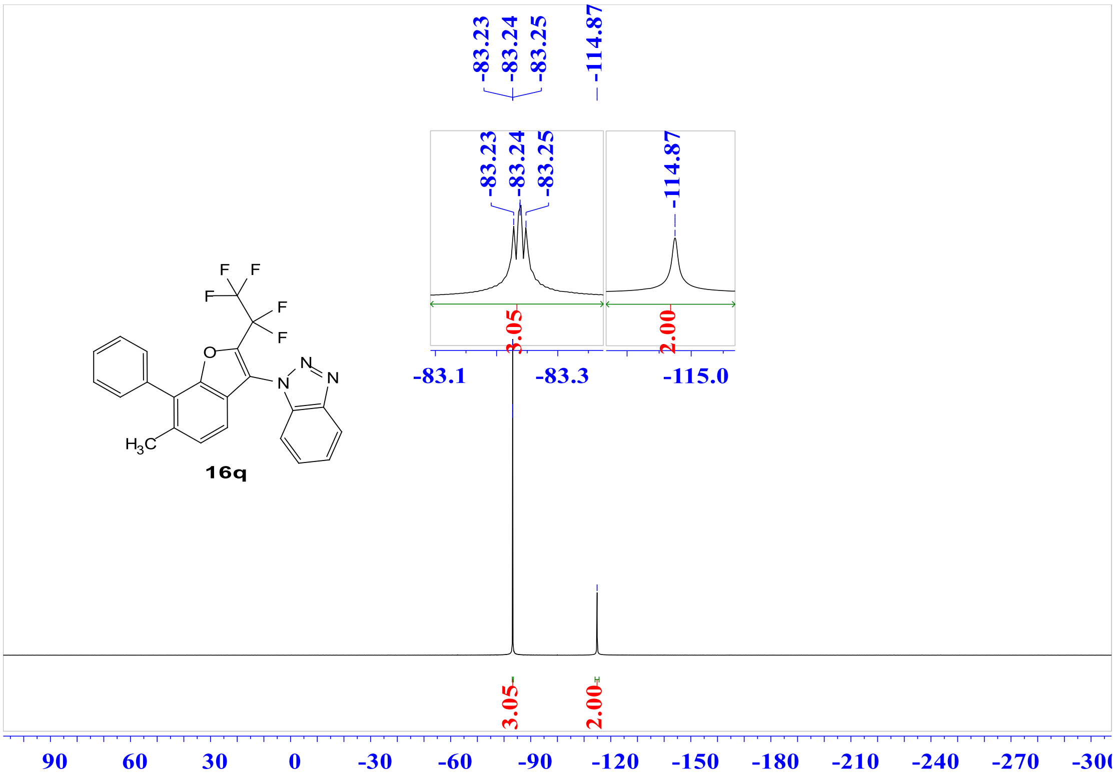

Figure S183. ^{13}C NMR spectrum of **16q**, related to **Scheme 3**.

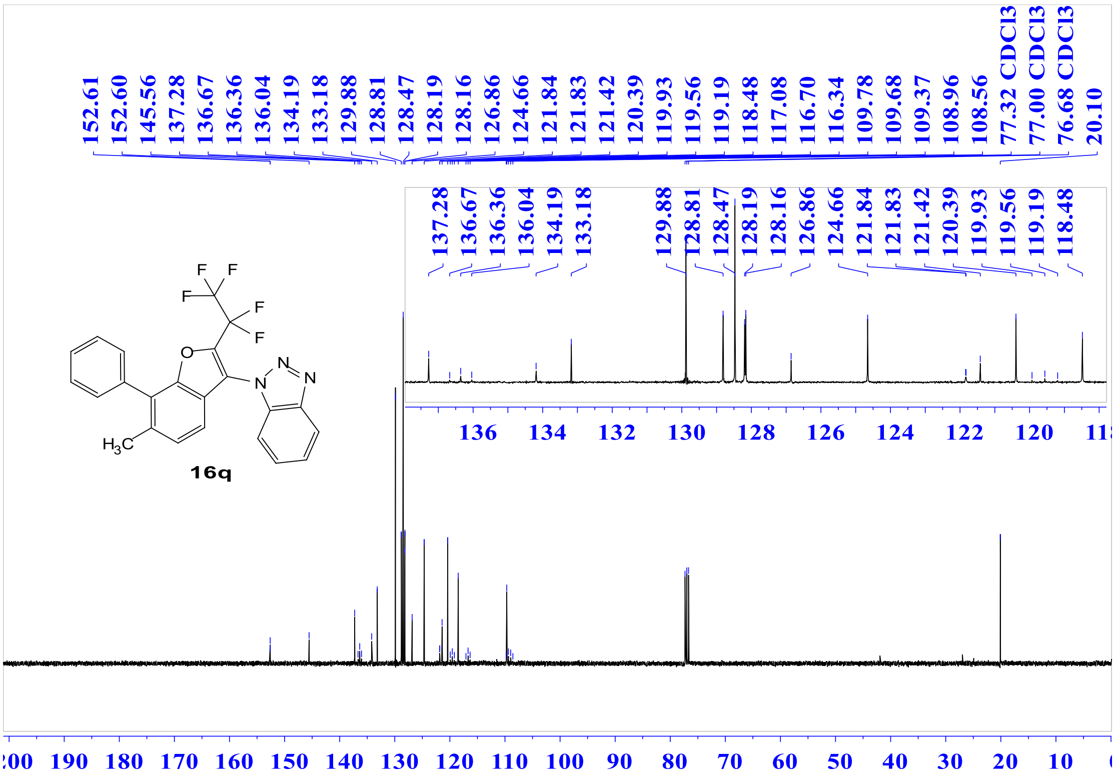

Figure S184. ^{1}H NMR spectrum of **16r**, related to **Scheme 3**.

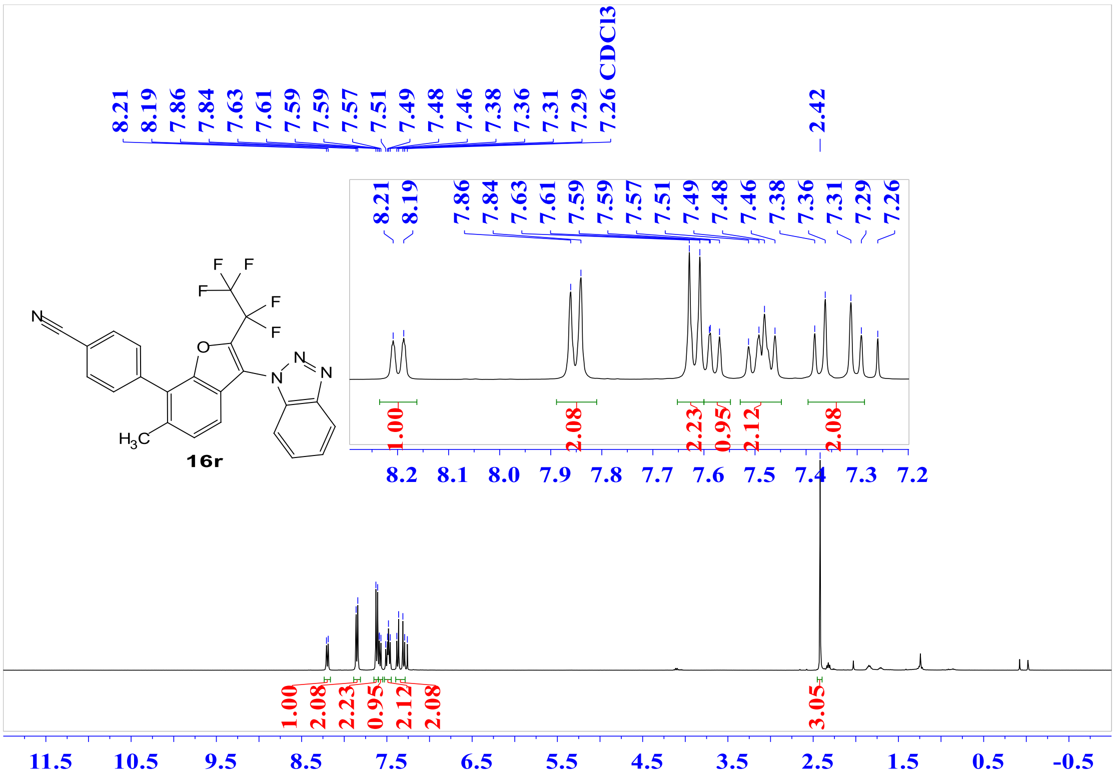

Figure S185. ^{19}F NMR spectrum of **16r**, related to **Scheme 3**.

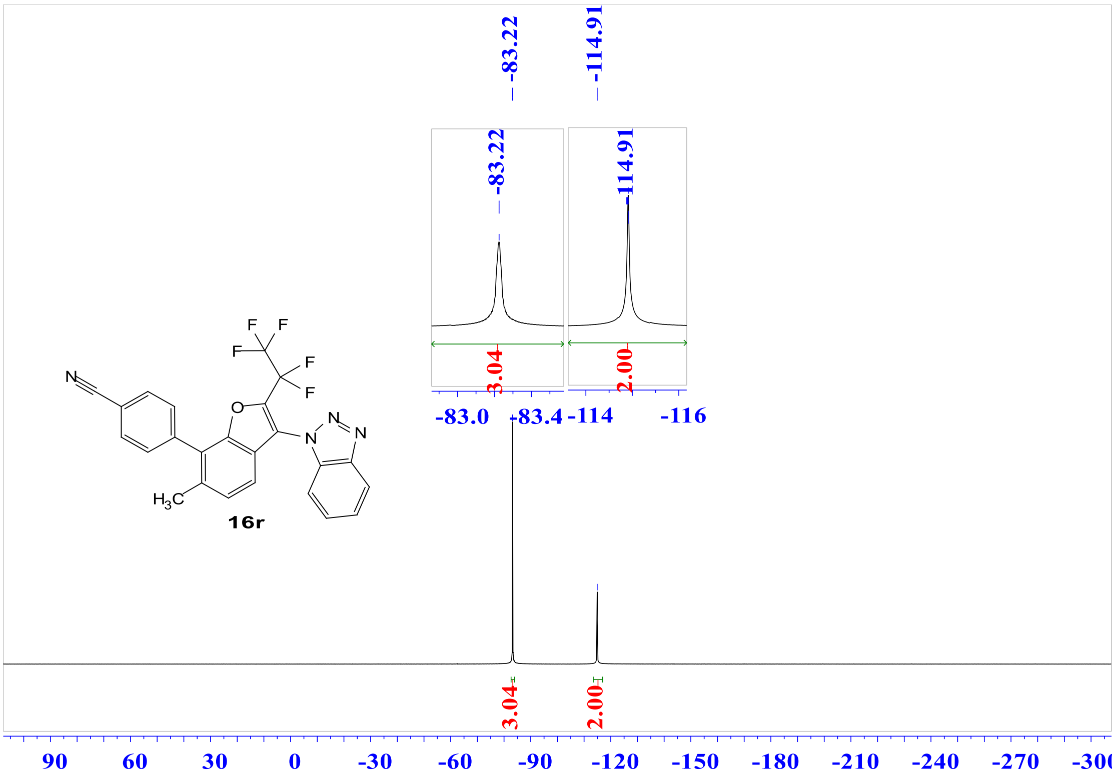

Figure S186. ^{13}C NMR spectrum of **16r**, related to **Scheme 3**.

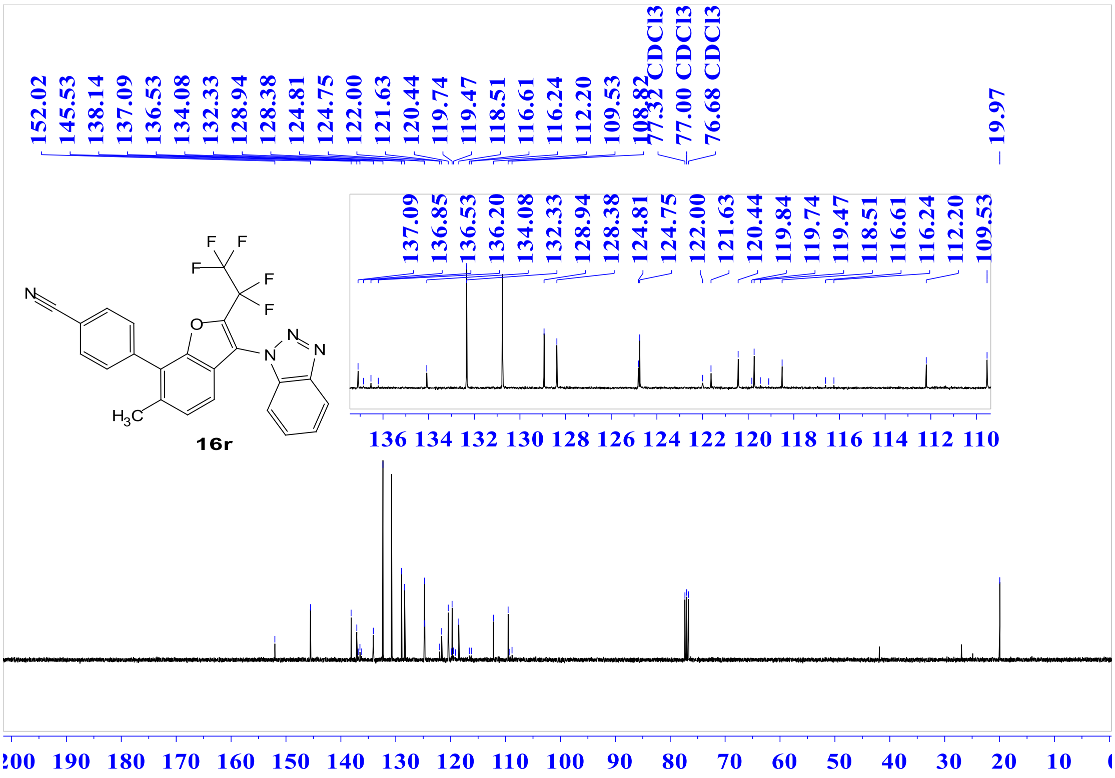

Figure S187. ^{1}H NMR spectrum of **16s**, related to **Scheme 3**.

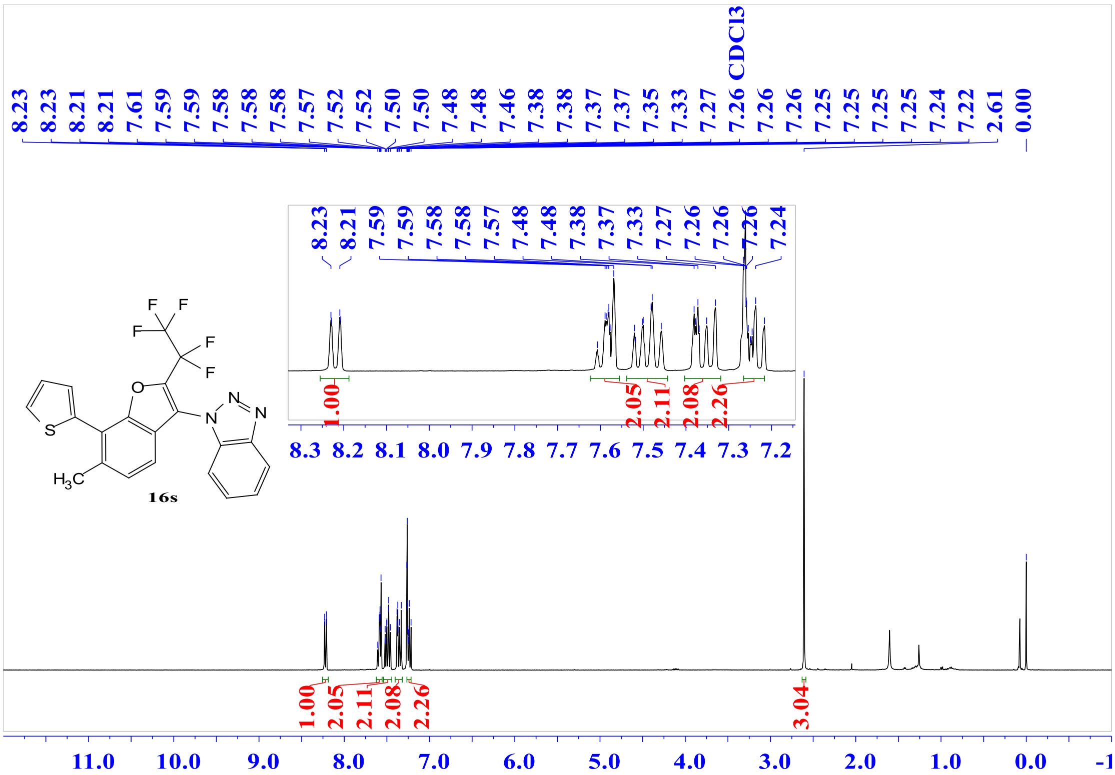

Figure S188. ^{19}F NMR spectrum of **16s**, related to **Scheme 3**.

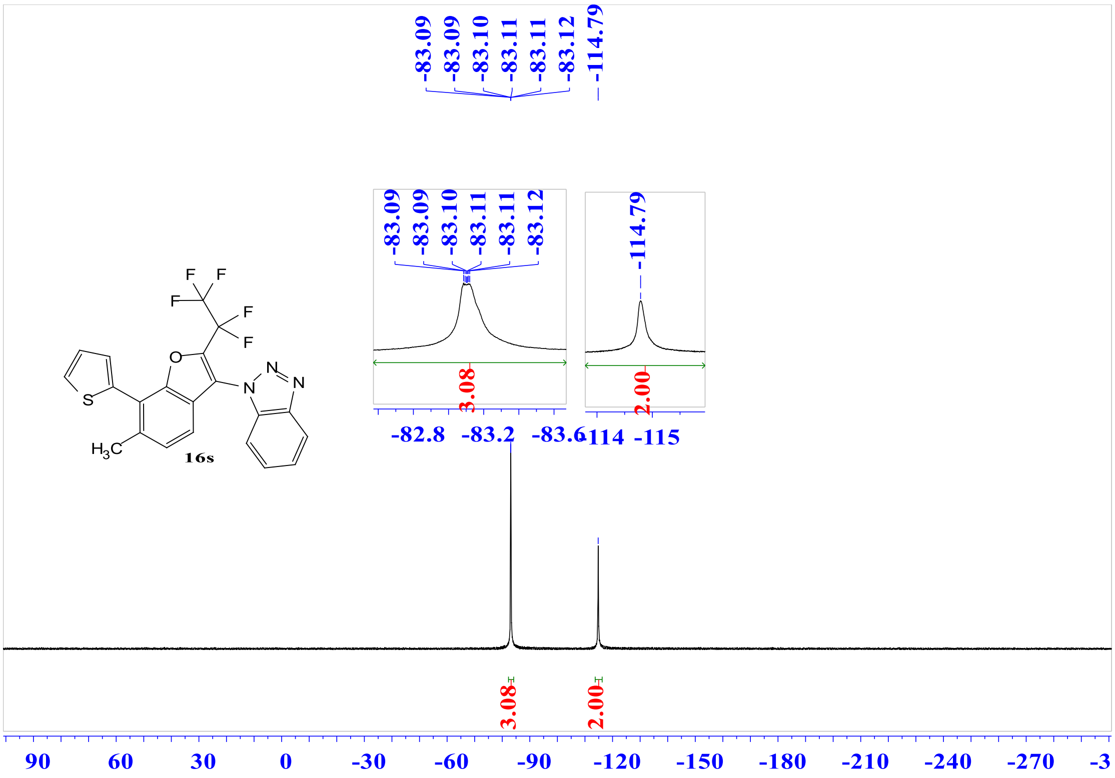

Figure S189. ^{13}C NMR spectrum of **16s**, related to **Scheme 3**.

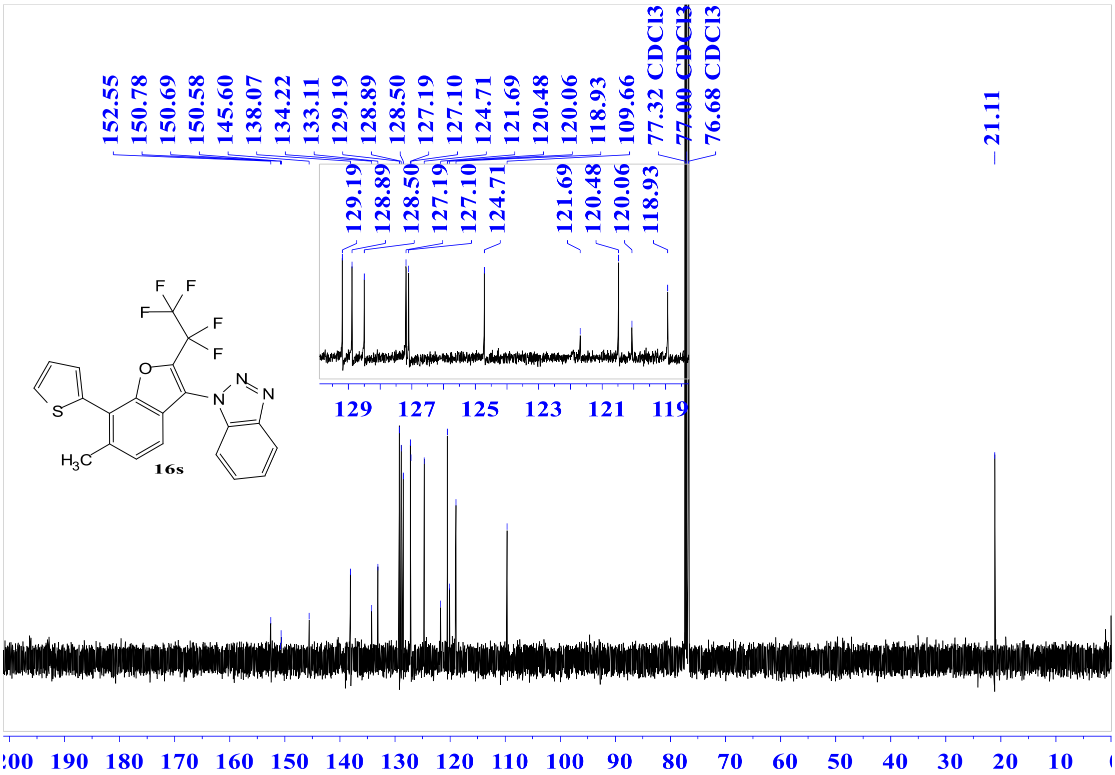

Figure S190. ^{1}H NMR spectrum of **16t**, related to **Scheme 3**.

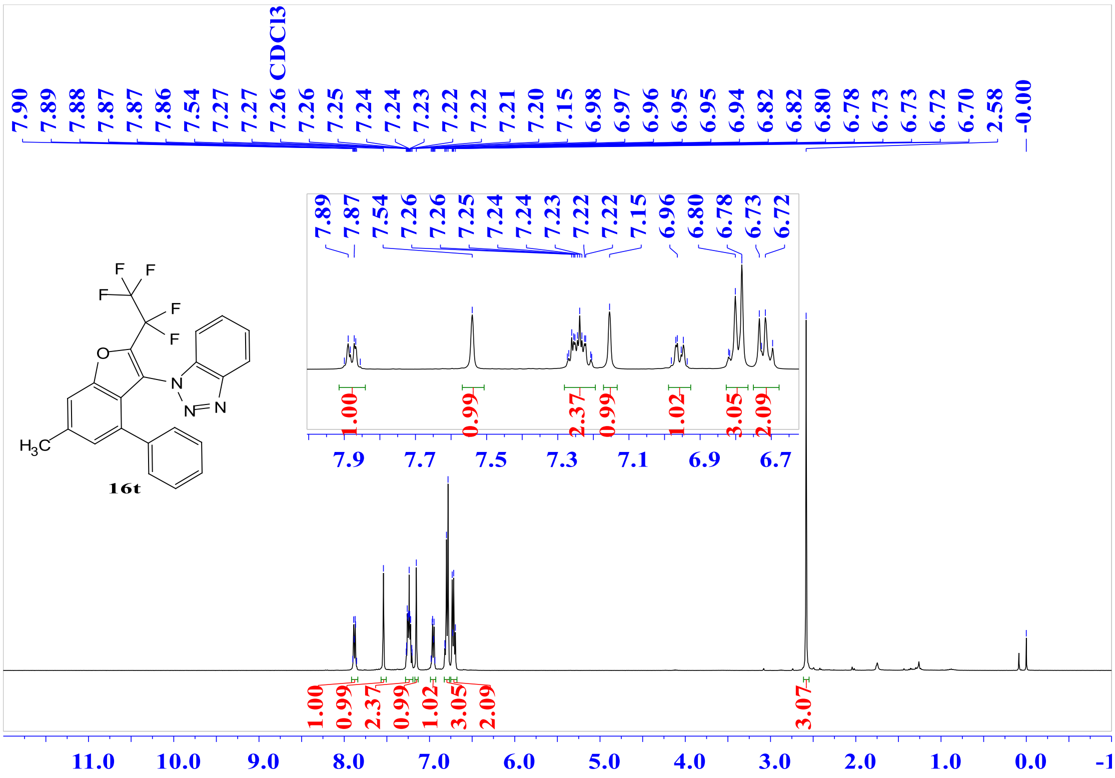

Figure S191. ^{19}F NMR spectrum of **16t**, related to **Scheme 3**.

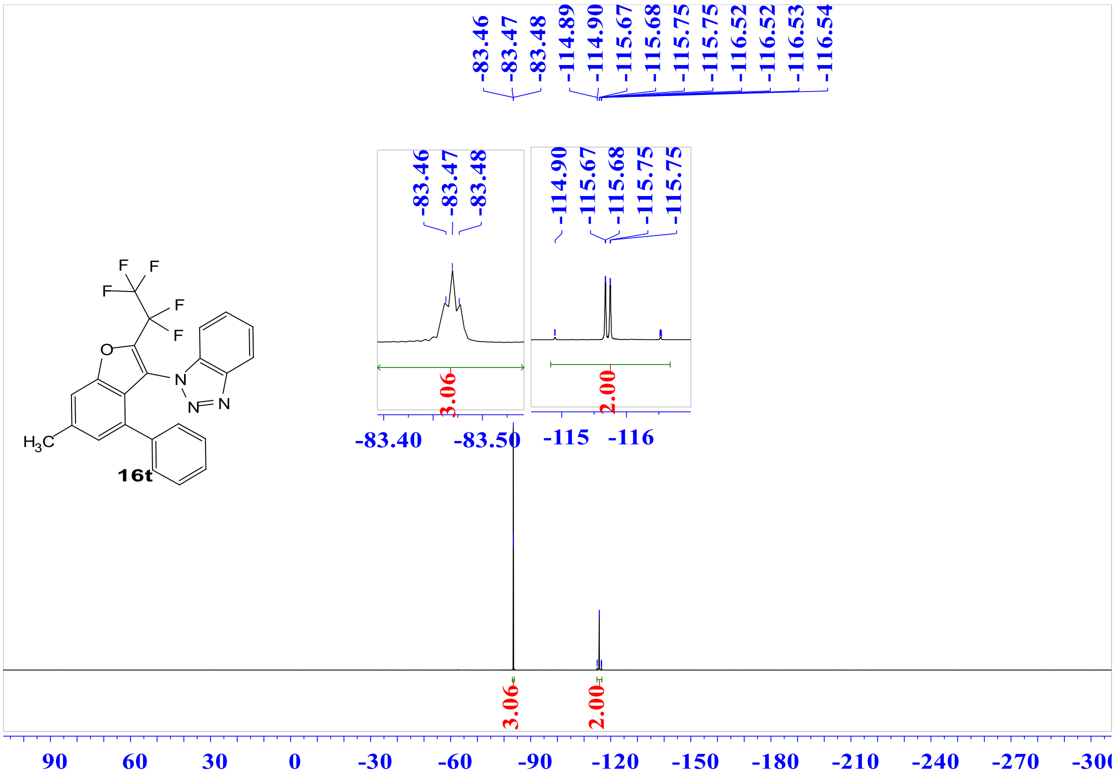

Figure S192. ^{13}C NMR spectrum of **16t**, related to **Scheme 3**.

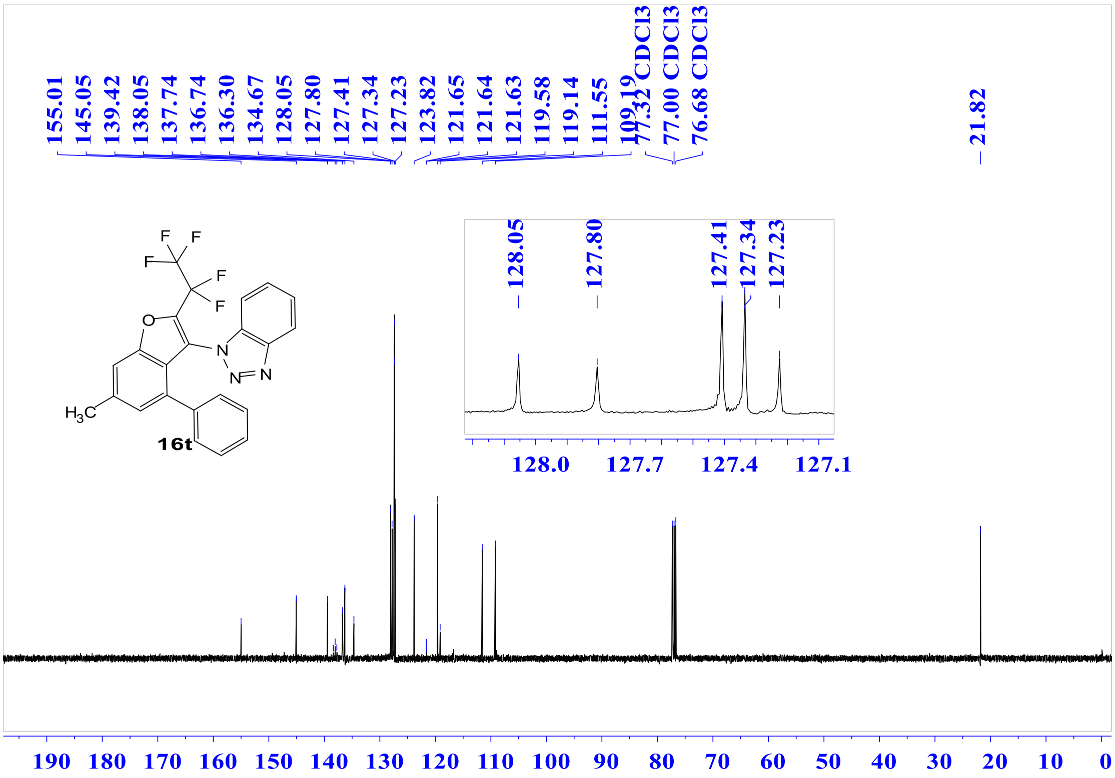

Figure S193. ^{1}H NMR spectrum of **16u**, related to **Scheme 3**.

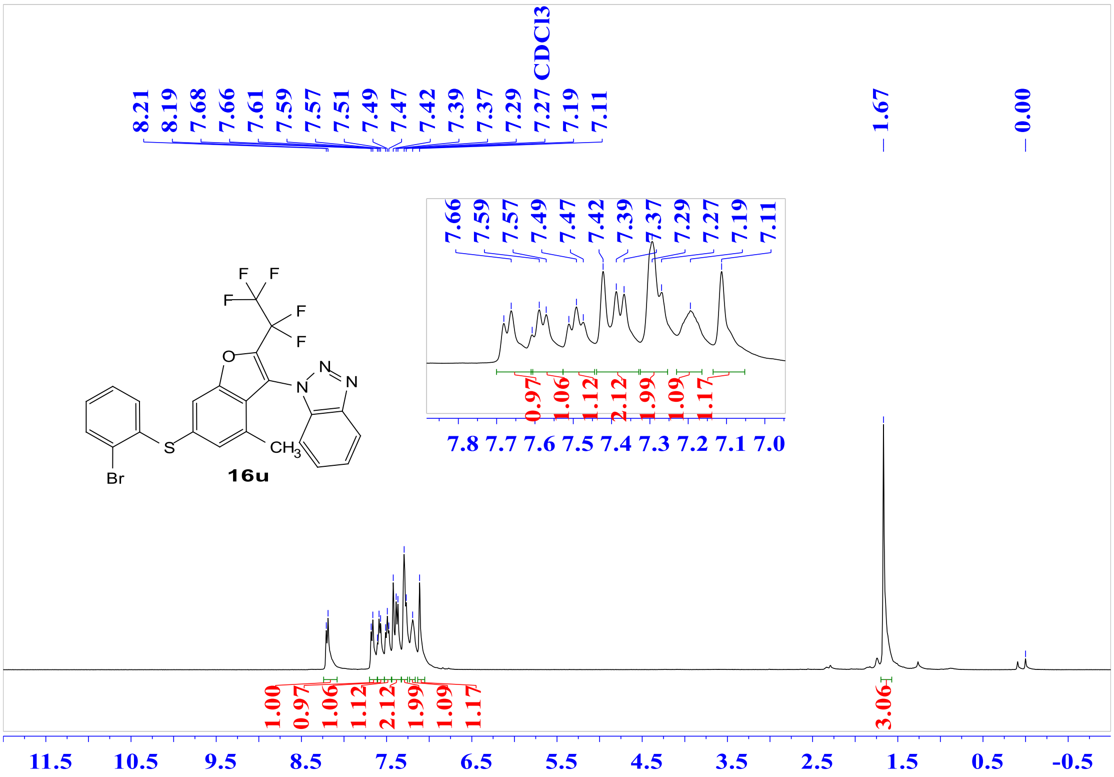

Figure S194. ^{19}F NMR spectrum of **16u**, related to **Scheme 3**.

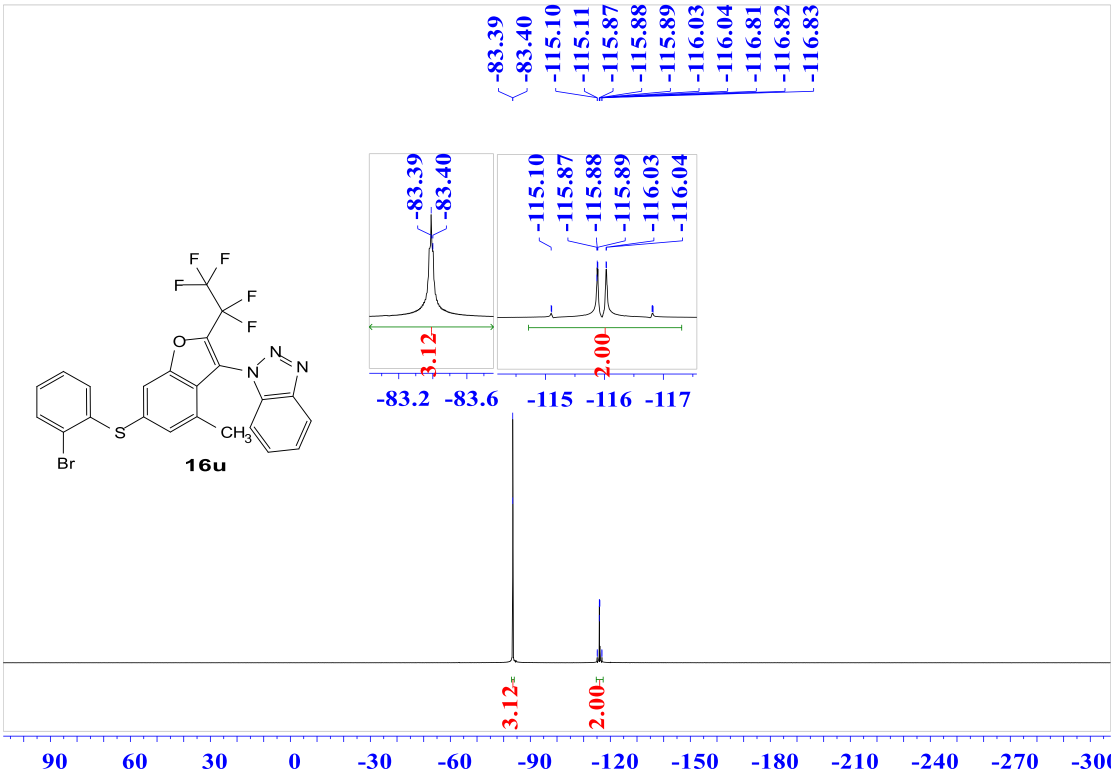

Figure S195. ^{13}C NMR spectrum of **16u**, related to **Scheme 3**.

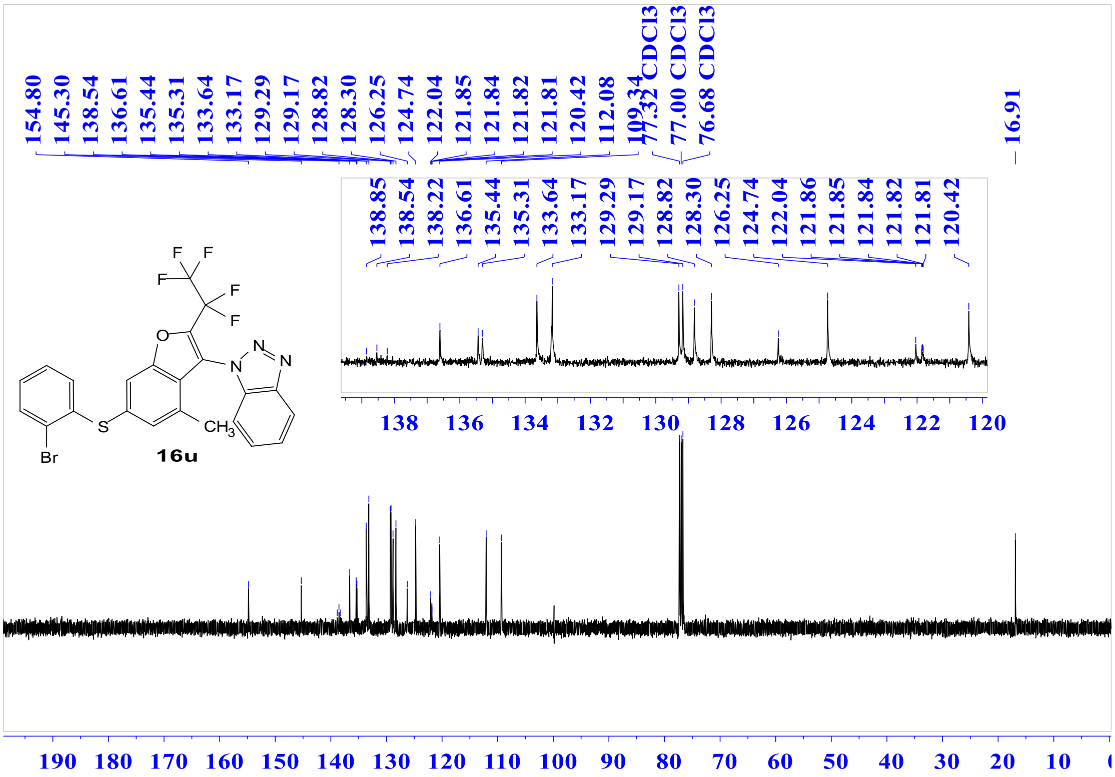

Figure S196. ^{1}H NMR spectrum of **16v**, related to **Scheme 3**.

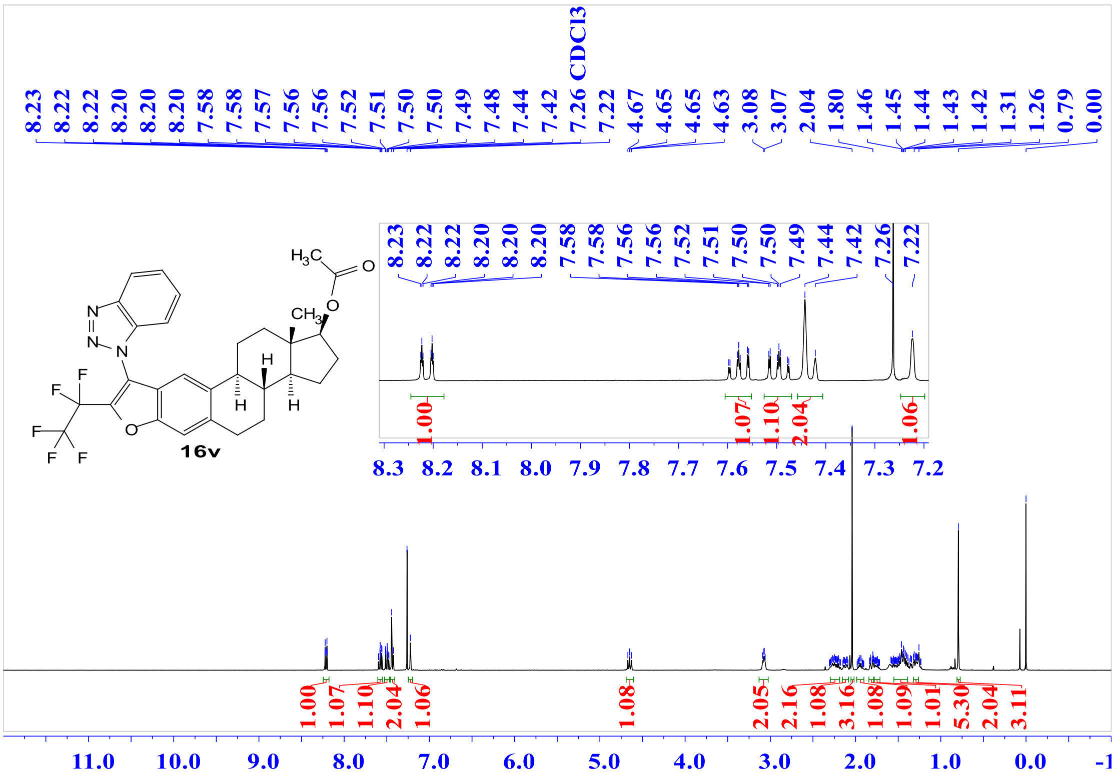

Figure S197. ^{19}F NMR spectrum of **16v**, related to **Scheme 3**.

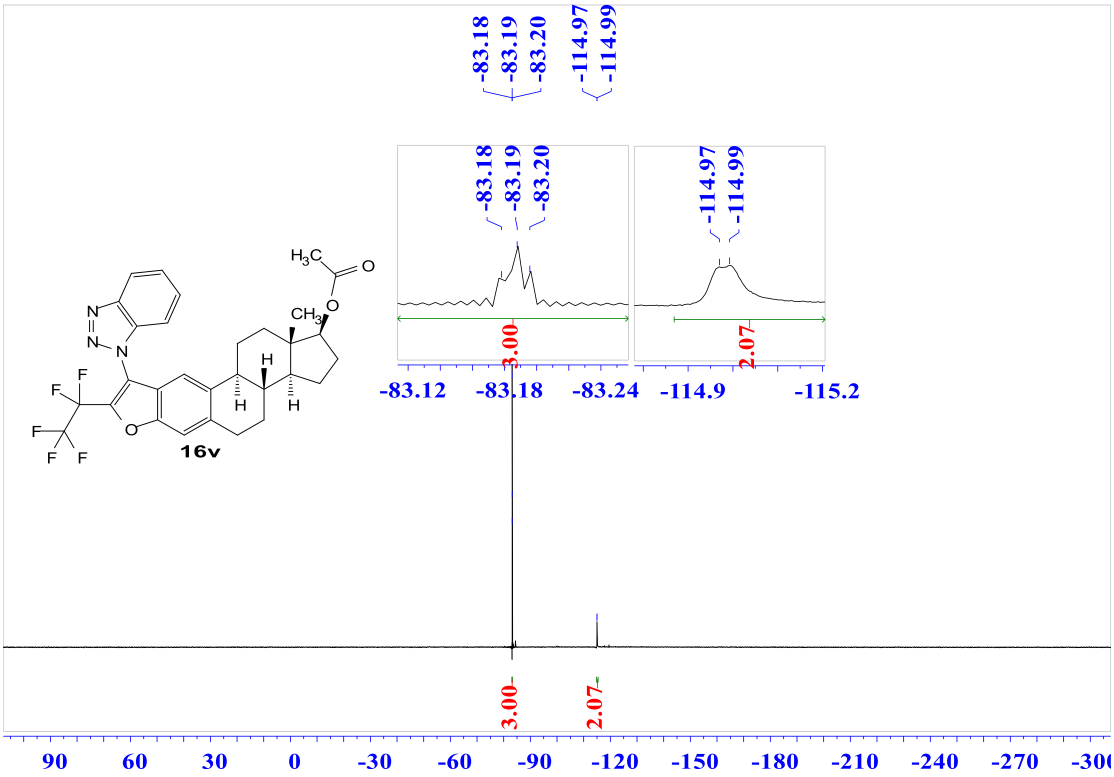

Figure S198. ^{13}C NMR spectrum of **16v**, related to **Scheme 3**.

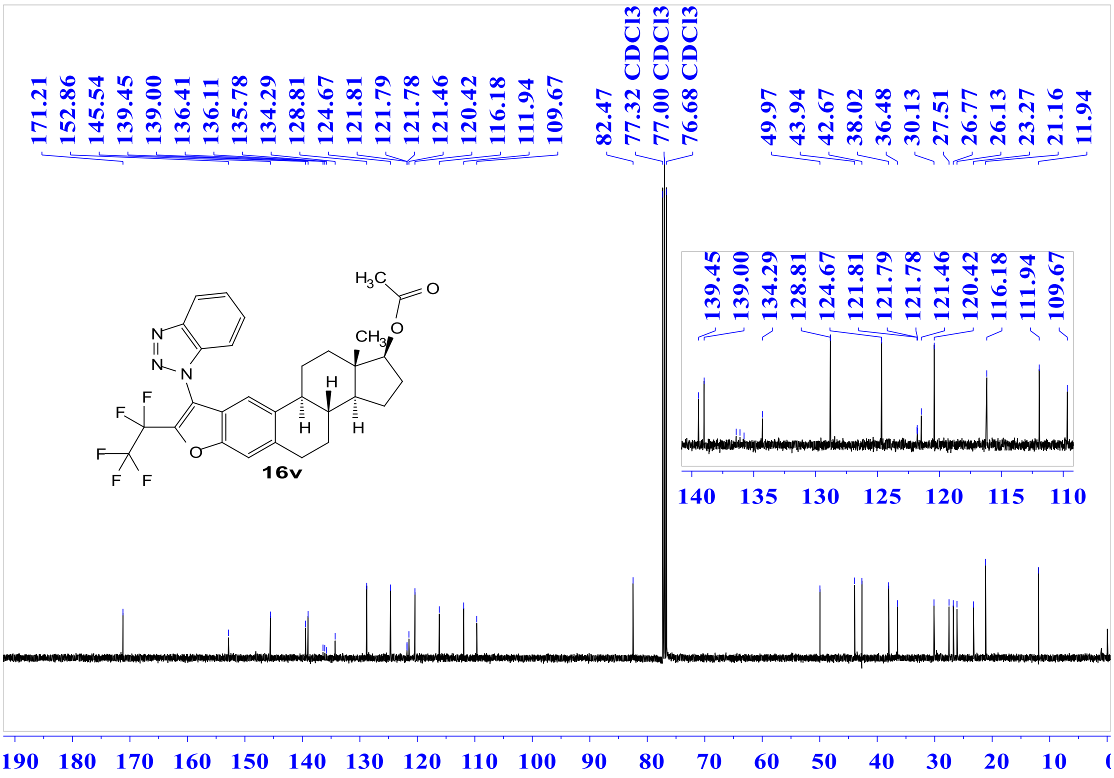

Figure S199. ^{1}H NMR spectrum of **16w**, related to **Scheme 3**.

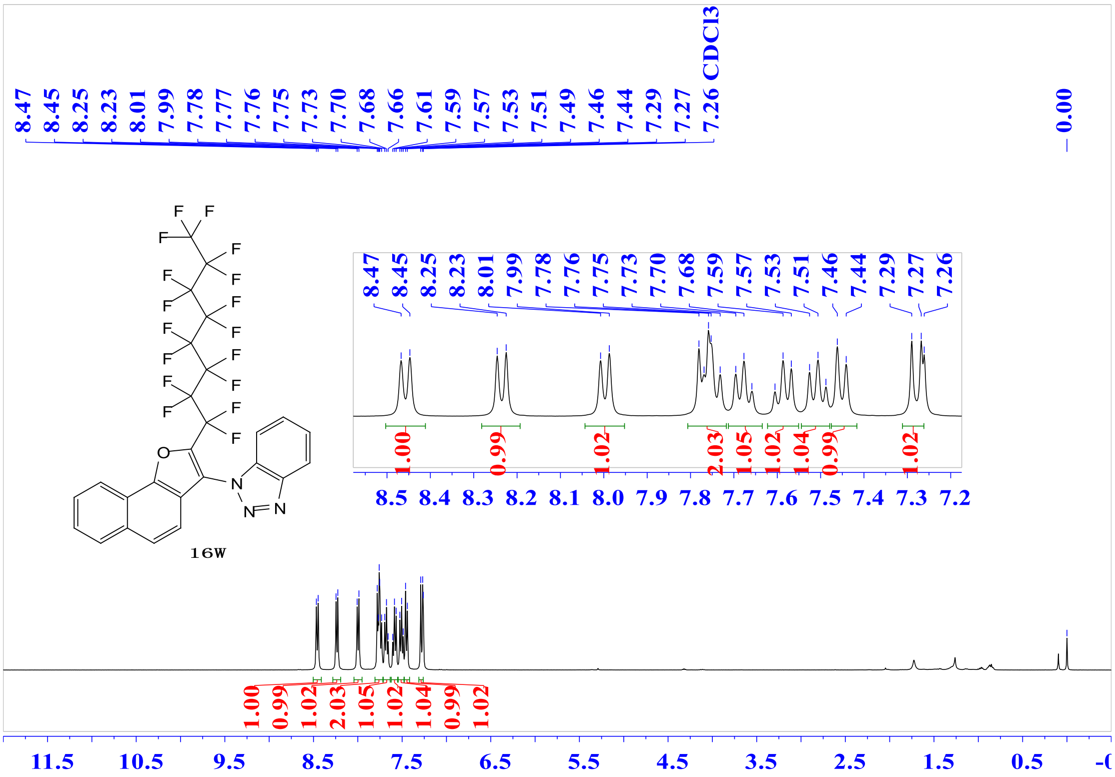

Figure S200. ^{19}F NMR spectrum of **16w**, related to **Scheme 3**.

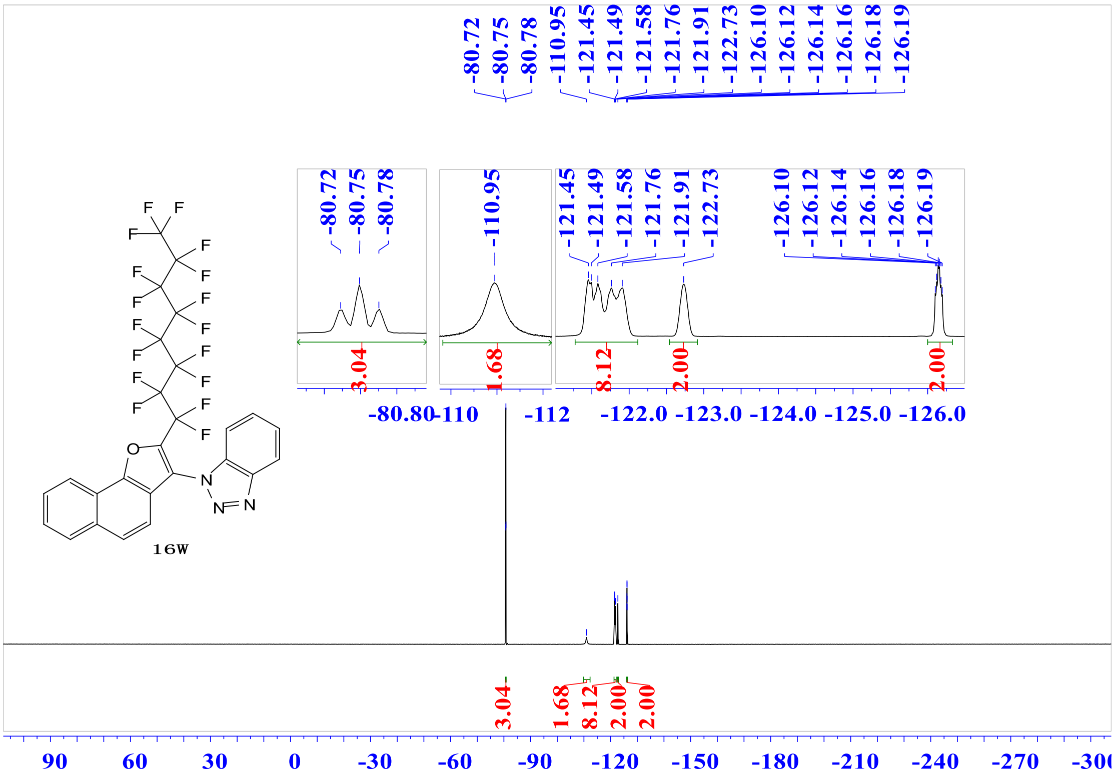

Figure S201. ^{13}C NMR spectrum of **16w**, related to **Scheme 3**.

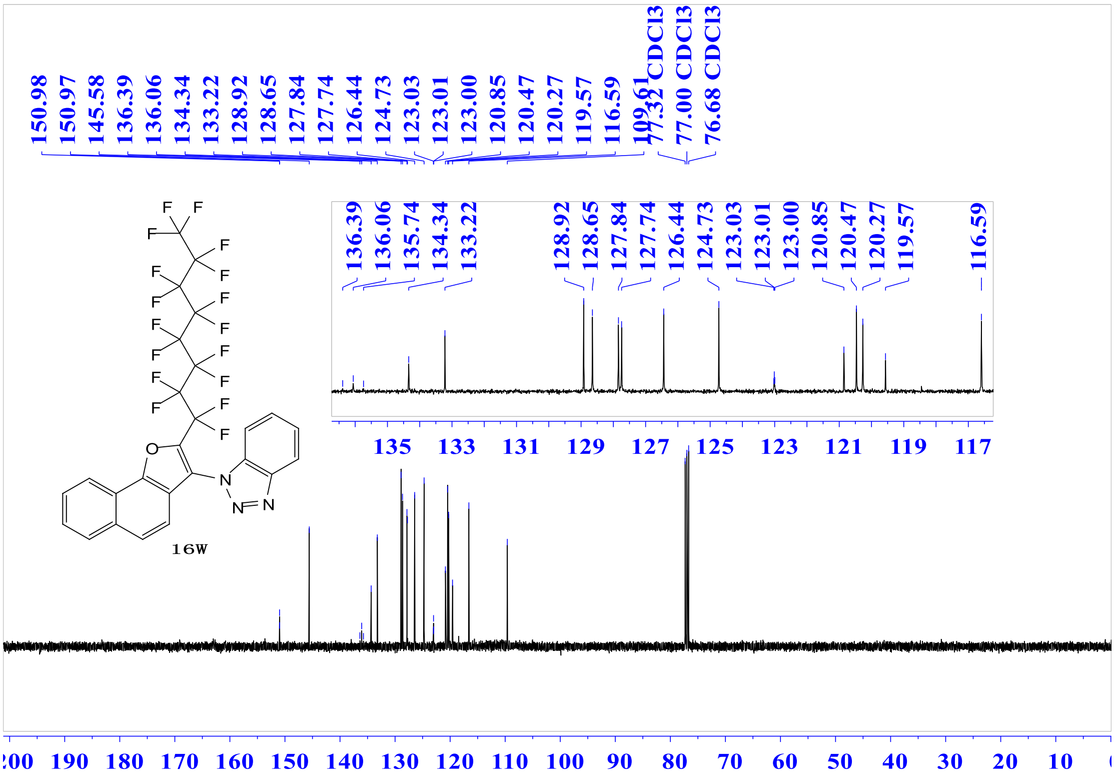

Figure S202. ^{1}H NMR spectrum of **16x**, related to **Scheme 3**.

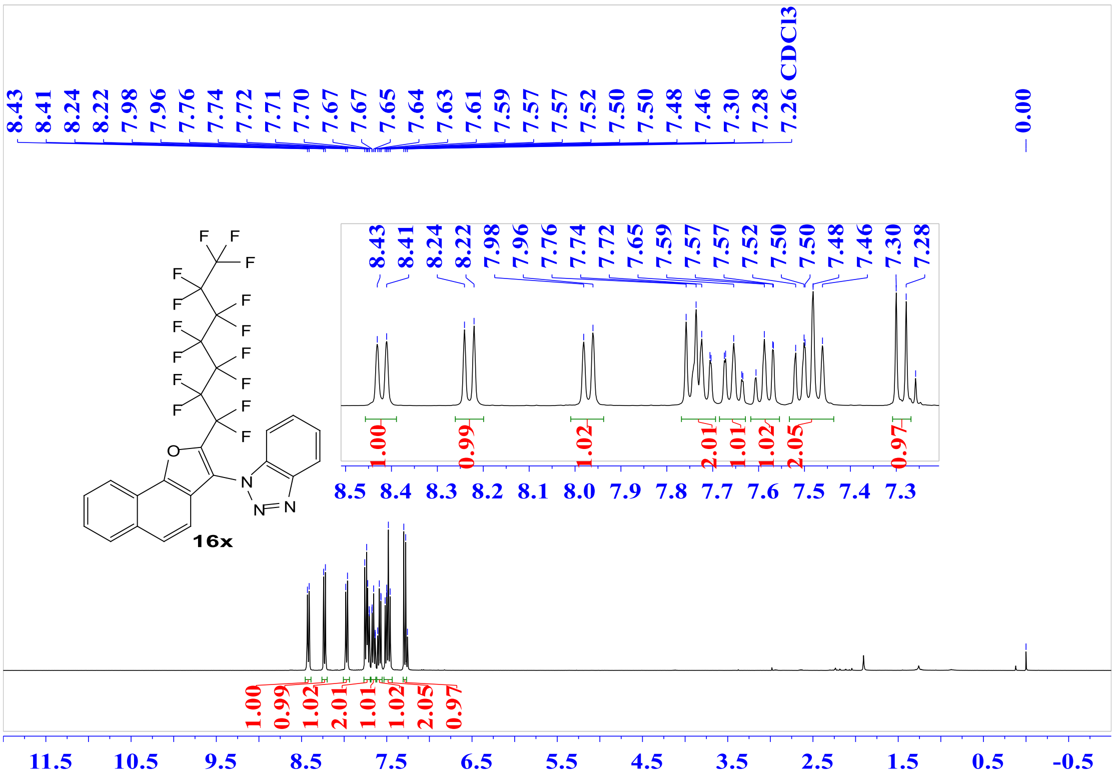

Figure S203. ^{19}F NMR spectrum of **16x**, related to **Scheme 3**.

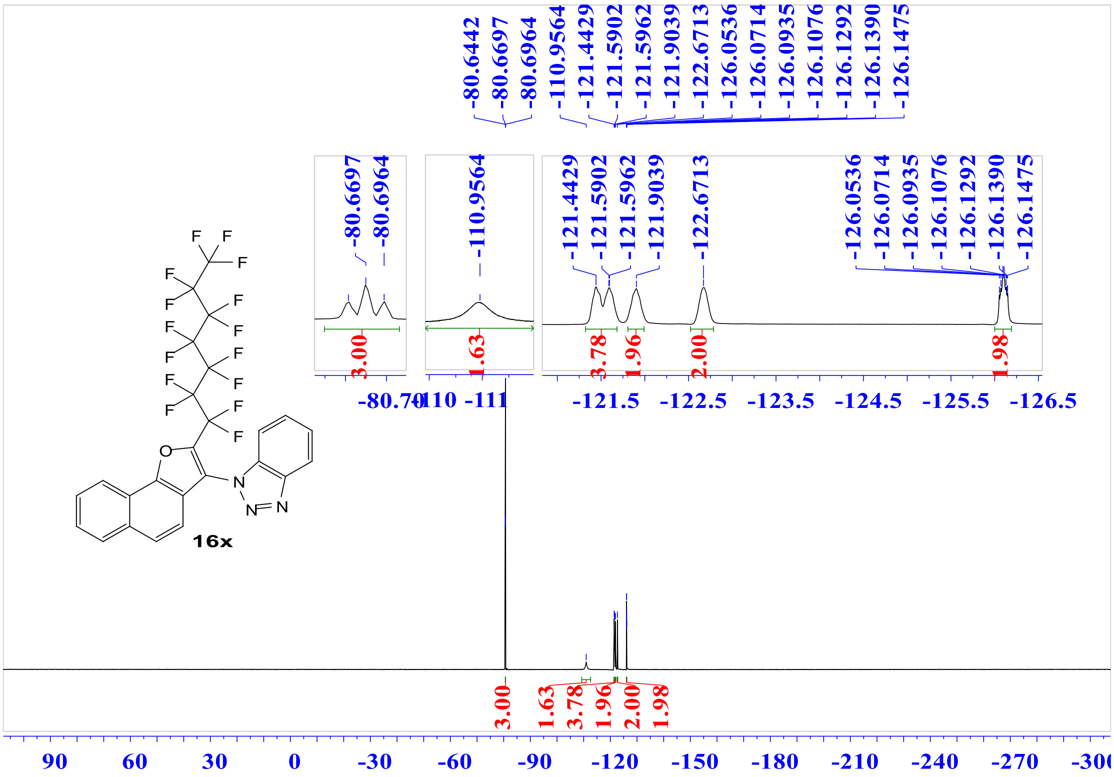

Figure S204. ^{13}C NMR spectrum of **16x**, related to **Scheme 3**.

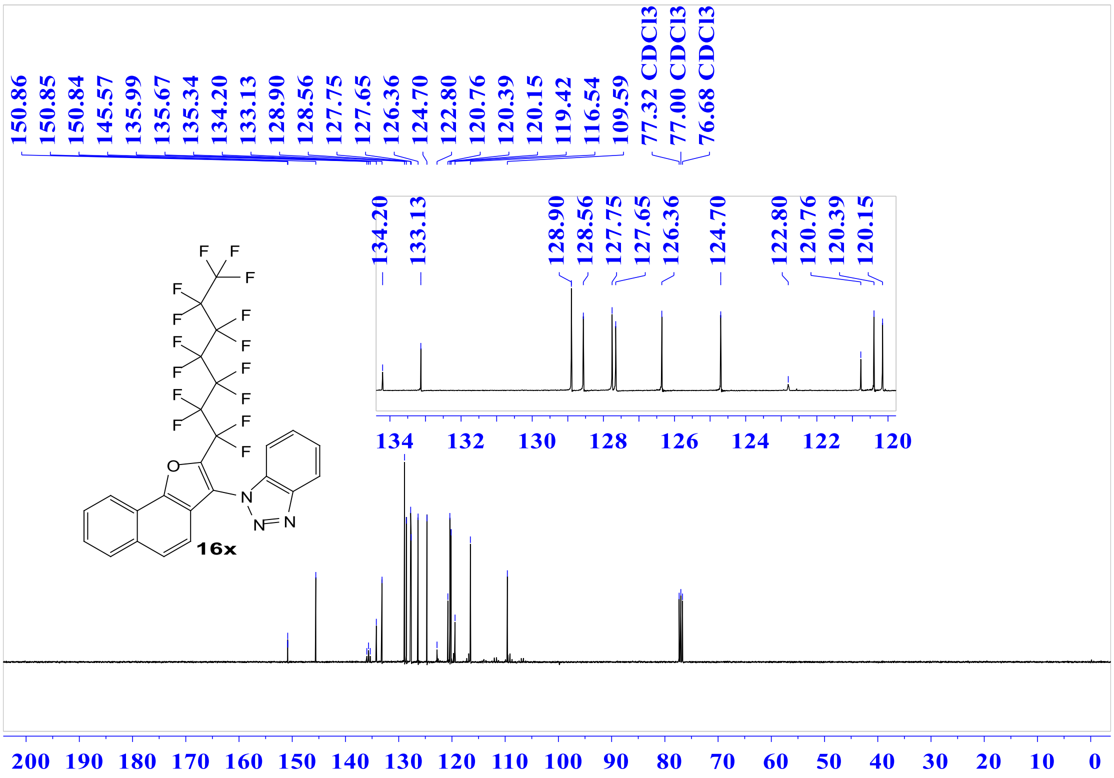

Figure S205. ^{1}H NMR spectrum of **16y**, related to **Scheme 3**.

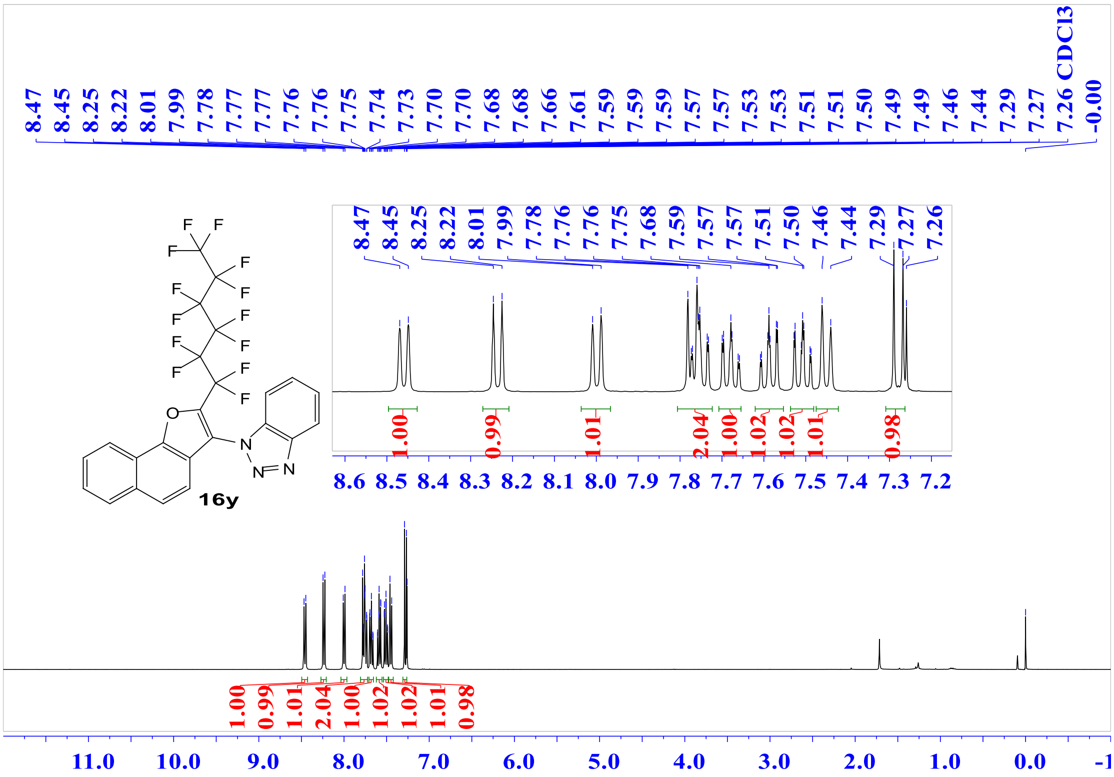

Figure S206. ^{19}F NMR spectrum of **16y**, related to **Scheme 3**.

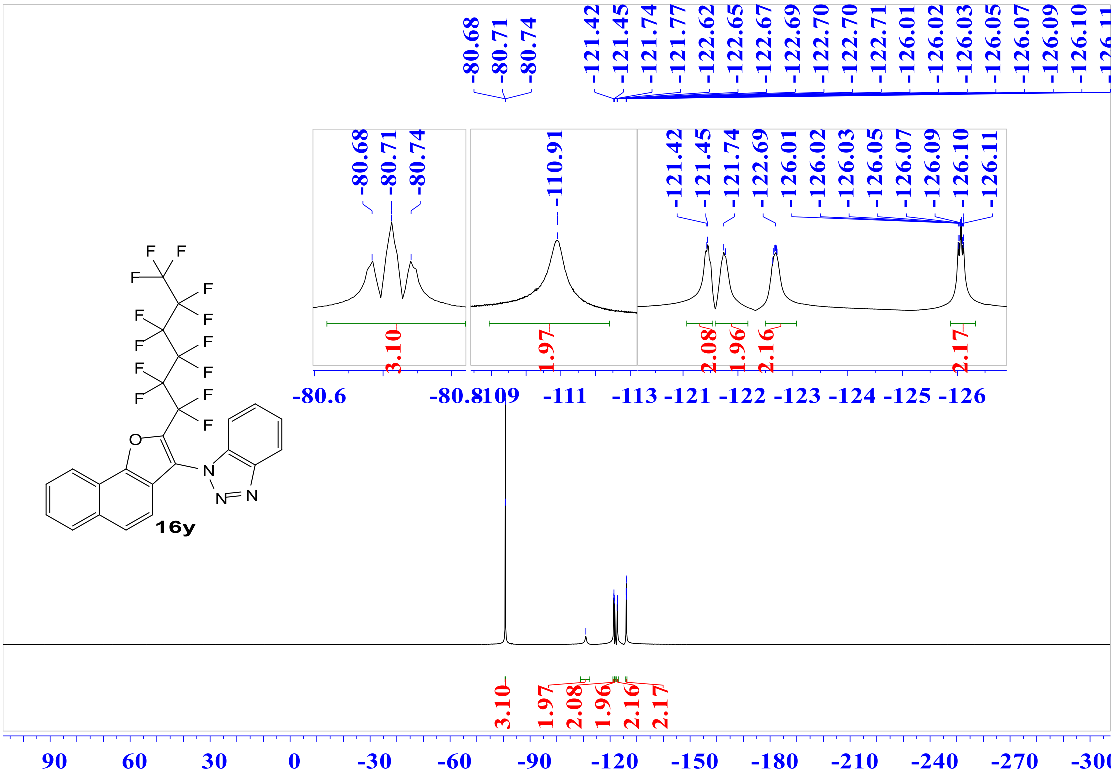

Figure S207. ^{13}C NMR spectrum of **16y**, related to **Scheme 3**.

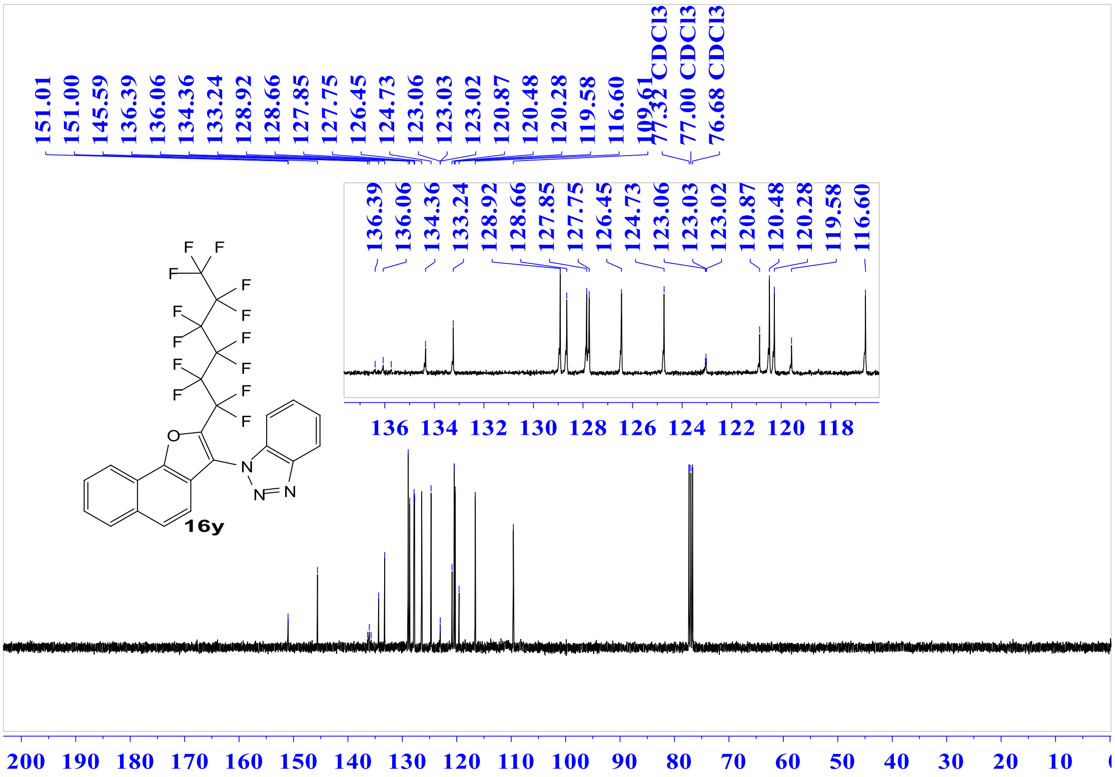

Figure S208. ^{1}H NMR spectrum of **16z**, related to **Scheme 3**.

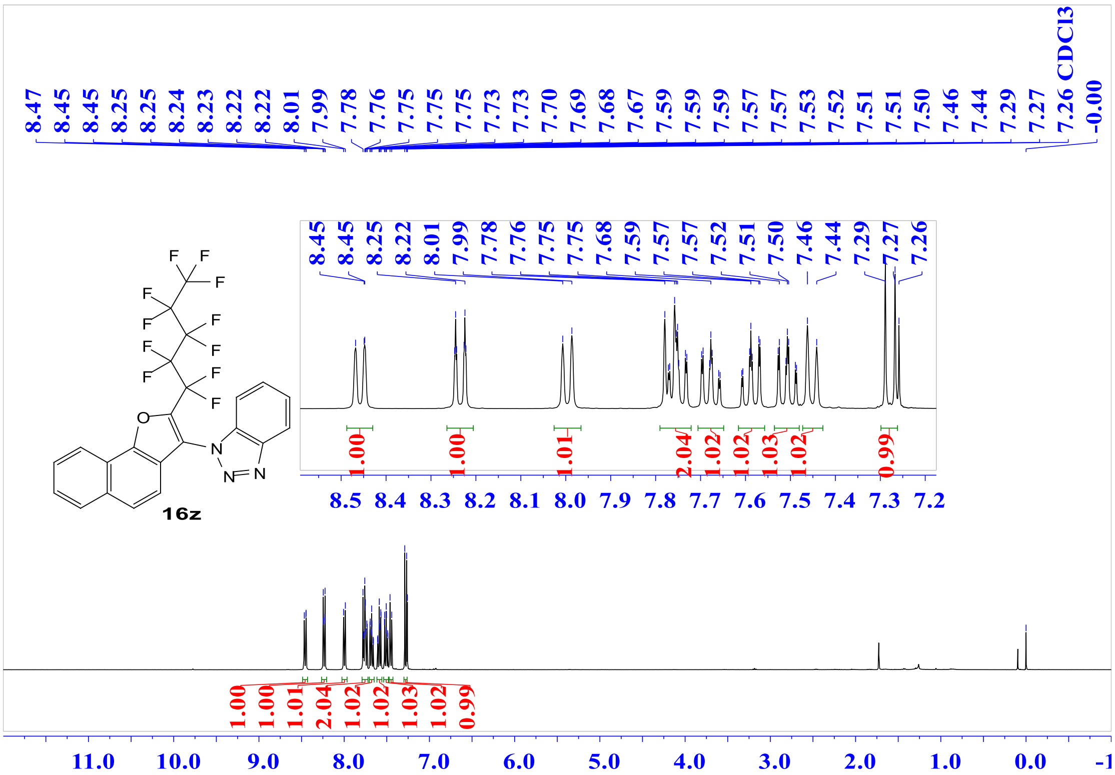

Figure S209. ^{19}F NMR spectrum of **16z**, related to **Scheme 3**.

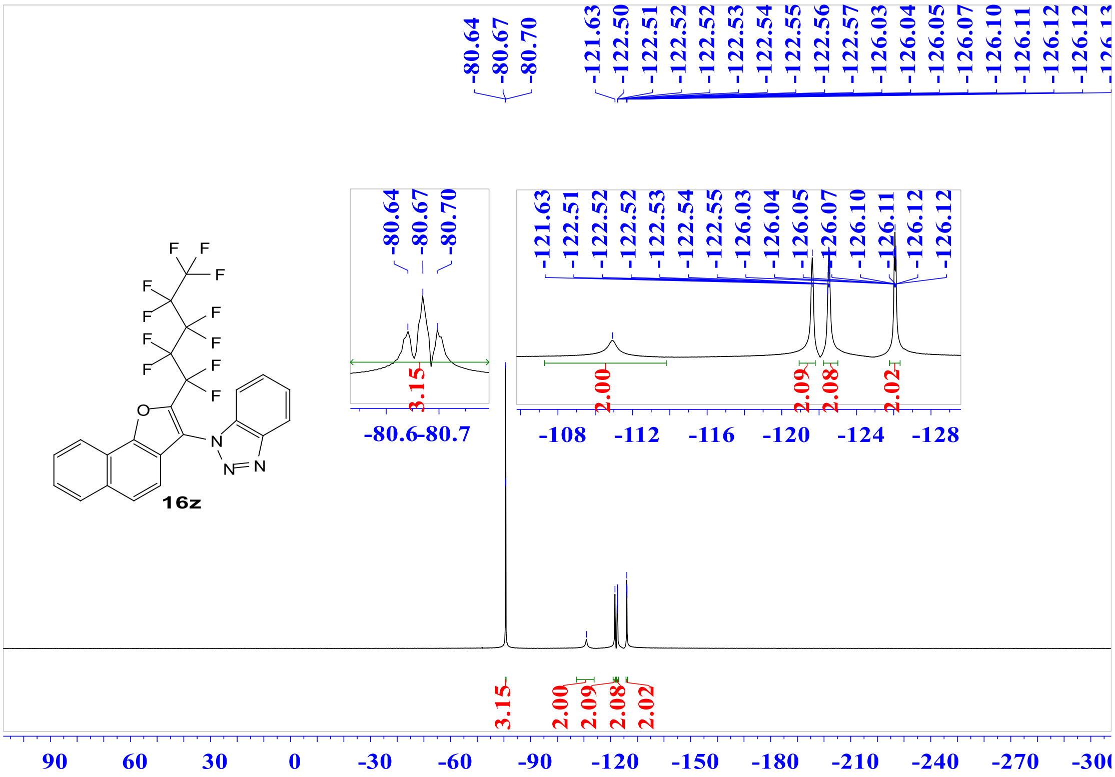

Figure S210. ^{13}C NMR spectrum of **16z**, related to **Scheme 3**.

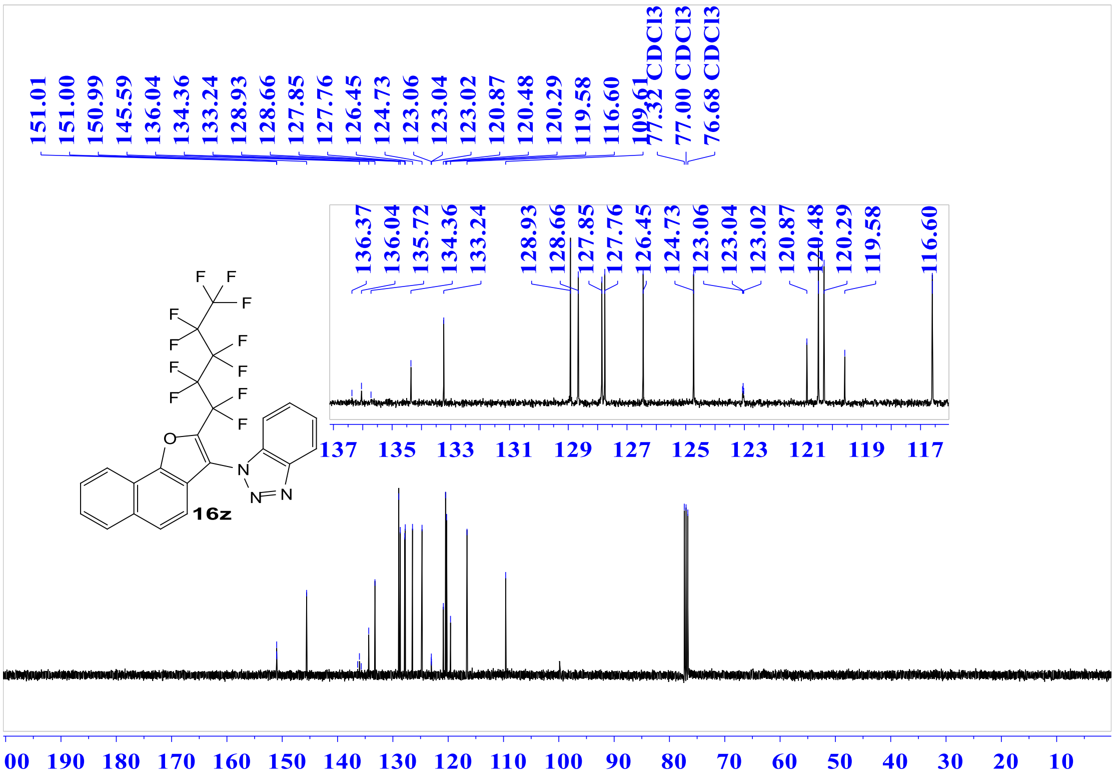

Figure S211. ^{1}H NMR spectrum of **16a'**, related to **Scheme 3**.

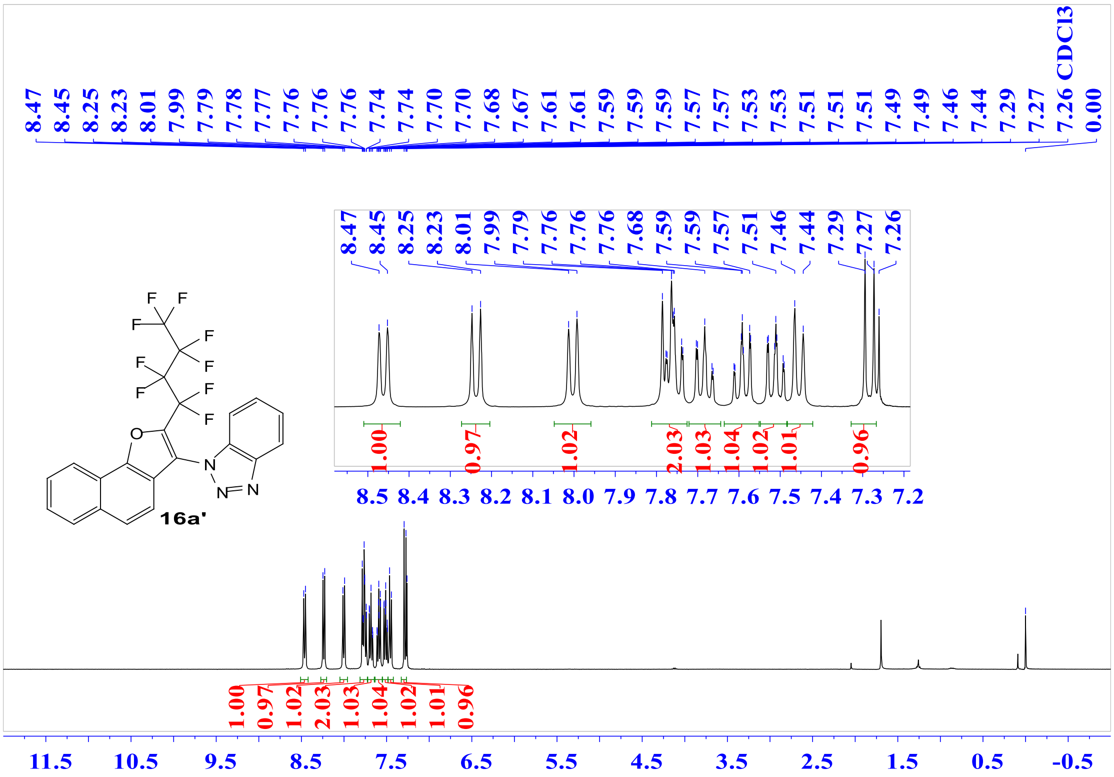

Figure S212. ^{19}F NMR spectrum of **16a'**, related to **Scheme 3**.

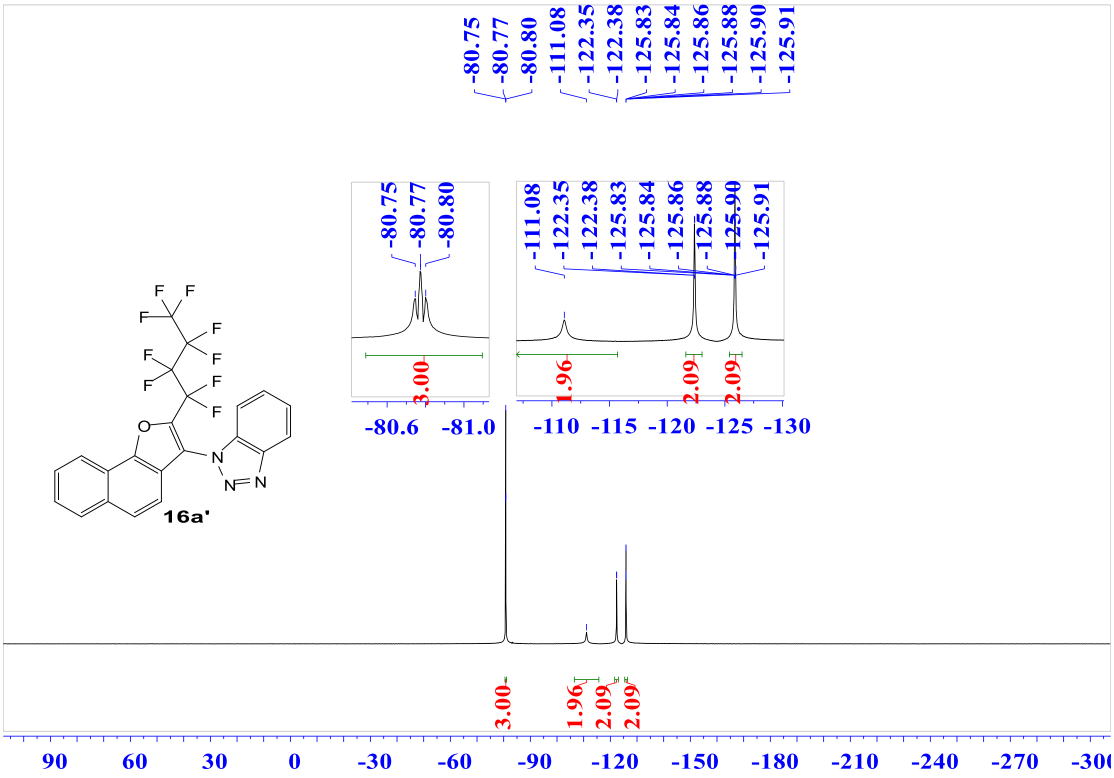

Figure S213. ^{13}C NMR spectrum of **16a’**, related to **Scheme 3**.

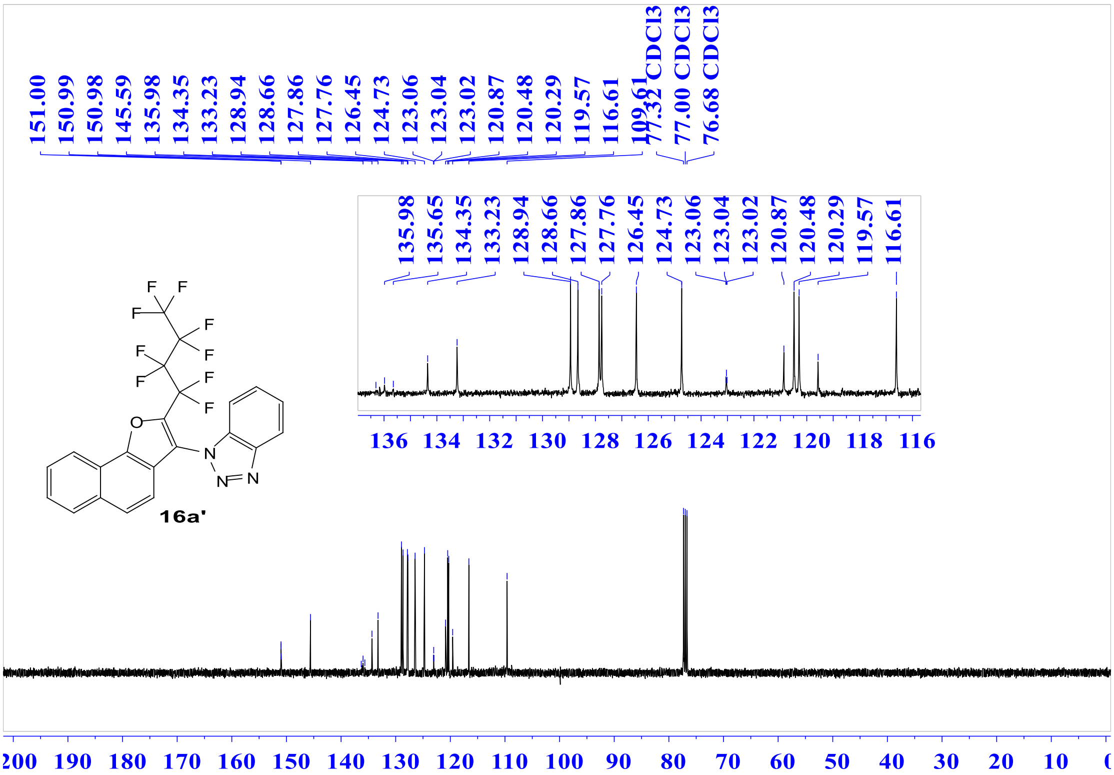

Figure S214. ^{1}H NMR spectrum of **16b’**, related to **Scheme 3**.

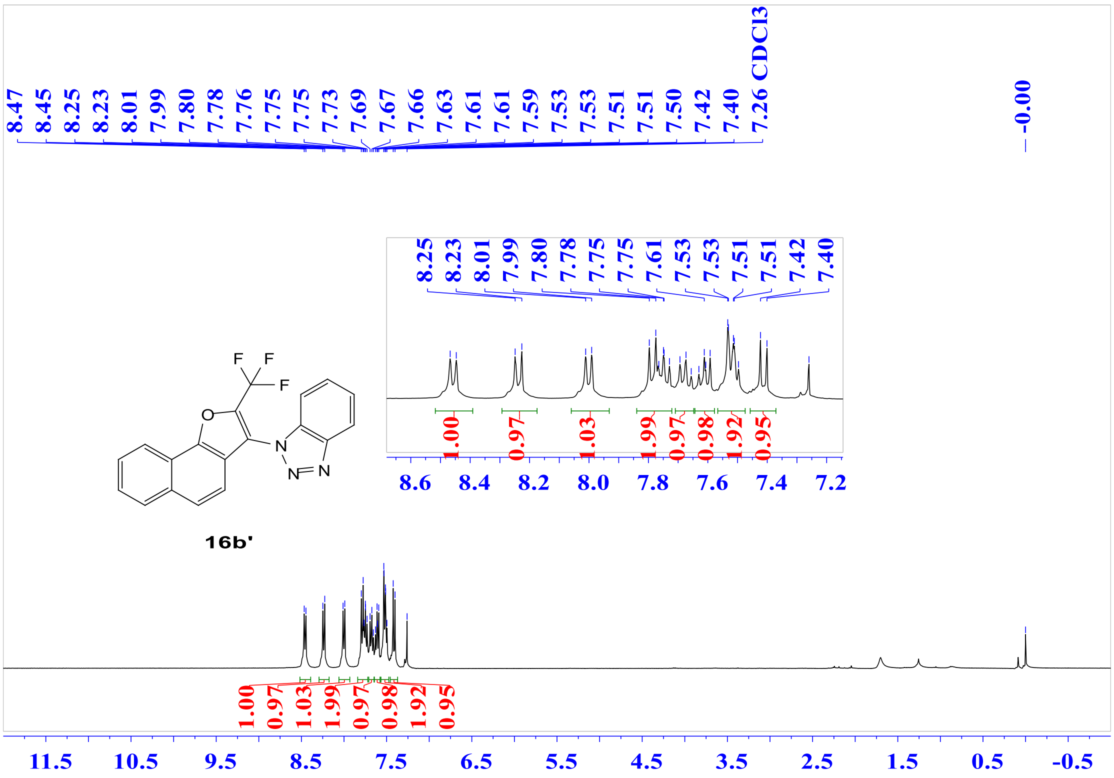

Figure S215. ^{19}F NMR spectrum of **16b'**, related to **Scheme 3**.

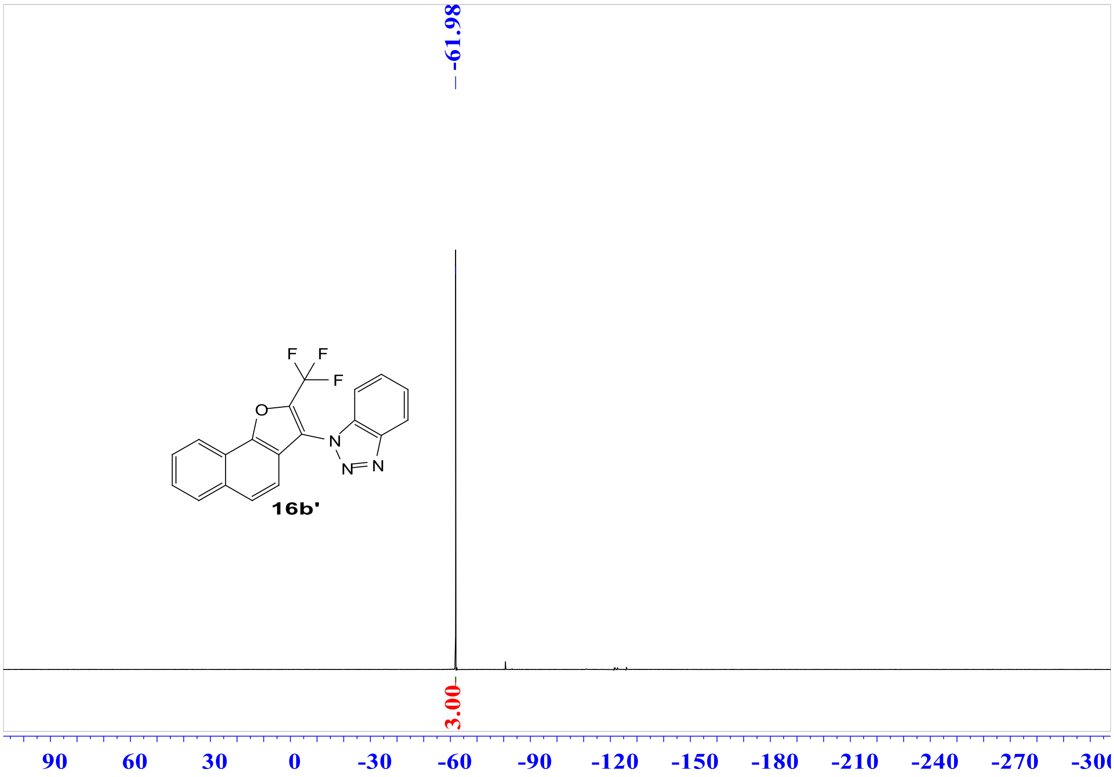

Figure S216. ^{13}C NMR spectrum of **16b'**, related to **Scheme 3**.

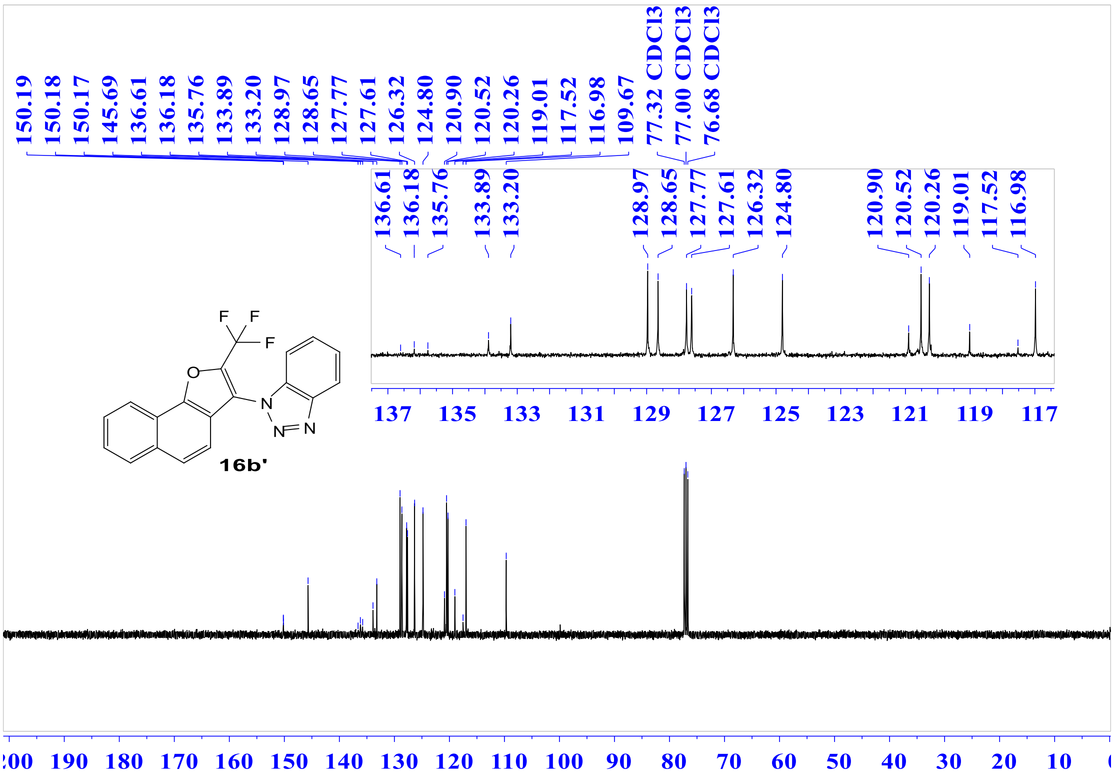

Figure S217. ^{1}H NMR spectrum of **17**, related to **Scheme 4**.

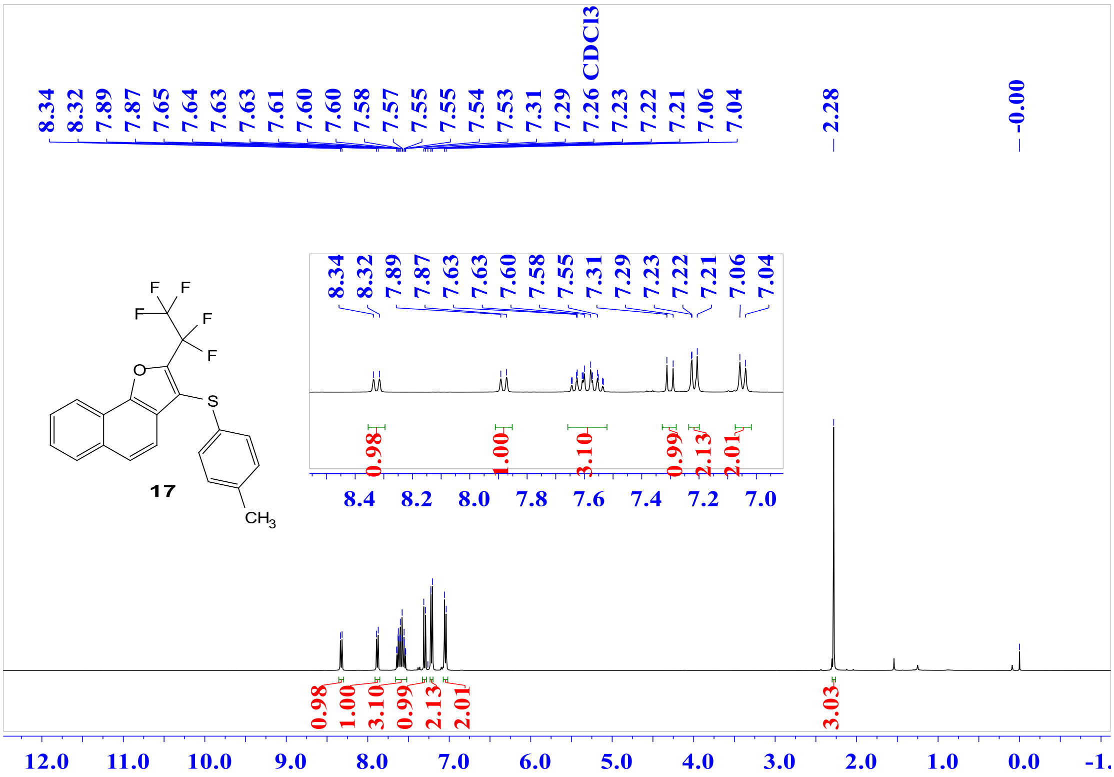

Figure S218. ^{19}F NMR spectrum of **17**, related to **Scheme 4**.

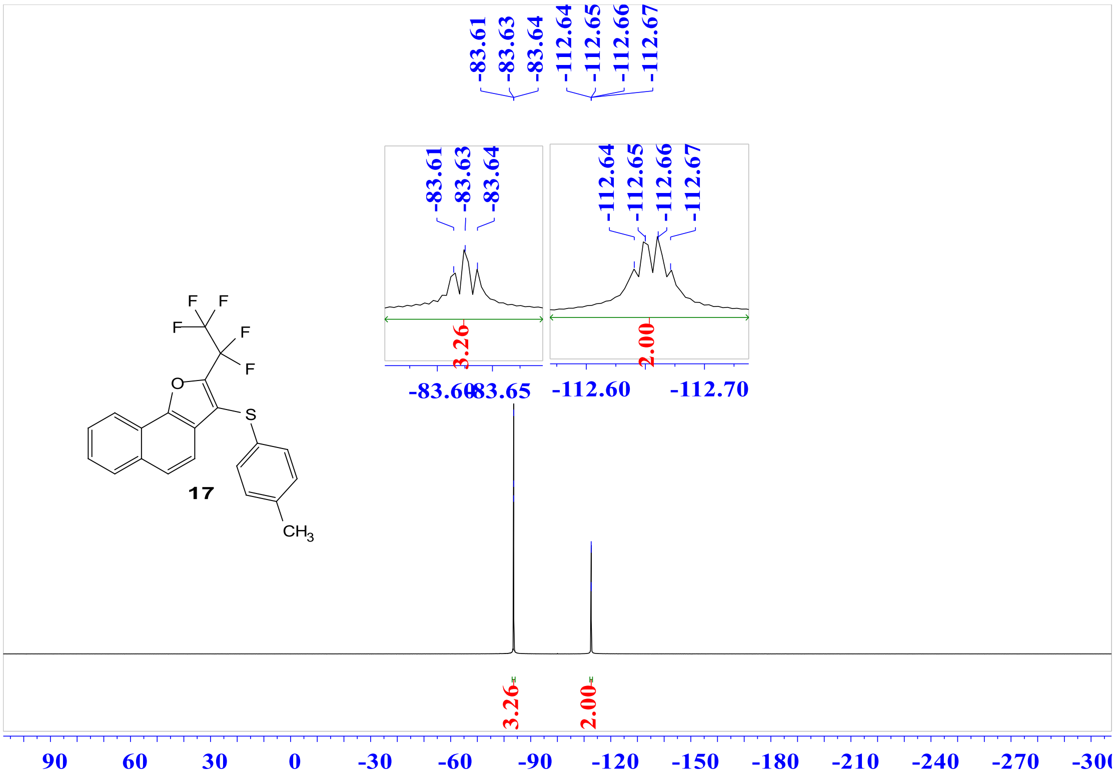

Figure S219. ^{13}C NMR spectrum of **17**, related to **Scheme 4**.

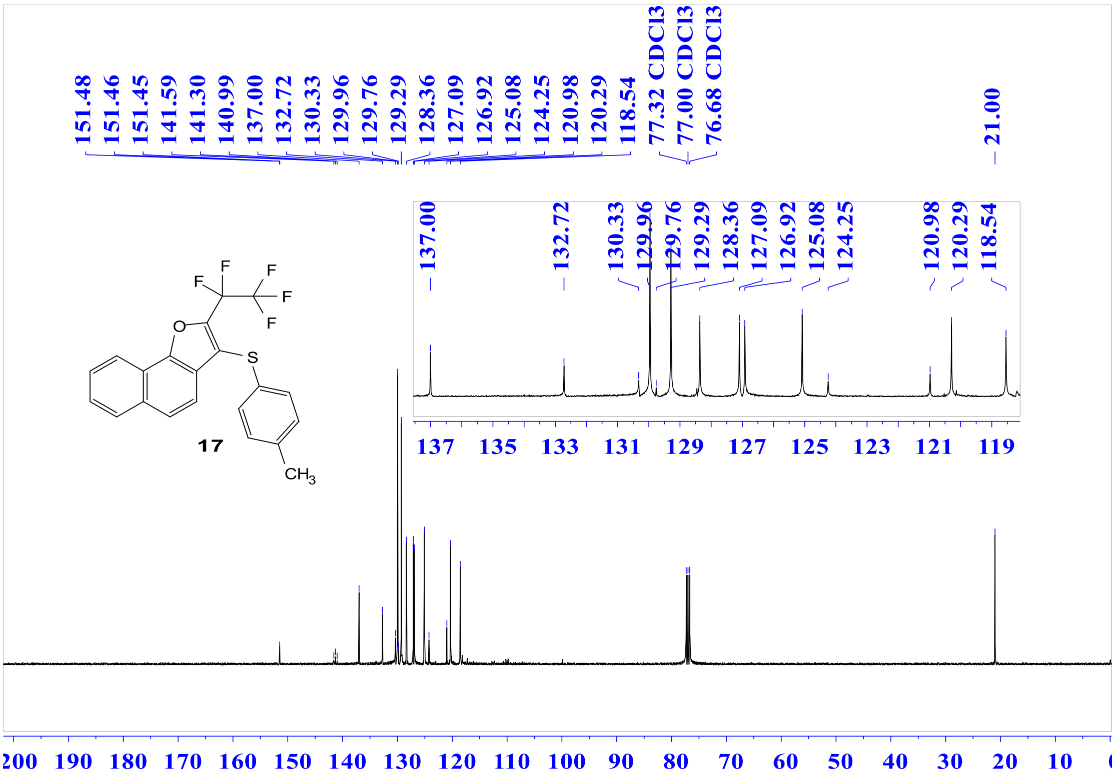

Figure S220. ^{1}H NMR spectrum of **18**, related to **Scheme 4**.

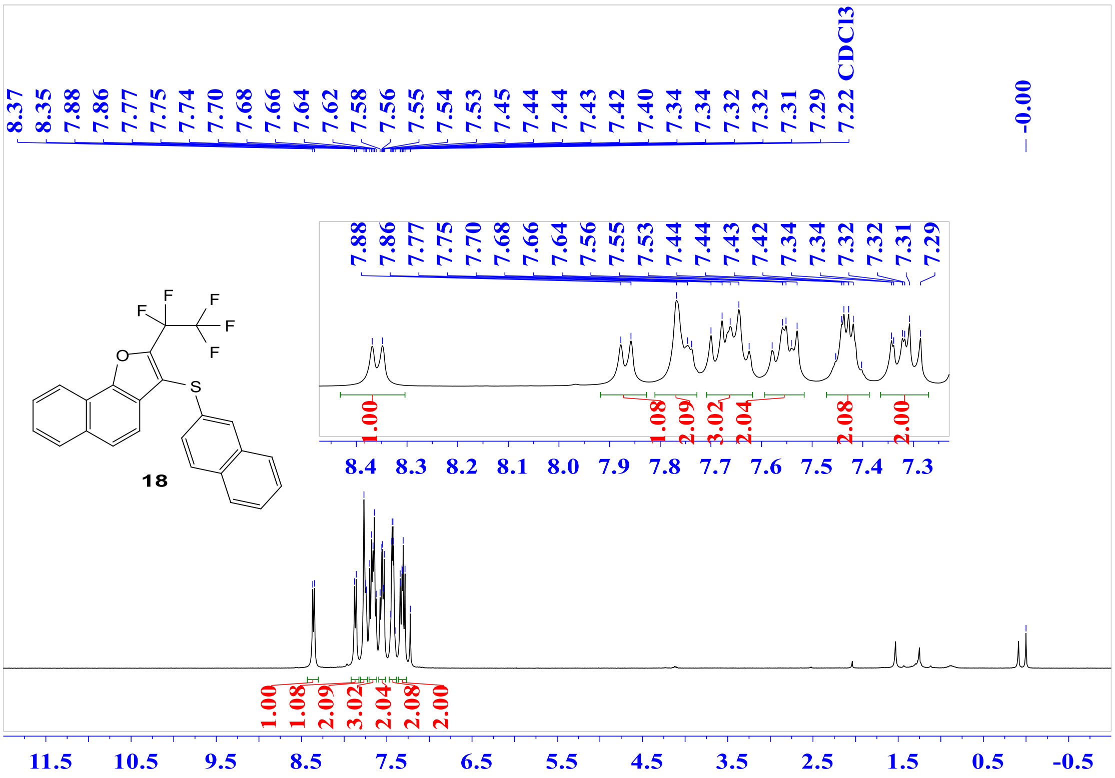

Figure S221. ^{19}F NMR spectrum of **18**, related to **Scheme 4**.

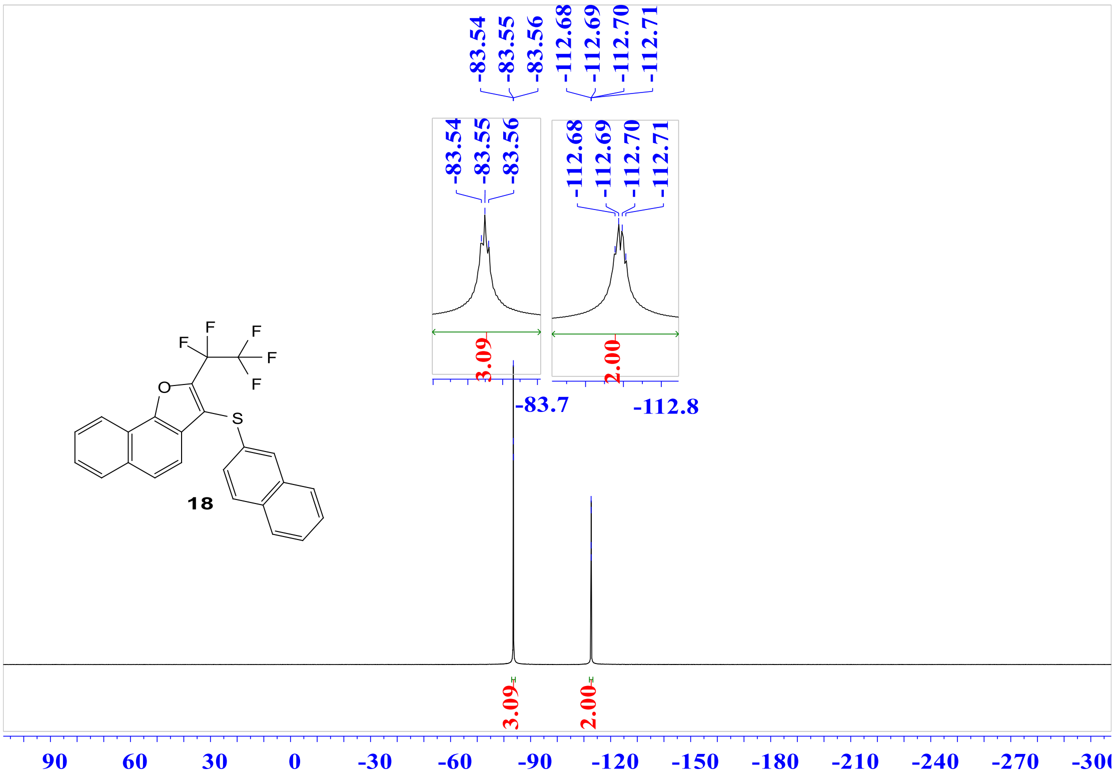

Figure S222. ^{13}C NMR spectrum of **18**, related to **Scheme 4**.

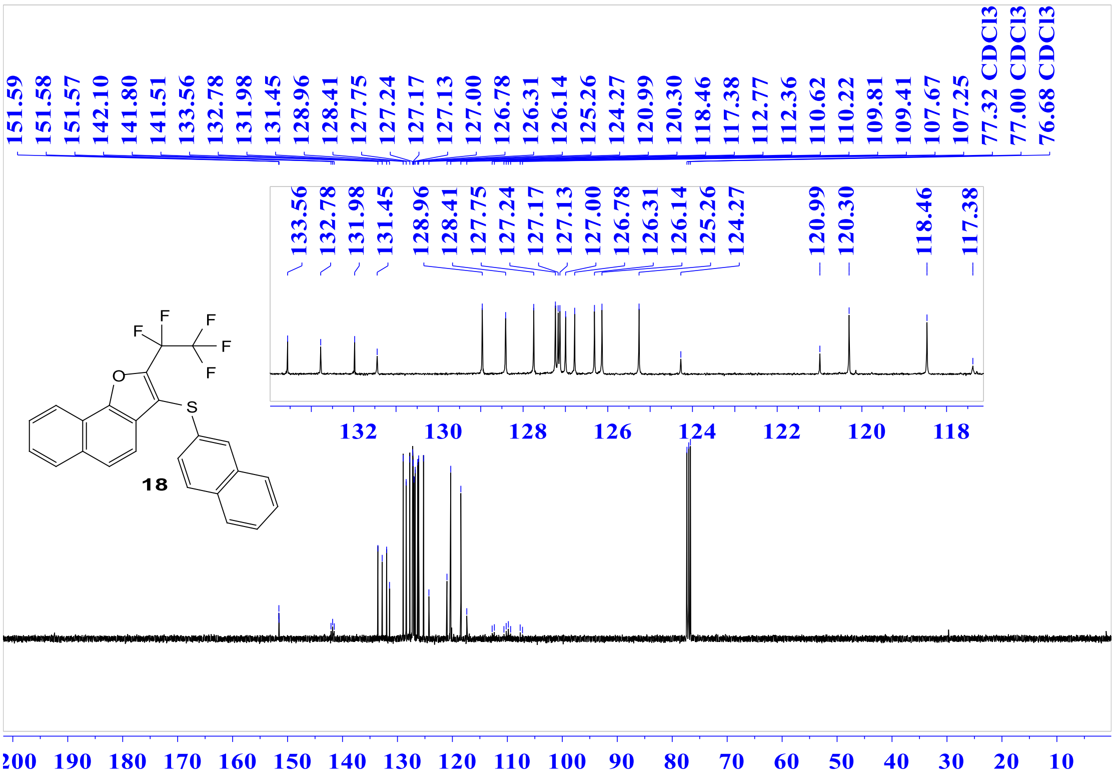

Figure S223. ^{1}H NMR spectrum of **19**, related to **Scheme 4**.

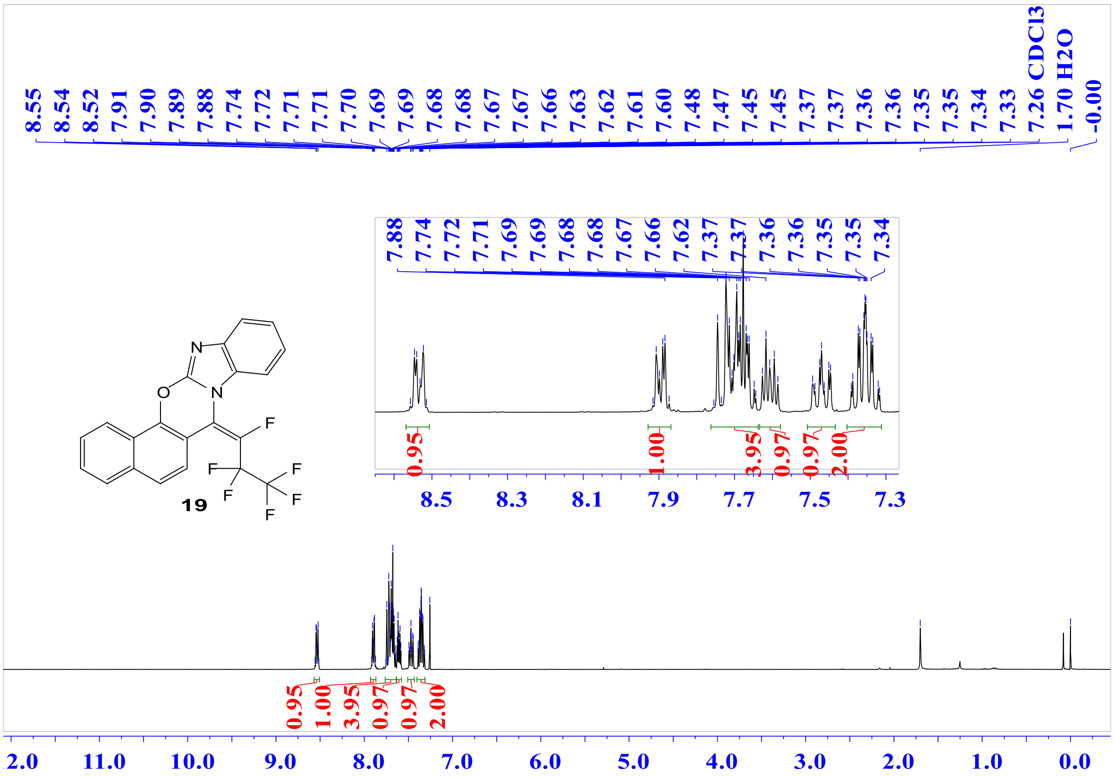

Figure S224. ^{19}F NMR spectrum of **19**, related to **Scheme 4**.

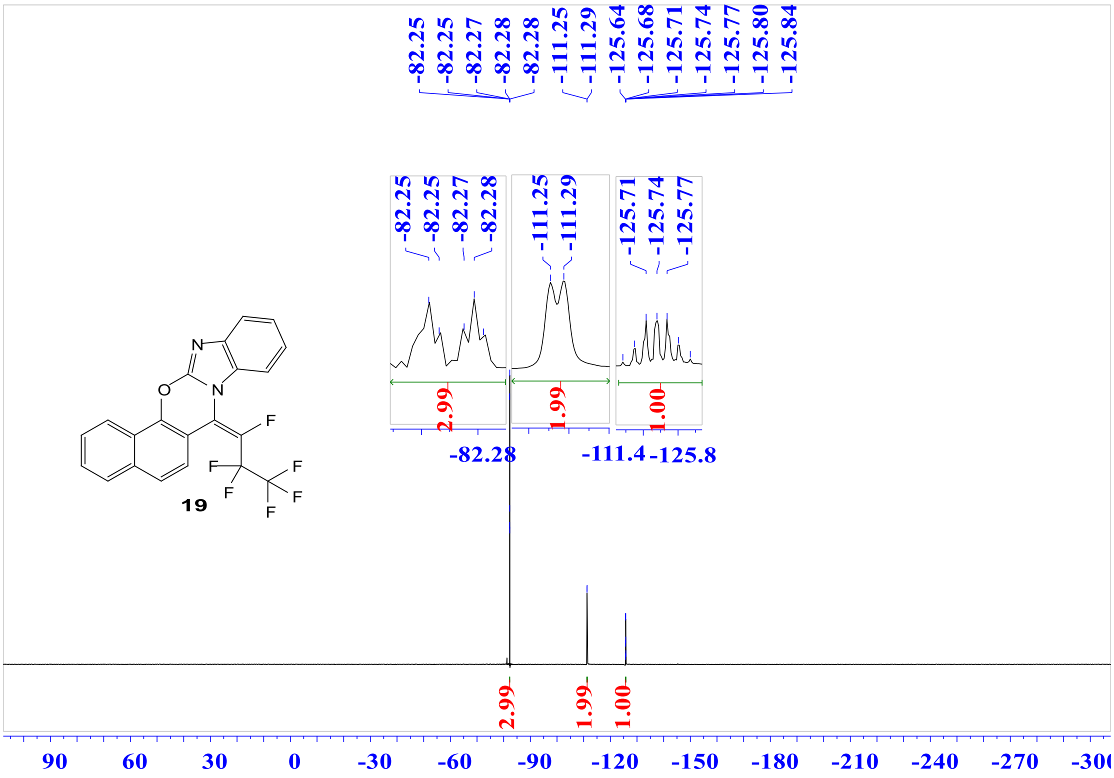

Figure S225. ^{13}C NMR spectrum of **19**, related to **Scheme 4**.

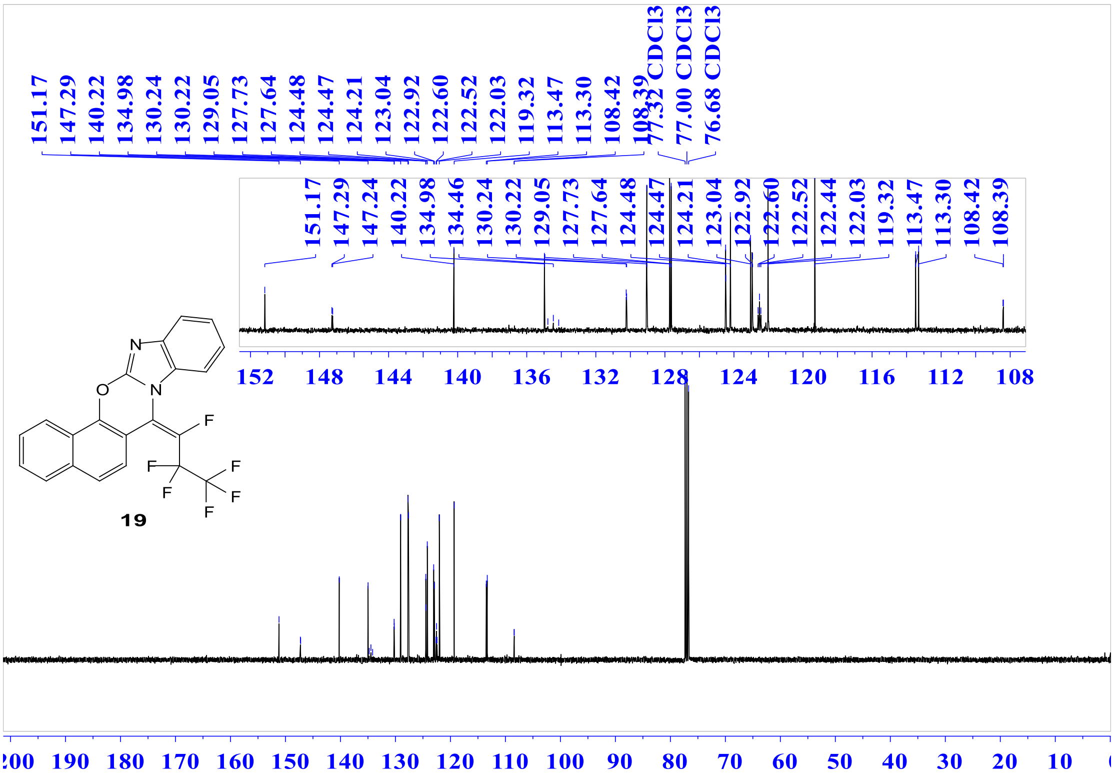

Figure S226. ^{1}H NMR spectrum of **20**, related to **Scheme 4**.

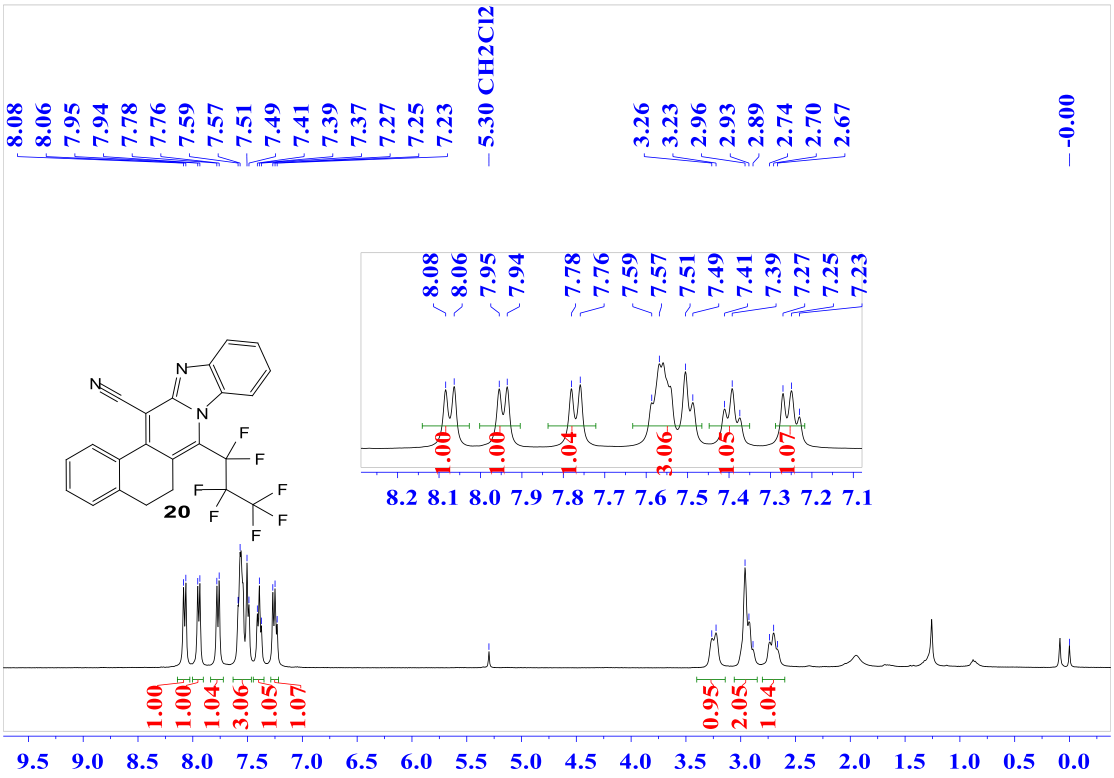

Figure S227. ^{19}F NMR spectrum of **20**, related to **Scheme 4**.

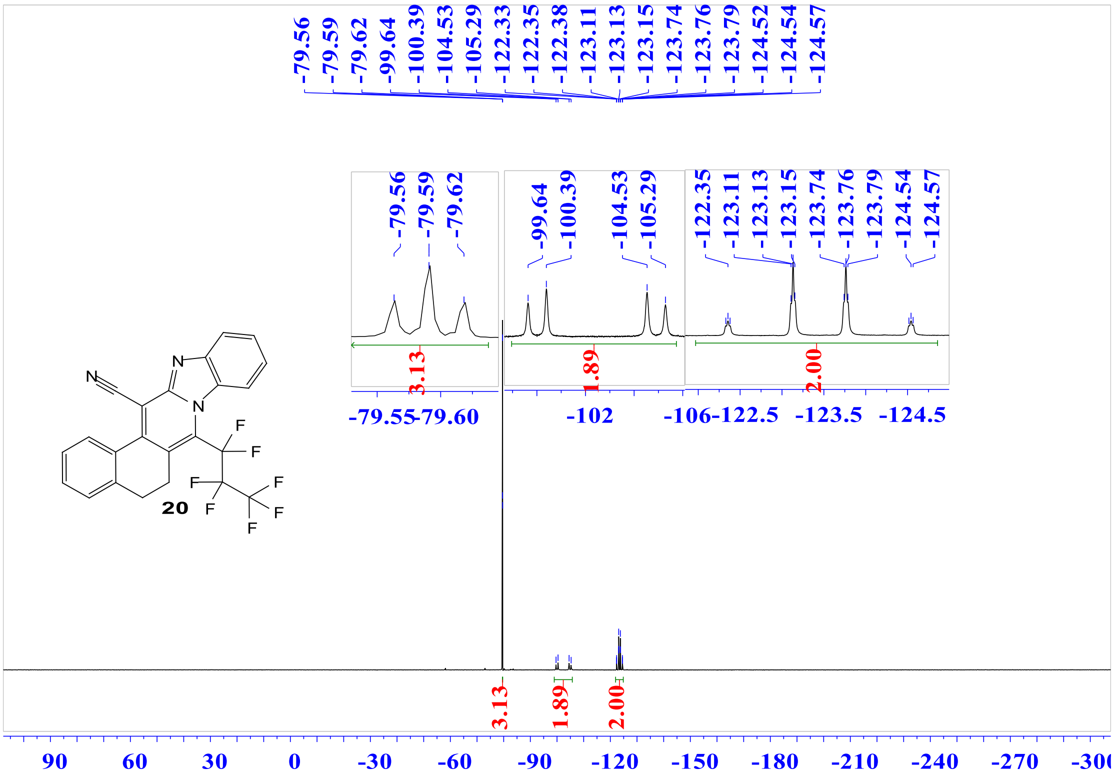

Figure S228. ^{13}C NMR spectrum of **20**, related to **Scheme 4**.

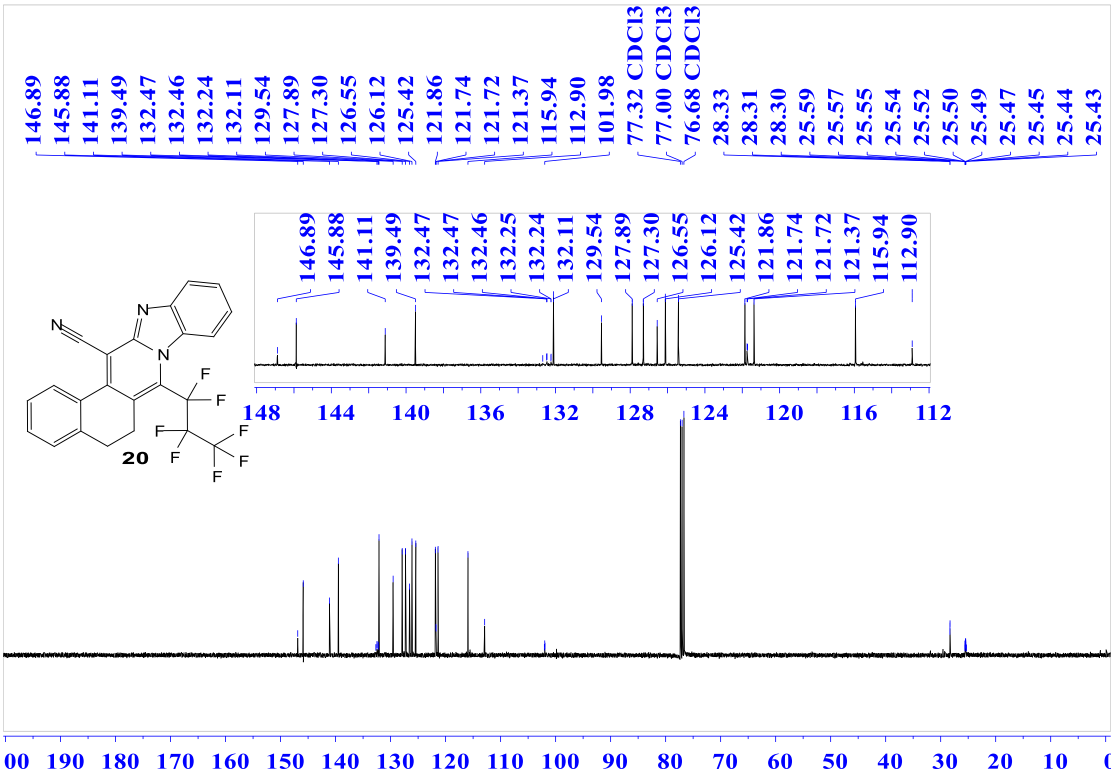

Figure S229. ^{1}H NMR spectrum of **21**, related to **Scheme 4**.

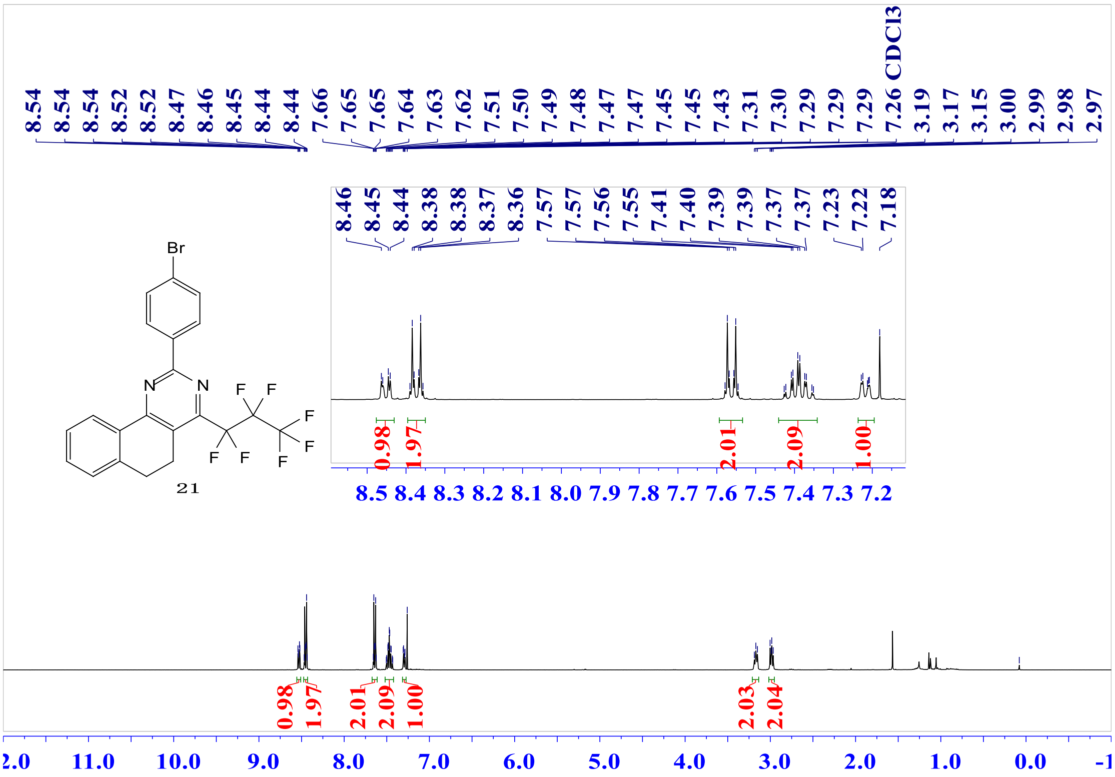

Figure S230. ^{19}F NMR spectrum of **21**, related to **Scheme 4**.

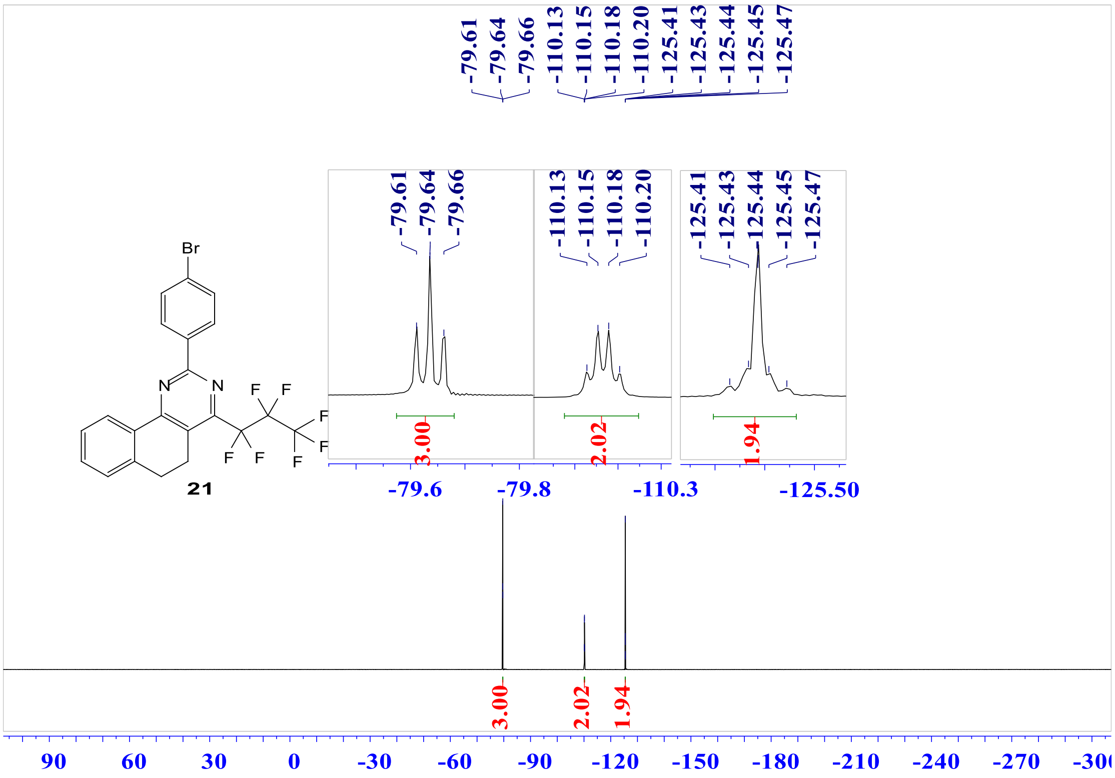

Figure S231. ^{13}C NMR spectrum of **21**, related to **Scheme 4**.

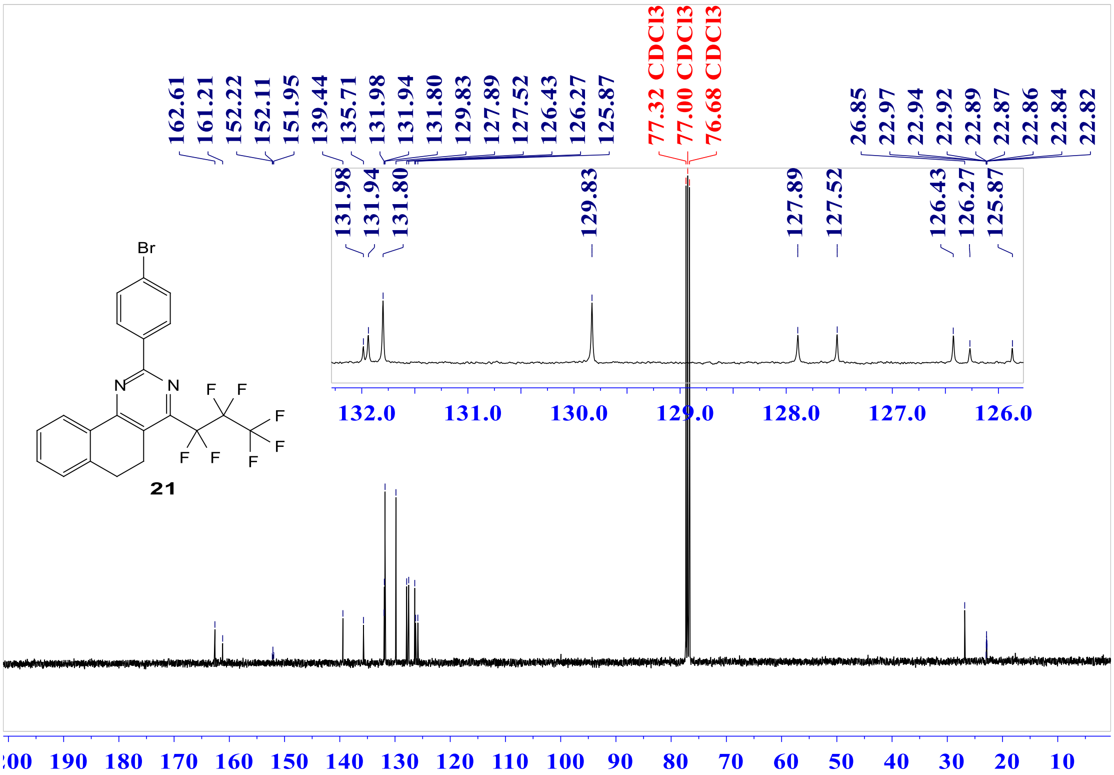

Supplemental figures for X-Ray structures

Figure S232. X-Ray crystal data of **6b**, related to **Figure 2**.

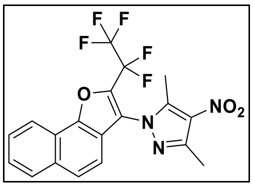

Compound: **3,5-dimethyl-4-nitro-1-(2-(perfluoroethyl)naphtho[1,2-b]furan-3-yl)-1H-pyrazole (6b)**

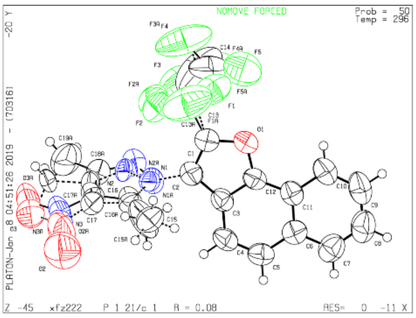

Crystal Number: CCDC 1881997

Chemical Formula: $C_{19}H_{12}F_5N_3O_3$

Formula weight: 425.3150

Space Group: P 21/c

Cell: a = 18.612(4) b = 13.533(3) c = 7.5759(17)

Figure S233. X-Ray crystal data of **19**, related to **Figure 2**.

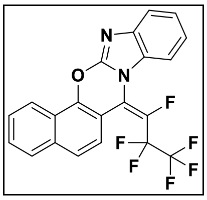

Compound: **(*Z*)-7-(perfluoropropylidene)-7*H*-benzo[4,5]imidazo[2,1-*b*]naphtho[2,1-*e*][1,3]oxazine (19)**

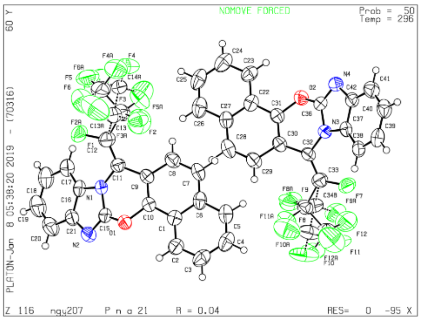

Crystal Number: CCDC 1881996

Chemical Formula: $C_{21}H_{10}F_6N_2O$

Formula weight: 420.3144

Space Group: P n a 21

Cell: a = 8.920(8) b = 12.280(11) c = 32.31(3)

Notice: these structures were collected at room temperature, where the disorder in the fluorinated groups is severe and can hardly be modelled.

Supplemental figures for computational details and discussion

All calculations were performed with the Gaussian 09 package (Frischet al., 2013). Geometry optimizations and frequency calculations were performed at the UB3LYP (Becke, 1988; Lee et al., 1988; Becke, 1993)/def2-SVP level (Weigend et al., 2005) in conjunction with the polarizable continuum model (PCM) (Tomasi et al. 1994) to account for the solvation effects of dimethyl sulfoxide (DMSO). To get more accurate energies, single point energies were computed at the UB3LYP/def2-TZVP (Weigend et al., 2005) level combined with Grimme's DFT empirical dispersion correction (DFT-D3) for all the species (Grimme et al., 2010; Goerigk et al., 2011). The 3D structures of the optimized species were generated using CYLview (Legault, 2009). Activation free energy barriers reported here are defined as the free energy difference between the transition state and the lowest-energy stationary point or separated reactants before it along the reaction pathways.

Figure S234. 3D structures of the species involved in the Cs_2CO_3-mediated four C(sp^3)-F bonds cleavage and C-N/O coupling reaction of **1c′** and **2a**, related to **Figure 3** and **4**.

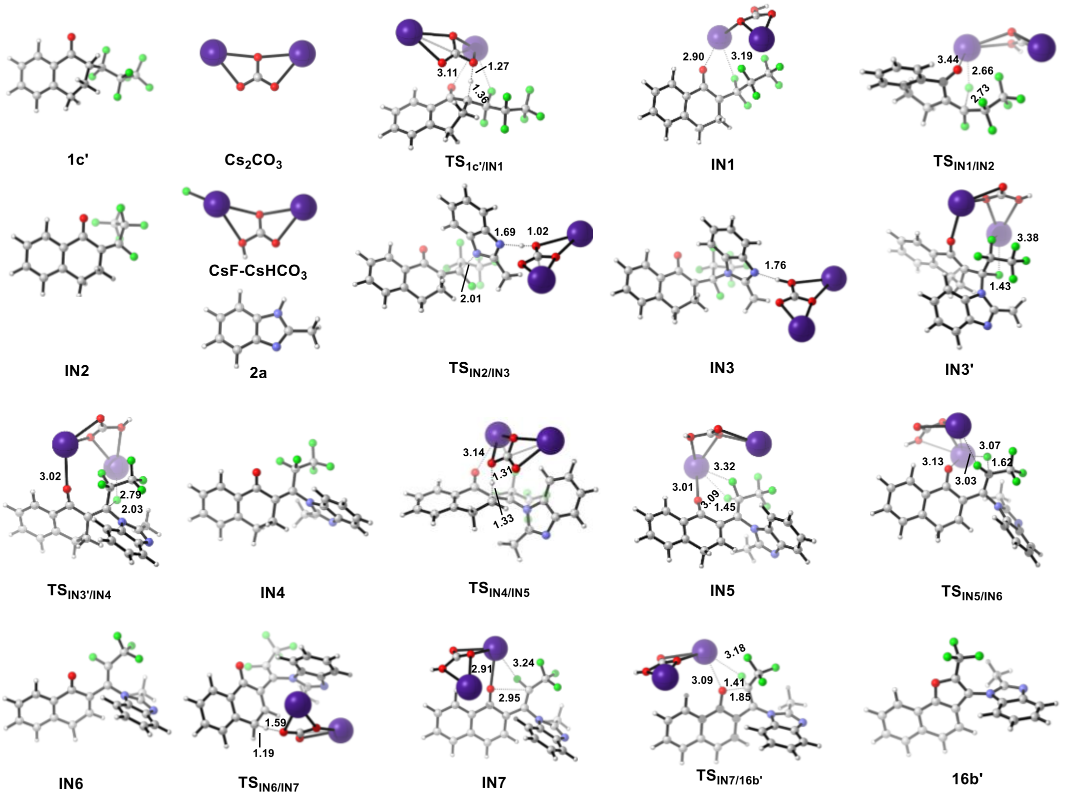

Figure S235. Transition state structures of Cs_2CO_3-mediated nucleophilic addition of **2a** to **IN2** without or with $CoBr_2$, related to **Figure 3** and **4**.

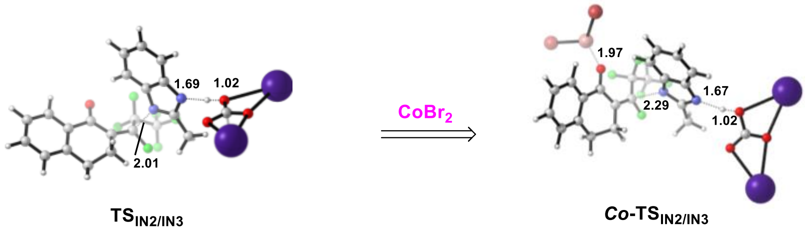

TS$_{IN2/IN3}$ $\triangle G^{\ddagger}$ = 15.2 kcal/mol

***Co*-TS$_{IN2/IN3}$** $\triangle G^{\ddagger}$ = 5.9 kcal/mol

Figure S236. Transition state structures of Cs_2CO_3-mediated intramolecular cyclization reaction of **IN7** without or with $CoBr_2$, related to **Figure 3** and **4**.

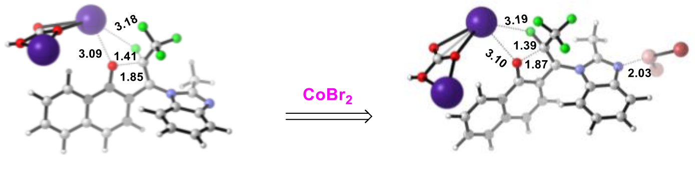

TS$_{IN7/16b'}$ $\triangle G^{\ddagger}$ = 25.6 kcal/mol

***Co*-TS$_{IN7/16b'}$** $\triangle G^{\ddagger}$ = 26.7 kcal/mol

Figure S237. Cs_2CO_3- and $CsHCO_3$-mediated intramolecular cyclization reaction of **IN7**, related to **Figure 3** and **4**.

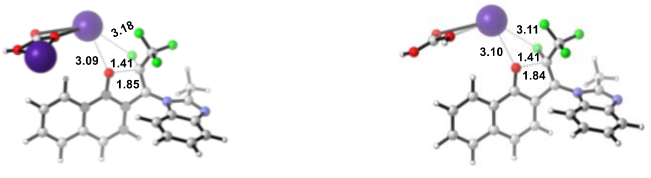

TS$_{IN7/16b'}$ (Cs_2CO_3) $\triangle G^{\ddagger}$ = 25.6 kcal/mol

TS$_{IN7/16b'}$' ($CsHCO_3$) $\triangle G^{\ddagger}$ = 25.9 kcal/mol

The $CsHCO_3$-mediated intramolecular cyclization reaction of **IN7** (rate-limiting step) was also investigated. As shown in Figure S237, the corresponding transition states, **TS$_{IN7/16b'}$** and **TS$_{IN7/16b'}$′**, have almost similar activation barriers with Cs_2CO_3 and $CsHCO_3$. This result indicates that the base does not have significant influence on this process.

Figure S238. **Various α-perfluoroalkyl ketones**, related to **Scheme 1-5**.

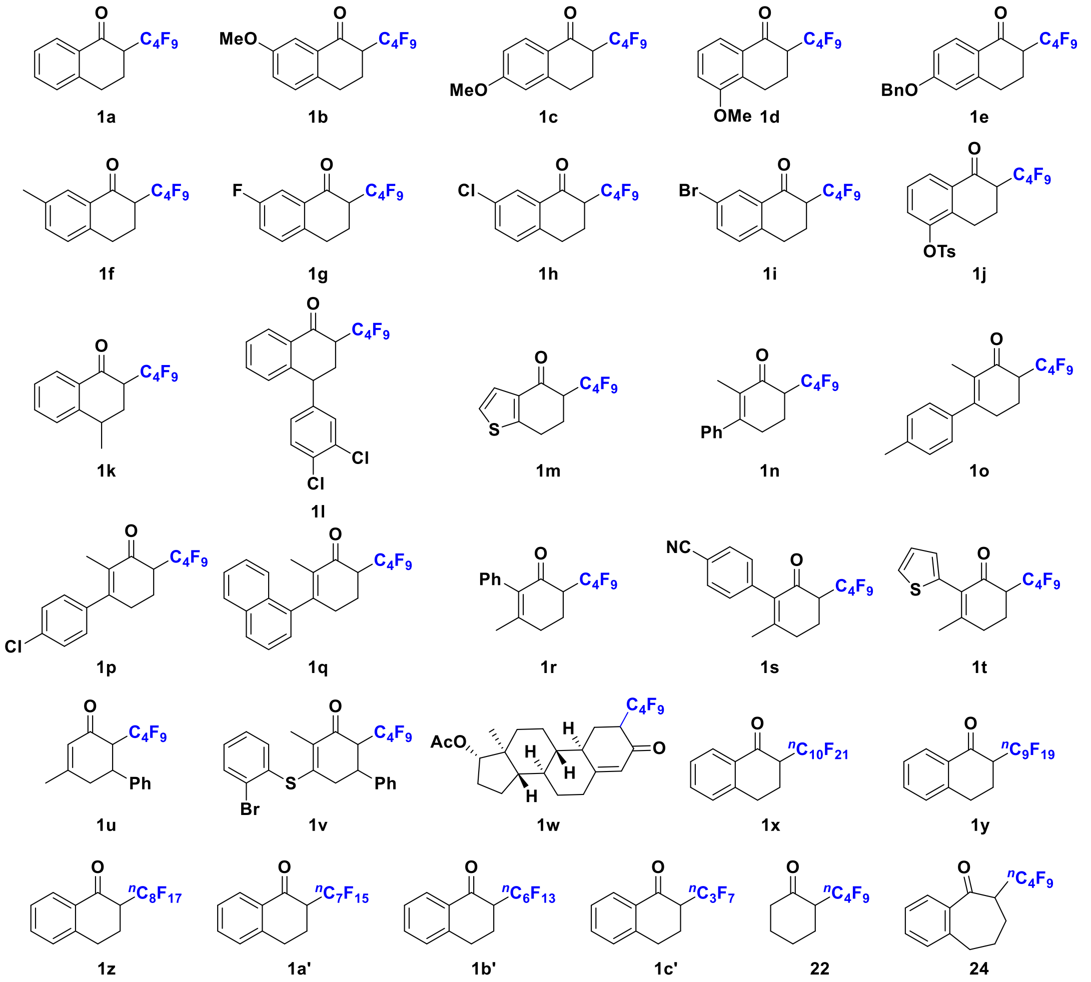

Figure S239. **Various nucleophiles**, related to **Scheme 1-5**.

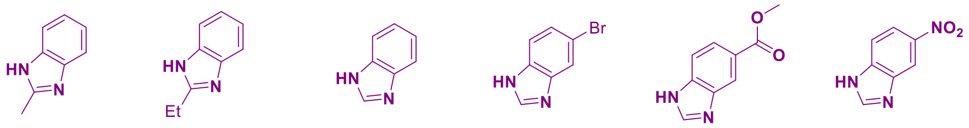

2a 2b 2c 2d 2e 2f

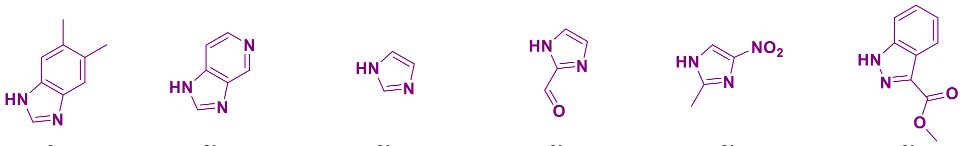

2g 2h 2i 2j 2k 2l

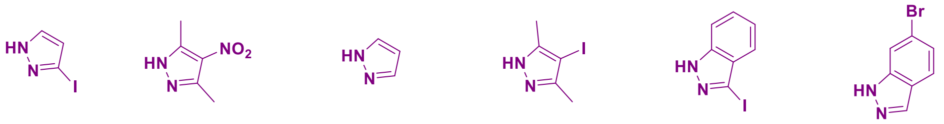

2m 2n 2o 2p 2q 2r

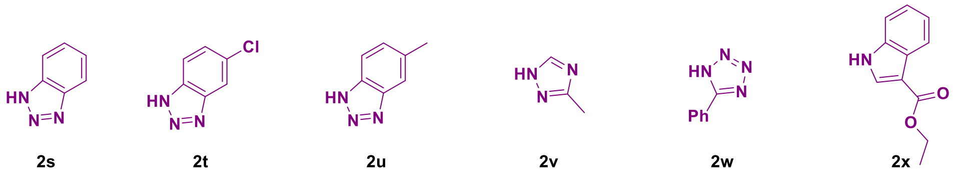

2s 2t 2u 2v 2w 2x

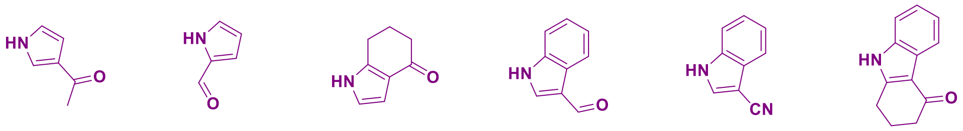

2y 2z 2a' 2b' 2c' 2d'

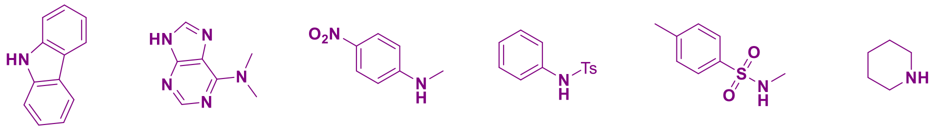

2e' 2f' 2g' 2h' 2i' 2j'

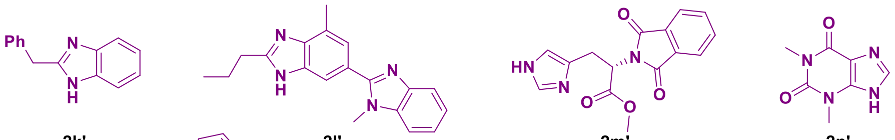

2k' 2l' 2m' 2n'

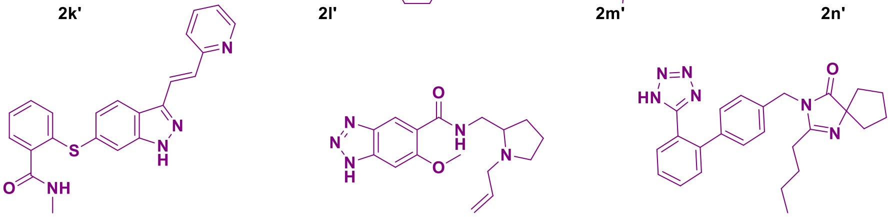

2o' 2p' 2q'

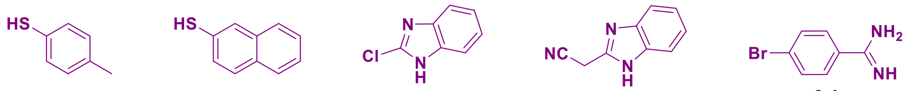

2r' 2s' 2t' 2u' 2v'

Unsuccessful O-nucleophile substrates

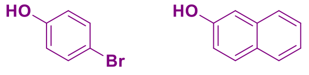

2w' 2x'

Optimization of reaction conditions

Table S1. Initial attempts for the designed reaction[a], related to **Table 1**.

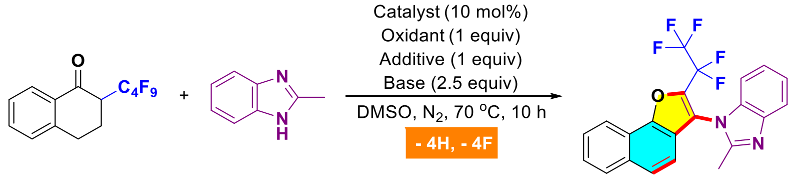

1a **2a** **3a**

Entry	Catatalyst	Additive	Oxidant	Base	Yield of **3a** (%)[b]
1	$CoBr_2$	TBAB	$K_2S_2O_8$	Cs_2CO_3	55 (52)[c]
**2**	**$CoBr_2$**	**TBAB**	**--**	**Cs_2CO_3**	**74 (70)[c]**
3	--	TBAB	$K_2S_2O_8$	Cs_2CO_3	39
4	--	TBAB	--	Cs_2CO_3	39
5	$CoBr_2$	--	--	Cs_2CO_3	58
6	$CoBr_2$	TBAB	$K_2S_2O_8$	--	trace

[a] Reaction conditions: 2-(perfluorobutyl)-3,4-dihydronaphthalen-1(2*H*)-one (**1a**, 0.30 mmol), 2-methyl-1*H*-benzo[*d*]imidazole (**2a**, 0.60 mmol), catalyst (0.03 mmol), oxidant (0.3 mmol), additive (0.3 mmol), and base (0.75 mmol) in DMSO (2.0 mL) at 70 °C under N_2 for 10 h. [b] Yields were determined by NMR analysis with 1,4-dimethoxybenzene as an internal standard. [c] Isolated yield.

Table S2. Optimization of the reaction temperature[a], related to **Table 1**.

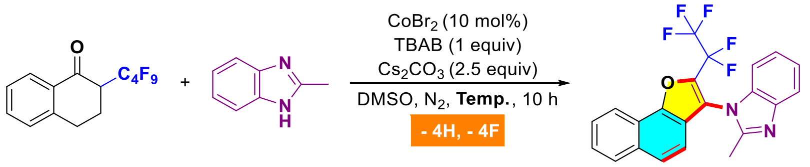

1a **2a** **3a**

Entry	**Temperature (°C)**	Yield of **3a** (%)[b]
**1**	**rt**	**74 (71)[c]**
2	50	55
3	70	74
4	100	46

[a] Reaction conditions: 2-(perfluorobutyl)-3,4-dihydronaphthalen-1(2*H*)-one (**1a**, 0.30 mmol), 2-methyl-1*H*-benzo[*d*]imidazole (**2a**, 0.60 mmol), $CoBr_2$ (0.03 mmol), TBAB (0.3 mmol), and Cs_2CO_3 (0.75 mmol) in DMSO (2.0 mL) under N_2 for 10 h. [b] Yields were determined by NMR analysis with 1,4-dimethoxybenzene as an internal standard. [c] Isolated yield.

Table S3. Optimization of the reaction solvent[a], related to **Table 1**.

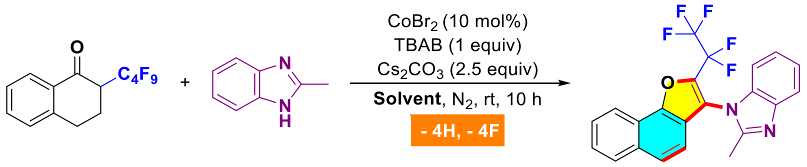

Entry	**Solvent**	Yield of **3a** (%)[b]
**1**	**DMSO**	**74 (71)[c]**
2	MeCN	55
3	$MeNO_2$	0
4	DCM	66
5	DMF	61

[a] Reaction conditions: 2-(perfluorobutyl)-3,4-dihydronaphthalen-1(2*H*)-one (**1a**, 0.30 mmol), 2-methyl-1*H*-benzo[*d*]imidazole (**2a**, 0.60 mmol), $CoBr_2$ (0.03 mmol), TBAB (0.3 mmol), and Cs_2CO_3 (0.75 mmol) in solvent (2.0 mL) at room temperature under N_2 for 10 h. [b] Yields were determined by NMR analysis with 1,4-dimethoxybenzene as an internal standard. [c] Isolated yield.

Table S4. Optimization of the reaction base[a], related to **Table 1**.

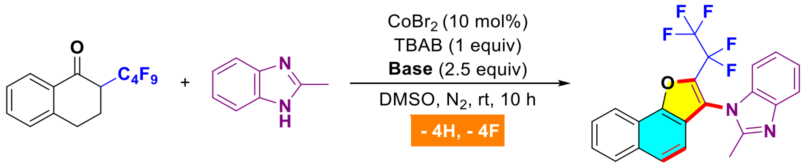

Entry	**Base**	Yield of **3a** (%)[b]
**1**	**Cs_2CO_3**	**74 (71)[c]**
2	K_2CO_3	67
3	Li_2CO_3	0
4	^{t}BuONa	62
5	DABCO	<10
6	LiOH	56

[a] Reaction conditions: 2-(perfluorobutyl)-3,4-dihydronaphthalen-1(2*H*)-one (**1a**, 0.30 mmol), 2-methyl-1*H*-benzo[*d*]imidazole (**2a**, 0.60 mmol), $CoBr_2$ (0.03 mmol), TBAB (0.3 mmol), and base (0.75 mmol) in DMSO (2.0 mL) at room temperature under N_2 for 10 h. [b] Yields were determined by NMR analysis with 1,4-dimethoxybenzene as an internal standard. [c] Isolated yield.

Table S5. Optimization of the reaction catalyst[a], related to **Table 1**.

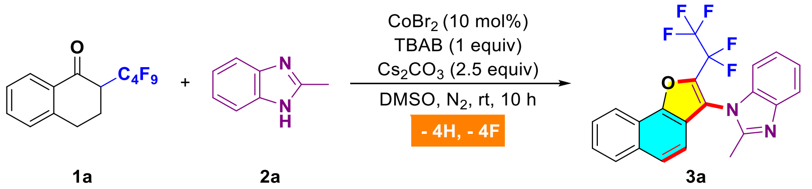

Entry	**Catalyst**	Yield of **3a** (%)[b]
**1**	**$CoBr_2$**	**74 (71)[c]**
2	$Co(OAc)_2$	49
**3**	**$CoCl_2{\cdot}6H_2O$**	**75 (72)[c]**
4	$Co(C_2O_4)_2{\cdot}2H_2O$	0
5	$CuBr_2$	70

[a] Reaction conditions: 2-(perfluorobutyl)-3,4-dihydronaphthalen-1(2*H*)-one (**1a**, 0.30 mmol), 2-methyl-1*H*-benzo[*d*]imidazole (**2a**, 0.60 mmol), catalyst (0.03 mmol), TBAB (0.3 mmol), and Cs_2CO_3 (0.75 mmol) in DMSO (2.0 mL) at room temperature under N_2 for 10 h. [b] Yields were determined by NMR analysis with 1,4-dimethoxybenzene as an internal standard. [c] Isolated yield.

Table S6. Other Lewis acids as catalysts[a], related to **Table 1**.

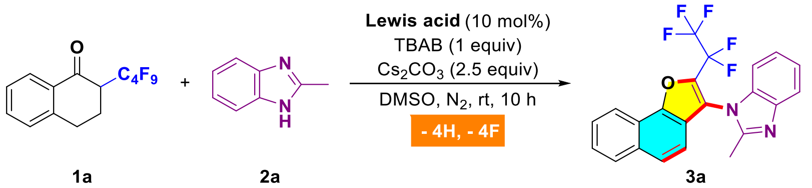

Entry	**Lewis acid**	Yield of **3a** (%)[b]
1	--	39
2	$CoBr_2$	74
3	BF_3 Et_2O	60
4	$AlCl_3$	74
5	$NiBr_2$	50
6	$InBr_3$	72
7	$ZnBr_2$	66
8	$GaBr_3$	75
9	$Fe(OTf)_3$	71
10	$FeCl_2$	63
11	$Fe(acac)_3$	44

[a] Reaction conditions: 2-(perfluorobutyl)-3,4-dihydronaphthalen-1(2*H*)-one (**1a**, 0.30 mmol), 2-methyl-1*H*-benzo[*d*]imidazole (**2a**, 0.60 mmol), Lewis acid (0.03 mmol), TBAB (0.3 mmol), and Cs_2CO_3 (0.75 mmol) in DMSO (2.0 mL) at room temperature under N_2 for 10 h. [b] Yields were determined by NMR analysis with 1,4-dimethoxybenzene as an internal standard.

Preparation of Substrates

General procedures for the synthesis of α-perfluoroalkyl ketones

Figure S240. General procedure A (Pham et al., 2011), related to **Scheme 1-4**.

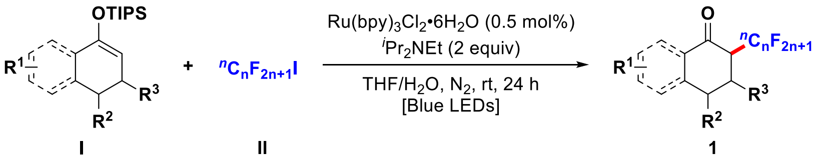

According to MacMillan's reported method, a solution of enolsilane **I** (1.2 mmol), $Ru(bpy)_3Cl_2$•$6H_2O$ (4.5 mg, 0.006 mmol, 0.5 mol%), *N*-ethyl-*N*-isopropylpropan-2-amine (424.0 μL, 2.4 mmol), perfluoroalkyl iodide **II** (12 mmol), H_2O (32.0 μL, 17.8 mmol) in THF (6.0 mL) was stirred under nitrogen atmosphere (by 3 times' vacuum evacuation/N_2 backfill cycles) by irradiation with 8 W Blue LEDs at room temperature for 24 h. Upon completion of the reaction (indicated by TLC), solvent was removed under vacuum and the residue was purified by flash silica gel column chromatography (300-400 mesh) using petroleum ether/ethyl acetate (1:500) as eluent to afford the α-perfluoroalkyl ketone **1**.

Representative examples:

2-(Perfluorobutyl)-3,4-dihydronaphthalen-1(2*H*)-one (1a) (Lee et al., 1988)

^{1}H NMR (400 MHz, $CDCl_3$): δ = 8.08 – 8.01 (m, 1H), 7.57 – 7.49 (m, 1H), 7.35 (t, J = 7.6 Hz, 1H), 7.28 (s, 1H), 3.49 – 3.35 (m, 1H), 3.23 – 3.15 (m, 1H), 3.08 – 2.98 (m, 1H), 2.55 – 2.36 (m, 2H) ppm.

^{19}F NMR (376 MHz, $CDCl_3$): δ = -80.45 – -81.25 (m, 3F), -108.96 – -113.38 (m, 2F), -118.78 – -121.94 (m, 2F), -125.80 – -126.50 (m, 2F) ppm.

^{13}C NMR (100 MHz, $CDCl_3$): δ = 190.3 (m), 142.9, 134.2, 132.3 (m), 128.7, 127.9, 127.0, 49.3 (t, J = 20.3 Hz), 27.1, 22.9 (m) ppm; carbons corresponding to the C_4F_9 group cannot be identified due to C-F coupling.

2-Methyl-4-(perfluorobutyl)-5,6-dihydro-[1,1'-biphenyl]-3(4*H*)-one (1n)

^{1}H NMR (400 MHz, $CDCl_3$): δ = 7.43 – 7.38 (m, 2H), 7.37 – 7.32 (m, 1H), 7.23 – 7.19 (m, 2H), 3.39 – 3.24 (m, 1H), 2.88 – 2.76 (m, 1H), 2.73 – 2.64 (m, 1H), 2.48 – 2.34 (m, 2H), 1.76 (t, J = 1.7 Hz, 3H) ppm.

^{19}F NMR (376 MHz, $CDCl_3$): δ = -80.67 – -81.08 (m, 3F), -108.12 – -114.26 (m, 2F), -119.45 – -122.63 (m, 2F), -125.74 – -126.38 (m, 2F) ppm.

^{13}C NMR (100 MHz, $CDCl_3$): δ = 191.6, 156.5, 140.3, 132.2, 128.5, 128.3, 126.9, 47.6 (t, *J* = 20.3 Hz), 30.7, 22.0, 13.1 ppm; carbons corresponding to the C_4F_9 group cannot be identified due to C-F coupling.

2,4'-Dimethyl-4-(perfluorobutyl)-5,6-dihydro-[1,1'-biphenyl]-3(4*H*)-one (1o)

^{1}H NMR (400 MHz, $CDCl_3$): δ = 7.25 – 7.20 (m, 2H), 7.13 – 7.08 (m, 2H), 3.36 – 3.22 (m, 1H), 2.87 – 2.75 (m, 1H), 2.72 – 2.60 (m, 1H), 2.38 (s, 3H) ppm.

^{19}F NMR (376 MHz, $CDCl_3$): δ = -80.77 (t, *J* = 9.8 Hz, 3F), -109.70 – -114.25 (m, 2F), -119.29 – -122.06 (m, 2F), -125.98 (t, *J* = 15.4 Hz, 2F) ppm.

^{13}C NMR (100 MHz, $CDCl_3$): δ = 191.7 (t, *J* = 1.4 Hz), 156.6, 138.4, 137.3, 132.1, 129.1, 127.0, 47.6 (t, *J* = 20.2 Hz), 30.7, 22.0 (m), 21.2, 13.3 ppm; carbons corresponding to the C_4F_9 group cannot be identified due to C-F coupling.

4'-Chloro-2-methyl-4-(perfluorobutyl)-5,6-dihydro-[1,1'-biphenyl]-3(4*H*)-one (1p)

^{1}H NMR (400 MHz, $CDCl_3$): δ = 7.34 (d, *J* = 8.4 Hz, 2H), 7.14 (d, *J* = 8.4 Hz, 2H), 3.35 – 3.20 (m, 1H), 2.83 – 2.71 (m, 1H), 2.70 – 2.58 (m, 1H), 2.46 – 2.29 (m, 2H), 1.79 (s, 3H) ppm.

^{19}F NMR (376 MHz, $CDCl_3$): δ = -80.80 – -80.92 (m, 3F), -110.28 – -114.09 (m, 2F), -119.53 – -121.96 (m, 2F), -125.96 – -126.14 (m, 2F) ppm.

^{13}C NMR (100 MHz, $CDCl_3$): δ = 191.2, 154.9, 138.6, 134.2, 132.4, 128.6, 128.4, 47.6 (t, *J* = 20.6 Hz), 30.4, 21.8, 12.9 ppm; carbons corresponding to the C_4F_9 group cannot be identified due to C-F coupling.

2-Methyl-3-(naphthalen-1-yl)-6-(perfluorobutyl)cyclohex-2-en-1-one (1q)

^{1}H NMR (400 MHz, $CDCl_3$): δ = 7.93 – 7.86 (m, 1H), 7.83 (d, *J* = 8.3 Hz, 1H), 7.69 – 7.56 (m, 1H), 7.54 – 7.46 (m, 3H), 7.24 – 7.18 (m, 1H), 3.55 – 3.31 (m, 1H), 2.96 – 2.78 (m, 1H), 2.78 – 2.58 (m, 1H), 2.50 (d, *J* = 5.4 Hz, 2H), 1.56 (s, 3H) ppm.

^{19}F NMR (376 MHz, $CDCl_3$): δ = -80.80 (s, 3F), -109.50 – -113.97 (m, 2F), -119.36 – -122.12 (m, 2F), -125.92 (t, *J* = 12.4 Hz, 2F) ppm.

^{13}C NMR (100 MHz, $CDCl_3$): δ = 191.3 (m), 156.2 (d, J = 18.6 Hz), 138.1 (d, J = 5.1 Hz), 134.2 (d, J = 9.4 Hz), 133.6, 129.1 (d, J = 9.3 Hz), 128.7 (d, J = 9.2 Hz), 128.2 (d, J = 7.5 Hz), 126.8 (d, J = 16.2 Hz), 126.2 (d, J = 5.1 Hz), 125.4 (d, J = 11.6 Hz), 124.3 (d, J = 4.8 Hz), 123.6 (d, J = 3.6 Hz), 47.7 (q, J = 20.0 Hz), 31.1 (d, J = 23.2 Hz), 22.3 (m), 12.9 ppm; carbons corresponding to the C_4F_9 group cannot be identified due to C-F coupling.

6-Methyl-3-(perfluorobutyl)-4,5-dihydro-[1,1'-biphenyl]-2(3*H*)-one (1r)

^{1}H NMR (400 MHz, $CDCl_3$): δ = 7.40 – 7.32 (m, 2H), 7.31 – 7.25 (m, 1H), 7.04 (d, J = 7.1 Hz, 2H), 3.40 – 3.19 (m, 1H), 2.73 – 2.59 (m, 1H), 2.56 – 2.43 (m, 1H), 2.42 – 2.30 (m, 2H), 1.81 (s, 3H) ppm.

^{19}F NMR (376 MHz, $CDCl_3$): δ = -80.91 (s, 3F), -110.17 – -113.94 (m, 2F), -119.49 – -122.08 (m, 2F), -126.06 (t, J = 14.9 Hz, 2F) ppm.

^{13}C NMR (100 MHz, $CDCl_3$): δ = 189.8 (m), 157.5, 137.9 (d, J = 1.1 Hz), 135.1, 129.8, 128.0, 127.3, 47.7 (t, J = 21.0 Hz), 30.1, 22.4, 21.4 (m) ppm; carbons corresponding to the C_4F_9 group cannot be identified due to C-F coupling.

6'-Methyl-2'-oxo-3'-(perfluorobutyl)-2',3',4',5'-tetrahydro-[1,1'-biphenyl]-4-carbonitrile (1s)

^{1}H NMR (400 MHz, $CDCl_3$): δ = 7.66 (d, J = 7.6 Hz, 2H), 7.18 (d, J = 7.5 Hz, 2H), 3.43 – 3.26 (m, 1H), 2.82 – 2.67 (m, 1H), 2.64 – 2.52 (m, 1H), 2.46 – 2.37 (m, 2H), 1.85 (s, 3H) ppm.

^{19}F NMR (376 MHz, $CDCl_3$): δ = -80.83 (t, J = 9.7 Hz, 3F), -110.32 – -113.64 (m, 2F), -119.39 – -122.20 (m, 2F), -126.01 (t, J = 15.1 Hz, 2F) ppm.

^{13}C NMR (100 MHz, $CDCl_3$): δ = 189.2 (t, J = 2.1 Hz), 158.9, 140.1, 136.5 (d, J = 2.0 Hz), 131.9, 130.8, 118.7, 111.3, 47.5 (t, J = 21.4 Hz), 30.2, 22.4, 21.3 (m) ppm; carbons corresponding to the C_4F_9 group cannot be identified due to C-F coupling.

3-Methyl-6-(perfluorobutyl)-2-(thiophen-2-yl)cyclohex-2-en-1-one (1t)

^{1}H NMR (400 MHz, $CDCl_3$): δ = 7.37 (d, J = 4.9 Hz, 1H), 7.04 (t, J = 4.1 Hz, 1H), 6.84 (d, J = 2.5 Hz, 1H), 3.41 – 3.25 (m, 1H), 2.80 – 2.67 (m, 1H), 2.63 – 2.50 (m, 1H), 2.43 – 2.33 (m, 2H), 2.01 (s, 3H) ppm.

^{19}F NMR (376 MHz, $CDCl_3$): δ = -80.09 (t, J = 9.8 Hz, 3F), -109.64 – -113.10 (m, 2F), -118.74

– -121.25 (m, 2F), -125.28 (t, *J* = 15.1 Hz, 2F) ppm.

^{13}C NMR (100 MHz, $CDCl_3$): *δ* = 189.1 (m), 160.1, 134.6, 131.1, 128.4, 126.5, 126.4, 47.8 (t, *J* = 21.3 Hz), 30.8 (t, *J* = 1.5 Hz), 23.0, 21.3 (m) ppm; carbons corresponding to the C_4F_9 group cannot be identified due to C-F coupling.

5-Methyl-2-(perfluorobutyl)-1,6-dihydro-[1,1'-biphenyl]-3(2*H*)-one (1u)

^{1}H NMR (400 MHz, $CDCl_3$): *δ* = 7.31 – 7.18 (m, 3H), 7.11 (d, *J* = 7.3 Hz, 2H), 6.12 (s, 1H), 4.00 – 3.89 (m, 1H), 3.47 – 3.26 (m, 1H), 3.03 (dd, *J* = 19.7, 5.2 Hz, 1H), 2.55 (d, *J* = 19.8 Hz, 1H), 2.03 (s, 3H) ppm.

^{19}F NMR (376 MHz, $CDCl_3$): *δ* = -80.81 – -81.32 (m, 3F), -110.57 – -112.97 (m, 2F), -119.91 – -122.06 (m, 2F), -124.76 – -127.17 (m, 2F) ppm.

^{13}C NMR (100 MHz, $CDCl_3$): *δ* = 189.6 (m), 161.6, 142.0, 128.9, 128.9, 127.3 (t, *J* = 1.9 Hz), 126.8, 52.6 (t, *J* = 20.1 Hz), 37.4 (t, *J* = 1.7 Hz), 34.2, 24.1 (m) ppm; carbons corresponding to the C_4F_9 group cannot be identified due to C-F coupling.

3-((2-Bromophenyl)thio)-5-methyl-6-(perfluorobutyl)cyclohex-2-en-1-one (1v)

^{1}H NMR (400 MHz, $CDCl_3$): *δ* = 7.74 (d, *J* = 7.6 Hz, 1H), 7.66 – 7.60 (m, 1H), 7.42 – 7.30 (m, 2H), 5.50 (d, *J* = 2.0 Hz, 1H), 3.12 – 2.80 (m, 3H), 2.27 (d, *J* = 18.4 Hz, 1H), 1.20 (d, *J* = 7.1 Hz, 3H) ppm.

^{19}F NMR (376 MHz, $CDCl_3$): *δ* = -80.74 – -80.90 (m, 3F), -109.53 – -114.50 (m, 2F), -120.05 – -122.55 (m, 2F), -125.68 – -126.00 (m, 2F) ppm.

^{13}C NMR (100 MHz, $CDCl_3$): *δ* = 186.8 (m), 163.1, 137.7, 134.3, 132.1, 130.5, 128.8, 128.7, 120.1 (d, *J* = 1.7 Hz), 53.0 (t, *J* = 20.1 Hz), 33.6 (t, *J* = 2.6 Hz), 27.7 (m), 19.9 (d, *J* = 0.9 Hz) ppm; carbons corresponding to the C_4F_9 group cannot be identified due to C-F coupling.

(8*R*,9*S*,10*R*,13*S*,14*S*,17*S*)-13-Methyl-3-oxo-2-(perfluorobutyl)-2,3,6,7,8,9,10,11,12,13,14,15,16,17-tetradecahydro-1*H*-cyclopenta[*a*]phenanthren-17-yl acetate (1w)

^{1}H NMR (400 MHz, $CDCl_3$): *δ* = 5.96 (s, 1H), 4.67 – 4.57 (m, 1H), 3.18 (t, *J* = 17.9 Hz, 1H), 2.68 – 2.47 (m, 2H), 2.35 – 2.25 (m, 2H), 2.24 – 2.13 (m, 1H), 2.05 (s, 3H), 1.91 – 1.75 (m, 4H), 1.72

– 1.60 (m, 2H), 1.58 – 1.48 (m, 1H), 1.41 – 1.24 (m, 4H), 1.14 – 1.05 (m, 2H), 0.86 (s, 3H) ppm.

^{19}F NMR (376 MHz, $CDCl_3$): *δ* = -80.82 (t, *J* = 9.5 Hz, 3F), -110.30 – -113.92 (m, 2F), -120.11 – -122.69 (m, 2F), -125.83 (t, *J* = 15.1 Hz, 2F) ppm.

^{13}C NMR (100 MHz, $CDCl_3$): *δ* = 190.6 (m), 171.2, 167.9, 124.3 (d, *J* = 1.5 Hz), 82.4, 50.2, 49.4, 46.5 (t, *J* = 20.4 Hz), 42.6, 40.0, 39.9 (t, *J* = 1.4 Hz), 36.4, 35.5, 30.7, 27.4, 26.4 (m), 25.7, 23.3, 21.1, 12.0 ppm; carbons corresponding to the C_4F_9 group cannot be identified due to C-F coupling.

2-(Perfluorobutyl)-3,4-dihydronaphthalen-1(2*H*)-one (**1a**), 6-(benzyloxy)-2-(perfluorobutyl)-3,4-dihydronaphthalen-1(2*H*)-one (**1e**), 5-oxo-6-(perfluorobutyl)-5,6,7,8-tetrahydronaphthalen-1-yl 4-methylbenzenesulfonate (**1j**), 5-(perfluorobutyl)-6,7-dihydrobenzo[*b*]thiophen-4(5*H*)-one (**1m**), 2-methyl-4-(perfluorobutyl)-5,6-dihydro-[1,1'-biphenyl]-3(4*H*)-one (**1n**), 2,4'-dimethyl-4-(perfluorobutyl)-5,6-dihydro-[1,1'-biphenyl]-3(4*H*)-one (**1o**), 4'-chloro-2-methyl-4-(perfluorobutyl)-5,6-dihydro-[1,1'-biphenyl]-3(4*H*)-one (**1p**), 2-methyl-3-(naphthalen-1-yl)-6-(perfluorobutyl)cyclohex-2-en-1-one (**1q**), 6-methyl-3-(perfluorobutyl)-4,5-dihydro-[1,1'-biphenyl]-2(3*H*)-one (**1r**), 6'-methyl-2'-oxo-3'-(perfluorobutyl)-2',3',4',5'-tetrahydro-[1,1'-biphenyl]-4-carbonitrile (**1s**), 3-methyl-6-(perfluorobutyl)-2-(thiophen-2-yl)cyclohex-2-en-1-one (**1t**), 5-methyl-2-(perfluorobutyl)-1,6-dihydro-[1,1'-biphenyl]-3(2*H*)-one (**1u**), 3-((2-bromophenyl)thio)-5-methyl-6-(perfluorobutyl)cyclohex-2-en-1-one (**1v**), 2-(perfluorodecyl)-3,4-dihydronaphthalen-1(2*H*)-one (**1x**), 2-(perfluorononyl)-3,4-dihydronaphthalen-1(2*H*)-one (**1y**), 2-(perfluorooctyl)-3,4-dihydronaphthalen-1(2*H*)-one (**1z**), 2-(perfluoroheptyl)-3,4-dihydronaphthalen-1(2*H*)-one (**1a'**), 2-(perfluorohexyl)-3,4-dihydronaphthalen-1(2*H*)-one (**1b'**), and 2-(perfluoropropyl)-3,4-dihydronaphthalen-1(2*H*)-one (**1c'**) were synthesized according to general procedure A.

Figure S241. General procedure B (Su et al., 2017; Xie et al., 2018), related to **Scheme 1-4**.

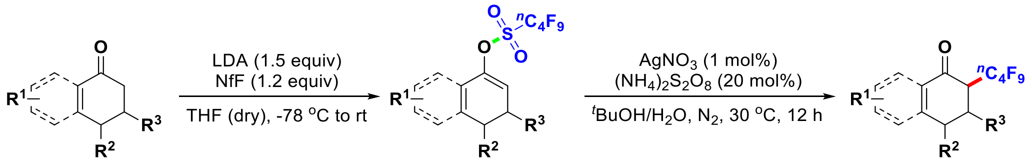

Step 1: The solution of ketone **III** (5 mmol) in dry THF (25 mL) was cooled to -78 °C and then lithium diisopropylamide (LDA, 3.75 mL, 7.5 mmol, 2.0 mol/L in THF/hexane) was dropwise added to the reaction mixture. Nonafluorobutanesulfonyl fluoride (1.1 mL, 6 mmol) was added slowly by a syringe over 10 min. The reaction mixture was warmed to room temperature and stirred overnight. The reaction was then quenched by saturated NH_4Cl solution (20 mL) and diluted with EtOAc (20 mL). The organic layer was washed with saturated brine twice, dried over $MgSO_4$, filtered, and concentrated under reduced pressure. The crude product was purified by flash column chromatography (300-400 mesh) on Et_3N-treated silica gel eluting with petroleum ether to afford enol nonaflate **IV**.

Step 2: A solution of enol nonaflate **IV** (0.8 mmol), $(NH_4)_2S_2O_8$ (0.16 mmol, 37 mg), and $AgNO_3$ (0.008 mmol, 1.4 mg) in ^{t}BuOH (2.0 mL) and H_2O (2.0 mL) was stirred vigorously under nitrogen atmosphere (by 3 times' vacuum evacuation/N_2 backfill cycles) at 30 °C for 12 h. Upon completion of the reaction (indicated by TLC), the reaction mixture was diluted with dichloromethane. The organic layer was separated and the aqueous layer was washed with dichloromethane (3×10 mL). The combined organic layers were dried over Na_2SO_4. The solvent was removed under vacuum and the residue was purified by flash silica gel column chromatography (300-400 mesh) using petroleum ether/ethyl acetate as eluent to afford α-perfluoroalkyl ketone **1**.

Representative examples:

6-Methoxy-2-(perfluorobutyl)-3,4-dihydronaphthalen-1(2*H*)-one (1b)

^{1}H NMR (400 MHz, $CDCl_3$): δ = 8.02 (d, J = 8.8 Hz, 1H), 6.86 (dd, J = 8.8, 2.5 Hz, 1H), 6.70 (d, J = 2.4 Hz, 1H), 3.87 (s, 3H), 3.44 – 3.30 (m, 1H), 3.20 – 3.10 (m, 1H), 3.00 – 2.91 (m, 1H), 2.50 – 2.34 (m, 2H) ppm.

^{19}F NMR (376 MHz, $CDCl_3$): δ = -80.45 – -81.24 (m, 3F), -108.98 – -113.48 (m, 2F), -118.81 –

-121.88 (m, 2F), -125.99 – -126.55 (m, 2F) ppm.

^{13}C NMR (100 MHz, $CDCl_3$): δ = 188.8 (m), 164.1, 145.5, 130.3, 125.8, 113.7, 112.3, 55.4, 48.9 (t, J = 20.4 Hz), 27.3, 23.0 ppm; carbons corresponding to the C_4F_9 group cannot be identified due to C-F coupling.

7-Methoxy-2-(perfluorobutyl)-3,4-dihydronaphthalen-1(2*H*)-one (**1b**), 6-methoxy-2-(perfluorobutyl)-3,4-dihydronaphthalen-1(2*H*)-one (**1c**), 5-methoxy-2-(perfluorobutyl)-3,4-dihydronaphthalen-1(2*H*)-one (**1d**), 7-methyl-2-(perfluorobutyl)-3,4-dihydronaphthalen-1(2*H*)-one (**1f**), 7-fluoro-2-(perfluorobutyl)-3,4-dihydronaphthalen-1(2*H*)-one (**1g**), 7-chloro-2-(perfluorobutyl)-3,4-dihydronaphthalen-1(2*H*)-one (**1h**), 7-bromo-2-(perfluorobutyl)-3,4-dihydronaphthalen-1(2*H*)-one (**1i**), 4-methyl-2-(perfluorobutyl)-3,4-dihydronaphthalen-1(2*H*)-one (**1k**), 4-(3,4-dichlorophenyl)-2-(perfluorobutyl)-3,4-dihydronaphthalen-1(2*H*)-one (**1l**), 2-(perfluorobutyl)cyclohexan-1-one (**22**), and 6-(perfluorobutyl)-6,7,8,9-tetrahydro-5*H*-benzo[7]annulen-5-one (**24**) were synthesized according to general procedure B.

Mechanistic studies

Figure S242. Control experiment of 2-(perfluorobutyl)cyclohexan-1-one (22) with 1*H*-benzo[*d*][1,2,3]triazole (2s), related to **Scheme 4**.

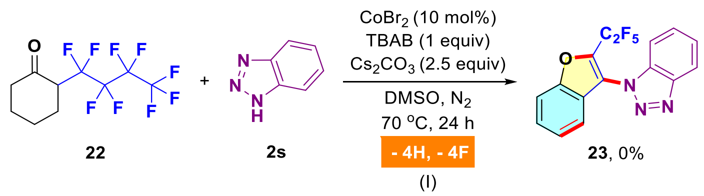

A solution of 2-(perfluorobutyl)cyclohexan-1-one (**22**, 95 mg, 0.3 mmol), 1*H*-benzo[*d*][1,2,3]triazole (**2s**, 71 mg, 0.6 mmol), $CoBr_2$ (66 mg, 0.03 mmol), tetrabutylammonium bromide (97 mg, 0.3 mmol, TBAB), and Cs_2CO_3 (244 mg, 0.75 mmol) in DMSO (2.0 mL) was stirred under nitrogen atmosphere at 70 °C for 24 h. No target product **23** was obtained. **This result suggested that the phenyl moiety or unsaturated C=C bond in the α-perfluoroalkyl ketone was essential for the established reaction.**

Figure S243. Control experiment of 6-(perfluorobutyl)-6,7,8,9-tetrahydro-5*H*-

benzo[7]annulen-5-one (24) with 1*H*-benzo[*d*][1,2,3]triazole (2s), related to **Scheme 4**.

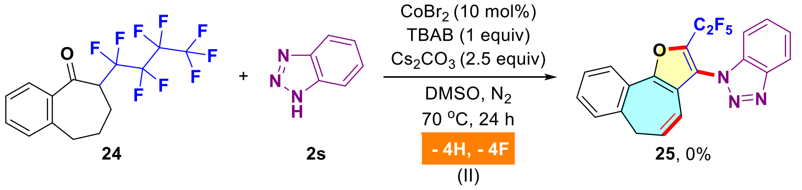

A solution of 6-(perfluorobutyl)-6,7,8,9-tetrahydro-5*H*-benzo[7]annulen-5-one (**24**, 114 mg, 0.3 mmol), 1*H*-benzo[*d*][1,2,3]triazole (**2s**, 71 mg, 0.6 mmol), $CoBr_2$ (66 mg, 0.03 mmol), tetrabutylammonium bromide (97 mg, 0.3 mmol, TBAB), and Cs_2CO_3 (244 mg, 0.75 mmol) in DMSO (2.0 mL) was stirred under nitrogen atmosphere at 70 °C for 24 h. No target product **25** was obtained. **This result suggested that the aromatization was the important driving force for the C-F cleavage reaction.**

Figure S244. Control experiment of 2-(perfluorobutyl)-3,4-dihydronaphthalen-1(2*H*)-one (1a) with 1*H*-benzo[*d*][1,2,3]triazole (2j'), related to **Scheme 4**.

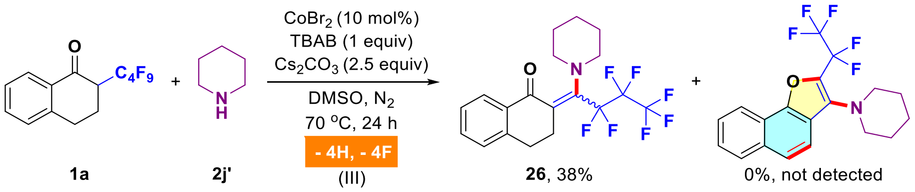

A solution of 2-(perfluorobutyl)-3,4-dihydronaphthalen-1(2*H*)-one (**1a**, 109 mg, 0.3 mmol), piperidine (**2j'**, 64 mg, 0.75 mmol), $CoBr_2$ (66 mg, 0.03 mmol), tetrabutylammonium bromide (97 mg, 0.3 mmol, TBAB), and Cs_2CO_3 (244 mg, 0.75 mmol) in DMSO (2.0 mL) was stirred under nitrogen atmosphere at 70 °C for 24 h. The reaction was then quenched by saturated NH_4Cl solution (20 mL) and diluted with EtOAc (20 mL). The organic layer was washed with saturated brine twice, dried over $MgSO_4$, filtered, and concentrated under reduced pressure. The crude product was purified by flash column chromatography (300-400 mesh) using petroleum ether/ethyl acetate (500/1~200/1) as eluent to afford the pure product **26** (46 mg, 38%). No target product was obtained. **This result suggested that the nucleophile was also essential for the aromatization/cyclization and successive dehydrogenation/defluorination.**

Figure S245. Compound 26, related to **Scheme 4**.

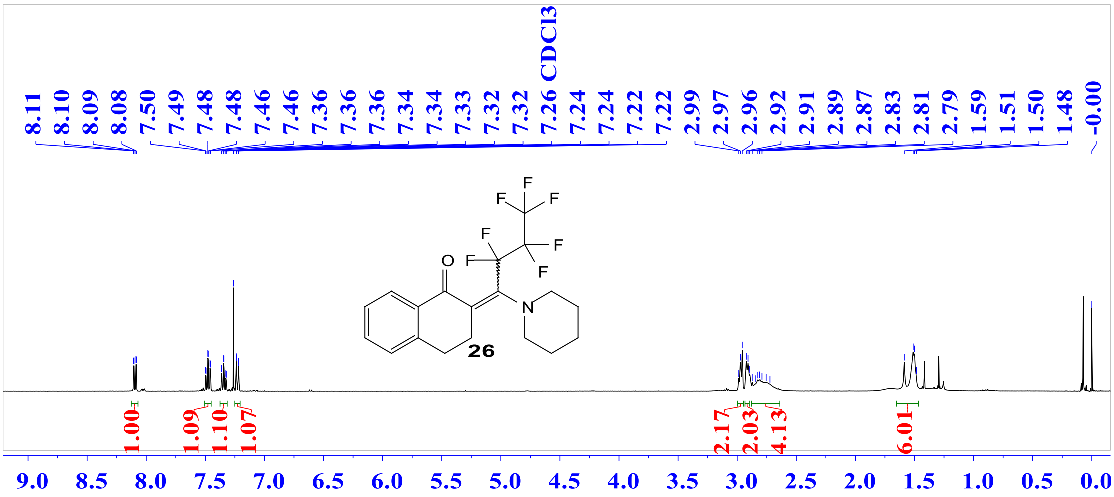

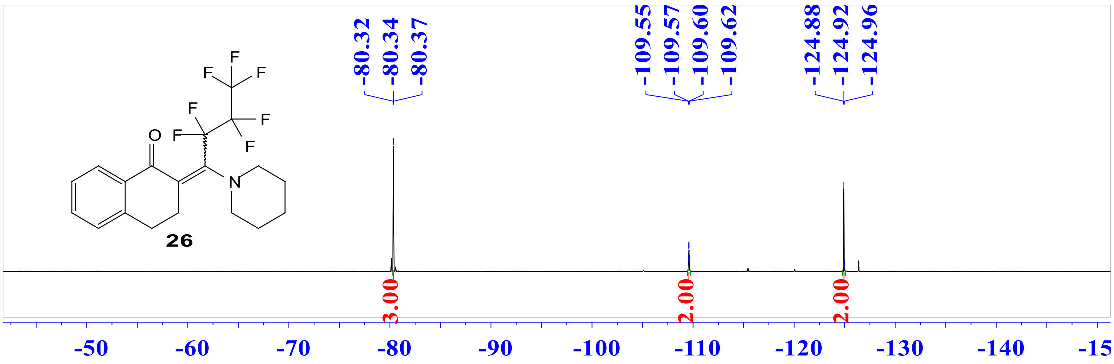

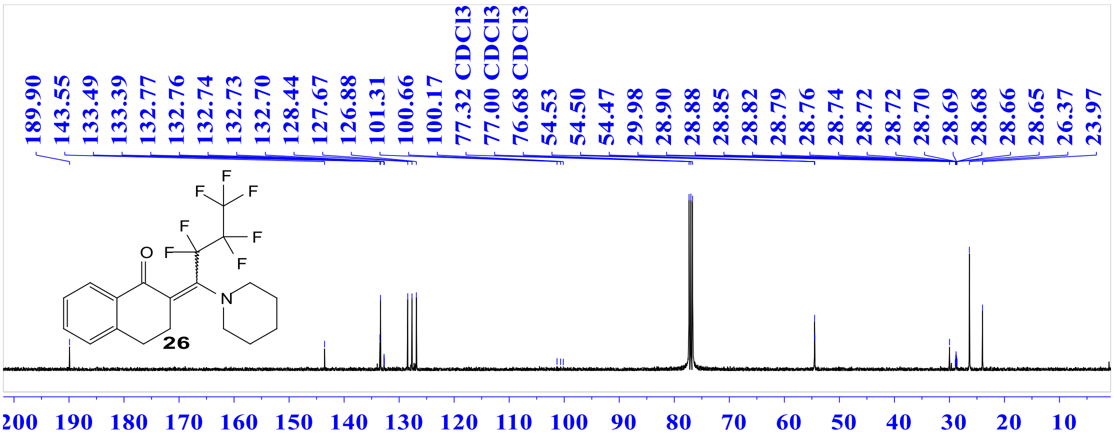

Figure S246. Detection of the by-product 2-(1-bromo-2,2,3,3,4,4,4-heptafluorobutylidene)-3,4-dihydronaphthalen-1(2*H*)-one (27), related to **Scheme 4**.

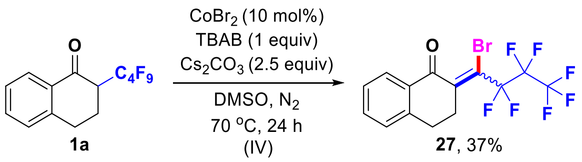

A solution of 2-(perfluorobutyl)-3,4-dihydronaphthalen-1(2*H*)-one (**1a**, 109 mg, 0.3 mmol), $CoBr_2$ (66 mg, 0.03 mmol), tetrabutylammonium bromide (97 mg, 0.3 mmol, TBAB), and

Cs_2CO_3 (244 mg, 0.75 mmol) in DMSO (2.0 mL) was stirred under nitrogen atmosphere at 70 °C for 24 h. The reaction was then quenched by saturated NH_4Cl solution (20 mL) and diluted with EtOAc (20 mL). The organic layer was washed with saturated brine twice, dried over $MgSO_4$, filtered, and concentrated under reduced pressure. The crude product was purified by flash column chromatography (300-400 mesh) using petroleum ether/ethyl acetate (500/1~200/1) as eluent to afford the pure product **27** (45 mg, 37%). **This result suggested that TBAB might participate in the reaction process.**

Figure S247. Compound 27, related to **Scheme 4**.

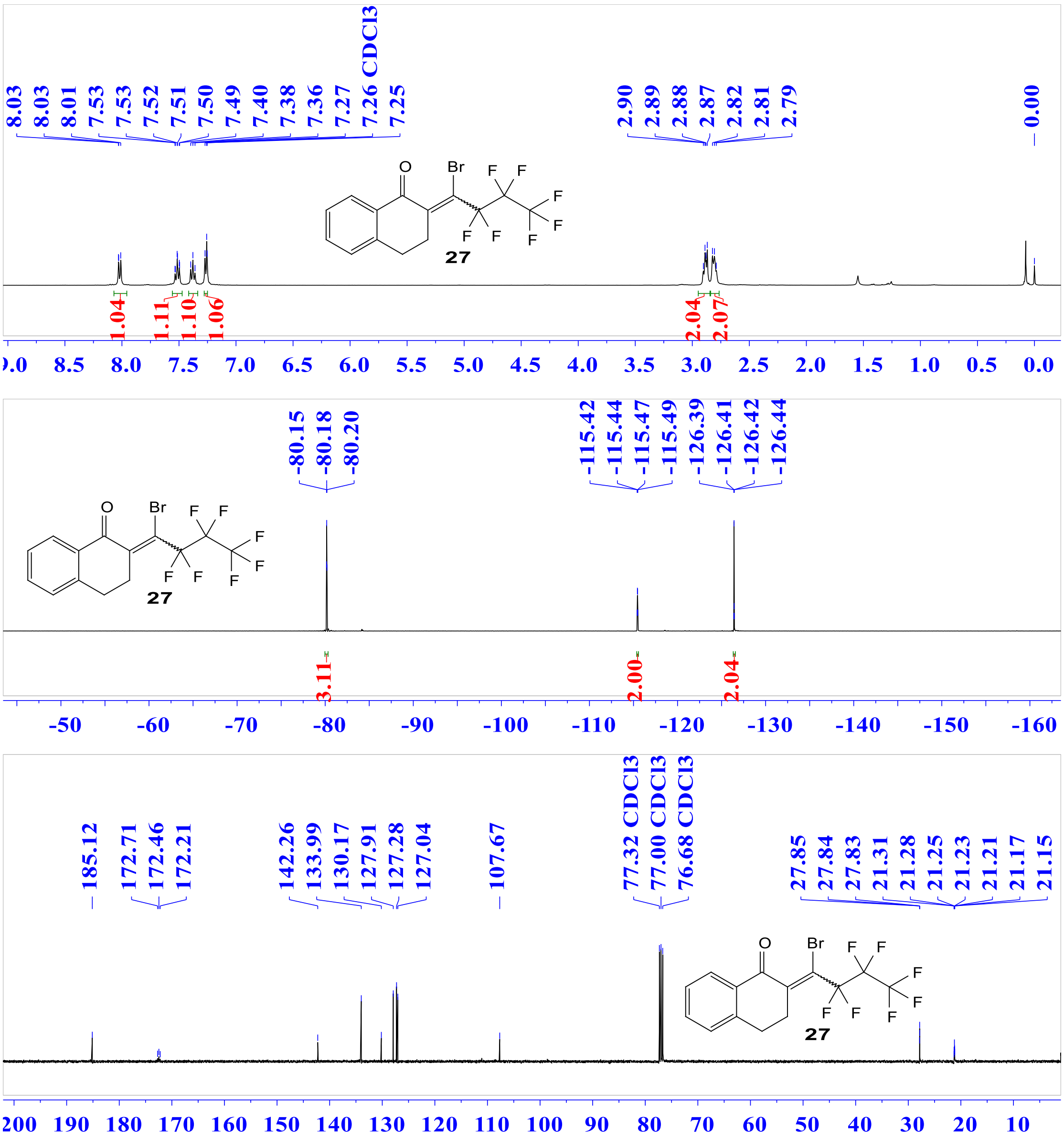

Figure S248. Detection of the intermediate 2-(perfluorobutylidene)-3,4-dihydronaphthalen-1(2*H*)-one (28) and byproduct 2-(trifluoromethyl)-5,6-dihydro-4*H*-benzo[*h*]chromen-4-one (29), related to **Scheme 4**.

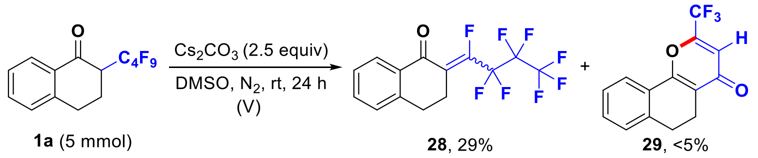

A solution of 2-(perfluorobutyl)-3,4-dihydronaphthalen-1(2*H*)-one (**1a**, 1.82 g, 5 mmol), Cs_2CO_3 (4.1 g, 12.5 mmol) in DMSO (20.0 mL) was stirred under nitrogen atmosphere at room temperature for 24 h. The reaction was then quenched by saturated NH_4Cl solution (20 mL) and diluted with EtOAc (20 mL). The organic layer was washed with saturated brine twice, dried over $MgSO_4$, filtered, and concentrated under reduced pressure. The crude product was purified by flash column chromatography (300-400 mesh) using petroleum ether/ethyl acetate (500/1~200/1) as eluent to afford the pure product **28** (499 mg, 29%) and byproduct **29** (<5%). **This result suggested that compound 28 was the possible reaction intermediate.**

Figure S249. Compound 28, related to **Scheme 4**.

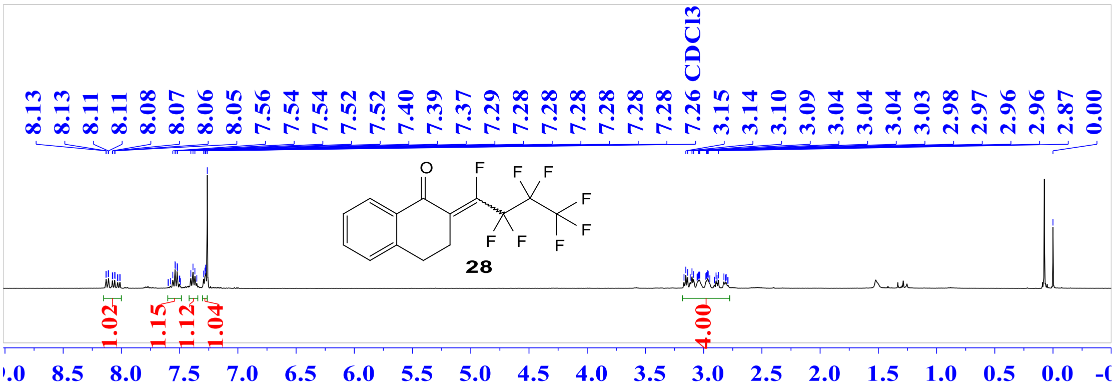

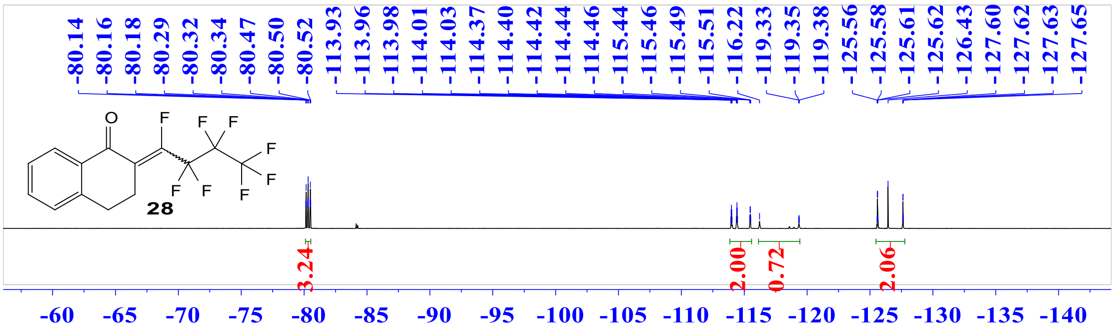

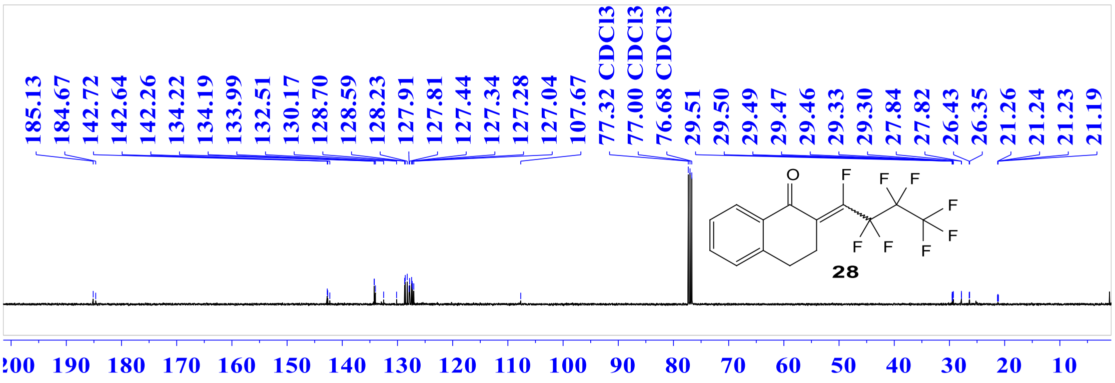

Figure S250. Compound 29, related to **Scheme 4**.

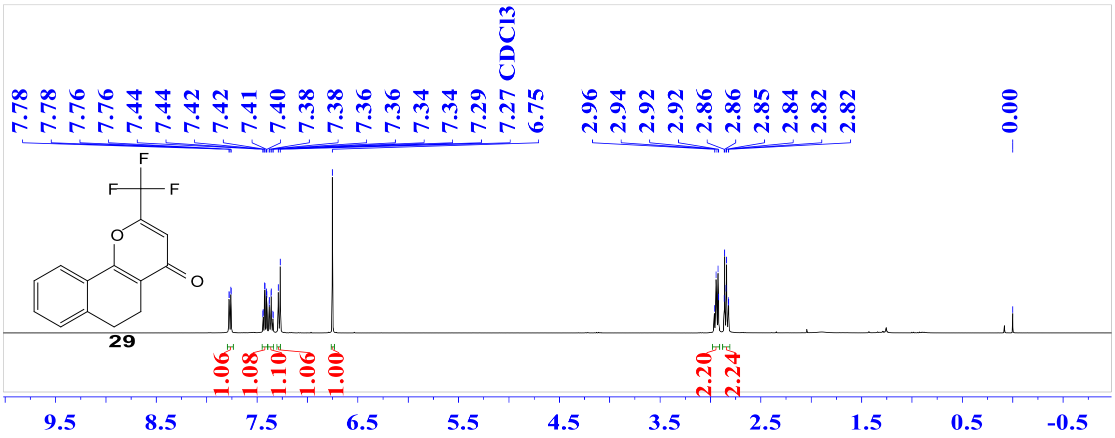

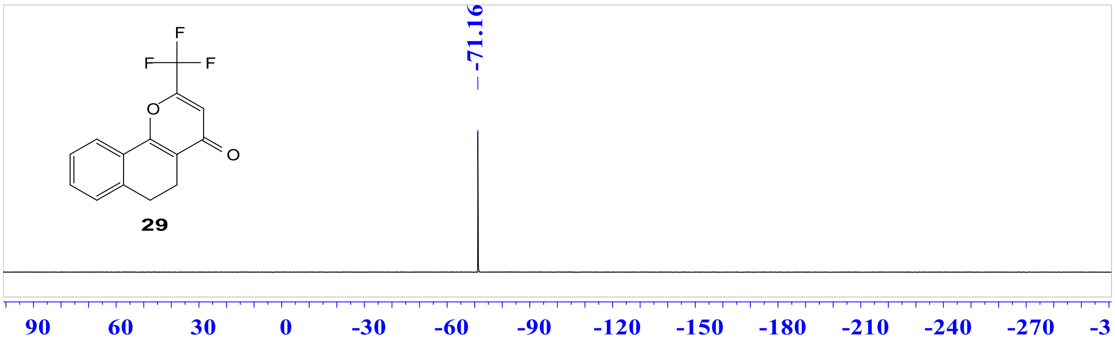

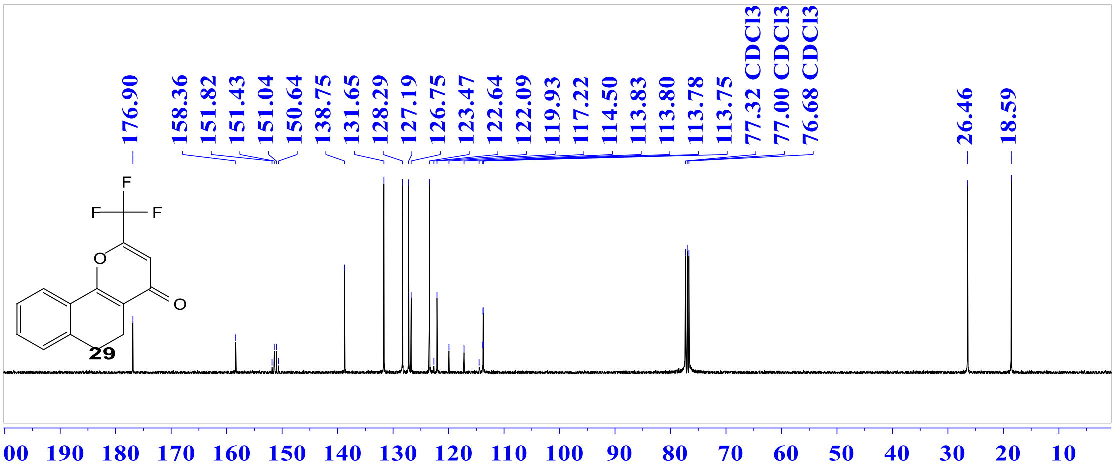

Figure S251. The reaction of 2-(perfluorobutylidene)-3,4-dihydronaphthalen-1(2*H*)-one (28) with 2-methyl-1*H*-benzo[*d*]imidazole (2a), related to **Scheme 4**.

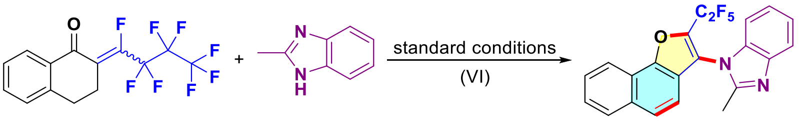

28 **2a** **3a**, 86%

A solution of 2-(perfluorobutyl)-3,4-dihydronaphthalen-1(2*H*)-one (**28**, 103 mg, 0.3 mmol), 2-methyl-1*H*-benzo[*d*]imidazole (**2a**, 79 mg, 0.6 mmol), $CoBr_2$ (66 mg, 0.03 mmol), tetrabutylammonium bromide (97 mg, 0.3 mmol, TBAB), and Cs_2CO_3 (244 mg, 0.75 mmol) in DMSO (2.0 mL) was stirred under nitrogen atmosphere at 70 °C for 24 h. The reaction was then quenched by saturated NH_4Cl solution (20 mL) and diluted with EtOAc (20 mL). The organic layer was washed with saturated brine twice, dried over $MgSO_4$, filtered, and concentrated under reduced pressure. The crude product was purified by flash column chromatography (300-400 mesh) using petroleum ether/ethyl acetate (100/1~20/1) as eluent to afford the pure product **3a** (107 mg, 86%). **This result suggested that compound 28 was the possible reaction intermediate.**

Transparent Methods

General information

Unless otherwise stated, all reagents were purchased from commercial suppliers and used without further purification. All reactions were carried out under N_2 atomosphere using undistilled solvent. Melting points were recorded on an Electrothermal digital melting point apparatus. IR spectra were recorded on a FT-IR spectrophotometer using KBr optics. ^{1}H, ^{19}F, and ^{13}C NMR spectra were recorded in $CDCl_3$ or DMSO-d_6 on Bruker Avance or Joel 400 MHz spectrometers. The chemical shifts (δ) are reported in ppm and coupling constants (J) in Hz. High resolution mass spectra (HRMS) were obtained using a commercial apparatus (ESI or EI Source). Column chromatography was generally performed on silica gel (300-400 mesh) or alkali alumina (200-300 mesh) and reactions were monitored by thin layer chromatography (TLC) using UV light to visualize the course of the reactions.

General procedures for the synthesis of perfluoroalkylated naphtho[1,2-*b*]furan/benzofuran derivatives, related to **Scheme 1-4**.

A solution of α-perfluoroalkyl ketone **1** (0.3 mmol), *N*-heterocycle **2a-2v'** (0.6-0.9 mmol), $CoBr_2$ (66 mg, 0.03 mmol), tetrabutylammonium bromide (97 mg, 0.3 mmol, TBAB), and Cs_2CO_3 (244 mg, 0.75 mmol) in DMSO (2.0 mL) was stirred under nitrogen atmosphere at room temperature to 120 ^{o}C for 10-48 h. The reaction was then quenched by saturated NH_4Cl solution (20 mL) and diluted with EtOAc (20 mL). The organic layer was washed with saturated brine twice, dried over $MgSO_4$, filtered, and concentrated under reduced pressure. The crude product was purified by flash column chromatography (300-400 mesh) using petroleum ether/ethyl acetate or dichloromethane/methanol as eluent to afford the pure products **3**-**16**.

Large scale synthesis of perfluoroalkylated naphtho[1,2-*b*]furan 10d, related to **Scheme 1**.

A solution of 2-(perfluorobutyl)-3,4-dihydronaphthalen-1(2*H*)-one (**1a**, 1.09 g, 3 mmol), 1,2,3,9-tetrahydro-4*H*-carbazol-4-one (**2d'**, 1.67 g, 9 mmol), $CoBr_2$ (0.07 g, 0.3 mmol), tetrabutylammonium bromide (0.97 g, 3 mmol, TBAB), and Cs_2CO_3 (2.44 g, 7.5 mmol) in DMSO (10.0 mL) was stirred under nitrogen atmosphere at 100 ^{o}C for 48 h. The reaction was then quenched by saturated NH_4Cl solution (50 mL) and diluted with EtOAc (50 mL). The organic layer was washed with saturated brine twice, dried over $MgSO_4$, filtered, and concentrated under reduced pressure. The crude product was purified by flash column chromatography (300-

400 mesh) using petroleum ether/ethyl acetate (6/1) as eluent to afford the pure product **10d** (0.87 g, 62%).

General procedure for the synthesis of perfluoroalkylated naphtho[1,2-*b*]furan 17-18, related to **Scheme 4**.

A solution of 2-(perfluorobutyl)-3,4-dihydronaphthalen-1(2*H*)-one (**1a**, 109 mg, 0.3 mmol), 4-methylbenzenethiol (**2r'**, 75 mg, 0.6 mmol) or naphthalene-2-thiol (**2s'**, 96 mg, 0.6 mmol), $CoBr_2$ (66 mg, 0.03 mmol), tetrabutylammonium bromide (97 mg, 0.3 mmol, TBAB), and Cs_2CO_3 (244 mg, 0.75 mmol) in DMSO (2.0 mL) was stirred under nitrogen atmosphere at 70 ^{o}C for 24 h. The reaction was then quenched by saturated NH_4Cl solution (20 mL) and diluted with EtOAc (20 mL). The organic layer was washed with saturated brine twice, dried over $MgSO_4$, filtered, and concentrated under reduced pressure. The crude product was purified by flash column chromatography (300-400 mesh) using petroleum ether as eluent to afford the pure product **17** (56 mg, 46%) or **18** (69 mg, 52%).

General procedure for the synthesis of perfluoroalkylated benzo[4,5]imidazo[2,1-*b*]naphtho[2,1-*e*][1,3]oxazine 19, related to **Scheme 4**.

A solution of 2-(perfluorobutyl)-3,4-dihydronaphthalen-1(2*H*)-one (**1a**, 109 mg, 0.3 mmol), 2-chloro-1*H*-benzo[*d*]imidazole (**2t'**, 92 mg, 0.6 mmol), $CoBr_2$ (66 mg, 0.03 mmol), tetrabutylammonium bromide (97 mg, 0.3 mmol, TBAB), and Cs_2CO_3 (244 mg, 0.75 mmol) in DMSO (2.0 mL) was stirred under nitrogen atmosphere at 70 ^{o}C for 36 h. The reaction was then quenched by saturated NH_4Cl solution (20 mL) and diluted with EtOAc (20 mL). The organic layer was washed with saturated brine twice, dried over $MgSO_4$, filtered, and concentrated under reduced pressure. The crude product was purified by flash column chromatography (300-400 mesh) using petroleum ether/ethyl acetate (50/1~3/1) as eluent to afford the pure product **19** (111 mg, 88%).

General procedure for the synthesis of perfluoroalkylated benzo[4,5]imidazo[1,2-*b*]isoquinoline 20, related to **Scheme 4**.

A solution of 2-(perfluorobutyl)-3,4-dihydronaphthalen-1(2*H*)-one (**1a**, 109 mg, 0.3 mmol), 2-

(1*H*-benzo[*d*]imidazol-2-yl)acetonitrile (**2u'**, 94 mg, 0.6 mmol), $CoBr_2$ (66 mg, 0.03 mmol), tetrabutylammonium bromide (97 mg, 0.3 mmol, TBAB), and Cs_2CO_3 (244 mg, 0.75 mmol) in DMSO (2.0 mL) was stirred under nitrogen atmosphere at 70 °C for 24 h. The reaction was then quenched by saturated NH_4Cl solution (20 mL) and diluted with EtOAc (20 mL). The organic layer was washed with saturated brine twice, dried over $MgSO_4$, filtered, and concentrated under reduced pressure. The crude product was purified by flash column chromatography (300-400 mesh) using petroleum ether/ethyl acetate (50/1~3/1) as eluent to afford the pure product **20** (100 mg, 72%).

General procedure for the synthesis of perfluoroalkylated dihydrobenzo[*h*]quinazoline 21, related to **Scheme 4**.

A solution of 2-(perfluorobutyl)-3,4-dihydronaphthalen-1(2*H*)-one (**1a**, 109 mg, 0.3 mmol), 4-bromobenzimidamide (**2v'**, 119 mg, 0.6 mmol), $CoBr_2$ (66 mg, 0.03 mmol), tetrabutylammonium bromide (97 mg, 0.3 mmol, TBAB), and Cs_2CO_3 (244 mg, 0.75 mmol) in DMSO (2.0 mL) was stirred under nitrogen atmosphere at 70 °C for 24 h. The reaction was then quenched by saturated NH_4Cl solution (20 mL) and diluted with EtOAc (20 mL). The organic layer was washed with saturated brine twice, dried over $MgSO_4$, filtered, and concentrated under reduced pressure. The crude product was purified by flash column chromatography (300-400 mesh) using petroleum ether/ethyl acetate (50/1~20/1) as eluent to afford the pure product **21** (111 mg, 73%).

Characterization data for perfluoroalkylated naphtho[1,2-*b*]furan derivatives

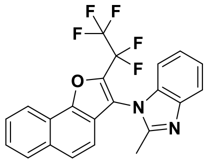

2-Methyl-1-(2-(perfluoroethyl)naphtho[1,2-*b*]furan-3-yl)-1*H*-benzo[*d*]imidazole (3a):

Yield = 71% (89 mg), 0.3 mmol scale. Yellow oil.

IR (KBr): ν = 3063, 1625, 1538, 812, 744 cm^{-1}.

^{1}**H NMR** (400 MHz, $CDCl_3$): δ = 8.46 (d, *J* = 8.2 Hz, 1H), 7.98 (d, *J* = 8.1 Hz, 1H), 7.84 (d, *J* = 8.0 Hz, 1H), 7.77 – 7.70 (m, 2H), 7.67 – 7.63 (m, 1H), 7.32 (td, *J* = 7.8, 1.1 Hz, 1H), 7.23 – 7.18

(m, 1H), 7.14 (d, J = 8.7 Hz, 1H), 7.03 (d, J = 8.0 Hz, 1H), 2.52 (s, 3H) ppm.

^{19}F NMR (376 MHz, $CDCl_3$): δ = -83.36 (t, J = 4.0 Hz, 3F), -113.83 – -116.67 (m, 2F) ppm.

^{13}C NMR (100 MHz, DMSO-D_6): δ = 151.7, 150.7, 142.6, 136.4, 135.5 (t, J = 31.0 Hz), 133.0, 128.8, 128.1, 127.9, 126.6, 123.0, 122.7, 122.6, 120.2, 120.0, 119.5, 118.9, 116.5, 109.7, 13.2 ppm; carbons corresponding to the C_2F_5 group cannot be identified due to C-F coupling.

HRMS m/z: calcd for $C_{22}H_{14}F_5N_2O$ $[M+H]^+$ 417.1021, found: 417.1026.

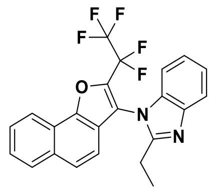

2-Ethyl-1-(2-(perfluoroethyl)naphtho[1,2-*b*]furan-3-yl)-1*H*-benzo[*d*]imidazole (3b):

Yield = 69% (89 mg), 0.3 mmol scale. Yellow solid. M.p. 70.8–72.2 °C.

IR (KBr): ν = 3064, 1616, 1535, 818, 747 cm^{-1}.

^{1}H NMR (400 MHz, $CDCl_3$): δ = 8.50 – 8.44 (m, 1H), 7.99 (d, J = 8.2 Hz, 1H), 7.90 – 7.84 (m, 1H), 7.78 – 7.72 (m, 2H), 7.70 – 7.64 (m, 1H), 7.35 – 7.29 (m, 1H), 7.24 – 7.15 (m, 2H), 7.02 (d, J = 8.0 Hz, 1H), 2.91 – 2.69 (m, 2H), 1.39 (t, J = 7.5 Hz, 3H) ppm.

^{19}F NMR (376 MHz, $CDCl_3$): δ = -82.99 – -83.53 (m, 3F), -113.41 – -116.82 (m, 2F) ppm.

^{13}C NMR (100 MHz, $CDCl_3$): δ = 156.7, 151.2 (t, J = 1.4 Hz), 142.8, 137.0 (t, J = 31.5 Hz), 136.5, 133.2, 128.6, 127.8, 127.7, 126.4, 123.2, 122.9, 122.4 (t, J = 1.5 Hz), 121.0, 120.3, 120.2, 119.5, 116.2, 109.5, 20.8, 11.5 ppm; carbons corresponding to the C_2F_5 group cannot be identified due to C-F coupling.

HRMS m/z: calcd for $C_{23}H_{16}F_5N_2O$ $[M+H]^+$ 431.1177, found: 431.1183.

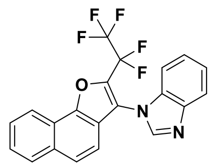

1-(2-(Perfluoroethyl)naphtho[1,2-*b*]furan-3-yl)-1*H*-benzo[*d*]imidazole (3c):

Yield = 83% (100 mg), 0.3 mmol scale. Yellow oil.

IR (KBr): ν = 3120, 1628, 1614, 829, 746 cm^{-1}.

^{1}H NMR (400 MHz, DMSO-D_6): δ = 8.61 (s, 1H), 8.42 – 8.37 (m, 1H), 8.18 (d, J = 8.0 Hz, 1H),

7.94 (d, *J* = 8.6 Hz, 1H), 7.88 – 7.85 (m, 1H), 7.84 – 7.79 (m, 1H), 7.78 – 7.72 (m, 1H), 7.39 – 7.29 (m, 4H) ppm.

^{19}F NMR (376 MHz, DMSO-*D$_6$*): δ = -83.16 (t, *J* = 3.5 Hz, 3F), -113.87 (q, *J* = 3.4 Hz, 2F) ppm.

^{13}C NMR (100 MHz, DMSO-*D$_6$*): δ = 150.2, 144.4, 143.0, 134.7, 134.4 (t, *J* = 31.0 Hz), 132.9, 129.0, 128.3, 128.0, 126.5, 124.1, 123.3 (t, *J* = 1.8 Hz), 123.1, 120.1, 120.1, 120.0, 119.7, 116.9, 110.4 ppm; carbons corresponding to the C_2F_5 group cannot be identified due to C-F coupling.

HRMS m/z: calcd for $C_{21}H_{12}F_5N_2O$ $[M+H]^+$ 403.0864, found: 403.0866.

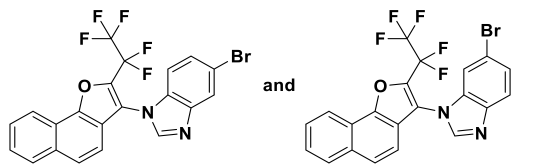

5-Bromo-1-(2-(perfluoroethyl)naphtho[1,2-*b*]furan-3-yl)-1*H*-benzo[*d*]imidazole (3d-I) and 6-bromo-1-(2-(perfluoroethyl)naphtho[1,2-*b*]furan-3-yl)-1H-benzo[*d*]imidazole (3d-II):

3d-I and its isomer **3d-II** were separable but unidentified regioisomers.

Total yield = 62% (90 mg, **3d-I**/**3d-II** = 1/1), 0.3 mmol scale.

Isomer I: Yellow solid. M.p. 108.8–110.6 ^{o}C.

IR (KBr): ν = 3069, 1719, 1491, 818, 792 cm^{-1}.

^{1}H NMR (400 MHz, CDCl$_3$): δ = 8.48 – 8.43 (m, 1H), 8.14 – 8.06 (m, 2H), 8.04 – 7.99 (m, 1H), 7.80 – 7.74 (m, 2H), 7.72 – 7.67 (m, 1H), 7.45 (dd, *J* = 8.6, 1.6 Hz, 1H), 7.28 – 7.24 (m, 1H), 7.15 (d, *J* = 8.6 Hz, 1H) ppm.

^{19}F NMR (376 MHz, CDCl$_3$): δ = -83.56 (t, *J* = 4.1 Hz, 3F), -112.49 – -115.54 (m, 2F) ppm.

^{13}C NMR (100 MHz, CDCl$_3$): δ = 151.0 (t, *J* = 1.3 Hz), 144.8 (t, *J* = 3.3 Hz), 144.1 (m), 135.5 (t, *J* = 31.8 Hz), 133.6, 133.2, 128.7, 128.0, 127.9, 127.5, 126.4, 123.7, 122.2 (t, *J* = 1.5 Hz), 121.0, 120.3, 119.5, 116.5, 116.4, 111.6 ppm; carbons corresponding to the C_2F_5 group cannot be identified due to C-F coupling.

HRMS m/z: calcd for $C_{21}H_{11}BrF_5N_2O$ $[M+H]^+$ 480.9975, found: 480.9975.

Isomer II: Yellow solid. M.p. 108.6–109.6 ^{o}C.

IR (KBr): ν = 3047, 1620, 1529, 811, 749 cm^{-1}.

^{1}H NMR (400 MHz, CDCl$_3$): δ = 8.49 – 8.42 (m, 1H), 8.07 (s, 1H), 8.01 (d, *J* = 8.5 Hz, 1H), 7.80 (dd, *J* = 8.7, 5.3 Hz, 2H), 7.75 (dt, *J* = 8.1, 0.9 Hz, 1H), 7.72 – 7.66 (m, 1H), 7.51 (dd, *J* = 8.6,

1.6 Hz, 1H), 7.43 (s, 1H), 7.28 (d, *J* = 8.7 Hz, 1H) ppm.

^{19}F NMR (376 MHz, $CDCl_3$): δ = -83.54 (t, *J* = 3.9 Hz, 3F), -112.45 – -115.58 (m, 2F) ppm.

^{13}C NMR (100 MHz, $CDCl_3$): δ = 151.0 (t, *J* = 1.2 Hz), 143.8 (m), 142.4 (m), 135.6 (t, *J* = 31.9 Hz), 133.2, 128.7, 128.0, 127.9, 126.9, 126.5, 122.1, 122.1 (t, *J* = 1.9 Hz), 121.0, 120.3, 119.5, 117.7, 116.3, 113.4 ppm; carbons corresponding to the C_2F_5 group cannot be identified due to C-F coupling.

HRMS m/z: calcd for $C_{21}H_{11}BrF_5N_2O$ $[M+H]^+$ 480.9969, found: 480.9965.

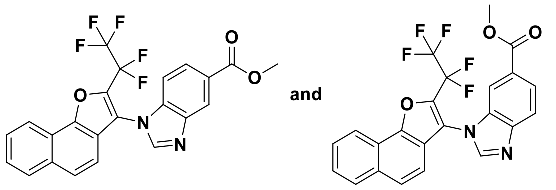

Methyl 1-(2-(perfluoroethyl)naphtho[1,2-*b*]furan-3-yl)-1*H*-benzo[*d*]imidazole-5-carboxylate (3e-I) and methyl 1-(2-(perfluoroethyl)naphtho[1,2-*b*]furan-3-yl)-1*H*-benzo[*d*]imidazole-6-carboxylate (3e-II):

3e-I and its isomer **3e-II** were inseparable regioisomers.

Total yield = 68% (94 mg, **3e-I**/**3e-II** = 1/1), 0.3 mmol scale. Yellow solid.

Isomer I & II: **IR** (KBr): ν = 3187, 1719, 1620, 1529, 811, 749 cm^{-1}.

^{1}H NMR (400 MHz, $CDCl_3$): δ = 8.69 – 8.66 (m, 1H), 8.48 – 8.43 (m, 2H), 8.21 (d, *J* = 16.0 Hz, 2H), 8.15 – 8.10 (m, 1H), 8.09 – 8.06 (m, 1H), 8.04 – 7.94 (m, 4H), 7.80 – 7.72 (m, 4H), 7.72 – 7.65 (m, 2H), 7.31 (d, *J* = 8.5 Hz, 1H), 7.28 – 7.24 (m, 2H), 3.98 (t, *J* = 1.2 Hz, 3H), 3.87 (t, *J* = 1.2 Hz, 3H) ppm.

^{19}F NMR (376 MHz, $CDCl_3$): δ = -83.27 – -83.87 (m, 3F), -111.73 – -115.99 (m, 2F) ppm.

^{13}C NMR (100 MHz, $CDCl_3$): δ = 167.0, 166.8, 151.0, 146.7, 145.6, 144.6, 144.6, 143.1, 137.6, 135.6 (t, *J* = 31.5 Hz), 135.5 (t, *J* = 31.6 Hz), 134.3, 133.2, 133.2, 128.6, 128.6, 127.9, 127.9, 127.8, 127.8, 126.5, 126.4, 126.4, 125.8, 125.8, 124.7, 123.1, 122.1 (t, *J* = 1.7 Hz), 122.1 (t, *J* = 1.5 Hz), 120.9, 120.5, 120.2, 120.2, 119.6, 119.5, 116.3, 116.3, 112.4, 110.0, 52.1, 52.1 ppm; carbons corresponding to the C_2F_5 group cannot be identified due to C-F coupling.

HRMS m/z: calcd for $C_{23}H_{14}F_5N_2O_3$ $[M+H]^+$ 461.0919, found: 461.0925.

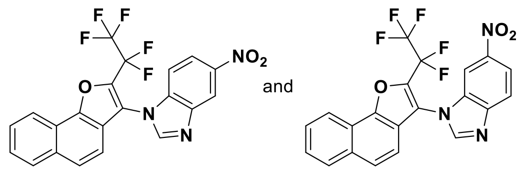

5-Nitro-1-(2-(perfluoroethyl)naphtho[1,2-*b*]furan-3-yl)-1*H*-benzo[*d*]imidazole (3f-I) and 6-nitro-1-(2-(perfluoroethyl)naphtho[1,2-*b*]furan-3-yl)-1*H*-benzo[*d*]imidazole (3f-II):

3f-I and its isomer **3f-II** were separable but unidentified regioisomers.

Yield = 87% (117 mg, **3f-I**/**3f-II** = 1/1), 0.3 mmol scale.

Isomer I: Yellow solid. M.p. 131.4–132.6 °C.

IR (KBr): ν = 3071, 1629, 1523, 1345, 801, 739 cm^{-1}.

^{1}H NMR (400 MHz, $CDCl_3$): δ = 8.86 (d, *J* = 2.1 Hz, 1H), 8.51 – 8.44 (m, 1H), 8.33 – 8.25 (m, 2H), 8.07 – 8.01 (m, 1H), 7.83 (d, *J* = 8.7 Hz, 1H), 7.81 – 7.76 (m, 1H), 7.75 – 7.69 (m, 1H), 7.36 (d, *J* = 9.0 Hz, 1H), 7.26 (d, *J* = 8.7 Hz, 1H) ppm.

^{19}F NMR (376 MHz, $CDCl_3$): δ = -83.32 – -83.75 (m, 3F), -112.46 – -115.70 (m, 2F) ppm.

^{13}C NMR (100 MHz, $CDCl_3$): δ = 151.2 (t, *J* = 1.1 Hz), 146.3, 144.7, 143.0, 138.5, 136.0 (t, *J* = 31.7 Hz), 133.3, 128.8, 128.2, 128.1, 126.8, 121.6 (t, *J* = 1.7 Hz), 120.9, 120.3, 120.1, 119.3, 117.6, 115.9, 110.5 ppm; carbons corresponding to the C_2F_5 group cannot be identified due to C-F coupling.

HRMS m/z: calcd for $C_{21}H_{11}F_5N_3O_3$ [M+H]$^+$ 448.0721, found: 448.0719.

Isomer II: Yellow solid. M.p. 131.6–132.9 °C.

IR (KBr): ν = 3319, 1770, 1633, 1523, 803, 732 cm^{-1}.

^{1}H NMR (400 MHz, $CDCl_3$): δ = 8.51 – 8.46 (m, 1H), 8.36 – 8.32 (m, 2H), 8.21 (d, *J* = 2.1 Hz, 1H), 8.06 – 8.02 (m, 2H), 7.84 – 7.77 (m, 2H), 7.75 – 7.70 (m, 1H), 7.24 (s, 1H) ppm.

^{19}F NMR (376 MHz, $CDCl_3$): δ = -83.25 – -83.78 (m, 3F), -112.27 – -115.81 (m, 2F) ppm.

^{13}C NMR (100 MHz, $CDCl_3$): δ = 151.2 (t, *J* = 1.3 Hz), 147.6, 147.4 (t, *J* = 1.7 Hz), 144.9, 136.0 (t, *J* = 31.5 Hz), 134.1, 133.4, 128.8, 128.2, 128.1, 127.0, 121.3, 121.3, 121.0, 120.4, 119.3, 119.2, 115.8, 107.2 ppm; carbons corresponding to the C_2F_5 group cannot be identified due to C-F coupling.

HRMS m/z: calcd for $C_{21}H_{11}F_5N_3O_3$ [M+H]$^+$ 448.0715, found: 448.0719.

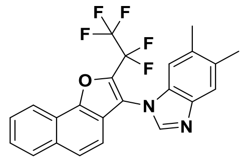

5,6-Dimethyl-1-(2-(perfluoroethyl)naphtho[1,2-*b*]furan-3-yl)-1*H*-benzo[*d*]imidazole (3g):

Yield = 39% (50 mg), 0.3 mmol scale. Colorless oil.

IR (KBr): ν = 2924, 1628, 1496, 810, 757 cm^{-1}.

^{1}H NMR (400 MHz, $CDCl_3$): δ = 8.48 – 8.43 (m, 1H), 8.01 (d, *J* = 8.1 Hz, 1H), 7.98 (s, 1H), 7.78 – 7.73 (m, 2H), 7.71 – 7.65 (m, 2H), 7.30 (d, *J* = 8.7 Hz, 1H), 7.04 (s, 1H), 2.42 (s, 3H), 2.32 (s, 3H) ppm.

^{19}F NMR (376 MHz, $CDCl_3$): δ = -83.54 (t, *J* = 4.1 Hz, 3F), -112.37 – -115.49 (m, 2F) ppm.

^{13}C NMR (100 MHz, $CDCl_3$): δ = 150.9, 142.4, 141.8, 135.3 (t, *J* = 31.7 Hz), 133.7, 133.2, 133.1, 132.4, 128.6, 127.8, 127.7, 126.1, 123.0 (t, *J* = 2.0 Hz), 121.0, 120.7, 120.3, 119.9, 116.9, 110.4, 20.5, 20.3 ppm; carbons corresponding to the C_2F_5 group cannot be identified due to C-F coupling.

HRMS m/z: calcd for $C_{23}H_{16}F_5N_2O$ $[M+H]^+$ 431.1177, found: 431.1179.

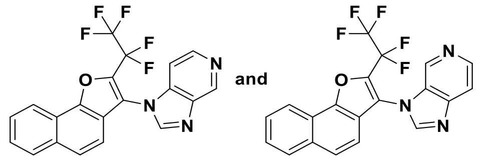

1-(2-(Perfluoroethyl)naphtho[1,2-*b*]furan-3-yl)-1*H*-imidazo[4,5-*c*]pyridine (3h-I) and 3-(2-(perfluoroethyl)naphtho[1,2-*b*]furan-3-yl)-3*H*-imidazo[4,5-*c*]pyridine (3h-II):

3h-I and its isomer **3h-II** were separable but unidentified regioisomers.

Total yield = 64% (77 mg, **3h-I**/**3h-II** = 1/1), 0.3 mmol scale.

Isomer I: Brown solid. M.p. 130.9–132.3 ^{o}C.

IR (KBr): ν = 3053, 1629, 1609, 823, 751 cm^{-1}.

^{1}H NMR (400 MHz, $CDCl_3$): δ = 8.75 (s, 1H), 8.62 (d, *J* = 5.6 Hz, 1H), 8.50 – 8.45 (m, 1H), 8.22 (s, 1H), 8.03 (d, *J* = 8.1 Hz, 1H), 7.91 – 7.87 (m, 1H), 7.83 – 7.76 (m, 2H), 7.74 – 7.68 (m, 1H), 7.30 (d, *J* = 8.7 Hz, 1H) ppm.

^{19}F NMR (376 MHz, $CDCl_3$): δ = -83.27 – -83.77 (m, 3F), -112.19 – -115.63 (m, 2F) ppm.

^{13}C NMR (100 MHz, $CDCl_3$): δ = 151.1 (t, *J* = 1.1 Hz), 148.7, 145.9 (t, *J* = 2.0 Hz), 143.3, 135.6

(t, J = 32.0 Hz), 133.9, 133.3, 132.2 (t, J = 1.6 Hz), 128.7, 128.1, 128.0, 126.7, 121.7 (t, J = 1.5 Hz), 120.9, 120.3, 119.3, 116.1, 115.5 ppm; carbons corresponding to the C_2F_5 group cannot be identified due to C-F coupling.

HRMS m/z: calcd for $C_{20}H_{11}F_5N_3O$ $[M+H]^+$ 404.0823, found: 404.0829.

Isomer II: Yellow solid. M.p. 170.6–171.7 °C.

IR (KBr): ν = 3462, 1632, 1607, 816, 750 cm^{-1}.

^{1}H NMR (400 MHz, $CDCl_3$): δ = 9.29 (s, 1H), 8.53 (d, J = 5.4 Hz, 1H), 8.47 (d, J = 8.3 Hz, 1H), 8.16 (d, J = 1.0 Hz, 1H), 8.03 (d, J = 8.1 Hz, 1H), 7.85 – 7.75 (m, 2H), 7.74 – 7.68 (m, 1H), 7.28 – 7.25 (m, 2H) ppm.

^{19}F NMR (376 MHz, $CDCl_3$): δ = -83.55 (d, J = 2.8 Hz, 3F), -112.55 – -115.65 (m, 2F) ppm.

^{13}C NMR (100 MHz, $CDCl_3$): δ = 151.1, 144.2, 143.8, 143.6, 140.4 (m), 139.4 (t, J = 2.1 Hz), 135.6 (t, J = 30.9 Hz), 133.3, 128.7, 128.1, 128.0, 126.6, 121.6 (m), 120.9, 120.3, 119.3, 116.1, 105.7 ppm; carbons corresponding to the C_2F_5 group cannot be identified due to C-F coupling.

HRMS m/z: calcd for $C_{20}H_{11}F_5N_3O$ $[M+H]^+$ 404.0817, found: 404.0825.

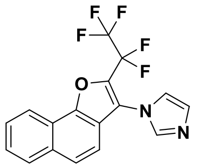

1-(2-(Perfluoroethyl)naphtho[1,2-*b*]furan-3-yl)-1*H*-imidazole (4a):

Yield = 44% (47 mg), 0.3 mmol scale. Yellow oil.

IR (KBr): ν = 2963, 1630, 1492, 809, 748 cm^{-1}.

^{1}H NMR (400 MHz, $CDCl_3$): δ = 8.36 – 8.32 (m, 1H), 7.94 (d, J = 8.0 Hz, 1H), 7.77 – 7.72 (m, 2H), 7.69 – 7.64 (m, 1H), 7.63 – 7.58 (m, 1H), 7.40 (d, J = 8.7 Hz, 1H), 7.26 – 7.25 (m, 1H), 7.21 (s, 1H) ppm.

^{19}F NMR (376 MHz, $CDCl_3$): δ = -83.55 (t, J = 3.3 Hz, 3F), -113.56 – -113.62 (m, 2F) ppm.

^{13}C NMR (100 MHz, $CDCl_3$): δ = 150.6 (t, J = 1.3 Hz), 138.1, 138.1, 133.8 (t, J = 31.5 Hz), 133.2, 130.4, 128.6, 127.8, 127.7, 126.3, 124.2 (t, J = 1.4 Hz), 120.8, 120.3, 119.6, 116.2 ppm; carbons corresponding to the C_2F_5 group cannot be identified due to C-F coupling.

HRMS m/z: calcd for $C_{17}H_{10}F_5N_2O$ $[M+H]^+$ 353.0708, found: 353.0710.

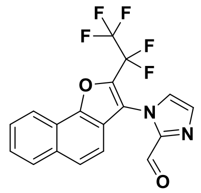

1-(2-(Perfluoroethyl)naphtho[1,2-*b*]furan-3-yl)-1*H*-imidazole-2-carbaldehyde (4b):

Yield = 55% (63 mg), 0.3 mmol scale. White solid. M.p. 177.5–178.8 °C.

IR (KBr): ν = 3441, 1770, 1690, 1634, 826, 776 cm^{-1}.

^{1}H NMR (400 MHz, $CDCl_3$): δ = 9.86 (d, *J* = 0.9 Hz, 1H), 8.39 (d, *J* = 8.2 Hz, 1H), 7.96 (d, *J* = 8.2 Hz, 1H), 7.75 (d, *J* = 8.7 Hz, 1H), 7.73 – 7.67 (m, 1H), 7.67 – 7.60 (m, 1H), 7.54 (d, *J* = 1.0 Hz, 1H), 7.34 (s, 1H), 7.19 (d, *J* = 8.7 Hz, 1H) ppm.

^{19}F NMR (376 MHz, $CDCl_3$): δ = -83.66 (t, *J* = 4.1 Hz, 3F), -113.17 – -117.19 (m, 2F) ppm.

^{13}C NMR (100 MHz, $CDCl_3$): δ = 180.0, 150.5 (t, *J* = 1.4 Hz), 144.4, 134.9 (t, *J* = 31.6 Hz), 133.1, 132.3, 128.6, 127.7, 127.5, 127.5, 126.3, 123.9 (t, *J* = 2.6 Hz), 120.9, 120.2, 120.1, 115.9 ppm; carbons corresponding to the C_2F_5 group cannot be identified due to C-F coupling.

HRMS m/z: calcd for $C_{18}H_{10}F_5N_2O_2$ $[M+H]^+$ 381.0657, found: 381.0660.

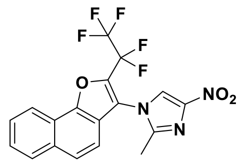

2-Methyl-4-nitro-1-(2-(perfluoroethyl)naphtho[1,2-*b*]furan-3-yl)-1*H*-imidazole (4c):

Yield = 94% (116 mg), 0.3 mmol scale. White solid. M.p. 112.5–113.7 °C.

IR (KBr): ν = 3160, 1629, 1547, 819, 753 cm^{-1}.

^{1}H NMR (400 MHz, $CDCl_3$): δ = 8.47 – 8.42 (m, 1H), 8.06 (d, *J* = 8.0 Hz, 1H), 7.92 (d, *J* = 8.4 Hz, 2H), 7.82 – 7.76 (m, 1H), 7.76 – 7.70 (m, 1H), 7.37 (d, *J* = 8.7 Hz, 1H), 2.38 (s, 3H) ppm.

^{19}F NMR (376 MHz, $CDCl_3$): δ = -83.30 – -83.81 (m, 3F), -113.27 – -116.45 (m, 2F) ppm.

^{13}C NMR (100 MHz, $CDCl_3$): δ = 151.0 (t, *J* = 1.4 Hz), 147.7, 146.4, 136.2 (t, *J* = 31.6 Hz), 133.3, 128.7, 128.2, 128.1, 127.3, 122.1 (t, *J* = 2.2 Hz), 121.2, 120.7, 120.2, 119.1, 115.1, 12.9 ppm; carbons corresponding to the C_2F_5 group cannot be identified due to C-F coupling.

HRMS m/z: calcd for $C_{18}H_{11}F_5N_3O_3$ $[M+H]^+$ 412.0715, found: 412.0717.

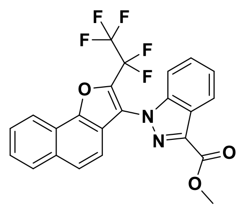

Methyl 1-(2-(perfluoroethyl)naphtho[1,2-*b*]furan-3-yl)-1*H*-indazole-3-carboxylate (5a):

Yield = 79% (109 mg), 0.3 mmol scale. White solid. M.p. 142.6–143.4 ^{o}C.

IR (KBr): ν = 3071, 1710, 1629, 817, 750 cm^{-1}.

^{1}H NMR (400 MHz, CDCl$_3$): δ = 8.43 (d, *J* = 7.8 Hz, 1H), 8.41 – 8.36 (m, 1H), 7.97 (d, *J* = 8.1 Hz, 1H), 7.76 – 7.69 (m, 2H), 7.67 – 7.61 (m, 1H), 7.53 – 7.45 (m, 1H), 7.47 – 7.41 (m, 1H), 7.36 (d, *J* = 8.3 Hz, 1H), 7.32 (d, *J* = 8.7 Hz, 1H), 4.09 (s, 3H) ppm.

^{19}F NMR (376 MHz, CDCl$_3$): δ = -83.08 – -83.18 (m, 3F), -113.22 – -115.69 (m, 2F) ppm.

^{13}C NMR (100 MHz, CDCl$_3$): δ = 162.6, 150.7 (t, *J* = 1.3 Hz), 142.6, 138.5, 136.3 (t, *J* = 32.5 Hz), 133.1, 128.6, 128.2, 127.6, 127.5, 126.1, 125.1 (t, *J* = 1.9 Hz), 124.1, 123.5, 122.4, 120.8, 120.2, 120.1, 116.9, 110.1, 52.3 ppm; carbons corresponding to the C_2F_5 group cannot be identified due to C-F coupling.

HRMS m/z: calcd for $C_{23}H_{14}F_5N_2O_3$ [M+H]$^+$ 461.0919, found: 461.0919.

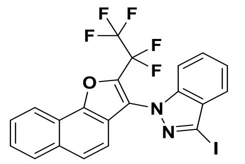

3-Iodo-1-(2-(perfluoroethyl)naphtho[1,2-*b*]furan-3-yl)-1*H*-indazole (5b):

Yield = 70% (111 mg), 0.3 mmol scale. Brown solid. M.p. 49.8–51.7 ^{o}C.

IR (KBr): ν = 3066, 1756, 1613, 810, 746 cm^{-1}.

^{1}H NMR (400 MHz, CDCl$_3$): δ = 8.40 (d, *J* = 8.1 Hz, 1H), 7.94 (d, *J* = 8.1 Hz, 1H), 7.72 – 7.66 (m, 2H), 7.64 – 7.58 (m, 2H), 7.51 – 7.45 (m, 1H), 7.36 – 7.28 (m, 3H) ppm.

^{19}F NMR (376 MHz, CDCl$_3$): δ = -82.99 (t, *J* = 2.9 Hz, 3F), -114.11 (s, 2F) ppm.

^{13}C NMR (100 MHz, CDCl$_3$): δ = 150.7 (t, *J* = 1.0 Hz), 141.8, 135.4 (t, *J* = 32.3 Hz), 133.1, 128.9, 128.8, 128.6, 127.5, 127.4, 125.9, 125.4 (t, *J* = 1.9 Hz), 122.7, 121.9, 120.9, 120.2, 120.1, 117.3, 109.9, 97.1 ppm; carbons corresponding to the C_2F_5 group cannot be identified due to C-F coupling.

HRMS m/z: calcd for $C_{21}H_{11}F_5IN_2O$ [M+H]$^+$ 528.9831, found: 528.9831.

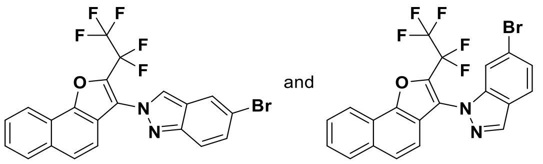

5-Bromo-2-(2-(perfluoroethyl)naphtho[1,2-*b*]furan-3-yl)-2*H*-indazole (5c-I) and 6-bromo-1-(2-(perfluoroethyl)naphtho[1,2-*b*]furan-3-yl)-1*H*-indazole (5c-II):

5c-I and its isomer **5c-II** were separable and identified regioisomers.

Total yield = 60% (87 mg, **5c-I**/**5c-II** = 1:2), 0.3 mmol scale.

5c-I: Yellow solid. M.p. 120.3–123.0 °C.

IR (KBr): ν = 3440, 1626, 1536, 801, 756 cm^{-1}.

^{1}H NMR (400 MHz, $CDCl_3$): δ = 8.44 – 8.39 (m, 1H), 8.34 – 8.30 (m, 1H), 8.06 – 8.03 (m, 1H), 8.02 – 7.97 (m, 1H), 7.84 – 7.75 (m, 2H), 7.75 – 7.70 (m, 1H), 7.69 – 7.63 (m, 2H), 7.27 (d, *J* = 7.5 Hz, 1H) ppm.

^{19}F NMR (376 MHz, $CDCl_3$): δ = -83.18 (t, *J* = 4.0 Hz, 3F), -111.91 – -113.37 (m, 2F) ppm.

^{13}C NMR (100 MHz, $CDCl_3$): δ = 151.0, 150.7 (t, *J* = 1.0 Hz), 133.3, 132.8, 128.6, 127.9 (t, *J* = 1.4 Hz), 127.6, 127.6, 127.0, 126.5 (t, *J* = 3.3 Hz), 126.3, 121.9, 121.7, 120.7, 120.6, 120.4, 120.4, 119.5, 118.0 ppm; carbons corresponding to the C_2F_5 group cannot be identified due to C-F coupling.

HRMS m/z: calcd for $C_{21}H_{11}BrF_5N_2O$ [M+H]$^+$ 480.9975, found: 480.9975.

5c-II: Yellow solid. M.p. 132.1–133.8 °C.

IR (KBr): ν = 3451, 1630, 1478, 810, 749 cm^{-1}.

^{1}H NMR (400 MHz, $CDCl_3$): δ = 8.44 (d, *J* = 8.2 Hz, 1H), 8.31 (t, *J* = 1.0 Hz, 1H), 7.98 (d, *J* = 8.1 Hz, 1H), 7.78 – 7.69 (m, 3H), 7.68 – 7.62 (m, 1H), 7.54 (s, 1H), 7.42 – 7.37 (m, 1H), 7.35 – 7.31 (m, 1H) ppm.

^{19}F NMR (376 MHz, $CDCl_3$): δ = -83.10 (t, *J* = 3.4 Hz, 3F), -114.26 (s, 2F) ppm.

^{13}C NMR (100 MHz, $CDCl_3$): δ = 150.7 (t, *J* = 1.9 Hz), 142.1, 137.0, 135.7 (t, *J* = 1.3 Hz), 133.1, 128.6, 127.6, 127.5, 126.0, 125.8, 125.5 (t, *J* = 1.0 Hz), 123.4, 122.4, 122.2, 121.0, 120.3, 120.1, 117.1, 112.9 ppm; carbons corresponding to the C_2F_5 group cannot be identified due to C-F coupling.

HRMS m/z: calcd for $C_{21}H_{11}BrF_5N_2O$ [M+H]$^+$ 480.9969, found: 480.9975.

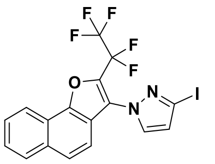

3-Iodo-1-(2-(perfluoroethyl)naphtho[1,2-*b*]furan-3-yl)-1*H*-pyrazole (6a):

Yield = 43% (62 mg), 0.3 mmol scale. Yellow solid. M.p. 82.8–83.3 ^{o}C.

IR (KBr): ν = 3447, 1633, 1475, 811, 741 cm^{-1}.

^{1}H NMR (400 MHz, $CDCl_3$): δ = 8.39 – 8.34 (m, 1H), 7.99 – 7.95 (m, 1H), 7.88 (d, *J* = 0.4 Hz, 1H), 7.86 – 7.84 (m, 1H), 7.78 (d, *J* = 8.6 Hz, 1H), 7.73 – 7.67 (m, 2H), 7.66 – 7.61 (m, 1H) ppm.

^{19}F NMR (376 MHz, $CDCl_3$): δ = -83.20 (t, *J* = 4.0 Hz, 3F), -112.24 – -113.07 (m, 2F) ppm.

^{13}C NMR (100 MHz, $CDCl_3$): δ = 150.5 (t, *J* = 1.3 Hz), 147.3, 136.1 (t, *J* = 3.3 Hz), 133.1, 132.0 (t, *J* = 32.2 Hz), 128.5, 127.5, 127.0 (m), 125.9, 120.7, 120.3, 119.9, 119.1, 118.0, 59.0 ppm; carbons corresponding to the C_2F_5 group cannot be identified due to C-F coupling.

HRMS m/z: calcd for $C_{17}H_9F_5IN_2O$ [M+H]$^{+}$ 478.9674, found: 478.9675.

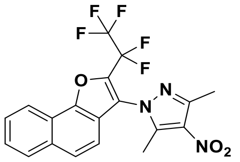

3,5-Dimethyl-4-nitro-1-(2-(perfluoroethyl)naphtho[1,2-*b*]furan-3-yl)-1*H*-pyrazole (6b):

Yield = 92% (117 mg), 0.3 mmol scale. White solid. M.p. 155.0–156.2 ^{o}C.

IR (KBr): ν = 3447, 1633, 1532, 811, 742 cm^{-1}.

^{1}H NMR (400 MHz, $CDCl_3$): δ = 8.45 – 8.36 (m, 1H), 7.99 (d, *J* = 8.0 Hz, 1H), 7.82 (d, *J* = 8.6 Hz, 1H), 7.75 – 7.70 (m, 1H), 7.69 – 7.64 (m, 1H), 7.36 (d, *J* = 8.7 Hz, 1H), 2.65 (s, 3H), 2.58 (s, 3H) ppm.

^{19}F NMR (376 MHz, $CDCl_3$): δ = -83.19 (t, *J* = 2.6 Hz, 3F), -114.21 – -116.27 (m, 2F) ppm.

^{13}C NMR (100 MHz, $CDCl_3$): δ = 150.8 (t, *J* = 1.1 Hz), 148.1, 143.7, 136.8 (t, *J* = 32.1 Hz), 133.2, 132.2, 128.6, 127.9, 127.7, 126.7, 124.3 (t, *J* = 1.8 Hz), 120.7, 120.2, 119.7, 116.0, 14.1, 12.2 ppm; carbons corresponding to the C_2F_5 group cannot be identified due to C-F coupling.

HRMS m/z: calcd for $C_{19}H_{13}F_5N_3O_3$ [M+H]$^{+}$ 426.0872, found: 426.0880.

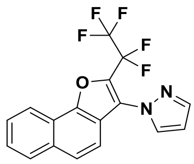

1-(2-(Perfluoroethyl)naphtho[1,2-*b*]furan-3-yl)-1*H*-pyrazole (6c):

Yield = 46% (49 mg), 0.3 mmol scale. White solid. M.p. 78.9–79.1 °C.

IR (KBr): ν = 3077, 1634, 1532, 811, 742 cm^{-1}.

^{1}H NMR (400 MHz, CDCl$_3$): δ = 8.37 (d, J = 8.1 Hz, 1H), 7.96 (d, J = 8.0 Hz, 1H), 7.89 (d, J = 1.6 Hz, 1H), 7.85 – 7.81 (m, 1H), 7.81 – 7.74 (m, 2H), 7.72 – 7.58 (m, 2H), 6.55 (t, J = 2.1 Hz, 1H) ppm.

^{19}F NMR (376 MHz, CDCl$_3$): δ = -83.25 (t, J = 2.3 Hz, 3F), -112.49 – -112.62 (m, 2F) ppm.

^{13}C NMR (100 MHz, CDCl$_3$): δ = 150.5 (t, J = 1.3 Hz), 142.3, 133.1, 132.1 (t, J = 1.3 Hz), 131.6 (t, J = 32.8 Hz), 128.5, 127.9 (t, J = 2.1 Hz), 127.3, 125.7, 122.8, 120.8, 120.3, 119.5 (t, J = 1.0 Hz), 118.5, 107.5 ppm; carbons corresponding to the C_2F_5 group cannot be identified due to C-F coupling.

HRMS m/z: calcd for $C_{17}H_{10}F_5N_2O$ [M+H]$^+$ 353.0708, found: 353.0714.

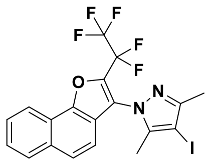

4-Iodo-3,5-dimethyl-1-(2-(perfluoroethyl)naphtho[1,2-*b*]furan-3-yl)-1*H*-pyrazole (6d):

Yield = 75% (114 mg), 0.3 mmol scale. White solid. M.p. 80.3–81.8 °C.

IR (KBr): ν = 3134, 1628, 1543, 804, 744 cm^{-1}.

^{1}H NMR (400 MHz, CDCl$_3$): δ = 8.41 – 8.35 (m, 1H), 7.97 (d, J = 8.1 Hz, 1H), 7.76 (d, J = 8.6 Hz, 1H), 7.72 – 7.67 (m, 1H), 7.65 – 7.60 (m, 1H), 7.36 (d, J = 8.7 Hz, 1H), 2.34 (s, 3H), 2.22 (s, 3H) ppm.

^{19}F NMR (376 MHz, CDCl$_3$): δ = -83.13 – -83.20 (m, 3F), -112.58 – -117.51 (m, 2F) ppm.

^{13}C NMR (100 MHz, CDCl$_3$): δ = 152.5, 150.5 (t, J = 1.4 Hz), 143.6, 136.1 (t, J = 32.0 Hz), 133.1, 128.6, 127.6, 127.4, 126.3 (t, J = 1.8 Hz), 126.1, 120.9, 120.4 (t, J = 0.9 Hz), 120.2, 116.8, 64.8, 14.3, 12.2 ppm; carbons corresponding to the C_2F_5 group cannot be identified due to C-F

coupling.

HRMS m/z: calcd for $C_{19}H_{13}F_5IN_2O$ $[M+H]^+$ 506.9987, found: 506.9995.

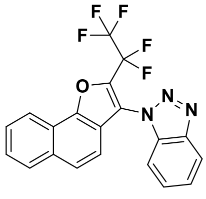

1-(2-(Perfluoroethyl)naphtho[1,2-*b*]furan-3-yl)-1*H*-benzo[*d*][1,2,3]triazole (7a):

Yield = 98% (119 mg), 0.3 mmol scale. Pink solid. M.p. 71.1–73.2 °C.

IR (KBr): ν = 3056, 1634, 1492, 811, 747 cm^{-1}.

^{1}H NMR (400 MHz, $CDCl_3$): δ = 8.45 – 8.38 (m, 1H), 8.25 – 8.20 (m, 1H), 7.97 (d, *J* = 8.1 Hz, 1H), 7.76 – 7.69 (m, 2H), 7.67 – 7.62 (m, 1H), 7.61 – 7.55 (m, 1H), 7.52 – 7.45 (m, 2H), 7.28 (d, *J* = 8.7 Hz, 1H) ppm.

^{19}F NMR (376 MHz, $CDCl_3$): δ = -83.15 (d, *J* = 2.3 Hz, 3F), -114.25 (s, 2F) ppm.

^{13}C NMR (100 MHz, $CDCl_3$): δ = 150.9 (t, *J* = 1.2 Hz), 145.6, 135.7 (t, *J* = 32.3 Hz), 134.2, 133.1, 128.9, 128.6, 127.8, 127.7, 126.4, 124.7, 122.8, 120.8 (t, *J* = 1.9 Hz), 120.4, 120.2, 119.4, 116.6, 109.6 ppm; carbons corresponding to the C_2F_5 group cannot be identified due to C-F coupling.

HRMS m/z: calcd for $C_{20}H_{11}F_5N_3O$ $[M+H]^+$ 404.0817, found: 404.0826.

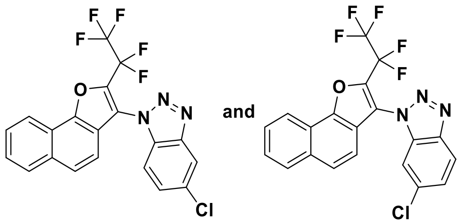

5-Chloro-1-(2-(perfluoroethyl)naphtho[1,2-*b*]furan-3-yl)-1*H*-benzo[*d*][1,2,3]triazole (7b-I) and 6-chloro-1-(2-(perfluoroethyl)naphtho[1,2-*b*]furan-3-yl)-1*H*-benzo[*d*][1,2,3]triazole (7b-II):

7b-I and its isomer **7b-II** were separable but unidentified regioisomers.

Total yield = 99% (130 mg, **7b-I**/**7b-II** = 1/1), 0.3 mmol scale.

Isomer I: White solid. M.p. 117.8–120.1 °C.

IR (KBr): ν = 3065, 1632, 1610, 809, 759 cm^{-1}.

^{1}H NMR (400 MHz, $CDCl_3$): δ = 8.47 – 8.42 (m, 1H), 8.21 (dd, *J* = 1.8, 0.7 Hz, 1H), 8.00 (d, *J* = 8.1 Hz, 1H), 7.81 – 7.74 (m, 2H), 7.71 – 7.66 (m, 1H), 7.56 (dd, *J* = 8.8, 1.8 Hz, 1H), 7.41 (d, *J* = 8.8 Hz, 1H), 7.29 (d, *J* = 8.7 Hz, 1H) ppm.

^{19}F NMR (376 MHz, $CDCl_3$): δ = -83.16 (t, *J* = 3.9 Hz, 3F), -114.30 (s, 2F) ppm.

^{13}C NMR (100 MHz, $CDCl_3$): δ = 151.0 (t, *J* = 1.1 Hz), 146.3, 135.9 (t, *J* = 32.5 Hz), 133.3, 133.0, 130.8, 129.9, 128.7, 128.0, 127.9, 126.7, 122.4 (t, *J* = 1.8 Hz), 120.8, 120.3, 119.9, 119.3, 116.4, 110.6 ppm; carbons corresponding to the C_2F_5 group cannot be identified due to C-F coupling.

HRMS m/z: calcd for $C_{20}H_{10}ClF_5N_3O$ $[M+H]^+$ 438.0433, found: 438.0432.

Isomer II: White solid. M.p. 118.9–120.0 °C.

IR (KBr): ν = 3069, 1633, 1532, 811, 742 cm^{-1}.

^{1}H NMR (400 MHz, $CDCl_3$): δ = 8.47 – 8.42 (m, 1H), 8.15 (d, *J* = 9.3 Hz, 1H), 8.00 (d, *J* = 8.1 Hz, 1H), 7.80 (d, *J* = 8.6 Hz, 1H), 7.78 – 7.72 (m, 1H), 7.71 – 7.65 (m, 1H), 7.49 – 7.45 (m, 2H), 7.29 (d, *J* = 8.7 Hz, 1H) ppm.

^{19}F NMR (376 MHz, $CDCl_3$): δ = -83.12 – -83.25 (m, 3F), -114.34 (s, 2F) ppm.

^{13}C NMR (100 MHz, $CDCl_3$): δ = 151.0 (t, *J* = 1.0 Hz), 144.2, 136.0 (t, *J* = 32.5 Hz), 135.7, 134.9, 133.2, 128.7, 127.9, 127.8, 126.7, 126.0, 122.3 (t, *J* = 2.1 Hz), 121.4, 120.8, 120.3, 119.3, 116.3, 109.5 ppm; carbons corresponding to the C_2F_5 group cannot be identified due to C-F coupling.

HRMS m/z: calcd for $C_{20}H_{10}ClF_5N_3O$ $[M+H]^+$ 438.0427, found: 438.0430.

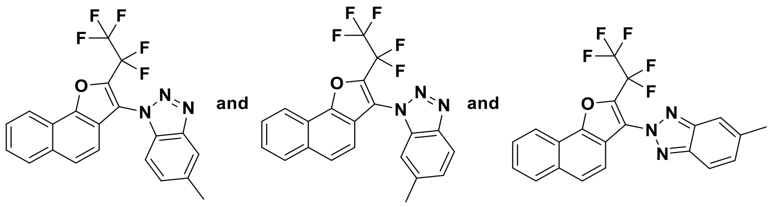

5-Methyl-1-(2-(perfluoroethyl)naphtho[1,2-*b*]furan-3-yl)-1*H*-benzo[*d*][1,2,3]triazole (7c-I), 6-methyl-1-(2-(perfluoroethyl)naphtho[1,2-*b*]furan-3-yl)-1*H*-benzo[*d*][1,2,3]triazole (7c-II), and 5-methyl-2-(2-(perfluoroethyl)naphtho[1,2-*b*]furan-3-yl)-2*H*-benzo[*d*][1,2,3]triazole (7c-III):

7c-I, 7c-II and its isomer **7c-II** were inseparable and unidentified regioisomers.

Total yield = 87% (109 mg, **7c-I**/**7c-II**/**7c-III** = 1/1/1), 0.3 mmol scale. Yellow solid.

IR (KBr): ν = 2962, 1632, 1496, 809, 722 cm^{-1}.

^{1}H NMR (400 MHz, $CDCl_3$): δ = 8.27 (d, J = 8.1 Hz, 1H), 7.97 – 7.79 (m, 1.5H), 7.64 – 7.54 (m, 2H), 7.50 (t, J = 7.5 Hz, 1H), 7.35 – 7.09 (m, 3.5H), 2.81 – 2.34 (m, 3H) ppm.

^{19}F NMR (376 MHz, $CDCl_3$): δ = -83.11 – -83.19 (m, 3F), -114.18 (d, J = 25.9 Hz, 2F) ppm.

^{13}C NMR (100 MHz, $CDCl_3$): δ = 150.8, 146.2, 145.5, 144.2, 139.9, 135.8 (t, J = 32.1 Hz), 135.7 (t, J = 32.1 Hz), 135.6 (t, J = 32.1 Hz), 134.9, 134.8, 134.2, 133.1, 132.8, 131.5, 131.0, 128.8, 128.6, 127.7, 127.6, 126.9, 126.3, 126.3, 126.3, 126.0, 124.6, 123.0 (t, J = 1.4 Hz), 123.0 (t, J = 1.4 Hz), 122.9 (t, J = 1.4 Hz), 122.6, 120.8, 120.8, 120.1, 119.8, 119.8, 119.5, 119.5, 119.5, 119.3, 116.6, 109.1, 108.8, 106.8, 21.9, 21.3, 16.6 ppm; carbons corresponding to the C_2F_5 group cannot be identified due to C-F coupling.

HRMS m/z: calcd for $C_{21}H_{13}F_5N_3O$ $[M+H]^+$ 418.0973, found: 418.0991.

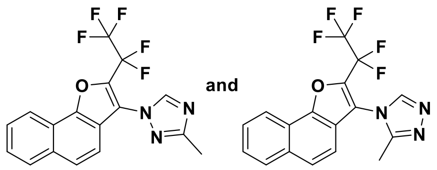

3-Methyl-1-(2-(perfluoroethyl)naphtho[1,2-*b*]furan-3-yl)-1*H*-1,2,4-triazole (8-I) and 3-methyl-4-(2-(perfluoroethyl)naphtho[1,2-*b*]furan-3-yl)-4*H*-1,2,4-triazole (8-II):

8-I and its isomer **8-II** were separable and identified regioisomers.

Total yield = 91% (100 mg, **8-I**/**8-II** = 2.8/1), 0.3 mmol scale.

Isomer 8-I: White solid. M.p. 143.0–144.3 °C.

IR (KBr): ν = 3119, 1653, 1635, 811, 729 cm^{-1}.

^{1}H NMR (400 MHz, $CDCl_3$): δ = 8.36 (d, J = 9.4 Hz, 2H), 7.98 (d, J = 8.0 Hz, 1H), 7.79 (d, J = 8.8 Hz, 1H), 7.73 – 7.67 (m, 2H), 7.64 (t, J = 7.5 Hz, 1H), 2.58 (s, 3H) ppm.

^{19}F NMR (376 MHz, $CDCl_3$): δ = -83.39 (t, J = 4.2 Hz, 3F), -112.68 – -113.49 (m, 2F) ppm.

^{13}C NMR (100 MHz, $CDCl_3$): δ = 163.0, 150.7, 145.5 (t, J = 3.6 Hz), 133.2, 132.6 (t, J = 32.1 Hz), 128.5, 127.6, 127.6, 126.1, 124.2 (m), 120.6, 120.2, 118.9, 117.6, 13.9 ppm; carbons corresponding to the C_2F_5 group cannot be identified due to C-F coupling.

HRMS m/z: calcd for $C_{17}H_{11}F_5N_3O$ $[M+H]^+$ 368.0823, found: 368.0830.

Isomer 8-II: White solid. M.p. 144.2–145.5 °C.

IR (KBr): ν = 3057, 1750, 1528, 803, 748 cm^{-1}.

^{1}H NMR (400 MHz, $CDCl_3$): *δ* = 8.45 – 8.38 (m, 1H), 8.10 (s, 1H), 8.01 (d, *J* = 8.1 Hz, 1H), 7.82 (d, *J* = 8.7 Hz, 1H), 7.77 – 7.71 (m, 1H), 7.71 – 7.65 (m, 1H), 7.34 (d, *J* = 8.7 Hz, 1H), 2.45 (s, 3H) ppm.

^{19}F NMR (376 MHz, $CDCl_3$): *δ* = -83.24 (t, *J* = 3.9 Hz, 3F), -112.81 – -117.59 (m, 2F) ppm.

^{13}C NMR (100 MHz, $CDCl_3$): *δ* = 155.0 (m), 152.5, 150.8 (t, *J* = 1.2 Hz), 136.3 (t, *J* = 31.6 Hz), 133.2, 128.7, 127.9, 127.7, 126.6, 123.8 (t, *J* = 2.2 Hz), 120.8, 120.3, 119.7, 116.2, 12.0 ppm; carbons corresponding to the C_2F_5 group cannot be identified due to C-F coupling.

HRMS m/z: calcd for $C_{17}H_{11}F_5N_3O$ $[M+H]^+$ 368.0817, found: 368.0833.

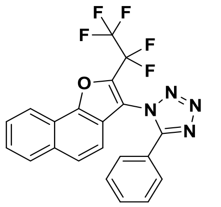

1-(2-(Perfluoroethyl)naphtho[1,2-*b*]furan-3-yl)-5-phenyl-1*H*-tetrazole (9):

Yield = 39% (50 mg), 0.3 mmol scale. Yellow solid. M.p. 131.7–132.5 °C.

IR (KBr): *ν* = 3423, 1620, 1536, 819, 728 cm^{-1}.

^{1}H NMR (400 MHz, $CDCl_3$): *δ* = 8.41 – 8.36 (m, 1H), 8.32 – 8.26 (m, 2H), 7.99 (d, *J* = 7.3 Hz, 1H), 7.96 – 7.92 (m, 1H), 7.84 (d, *J* = 8.7 Hz, 1H), 7.74 – 7.68 (m, 1H), 7.68 – 7.63 (m, 1H), 7.59 – 7.52 (m, 3H) ppm.

^{19}F NMR (376 MHz, $CDCl_3$): *δ* = -82.78 (t, *J* = 4.1 Hz, 3F), -112.60 – -112.98 (m, 2F) ppm.

^{13}C NMR (100 MHz, $CDCl_3$): *δ* = 165.6, 150.5 (t, *J* = 1.4 Hz), 133.5 (t, *J* = 33.4 Hz), 133.2, 131.0, 129.1, 128.6, 127.8, 127.8, 127.2, 126.6, 126.5, 123.7 (m), 120.5, 120.3, 117.8, 117.3 (t, *J* = 1.0 Hz) ppm; carbons corresponding to the C_2F_5 group cannot be identified due to C-F coupling.

HRMS m/z: calcd for $C_{21}H_{12}F_5N_4O$ $[M+H]^+$ 431.0926, found: 431.0921.

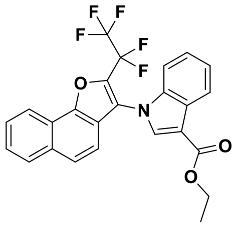

Ethyl 1-(2-(perfluoroethyl)naphtho[1,2-*b*]furan-3-yl)-1*H*-indole-3-carboxylate (10a):

Yield = 63% (89 mg), 0.3 mmol scale. Yellow solid. M.p. 134.0–135.2 °C.

IR (KBr): ν = 3066, 1626, 1540, 808, 751 cm^{-1}.

^{1}H NMR (400 MHz, $CDCl_3$): δ = 8.44 (d, J = 7.9 Hz, 1H), 8.32 (d, J = 7.6 Hz, 1H), 7.98 (d, J = 10.9 Hz, 2H), 7.73 (t, J = 8.1 Hz, 2H), 7.69 – 7.62 (m, 1H), 7.41 – 7.34 (m, 1H), 7.30 – 7.25 (m, 1H), 7.25 – 7.22 (m, 1H), 7.19 – 7.14 (m, 1H), 4.52 – 4.39 (m, 2H), 1.46 (t, J = 7.1 Hz, 3H) ppm.

^{19}F NMR (376 MHz, $CDCl_3$): δ = -83.14 – -83.73 (m, 3F), -112.59 – -115.84 (m, 2F) ppm.

^{13}C NMR (100 MHz, $CDCl_3$): δ = 164.6, 150.8 (t, J = 1.3 Hz), 137.9, 135.7 (t, J = 31.8 Hz), 135.0, 133.2, 128.6, 127.7, 127.6, 126.4, 126.0, 124.9 (t, J = 1.8 Hz), 123.9, 122.9, 122.0, 121.0, 120.3, 120.2, 116.9, 110.9, 110.8, 60.1, 14.5 ppm; carbons corresponding to the C_2F_5 group cannot be identified due to C-F coupling.

HRMS m/z: calcd for $C_{25}H_{17}F_5NO_3$ [M+H]$^+$ 474.1123, found: 474.1126.

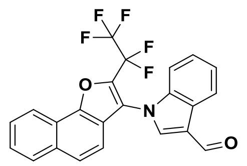

1-(2-(Perfluoroethyl)naphtho[1,2-*b*]furan-3-yl)-1*H*-indole-3-carbaldehyde (10b):

Yield = 57% (74 mg), 0.3 mmol scale. Light brown solid. M.p. 150.5–152.2 °C.

IR (KBr): ν = 3104, 1676, 1626, 1611, 805, 755 cm^{-1}.

^{1}H NMR (400 MHz, $CDCl_3$): δ = 10.16 (s, 1H), 8.48 – 8.42 (m, 2H), 8.00 (d, J = 8.1 Hz, 1H), 7.90 (s, 1H), 7.79 – 7.72 (m, 2H), 7.71 – 7.65 (m, 1H), 7.44 – 7.38 (m, 1H), 7.36 – 7.30 (m, 1H), 7.26 (d, J = 8.7 Hz, 1H), 7.18 (d, J = 8.2 Hz, 1H) ppm.

^{19}F NMR (376 MHz, $CDCl_3$): δ = -83.45 (t, J = 4.0 Hz, 3F), -112.29 – -115.94 (m, 2F) ppm.

^{13}C NMR (100 MHz, $CDCl_3$): δ = 185.0, 150.9 (t, J = 1.0 Hz), 139.2, 138.4, 135.7 (t, J = 31.7 Hz), 133.2, 128.6, 127.9, 127.7, 126.2, 125.1, 125.0, 124.6 (t, J = 1.6 Hz), 123.8, 122.4, 121.0, 120.7, 120.3, 119.9, 116.7, 110.8 ppm; carbons corresponding to the C_2F_5 group cannot be identified due to C-F coupling.

HRMS m/z: calcd for $C_{23}H_{13}F_5NO_2$ [M+H]$^+$ 430.0861, found: 430.0871.

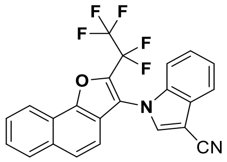

1-(2-(Perfluoroethyl)naphtho[1,2-*b*]furan-3-yl)-1*H*-indole-3-carbonitrile (10c):

Yield = 65% (83 mg), 0.3 mmol scale. White solid. M.p. 169.8–171.3 °C.

IR (KBr): ν = 3118, 2228, 1748, 1471, 804, 746 cm^{-1}.

^{1}H NMR (400 MHz, $CDCl_3$): δ = 8.46 (d, *J* = 8.2 Hz, 1H), 8.01 (d, *J* = 8.1 Hz, 1H), 7.92 – 7.86 (m, 1H), 7.80 – 7.73 (m, 3H), 7.72 – 7.66 (m, 1H), 7.45 – 7.39 (m, 1H), 7.38 – 7.33 (m, 1H), 7.26 – 7.19 (m, 2H) ppm.

^{19}F NMR (376 MHz, $CDCl_3$): δ = -83.01 – -83.86 (m, 3F), -112.07 – -116.33 (m, 2F) ppm.

^{13}C NMR (100 MHz, $CDCl_3$): δ = 150.9 (t, *J* = 1.1 Hz), 136.6, 135.9 (t, *J* = 31.9 Hz), 135.7, 133.2, 128.7, 127.9, 127.8, 127.3, 126.3, 125.2, 124.2 (t, *J* = 1.3 Hz), 123.3, 120.9, 120.3, 120.1, 119.8, 116.5, 114.7, 111.4, 89.8 ppm; carbons corresponding to the C_2F_5 group cannot be identified due to C-F coupling.

HRMS m/z: calcd for $C_{23}H_{12}F_5N_2O$ $[M+H]^+$ 427.0864, found: 427.0870.

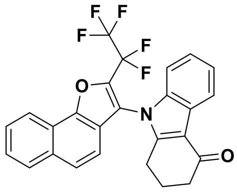

9-(2-(Perfluoroethyl)naphtho[1,2-*b*]furan-3-yl)-1,2,3,9-tetrahydro-4*H*-carbazol-4-one (10d):

Yield = 76% (107 mg), 0.3 mmol scale. Light brown solid. M.p. 150.8–151.3 °C.

IR (KBr): ν = 3116, 1721, 1659, 1617, 810, 749 cm^{-1}.

^{1}H NMR (400 MHz, $CDCl_3$): δ = 8.50 – 8.45 (m, 1H), 8.39 – 8.34 (m, 1H), 8.00 (d, *J* = 7.5 Hz, 1H), 7.80 – 7.73 (m, 2H), 7.71 – 7.65 (m, 1H), 7.38 – 7.31 (m, 1H), 7.24 – 7.19 (m, 1H), 7.17 (d, *J* = 8.7 Hz, 1H), 7.01 (d, *J* = 8.2 Hz, 1H), 2.92 – 2.80 (m, 1H), 2.76 – 2.63 (m, 3H), 2.30 – 2.18 (m, 2H) ppm.

^{19}F NMR (376 MHz, $CDCl_3$): δ = -83.01 – -83.57 (m, 3F), -113.30 – -117.05 (m, 2F) ppm.

^{13}C NMR (100 MHz, $CDCl_3$): δ = 194.3, 152.6, 151.2 (t, *J* = 1.2 Hz), 138.4, 137.2 (t, *J* = 31.4 Hz), 133.2, 128.6, 127.9, 127.7, 126.4, 124.8, 123.9, 123.4, 122.7 (t, *J* = 1.6 Hz), 121.7, 121.0,

120.4, 120.2, 116.3, 114.8, 110.0, 38.0, 23.4, 22.2 ppm; carbons corresponding to the C_2F_5 group cannot be identified due to C-F coupling.

HRMS m/z: calcd for $C_{26}H_{17}F_5NO_2$ $[M+H]^+$ 470.1174, found: 470.1184.

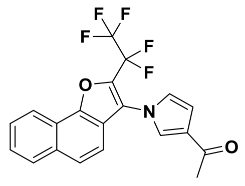

1-(1-(2-(Perfluoroethyl)naphtho[1,2-*b*]furan-3-yl)-1*H*-pyrrol-3-yl)ethan-1-one (11a):

Yield = 39% (46 mg), 0.3 mmol scale. White solid. M.p. 98.1–98.7 °C.

IR (KBr): ν = 3141, 1667, 1629, 810, 759 cm^{-1}.

^{1}H NMR (400 MHz, $CDCl_3$): δ = 8.40 (d, J = 8.1 Hz, 1H), 8.00 (d, J = 8.0 Hz, 1H), 7.81 (d, J = 8.7 Hz, 1H), 7.75 – 7.69 (m, 1H), 7.69 – 7.63 (m, 1H), 7.58 (t, J = 1.8 Hz, 1H), 7.51 (d, J = 8.7 Hz, 1H), 7.00 – 6.96 (m, 1H), 6.88 – 6.84 (m, 1H), 2.50 (s, 3H) ppm.

^{19}F NMR (376 MHz, $CDCl_3$): δ = -83.48 (t, J = 4.0 Hz, 3F), -113.36 (q, J = 3.7 Hz, 2F) ppm.

^{13}C NMR (100 MHz, $CDCl_3$): δ = 193.3, 150.5 (t, J = 1.0 Hz), 133.5 (t, J = 31.9 Hz), 133.2, 128.6, 127.9, 127.8, 127.6, 127.3 (t, J = 1.5 Hz), 127.0 (t, J = 1.9 Hz), 126.2, 124.5, 120.8, 120.3, 119.7, 116.4, 110.6, 27.3 ppm; carbons corresponding to the C_2F_5 group cannot be identified due to C-F coupling.

HRMS m/z: calcd for $C_{20}H_{13}F_5NO_2$ $[M+H]^+$ 394.0861, found: 394.0869.

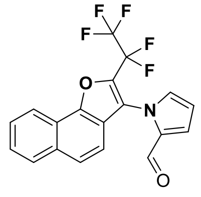

1-(2-(Perfluoroethyl)naphtho[1,2-*b*]furan-3-yl)-1*H*-pyrrole-2-carbaldehyde (11b):

Yield = 66% (75 mg), 0.3 mmol scale. Yellow solid. M.p. 119.0–119.4 °C.

IR (KBr): ν = 3137, 1672, 1537, 815, 768 cm^{-1}.

^{1}H NMR (400 MHz, $CDCl_3$): δ = 9.61 – 9.59 (m, 1H), 8.41 – 8.35 (m, 1H), 7.94 (d, J = 8.1 Hz, 1H), 7.72 (d, J = 8.6 Hz, 1H), 7.70 – 7.65 (m, 1H), 7.63 – 7.57 (m, 1H), 7.25 (d, J = 8.6 Hz, 1H), 7.23 – 7.20 (m, 1H), 7.09 (s, 1H), 6.53 (dd, J = 4.0, 2.7 Hz, 1H) ppm.

^{19}F NMR (376 MHz, $CDCl_3$): δ = -83.62 (t, J = 3.9 Hz, 3F), -112.24 – -117.23 (m, 2F) ppm.

^{13}C NMR (100 MHz, $CDCl_3$): δ = 178.2, 150.2 (t, J = 1.2 Hz), 135.0 (t, J = 31.2 Hz), 133.6, 133.1, 132.2, 128.5, 127.5, 127.2, 126.5 (t, J = 2.3 Hz), 125.9, 123.7 (m), 121.1, 121.0, 120.3, 116.6, 111.6 ppm; carbons corresponding to the C_2F_5 group cannot be identified due to C-F coupling.

HRMS m/z: calcd for $C_{19}H_{11}F_5NO_2$ $[M+H]^+$ 380.0704, found: 380.0704.

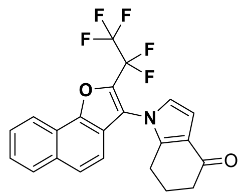

1-(2-(Perfluoroethyl)naphtho[1,2-*b*]furan-3-yl)-1,5,6,7-tetrahydro-4*H*-indol-4-one (11c):

Yield = 46% (78 mg), 0.3 mmol scale. Yellow solid. M.p. 113.4–114.9 ^{o}C.

IR (KBr): ν = 2951, 1668, 1627, 809, 748 cm^{-1}.

^{1}H NMR (400 MHz, $CDCl_3$): δ = 8.46 – 8.38 (m, 1H), 8.01 (d, J = 8.1 Hz, 1H), 7.82 (d, J = 8.7 Hz, 1H), 7.74 (t, J = 7.5 Hz, 1H), 7.68 (t, J = 7.6 Hz, 1H), 7.35 (d, J = 8.7 Hz, 1H), 6.80 (d, J = 4.2 Hz, 2H), 2.61 (t, J = 6.3 Hz, 2H), 2.59 – 2.52 (m, 2H), 2.15 (p, J = 6.3 Hz, 2H) ppm.

^{19}F NMR (376 MHz, $CDCl_3$): δ = -83.53 (t, J = 3.2 Hz, 3F), -112.29 – -117.12 (m, 2F) ppm.

^{13}C NMR (100 MHz, $CDCl_3$): δ = 194.3, 150.6 (t, J = 1.3 Hz), 145.3, 135.8 (t, J = 31.5 Hz), 133.2, 128.6, 127.8, 127.6, 126.4, 125.1 (t, J = 1.8 Hz), 124.5, 122.1, 120.9, 120.4, 120.2, 116.2, 107.0, 37.7, 23.7, 21.8 ppm; carbons corresponding to the C_2F_5 group cannot be identified due to C-F coupling.

HRMS m/z: calcd for $C_{22}H_{15}F_5NO_2$ $[M+H]^+$ 420.1017, found: 420.1018.

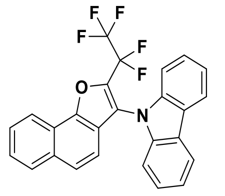

9-(2-(Perfluoroethyl)naphtho[1,2-*b*]furan-3-yl)-9*H*-carbazole (12):

Yield = 40% (54 mg), 0.3 mmol scale. Yellow solid. M.p. 111.8–112.3 ^{o}C.

IR (KBr): ν = 3363, 1790, 1620, 812, 746 cm^{-1}.

^{1}H NMR (400 MHz, $CDCl_3$): δ = 8.53 – 8.43 (m, 1H), 8.20 – 8.16 (m, 2H), 7.91 (d, J = 8.2 Hz,

1H), 7.70 (t, *J* = 7.5 Hz, 1H), 7.59 (d, *J* = 6.9 Hz, 2H), 7.42 – 7.36 (m, 2H), 7.35 – 7.30 (m, 2H), 7.19 – 7.16 (m, 2H), 7.01 (s, 1H) ppm.

^{19}F NMR (376 MHz, $CDCl_3$): δ = -82.76 – -83.72 (m, 3F), -114.60 – -115.57 (m, 2F) ppm.

^{13}C NMR (100 MHz, $CDCl_3$): δ = 151.4, 141.4, 137.5 (t, *J* = 31.6 Hz), 133.1, 128.6, 127.5, 127.3, 126.3, 125.5, 123.9, 123.8 (t, *J* = 1.8 Hz), 121.2, 120.7, 120.7, 120.4, 120.2, 117.4, 110.0 ppm; carbons corresponding to the C_2F_5 group cannot be identified due to C-F coupling.

HRMS m/z: calcd for $C_{26}H_{15}F_5NO$ $[M+H]^+$ 452.1068, found: 452.1070.

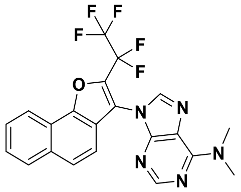

***N*,*N*-Dimethyl-9-(2-(perfluoroethyl)naphtho[1,2-*b*]furan-3-yl)-9*H*-purin-6-amine (13):**

Yield = 36% (48 mg), 0.3 mmol scale. Yellow solid. M.p. 175.2–175.6 °C.

IR (KBr): ν = 2934, 1614, 1566, 820, 745 cm^{-1}.

^{1}H NMR (400 MHz, $CDCl_3$): δ = 8.40 (d, *J* = 8.2 Hz, 1H), 8.38 – 8.35 (m, 1H), 7.96 (d, *J* = 8.1 Hz, 1H), 7.92 – 7.88 (m, 1H), 7.76 (d, *J* = 8.7 Hz, 1H), 7.70 (t, *J* = 7.6 Hz, 1H), 7.66 – 7.60 (m, 1H), 7.36 (d, *J* = 8.7 Hz, 1H), 3.60 (s, 6H) ppm.

^{19}F NMR (376 MHz, $CDCl_3$): δ = -83.33 – -83.61 (m, 3F), -108.83 – -109.25 (m, 2F) ppm.

^{13}C NMR (100 MHz, $CDCl_3$): δ = 155.1, 153.5, 151.6, 151.0 (t, *J* = 1.1 Hz), 138.0, 135.3 (t, *J* = 31.2 Hz), 133.2, 128.5, 127.6, 127.5, 126.1, 121.5 (t, *J* = 1.3 Hz), 120.9, 120.2, 119.9, 119.6, 117.2, 116.9, 38.6 ppm; carbons corresponding to the C_2F_5 group cannot be identified due to C-F coupling.

HRMS m/z: calcd for $C_{21}H_{15}F_5N_5O$ $[M+H]^+$ 448.1191, found: 448.1196.

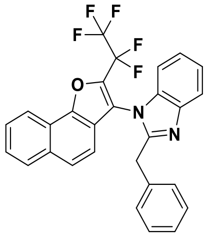

2-Benzyl-1-(2-(perfluoroethyl)naphtho[1,2-*b*]furan-3-yl)-1*H*-benzo[*d*]imidazole (15a):

Yield = 38% (56 mg), 0.3 mmol scale. Yellow oil.

IR (KBr): ν = 2993, 1620, 1492, 812, 746 cm^{-1}.

^{1}H NMR (400 MHz, $CDCl_3$): δ = 8.46 – 8.40 (m, 1H), 7.94 (d, J = 8.1 Hz, 1H), 7.90 – 7.86 (m, 1H), 7.76 – 7.70 (m, 1H), 7.68 – 7.62(m, 1H), 7.54 (d, J = 8.6 Hz, 1H), 7.36 – 7.30 (m, 1H), 7.23 – 7.18 (m, 1H), 7.03 – 6.91 (m, 6H), 6.78 (d, J = 8.7 Hz, 1H), 4.43 – 4.02 (m, 2H) ppm.

^{19}F NMR (376 MHz, $CDCl_3$): δ = -82.68 – -83.46 (m, 3F), -112.29 – -118.35 (m, 2F) ppm.

^{13}C NMR (100 MHz, $CDCl_3$): δ = 154.0, 150.9 (t, J = 1.1 Hz), 142.7, 136.8 (m), 136.7, 135.2, 133.1, 128.6, 128.6, 128.4, 127.6, 127.6, 126.7, 125.9, 123.5, 123.0, 122.4 (d, J = 2.6 Hz), 120.8, 120.2 (d, J = 0.7 Hz), 120.1, 119.8, 116.2, 109.6, 34.5 ppm; carbons corresponding to the C_2F_5 group cannot be identified due to C-F coupling.

HRMS m/z: calcd for $C_{28}H_{18}F_5N_2O$ $[M+H]^+$ 493.1334, found: 493.1336.

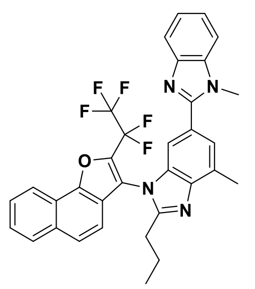

1,7'-Dimethyl-3'-(2-(perfluoroethyl)naphtho[1,2-*b*]furan-3-yl)-2'-propyl-1*H*,3'*H*-2,5'-bibenzo[*d*]imidazole (15b):

Yield = 88% (156 mg), 0.3 mmol scale. Yellow solid. M.p. 121.3–121.7 °C.

IR (KBr): ν = 2967, 1624, 1529, 814, 745 cm^{-1}.

^{1}H NMR (400 MHz, $CDCl_3$): δ = 8.51 – 8.38 (m, 1H), 8.02 – 7.91 (m, 1H), 7.81 – 7.68 (m, 3H), 7.64 (t, J = 7.6 Hz, 1H), 7.57 (s, 1H), 7.32 – 7.26 (m, 2H), 7.25 – 7.18 (m, 3H), 3.77 (s, 3H), 2.91 – 2.77 (m, 5H), 1.95 – 1.79 (m, 2H), 0.95 (t, J = 7.4 Hz, 3H) ppm.

^{19}F NMR (376 MHz, $CDCl_3$): δ = -83.01 (s, 3F), -114.62 – -114.84 (m, 2F) ppm.

^{13}C NMR (100 MHz, $CDCl_3$): δ = 156.5, 154.1, 151.2 (t, J = 1.1 Hz), 143.2, 142.7, 136.8 (t, J = 31.8 Hz), 136.5, 136.1, 133.2, 129.9, 128.6, 127.8, 127.6, 126.5, 124.9, 122.4, 122.3 (t, J = 1.6 Hz), 122.1, 120.9, 120.2, 120.0, 119.4, 116.0, 109.3, 108.5, 31.5, 29.5, 21.1, 16.7, 13.7 ppm; carbons corresponding to the C_2F_5 group cannot be identified due to C-F coupling.

HRMS m/z: calcd for $C_{33}H_{26}F_5N_4O$ $[M+H]^+$ 589.2021, found: 589.2028.

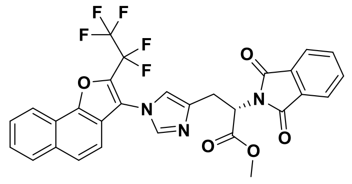

Methyl (*S*)-2-(1,3-dioxoisoindolin-2-yl)-3-(1-(2-(perfluoroethyl)naphtho[1,2-*b*]furan-3-yl)-1*H*-imidazol-4-yl)propanoate (15c):

Yield = 35% (61 mg), 0.3 mmol scale. Yellow oil.

IR (KBr): ν = 2956, 1717, 1631, 814, 718 cm^{-1}.

^{1}H NMR (400 MHz, $CDCl_3$): δ = 8.33 (d, *J* = 8.1 Hz, 1H), 7.96 (d, *J* = 7.8 Hz, 1H), 7.87 – 7.81 (m, 2H), 7.75 – 7.69 (m, 3H), 7.69 – 7.62 (m, 2H), 7.59 (s, 1H), 7.22 (d, *J* = 8.7 Hz, 1H), 6.96 (s, 1H), 5.42 – 5.35 (m, 1H), 3.81 (s, 3H), 3.73 – 3.59 (m, 2H) ppm.

^{19}F NMR (376 MHz, $CDCl_3$): δ = -83.77 (t, *J* = 4.0 Hz, 3F), -113.50 – -114.18 (m, 2F) ppm.

^{13}C NMR (100 MHz, $CDCl_3$): δ = 169.3, 167.3, 150.4 (t, *J* = 1.2 Hz), 139.0, 137.9, 134.0, 133.7 (t, *J* = 31.9 Hz), 133.1, 131.7, 128.5, 127.7, 127.6, 126.2, 124.0 (t, *J* = 2.0 Hz), 123.4, 120.7, 120.2, 119.5, 118.5, 116.0, 52.8, 51.9, 27.6 ppm; carbons corresponding to the C_2F_5 group cannot be identified due to C-F coupling.

HRMS m/z: calcd for $C_{29}H_{19}F_5N_3O_5$ $[M+H]^+$ 584.1239, found: 584.1245.

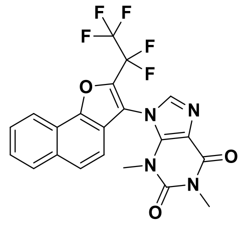

1,3-Dimethyl-9-(2-(perfluoroethyl)naphtho[1,2-*b*]furan-3-yl)-3,9-dihydro-1*H*-purine-2,6-dione (15d):

Yield = 49% (68 mg), 0.3 mmol scale. Yellow solid. M.p. 236.5–237.1 ^{o}C.

IR (KBr): ν = 3125, 1712, 1676, 818, 748 cm^{-1}.

^{1}H NMR (400 MHz, $CDCl_3$): δ = 8.43 – 8.35 (m, 1H), 7.97 (d, *J* = 8.1 Hz, 1H), 7.83 – 7.76 (m, 2H), 7.74 – 7.68 (m, 1H), 7.67 – 7.61 (m, 1H), 7.34 (d, *J* = 8.7 Hz, 1H), 3.70 (s, 3H), 3.36 (s, 3H) ppm.

^{19}**F NMR** (376 MHz, $CDCl_3$): δ = -83.53 (t, J = 4.1 Hz, 3F), -111.87 – -117.02 (m, 2F) ppm.

^{13}**C NMR** (100 MHz, $CDCl_3$): δ = 153.8, 151.6, 150.6 (t, J = 1.2 Hz), 149.2, 142.4, 135.5 (t, J = 31.1 Hz), 133.2, 128.5, 127.7, 127.6, 126.4, 122.0 (t, J = 1.2 Hz), 120.8, 120.2, 120.2, 116.2, 108.4, 30.0, 28.1 ppm; carbons corresponding to the C_2F_5 group cannot be identified due to C-F coupling.

HRMS m/z: calcd for $C_{21}H_{14}F_5N_4O_3$ $[M+H]^+$ 465.0981, found: 465.0986.

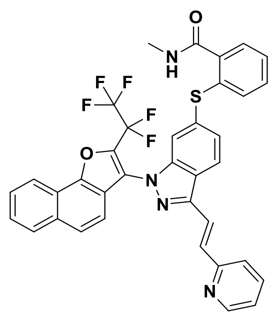

(*E*)-*N*-Methyl-2-((1-(2-(perfluoroethyl)naphtho[1,2-*b*]furan-3-yl)-3-(2-(pyridin-2-yl)vinyl)-1*H*-indazol-6-yl)thio)benzamide (15e):

Yield = 80% (54 mg), 0.1 mmol scale. Yellow solid. M.p. 113.1–113.5 °C.

IR (KBr): ν = 3072, 1676, 1623, 802, 760 cm^{-1}.

^{1}**H NMR** (400 MHz, $CDCl_3$): δ = 8.65 (d, J = 4.4 Hz, 1H), 8.41 (d, J = 8.1 Hz, 1H), 8.08 (d, J = 8.4 Hz, 1H), 8.04 – 7.93 (m, 2H), 7.77 – 7.63 (m, 5H), 7.57 (d, J = 7.3 Hz, 1H), 7.48 (d, J = 7.8 Hz, 1H), 7.44 – 7.39 (m, 1H), 7.37 (s, 1H), 7.34 – 7.30 (m, 1H), 7.28 – 7.18 (m, 4H), 6.31 (s, 1H), 2.91 (dd, J = 4.8, 1.1 Hz, 3H) ppm.

^{19}**F NMR** (376 MHz, $CDCl_3$): δ = -82.92 (t, J = 3.2 Hz, 3F), -111.73 – -118.12 (m, 2F) ppm.

^{13}**C NMR** (100 MHz, $CDCl_3$): δ = 168.3, 155.0, 150.7 (t, J = 1.4 Hz), 149.8, 145.4, 142.9, 136.9, 136.7, 135.8, 135.1 (t, J = 33.1 Hz), 133.9, 133.1, 132.2, 131.9, 130.8, 128.7, 128.6, 127.5, 127.4, 127.3, 125.9, 125.9, 125.5 (t, J = 1.5 Hz), 122.6, 122.4, 122.4, 122.3, 121.8, 120.9, 120.2, 119.9, 117.4, 112.9, 26.6 ppm; carbons corresponding to the C_2F_5 group cannot be identified due to C-F coupling.

HRMS m/z: calcd for $C_{36}H_{24}F_5N_4O_2S$ $[M+H]^+$ 671.1535, found: 671.1539.

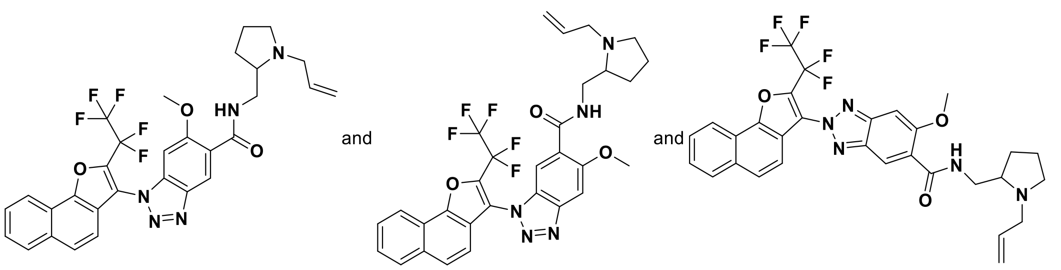

***N*-((1-allylpyrrolidin-2-yl)methyl)-6-methoxy-1-(2-(perfluoroethyl)naphtho[1,2-*b*]furan-3-yl)-1*H*-benzo[*d*][1,2,3]triazole-5-carboxamide (15f-I), *N*-((1-allylpyrrolidin-2-yl)methyl)-5-methoxy-1-(2-(perfluoroethyl)naphtho[1,2-*b*]furan-3-yl)-1*H*-benzo[*d*][1,2,3]triazole-6-carboxamide (15f-II), and *N*-((1-allylpyrrolidin-2-yl)methyl)-6-methoxy-2-(2-(perfluoroethyl)naphtho[1,2-*b*]furan-3-yl)-2*H*-benzo[*d*][1,2,3]triazole-5-carboxamide (15f-III):**

15f-I, **15f-II** and its isomer **15f-III** were inseparable and unidentified regioisomers.

Total yield = 58% (35 mg, **15f-I/15f-II/15f-III** = 10/6/1), 0.1 mmol scale. Yellow solid.

IR (KBr): ν = 2967, 1659, 1525, 807, 757 cm^{-1}.

^{1}H NMR (400 MHz, $CDCl_3$): *δ* = 9.08 – 8.30 (m, 3H), 8.01 (t, *J* = 8.3 Hz, 1H), 7.90 – 7.64 (m, 3H), 7.38 – 7.28 (m, 0.6H), 6.84 (s, 0.4H), 6.08 – 5.75 (m, 1H), 5.40 – 5.00 (m, 2H), 4.20 – 3.89 (m, 3H), 3.86 – 3.69 (m, 1H), 3.56 – 3.33 (m, 2H), 3.26 – 3.13 (m, 1H), 3.04 – 2.71 (m, 2H), 2.43 – 2.08 (m, 2H), 2.01 – 1.89 (m, 1H), 1.82 – 1.60 (m, 3H) ppm.

^{19}F NMR (376 MHz, $CDCl_3$): *δ* = -82.49 – -83.30 (m, 3F), -111.54 – -114.87 (m, 2F) ppm.

^{13}C NMR (100 MHz, $CDCl_3$): *δ* = 164.6, 164.2, 158.9, 155.5, 151.0 (t, *J* = 1.4 Hz), 150.9 (t, *J* = 1.4 Hz), 147.2, 140.4, 136.3 (m), 135.7 (m), 133.2, 129.5, 128.6, 127.9, 127.8, 127.7, 126.7, 126.6, 125.6, 125.1, 122.5 (t, *J* = 1.5 Hz), 122.4 (t, *J* = 1.5 Hz), 120.9, 120.8, 120.3, 120.3, 119.5, 119.4, 116.6, 116.3, 113.9, 100.0, 90.1, 62.1 (m), 61.7 (m), 57.1 (m), 57.0 (m), 56.4, 56.4, 54.2, 41.4, 41.2, 28.4, 28.3, 23.0 ppm; carbons corresponding to the C_2F_5 group cannot be identified due to C-F coupling.

HRMS m/z: calcd for $C_{30}H_{27}F_5N_5O_3$ $[M+H]^+$ 600.2029, found: 600.2036.

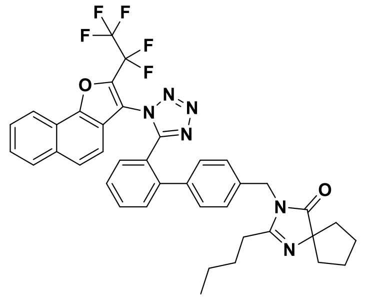

2-Butyl-3-((2'-(1-(2-(perfluoroethyl)naphtho[1,2-*b*]furan-3-yl)-1*H*-tetrazol-5-yl)-[1,1'-biphenyl]-4-yl)methyl)-1,3-diazaspiro[4.4]non-1-en-4-one (15g):

Yield = 15% (32 mg), 0.3 mmol scale. Yellow solid. M.p. 184.6–185.9 ^{o}C.

IR (KBr): ν = 2962, 1727, 1629, 808, 758 cm^{-1}.

^{1}H NMR (400 MHz, $CDCl_3$): δ = 8.32 – 8.24 (m, 1H), 8.02 (d, *J* = 7.8 Hz, 1H), 7.88 – 7.81 (m, 1H), 7.76 – 7.65 (m, 3H), 7.63 – 7.54 (m, 2H), 7.25 – 7.20 (m, 1H), 6.69 (d, *J* = 8.0 Hz, 2H), 6.64 (d, *J* = 8.8 Hz, 1H), 6.57 (d, *J* = 8.0 Hz, 2H), 4.23 (s, 2H), 2.15 – 2.08 (m, 2H), 1.97 – 1.92 (m, 5H), 1.80 – 1.71 (m, 2H), 1.52 – 1.42 (m, 2H), 1.28 – 1.22 (m, 3H), 0.80 (t, *J* = 7.3 Hz, 3H) ppm.

^{19}F NMR (376 MHz, $CDCl_3$): δ = -82.88 (t, *J* = 2.5 Hz, 3F), -106.78 – -125.10 (m, 2F) ppm.

^{13}C NMR (100 MHz, $CDCl_3$): δ = 186.5, 161.3, 156.3, 150.3 (t, *J* = 1.3 Hz), 140.8, 138.3, 136.3, 133.8 (t, *J* = 32.1 Hz), 132.9, 132.5, 132.0, 130.8, 128.7, 128.4, 128.1, 127.9, 127.8, 126.9, 125.8, 121.3, 120.5 (t, *J* = 1.3 Hz), 120.2, 120.0, 117.9, 116.6, 76.4, 60.4, 42.7, 37.3, 28.5, 27.6, 26.0, 22.2, 14.2, 13.6 ppm; carbons corresponding to the C_2F_5 group cannot be identified due to C-F coupling.

HRMS m/z: calcd for $C_{39}H_{34}F_5N_6O_2$ $[M+H]^+$ 713.2658, found: 713.2661.

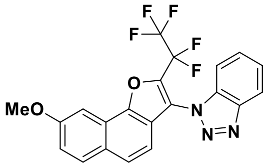

1-(8-Methoxy-2-(perfluoroethyl)naphtho[1,2-*b*]furan-3-yl)-1*H*-benzo[*d*][1,2,3]triazole (16a):

Yield = 96% (83 mg), 0.2 mmol scale. White solid. M.p. 176.8–177.5 ^{o}C.

IR (KBr): ν = 3074, 1636, 1601, 836, 753 cm^{-1}.

^{1}H NMR (400 MHz, $CDCl_3$): δ = 8.23 (d, J = 8.3 Hz, 1H), 7.87 (d, J = 9.0 Hz, 1H), 7.72 – 7.64 (m, 2H), 7.61 – 7.56 (m, 1H), 7.54 – 7.44 (m, 2H), 7.29 (dd, J = 8.9, 2.4 Hz, 1H), 7.14 (d, J = 8.6 Hz, 1H), 4.04 (s, 3H) ppm.

^{19}F NMR (376 MHz, $CDCl_3$): δ = -83.16 (t, J = 4.0 Hz, 3F), -114.37 (s, 2F) ppm.

^{13}C NMR (100 MHz, $CDCl_3$): δ = 159.2, 150.4 (t, J = 0.9 Hz), 145.6, 135.7 (t, J = 32.1 Hz), 134.2, 130.2, 128.9, 128.3, 126.0, 124.7, 122.8 (t, J = 1.4 Hz), 121.9, 120.4, 120.0, 119.9, 113.9, 109.6, 98.9, 55.6 ppm; carbons corresponding to the C_2F_5 group cannot be identified due to C-F coupling.

HRMS m/z: calcd for $C_{21}H_{13}F_5N_3O_2$ $[M+H]^+$ 434.0922, found: 434.0927.

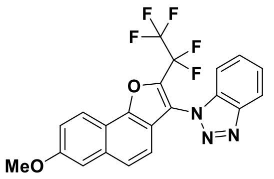

1-(7-Methoxy-2-(perfluoroethyl)naphtho[1,2-*b*]furan-3-yl)-1*H*-benzo[*d*][1,2,3]triazole (16b):

Yield = 74% (64 mg), 0.2 mmol scale. Yellow solid. M.p. 134.0–135.2 °C.

IR (KBr): ν = 3066, 1626, 1540, 808, 751 cm^{-1}.

^{1}H NMR (400 MHz, $CDCl_3$): δ = 8.34 (d, J = 9.0 Hz, 1H), 8.22 (d, J = 8.3 Hz, 1H), 7.66 (d, J = 8.7 Hz, 1H), 7.62 – 7.55 (m, 1H), 7.54 – 7.45 (m, 2H), 7.38 (dd, J = 9.0, 2.4 Hz, 1H), 7.31 (d, J = 2.3 Hz, 1H), 7.29 – 7.25 (m, 1H), 3.97 (s, 3H) ppm.

^{19}F NMR (376 MHz, $CDCl_3$): δ = -83.17 (t, J = 2.6 Hz, 3F), -114.19 (s, 2F) ppm.

^{13}C NMR (100 MHz, $CDCl_3$): δ = 159.1, 151.3 (t, J = 1.1 Hz), 145.6, 135.0, 134.9 (t, J = 32.4 Hz), 134.2, 128.9, 125.5, 124.7, 122.8 (t, J = 1.7 Hz), 121.9, 120.4, 119.8, 117.8, 117.3, 115.8, 109.7, 107.5, 55.4 ppm; carbons corresponding to the C_2F_5 group cannot be identified due to C-F coupling.

HRMS m/z: calcd for $C_{21}H_{13}F_5N_3O_2$ $[M+H]^+$ 434.0922, found: 434.0926.

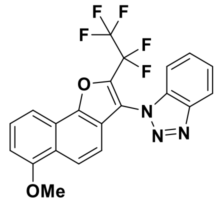

1-(6-Methoxy-2-(perfluoroethyl)naphtho[1,2-*b*]furan-3-yl)-1*H*-benzo[*d*][1,2,3]triazole (16c):

Yield = 53% (46 mg, 0.2 mmol scale). White solid. M.p. 137.0–138.9 °C.

IR (KBr): ν = 2944, 1634, 1533, 796, 721 cm^{-1}.

^{1}H NMR (400 MHz, $CDCl_3$): δ = 8.22 (dd, J = 8.5, 5.6 Hz, 2H), 8.01 (d, J = 8.3 Hz, 1H), 7.65 (t, J = 8.1 Hz, 1H), 7.62 – 7.55 (m, 1H), 7.54 – 7.44 (m, 2H), 7.28 (d, J = 8.9 Hz, 1H), 7.03 (d, J = 7.8 Hz, 1H), 4.04 (s, 3H) ppm.

^{19}F NMR (376 MHz, $CDCl_3$): δ = -82.89 – -83.30 (m, 3F), -114.30 (s, 2F) ppm.

^{13}C NMR (100 MHz, $CDCl_3$): δ = 155.9, 150.8 (t, J = 1.3 Hz), 145.6, 135.9 (t, J = 32.4 Hz), 134.3, 128.9, 128.4, 124.9, 124.7, 122.7 (t, J = 1.4 Hz), 121.8, 120.5, 120.4, 120.0, 115.6, 112.3, 109.7, 106.2, 55.7 ppm; carbons corresponding to the C_2F_5 group cannot be identified due to C-F coupling.

HRMS m/z: calcd for $C_{21}H_{13}F_5N_3O_2$ $[M+H]^+$ 434.0922, found: 434.0927.

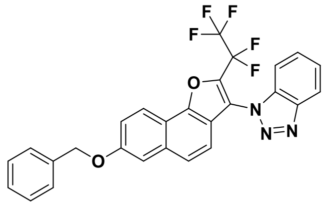

1-(7-(Benzyloxy)-2-(perfluoroethyl)naphtho[1,2-*b*]furan-3-yl)-1*H*-benzo[*d*][1,2,3]triazole (16d):

Yield = 36% (37 mg), 0.2 mmol scale. Light yellow solid. M.p. 109.7–111.1 °C.

IR (KBr): ν = 2907, 1641, 1612, 800, 746 cm^{-1}.

^{1}H NMR (400 MHz, $CDCl_3$): δ = 8.37 (d, J = 9.0 Hz, 1H), 8.23 (d, J = 8.3 Hz, 1H), 7.66 (d, J = 8.7 Hz, 1H), 7.62 – 7.55 (m, 1H), 7.54 – 7.34 (m, 9H), 7.28 (s, 1H), 5.23 (s, 2H) ppm.

^{19}F NMR (376 MHz, $CDCl_3$): δ = -83.07 – -83.52 (m, 3F), -114.32 (s, 2F) ppm.

^{13}C NMR (100 MHz, $CDCl_3$): δ = 158.2, 151.2 (t, J = 1.2 Hz), 145.6, 136.2, 135.0, 134.9, 134.2, 128.9, 128.7, 128.2, 127.6, 125.5, 124.7, 122.8 (t, J = 1.5 Hz), 122.0, 120.4, 120.2, 117.9, 117.3,

115.9, 109.7, 108.9, 70.2 ppm; carbons corresponding to the C_2F_5 group cannot be identified due to C-F coupling.

HRMS m/z: calcd for $C_{27}H_{17}F_5N_3O_2$ $[M+H]^+$ 510.1235, found: 510.1242.

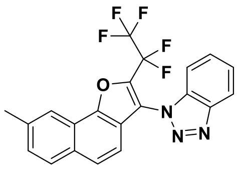

1-(8-Methyl-2-(perfluoroethyl)naphtho[1,2-*b*]furan-3-yl)-1*H*-benzo[*d*][1,2,3]triazole (16e):

Yield = 90% (75 mg), 0.2 mmol scale. Yellow solid. M.p. 150.5–151.4 °C.

IR (KBr): ν = 2920, 1630, 1615, 830, 749 cm^{-1}.

^{1}H NMR (400 MHz, $CDCl_3$): δ = 8.26 – 8.18 (m, 2H), 7.88 (d, J = 8.4 Hz, 1H), 7.71 (d, J = 8.6 Hz, 1H), 7.61 – 7.55 (m, 1H), 7.53 – 7.44 (m, 3H), 7.22 (d, J = 8.7 Hz, 1H), 2.64 (s, 3H) ppm.

^{19}F NMR (376 MHz, $CDCl_3$): δ = -83.00 – -83.37 (m, 3F), -114.29 (s, 2F) ppm.

^{13}C NMR (100 MHz, $CDCl_3$): δ = 150.7 (t, J = 1.4 Hz), 145.6, 138.1, 135.6 (t, J = 32.4 Hz), 134.2, 131.4, 129.8, 128.9, 128.4, 126.2, 124.7, 122.8 (t, J = 1.6 Hz), 121.0, 120.4, 119.5, 119.3, 115.6, 109.7, 21.9 ppm; carbons corresponding to the C_2F_5 group cannot be identified due to C-F coupling.

HRMS m/z: calcd for $C_{21}H_{13}F_5N_3O$ $[M+H]^+$ 418.0973, found: 418.0979.

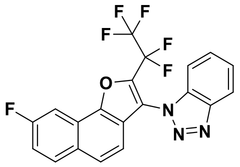

1-(8-Fluoro-2-(perfluoroethyl)naphtho[1,2-*b*]furan-3-yl)-1*H*-benzo[*d*][1,2,3]triazole (16f):

Yield = 80% (67 mg), 0.2 mmol scale. Colorless oil.

IR (KBr): ν = 3073, 1629, 1596, 832, 734 cm^{-1}.

^{1}H NMR (400 MHz, $CDCl_3$): δ = 8.27 – 8.21 (m, 1H), 8.07 – 7.97 (m, 2H), 7.77 (d, J = 8.7 Hz, 1H), 7.63 –7.58 (m, 1H), 7.55 – 7.50 (m, 1H), 7.48 (d, J = 8.3 Hz, 1H), 7.46 – 7.40 (m, 1H), 7.29 (d, J = 8.7 Hz, 1H) ppm.

^{19}F NMR (376 MHz, $CDCl_3$): δ = -83.00 – -83.26 (m, 3F), -109.58 – -110.06 (m, 1F), -114.38 (s,

2F) ppm.

^{13}C NMR (100 MHz, $CDCl_3$): δ = 161.7 (d, J = 248.8 Hz), 150.4 (dt, J = 5.3, 1.2 Hz), 145.6, 136.3 (t, J = 32.4 Hz), 134.2, 131.3 (d, J = 9.2 Hz), 130.1 (d, J = 1.4 Hz), 129.0, 126.2 (d, J = 1.2 Hz), 124.8, 122.9 (t, J = 1.4 Hz), 121.7 (d, J = 10.0 Hz), 120.5, 120.4, 117.7 (d, J = 24.8 Hz), 116.0 (d, J = 2.6 Hz), 109.6, 104.8 (d, J = 23.4 Hz) ppm; carbons corresponding to the C_2F_5 group cannot be identified due to C-F coupling.

HRMS m/z: calcd for $C_{20}H_{10}F_6N_3O$ [M+H]$^+$ 422.0723, found: 422.0726.

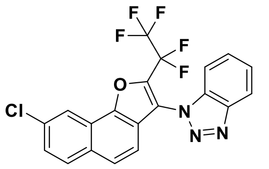

1-(8-Chloro-2-(perfluoroethyl)naphtho[1,2-*b*]furan-3-yl)-1*H*-benzo[*d*][1,2,3]triazole (16g):

Yield = 65% (57 mg), 0.2 mmol scale. White solid. M.p. 135.7–137.1 ^{o}C.

IR (KBr): ν = 3066, 1749, 1491, 812, 748 cm^{-1}.

^{1}H NMR (400 MHz, $CDCl_3$): δ = 8.41 (d, J = 2.1 Hz, 1H), 8.25 – 8.21 (m, 1H), 7.94 (d, J = 8.7 Hz, 1H), 7.76 (d, J = 8.6 Hz, 1H), 7.64 – 7.58 (m, 2H), 7.55 – 7.45 (m, 2H), 7.33 (d, J = 8.7 Hz, 1H) ppm.

^{19}F NMR (376 MHz, $CDCl_3$): δ = -82.96 – -83.28 (m, 3F), -114.36 (s, 2F) ppm.

^{13}C NMR (100 MHz, $CDCl_3$): δ = 149.8 (t, J = 1.4 Hz), 145.6, 136.3 (t, J = 32.5 Hz), 134.2, 134.0, 131.3, 130.2, 129.0, 128.6, 126.1, 124.8, 122.9 (t, J = 2.0 Hz), 121.5, 120.5, 120.4, 119.5, 117.0, 109.6 ppm; carbons corresponding to the C_2F_5 group cannot be identified due to C-F coupling.

HRMS m/z: calcd for $C_{20}H_{10}ClF_5N_3O$ [M+H]$^+$ 438.0427, found: 438.0437.

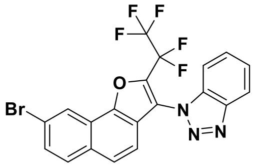

1-(8-Bromo-2-(perfluoroethyl)naphtho[1,2-*b*]furan-3-yl)-1*H*-benzo[*d*][1,2,3]triazole (16h):

Yield = 73% (70 mg), 0.2 mmol scale. Light brown solid. M.p. 156.1–156.7 ^{o}C.

IR (KBr): ν = 3067, 1630, 1611, 803, 733 cm^{-1}.

^{1}H NMR (400 MHz, $CDCl_3$): δ = 8.60 – 8.54 (m, 1H), 8.26 – 8.20 (m, 1H), 7.89 – 7.83 (m, 1H), 7.77 – 7.70 (m, 2H), 7.64 – 7.57 (m, 1H), 7.55 – 7.45 (m, 2H), 7.37 – 7.32 (m, 1H) ppm.

^{19}F NMR (376 MHz, $CDCl_3$): δ = -83.07 (t, J = 2.5 Hz, 3F), -114.35 (s, 2F) ppm.

^{13}C NMR (100 MHz, $CDCl_3$): δ = 149.6 (t, J = 1.4 Hz), 145.6, 136.3 (t, J = 32.2 Hz), 134.2, 131.5, 131.1, 130.2, 129.0, 126.2, 124.8, 122.9 (t, J = 1.5 Hz), 122.7, 122.2, 121.8, 120.5, 120.4, 117.2, 109.5 ppm; carbons corresponding to the C_2F_5 group cannot be identified due to C-F coupling.

HRMS m/z: calcd for $C_{20}H_{10}BrF_5N_3O$ $[M+H]^+$ 481.9922, found: 481.9924.

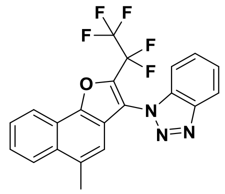

1-(5-Methyl-2-(perfluoroethyl)naphtho[1,2-*b*]furan-3-yl)-1*H*-benzo[*d*][1,2,3]triazole (16j):

Yield = 59% (49 mg), 0.2 mmol scale. White solid. M.p. 122.9–124.2 ^{o}C.

IR (KBr): ν = 3071, 1630, 1613, 785, 756 cm^{-1}.

^{1}H NMR (400 MHz, $CDCl_3$): δ = 8.50 – 8.44 (m, 1H), 8.24 (d, J = 8.3 Hz, 1H), 8.10 (d, J = 7.7 Hz, 1H), 7.80 – 7.68 (m, 2H), 7.64 – 7.56 (m, 1H), 7.54 – 7.44 (m, 2H), 7.14 (s, 1H), 2.66 (s, 3H) ppm.

^{19}F NMR (376 MHz, $CDCl_3$): δ = -83.18 (t, J = 4.0 Hz, 3F), -114.23 (s, 2F) ppm.

^{13}C NMR (100 MHz, $CDCl_3$): δ = 150.2 (t, J = 1.3 Hz), 145.6, 135.5 (t, J = 32.5 Hz), 134.3, 133.1, 132.5, 128.9, 127.6, 127.4, 125.3, 124.7, 122.5 (t, J = 1.3 Hz), 120.9, 120.7, 120.4, 119.1, 116.2, 109.7, 19.8 ppm; carbons corresponding to the C_2F_5 group cannot be identified due to C-F coupling.

HRMS m/z: calcd for $C_{21}H_{13}F_5N_3O$ $[M+H]^+$ 418.0973, found: 418.0982.

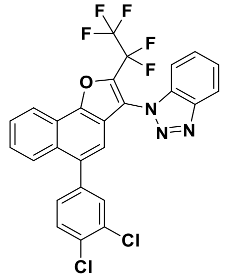

1-(5-(3,4-Dichlorophenyl)-2-(perfluoroethyl)naphtho[1,2-*b*]furan-3-yl)-1*H*-

benzo[*d*][1,2,3]triazole (16k):

Yield = 95% (104 mg), 0.2 mmol scale. White solid. M.p. 173.0–174.1 °C.

IR (KBr): ν = 3068, 1639, 1591, 819, 756 cm^{-1}.

^{1}H NMR (400 MHz, $CDCl_3$): δ = 8.55 (d, *J* = 8.2 Hz, 1H), 8.25 – 8.19 (m, 1H), 7.89 (d, *J* = 8.5 Hz, 1H), 7.80 (t, *J* = 7.5 Hz, 1H), 7.70 – 7.64 (m, 1H), 7.63 – 7.57 (m, 1H), 7.55 – 7.47 (m, 4H), 7.25 (d, *J* = 7.2 Hz, 2H) ppm.

^{19}F NMR (376 MHz, $CDCl_3$): δ = -83.19 (t, *J* = 3.6 Hz, 3F), -114.42 (s, 2F) ppm.

^{13}C NMR (100 MHz, $CDCl_3$): δ = 150.8 (t, *J* = 1.2 Hz), 145.6, 139.4, 136.7, 136.1 (t, *J* = 32.5 Hz), 134.2, 132.6, 132.2, 131.7, 131.5, 130.3, 129.4, 129.1, 128.1, 128.0, 126.8, 124.8, 122.9 (t, *J* = 1.1 Hz), 121.1, 120.7, 120.5, 119.0, 117.4, 109.5 ppm; carbons corresponding to the C_2F_5 group cannot be identified due to C-F coupling.

HRMS m/z: calcd for $C_{26}H_{13}Cl_2F_5N_3O$ $[M+H]^+$ 548.0350, found: 548.0358.

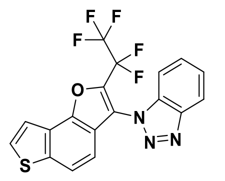

1-(2-(Perfluoroethyl)thieno[2,3-*g*]benzofuran-3-yl)-1*H*-benzo[*d*][1,2,3]triazole (16l):

Yield = 96% (79 mg), 0.2 mmol scale. Yellow solid. M.p. 111.7–112.9 °C.

IR (KBr): ν = 3068, 1630, 1612, 797, 748 cm^{-1}.

^{1}H NMR (400 MHz, $CDCl_3$): δ = 8.23 (d, *J* = 8.3 Hz, 1H), 7.87 – 7.81 (m, 2H), 7.72 (d, *J* = 5.5 Hz, 1H), 7.62 – 7.56 (m, 1H), 7.53 – 7.48 (m, 1H), 7.46 (d, *J* = 8.2 Hz, 1H), 7.25 (d, *J* = 8.6 Hz, 1H) ppm.

^{19}F NMR (376 MHz, $CDCl_3$): δ = -83.15 (t, *J* = 3.9 Hz, 3F), -114.47 (s, 2F) ppm.

^{13}C NMR (100 MHz, $CDCl_3$): δ = 149.3 (t, *J* = 1.4 Hz), 145.6, 141.4, 135.1 (t, *J* = 32.3 Hz), 134.2, 129.0, 128.9, 125.4, 124.7, 122.8 (t, *J* = 1.5 Hz), 120.5, 120.1, 119.5, 118.7, 115.4, 109.6 ppm; carbons corresponding to the C_2F_5 group cannot be identified due to C-F coupling.

HRMS m/z: calcd for $C_{18}H_9F_5N_3OS$ $[M+H]^+$ 410.0381, found: 410.0384.

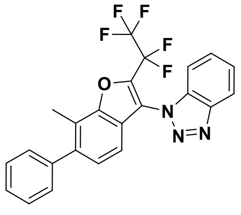

1-(7-Methyl-2-(perfluoroethyl)-6-phenylbenzofuran-3-yl)-1*H*-benzo[*d*][1,2,3]triazole (16m):

Yield = 60% (53 mg), 0.2 mmol scale. Yellow oil.

IR (KBr): ν = 3062, 1637, 1613, 772, 743 cm^{-1}.

^{1}H NMR (400 MHz, $CDCl_3$): δ = 8.25 – 8.19 (m, 1H), 7.62 – 7.56 (m, 1H), 7.53 – 7.45 (m, 4H), 7.44 – 7.36 (m, 3H), 7.33 (d, *J* = 8.2 Hz, 1H), 7.23 (d, *J* = 8.2 Hz, 1H), 2.57 (s, 3H) ppm.

^{19}F NMR (376 MHz, $CDCl_3$): *δ* = -83.11 (t, *J* = 3.1 Hz, 3F), -114.92 (s, 2F) ppm.

^{13}C NMR (100 MHz, $CDCl_3$): *δ* = 153.8 (t, *J* = 1.1 Hz), 145.6, 142.5, 139.8, 136.8 (t, *J* = 32.0 Hz), 134.2, 129.4, 128.9, 128.4, 127.6, 127.6, 124.7, 122.0 (t, *J* = 1.0 Hz), 121.9, 121.0, 120.4, 117.1, 109.6, 12.5 ppm; carbons corresponding to the C_2F_5 group cannot be identified due to C-F coupling.

HRMS m/z: calcd for $C_{23}H_{15}F_5N_3O$ $[M+H]^+$ 444.1130, found: 444.1128.

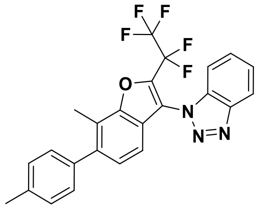

1-(7-Methyl-2-(perfluoroethyl)-6-(*p*-tolyl)benzofuran-3-yl)-1*H*-benzo[*d*][1,2,3]triazole (16n):

Yield = 78% (71 mg), 0.2 mmol scale. Yellow oil.

IR (KBr): ν = 2927, 1682, 1623, 766, 746 cm^{-1}.

^{1}H NMR (400 MHz, $CDCl_3$): *δ* = 8.25 – 8.18 (m, 1H), 7.62 – 7.55 (m, 1H), 7.53 – 7.45 (m, 2H), 7.34 – 7.26 (m, 5H), 7.21 (d, *J* = 8.2 Hz, 1H), 2.57 (s, 3H), 2.43 (s, 3H) ppm.

^{19}F NMR (376 MHz, $CDCl_3$): *δ* = -82.86 – -83.38 (m, 3F), -114.89 (s, 2F) ppm.

^{13}C NMR (100 MHz, $CDCl_3$): *δ* = 153.9 (t, *J* = 1.0 Hz), 145.6, 142.6, 137.4, 136.9, 136.7 (t, *J* = 31.9 Hz), 134.2, 129.2, 129.1, 128.8, 127.7, 124.7, 122.0 (t, *J* = 1.7 Hz), 121.8, 121.0, 120.4, 117.0, 109.6, 21.1, 12.5 ppm; carbons corresponding to the C_2F_5 group cannot be identified

due to C-F coupling.

HRMS m/z: calcd for $C_{24}H_{17}F_5N_3O$ $[M+H]^+$ 458.1286, found: 458.1287.

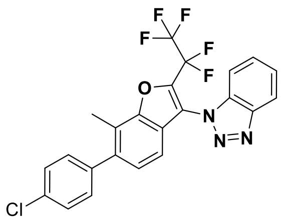

1-(6-(4-Chlorophenyl)-7-methyl-2-(perfluoroethyl)benzofuran-3-yl)-1*H*-benzo[*d*][1,2,3]triazole (16o):

Yield = 72% (69 mg), 0.2 mmol scale. Yellow solid. M.p. 95.5– 96.3 °C.

IR (KBr): ν = 3066, 1634, 1614, 747, 729 cm^{-1}.

^{1}H NMR (400 MHz, $CDCl_3$): δ = 8.22 (d, *J* = 8.3 Hz, 1H), 7.63 – 7.56 (m, 1H), 7.53 – 7.50 (m, 1H), 7.49 – 7.43 (m, 3H), 7.33 – 7.27 (m, 3H), 7.24 (d, *J* = 8.2 Hz, 1H), 2.56 (s, 3H) ppm.

^{19}F NMR (376 MHz, $CDCl_3$): δ = -83.11 (t, *J* = 2.6 Hz, 3F), -114.92 (s, 2F) ppm.

^{13}C NMR (100 MHz, $CDCl_3$): δ = 153.8 (t, *J* = 1.0 Hz), 145.6, 141.2, 138.2, 137.0 (t, *J* = 31.9 Hz), 134.2, 133.8, 130.7, 128.9, 128.6, 127.4, 124.7, 122.2, 122.0 (t, *J* = 1.8 Hz), 121.1, 120.5, 117.3, 109.6, 12.4 ppm; carbons corresponding to the C_2F_5 group cannot be identified due to C-F coupling.

HRMS m/z: calcd for $C_{23}H_{14}ClF_5N_3O$ $[M+H]^+$ 478.0740, found: 478.0743.

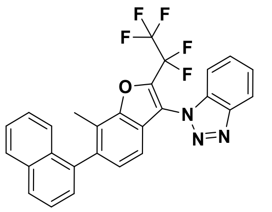

1-(7-Methyl-6-(naphthalen-1-yl)-2-(perfluoroethyl)benzofuran-3-yl)-1*H*-benzo[*d*][1,2,3]triazole (16p):

Yield = 78% (77 mg), 0.2 mmol scale. Yellow solid. M.p. 68.1– 68.3 °C.

IR (KBr): ν = 3062, 1636, 1593, 746, 731 cm^{-1}.

^{1}H NMR (400 MHz, $CDCl_3$): δ = 8.25 (d, *J* = 8.3 Hz, 1H), 7.95 (dd, *J* = 8.1, 4.0 Hz, 2H), 7.66 – 7.61 (m, 1H), 7.60 – 7.43 (m, 6H), 7.42 – 7.38 (m, 1H), 7.37 – 7.28 (m, 2H), 2.35 (s, 3H) ppm.

^{19}**F NMR** (376 MHz, $CDCl_3$): δ = -83.03 (d, J = 2.6 Hz, 3F), -114.80 (s, 2F) ppm.

^{13}**C NMR** (100 MHz, $CDCl_3$): δ = 153.6, 145.6, 141.1, 137.4, 136.9 (t, J = 31.9 Hz), 134.2, 133.5, 131.7, 128.9, 128.4, 128.2, 127.1, 126.4, 126.0, 125.6, 125.3, 124.7, 122.6, 122.3, 122.2, 122.1, 120.5, 116.9, 109.7, 12.3 ppm; carbons corresponding to the C_2F_5 group cannot be identified due to C-F coupling.

HRMS m/z: calcd for $C_{27}H_{17}F_5N_3O$ $[M+H]^+$ 494.1286, found: 494.1287.

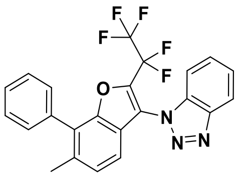

1-(6-Methyl-2-(perfluoroethyl)-7-phenylbenzofuran-3-yl)-1*H*-benzo[*d*][1,2,3]triazole (16q):

Yield = 84% (74 mg), 0.2 mmol scale. Yellow solid. M.p. 67.7– 68.1 °C.

IR (KBr): ν = 3060, 1636, 1612, 748, 723 cm^{-1}.

^{1}**H NMR** (400 MHz, $CDCl_3$): δ = 8.21 (d, J = 8.7 Hz, 1H), 7.61 – 7.56 (m, 1H), 7.55 – 7.52 (m, 2H), 7.51 – 7.45 (m, 5H), 7.34 (d, J = 8.2 Hz, 1H), 7.23 (d, J = 8.1 Hz, 1H), 2.43 (s, 3H) ppm.

^{19}**F NMR** (376 MHz, $CDCl_3$): δ = -83.09 – -83.50 (m, 3F), -114.87 (s, 2F) ppm.

^{13}**C NMR** (100 MHz, $CDCl_3$): δ = 152.6 (t, J = 1.2 Hz), 145.6, 137.3, 136.4 (t, J = 31.5 Hz), 134.2, 133.2, 129.9, 128.8, 128.5, 128.2, 128.2, 126.9, 124.7, 121.8 (t, J = 1.1 Hz), 121.4, 120.4, 118.5, 109.7, 20.1 ppm; carbons corresponding to the C_2F_5 group cannot be identified due to C-F coupling.

HRMS m/z: calcd for $C_{23}H_{15}F_5N_3O$ $[M+H]^+$ 444.1130, found: 444.1132.

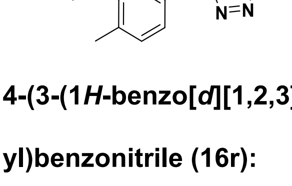

4-(3-(1*H*-benzo[*d*][1,2,3]triazol-1-yl)-6-methyl-2-(perfluoroethyl)benzofuran-7-yl)benzonitrile (16r):

Yield = 80% (75 mg), 0.2 mmol scale. Yellow solid. M.p. 93.6– 94.3 °C.

IR (KBr): ν = 2992, 2228, 1637, 1607, 767 cm^{-1}.

^{1}**H NMR** (400 MHz, $CDCl_3$): δ = 8.20 (d, J = 8.3 Hz, 1H), 7.85 (d, J = 8.0 Hz, 2H), 7.62 (d, J = 8.2 Hz, 2H), 7.60 – 7.55 (m, 1H), 7.53 – 7.45 (m, 2H), 7.40 – 7.29 (m, 2H), 2.42 (s, 3H) ppm.

^{19}**F NMR** (376 MHz, $CDCl_3$): δ = -83.22 (s, 3F), -114.91 (s, 2F) ppm.

^{13}**C NMR** (100 MHz, $CDCl_3$): δ = 152.0, 145.5, 138.1, 137.1, 136.5 (t, J = 32.3 Hz), 134.1, 132.3, 130.8, 128.9, 128.4, 124.8, 124.8, 122.0, 121.6, 120.4, 119.7, 118.5, 112.2, 109.5, 20.0 ppm; carbons corresponding to the C_2F_5 group cannot be identified due to C-F coupling.

HRMS m/z: calcd for $C_{24}H_{14}F_5N_4O$ $[M+H]^+$ 469.1082, found: 469.1082.

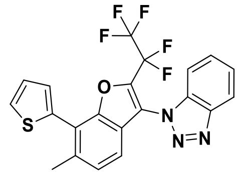

1-(6-Methyl-2-(perfluoroethyl)-7-(thiophen-2-yl)benzofuran-3-yl)-1*H*-benzo[*d*][1,2,3]triazole (16s):

Yield = 59% (53 mg), 0.2 mmol scale. Yellow solid. M.p. 114.9– 115.8 °C.

IR (KBr): ν = 2962, 1639, 1613, 745, 701 cm^{-1}.

^{1}**H NMR** (400 MHz, $CDCl_3$): δ = 8.26 – 8.19 (m, 1H), 7.62 – 7.56 (m, 2H), 7.54 – 7.44 (m, 2H), 7.40 – 7.32 (m, 2H), 7.27 – 7.22 (m, 2H), 2.61 (s, 3H) ppm.

^{19}**F NMR** (376 MHz, $CDCl_3$): δ = -82.27 – -84.16 (m, 3F), -114.79 (s, 2F) ppm.

^{13}**C NMR** (100 MHz, $CDCl_3$): δ = 152.5, 150.7 (t, J = 1.1 Hz), 145.6, 138.1, 136.1 (t, J = 32.0 Hz), 134.2, 133.1, 129.2, 128.9, 128.5, 127.2, 127.1, 124.7, 121.7, 120.5, 120.1, 118.9, 109.7, 21.1 ppm; carbons corresponding to the C_2F_5 group cannot be identified due to C-F coupling.

HRMS m/z: calcd for $C_{21}H_{13}F_5N_3OS$ $[M+H]^+$ 450.0694, found: 450.0692.

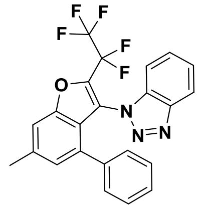

1-(6-Methyl-2-(perfluoroethyl)-4-phenylbenzofuran-3-yl)-1*H*-benzo[*d*][1,2,3]triazole (16t):

Yield = 62% (55 mg), 0.2 mmol scale. Yellow solid. M.p. 104.8– 105.4 °C.

IR (KBr): ν = 3034, 1635, 1621, 756, 745 cm^{-1}.

^{1}H NMR (400 MHz, $CDCl_3$): δ = 7.91 – 7.85 (m, 1H), 7.54 (s, 1H), 7.28 – 7.19 (m, 2H), 7.15 (s, 1H), 6.99 – 6.93 (m, 1H), 6.79 (d, J = 7.3 Hz, 3H), 6.75 – 6.68 (m, 2H), 2.58 (s, 3H) ppm.

^{19}F NMR (376 MHz, $CDCl_3$): δ = -83.47 (t, J = 2.5 Hz, 3F), -115.71 (dd, J = 28.2, 2.6 Hz, 2F) ppm.

^{13}C NMR (100 MHz, $CDCl_3$): δ = 155.0, 145.0, 139.4, 138.1 (t, J = 30.0 Hz), 136.7, 136.3, 134.7, 128.1, 127.8, 127.4, 127.3, 127.2, 123.8, 121.6 (m), 119.6, 119.1, 111.6, 109.2, 21.8 ppm; carbons corresponding to the C_2F_5 group cannot be identified due to C-F coupling.

HRMS m/z: calcd for $C_{23}H_{15}F_5N_3O$ [M+H]$^+$ 444.1130, found: 444.1127.

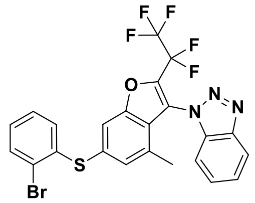

1-(6-((2-Bromophenyl)thio)-4-methyl-2-(perfluoroethyl)benzofuran-3-yl)-1*H*-benzo[*d*][1,2,3]triazole (16u):

Yield = 75% (83 mg), 0.2 mmol scale. Yellow oil.

IR (KBr): ν = 3063, 1633, 1609, 783, 744 cm^{-1}.

^{1}H NMR (400 MHz, $CDCl_3$): δ = 8.20 (d, J = 8.3 Hz, 1H), 7.70 – 7.61 (m, 1H), 7.58 (d, J = 7.3 Hz, 1H), 7.53 – 7.44 (m, 1H), 7.43 – 7.33 (m, 2H), 7.28 (d, J = 9.9 Hz, 2H), 7.19 (s, 1H), 7.11 (s, 1H), 1.67 (s, 3H) ppm.

^{19}F NMR (376 MHz, $CDCl_3$): δ = -82.95 – -83.97 (m, 3F), -114.71 – -117.31 (m, 2F) ppm.

^{13}C NMR (100 MHz, $CDCl_3$): δ = 154.8, 145.3, 138.5 (t, J = 31.6 Hz), 136.6, 135.4, 135.3, 133.6, 133.2, 133.2, 129.3, 129.2, 128.8, 128.3, 126.2, 124.7, 122.0, 121.8 (m), 120.4, 112.1, 109.3, 16.9 ppm; carbons corresponding to the C_2F_5 group cannot be identified due to C-F coupling.

HRMS m/z: calcd for $C_{23}H_{14}BrF_5N_3OS$ [M+H]$^+$ 553.9956, found: 553.9954.

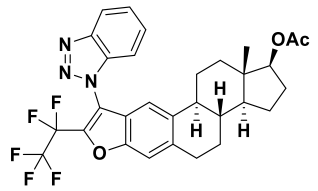

(1*S*,3a*S*,3b*R*,10b*S*,12a*S*)-9-(1*H*-Benzo[*d*][1,2,3]triazol-1-yl)-12a-methyl-8-(perfluoroethyl)-2,3,3a,3b,4,5,10b,11,12,12a-decahydro-1*H*-cyclopenta[7,8]phenanthro[2,3-*b*]furan-1-yl acetate (16v):

Yield = 58% (17 mg), 0.05 mmol scale. Yellow solid. M.p. 96.1– 96.5 ^{o}C.

IR (KBr): ν = 3441, 2930, 1736, 1633, 745 cm^{-1}.

^{1}**H NMR** (400 MHz, CDCl$_3$): δ = 8.25 – 8.18 (m, 1H), 7.61 – 7.55 (m, 1H), 7.52 – 7.47 (m, 1H), 7.46 – 7.41 (m, 2H), 7.22 (s, 1H), 4.65 (dd, *J* = 9.1, 7.8 Hz, 1H), 3.13 – 3.03 (m, 2H), 2.30 – 2.19 (m, 2H), 2.16 – 2.08 (m, 1H), 2.04 (s, 3H), 1.99 – 1.90 (m, 1H), 1.84 – 1.78 (m, 1H), 1.77 – 1.71 (m, 1H), 1.55 – 1.39 (m, 5H), 1.32 – 1.26 (m, 2H), 0.79 (s, 3H) ppm.

^{19}**F NMR** (376 MHz, CDCl$_3$): δ = -83.19 (t, *J* = 3.3 Hz, 3F), -114.87 – -115.40 (m, 2F) ppm.

^{13}**C NMR** (100 MHz, CDCl$_3$): δ = 171.2, 152.9, 145.5, 139.5, 139.0, 136.1 (t, *J* = 30.5 Hz), 134.3, 128.8, 124.7, 121.8 (t, *J* = 1.7 Hz), 121.5, 120.4, 116.2, 111.9, 109.7, 82.5, 50.0, 43.9, 42.7, 38.0, 36.5, 30.1, 27.5, 26.8, 26.1, 23.3, 21.2, 11.9 ppm; carbons corresponding to the C_2F_5 group cannot be identified due to C-F coupling.

HRMS m/z: calcd for $C_{30}H_{29}F_5N_3O_3$ [M+H]$^+$ 574.2124, found: 574.2128.

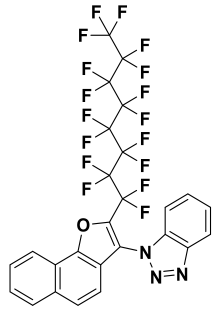

1-(2-(Perfluorooctyl)naphtho[1,2-*b*]furan-3-yl)-1*H*-benzo[*d*]imidazole (16w):

Yield = 67% (94 mg), 0.2 mmol scale. Yellow solid. M.p. 125.5–126.6 ^{o}C.

IR (KBr): ν = 3057, 1749, 1650, 815, 747 cm^{-1}.

^{1}**H NMR** (400 MHz, CDCl$_3$): δ = 8.46 (d, *J* = 8.1 Hz, 1H), 8.24 (d, *J* = 8.3 Hz, 1H), 8.00 (d, *J* = 8.1 Hz, 1H), 7.81 – 7.72 (m, 2H), 7.68 (t, *J* = 7.4 Hz, 1H), 7.59 (t, *J* = 7.5 Hz, 1H), 7.51 (t, *J* = 7.5 Hz, 1H), 7.45 (d, *J* = 8.2 Hz, 1H), 7.28 (d, *J* = 8.7 Hz, 1H) ppm.

^{19}**F NMR** (376 MHz, CDCl$_3$): δ = -80.75 (t, *J* = 9.9 Hz, 3F), -110.95 (s, 2F), -121.28 – -122.12 (m, 8F), -122.73 (s, 2F), -126.01 – -126.28 (m, 2F) ppm.

^{13}**C NMR** (100 MHz, $CDCl_3$): δ = 151.0 (t, J = 1.4 Hz), 145.6, 136.1 (t, J = 32.8 Hz), 134.3, 133.2, 128.9, 128.6, 127.8, 127.7, 126.4, 124.7, 123.0 (t, J = 1.9 Hz), 120.9, 120.5, 120.3, 119.6, 116.6, 109.6 ppm; carbons corresponding to the C_8F_{17} group cannot be identified due to C-F coupling.

HRMS m/z: calcd for $C_{26}H_{11}F_{17}N_3O$ $[M+H]^+$ 704.0625, found: 704.0640.

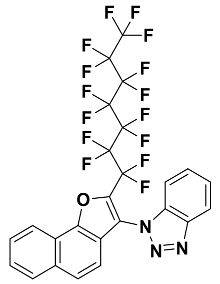

1-(2-(Perfluoroheptyl)naphtho[1,2-*b*]furan-3-yl)-1*H*-benzo[*d*]imidazole (16x):

Yield = 85% (167 mg), 0.2 mmol scale. Yellow solid. M.p. 119.4–119.7 ^{o}C.

IR (KBr): ν = 3072, 1631, 1613, 810, 749 cm^{-1}.

^{1}**H NMR** (400 MHz, $CDCl_3$): δ = 8.42 (d, J = 8.1 Hz, 1H), 8.23 (d, J = 8.3 Hz, 1H), 7.97 (d, J = 8.1 Hz, 1H), 7.77 – 7.69 (m, 2H), 7.69 – 7.63 (m, 1H), 7.62 – 7.55 (m, 1H), 7.53 – 7.44 (m, 2H), 7.29 (d, J = 8.7 Hz, 1H) ppm.

^{19}**F NMR** (376 MHz, $CDCl_3$): δ = -80.67 (t, J = 9.8 Hz, 3F), -110.96 (s, 2F), -121.32 – -121.68 (m, 2F), -121.90 (s, 2F), -122.67 (s, 2F), -126.00 – -126.20 (m, 2F) ppm.

^{13}**C NMR** (100 MHz, $CDCl_3$): δ = 150.9 (t, J = 1.3 Hz), 145.6, 135.7 (t, J = 32.3 Hz), 134.2, 133.1, 128.9, 128.6, 127.8, 127.7, 126.4, 124.7, 122.8 (t, J = 1.6 Hz), 120.8, 120.4, 120.2, 119.4, 116.5, 109.6 ppm; carbons corresponding to the C_7F_{15} group cannot be identified due to C-F coupling.

HRMS m/z: calcd for $C_{25}H_{11}F_{15}N_3O$ $[M+H]^+$ 654.0657, found: 654.0660.

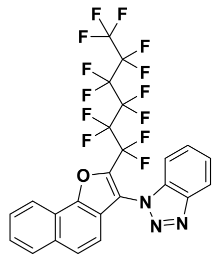

1-(2-(Perfluorohexyl)naphtho[1,2-*b*]furan-3-yl)-1*H*-benzo[*d*]imidazole (16y):

Yield = 70% (84 mg), 0.2 mmol scale). White solid. M.p. 105.7–106.2 ^{o}C.

IR (KBr): ν = 3073, 1627, 1533, 805, 720 cm^{-1}.

^{1}H NMR (400 MHz, $CDCl_3$): δ = 8.46 (d, J = 8.2 Hz, 1H), 8.24 (d, J = 8.3 Hz, 1H), 8.00 (d, J = 8.1 Hz, 1H), 7.81 – 7.72 (m, 2H), 7.71 – 7.65 (m, 1H), 7.62 – 7.55 (m, 1H), 7.54 – 7.48 (m, 1H), 7.45 (d, J = 8.2 Hz, 1H), 7.28 (d, J = 8.7 Hz, 1H) ppm.

^{19}F NMR (376 MHz, $CDCl_3$): δ = -80.71 (t, J = 10.7 Hz, 3F), -110.91 (s, 2F), -121.07 – -121.54 (m, 2F), -121.59 – -122.18 (m, 2F), -122.50 – -123.07 (m, 2F), -125.87 – -126.33 (m, 2F) ppm.

^{13}C NMR (100 MHz, $CDCl_3$): δ = 151.0 (t, J = 1.0 Hz), 145.6, 136.1 (t, J = 32.3 Hz), 134.4, 133.2, 128.9, 128.7, 127.9, 127.8, 126.4, 124.7, 123.0 (t, J = 1.0 Hz), 120.9, 120.5, 120.3, 119.6, 116.6, 109.6 ppm; carbons corresponding to the C_6F_{13} group cannot be identified due to C-F coupling.

HRMS m/z: calcd for $C_{24}H_{11}F_{13}N_3O$ $[M+H]^+$ 604.0689, found: 604.0698.

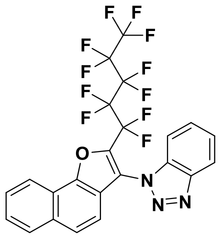

1-(2-(Perfluoropentyl)naphtho[1,2-*b*]furan-3-yl)-1*H*-benzo[*d*][1,2,3]triazole (16z):

Yield = 72% (80 mg), 0.2 mmol scale. Yellow solid. M.p. 118.6–120.5 °C.

IR (KBr): ν = 3056, 1629, 1533, 814, 746 cm^{-1}.

^{1}H NMR (400 MHz, $CDCl_3$): δ = 8.49 – 8.43 (m, 1H), 8.26 – 8.20 (m, 1H), 8.00 (d, J = 8.1 Hz, 1H), 7.79 – 7.72 (m, 2H), 7.71 – 7.65 (m, 1H), 7.62 – 7.56 (m, 1H), 7.53 – 7.48 (m, 1H), 7.45 (d, J = 8.2 Hz, 1H), 7.28 (d, J = 8.7 Hz, 1H) ppm.

^{19}F NMR (376 MHz, $CDCl_3$): δ = -80.67 (t, J = 10.6 Hz, 3F), -110.93 (s, 2F), -121.63 (s, 2F), -122.23 – -123.01 (m, 2F), -125.77 – -126.34 (m, 2F) ppm.

^{13}C NMR (100 MHz, $CDCl_3$): δ = 151.0 (t, J = 1.1 Hz), 145.6, 136.0 (t, J = 32.6 Hz), 134.4, 133.2, 128.9, 128.7, 127.9, 127.8, 126.5, 124.7, 123.0 (t, J = 2.0 Hz), 120.9, 120.5, 120.3, 119.6, 116.6, 109.6 ppm; carbons corresponding to the C_5F_{11} group cannot be identified due to C-F coupling.

HRMS m/z: calcd for $C_{23}H_{11}F_{11}N_3O$ $[M+H]^+$ 554.0721, found: 554.0722.

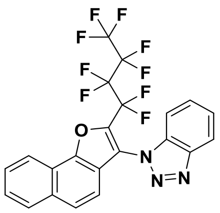

1-(2-(Perfluorobutyl)naphtho[1,2-*b*]furan-3-yl)-1*H*-benzo[*d*][1,2,3]triazole (16a'):

Yield = 67% (67 mg), 0.2 mmol scale. White solid. M.p. 112.7–114.1 ^{o}C.

IR (KBr): ν = 3075, 1629, 1491, 805, 732 cm^{-1}.

^{1}H NMR (400 MHz, $CDCl_3$): δ = 8.46 (d, *J* = 8.1 Hz, 1H), 8.24 (d, *J* = 8.3 Hz, 1H), 8.00 (d, *J* = 8.1 Hz, 1H), 7.81 – 7.73 (m, 2H), 7.72 – 7.64 (m, 1H), 7.63 – 7.56 (m, 1H), 7.54 – 7.48 (m, 1H), 7.45 (d, *J* = 8.2 Hz, 1H), 7.28 (d, *J* = 8.7 Hz, 1H) ppm.

^{19}F NMR (376 MHz, $CDCl_3$): δ = -80.77 (t, *J* = 10.3 Hz, 3F), -111.08 (s, 2F), -121.61 – -123.02 (m, 2F), -125.87 (td, *J* = 13.2, 5.9 Hz, 2F) ppm.

^{13}C NMR (100 MHz, $CDCl_3$): δ = 151.0 (t, *J* = 1.6 Hz), 145.6, 136.0 (t, *J* = 33.2 Hz), 134.3, 133.2, 128.9, 128.7, 127.9, 127.8, 126.4, 124.7, 123.0 (t, *J* = 2.2 Hz), 120.9, 120.5, 120.3, 119.6, 116.6, 109.6 ppm; carbons corresponding to the C_4F_9 group cannot be identified due to C-F coupling.

HRMS m/z: calcd for $C_{22}H_{11}F_9N_3O$ [M+H]$^{+}$ 504.0753, found: 504.0762.

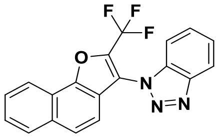

1-(2-(Trifluoromethyl)naphtho[1,2-*b*]furan-3-yl)-1*H*-benzo[*d*][1,2,3]triazole (16b'):

Yield = 50% (35 mg), 0.2 mmol scale. Yellow solid. M.p. 86.1–87.3 ^{o}C.

IR (KBr): ν = 3063, 1627, 1613, 808, 745 cm^{-1}.

^{1}H NMR (400 MHz, $CDCl_3$): δ = 8.46 (d, *J* = 8.2 Hz, 1H), 8.24 (d, *J* = 9.0 Hz, 1H), 8.00 (d, *J* = 8.1 Hz, 1H), 7.84 – 7.72 (m, 2H), 7.71 – 7.65 (m, 1H), 7.64 – 7.58 (m, 1H), 7.57 – 7.47 (m, 2H), 7.41 (d, *J* = 8.7 Hz, 1H) ppm.

^{19}F NMR (376 MHz, $CDCl_3$): δ = -61.98 (s, 3F) ppm.

^{13}C NMR (100 MHz, $CDCl_3$): δ = 150.2 (t, *J* = 1.0 Hz), 145.7, 136.2 (t, *J* = 32.1 Hz), 133.9, 133.2, 129.0, 128.6, 127.8, 127.6, 126.3, 124.8, 120.9, 120.5, 120.3, 120.3 (q, *J* = 297.3 Hz), 119.0, 117.5, 117.0, 109.7 ppm

HRMS m/z: calcd for $C_{19}H_{11}F_3N_3O$ $[M+H]^+$ 354.0849, found: 354.0850.

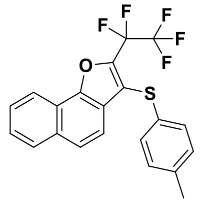

2-(Perfluoroethyl)-3-(*p*-tolylthio)naphtho[1,2-*b*]furan (17):

Yield = 46% (56 mg), 0.3 mmol scale. White solid. M.p. 101.0–102.7 °C.

IR (KBr): ν = 3060, 1771, 1579, 810, 720 cm^{-1}.

^{1}H NMR (400 MHz, $CDCl_3$): δ = 8.35 – 8.30 (m, 1H), 7.88 (d, *J* = 8.1 Hz, 1H), 7.66 – 7.52 (m, 3H), 7.30 (d, *J* = 8.7 Hz, 1H), 7.24 – 7.20 (m, 2H), 7.05 (d, *J* = 8.0 Hz, 2H), 2.28 (s, 3H) ppm.

^{19}F NMR (376 MHz, $CDCl_3$): δ = -83.63 (t, *J* = 4.1 Hz, 3F), -112.18 – -113.16 (m, 2F) ppm.

^{13}C NMR (100 MHz, $CDCl_3$): δ = 151.5 (t, *J* = 1.3 Hz), 141.3 (t, *J* = 29.6 Hz), 137.0, 132.7, 130.3, 130.0, 129.8, 129.3, 128.4, 127.1, 126.9, 125.1, 124.2, 121.0, 120.3, 118.5, 21.0 ppm; carbons corresponding to the C_2F_5 group cannot be identified due to C-F coupling.

HRMS m/z: calcd for $C_{21}H_{14}F_5OS$ $[M+H]^+$ 409.0680, found: 409.0685.

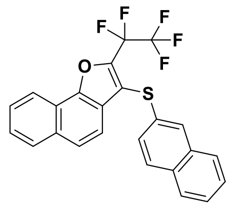

3-(Naphthalen-2-ylthio)-2-(perfluoroethyl)naphtho[1,2-*b*]furan (18):

Yield = 52% (69 mg), 0.3 mmol scale. White solid. M.p. 135.1–135.8 °C.

IR (KBr): ν = 3366, 1771, 1579, 1501, 810, 747 cm^{-1}.

^{1}H NMR (400 MHz, $CDCl_3$): δ = 8.36 (d, *J* = 8.1 Hz, 1H), 7.87 (d, *J* = 8.0 Hz, 1H), 7.81 – 7.73 (m, 2H), 7.71 – 7.61 (m, 3H), 7.59 – 7.52 (m, 2H), 7.46 – 7.38 (m, 2H), 7.37 – 7.27 (m, 2H) ppm.

^{19}F NMR (376 MHz, $CDCl_3$): δ = -82.90 – -84.28 (m, 3F), -112.06 – -113.36 (m, 2F) ppm.

^{13}C NMR (100 MHz, $CDCl_3$): δ = 151.6 (t, *J* = 1.0 Hz), 141.8 (t, *J* = 29.9 Hz), 133.6, 132.8, 132.0, 131.4, 129.0, 128.4, 127.7, 127.2, 127.2, 127.1, 127.0, 126.8, 126.3, 126.1, 125.3, 124.3, 121.0, 120.3, 118.5, 117.4 ppm; carbons corresponding to the C_2F_5 group cannot be identified due to

C-F coupling.

HRMS m/z: calcd for $C_{24}H_{14}F_5OS$ $[M+H]^+$ 445.0680, found: 445.0685.

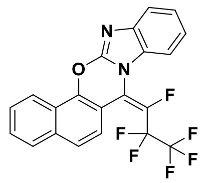

(*Z*)-7-(Perfluoropropylidene)-7*H*-benzo[4,5]imidazo[2,1-*b*]naphtho[2,1-*e*][1,3]oxazine (19):

Yield = 88% (111 mg), 0.3 mmol scale. Yellow solid. M.p. 234.8–235.1 ^{o}C.

IR (KBr): ν = 3064, 1676, 1623, 801, 745 cm^{-1}.

^{1}H NMR (400 MHz, $CDCl_3$): δ = 8.57 – 8.51 (m, 1H), 7.92 – 7.86 (m, 1H), 7.76 – 7.64 (m, 4H), 7.63 – 7.58 (m, 1H), 7.50 – 7.43 (m, 1H), 7.40 – 7.31 (m, 2H) ppm.

^{19}F NMR (376 MHz, $CDCl_3$): δ = -82.22 – -82.30 (m, 3F), -111.27 (d, *J* = 12.4 Hz, 2F), -125.75 (dq, *J* = 25.1, 12.5 Hz, 1F) ppm.

^{13}C NMR (100 MHz, $CDCl_3$): δ = 151.2, 147.3 (d, *J* = 4.8 Hz), 140.2, 135.0, 134.5 (t, *J* = 31.3 Hz), 130.2 (t, *J* = 2.5 Hz), 129.0, 127.7, 127.6, 124.5 (t, *J* = 0.9 Hz), 124.2, 123.0, 122.9 (d, *J* = 2.6 Hz), 122.5 (t, *J* = 7.9 Hz), 122.0, 119.3, 113.4 (d, *J* = 17.2 Hz), 108.4 (d, *J* = 2.4 Hz) ppm; carbons corresponding to the C_3F_6 group cannot be identified due to C-F coupling.

HRMS m/z: calcd for $C_{21}H_{11}F_6N_2O$ $[M+H]^+$ 421.0770, found: 421.0769.

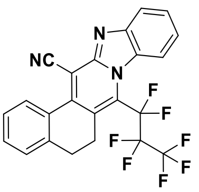

7-(Perfluoropropyl)-5,6-dihydrobenzo[*f*]benzo[4,5]imidazo[1,2-*b*]isoquinoline-14-carbonitrile (20):

Yield = 72% (100 mg), 0.3 mmol scale. Yellow solid. M.p. 257.9–258.2 ^{o}C.

IR (KBr): ν = 2901, 2230, 1602, 1475, 742, 721 cm^{-1}.

^{1}H NMR (400 MHz, $CDCl_3$): δ = 8.07 (d, *J* = 8.2 Hz, 1H), 7.95 (d, *J* = 7.7 Hz, 1H), 7.77 (d, *J* = 8.5 Hz, 1H), 7.63 – 7.47 (m, 3H), 7.39 (t, *J* = 7.4 Hz, 1H), 7.29 – 7.22 (m, 1H), 3.24 (d, *J* = 15.6 Hz, 1H), 2.94 (d, *J* = 14.2 Hz, 2H), 2.70 (t, *J* = 14.3 Hz, 1H) ppm.

^{19}**F NMR** (376 MHz, $CDCl_3$): δ = -79.59 (t, J = 10.4 Hz, 3F), -98.9 – -105.8 (m, 2F), -121.97 – -124.86 (m, 2F) ppm.

^{13}**C NMR** (100 MHz, $CDCl_3$): δ = 146.9, 145.9, 141.1, 139.5, 132.5 (t, J = 23.1 Hz), 132.1, 129.5, 127.9, 127.3, 126.6, 126.1, 125.4, 121.9, 121.7 (d, J = 2.8 Hz), 121.4, 115.9, 112.9, 102.0 (t, J = 5.5 Hz), 28.3 (t, J = 1.5 Hz), 25.5 (m) ppm; carbons corresponding to the C_3F_7 group cannot be identified due to C-F coupling.

HRMS m/z: calcd for $C_{23}H_{13}F_7N_3$ $[M+H]^+$ 464.0992, found: 464.0995.

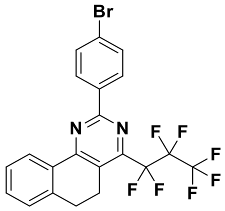

2-(4-Bromophenyl)-4-(perfluoropropyl)-5,6-dihydrobenzo[*h*]quinazoline (21):

Yield = 73% (111 mg), 0.3 mmol scale. White solid. M.p. 111.1–112.9 ^{o}C.

IR (KBr): ν = 3072, 2943, 1556, 1399, 1227, 930, 770, 743, 606 cm^{-1}.

^{1}**H NMR** (400 MHz, $CDCl_3$): δ = 8.53 (dd, J = 7.4, 1.7 Hz, 1H), 8.47 – 8.43 (m, 2H), 7.67 – 7.61 (m, 2H), 7.51 – 7.43 (m, 2H), 7.32 – 7.27 (m, 1H), 3.21 – 3.14 (m, 2H), 3.00 – 2.97 (m, 2H) ppm.

^{19}**F NMR** (376 MHz, $CDCl_3$): δ = -79.64 (t, J = 9.5 Hz, 3F), -110.17 (q, J = 9.6 Hz, 2F), -125.41 – -125.47 (d, J = 3.6 Hz, 2F) ppm.

^{13}**C NMR** (100 MHz, $CDCl_3$): δ = 162.6, 161.2, 152.1 (t, $J_{C\text{-}F}$ = 25.3 Hz), 139.4, 135.7, 132.0, 131.9, 131.8, 129.8, 127.9, 127.5, 126.4, 126.3, 125.9, 26.8, 22.9 (m) ppm; carbons corresponding to the C_3F_7 group cannot be identified due to C-F coupling.

HRMS m/z: calcd for $C_{21}H_{13}BrF_7N_2$ $[M+H]^+$ 505.0145, found: 505.0163.

Supplemental References

Frisch, M. J.; et al (2013). Gaussian 09, Revision D.01; Gaussian, Inc.: Wallingford, CT.

Becke, A. D. (1988). Density-Functional Exchange-Energy Approximation with Correct Asymptotic Behaviour. *Phys. Rev. A: At., Mol., Opt. Phys. 38*, 3098-3100.

Lee, C., Yang, W., and Parr, R. G. (1988). Development of the Colle-Salvetti Correlation-Energy Formula into a Functional of the Electron Density. *Phys. Rev. B: Condens. Matter Mater. Phys. 37*, 785-789.

Becke, A. D. (1993). Density-Functional Thermochemistry. III. The Role of Exact Exchange. *J. Chem. Phys. 98*, 5648-5652.

Weigend, F., and Ahlrichs, R. (2005). Balanced Basis Sets of Split Valence, Triple Zeta Valence and Quadruple Zeta Valence Quality for H to Rn: Design and Assessment of Accuracy. *Phys. Chem. Chem. Phys. 7*, 3297-3305.

Tomasi, J., and Persico, M. (1994). Molecular Interactions in Solution: An Overview of Methods Based on Continuous Distributions of the Solvent. *Chem. Rev. 94*, 2027-2094.

Grimme, S., Antony, J., Ehrlich, S., and Krieg, H. (2010). A Consistent and Accurate *ab initio* Parametrization of Density Functional Dispersion Correction (DFT-D) for the 94 Elements H-Pu. *J. Chem. Phys. 132*, 154104.

Goerigk, L., and Grimme, S. (2011). Efficient and Accurate Double-Hybrid-Meta-GGA Density Functionals—Evaluation with the Extended GMTKN30 Database for General Main Group Thermochemistry, Kinetics, and Noncovalent Interactions. *J. Chem. Theory Comput. 7*, 291-309.

Legault, C. Y. (2009). CYLview, version 1.0b; Universitéde Sherbrooke: Quebec, Canada; http://www.cylview.org.

Pham, P. V., Nagib, D. A., and MacMillan, D. W. C. (2011). Photoredox Catalysis: A Mild, Operationally Simple Approach to the Synthesis of α-Trifluoromethyl Carbonyl Compounds. *Angew. Chem. Int. Ed. 50*, 6119–6122.

Su, X., Huang, H., Yuan, Y., and Li, Y. (2017). Radical Desulfur-Fragmentation and Reconstruction of Enol Triflates: Facile Access to α-Trifluoromethyl Ketones. *Angew. Chem., Int. Ed. 56*, 1338–1341.

Xie, T., Zhang, Y.-W., Liu, L.-L., Shen, Z.-L., Loh, T.-P., and Chu, X.-Q. (2018). Polycyclic Heteroaromatic Ring Construction Driven by Silver/Cobalt Co-Catalyzed Desulfonylative and Defluorinative Fragment-Recombination of Enol Nonaflates with Amidines. *Chem. Commun.* *54*, 12722–12725.
